# Diazo Transfer From Nitrous Oxide Employing Phosphorus Ylides

**DOI:** 10.1002/anie.3352042

**Published:** 2026-04-19

**Authors:** Jhen‐Kuei Yu, Jan Niclas Ludwig, Patrick Wolf Antoni, David Tymann, Max Martin Hansmann

**Affiliations:** ^1^ Fakultät für Chemie und Chemische Biologie Technische Universität Dortmund Dortmund Germany

**Keywords:** diazo compounds, heterocycles, nitrogen oxides, one‐pot reactions, ylides

## Abstract

The synthesis of diazo‐containing molecules traditionally relies on hazardous reagents such as azides or hydrazines, which restrict their practicality, especially for large‐scale applications. Herein, we report a novel strategy for diazo transfer utilizing nitrous oxide (N_2_O) fixation at a phosphorus ylide. This method enables the efficient synthesis of valuable organic building blocks under mild, hydrazine‐free conditions. The one‐pot approach provides a safer and more practical route to diazo compounds, which can be trapped directly. This facile pathway gives access to a broad range of dinitrogen‐containing compounds, including 1,2,3‐triazolo heterocycles and (macrocyclic) aldazines. We additionally disclose a new phthalazine heterocycle synthesis utilizing bis‐P‐ylides and N_2_O. Both intra‐ and intermolecular trapping are investigated, which establishes N_2_O fixation as a powerful tool in synthetic methodology.

## Graphical abstract

A phosphorus ylide‐mediated strategy achieves the direct fixation of nitrous oxide (N_2_O) to access diazo compounds, which after intra‐ or intermolecular trapping, afford N_2_‐containing organic building blocks. This approach provides an alternative to hazardous diazo‐transfer reagents and establishes N_2_O fixation as a sustainable methodology.

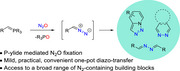

1

With the escalating urgency of climate change, nitrous oxide (N_2_O) has emerged as a molecule of significant environmental and chemical interest [1, 2, 3, 4, 5, 6, 7]. As a potent greenhouse gas, N_2_O presents both challenges and opportunities for synthetic chemistry [8]. Its high stability allows for safe handling, yet its inertness severely limits its reactivity and application in synthetic chemistry [9, 10, 11, 12, 13, 14]. Although N_2_O is thermodynamically regarded as a powerful oxidant, most successful transformations to date require harsh conditions, highlighting the necessity for kinetic activation strategies [11, 12, 13, 14]. Recent studies have demonstrated some low‐valent metal complexes and main group species enable N_2_O fixation and selective oxygen‐atom transfer (OAT) under mild conditions [15, 16, 17, 18, 19, 20, 21]. For instance, recent reports show metal‐free OAT achieved by photoinduced activation [22] and by P(III)/P(V) = O redox catalysis [23].

Compared to OAT, N_2_‐transfer remains underdeveloped in organic synthesis [24]. The most common activation of N_2_O toward N_2_‐transfer is based upon the addition of strong carbon nucleophiles such as organolithium compounds, first reported in 1953 [25]. However, these reactions often suffer from low yields and diminished selectivity, affording azo products (**I**) (Scheme 1A) [26, 27, 28, 29, 30, 31]. Recently, intramolecular trapping was described by Cui and coworkers to afford benzotriazin‐4(3*H*)‐ones (**II**) and by Severin and coworkers to give triazolopyridines (**III**) in good yields (Scheme 1A,B) [32, 33]. Along with the N_2_O fixation under Wislicenus’ conditions [34], Koga and Anselme demonstrated in 1968 that the methodology could be applied to aryl azides [35]. Employing substituted lithium amides, the N_2_O addition product was isolated by Severin and co‐workers, which could be further functionalized to give triazenes [36]. In a metal‐free approach, *N*‐heterocyclic carbenes have been shown to covalently capture N_2_O [37, 38], enabling access to cationic azo dyes [39]. Severin and co‐workers also demonstrated that highly polarized exocyclic olefins can add to N_2_O (**IV**) producing organic super‐electron‐donors (**V**) [40]. In 2021, the Severin group and us independently achieved the synthesis and isolation of *N*‐heterocyclic diazoalkenes (**VI**) via direct N_2_‐transfer from N_2_O employing a related addition/elimination process [41, 42].

**SCHEME 1:**
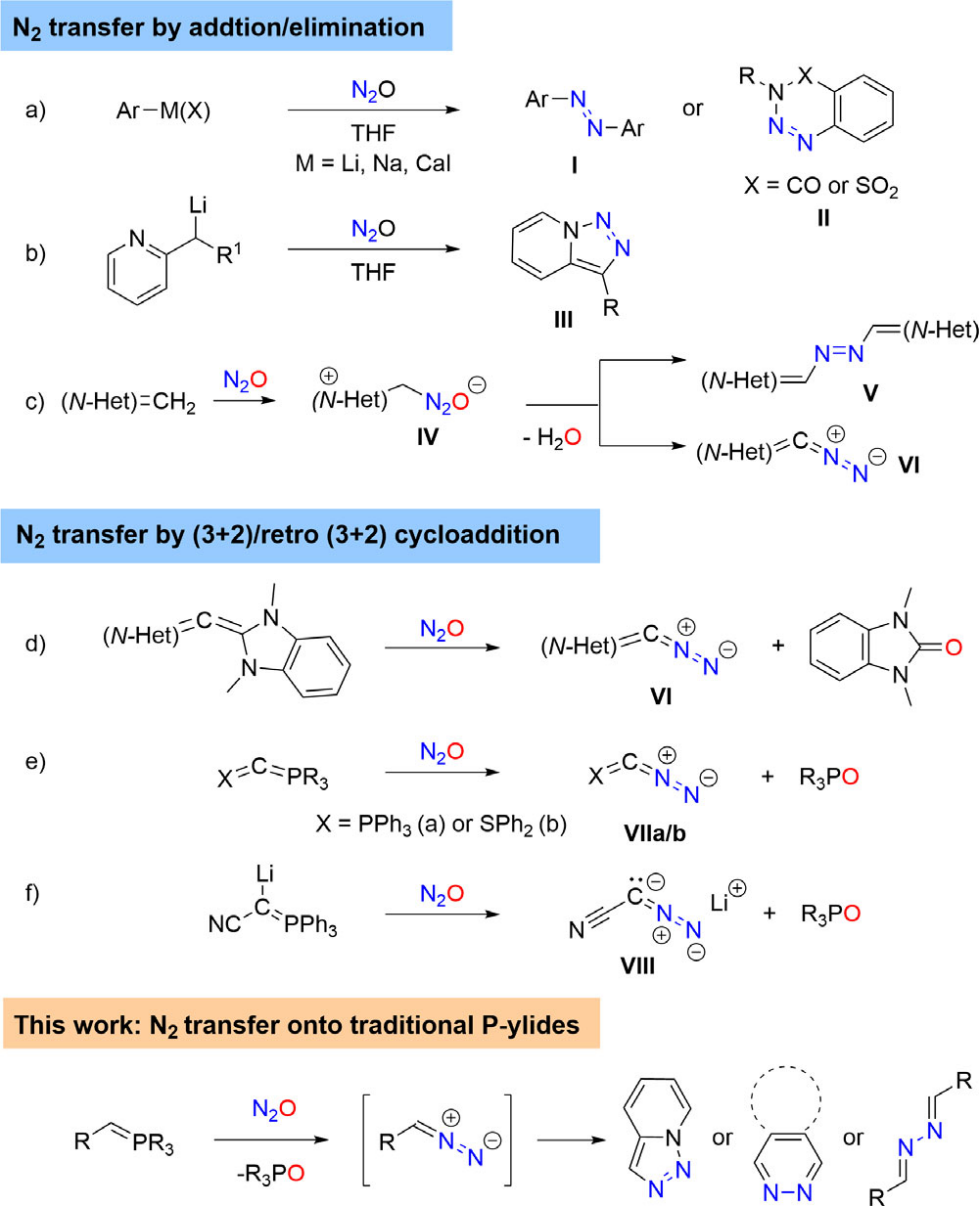
Literature examples for N_2_‐transfer employing nitrous oxide.

Recently, our group reported the synthesis of unsaturated diazo reagents, including new classes of diazoalkenes (**VI**), phosphorus (**VIIa**), and sulfur diazo reagents (**VIIb**) via an exchange of carbene (**VI**) or PPh_3_ (**VII**) and N_2_ fragments (Scheme 1D,E) [43, 44, 45]. A related PPh_3_/N_2_‐exchange strategy was also utilized in the reaction of metalated P‐ylides to give the corresponding metalated diazo compounds by Gessner and coworkers [46, 47]. Remarkably, such methodology allowed the synthesis of structurally simple diazo‐containing anions such as [NCCN_2_]^─^ (**VIII**) (Scheme 1F) [45, 47, 48] equivalents. All of these recent reports share a common mechanistic feature of N_2_O activation triggered by a (3 + 2) cycloaddition and cycloreversion sequence. Despite the thermodynamical propensity for triphenyl phosphine oxide or urea cleavage, the use of strong nucleophiles such as metalated ylides or carbon(0) compounds remains necessary. Based on these recent developments, we sought to investigate whether structurally simple phosphorus ylides could directly be utilized to incorporate the N_2_‐fragment into valuable organic building blocks.

There is significant interest in developing safer methods for diazo compound synthesis, since their conventional preparation usually involves toxic and explosive hydrazines or azides. Although effort has been devoted to the design of less explosive azides [49, 50, 51], they ultimately rely on the toxic and explosive sodium azide produced from N_2_O via the Wislicenus process. Hence, a direct diazo transfer using inert N_2_O as a chemical feedstock should shorten synthesis routes and avoid intermediates with pronounced safety hazards. A solitary example published in 1964 indicates that N_2_O can react with the phosphorus ylide Ph_3_P═CH_2_ to form diazomethane [52, 53]. However, detailed evidence remains limited, and the formation of diverse side products, including isodiazomethane, contributes to the poor yield (20%–25%). Despite explicit statements that higher diazoalkanes were non‐accessible, the origin of this limitation remained unclear. Herein, we address this issue, seek to overcome the restriction, and provide mechanistic insights.

To begin our investigation, we targeted the in situ preparation of diazoalkanes from phosphorus ylides followed by an intramolecular trapping. We selected 2‐pyridyl diazoalkanes, which are known to undergo fast intramolecular cyclization to afford triazolopyridines (Scheme 2) [32, 33, 54, 55, 56]. A standard deprotonation procedure of the phosphonium salt precursor (**1**) was performed using selected bases at ambient temperature (see the Supporting Information) [57, 58]. Upon replacing the argon atmosphere with N_2_O, a screen of conditions was performed to optimize the diazo‐transfer giving the parent triazolopyridine **3a** (for details, see the Supporting Information). Notably, the use of potassium *tert*‐butoxide at 60°C for 48 h promoted the formation of **3a** in nearly quantitative yield. To confirm the thermal requirement, the N_2_‐transfer was performed at 50°C, which led to a significant decrease in reaction rate (96 h, 75% yield). In contrast, the use of tri‐*n*‐butyl phosphonium salt **2a** resulted in a quantitative yield at room temperature in 12 h, suggesting that more electron‐rich phosphorus ylides effectively promote the diazo transfer. The milder conditions encouraged us to perform the reaction on a larger scale, affording the triazolopyridine **3a** in 89% isolated yield (530 mg). Nevertheless, given that quantitative yields with minimal side reactions were achieved at 60°C, we proceeded with further investigations with triphenyl phosphonium salts **1** due to the isolation complexity of tri‐*n*‐butyl phosphonium salt **2**. To our satisfaction, this method demonstrates excellent efficiency, accommodating a broad range of substituted pyridines, including bromo‐substituted pyridine (**3b**‐**3c**), *p*‐methoxy pyridine (**3d**), *o*‐methylpyridine (**3e**), as well as different quinolines (**3f**‐**3i**), all of which afforded high yields (86%‐99%) (Scheme 2). In addition, weak electron‐withdrawing groups, such as chlorine, were found to be compatible with these reaction conditions (**3** **g**). To further expand the synthetic utility, other nitrogen‐containing heterocycles were inspected, aiming to access a broader library of triazolo‐annulated building blocks. Gratifyingly, a wide variety of heterocycles, including quinoxaline (**3j**), naphthyridine (**3k**), and pyrimidines (**3l**, **3o**), successfully furnished the desired triazolo‐fused products in 63% up to 83% yields. The here‐presented structural flexibility presents a distinct benefit relative to the lithiation strategy described by Severin and co‐workers, in which selectivity issues might occur from lithiation on the electron‐deficient heterocycles instead of the required 2‐alkyl position [33]. Furthermore, fully substituted P‐ylides gave sterically encumbered products in satisfactory yields (**3m** and **3n**). Interestingly, this method demonstrates the formation of bis‐heteroarenes, which structurally resemble bidentate ligands (**3n**‐**3p**) [59, 60, 61]. Notably, tri‐*n*‐butyl phosphonium salts (**2**) were employed instead of triphenyl phosphonium salt (**1**) in selected examples due to the difficulty of purification caused by phosphine oxide removal (**3k**, **3p**), enhancing nucleophilicity (**3l**‐**3n**), and the thermal instability of the product (**3p**) requiring mild reaction conditions.

**SCHEME 2:**
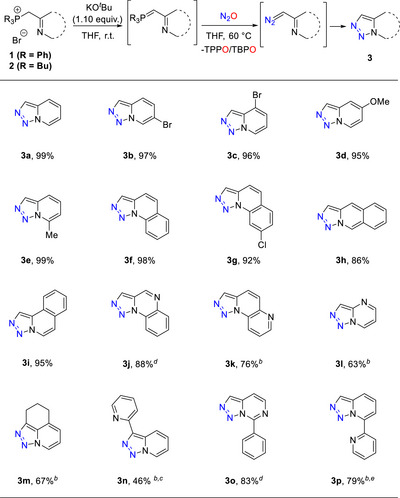
Substrate scope of 1,2,3‐triazolo heterocycle synthesis. Unless otherwise noted, the reactions were carried out under inert atmosphere in anhydrous THF (5 mL) on a scale of 0.20 mmol phosphonium salt **1**. Deprotonation was performed at ambient temperature with 1.1 equiv. of KO*^t^* Bu, and the diazo transfer was performed at 60°C under an N_2_O atmosphere for 48 h. Isolated yields are reported. ^(b)^ Tri‐*n*‐butyl phosphonium salt **2** was employed. ^(c)^ *n*‐BuLi was used in the deprotonation process. ^(d)^ The reaction was performed at 70°C. ^(e)^ The Reaction was performed at ambient temperature.

In order to analyze the N_2_‐transfer mechanism, DFT calculations were conducted comparing the influence of the two P‐ylides (PPh_3_ and PMe_3_). Calculations support the rate‐determining step to be the nucleophilic addition of the P‐ylide to the outer N‐atom of N_2_O which is lower in energy for PMe_3_ (ΔG* = 21.1 kcal/mol) than PPh_3_ (ΔG* = 24.0 kcal/mol) in agreement with the experimental data (Figure 1). Addition of N_2_O to the P‐ylide triggers a stepwise cycloaddition/cycloreversion sequence (for details, see the Supporting Information).

**FIGURE 1:**
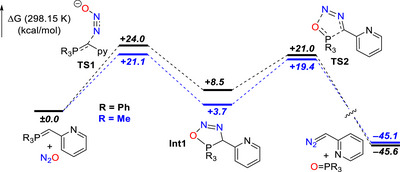
Simplified DFT‐calculated energy profiles [PBE0‐D3(BJ)/def2‐TZVP/SMD(THF)] illustrating the effect of phosphine motifs on the reaction pathway; for a detailed mechanistic picture, see the Supporting Information.

Upon investigating the possibility of a bidirectional N_2_‐transfer employing bis‐phosphorus ylides to access bistriazolopyrazine **6**, we experienced a different reaction outcome. Instead, double deprotonation of **4a** and treatment with N_2_O afforded pyrazino[2,3‐d]pyridazine **5a** in quantitative yield (Scheme 3A). Its connectivity was unambiguously verified by x‐ray diffraction [62]. This new reaction pathway can be rationalized by an intramolecular nucleophilic attack of the P‐ylide onto the newly formed diazoalkane (Scheme 3B). Monitoring the reaction by in situ NMR shows, in agreement with the postulated mechanism, the formation of a 1:1 mixture of Ph_3_PO/PPh_3_ derived from diazo transfer and elimination, respectively.

**SCHEME 3:**
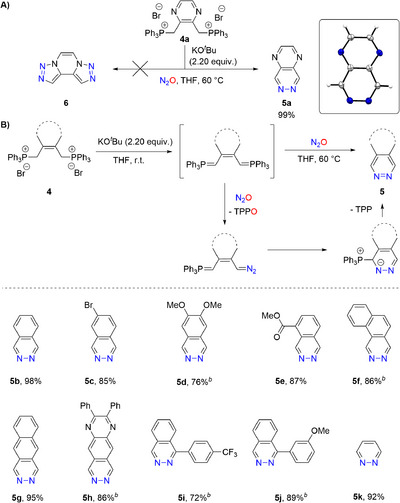
(A) N_2_‐transfer of bis P‐ylides to give phthalazines and pyridazine. (B) Postulated mechanism and substrate scope of diazine synthesis. ^(a)^ Unless otherwise noted, the reactions were carried out under N_2_O atmosphere in 5 mL anhydrous THF on a scale of 0.20 mmol phosphonium salt **1**. The deprotonation was performed at ambient temperature with 2.20 equiv. of KO*^t^* Bu and the diazo‐transfer at 60°C for 48 h. Isolated yields are shown. ^(b)^ Instead of triphenyl phosphonium salt, a tributylphosphonium salt was used. ^(c)^ The entire reaction process was performed at ambient temperature. Inset: x‐ray structure of **5a**; thermal elipsoids are shown at 50% probability.

To explore the scope of this novel phthalazine synthesis, a series of bis‐phosphonium salts **4** featuring π‐extended and both electron‐deficient and electron‐rich aromatic backbones were evaluated (Scheme 3; **5a**‐**5h**). In addition, 2‐substituted phthalazines were successfully achieved using this protocol (Scheme 3; **5i**‐**5j**). The high yields obtained across all examples demonstrate the robustness of new pyridazine/phthalazine synthesis to electronic variations (72%–99%). The transformation was expanded to a non‐aromatic backbone containing a *cis*‐alkene (**4k**), which also gave the parent pyridazine (**5k**) in 92% yield. It is worth noting that this methodology might be particularly interesting for ^15^N‐labeling employing mono or doubly ^15^N‐labeled N_2_O [63].

Next, the designed bis‐phosphonium salts **4l** and **4m** were synthesized and tested under the optimized diazo‐transfer conditions with the aim of promoting macrocyclization (Scheme 4). Remarkably, the ring size played a crucial role in dictating selectivity. When a C2‐linker was incorporated, formation of the 12‐membered ring (**5l**) predominated at 60°C, whereas no conversion was observed at room temperature. However, extending the linker by an additional carbon‐atom altered the pathway toward dimerization, giving a 26‐membered macrocycle (**5m’**) as the major product at room temperature, which could be structurally verified by x‐ray crystallography (Scheme 4) [62]. Moreover, the classical condensation approach employing hydrazine and the corresponding bis‐aldehyde failed to provide the desired product. Instead, the reaction resulted in a viscous mixture, suggesting competing side reactions such as oligomerization or polymerization. These findings underscore the robustness of the diazo‐transfer strategy presented herein.

**SCHEME 4:**
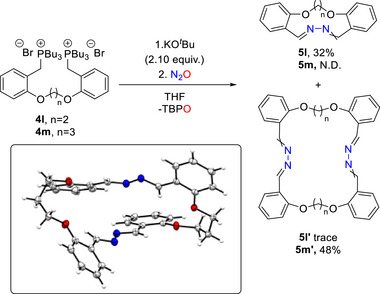
Macrocyclization employing N_2_‐transfer from N_2_O. Inset: solid‐state structure of **5m’**. Two molecules of THF and benzene were omitted for clarity. Thermal elipsoids shown with 50% probability.

Next, we examined the possibility of trapping the diazoalkane by an intermolecular reaction employing reactive alkenes and alkynes. Besides aromatic diazo compounds, we investigated isopropyl phosphonium salt **7a** in the three‐component assembly with excess norbornene, a well‐known highly reactive dipolarophile [64]. The formed transient alkyl diazoalkane could be intermolecularly trapped, resulting in the successful isolation of the multicyclic product **8** (Scheme 5) [65]. Furthermore, the possibility of utilizing cyclooctyne as an alternative trapping reagent enabled a three‐component assembly with **9a**, affording pyrazole **10** in 45% yield (Scheme 5).

**SCHEME 5:**
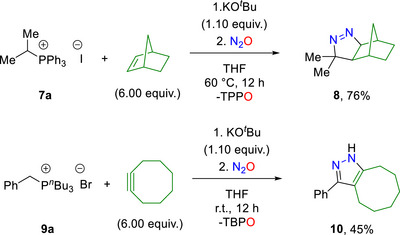
Intermolecular trapping for three component reactions employing alkenes and alkynes.

Finally, we investigated the N_2_‐transfer without applying any intramolecular or intermolecular trapping entities. Selected phosphonium bromides **7b**‐**7d** were subjected to the optimized diazo transfer protocol. Unfortunately, apart from expected azines **13**, stilbene derivatives **14** were as well observed in a non‐stereoselective fashion along with 12% of 1,2,3‐triazoles **12**. Previous studies have shown that arenecarbaldehyde azines **13** eliminate dinitrogen under strongly basic conditions (potassium *t*‐butoxide) at elevated temperature (110°C for 40 h) to produce stilbenes **14** [66]. Encouragingly, this decomposition can be prohibited in the presence of aryl diazomethanes, facilitating the formation of symmetrical triazoles **12** via (3 + 2) cycloaddition [66]. Herein, we modified the reaction conditions by adding excess (3.30 equiv.) of potassium *tert*‐butoxide instead of using 1.10 equivalents. To our delight, the (3+2) cycloaddition predominates over decomposition under these conditions (Scheme 6). Imine elimination produces symmetrical 1,2,3‐triazoles **12b**–**12d** with yields of 54% up to 86% (Scheme 6A).

**SCHEME 6:**
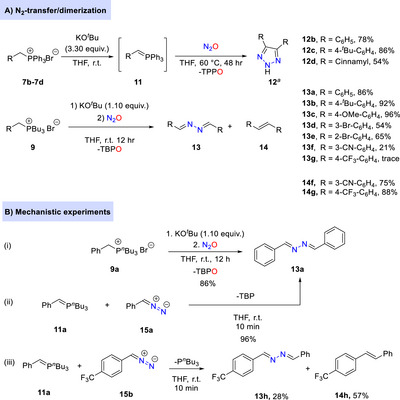
(A) N_2_‐transfer without trapping reagents. ^(a)^ The yield was calculated based on the consumption of three equivalents of phosphonium salt. (B) Mechanistic experiments employing ylides and diazo reagents.

In addition, the selective formation of azines **13** was targeted, exploiting tri‐*n*‐butylphosphonium salts **9** at ambient temperature to avoid dinitrogen loss [66]. Gratifyingly, the desired azine products **13** were isolated in good yields bearing various electron‐donating groups and halogen substituents (Scheme 6A). Notably, the incorporation of electron‐withdrawing groups drastically altered the reactivity, yielding (*E*)‐stilbene derivatives **14f** and **14g** with minor amounts of the corresponding azines **13f** and **13g**, respectively. To gain mechanistic insight into the diverging pathways of diazoalkane along azine, triazole, and olefin formation, we performed mechanistic control experiments (Scheme 6B and see the Supporting Information). We independently prepared the isolated diazoalkane **15a** as well as P‐ylide **11a**. Interestingly, both compounds reacted fast to give azine **13a** in high yields along with the elimination of tri‐*n*‐butyl phosphine [67, 68]. This finding supports azine formation through the combination of diazoalkane and P‐ylide instead of a slow denitrogenative dimerization of aryldiazomethanes. Clearly a faster trapping reaction is required to inhibit the consumption of diazoalkane by unreacted P‐ylide. Note, the previously observed formation of phosphine oxide instead of the corresponding phosphine can be rationalized by the facile room temperature oxidation of tri‐*n*‐butyl phosphine with N_2_O (see the Supporting Information). Lastly, we studied the control reaction between P‐ylide **11a** and diazoalkane **15b**. The corresponding asymmetric azine **13h** and the asymmetric (*E*)‐stilbene **14h** were obtained in a ca. 1:2 ratio in line with the selectivity observed (Scheme 6B).

With these results, we therefore could rationalize the difference from Rundel's work and our methodology [53]. In their work, deprotonation of CH_2_N_2_ by a more basic phosphorus ylide (Ph_3_P^+^‐CH_3_; pK_a_ ∼ 22) [69] predominates, generating the diazomethane anion, which is most likely reluctant to form azines. This assumption is in line with our recent work on carbodiphosphoranes and Gessner's work on metalated ylides. The anionic or anionic polarized character of these diazo compounds confers appreciable stability; the resulting diazo species are therefore less prone to azine formation [43, 44, 45, 46, 47]. In contrast, the significantly reduced basicity of the P‐ylide in this work renders deprotonation of RCH(N_2_) unfavorable, shifting the reaction manifold toward nucleophilic addition.

Of particular relevance, we demonstrate the direct transformation of azines into valuable heterocycles without intermediate isolation, emphasizing the convenience of this one‐pot N_2_‐transfer procedure. After azine formation under the typical reaction conditions, a crude mixture of the desired azine **13a** and tri‐*n‐*butylphosphine oxide can be used directly in subsequent transformations. For instance, cyclization was performed under oxidative conditions using bis(trifluoroacetoxy)iodo benzene (PIFA) to form 1,3,4‐oxadiazole **16**, adopting an established method [70]. Under our conditions, both the desired oxadiazole **16** and its intermediate **17** were obtained in moderate yields (Scheme 7). Additionally, a bromination using elemental bromine could be performed to afford a highly reactive yet versatile intermediate [71, 72, 73], which undergoes hydrolysis upon aqueous work‐up to generate oxadiazole **16** (Scheme 7). In the presence of methylamine, similar cyclization could also occur, presumably forming *N*‐methylated‐1,2,4‐triazole; however, isolation of this product proved challenging. Upon acidic workup, the demethylation can take place and result in isolable 1,2,4‐triazole **18** in moderate yield (Scheme 7).

**SCHEME 7:**
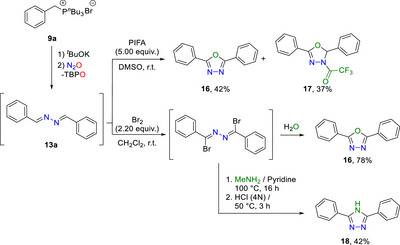
One‐pot N_2_‐transfer followed by heterocycle formation.

In summary, we have developed a mild and efficient diazo‐transfer methodology directly using nitrous oxide (N_2_O) as a N_2_‐source. Employing phosphorus ylides as key intermediates, N_2_‐fixation proceeds via a (3 + 2)/retro‐(3 + 2) cycloaddition generating only phosphine oxide as a byproduct. Computational data and mechanistic experiments are provided to verify each reaction step in this one‐pot transformation and differentiate this methodology from typical N_2_O covalent trapping strategies by metalation. Instead of isolating the diazo compound, substrate design and selection prove the higher diazoalkane is accessible in situ, allowing subsequent transformations to produce versatile organic building blocks. This methodology circumvents the use of hazardous compounds such as hydrazines or azides and provides operational simplicity. The high‐yielding, transition metal‐free, and mild conditions underscore the synthetic utility of this approach, positioning it as a valuable synthetic tool for N_2_‐fragment incorporation in heterocyclic building blocks, offering an environmentally conscious approach to synthetic methodology.

## Conflicts of Interest

The authors declare no conflicts of interest.

## Supporting information




**Supporting File**: anie72274‐sup‐0001‐SuppMat.pdf.

## Data Availability

The data that supports the findings of this study are available in the supplementary material of this article.
