# Supplementary material for: Diazo Transfer From Nitrous Oxide Employing Phosphorus Ylides
Source: Angew Chem Int Ed Engl. 2026 Apr 19;65(22):e3352042. doi: 10.1002/anie.3352042 (PMC13206431; doi:10.1002/anie.3352042)
Supplement: Supplementary file 1 — Supporting File: anie72274‐sup‐0001‐SuppMat.pdf. [file ANIE-65-e3352042-s001.pdf]

Supporting information

for

Diazo Transfer from Nitrous Oxide Employing Phosphorus Ylides

Jhen-Kuei Yu, Jan Niclas Ludwig, Patrick Wolf Antoni, David Tymann, and Max Martin Hansmann

Technische Universität Dortmund,

Fakultät für Chemie und Chemische Biologie

Otto-Hahn-Str. 6; 44227 Dortmund, Germany

Table of Contents

1. **General procedure** 2
2. **Optimization** 3
3. **Synthetic procedures** 4
4. **X-ray characterization data** 73
5. **Mechanism study** 76
6. **Computational details** 87
7. **NMR spectra** 138
8. **References** 340

1. General procedure

All reagents and solvents were obtained from commercial suppliers and used without further purification unless otherwise noted. Solvent purification followed the procedures described in *Purification of Laboratory Chemicals*, 6th Ed. (ISBN: 978-1-85617-567-8). Dichloromethane, tetrahydrofuran, acetonitrile, and toluene were dried using an M. Braun Solvent Purification System (SPS-800). Deuterated solvents (CD_2Cl_2, C_6D_6, CD_3CN) and anhydrous 1,4-dioxane were distilled from sodium or calcium hydride, degassed, and stored over molecular sieves. Anhydrous solvents were stored under argon. All reactions sensitive to moisture or air were performed under an inert atmosphere of argon or nitrogen using standard Schlenk techniques or in a glovebox (GS MEGA). Glassware was dried at 120 °C prior to use. N_2O (purity 5.0) was used as received from Messer. Analytical thin-layer chromatography (TLC) was carried out on Merck silica gel 60 F_{254} plates (alumina-backed) and visualized under UV light or by staining (ceric ammonium molybdate, $KMnO_4$, or *p*-anisaldehyde). Flash column chromatography was performed using silica gel 60 (40-63 μm, Merck). Melting points were determined using a Büchi M-560 apparatus and are reported uncorrected. Infrared (IR) spectra were recorded as thin films using a Bruker Alpha II or JASCO FT-IR 6200 spectrometer; only characteristic absorptions are reported. Mass spectra were recorded using Finnigan MAT 8200 (EI), Finnigan MAT 95 (ESI), Bruker APEX III FT-MS (7 T), or LTQ-Orbitrap-XL (HESI). NMR spectra (^{1}H, ^{13}C, ^{31}P and ^{19}F) were recorded on Bruker AV 600 Avance III HD, or Bruker Avance III 500 MHz instruments. Chemical shifts (δ) of ^{1}H, ^{13}C spectra are reported in ppm and referenced to residual solvent signals: $CDCl_3$ (δ = 7.26 ppm for ^{1}H, 77.16 ppm for ^{13}C), CD_2Cl_2 (δ = 5.32 ppm for ^{1}H, 53.8 ppm for ^{13}C), C_6D_6 (δ = 7.15 ppm for ^{1}H, 128.06 ppm for ^{13}C), CD_3CN (δ = 1.94 ppm for ^{1}H, 118.26 ppm for ^{13}C), DMSO-d_6 (δ = 2.50 ppm for ^{1}H, 39.52 ppm for ^{13}C). Coupling constants (J) are given in Hertz (Hz). All ^{13}C NMR spectra were recorded with ^{1}H decoupling unless otherwise noted.

2. Optimization

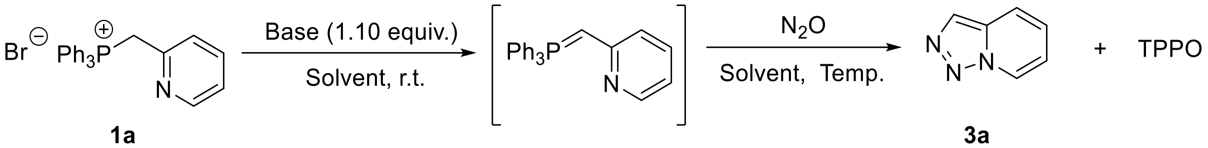

Entry	Solvent	Base	Temperature (°C)	Reaction time (h)	Ylide (%)
1	THF	NaH	60	48	35
2	1,4-Dioxane	NaH	60	48	37
3	MeCN	NaH	60	48	16
4	THF	KO*t*Bu	60	48	99
5	THF	KO*t*Bu	50	96	75
6[b]	THF	KO*t*Bu	r.t.	12	99
7[b,c]	THF	KO*t*Bu	r.t.	48	89

Table S1. Optimization of one-pot diazo-transfer reaction affording triazolopyridines. [a] Unless otherwise noted, reactions were carried out under an inert atmosphere using 5 mL of anhydrous solvent on a 0.20 mmol scale. Yields refer to isolated products. [b] Tributyl phosphonium salt **2a** was employed instead. [c] reaction was carried out on a 5.00 mmol scale in 10 mL of solvent.

3. Synthetic procedures

General procedure for benzylic halogenation (GP1)

The precursors for phosphonium salts **1b**, **1e**, **1g**, **1h**, **1i**, **1j**, **2k**, **2l**, and **2p**, which are not commercially available, were synthesized following a previously reported halogenation protocol.[1] The corresponding methyl-substituted heteroarene (1.00 equiv.) was dissolved in carbon tetrachloride, and *N*-bromosuccinimide (NBS, 1.10 equiv.) together with a radical initiator (benzoyl peroxide (BPO) or 2,2′-azobisisobutyronitrile (AIBN, 5.0 mol%). The reaction mixture was stirred at 80 °C under a nitrogen atmosphere. Reaction progress was monitored by TLC.

Upon completion, the reaction mixture was concentrated under reduced pressure and the residue was diluted with dichloromethane and washed with aqueous sodium thiosulfate solution. The organic layer was separated, dried over Na_2SO_4, and concentrated, and the crude product was purified by flash column chromatography on silica gel (EtOAc/CyHex). Owing to the instability of the halogenated intermediates, they were used immediately in subsequent transformations without further characterization. Full characterization was performed on the corresponding phosphonium salts **1** and **2**.

5-bromo-2-(bromomethyl)pyridine

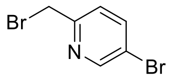

According to **GP1**, 5-bromo-2-methylpyridine (860 mg, 5.00 mmol) was dissolved in carbon tetrachloride (5 mL), To the solution, 2,2′-azobisisobutyronitrile (AIBN, 41.1 mg, 0.25 mmol) and *N*-bromosuccinimide (NBS, 979 mg, 5.50 mmol) were added. Reaction completion was confirmed by TLC. The mixture was concentrated under reduced pressure and purified by column chromatography on silica gel (EtOAc/CyHex = 1:20, R_f = 0.34). The title compound was obtained as a pink oil (364 mg, 29% yield) and was used immediately in subsequent transformations without further characterization.

2-(bromomethyl)-6-methylpyridine

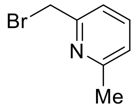

According to **GP1**, 2,6-lutidine (536 mg, 5.00 mmol) was dissolved in carbon tetrachloride (5 mL). To the solution, 2,2′-azobisisobutyronitrile (AIBN, 41.1 mg, 0.25 mmol) and *N*-bromosuccinimide (NBS, 979 mg, 5.50 mmol) were added. Reaction completion was confirmed by TLC. The mixture was concentrated under reduced pressure and purified by column chromatography on silica gel (EtOAc/CyHex = 1:20, R_f = 0.34). The title compound was obtained as a pink oil (372 mg, 40% yield) and was used immediately in subsequent transformations without further characterization.

2-(bromomethyl)-7-chloroquinoline

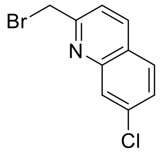

According to **GP1**, 7-chloro-2-methylquinoline (888 mg, 5.00 mmol) was dissolved in carbon tetrachloride (5 mL). To the solution, 2,2′-azobisisobutyronitrile (AIBN, 41.1 mg, 0.25 mmol) and *N*-bromosuccinimide (NBS, 979 mg, 5.50 mmol) were added. Reaction completion was confirmed by TLC. The mixture was concentrated under reduced pressure and purified by column chromatography on silica gel (EtOAc/CyHex = 1:20, R_f = 0.34). The title compound was obtained as a pink solid (577 mg, 45% yield) and was used immediately in subsequent transformations without further characterization.

3-(bromomethyl)isoquinoline

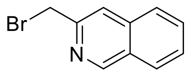

According to **GP1**, 3-methylisoquinoline (716 mg, 5.00 mmol) was dissolved in carbon tetrachloride (5 mL). To the solution, 2,2′-azobisisobutyronitrile (AIBN, 41.1 mg, 0.25 mmol) and *N*-bromosuccinimide (NBS, 979 mg, 5.50 mmol) were added. Reaction completion was confirmed by TLC. The mixture was concentrated under reduced pressure and purified by column chromatography on silica gel (EtOAc/CyHex = 1:20, R_f = 0.36). The title compound was obtained as an off-white solid (637 mg, 57% yield) and was used immediately in subsequent transformations without further characterization.

1-(bromomethyl)isoquinoline

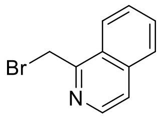

According to **GP1**, 1-methylisoquinoline (1.43 g, 10.0 mmol) was dissolved in carbon tetrachloride (10 mL). To the solution, 2,2′-azobisisobutyronitrile (AIBN, 82.2 mg, 0.50 mmol) and *N*-bromosuccinimide (NBS, 1.96 g, 11.0 mmol) were added. Reaction completion was confirmed by TLC. The mixture was concentrated under reduced pressure and purified by column chromatography on silica gel (EtOAc/CyHex = 1:10, R_f = 0.31). The title compound was obtained as a violet solid (599 mg, 27% yield) and was used immediately in subsequent transformations without further characterization.

2-(bromomethyl)quinoxaline

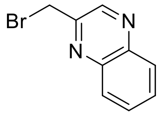

According to **GP1**, 2-methylquinoxaline (1.44 g, 10.0 mmol) was dissolved in carbon tetrachloride (10 mL). To the solution, benzoyl peroxide (BPO, 30%, 404 mg, 0.50 mmol) and *N*-bromosuccinimide (NBS, 1.96 g, 11.0 mmol) were added. Reaction completion was confirmed by TLC. The mixture was concentrated under reduced pressure and purified by column chromatography on silica gel (EtOAc/CyHex = 1:4, R_f = 0.35). The title compound was obtained as a grey solid (625 mg, 28% yield) and was used immediately in subsequent transformations without further characterization.

2-(bromomethyl)-1,5-naphthyridine

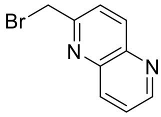

According to **GP1**, 2-methyl-1,5-naphthyridine (721 mg, 5.00 mmol) was dissolved in carbon tetrachloride (5 mL). To the solution, benzoyl peroxide (BPO, 30%, 202 mg, 0.25 mmol) and *N*-bromosuccinimide (NBS, 979 mg, 5.50 mmol) were added. Reaction completion was confirmed by TLC. The mixture was concentrated under reduced pressure and purified by column chromatography on silica gel (EtOAc/CyHex = 1:2, R_f = 0.26). The title compound was obtained as a pink solid (112 mg, 11% yield) and was used immediately in subsequent transformations without further characterization.

2-(bromomethyl)pyrimidine

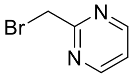

According to **GP1**, 2-methyl-pyrimidine (941 mg, 10.0 mmol) was dissolved in carbon tetrachloride (10 mL). To the solution, 2,2′-azobisisobutyronitrile (AIBN, 82.2 mg, 0.50 mmol) and *N*-bromosuccinimide (NBS, 1.96 g, 11.0 mmol) were added. Reaction completion was confirmed by TLC. The mixture was concentrated under reduced pressure and purified by column chromatography on silica gel (EtOAc/CyHex = 1:1, R_f = 0.28). The title compound was obtained as a yellowish solid (1.18 g, 68% yield) and was used immediately in subsequent transformations without further characterization.

6-(bromomethyl)-2,2'-bipyridine

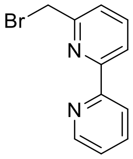

According to **GP1**, 6-methyl-2,2'-bipyridine (851 mg, 5.00 mmol) was dissolved in carbon tetrachloride (5 mL). To the solution, benzoyl peroxide (BPO, 30%, 202 mg, 0.25 mmol) and *N*-bromosuccinimide (NBS, 979 mg, 5.50 mmol) were added. Reaction completion was confirmed by TLC. The mixture was concentrated under reduced pressure and purified by column chromatography on silica gel (EtOAc/CyHex = 1:4, R_f = 0.35). The title compound was obtained as a grey solid (386 mg, 31% yield) and was used immediately in subsequent transformations without further characterization.

Preparation for precursor of phosphonium salt 1d, 2m, 2n

The precursors for phosphonium salts **1d**, **2m**, and **2n**, were synthesized via an alternative route resembling the Hell-Volhard-Zelinsky bromination. A solution of the corresponding alcohol (1.00 equiv.) in dichloromethane was prepared, and phosphorus tribromide (PBr_3, 0.70 equiv.) was added dropwise at 0 °C. The reaction mixture was then allowed to warm to room temperature and stirred for an additional 3 hours. Upon completion, as confirmed by TLC analysis, an aqueous workup was performed. The mixture was extracted with dichloromethane (3× 30 mL), and the combined organic layers were concentrated under reduced pressure. The crude product was purified by flash column chromatography (EtOAc/CyHex) to afford the corresponding brominated intermediate, which was immediately subjected to the subsequent transformation due to its instability. Full characterization was carried out on the corresponding phosphonium salts **1** and **2**.

2-(bromomethyl)-4-methoxypyridine

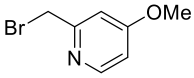

(4-Methoxypyridin-2-yl)-methanol (139 mg, 1.00 mmol) was dissolved in dichloromethane (2 mL). To the solution, phosphorus tribromide (PBr_3, 65.7 µL, 0.70 mmol) was added dropwise at 0 °C. Reaction completion was confirmed by TLC. The mixture was concentrated under reduced pressure and purified by column chromatography on silica gel (EtOAc/CyHex = 1:3, R_f = 0.22). The title compound was obtained as a yellowish oil (143 mg, 71% yield) and was used immediately in subsequent transformations without further characterization.

8-bromo-5,6,7,8-tetrahydroquinoline

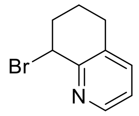

5,6,7,8-tetrahydroquinoline-8-ol (746 mg, 5.00 mmol) was dissolved in dichloromethane (10 mL). To the solution, phosphorus tribromide (PBr_3, 329 µL, 3.50 mmol) was added dropwise at 0 °C. Reaction completion was confirmed by TLC. The mixture was concentrated under reduced pressure and purified by column chromatography on silica gel (EtOAc/CyHex = 1:20, R_f = 0.23). The title compound was obtained as a yellowish oil (357 mg, 34% yield) and was used immediately in subsequent transformations without further characterization.

2,2'-(bromomethylene)dipyridine

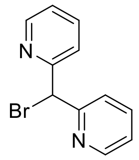

bis(pyridin-2-yl)methanol (372 mg, 2.00 mmol) was dissolved in dichloromethane (2 mL). To the solution, phosphorus tribromide (PBr_3, 132 µL, 1.40 mmol) was added dropwise at 0 °C. Reaction completion was confirmed by TLC. The mixture was concentrated under reduced pressure and purified by column chromatography on silica gel (EtOAc/CyHex = 1:10, R_f = 0.32). The title compound was obtained as an off-white solid (329 mg, 66% yield) and was used immediately in subsequent transformations without further characterization.

4-(bromomethyl)-2-phenylpyrimidine

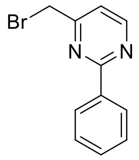

The 4-(bromomethyl)-2-phenylpyrimidine, precursor for phosphonium salt **1o**, was synthesized via Appel bromination. A solution of the (2-phenylpyrimidin-4-yl)methanol (186 mg, 1.00 equiv., 1.00 mmol) in dichloromethane (5 mL) was prepared. To this solution, carbon tetrabromide (CBr_4, 660 mg, 1.99 equiv., 1.99 mmol) was added and the mixture was cooled to 0 °C. Subsequently, triphenylphosphine (289 mg, 1.10 equiv., 1.10 mmol) was added at 0 °C, and the reaction was allowed to warm to room temperature and stirred for an additional 1 hour. Upon full conversion as confirmed by TLC analysis, the reaction underwent aqueous workup. The mixture was extracted with dichloromethane (3× 30 mL), and the combined organic layers were concentrated under reduced pressure. The crude product was purified by flash column chromatography (EtOAc/CyHex = 2:3, R_f = 0.29) to yield the title compound as yellowish solid (135 mg, 54% yield) which was immediately

subjected to the subsequent transformation due to its instability. Full characterization was carried out on the corresponding phosphonium salts **1o**.

Preparation for precursor of bis-phosphonium salt 4c, and 4e-4h

The precursors for phosphonium salts **4c**,and **4e-4h** were synthesized following a previously reported halogenation protocol.[2] The corresponding dimethyl-substituted heteroarene (1.00 equiv.) was dissolved in carbon tetrachloride, and *N*-bromosuccinimide (NBS, 2.10 equiv.) together with 2,2′-azobisisobutyronitrile (AIBN, 10.0 mol%). The reaction mixture was stirred at 80 °C under a nitrogen atmosphere. Reaction progress was monitored by TLC.

Upon completion, the reaction mixture was concentrated under reduced pressure and the residue was diluted with dichloromethane and washed with aqueous sodium thiosulfate solution. The organic layer was separated, dried over Na_2SO_4, and concentrated, and the crude product was purified by flash column chromatography on silica gel (EtOAc/CyHex). Owing to the instability of the halogenated intermediates, they were used immediately in subsequent transformations without further characterization. Full characterization was performed on the corresponding bis-phosphonium salts **4**.

4-bromo-1,2-bis(bromomethyl)benzene

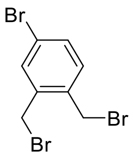

4-bromo-*o*-xylene (370 mg, 2.00 mmol) was dissolved in carbon tetrachloride (5 mL). To the solution, 2,2′-azobisisobutyronitrile (AIBN, 32.8 mg, 0.20 mmol) and *N*-bromosuccinimide (NBS, 748 mg, 4.20 mmol) were added. Reaction completion was confirmed by TLC. The mixture was concentrated under reduced pressure and purified by column chromatography on silica gel (EtOAc/CyHex = 1:20, R_f = 0.41). The title compound was obtained as a white solid (535 mg, 78% yield) and was used immediately in subsequent transformations without further characterization.

methyl 2,3-bis(bromomethyl)benzoate

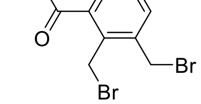

Methyl 2,3-dimethylbenzoate (328 mg, 2.00 mmol) was dissolved in carbon tetrachloride (5 mL). To the solution, 2,2′-azobisisobutyronitrile (AIBN, 32.8 mg, 0.20 mmol) and *N*-bromosuccinimide (NBS, 748 mg, 4.20 mmol) were added. Reaction completion was confirmed by TLC. The mixture was concentrated under reduced pressure and purified by column chromatography on silica gel

(EtOAc/CyHex = 1:20, R_f = 0.36). The title compound was obtained as a yellowish oil (566 mg, 88% yield) and was used immediately in subsequent transformations without further characterization.

1,2-bis(bromomethyl)naphthalene

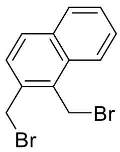

1,2-dimethylnaphthalene (313 mg, 2.00 mmol) was dissolved in carbon tetrachloride (5 mL). To the solution, 2,2′-azobisisobutyronitrile (AIBN, 32.8 mg, 0.20 mmol) and *N*-bromosuccinimide (NBS, 748 mg, 4.20 mmol) were added. Reaction completion was confirmed by TLC. The mixture was concentrated under reduced pressure and purified by column chromatography on silica gel (EtOAc/CyHex = 1:20, R_f = 0.42). The title compound was obtained as a yellowish powder (553 mg, 88% yield) and was used immediately in subsequent transformations without further characterization.

6,7-bis(bromomethyl)-2-phenylquinoxaline

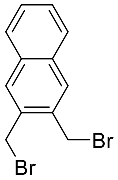

2,3-dimethylnaphthalene (313 mg, 2.00 mmol) was dissolved in carbon tetrachloride (5 mL). To the solution, 2,2′-azobisisobutyronitrile (AIBN, 32.8 mg, 0.20 mmol) and *N*-bromosuccinimide (NBS, 748 mg, 4.20 mmol) were added. Reaction completion was confirmed by TLC. The mixture was concentrated under reduced pressure and purified by column chromatography on silica gel (EtOAc/CyHex = 1:20, R_f = 0.45). The title compound was obtained as a yellowish powder (574 mg, 91% yield) and was used immediately in subsequent transformations without further characterization.

6,7-bis(bromomethyl)-2,3-diphenylquinoxaline

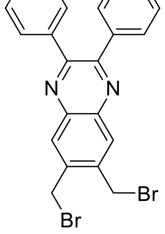

6,7-dimethyl-2,3-diphenylquinoxaline (1.00 g, 3.22 mmol) was prepared according to reported procedure[3] and was dissolved in carbon tetrachloride (5 mL). To the solution, 2,2′-azobisisobutyronitrile (AIBN, 52.5 mg, 0.32 mmol) and *N*-bromosuccinimide (NBS, 1.20 g, 6.77 mmol) were added. Reaction completion was confirmed by TLC. The mixture was concentrated

under reduced pressure and purified by column chromatography on silica gel (EtOAc/CyHex = 1:20, R_f = 0.34). The title compound was obtained as a yellowish powder (1.01 g, 67% yield) and was used immediately in subsequent transformations without further characterization.

Preparation for precursor of bis-phosphonium salt 4i, 4j, 4l, and 4m

The precursors for phosphonium salts **4i**, **4j**, **4l** and **4m** were synthesized via an alternative route resembling the Hell-Volhard-Zelinsky bromination. A solution of the corresponding alcohol (1.00 equiv.) in dichloromethane was prepared, and phosphorus tribromide (PBr_3, 1.40 equiv.) was added dropwise at 0 °C. The reaction mixture was then allowed to warm to room temperature and stirred for an additional 12 hours. Upon completion, as confirmed by TLC analysis, an aqueous workup was performed. The mixture was extracted with dichloromethane (3× 30 mL), and the combined organic layers were concentrated under reduced pressure. The crude product was purified by flash column chromatography (EtOAc/CyHex) to afford the corresponding brominated intermediate, which was immediately subjected to the subsequent transformation due to its instability. Full characterization was carried out on the corresponding phosphonium salts **4**.

1,2-bis(bromomethyl)-4,5-dimethoxybenzene

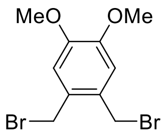

1,2-bis(bromomethyl)-4,5-dimethoxybenzene was synthesized following a previously reported protocol.[4] A solution of the 1,2-dimethoxybenzene (1.30 mL, 1.00 equiv., 10.0 mmol) in glacial acetic acid (10 mL) was prepared. To this solution, paraformaldehyde (601 mg, 2.00 equiv., 20.0 mmol). Subsequently, hydrogen bromide solution (33% in glacial acetic acid, 0.45 mL) was added at 0 °C, and the reaction was allowed to warm to room temperature and stirred for an additional 24 hour. Upon completion of the reaction, as confirmed by TLC analysis, the mixture was diluted with distilled water to precipitate the desired product. The title compound was obtained as a white solid (2.46 g, 76% yield) and immediately subjected to the subsequent transformation. Full characterization was carried out on the corresponding phosphonium salts **4d**.

1-(bromo(3-methoxyphenyl)methyl)-2-(bromomethyl)benzene

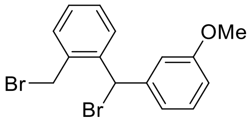

(2-(hydroxymethyl)phenyl)(3-methoxyphenyl)methanol (1.59 g, 6.51 mmol) was prepared according to reported procedure[5] and dissolved in dichloromethane (20 mL). To the solution, phosphorus tribromide (PBr_3, 857 µL, 9.11 mmol) was added dropwise at 0 °C. Reaction completion was confirmed by TLC. The mixture was concentrated under reduced pressure and purified by column chromatography on silica gel (EtOAc/CyHex = 1:20, R_f = 0.43). The title compound was obtained as a slightly yellowish oil (513 mg, 21% yield) and was used immediately in subsequent transformations without further characterization.

1-(bromo(4-(trifluoromethyl)phenyl)methyl)-2-(bromomethyl)benzene

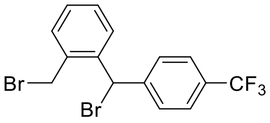

(2-(hydroxymethyl)phenyl)(4-(trifluoromethyl)phenyl)methanol (987 mg, 3.50 mmol) was prepared according to reported procedure[5] and dissolved in dichloromethane (20 mL). To the solution, phosphorus tribromide (PBr_3, 461 µL, 4.90 mmol) was added dropwise at 0 °C. Reaction completion was confirmed by TLC. The mixture was concentrated under reduced pressure and purified by column chromatography on silica gel (EtOAc/CyHex = 1:20, R_f = 0.45). The title compound was obtained as a slightly yellowish oil (678 mg, 47% yield) and was used immediately in subsequent transformations without further characterization.

1,2-bis(2-(bromomethyl)phenoxy)ethane

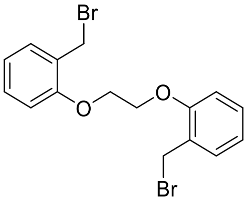

2,2'-(ethylenedioxy)bis(benzyl alcohol) (455 mg, 1.66 mmol) was dissolved in dichloromethane (5 mL). To the solution, phosphorus tribromide (PBr_3, 218 µL, 2.32 mmol) was added dropwise at 0 °C. Reaction completion was confirmed by TLC. The mixture was concentrated under reduced pressure and purified by column chromatography on silica gel (EtOAc/CyHex = 1:10, R_f = 0.45). The title compound was obtained as a white solid (452 mg, 68% yield) and was used immediately in subsequent transformations without further characterization.

1-bromo-2-(3-(2-(bromomethyl)phenoxy)propoxy)benzene

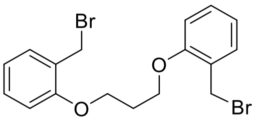

((propane-1,3-diylbis(oxy))bis(2,1-phenylene))dimethanol (711 mg, 2.47 mmol) was dissolved in dichloromethane (10 mL). To the solution, phosphorus tribromide (PBr_3, 325 µL, 2.46 mmol) was added dropwise at 0 °C. Reaction completion was confirmed by TLC. The mixture was concentrated under reduced pressure and purified by column chromatography on silica gel (EtOAc/CyHex = 1:10, R_f = 0.43). The title compound was obtained as a white solid (884 mg, 86% yield) and was used immediately in subsequent transformations without further characterization

General procedure for triphenylphosphonium salt 1 and 6 preparation (GP2)

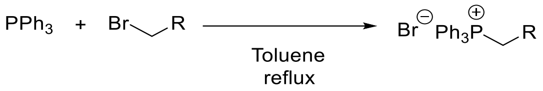

To a dried Schlenk-flask equipped with a magnetic stirring bar, a solution of the corresponding alkyl bromide (1.00 equiv.) in anhydrous toluene (5 mL) was prepared under an argon counterflow. Triphenylphosphine (1.00 equiv.) was then added, and the mixture was refluxed under an inert atmosphere for 12 hours. After cooling to room temperature, diethyl ether (20 mL) was added to the reaction mixture. The resulting suspension was filtered to collect the crude phosphonium salt as a precipitate. The product was recrystallized from a dichloromethane/ethyl acetate mixture (1:4) and washed with diethyl ether (2 × 10 mL) to afford the desired triphenylphosphonium salt.

triphenyl(pyridin-2-ylmethyl)phosphonium bromide (1a)

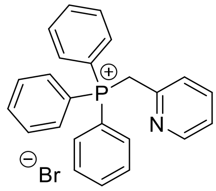

Synthesized according to the **GP2** employing 2-(bromomethyl)pyridine (344 mg, 2.00 mmol) and triphenylphosphine (525 mg, 2.00 mmol). The title compound **1a** was obtained in 86% yield (747 mg, 1.72 mmol) as a white powder.

m.p. = 252 – 253 °C.

^{1}H NMR (501 MHz, dimethyl sulfoxide-d_6, 298 K): δ [ppm] = 8.36 – 8.31 (m, 1H, Ar-*H*), 7.87 – 7.76 (m, 9H, Ar-*H*), 7.74 – 7.68 (m, 6H+1H, Ar-*H*), 7.33 (d, *J* = 7.8 Hz, 1H, Ar-*H*), 7.28 – 7.21 (m, 1H, Ar-*H*), 5.48 (d, *J* = 15.6 Hz, 2H, CH_2).

^{13}C{^{1}H} NMR (126 MHz, dimethyl sulfoxide-d_6, 298 K): δ [ppm] = 150.4 (d, *J* = 8.5 Hz, *C*-Ar), 149.1 (d, *J* = 1.8 Hz, *C*H-Ar), 137.4 (*C*H-Ar), 134.6 (d, *J* = 3.0 Hz, *C*H-Ar), 133.9 (d, *J* = 10.1 Hz, *C*H-Ar), 129.9 (d, *J* = 12.7 Hz, *C*H-Ar), 125.6 (d, *J* = 8.2 Hz, *C*H-Ar), 122.9 (*C*H-Ar), 119.2 (d, *J* = 87.2 Hz, *C*-Ar), 30.9 (d, *J* = 51.8 Hz, *C*H_2).

^{31}P NMR (202 MHz, dimethyl sulfoxide-d_6, 298 K): δ [ppm] = 23.8.

IR (ATR) [cm^{-1}]: ṽ = 3049, 2989, 2891, 2840, 1585,1485, 1473, 1434, 1318, 1190, 1161, 1108, 994, 852, 800, 747, 731, 717, 687.

HRMS-ESI(+): calc. $C_{24}H_{21}NP^+$ [M]$^+$ 354.1406; found 354.1400.

((5-bromopyridin-2-yl)methyl)triphenylphosphonium bromide (1b)

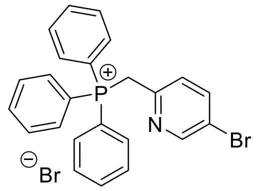

Synthesized according to the **GP2** employing 5-bromo-2-(bromomethyl)pyridine (364 mg, 1.45 mmol) and triphenylphosphine (380 mg, 1.45 mmol). The title compound **1b** was obtained in 72% yield (534 mg, 1.04 mmol) as a yellow powder.

m.p. = 207 – 208 °C.

^{1}H NMR (501 MHz, dimethyl sulfoxide-d_6, 298 K) δ [ppm] = 8.49 (d, J = 2.5 Hz, 1H, Ar-H), 8.00 (ddd, J = 8.4, 2.5, 1.0 Hz, 1H, Ar-H), 7.88 – 7.83 (m, 3H, Ar-H), 7.83 – 7.77 (m, 6H, Ar-H), 7.76 – 7.69 (m, 6H, Ar-H), 7.31 (d, J = 8.4 Hz, 1H, Ar-H), 5.49 (d, J = 15.7 Hz, 2H, CH_2).

^{13}C{^{1}H} NMR (126 MHz, dimethyl sulfoxide-d_6, 298 K): δ [ppm] = 149.9 (d, J = 1.9 Hz, CH-Ar), 149.4 (d, J = 8.6 Hz, C-Ar), 140.1 (CH-Ar), 134.8 (d, J = 3.0 Hz, CH-Ar), 133.9 (d, J = 10.4 Hz, CH-Ar), 130.0 (d, J = 12.8 Hz, CH-Ar), 127.4 (d, J = 7.9 Hz, CH-Ar), 119.6 (d, J = 2.8 Hz, C-Ar), 118.9 (d, J = 87.1 Hz, C-Ar), 30.3 (d, J = 51.8 Hz, CH_2).

^{31}P NMR (202 MHz, dimethyl sulfoxide-d_6, 298 K): δ [ppm] = 23.5.

IR (ATR) [cm^{-1}]: ṽ = 3002, 2845, 2763, 1575, 1438, 1372, 1105, 1004, 869, 685.

HRMS-ESI(+): calc. $C_{24}H_{20}BrNP^+$ $[M]^+$ 432.0511; found 432.0501.

((3-bromopyridin-2-yl)methyl)triphenylphosphonium bromide (1c)

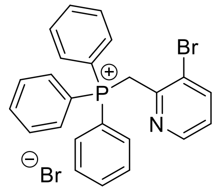

Synthesized according to the **GP2** employing 3-bromo-2-(bromomethyl)pyridine (502 mg, 2.00 mmol) and triphenylphosphine (525 mg, 2.00 mmol). The title compound **1c** was obtained in 73% yield (749 mg, 1.46 mmol) as a white powder.

m.p. = 233 – 234 °C.

^{1}H NMR (501 MHz, dimethyl sulfoxide-d_6, 298 K): δ [ppm] = 8.20 (dd, *J* = 4.8, 1.5 Hz, 1H, Ar-*H*), 8.13 (dt, *J* = 8.1, 1.5 Hz, 1H, Ar-*H*), 7.90-7.78 (m, 9H, Ar-*H*), 7.78-7.64 (m, 6H, Ar-*H*), 7.24 (dd, *J* = 8.1, 4.8 Hz, 1H, Ar-*H*), 5.64 (d, *J* = 14.2 Hz, 2H, CH_2).

^{13}C{^{1}H} NMR (126 MHz, dimethyl sulfoxide-d_6, 298 K): δ [ppm] = 149.5 (d, *J* = 6.2 Hz, *C*-Ar), 147.0 (*C*H-Ar), 141.2 (*C*H-Ar), 134.4 (d, *J* = 3.0 Hz, *C*H-Ar), 133.8 (d, *J* = 10.1 Hz, *C*H-Ar), 129.8 (d, *J* = 12.8 Hz, *C*H-Ar), 124.9 (*C*H-Ar), 121.6 (d, *J* = 10.1 Hz, *C*-Ar), 119.7 (d, *J* = 88.8 Hz, *C*-Ar), 31.6 (d, *J* = 58.7 Hz, *C*H_2).

^{31}P NMR (202 MHz, dimethyl sulfoxide-d_6, 298 K): δ [ppm] = 23.4.

IR (ATR) [cm^{-1}]: ṽ = 3048, 2799, 2725, 1560, 1481, 1425, 1170, 1106, 1011, 828, 743, 720, 681, 604, 505.

HRMS-ESI(+): calc. $C_{24}H_{20}BrNP^+$ $[M]^+$ 432.0511; found 432.0512.

((4-methoxypyridin-2-yl)methyl)triphenylphosphonium bromide (1d)

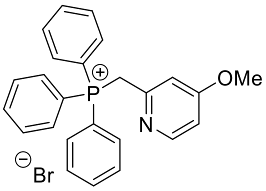

Synthesized according to the **GP2** employing 2-(bromomethyl)-4-methoxypyridine (143 mg, 710 µmol) and triphenylphosphine (186 mg, 710 µmol). The title compound **1d** was obtained in 43% yield (141 mg, 304 µmol) as a slightly yellow powder.

m.p. = 252 – 253 °C.

^{1}H NMR (600 MHz, chloroform-*d*, 300 K): δ [ppm] = 7.99 (d, *J* = 5.8 Hz, 1H, Ar-*H*), 7.84 (ddd, *J* = 12.9, 8.4, 1.3 Hz, 6H, Ar-*H*), 7.77 (dd, *J* = 2.5, 1.3 Hz, 1H, Ar-*H*), 7.74 – 7.69 (m, 3H, Ar-*H*), 7.63 – 7.58 (m, 6H, Ar-*H*), 6.59 (ddd, *J* = 5.8, 2.5, 1.5 Hz, 1H, Ar-*H*), 5.62 (d, *J* = 14.4 Hz, 2H, CH_2), 3.77 (s, 3H, CH_3).

^{13}C{^{1}H} NMR (151 MHz, chloroform-*d*, 300 K): δ [ppm] = 166.5 (*C*-Ar), 151.8 (d, *J* = 8.2 Hz, *C*-Ar), 149.4 (*C*H-Ar), 134.7 (d, *J* = 3.2 Hz, *C*H-Ar), 134.5 (d, *J* = 10.0 Hz, *C*H-Ar), 130.0 (d, *J* = 12.7 Hz, *C*H-Ar), 119.2 (d, *J* = 87.5 Hz, *C*-Ar), 112.2 (d, *J* = 7.8 Hz, *C*H-Ar), 111.2 (d, *J* = 2.1 Hz, *C*H-Ar), 56.2 (*C*H_3), 32.9 (d, *J* = 51.5 Hz, *C*H_2).

^{31}P NMR (243 MHz, chloroform-*d*, 300 K): δ [ppm] = 24.0.

IR (ATR) [cm^{-1}]: ṽ = 3853, 3735, 3649, 3355, 3009, 2922, 2178, 1648, 1593, 1567, 1518, 1488, 1437, 1310, 1234, 1110, 1027, 995, 924, 839, 793, 720, 688, 639.

HRMS-ESI(+): calc. $C_{25}H_{23}NOP^+$ $[M]^+$ 384.1512; found 384.1524.

((6-methylpyridin-2-yl)methyl)triphenylphosphonium bromide (1e)

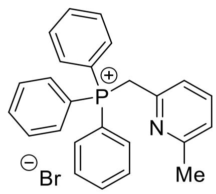

Synthesized according to the **GP2** employing 2-(bromomethyl)-6-methylpyridine (372 mg, 2.00 mmol) and triphenylphosphine (525 mg, 2.00 mmol). The title compound **1e** was obtained in 82% yield (735 mg, 1.64 mmol) as a slightly yellow powder.

m.p. = 225 – 226 °C.

^{1}H NMR (501 MHz, dimethyl sulfoxide-d_6, 298 K): δ [ppm] = 7.87 – 7.82 (m, 3H, Ar-*H*), 7.82 – 7.76 (m, 6H, Ar-*H*), 7.75 – 7.68 (m, 6H, Ar-*H*), 7.61 (t, *J* = 7.7 Hz, 1H, Ar-*H*), 7.16 (d, *J* = 7.7 Hz, 1H, Ar-*H*), 7.09 (d, *J* = 7.7 Hz, 1H, Ar-*H*), 5.41 (d, *J* = 15.4 Hz, 2H, CH_2), 2.16 (s, 3H, CH_3).

^{13}C{^{1}H} NMR (126 MHz, dimethyl sulfoxide-d_6, 298 K): δ [ppm] = 157.4 (*C*-Ar), 149.4 (d, *J* = 8.4 Hz, *C*-Ar), 137.7 (*C*H-Ar), 134.6 (d, *J* = 3.0 Hz, *C*H-Ar), 133.9 (d, *J* = 10.3 Hz, *C*H-Ar), 129.8 (d, *J* = 12.6 Hz, *C*H-Ar), 122.3 (d, *J* = 8.4 Hz, *C*H-Ar), 122.1 (*C*H-Ar), 119.4 (d, *J* = 87.2 Hz, *C*-Ar), 31.0 (d, *J* = 52.2 Hz, CH_2), 23.2 (CH_3).

^{31}P NMR (202 MHz, dimethyl sulfoxide-d_6, 298 K): δ [ppm] = 23.9.

IR (ATR) [cm^{-1}]: ṽ = 3051, 2986, 2835, 2795, 2738, 1633, 1588, 1573, 1484, 1455, 1434, 1374, 1161, 1108, 994, 856, 811, 771, 707, 686, 609.

HRMS-ESI(+): calc. $C_{25}H_{23}NP^+$ $[M]^+$ 368.1563; found 368.1553.

triphenyl(quinoline-2-ylmethyl)phosphonium bromide (1f)

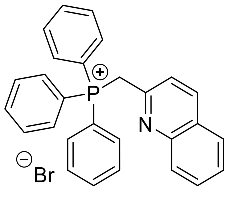

Synthesized according to the **GP2** employing quinoline-2-ylmethylbromide (444 mg, 2.00 mmol) and triphenylphosphine (525 mg, 2.00 mmol). The title compound **1f** was obtained in 70% yield (681 mg, 1.41 mmol) as a white powder.

^{1}H NMR (501 MHz, dimethyl sulfoxide-d_6, 298 K): δ [ppm] = 8.34 (d, *J* = 8.5 Hz, 1H, Ar-*H*), 7.96 – 7.91 (m, 1H, Ar-*H*), 7.91 – 7.85 (m, 6H, Ar-*H*), 7.85 – 7.80 (m, 3H, Ar-*H*), 7.74 – 7.66 (m, 6H+1H, Ar-*H*), 7.58 (t, *J* = 7.6 Hz, 2H, Ar-*H*), 7.51 (d, *J* = 8.5 Hz, 1H, Ar-*H*), 5.74 (d, *J* = 15.0 Hz, 2H, CH_2).

^{13}C{^{1}H} NMR (126 MHz, dimethyl sulfoxide-d_6, 298 K): δ [ppm] = 151.4 (d, *J* = 8.1 Hz, *C*-Ar), 146.3 (*C*-Ar), 137.5(*C*H-Ar), 134.6 (d, *J* = 2.9 Hz, *C*H-Ar), 133.9 (d, *J* = 10.4 Hz, *C*H-Ar), 130.3 (*C*H-Ar), 129.8 (d, *J* = 12.8 Hz, *C*H-Ar), 128.0 (*C*H-Ar), 127.6 (*C*H-Ar), 127.1 (*C*H-Ar), 126.3 (*C*-Ar), 122.6 (d, *J* = 8.3 Hz, *C*H-Ar), 119.6 (d, *J* = 87.8 Hz, *C*-Ar), 31.5 (d, *J* = 53.8 Hz, *C*H_2).

^{31}P NMR (202 MHz, dimethyl sulfoxide-d_6, 298 K): δ [ppm] = 23.5.

The analytical data are consistent with the literature data.[6]

((7-chloroquinolin-2-yl)methyl)triphenylphosphonium bromide (1g)

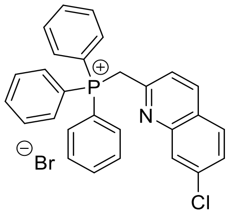

Synthesized according to the **GP2** employing 2-(bromomethyl)-7-chloroquinoline (513 mg, 2.00 mmol) and triphenylphosphine (525 mg, 2.00 mmol). The title compound **1g** was obtained in 68% yield (706 mg, 1.36 mmol) as a white powder.

m.p. = 301 – 302 °C.

^{1}H NMR (501 MHz, dimethyl sulfoxide-d_6, 298 K): δ [ppm] = 8.39 (d, *J* = 8.5 Hz, 1H, Ar-*H*), 8.00 (d, *J* = 8.5 Hz, 1H, Ar-*H*), 7.93 – 7.80 (m, 9H, Ar-*H*), 7.72 (td, *J* = 7.9, 3.6 Hz, 6H, Ar-*H*), 7.66 – 7.59 (m, 2H, Ar-*H*), 7.54 (d, *J* = 8.5 Hz, 1H, Ar-*H*), 5.75 (d, *J* = 15.2 Hz, 2H, CH_2).

^{13}C{^{1}H} NMR (126 MHz, dimethyl sulfoxide-d_6, 298 K): δ [ppm] = 153.0 (d, *J* = 8.2 Hz, *C*-Ar), 146.6 (*C*-Ar), 137.6 (*C*H-Ar), 134.8 (*C*H-Ar), 134.6 (d, *J* = 3.0 Hz, *C*H-Ar), 133.9 (d, *J* = 10.4 Hz, *C*H-Ar), 130.1 (*C*H-Ar), 129.9 (d, *J* = 12.8 Hz, *C*H-Ar), 127.7 (*C*H-Ar), 126.2 (*C*H-Ar), 125.0 (d, *J* = 7.7 Hz, *C*-Ar), 123.2 (d, *J* = 8.1 Hz, *C*-Ar), 119.4 (d, *J* = 87.7 Hz, *C*-Ar), 31.5 (d, *J* = 54.4 Hz,CH_2).

^{31}P NMR (202 MHz, dimethyl sulfoxide-d_6, 298 K): δ [ppm] = 23.5.

IR (ATR) [cm^{-1}]: ṽ = 3053, 2997, 2802, 2737, 1598, 1486, 1431, 1379, 1322, 1166, 1108, 987, 928, 853, 747, 683.

HRMS-ESI(+): calc. $C_{28}H_{22}ClNP^+$ $[M]^+$ 438.1173; found 438.1179.

(isoquinolin-3-ylmethyl)triphenylphosphonium bromide (1h)

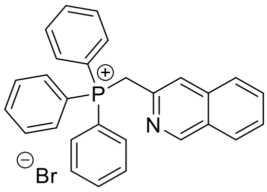

Synthesized according to the **GP2** employing 3-(bromomethyl)isoquinoline (637 mg, 2.87 mmol) and triphenylphosphine (753 mg, 2.87 mmol). The title compound **1h** was obtained in 73% yield (1.02 g, 2.11 mmol) as a slightly yellow powder.

m.p. = 174 – 175 °C.

^{1}H NMR (501 MHz, dimethyl sulfoxide-d_6, 298 K) [ppm] = 8.65 (d, J = 2.6 Hz, 1H, Ar-H), 8.50 (d, J = 2.4 Hz, 1H, Ar-H), 8.05 (d, J = 8.2 Hz, 1H, Ar-H), 8.00 (ddd, J = 8.4, 2.5, 1.0 Hz, 1H, Ar-H), 7.89 – 7.83 (m, 3H, Ar-H), 7.83 – 7.76 (m, 6H, Ar-H), 7.76 – 7.70 (m, 6H, Ar-H), 7.36 (d, J = 8.4 Hz, 1H, Ar-H), 7.28 (d, J = 8.4 Hz, 1H, Ar-H), 5.47 (d, J = 15.7 Hz, 2H, CH_2).

^{13}C{^{1}H} NMR (126 MHz, dimethyl sulfoxide-d_6, 298 K): δ [ppm] = 149.9 (d, J = 1.4 Hz, CH-Ar), 149.4 (d, J = 8.6 Hz, C-Ar), 140.1 (CH-Ar), 134.8 (d, J = 3.0 Hz, CH-Ar), 133.9 (d, J = 10.3 Hz, CH-Ar), 130.0 (d, J = 12.6 Hz, CH-Ar), 127.3 (d, J = 7.9 Hz, CH-Ar), 125.7 (C-Ar), 119.6 (d, J = 2.7 Hz, C-Ar), 118.9 (d, J = 87.2 Hz, C-Ar), 30.3 (d, J = 51.8 Hz, CH$_2$).

^{31}P NMR (202 MHz, dimethyl sulfoxide-d_6, 298 K): δ [ppm] = 23.5.

IR (ATR) [cm^{-1}]: ṽ = 3045, 2994, 2889, 2826, 2788, 2737, 1625, 1580, 1484, 1434, 1277, 1164, 1105, 994, 951, 871, 748, 684, 556.

HRMS-ESI(+): calc. $C_{28}H_{23}NP^+$ $[M]^+$ 404.1563; found 404.1562.

(isoquinolin-1-ylmethyl)triphenylphosphonium bromide (1i)

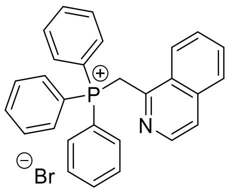

Synthesized according to the **GP2** employing 1-(bromomethyl)isoquinoline (444 mg, 2.00 mmol) and triphenylphosphine (525 mg, 2.00 mmol). The title compound **1i** was obtained in 65% yield (630 mg, 1.30 mmol) as a pink powder.

m.p. = 284 – 285 °C.

^{1}H NMR (501 MHz, dimethyl sulfoxide-d_6, 298 K): δ [ppm] = 8.41 (d, J = 8.3 Hz, 1H, Ar-H), 8.07 (d, J = 5.8 Hz, 1H, Ar-H), 8.00 (d, J = 8.3 Hz, 1H, Ar-H), 7.94 – 7.88 (m, 6H, Ar-H), 7.88 – 7.83 (m, 1H, Ar-H), 7.83 – 7.75 (m, 3H+1H, PPh_3-H+Ar-H), 7.73 (d, J = 5.8 Hz, 1H, Ar-H), 7.69 (td, J = 7.8, 3.5 Hz, 6H, Ar-H), 6.16 (d, J = 14.4 Hz, 2H, CH_2).

^{13}C{^{1}H} NMR (126 MHz, dimethyl sulfoxide-d_6, 298 K): δ [ppm] = 151.2 (d, J = 7.0 Hz, C-Ar), 140.2 (CH-Ar), 135.6 (C-Ar), 134.2 (d, J = 3.1 Hz, CH-Ar), 133.7 (d, J = 10.3 Hz, CH-Ar), 131.2 (CH-Ar), 129.7 (d, J = 12.9 Hz, CH-Ar), 128.1 (CH-Ar), 127.4 (CH-Ar), 126.7 (d, J = 8.5 Hz, C-Ar), 125.6 (CH-Ar), 120.9 (CH-Ar), 120.6 (d, J = 89.4 Hz, C-Ar), 29.4 (d, J = 60.8 Hz, $C$$H_2$).

^{31}P NMR (202 MHz, dimethyl sulfoxide-d_6, 298 K): δ [ppm] = 23.5.

IR (ATR) [cm^{-1}]: ṽ = 3049, 2804, 2751, 1587, 1564, 1434, 1387, 1362, 1159, 1112, 1038, 1016, 994, 881, 850, 806, 776, 751, 713, 692, 623.

HRMS-ESI(+): calc. $C_{28}H_{23}NP^+$ $[M]^+$ 404.1563; found 404.1578.

triphenyl(quinoxalin-2-ylmethyl)phosphonium bromide (1j)

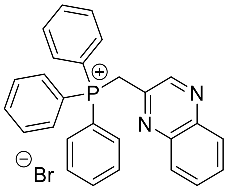

Synthesized according to the **GP2** employing 2-(bromomethyl)quinoxaline (446 mg, 2.00 mmol) and triphenylphosphine (525 mg, 2.00 mmol). The title compound **1j** was obtained in 67% yield (650 mg, 1.34 mmol) as a slightly yellow powder.

m.p. = 185 – 186 °C.

^{1}H NMR (501 MHz, dimethyl sulfoxide-d_6, 298 K): δ [ppm] = 8.87 (s, 1H, Ar-H), 8.05 (dd, J = 7.8, 1.8 Hz, 1H, Ar-H), 7.93 – 7.79 (m, 11H, Ar-H), 7.77 – 7.70 (m, 7H, Ar-H), 5.84 (d, J = 15.4 Hz, 2H, CH_2).

^{13}**C{^{1}H} NMR** (126 MHz, dimethyl sulfoxide-d_6, 298 K): δ [ppm] = 147.3 (d, *J* = 8.3 Hz, *C*-Ar), 146.5 (d, *J* = 8.5 Hz, *C*H-Ar), 140.4 (*C*-Ar), 140.2 (*C*-Ar), 134.9(*C*H-Ar), 133.9 (d, *J* = 10.5 Hz, *C*H-Ar), 131.1 (*C*H-Ar), 130.8(*C*H-Ar), 130.0 (d, *J* = 12.8 Hz, *C*H-Ar), 129.0 (*C*H-Ar), 128.1 (*C*H-Ar), 118.9 (d, *J* = 87.6 Hz, *C*-Ar), 29.2 (d, *J* = 53.2 Hz, *C*H_2).

^{31}**P NMR** (202 MHz, dimethyl sulfoxide-d_6, 298 K): δ [ppm] = 23.7.

IR (ATR) [cm^{-1}]: ṽ = 3028, 2844, 2814, 2749, 1574, 1485, 1434, 1399, 1324, 1160, 1106, 993, 893, 823, 761, 747, 686, 608.

HRMS-ESI(+): calc. $C_{27}H_{22}N_2P^+$ $[M]^+$ 405.1515; found 405.1511.

triphenyl((2-phenylpyrimidin-4-yl)methyl)phosphonium bromide (1o)

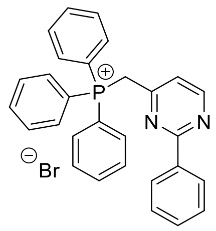

Synthesized according to the **GP2** employing 4-(bromomethyl)-2-phenylpyrimidine (135 mg, 0.54 mmol) and triphenylphosphine (142 mg, 0.54 mmol). The title compound **1o** was obtained in 70% yield (193 mg, 0.38 mmol) as a white powder.

m.p. = 247 – 248 °C.

^{1}**H NMR** (501 MHz, chloroform-*d*, 298 K): δ [ppm] = 8.64 (d, *J* = 4.9 Hz, 1H, Ar-*H*), 8.11 (d, *J* = 5.0 Hz, 1H, Ar-*H*), 7.89 (dd, *J* = 13.1, 7.6 Hz, 6H, Ar-*H*), 7.80 (d, *J* = 7.3 Hz, 2H, Ar-*H*), 7.74 (dd, *J* = 8.4, 6.4 Hz, 3H, Ar-*H*), 7.62 (td, *J* = 7.8, 3.5 Hz, 6H, Ar-*H*), 7.40 (t, *J* = 7.4 Hz, 1H, Ar-*H*), 7.29 (t, *J* = 7.7 Hz, 2H, Ar-*H*), 5.92 (d, *J* = 14.6 Hz, 2H, CH_2).

^{13}**C{^{1}H} NMR** (126 MHz, chloroform-*d*, 298 K): δ [ppm] = 163.7 (*C*-Ar), 159.4 (d, *J* = 7.9 Hz, *C*-Ar), 158.5 (*C*H-Ar), 136.7 (*C*-Ar), 135.0 (d, *J* = 3.0 Hz, *C*H-Ar), 134.3 (d, *J* = 10.4 Hz, *C*H-Ar), 131.1 (*C*H-Ar), 130.3 (d, *J* = 12.9 Hz, *C*H-Ar), 128.5 (*C*H-Ar), 128.0 (*C*H-Ar), 122.1 (d, *J* = 7.7 Hz, *C*H-Ar), 118.7 (d, *J* = 87.9 Hz, *C*-Ar), 32.5 (d, *J* = 52.7 Hz, *C*H_2).

^{31}**P NMR** (203 MHz, chloroform-*d*, 298 K): δ [ppm] = 23.7.

IR (ATR) [cm^{-1}]: ṽ =2834, 2769, 1726, 1585, 1554, 1485, 1458, 1430, 1384, 1241, 1147, 1106, 1042, 995, 899, 881, 845, 791, 750, 737, 716, 689, 652, 609, 573, 512.

HRMS-ESI(+): calc. $C_{29}H_{24}N_2P^+$ $[M]^+$ 431.1672; found 431.1671.

benzyltriphenylphosphonium bromide (7b)

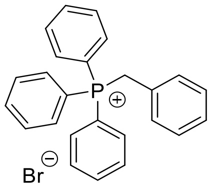

Synthesized according to the **GP2** employing benzyl bromide (342 mg, 2.00 mmol) and triphenylphosphine (525 mg, 2.00 mmol). The title compound **7b** was obtained in 96% yield (828 mg, 1.91 mmol) as a white powder.

^{1}H NMR (501 MHz, chloroform-*d*, 298 K): δ [ppm] = 7.78 – 7.68 (m, 9H), 7.67 – 7.57 (m, 6H), 7.23 - 7.18 (m, 1H, Ar-H), 7.13 – 7.06 (m, 4H, Ar-H), 5.39 (d, *J* = 14.3 Hz, 2H).

^{13}C{^{1}H} NMR (126 MHz, chloroform-*d*, 298 K): δ [ppm] = 135.1 (d, *J* = 3.1 Hz), 134.5 (d, *J* = 9.9 Hz), 131.6 (d, *J* = 5.6 Hz), 130.3 (d, *J* = 12.5 Hz), 128.9 (d, *J* = 3.4 Hz), 128.5 (d, *J* = 3.9 Hz), 127.2 (d, *J* = 8.7 Hz), 118.0 (d, *J* = 85.8 Hz), 31.0 (d, *J* = 46.8 Hz).

^{31}P NMR (203 MHz, chloroform-*d*, 298 K): δ [ppm] = 23.2.

The analytical data are consistent with the literature data.[7]

(4-(tert-butyl)benzyl)triphenylphosphonium bromide (7c)

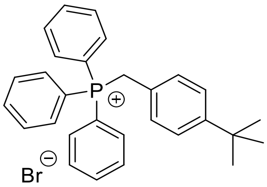

Synthesized according to the **GP2** employing 1-(bromomethyl)-4-(*tert*-butyl)benzene (227 mg, 1.00 mmol) and triphenylphosphine (262 mg, 1.00 mmol). The title compound **7c** was obtained in 95% yield (465 mg, 0.95 mmol) as a white powder.

^{1}H NMR (501 MHz, chloroform-*d*, 298 K): δ [ppm] = 7.70 (m, 3H), 7.65 – 7.58 (m, 6H), 7.58 - 7.51 (m, 6H), 7.05 (d, *J* = 7.6 Hz, 2H), 6.92 (dd, *J* = 8.4, 2.5 Hz, 2H), 5.15 (d, *J* = 13.9 Hz, 2H), 1.15 (s, 9H).

^{13}C{^{1}H} NMR (126 MHz, chloroform-*d*, 298 K): δ [ppm] = 151.6 (d, *J* = 4.2 Hz), 135.0 (d, *J* = 3.1 Hz), 134.3 (d, *J* = 9.8 Hz), 131.0 (d, *J* = 5.4 Hz), 130.1 (d, *J* = 12.5 Hz), 125.7 (d, *J* = 3.3 Hz), 123.7 (d, *J* = 8.6 Hz), 117.7 (d, *J* = 85.5 Hz), 34.5, 31.2, 30.4 (d, *J* = 47.0 Hz).

^{31}P NMR (203 MHz, chloroform-*d*, 298 K): δ [ppm] = 22.8.

The analytical data are consistent with the literature data.[8]

cinnamyltriphenylphosphonium bromide (7d)

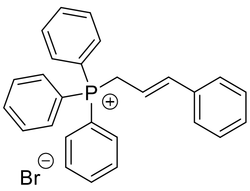

Synthesized according to the **GP2** employing (*E*)-(3-bromoprop-1-en-1-yl)benzene (197 mg, 1.00 mmol) and triphenylphosphine (262 mg, 1.00 mmol). The title compound **7d** was obtained in 84% yield (386 mg, 0.84 mmol) as a white powder.

^{1}H NMR (501 MHz, chloroform-*d*, 298 K): δ [ppm] = 7.91 – 7.84 (m, 6H), 7.77 (td, *J* = 7.5, 1.8 Hz, 3H), 7.67 (td, *J* = 7.8, 3.4 Hz, 6H), 7.29 – 7.16 (m, 5H), 6.79 (dd, *J* = 15.8, 5.7 Hz, 1H), 6.03 – 5.92 (m, 1H), 5.01 (dd, *J* = 15.4, 7.5 Hz, 2H).

^{13}C{^{1}H} NMR (126 MHz, chloroform-*d*, 298 K): δ [ppm] = 140.4 (d, *J* = 13.7 Hz), 135.9 (d, *J* = 3.9 Hz), 135.1 (d, *J* = 3.1 Hz), 134.2 (d, *J* = 9.9 Hz), 130.5 (d, *J* = 12.6 Hz), 128.7, 128.5, 126.7 (d, *J* = 2.3 Hz), 118.2 (d, *J* = 85.5 Hz), 113.8 (d, *J* = 10.9 Hz), 28.4 (d, *J* = 49.0 Hz).

^{31}P NMR (203 MHz, chloroform-*d*, 298 K): δ [ppm] = 21.6.

The analytical data are consistent with the literature data.[9]

General procedure for (bis(methylene))bis(phosphonium) bromide 4 (GP3)

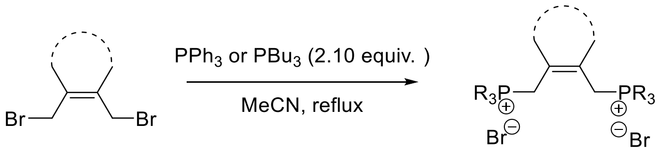

To a dried Schlenk-flask equipped with a magnetic stirring bar, a solution of alkyl bromide (1.00 equiv.) was prepared in anhydrous acetonitrile (5 mL). To this solution, triphenylphosphine or tributylphosphine (2.10 equiv.) was under an argon counterflow. The solution was refluxed under inert atmosphere for another 12 hours under an inert atmosphere. After completion, the volatiles were removed under reduced pressure. The product was precipitated from a dichloromethane/ethyl acetate (1:4) mixture and washed with diethyl ether (2 × 10 mL). If necessary, further purification was achieved by flash column chromatography ($MeOH/CH_2Cl_2$ = 1:20) to afford the desired phosphonium salt **4**.

(pyrazine-2,3-diylbis(methylene))bis(triphenylphosphonium) bromide (4a)

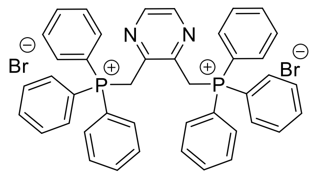

Synthesized according to the **GP3** employing 2,3-bis(bromomethyl)pyrazine (697 mg, 1.75 mmol, 1.00 equiv.) and triphenylphosphine (960 mg, 3.68 mmol, 2.10 equiv.). The title compound **4a** was obtained in 88% yield (1.22 g, 1.55 mmol) as a white powder.

m.p. >380 °C.

^{1}H NMR (501 MHz, chloroform-*d*, 298 K): δ [ppm] = 8.13 – 8.04 (m, 12H, Ar-*H*), 7.84 (s, 2H, Ar-*H*), 7.64 (m, 6H, Ar-*H*), 7.58 (m, 12H, Ar-*H*), 7.02 (d, *J* = 13.9 Hz, 4H, CH_2).

^{13}C{^{1}H} NMR (126 MHz, chloroform-*d*, 298 K): δ [ppm] = 149.5 (dd, *J* = 10.0, 7.2 Hz, *C*-Ar), 140.4 (*C*H-Ar), 134.6 (d, *J* = 10.5 Hz, *C*H-Ar), 134.0 (d, *J* = 3.1 Hz, *C*H-Ar), 129.9 (d, *J* = 13.2 Hz, *C*H-Ar), 120.0 (d, *J* = 89.4 Hz, *C*-Ar), 31.9 (d, *J* = 58.7 Hz, *C*H_2).

^{31}P NMR (203 MHz, chloroform-*d*, 298 K): δ [ppm] = 23.7.

IR (ATR) [cm^{-1}]: ṽ = 2795, 1586, 1484, 1434, 1408, 1186, 1159, 1096, 995, 868, 819, 796, 748, 712, 688, 629, 613, 515, 499.

HRMS-ESI(+): calc. $C_{29}H_{24}N_2P_2^{2+}$ $[M]^{2+}$ 315.1172; found 315.1169.

(1,2-phenylenebis(methylene))bis(triphenylphosphonium) bromide (4b)

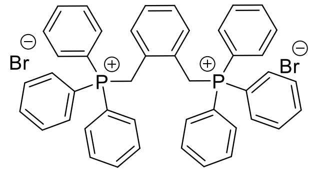

Synthesized according to the **GP3** employing 1,2-bis(bromomethyl)benzene (264 mg, 1.00 mmol, 1.00 equiv.) and triphenylphosphine (551 mg, 2.10 mmol, 2.10 equiv.). The title compound **4b** was obtained in 83% yield (658 mg, 834 µmol) as a white powder.

^{1}H NMR (501 MHz, chloroform-*d*, 298 K): δ [ppm] = 7.88 – 7.82 (m, 12H), 7.80 – 7.74 (m, 6H), 7.72 – 7.64 (m, 12H), 7.06 – 6.99 (m, 2H), 6.95 – 6.88 (m, 2H), 5.40 (d, *J* = 14.9 Hz, 4H).

^{13}C{^{1}H} NMR (126 MHz, chloroform-*d*, 298 K): δ [ppm] = 135.2, 134.5 (d, *J* = 10.5 Hz), 132.2 (d, *J* = 5.6 Hz), 130.6 (d, *J* = 13.1 Hz), 129.8 – 129.5 (m), 128.4, 118.1 (d, *J* = 86.0 Hz), 28.6 (d, *J* = 50.2 Hz).

^{31}P NMR (203 MHz, chloroform-*d*, 298 K): δ [ppm] = 22.1.

The analytical data are consistent with the literature data.[10]

((4-bromo-1,2-phenylene)bis(methylene))bis(triphenylphosphonium) bromide (4c)

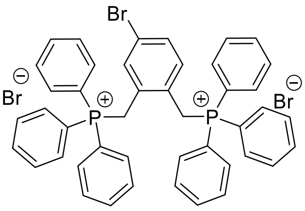

Synthesized according to the **GP3** employing 4-bromo-1,2-bis(bromomethyl)benzene (343 mg, 1.00 mmol, 1.00 equiv.) and triphenylphosphine (551 mg, 2.10 mmol, 2.10 equiv.). The title compound **4c** was obtained in 87% yield (754 mg, 869 µmol) as a white powder.

m.p. = 308 – 309 °C.

^{1}H NMR (501 MHz, chloroform-*d*, 298 K): δ [ppm] = 7.96 – 7.86 (m, 12H, PPh_3-*H*), 7.81 – 7.71 (m, 6H, PPh_3-*H*), 7.71 – 7.62 (m, 12H, PPh_3-*H*), 7.02 (dt, *J* = 8.4, 1.5 Hz, 1H, Ar-*H*), 6.95 (dt, *J* = 8.5, 1.6 Hz, 2H, Ar-*H*), 5.68 (d, *J* = 15.1 Hz, 2H, CH_2), 5.63 (d, *J* = 15.1 Hz, 2H, CH_2).

^{13}C{^{1}H} NMR (126 MHz, chloroform-*d*, 298 K): δ [ppm] = 135.2 (d, *J* = 2.5 Hz, *C*H-Ar), 135.1 (d, *J* = 2.9 Hz, *C*H-Ar), 134.5 (d, *J* = 10.9 Hz, *C*H-Ar), 134.5 (d, *J* = 10.6 Hz, *C*H-Ar), 134.4 (d, *J* = 8.6 Hz, *C*H-Ar), 133.0 (d, *J* = 6.8 Hz, *C*-Ar), 132.4 (d, *J* = 2.8 Hz, *C*-Ar), 131.1 (*C*H-Ar), 130.6 (d, *J* = 13.1 Hz, *C*H-Ar), 130.5 (d, *J* = 13.1 Hz, *C*H-Ar), 129.3 (dd, *J* = 9.9, 7.0 Hz, *C*-Ar), 121.8 (*C*H-Ar), 118.0 (d, *J* = 86.3 Hz, *C*-Ar), 117.9 (d, *J* = 86.2 Hz, *C*-Ar), 28.9 (d, *J* = 50.5 Hz, *C*H_2), 28.1 (d, *J* = 51.1 Hz, *C*H_2).

^{31}P NMR (203 MHz, chloroform-*d*, 298 K): δ [ppm] = 22.1.

IR (ATR) [cm^{-1}]: ṽ =3506, 3056, 2813, 1588, 1483, 1437, 1402, 1322, 1191, 1109, 996, 868, 830, 795, 742, 716, 688, 642, 616.

HRMS-ESI(+): calc. $C_{44}H_{37}BrP_2^{2+}$ 353.0772 $[M]^{2+}$; found 353.0762.

((4,5-dimethoxy-1,2-phenylene)bis(methylene))bis(triphenylphosphonium) bromide (4d)

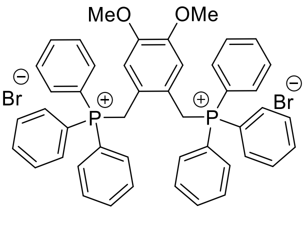

Synthesized according to the **GP3** employing 1,2-bis(bromomethyl)-4,5-dimethoxybenzene (648 mg, 2.00 mmol, 1.00 equiv.) and triphenylphosphine (1.10 g, 4.20 mmol, 2.10 equiv.). The title compound **4d** was obtained in 73% yield (1.24 g, 1.46 mmol) as a white powder.

m.p. = 244 – 245 °C.

^{1}H NMR (501 MHz, chloroform-*d*, 298 K): δ [ppm] = 7.84 – 7.79 (m, 6H, Ar-*H*), 7.74 – 7.62 (m, 24H, Ar-*H*), 6.89 (d, *J* = 1.8 Hz, 2H, Ar-*H*), 4.47 (d, *J* = 13.5 Hz, 4H, CH_2), 3.47 (s, 6H, CH_3).

^{13}C{^{1}H} NMR (126 MHz, chloroform-*d*, 298 K): δ [ppm] = 148.9 (*C*-Ar), 135.5 (*C*H-Ar), 134.7 – 134.1 (m, *C*H-Ar), 130.9 – 130.3 (m, *C*H-Ar), 119.5 (*C*-Ar), 117.5 (d, *J* = 85.7 Hz, *C*-Ar), 116.2 (*C*H-Ar), 56.3(*C*H_3), 28.0 (d, *J* = 48.2 Hz, *C*H_2).

^{31}P NMR (203 MHz, chloroform-*d*, 298 K): δ [ppm] = 20.6.

IR (ATR) [cm^{-1}]: ṽ = 3056, 3005, 2846, 2790, 1594, 1523, 1437, 1350, 1276, 1151, 1106, 991, 869, 746, 686, 545, 498.

HRMS-ESI(+): calc. $C_{46}H_{42}O_2P_2{}^{2+}$ $[M]^{2+}$; 344.1325 found 344.1326.

((3-(methoxycarbonyl)-1,2-phenylene)bis(methylene))bis(triphenylphosphonium) bromide (4e)

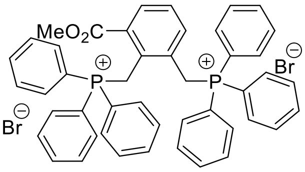

Synthesized according to the **GP3** employing methyl 2,3-bis(bromomethyl)benzoate (566 mg, 1.76 mmol, 1.00 equiv.) and triphenylphosphine (969 mg, 3.70 mmol, 2.10 equiv.). The title compound **4e** was obtained in 78% yield (1.16 g, 1.37 mmol) as a white powder.

m.p. = 218 – 222 °C.

^{1}H NMR (501 MHz, chloroform-*d*, 298 K): δ [ppm] = 7.83 – 7.72 (m, 15H, Ar-*H*), 7.69 – 7.59 (m, 11H, Ar-*H*), 7.56 – 7.50 (m, 4H+2H, Ar-*H*), 7.14 (td, *J* = 7.9, 2.5 Hz, 1H, Ar-*H*), 5.69 (d, *J* = 15.1 Hz, 2H, CH_2), 5.02 (br s, 2H, CH_2), 3.50 (s, 3H, CH_3).

^{13}C{^{1}H} NMR (126 MHz, chloroform-*d*, 298 K): δ [ppm] = 166.7 (*C*OOMe), 136.5 (m, *C*-Ar), 135.3 (d, *J* = 3.1 Hz, *C*H-Ar), 135.0 (d, *J* = 10.0 Hz, *C*H-Ar), 134.8 – 134.6 (m, *C*H-Ar), 133.4 (d, *J* = 10.7 Hz, *C*-Ar), 131.9 – 131.8 (m, *C*H-Ar), 131.7 – 131.6 (m, *C*-Ar), 131.1 (t, *J* = 6.2 Hz, *C*H-Ar), 130.9 (d, *J* = 5.2 Hz, *C*-Ar), 130.5 (d, *J* = 12.7 Hz, *C*H-Ar), 130.1 (d, *J* = 12.5 Hz, *C*H-Ar), 128.7 – 128.4 (m,

CH-Ar), 117.9 (d, J = 85.5 Hz, C-Ar), 117.1 (d, J = 86.1 Hz, C-Ar), 52.6 (CH_3), 28.3 (d, J = 48.0 Hz, CH_2), 28.1 (d, J = 50.4 Hz, CH_2).

^{31}P NMR (203 MHz, chloroform-*d*, 298 K): δ [ppm] = 23.84 (d, J = 3.8 Hz), 22.14 (d, J = 4.1 Hz).

IR (ATR) [cm^{-1}]: ṽ = 3490, 3054, 2991, 1702, 1586, 1485, 1438, 1410, 1376, 1325, 1274, 1204, 1162, 1108, 996, 898, 841, 811, 778, 754, 737, 718, 690, 616, 588, 541.

HRMS-ESI(+): calc. $C_{46}H_{40}O_2P_2^{2+}$ $[M]^{2+}$ 343.1247; found 343.1243.

(naphthalene-1,2-diylbis(methylene))bis(tributylphosphonium) bromide (4f)

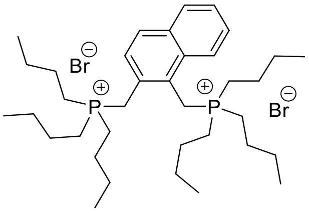

Synthesized according to the **GP3** employing 1,2-bis(bromomethyl)naphthalene (553 mg, 1.76 mmol, 1.00 equiv.) and tri-*n*-butyl phosphine (912 µL, 3.70 mmol, 2.10 equiv.). The title compound **4f** was obtained in 43% yield (544 mg, 757 µmol) as a pink powder.

m.p. = 88 – 89 °C.

^{1}H NMR (501 MHz, chloroform-*d*, 298 K): δ [ppm] = 8.12 (d, J = 8.6 Hz, 1H, Ar-*H*), 8.02 (d, J = 8.5 Hz, 1H, Ar-*H*), 7.85 (d, J = 8.3 Hz, 2H, Ar-*H*), 7.65 (t, J = 7.7 Hz, 1H, Ar-*H*), 7.52 (t, J = 7.5 Hz, 1H, Ar-*H*), 5.51 (br s, 1H, CH_2), 4.82 (br s, 2H, CH_2), 4.34 (br s, 1H, CH_2), 2.64 (br s, 3H, PBu_3-*H*), 2.61 – 2.51 (m, 6H, PBu_3-*H*), 2.22 – 2.10 (m, 3H, PBu_3-*H*), 1.40 – 0.94 (m, 24H, PBu_3-*H*), 0.82 – 0.62 (m, 18H, PBu_3-*H*).

^{13}C{^{1}H} NMR (126 MHz, chloroform-*d*, 298 K): δ [ppm] = 133.2 (t, J = 2.6 Hz, C-Ar), 132.2 (t, J = 3.4 Hz, C-Ar), 129.8(CH-Ar), 129.5 (d, J = 3.6 Hz, CH-Ar), 129.4 (CH-Ar), 128.3 (dd, J = 8.9, 6.8 Hz, C-Ar), 128.1 (CH-Ar), 127.2 (CH-Ar), 124.4 (m, C-Ar), 123.4 (CH-Ar), 26.91 (d, J = 44.4 Hz, CH_2), 23.9 (d, J = 21.9 Hz, C-*n*Bu), 23.77 (d, J = 13.3 Hz, C-*n*Bu), 23.75 (d, J = 13.3 Hz, C-*n*Bu), 23.6 (d, J = 5.1 Hz, C-*n*Bu), 22.30 (d, J = 45.3 Hz, CH_2). 19.2 (d, J = 45.3 Hz, C-*n*Bu), 19.0 (d, J = 45.3 Hz, C-*n*Bu), 13.3 (C-*n*Bu), 13.2 (C-*n*Bu).

^{31}P NMR (203 MHz, chloroform-*d*, 298 K): δ [ppm] = 36.02 (d, J = 9.0 Hz), 33.92 (d, J = 9.0 Hz).

IR (ATR) [cm^{-1}]: ṽ = 2961, 2933, 2874, 1600, 1513, 1464, 1409, 1383, 1345, 1314, 1277, 1234, 1140, 1098, 1005, 970, 949, 913, 837, 750, 725, 593, 533.

HRMS-ESI(+): calc. $C_{36}H_{64}P_2^{2+}$ $[M]^{2+}$ 279.2236; found 279.2230.

(naphthalene-2,3-diylbis(methylene))bis(triphenylphosphonium) bromide (4g)

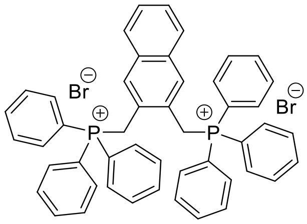

Synthesized according to the **GP3** employing 2,3-bis(bromomethyl)naphthalene (829 mg, 2.64 mmol, 1.00 equiv.) and triphenylphosphine (1.45 g, 5.54 mmol, 2.10 equiv.). The title compound **4g** was obtained in 80% yield (1.78 g, 2.12 mmol) as a white powder.

^{1}H NMR (501 MHz, acetonitrile-d_3, 298 K): δ [ppm] = 7.90 – 7.84 (m, 6H), 7.71 – 7.61 (m, 24H), 7.47 – 7.41 (m, 6H), 4.71 (d, J = 15.1 Hz, 4H).

^{13}C{^{1}H} NMR (126 MHz, acetonitrile-d_3, 298 K): δ [ppm] = 136.4 (d, J = 2.8 Hz), 135.4 (d, J = 10.2 Hz), 133.4, 133.1, 131.2 (d, J = 12.9), 128.8, 128.1, 126.1, 118.5, 28.4 (d, J = 49.6 Hz).

^{31}P NMR (203 MHz, acetonitrile-d_3, 298 K): δ [ppm] = 22.18.

The analytical data are consistent with the literature data.[11]

((2,3-diphenylquinoxaline-6,7-diyl)bis(methylene))bis(tributylphosphonium) bromide (4h)

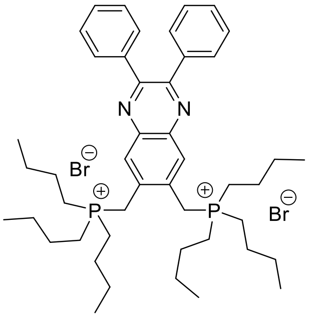

Synthesized according to the **GP3** employing 6,7-bis(bromomethyl)-2,3-diphenylquinoxaline (1.01 g, 2.16 mmol, 1.00 equiv.) and tri-n-butylphosphine (1.12 mL, 4.53 mmol, 2.10 equiv.). The title compound **4h** was obtained in 83% yield (1.56 g, 1.79 mmol) as a white powder.

m.p. = 288–291 °C.

^{1}H NMR (501 MHz, chloroform-d, 298 K): δ [ppm] = 8.29 (d, J = 2.9 Hz, 2H, Ar-H), 7.55 – 7.50 (m, 4H, Ar-H), 7.40 (t, J = 7.3 Hz, 2H, Ar-H), 7.35 (t, J = 7.4 Hz, 4H, Ar-H), 5.09 (d, J = 16.5 Hz, 4H, CH_2), 2.76 – 2.52 (m, 12H, PBu$_3$-H), 1.64 – 1.44 (m, 24H, PBu$_3$-H), 0.96 – 0.90 (m, 18H, PBu$_3$-H).

^{13}C{^{1}H, ^{31}P} NMR (126 MHz, chloroform-*d*, 298 K): δ [ppm] = 155.4 (*C*-Ar), 140.2 (*C*-Ar), 138.4 (*C*-Ar), 132.7 (*C*H-Ar), 131.4 (*C*-Ar), 130.0 (*C*H-Ar), 129.6 (*C*H-Ar), 128.5 (*C*H-Ar), 26.5(CH_2), 24.2 (*C*-*n*Bu), 19.3 (*C*-*n*Bu), 13.6 (*C*-*n*Bu).

^{31}P NMR (203 MHz, chloroform-*d*, 298 K): δ [ppm] = 34.4.

IR (ATR) [cm^{-1}]: ṽ = 2962, 2935, 2875, 1483, 1465, 1395, 1345, 1227, 1091, 1062, 1026, 978, 918, 815, 767, 731, 699, 598, 565, 540, 488, 422

HRMS-ESI(+): calc. $C_{46}H_{70}N_2P^{2+}$ $[M]^{2+}$ 356.2502; found 356.2496.

tributyl(2-((tributylphosphonio)(4-(trifluoromethyl)phenyl)methyl)benzyl)phosphonium bromide (4i)

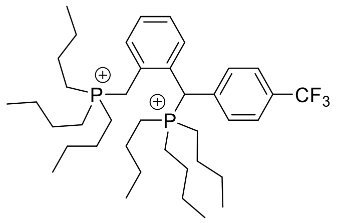

Synthesized according to the **GP3** employing 1-(bromo(4-(trifluoromethyl)phenyl)methyl)-2-(bromomethyl)benzene (678 mg, 1.66 mmol, 1.00 equiv.) and tri-*n*-butylphosphine (860 µL, 3.49 mmol, 2.10 equiv.). The title compound **4i** was purified via flash column chromatography and obtained as a white solid in 46% yield (744 mg, 91.5 µmol) .

m.p. = 121-122 °C

^{1}H NMR (501 MHz, chloroform-*d*, 298 K): δ [ppm] = 8.35 (d, *J* = 8.1 Hz, 2H, Ar-*H*), 8.12 (d, *J* = 7.9 Hz, 1H, Ar-*H*), 7.91 (d, *J* = 7.8 Hz, 1H, Ar-*H*), 7.78 (t, *J* = 7.6 Hz, 1H, Ar-*H*), 7.71 (d, *J* = 8.2 Hz, 2H, Ar-*H*), 7.52 (t, *J* = 7.6 Hz, 1H, Ar-*H*), 7.03 (s, 1H, C*H*), 5.39 (d, *J* = 15.4 Hz, 1H, C*H2*), 4.24 (d, *J* = 15.4 Hz, 1H, C*H2*), 2.75 – 2.65 (m, 3H, PBu_3-*H*), 2.47 – 2.33 (m, 6H, PBu_3-*H*), 2.12 – 2.02 (m, 3H, PBu_3-*H*), 1.45 – 1.33 (m, 6H, PBu_3-*H*), 1.31 – 1.09 (m, 12H, PBu_3-*H*), 0.86 – 0.76 (m, 18H, PBu_3-*H*).

^{13}C{^{1}H} NMR (126 MHz, chloroform-*d*, 298 K): δ [ppm] = 136.7 (d, *J* = 5.2 Hz, *C*-Ar), 134.2 (d, *J* = 4.4 Hz, *C*H-Ar), 132.4 (dd, *J* = 5.9, 2.6 Hz, *C*H-Ar), 131.9 (dd, *J* = 5.8, 3.3 Hz, *C*-Ar), 131.6 (m, *C*-Ar), 130.9 (d, *J* = 4.6 Hz, *C*H-Ar), 130.4 (*C*H-Ar), 129.7 (t, *J* = 8.1 Hz, *C*-Ar), 126.8-126.6 (m, *C*H-Ar), 123.6 (q, *J* = 272.5 Hz, CF_3), 40.7 (d, *J* = 40.9 Hz, *C*H), 25.5 (d, *J* = 45.2 Hz, *C*H2), 25.0 – 22.7 (m, *C*-*n*Bu), 19.5 (dd, *J* = 44.4, 34.6 Hz, *C*-*n*Bu), 13.4 (d, *J* = 2.7 Hz, *C*-*n*Bu).

^{31}P NMR (203 MHz, chloroform-*d*, 298 K): δ [ppm] = 38.26, 32.84 (d, *J* = 2.8 Hz).

^{19}F NMR (565 MHz, chloroform-*d*): δ [ppm] = -62.99.

IR (ATR) [cm^{-1}]: ṽ = 2963, 2936, 2875, 1619, 1465, 1419, 1326, 1232, 1165, 1120, 1069, 1018, 968, 913, 810, 726, 602, 523, 487.

HRMS-ESI(+): calc. $C_{39}H_{65}F_3P_2^{2+}$ $[M]^{2+}$; 326.2252 found 326.2254.

tributyl((3-methoxyphenyl)(2-((tributylphosphonio)methyl)phenyl)methyl)phosphonium bromide (4j)

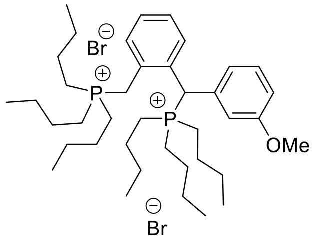

Synthesized according to the **GP3** employing 1-(bromo(3-methoxyphenyl)methyl)-2-(bromomethyl)benzene (513 mg, 1.39 mmol, 1.00 equiv.) and tri-*n*-butylphosphine (718 µL, 2.91 mmol, 2.10 equiv.). The title compound **4j** was purified via flash column chromatography and obtained as a white solid in 53% yield (571 mg, 737 µmol).

m.p. = 129 – 130 °C

^{1}H NMR (501 MHz, chloroform-*d*, 298 K): δ [ppm] = 8.01 (d, *J* = 7.9 Hz, 1H, Ar-*H*), 7.91 (d, *J* = 7.9 Hz, 1H, Ar-*H*), 7.78 (d, *J* = 2.4 Hz, 1H, Ar-*H*), 7.70 (t, *J* = 7.7 Hz, 1H, Ar-*H*), 7.54 (t, *J* = 8.0 Hz, 2H, Ar-*H*), 7.34 (t, *J* = 8.0 Hz, 1H), 6.92 (d, *J* = 8.4 Hz, 1H, Ar-*H*), 6.85 (d, *J* = 20.6 Hz, 1H, C*H*), 5.39 (t, *J* = 15.5 Hz, 1H, CH_2), 4.25 (t, *J* = 15.5 Hz, 1H, CH_2), 3.87 (s, 3H, O-CH_3), 2.66 (dtd, *J* = 16.6, 12.4, 4.6 Hz, 3H), 2.52 – 2.42 (m, 3H, PBu_3-*H*), 2.36 (dtd, *J* = 16.6, 12.4, 4.6 Hz, 3H, PBu_3-*H*), 2.15 – 2.03 (m, 3H, PBu_3-*H*), 1.45 – 1.33 (m, 7H, PBu_3-*H*), 1.33 – 1.16 (m, 17H, PBu_3-*H*), 0.89 – 0.78 (m, 18H, PBu_3-*H*).

^{13}C{^{1}H} NMR (126 MHz, chloroform-*d*, 298 K): δ [ppm] = 160.7 (m, *C*-Ar), 134.5 (m, *C*H-Ar), 133.7 (m, *C*-Ar), 132.5 (m, *C*-Ar), 131.6 (m, *C*H-Ar), 131.0 (m, *C*H-Ar), 130.2 (m, *C*H-Ar), 130.1 (m, *C*-Ar), 129.8 (m, *C*H-Ar), 121.9 (d, *J* = 4.7 Hz, *C*H-Ar), 116.0 (d, *J* = 4.8 Hz, *C*H-Ar), 115.2 (d, *J* = 3.2 Hz, *C*H-Ar), 56.2 (O*C*H_3), 40.9 (d, *J* = 41.2 Hz, *C*H), 25.5 (d, *J* = 45.1 Hz, *C*H_2), 24.49 – 23.48 (m, *C*-*n*Bu), 19.55 (dd, *J* = 44.5, 39.3 Hz, *C*-*n*Bu), 13.50 (d, *J* = 13.4 Hz, *C*-*n*Bu).

^{31}P NMR (203 MHz, chloroform-*d*, 298 K): δ [ppm] = 37.27 (d, *J* = 3.2 Hz), 32.82 (d, *J* = 3.2 Hz).

IR (ATR) [cm^{-1}]: ṽ = 2962, 2934, 2874, 1599, 1490, 1464, 1383, 1302, 1263, 1171, 1096, 1036, 999, 968, 914, 797, 705, 639, 555, 488, 449

HRMS-ESI(+): calc. $C_{39}H_{68}OP_2^{2+}$ $[M]^{2+}$ 307.2367; found 307.2364.

(*Z*)-but-2-ene-1,4-diylbis(triphenylphosphonium) chloride (4k)

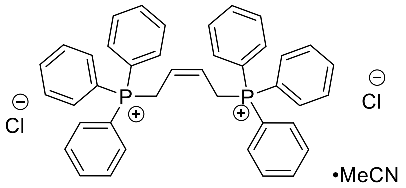

Synthesized according to the **GP3** employing (*Z*)-1,4-dichlorobutene (105 µL, 1.00 mmol, 1.00 equiv.) and triphenylphosphine (551 mg, 2.10 mmol, 2.10 equiv.). The title compound **4k** co-crystallized with MeCN was obtained as a white powder in 54% yield (374 mg, 542 µmol).

m.p. = 278 – 279 °C.

^{1}H NMR (501 MHz, chloroform-*d*, 298 K): δ [ppm] = 7.79 – 7.71 (m, 12H, Ar-*H*), 7.71 – 7.66 (m, 6H, Ar-*H*), 7.66 – 7.60 (m, 12H, Ar-*H*), 6.33 – 6.27 (m, 2H, C*H*=C*H*), 4.85 – 4.75 (m, 4H, C*H*$_2$).

^{13}C{^{1}H} NMR (126 MHz, chloroform-*d*, 298 K): δ [ppm] = 134.9 (*C*H-Ar), 134.3 – 134.0 (m, *C*H-Ar), 130.7 – 130.2 (m, *C*H-Ar), 126.7 – 126.1 (m, *C*H=*C*H), 117.7 (dd, *J* = 88.5, 2.4 Hz, *C*-Ar), 28.5 – 27.8 (m, *C*H$_2$).

^{31}P NMR (203 MHz, chloroform-*d*, 298 K): δ [ppm] = 21.0.

IR (ATR) [cm^{-1}]: ṽ = 2902, 2846, 2773, 2239, 1587, 1483, 1436, 1317, 1156, 1111, 1068, 997, 864, 746, 720, 691, 616, 544, 506.

HRMS-ESI(+): calc. $C_{40}H_{36}P_2^{2+}$ $[M]^{2+}$ 289.1141; found 289.1140.

(((ethane-1,2-diylbis(oxy))bis(2,1-phenylene))bis(methylene))bis(tributylphosphonium) bromide (4l)

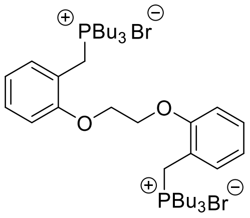

Synthesized according to the **GP3** employing 1,2-bis(2-(bromomethyl)phenoxy)ethane (452 mg, 1.13 mmol, 1.00 equiv.) and tributyl phosphine (616 µL, 2.37 mmol, 2.10 equiv.). The title compound **4l** was obtained in 64% yield (582 mg, 723 µmol) as a white powder.

m.p. = 233 – 239 °C.

^{1}H NMR (501 MHz, chloroform-*d*, 298 K): δ [ppm] = 7.38 – 7.29 (m, 4H, Ar-*H*), 7.11 (d, *J* = 8.6 Hz, 2H, Ar-*H*), 6.98 (t, *J* = 6.8 Hz, 2H, Ar-*H*), 4.63 (s, 4H, H_2C-CH_2), 4.11 (d, *J* = 15.1 Hz, 4H, CH_2), 2.38 – 2.28 (m, 12H, PBu_3-*H*), 1.44 – 1.31 (m, 24H, PBu_3-*H*), 0.87 – 0.74 (m, 18H, PBu_3-*H*).

^{13}C{^{1}H} NMR (126 MHz, chloroform-*d*, 298 K): δ [ppm] = 156.5 (d, *J* = 5.1 Hz, *C*-Ar), 131.8 (d, *J* = 4.7 Hz, *C*H-Ar), 130.4 (d, *J* = 3.6 Hz, *C*H-Ar), 122.1 (d, *J* = 3.1 Hz, *C*H-Ar), 117.7 (d, *J* = 8.8 Hz, *C*-Ar), 113.3 (d, *J* = 3.2 Hz, *C*H-Ar), 67.7 (H_2*C*-*C*H_2), 24.0 (d, *J* = 15.3 Hz, *n*-Bu), 23.7 (d, *J* = 4.8 Hz, *n*-Bu), 22.5 (d, *J* = 46.8 Hz, *C*H_2), 19.3 (d, *J* = 46.1 Hz, *n*-Bu), 13.5 (*n*-Bu).

^{31}P NMR (203 MHz, chloroform-*d*, 298 K): δ [ppm] = 32.6.

IR (ATR) [cm^{-1}]: ṽ = 2958, 2929, 2871, 1598, 1586, 1494, 1454, 1403, 1382, 1292, 1247, 1220, 1186, 1104, 1048, 1022, 969, 907, 830, 754, 724.

HRMS-ESI(+): calc. $C_{40}H_{70}O_2P_2^{2+}$ $[M]^{2+}$ 322.2420; found 322.2413.

(((propane-1,3-diylbis(oxy))bis(2,1-phenylene))bis(methylene))bis(tributylphosphonium) bromide (4m)

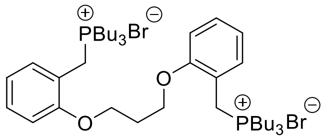

Synthesized according to the **GP3** employing 1,3-bis(2-(bromomethyl)phenoxy)propane (884 mg, 2.12 mmol, 1.00 equiv.) and tributyl phosphine (1.16 mL, 4.72 mmol, 2.10 equiv.). The title compound **4m** was obtained in 73% yield (1.26 g, 1.54 mmol) as a white powder.

m.p. = 195 – 197 °C.

^{1}H NMR (501 MHz, chloroform-*d*, 298 K): δ [ppm] = 7.39 (d, *J* = 7.4 Hz, 2H, Ar-*H*), 7.31 (td, *J* = 7.8, 3.7 Hz, 2H, Ar-*H*), 7.08 – 7.02 (m, 2H, Ar-*H*), 6.95 (dt, *J* = 9.2, 4.5 Hz, 2H, Ar-*H*), 4.36 – 4.29 (m, 4H, H_2C-CH_2-CH_2), 4.11 – 4.03 (m, 4H, CH_2), 2.46 (q, *J* = 5.6 Hz, 2H, H_2C-CH_2-CH_2), 2.42 – 2.32 (m, 12H, PBu_3-*H*), 1.47 – 1.36 (m, 24H, PBu_3-*H*), 0.92 – 0.84 (m, 18H, PBu_3-*H*).

^{13}C{^{1}H} NMR (126 MHz, chloroform-*d*, 298 K): δ [ppm] = 156.5 (*C*-Ar), 131.9 (*C*H-Ar), 130.4 (*C*H-Ar), 121.7(*C*H-Ar), 117.2 (d, *J* = 8.9 Hz, *C*-Ar), 112.8 (*C*H-Ar), 66.0 (H_2*C*-CH_2-*C*H_2), 29.0 (H_2C-*C*H_2-CH_2), 24.1 (d, *J* = 15.3 Hz, *n*-Bu), 23.8 (d, *J* = 4.8 Hz, *n*-Bu), 22.2 (d, *J* = 45.5 Hz, CH_2), 19.3 (d, *J* = 46.2 Hz, *n*-Bu), 13.6 (*n*-Bu).

^{31}P NMR (203 MHz, chloroform-*d*, 298 K): δ [ppm] = 32.5.

IR (ATR) [cm^{-1}]: ṽ = 2963, 2934, 2876, 1600, 1498, 1456, 1404, 1297, 1251, 1106, 1044, 990, 957, 907, 836, 753, 726, 486, 450.

HRMS-ESI(+): calc. $C_{41}H_{72}O_2P_2^{2+}$ $[M]^{2+}$ 329.2499; found 329.2496.

General procedure for tributyl phosphonium salt 2 and 9 (GP4)

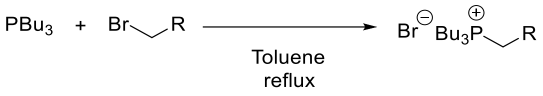

To a dried Schlenk-flask equipped with a magnetic stirring bar, a solution of alkyl bromide (1.00 equiv.) was prepared in anhydrous toluene (5 mL). To this solution, tributyl phosphine (1.10 equiv.) was added under argon counter flow. The solution was refluxed under inert atmosphere for another 12 hours. Volatiles were removed in *vacuo* at 50 °C. To the reaction residue, anhydrous diethyl ether was added. Upon sonication the phosphonium salt would precipitate out from the solution. The resulting suspension was filtered, and the desired product was obtained as a precipitate. Due to its instability in selected cases while exposure to air, the whole procedure is carried out under inert condition.

tributyl(pyridin-2-ylmethyl)phosphonium bromide (2a)

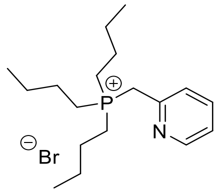

Synthesized according to the **GP4** employing 2-(bromomethyl)pyridine (1.72 g, 10.0 mmol, 1.00 equiv.) and tributyl phosphine (2.86 mL, 11.0 mmol, 1.10 equiv.). The title compound **2a** was obtained in 83% yield (3.12 g, 8.33 mmol) as a white powder.

m.p. = 254 – 256 °C

^{1}H NMR (501 MHz, acetonitrile-d_3, 298 K): δ [ppm] = 8.54 (d, J = 4.6 Hz, 1H, Ar-H), 7.80 (t, J = 7.5 Hz, 1H, Ar-H), 7.47 (d, J = 7.9 Hz, 1H, Ar-H), 7.35 (t, J = 6.3 Hz, 1H, Ar-H), 3.89 (d, J = 14.6 Hz, 2H, CH_2), 2.28 – 2.18 (m, 6H, PBu_3-H), 1.56 – 1.46 (m, 6H, PBu_3-H), 1.42 (h, J = 7.2 Hz, 6H, PBu_3-H), 0.91 (t, J = 7.3 Hz, 9H, PBu_3-H).

^{13}C{^{1}H} NMR (126 MHz, acetonitrile-d_3, 298 K): δ [ppm] = 151.8 (C-Ar), 150.6 (CH-Ar), 138.8 (CH-Ar), 125.9 (CH-Ar), 124.1 (CH-Ar), 28.7 (d, J = 48.7 Hz, CH_2), 24.4 (d, J = 15.9 Hz, n-Bu), 23.9 (d, J = 4.7 Hz, n-Bu), 19.7 (d, J = 47.7 Hz, n-Bu), 13.5 (n-Bu).

^{31}P NMR (203 MHz, acetonitrile-d_3, 298 K) δ [ppm] = 34.4

IR (ATR) [cm^{-1}]: ṽ = 3044, 2959, 2930, 2870, 1628, 1586, 1569, 1467, 1433, 1380, 1314, 1234, 1201, 1098, 1052, 996, 969, 916, 841, 795, 765, 707, 625, 596, 479, 423, 407.

HRMS-ESI(+): calc. $C_{18}H_{33}NP^+$ $[M]^+$ 294.2345; found 294.2340.

((1,5-naphthyridin-2-yl)methyl)tributylphosphonium bromide (2k)

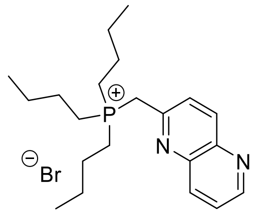

Synthesized according to the **GP4** employing 2-(bromomethyl)-1,5-naphthyridine (112 mg, 0.50 mmol, 1.00 equiv.) and tributyl phosphine (143 µL, 0.55 mmol, 1.10 equiv.). The title compound **2k** was obtained in 84% yield (179 mg, 421 µmol) as a white powder.

m.p. = 176 – 178 °C

^{1}H NMR (501 MHz, acetonitrile-d_3, 298 K): δ [ppm] = 9.04 (dd, J = 4.5, 1.6 Hz, 1H, Ar-H), 8.65 (d, J = 8.7 Hz, 1H, Ar-H), 8.55 (d, J = 8.7 Hz, 1H, Ar-H), 7.91 – 7.86 (m, 2H, Ar-H), 4.20 (d, J = 14.3 Hz, 2H, CH_2), 2.47 – 2.22 (m, 6H, PBu_3-H), 1.67 – 1.51 (m, 6H, PBu_3-H), 1.44 (h, J = 7.3 Hz, 6H, PBu_3-H), 0.91 (t, J = 7.3 Hz, 9H, PBu_3-H).

^{13}C{^{1}H} NMR (126 MHz, acetonitrile-d_3, 298 K): δ [ppm] = 154.9 (C-Ar), 151.1 (CH-Ar), 144.0 (CH-Ar), 141.8 (C-Ar), 139.9 (C-Ar), 137.9 (CH-Ar), 127.8 (d, J = 6.7 Hz, CH-Ar), 126.4 (CH-Ar), 29.1 (d, J = 49.6 Hz, CH$_2$), 24.4 (d, J = 16.2 Hz, n-Bu), 24.1 (d, J = 4.7 Hz, n-Bu), 19.9 (d, J = 47.8 Hz, n-Bu), 13.5 (n-Bu).

^{31}P NMR (203 MHz, acetonitrile-d_3, 298 K): δ [ppm] = 35.1.

IR (ATR) [cm^{-1}]: ṽ = 2959, 2931, 2871, 1590, 1494, 1462, 1384, 1340, 1313, 1234, 1100. 966, 919, 863, 827, 719, 611.

HRMS-ESI(+): calc. $C_{21}H_{34}N_2P^+$ $[M]^+$ 345.2454; found 345.2457.

tributyl(pyrimidin-2-ylmethyl)phosphonium chloride (2l)

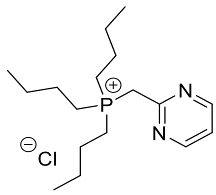

Synthesized according to the **GP4** employing 2-(chloromethyl)pyrimidine (643 mg, 5.00 mmol, 1.00 equiv.) and tributyl phosphine (1.43 mL, 5.50 mmol, 1.10 equiv.). The title compound **2l** was obtained in 73% yield (1.20 g, 3.63 mmol) as a white powder.

m.p. = 119 – 121 °C

^{1}H NMR (501 MHz, acetonitrile-d_3, 298 K): δ [ppm] = 8.75 (d, J = 5.0 Hz, 2H, Ar-H), 7.41 (td, J = 4.9, 1.3 Hz, 1H, Ar-H), 3.98 (d, J = 14.5 Hz, 2H, CH_2), 2.30 – 2.20 (m, 6H, PBu_3-H), 1.54 (tdd, J = 11.3, 8.6, 6.1 Hz, 6H, PBu_3-H), 1.43 (h, J = 7.2 Hz, 6H, PBu_3-H), 0.91 (t, J = 7.3 Hz, 9H, PBu_3-H).

^{13}C{^{1}H} NMR (126 MHz, acetonitrile-d_3, 298 K): δ [ppm] = 162.4 (d, J = 6.7 Hz, C-Ar), 159.1 (CH-Ar), 121.4 (d, J = 1.7 Hz, CH-Ar), 30.7 (d, J = 48.6 Hz, CH_2), 24.5 (d, J = 16.2 Hz, n-Bu), 23.9 (d, J = 4.7 Hz, n-Bu), 19.6 (d, J = 47.9 Hz, n-Bu), 13.5 (n-Bu).

^{31}P NMR (203 MHz, acetonitrile-d_3, 298 K): δ [ppm] = 34.6.

IR (ATR) [cm^{-1}]: ṽ = 2958, 2933, 2871, 1561, 1465, 1413, 1303, 1229, 1100, 993, 969, 917, 859, 831, 800, 722, 635, 618, 485, 464.

HRMS-ESI(+): calc. $C_{17}H_{32}N_2P^+$ $[M]^+$ 295.2298; found 295.2305.

tributyl(5,6,7,8-tetrahydroquinolin-8-yl)phosphonium bromide (2m)

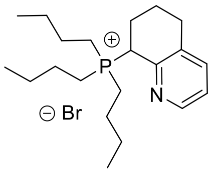

Synthesized according to the **GP4** employing 8-bromo-5,6,7,8-tetrahydroquinoline (357 mg, 1.68 mmol, 1.00 equiv.) and tributyl phosphine (480 µL, 1.85 mmol, 1.10 equiv.). The title compound **2m** was obtained in 49% yield (344 mg, 830 µmol) as a white powder.

^{1}H NMR (600 MHz, chloroform-d, 298 K): δ [ppm] = 8.35 (br s, 1H, Ar-H), 7.65 (br s, 1H, Ar- H), 7.32 (br s, 1H, Ar-H), 4.76 (br s, 1H, CH), 3.07 – 2.82 (m, 2H, CH_2), 2.74 – 2.46 (m, 6H, PBu_3-H), 2.35 – 1.85 (m, 4H, CH_2), 1.64 – 1.42 (m, 12H, PBu_3-H), 0.94 (m, 9H, PBu_3-H).

^{31}P NMR (243 MHz, chloroform-d, 298 K): δ [ppm] = 38.0.

Note: Due to fast exchange of the proton in the 8-position to form a pyridinium-ylide as well as inherent instability, characterization of the compound was limited to NMR Spectroscopy. The lifetime of the

compound was too short to measure a $^{13}C\{^1H\}$-NMR Spectrum and only ^1H and ^{31}P-Spectra could be acquired.

tributyl(di(pyridin-2-yl)methyl)phosphonium bromide (2n)

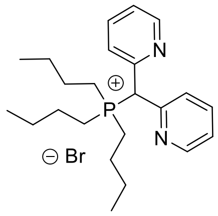

Synthesized according to the **GP4** employing 2,2'-(bromomethylene)dipyridine (329 mg, 1.32 mmol, 1.00 equiv.) and tributyl phosphine (377 µL, 1.45 mmol, 1.10 equiv.). The title compound **2n** was obtained in 62% yield (368 mg, 815 µmol) as a white powder.

m.p. = 155 – 156 °C

^{1}H NMR (501 MHz, methylene chloride-d_2, 298 K): δ [ppm] = 8.60 – 8.45 (m, 2H, Ar-*H*), 7.81 – 7.70 (m, 4H, Ar-*H*), 7.30 (ddt, *J* = 6.7, 5.1, 1.4 Hz, 2H, Ar-*H*), 7.04 (d, *J* = 15.5 Hz, 1H, C*H*), 2.61 – 2.41 (m, 6H, PBu_3-*H*), 1.54 – 1.29 (m, 12H, PBu_3-*H*), 0.87 (t, *J* = 7.2 Hz, 9H, PBu_3-*H*).

^{13}C{^{1}H} NMR (126 MHz, methylene chloride-d_2, 298 K): δ [ppm] = 155.4 (d, *J* = 6.3 Hz, *C*-Ar), 149.7 (*C*H-Ar), 138.4 (*C*H-Ar), 126.3 (d, *J* = 7.1 Hz, *C*H-Ar), 123.9 (*C*H-Ar), 48.7 (d, *J* = 49.4 Hz, *C*H), 24.6 (d, *J* = 5.1 Hz, *n*-Bu), 24.5 (d, *J* = 15.7 Hz, *n*-Bu), 20.8 (d, *J* = 46.1 Hz, *n*-Bu), 13.6 (*n*-Bu).

^{31}P NMR (203 MHz, methylene chloride-d_2, 298 K): δ [ppm] = 36.6.

IR (ATR) [cm^{-1}]: ṽ = 2958, 2931, 2871, 1585, 1570, 1467, 1431, 1379, 1333, 1309, 1230, 1146, 1098, 1050, 996, 972, 909, 817, 760, 748, 717, 658, 636, 618, 607.

HRMS-ESI(+): calc. $C_{23}H_{36}N_2P^+$ $[M]^+$ 371.2611; found 371.2606.

([2,2'-bipyridin]-6-ylmethyl)tributylphosphonium bromide (2p)

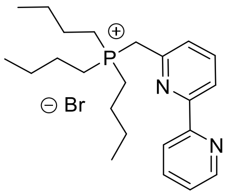

Synthesized according to the **GP4** employing 6-(bromomethyl)-2,2'-bipyridine (386 mg, 1.55 mmol, 1.00 equiv.) and tributyl phosphine (443 µL, 1.71 mmol, 1.10 equiv.). The title compound **2p** was obtained in 77% yield (542 mg, 1.20 mmol) as a white powder.

m.p. = 128 – 130 °C.

^{1}H NMR (501 MHz, methylene chloride-d_2, 298 K): δ [ppm] = 8.68 (d, *J* = 4.3 Hz, 1H, Ar-*H*), 8.41 – 8.37 (m, 1H, Ar-*H*), 8.23 (d, *J* = 8.0 Hz, 1H, Ar-*H*), 7.90 – 7.81 (m, 3H, Ar-*H*), 7.37 (t, *J* = 6.1 Hz, 1H, Ar-*H*), 4.49 (d, *J* = 15.1 Hz, 2H, CH_2), 2.54 – 2.43 (m, 6H, PBu_3-*H*), 1.62 – 1.51 (m, 6H, PBu_3-*H*), 1.51 – 1.40 (m, 6H, PBu_3-*H*), 0.92 (t, *J* = 7.3 Hz, 9H, PBu_3-*H*).

^{13}C{^{1}H} NMR (126 MHz, methylene chloride-d_2, 298 K): δ [ppm] = 156.6 (br, *C*-Ar), 155.5 (br, *C*-Ar), 150.9 (d, *J* = 9.0 Hz, *C*-Ar), 149.9 (br, *C*H-Ar), 139.2 (*C*H-Ar), 137.6 (br, *C*H-Ar), 126.4 (d, *J* = 5.4 Hz, *C*H-Ar), 124.8 (*C*H-Ar), 121.1 (*C*H-Ar), 120.9 (*C*H-Ar), 29.1 (d, *J* = 47.1 Hz, *C*H_2), 24.5 (d, *J* = 15.7 Hz, *n*-Bu), 24.2 (d, *J* = 4.8 Hz, *n*-Bu), 20.0 (d, *J* = 47.1 Hz, *n*-Bu), 13.7 (*n*-Bu).

^{31}P NMR (203 MHz, methylene chloride-d_2, 298 K): δ [ppm] = 33.2.

IR (ATR) [cm^{-1}]: ṽ = 3047, 2961, 2932, 2870, 1593, 1578, 1559, 1457, 1432, 1399, 1381, 1295, 1275, 1225, 1145, 1095, 1085, 1042, 991, 968, 913, 836, 792, 752, 714, 645, 622.

HRMS-ESI(+): calc. $C_{23}H_{36}N_2P^+$ $[M]^+$ 371.2611; found 371.2605.

benzyltributylphosphonium bromide (9a)

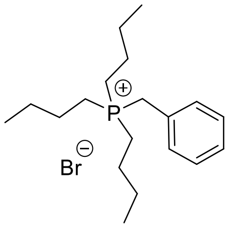

Synthesized according to the **GP4** employing benzyl bromide (855 mg, 5.00 mmol, 1.00 equiv.) and tributylphosphine (1.43 mL, 5.50 mmol, 1.10 equiv.). The title compound **9a** was obtained in 99% yield (1.84 g, 4.93 mmol) as a white powder.

^{1}H NMR (501 MHz, chloroform-*d*, 298 K): δ [ppm] = 7.49 – 7.40 (m, 2H), 7.37 – 7.27 (m, 3H), 4.26 (d, *J* = 15.1 Hz, 2H), 2.47 – 2.31 (m, 6H), 1.49 – 1.36 (m, 12H), 0.98 – 0.79 (m, 9H).

^{13}C{^{1}H} NMR (126 MHz, chloroform-*d*, 298 K): δ [ppm] = 130.3 (d, *J* = 5.1 Hz), 129.5 (d, *J* = 3.1 Hz), 128.6 (d, *J* = 8.6 Hz), 128.5 (d, *J* = 3.6 Hz), 27.1 (d, *J* = 45.2 Hz), 24.1 (d, *J* = 15.2 Hz), 23.8 (d, *J* = 4.9 Hz), 18.9 (d, *J* = 46.6 Hz), 13.5.

^{31}P NMR (203 MHz, chloroform-*d*, 298 K): δ [ppm] = 31.5.

The analytical data are consistent with the literature data.[12]

tributyl(4-(tert-butyl)benzyl)phosphonium bromide (9b)

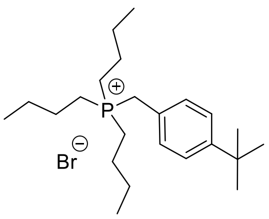

Synthesized according to the **GP4** employing 1-(bromomethyl)-4-(tert-butyl)benzene (227 mg, 1.00 mmol, 1.00 equiv.) and tributyl phosphine (286 µL, 1.10 mmol, 1.10 equiv.). The title compound **9b** was obtained in 97% yield (416 mg, 969 µmol) as a white powder.

m.p. = 208 – 209 °C

^{1}H NMR (501 MHz, chloroform-*d*, 298 K): δ [ppm] = 7.40 – 7.31 (m, 4H, Ar-*H*), 4.14 (d, *J* = 14.7 Hz, 2H, CH_2), 2.47 – 2.39 (m, 6H, PBu_3-*H*), 1.50 – 1.38 (m, 12H, PBu_3-*H*), 1.29 (s, 9H, *t*-Bu-*H*), 0.92 (t, *J* = 7.0 Hz, 9H, PBu_3-*H*).

^{13}C{^{1}H} NMR (126 MHz, chloroform-*d*, 298 K): δ [ppm] = 151.9 (d, *J* = 3.9 Hz, *C*-Ar), 129.9 (d, *J* = 5.0 Hz, *C*H-Ar), 126.5 (d, *J* = 3.3 Hz, *C*H-Ar), 125.2 (d, *J* = 8.7 Hz, *C*-Ar), 34.8 (*C*(CH_3)$_3$), 31.4 (C(*C*H_3)$_3$), 26.6 (d, *J* = 45.3 Hz, *C*H_2), 24.1 (d, *J* = 15.3 Hz, *n*-Bu), 23.9 (d, *J* = 4.9 Hz, *n*-Bu), 18.9 (d, *J* = 46.7 Hz, *n*-Bu), 13.6 (*n*-Bu).

^{31}P NMR (203 MHz, chloroform-*d*, 298 K): δ [ppm] = 31.5.

IR (ATR) [cm^{-1}]: ṽ = 3055, 2962, 2857, 2783, 1589, 1512, 1487, 1437, 1397, 1367, 1333, 1270, 1195, 1163, 1109, 1026, 998, 869, 835, 748, 720, 689, 616.

HRMS-ESI(+): calc. $C_{23}H_{42}P^+$ [M]$^+$ 349.3019; found 349.3031.

tributyl(4-methoxybenzyl)phosphonium chloride (9c)

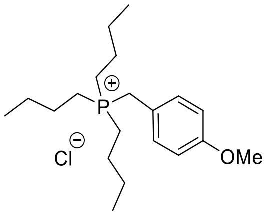

Synthesized according to the **GP4** employing 1-(chloromethyl)-4-methoxybenzene (235 mg, 1.50 mmol, 1.00 equiv.) and tributyl phosphine (334 µL, 1.65 mmol, 1.10 equiv.). The title compound **9c** was obtained in 83% yield (447 mg, 1.25 mmol) as a white powder.

m.p. = 79 – 80 °C

^{1}H NMR (501 MHz, chloroform-*d*, 298 K): δ [ppm] = 7.79 (d, *J* = 8.2 Hz, 2H, Ar-*H*), 7.25 (d, *J* = 8.2 Hz, 2H, Ar-*H*), 4.56 (d, *J* = 14.7 Hz, 2H, C*H*$_2$), 4.18 (s, 3H, OC*H*$_3$), 2.78 (t, *J* = 9.8 Hz, 6H, PBu$_3$-*H*), 1.85 (s, 12H, PBu$_3$-*H*), 1.31 (t, *J* = 6.9 Hz, 9H, PBu$_3$-*H*).

^{13}C{^{1}H} NMR (126 MHz, chloroform-*d*, 298 K): δ [ppm] = 159.1 (d, *J* = 3.4 Hz, *C*-Ar), 130.9 (d, *J* = 4.9 Hz, *C*H-Ar), 119.8 (d, *J* = 8.7 Hz, *C*-Ar), 114.3 (*C*H-Ar), 54.9 (O*C*H$_3$), 25.6 (d, *J* = 45.3 Hz, *C*H$_2$), 23.5 (d, *J* = 15.1 Hz, *n*-Bu), 23.2 (d, *J* = 4.9 Hz, *n*-Bu), 18.1 (d, *J* = 46.7 Hz, *n*-Bu), 13.0 (*n*-Bu).

^{31}P NMR (203 MHz, chloroform-*d*, 298 K): δ [ppm] = 31.2.

IR (ATR) [cm^{-1}]: ṽ = 2962, 2934, 2875, 1614, 1585, 1513, 1465, 1441, 1418, 1381, 1305, 1249, 1180, 1101, 1037, 997, 969, 917, 845, 750, 721, 687.

HRMS-ESI(+): calc. $C_{20}H_{36}OP^+$ [M]$^+$ 323.2498; found 323.2506

tributyl(3-bromobenzyl)phosphonium bromide (9d)

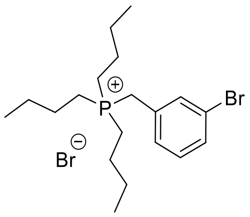

Synthesized according to the **GP4** employing 1-bromo-3-(bromomethyl)benzene (250 mg, 1.00 mmol, 1.00 equiv.) and tributyl phosphine (286 µL, 1.10 mmol, 1.10 equiv.). The title compound **9d** was obtained in 99% yield (448 mg, 991 µmol) as a white powder.

m.p. = 184 - 185 °C

^{1}H NMR (501 MHz, chloroform-*d*, 298 K): δ [ppm] = 7.59 (d, *J* = 7.7 Hz, 1H, Ar-*H*), 7.51 (q, *J* = 2.0 Hz, 1H, Ar-*H*), 7.48 – 7.44 (m, 1H, Ar-*H*), 7.25 (t, *J* = 7.9 Hz, 1H, Ar-*H*), 4.42 (d, *J* = 15.4 Hz, 2H, C*H*$_2$), 2.47-2.37 (m, 6H, PBu$_3$-*H*), 1.51 – 1.42 (m, 12H, PBu$_3$-*H*), 0.95 – 0.90 (m, 9H, PBu$_3$-*H*).

^{13}C{^{1}H} NMR (126 MHz, chloroform-*d*, 298 K): δ [ppm] = 132.7 (d, *J* = 4.9 Hz, *C*H-Ar), 131.8 (d, *J* = 3.7 Hz, *C*H-Ar), 131.2 (d, *J* = 8.4 Hz, *C*-Ar), 131.1 (*C*H-Ar), 129.4 (d, *J* = 5.1 Hz, *C*H-Ar), 123.3 (d, *J* = 3.8 Hz, *C*-Ar), 26.8 (d, *J* = 45.0 Hz, *C*H$_2$), 24.1 (d, *J* = 15.3 Hz, *n*-Bu), 23.9 (d, *J* = 4.9 Hz, *n*-Bu), 18.9 (d, *J* = 46.6 Hz, *n*-Bu), 13.6 (*n*-Bu).

^{31}P NMR (203 MHz, chloroform-*d*, 298 K): δ [ppm] = 31.9.

IR (ATR) [cm^{-1}]: ṽ = 3037, 3013, 2962, 2930, 2872, 2167, 1984, 1595, 1569, 1474, 1462, 1433, 1415, 1380, 1348, 1309, 1229, 1203, 1173, 1096, 1077, 998, 968, 916, 858, 804, 785, 725, 698, 667

HRMS-ESI(+): calc. $C_{19}H_{33}BrP^+$ $[M]^+$ 371.1498; found 371.1505.

tributyl(2-bromobenzyl)phosphonium bromide (9e)

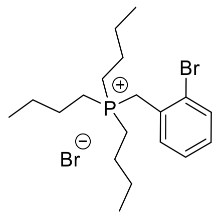

Synthesized according to the **GP4** employing 1-bromo-2-(bromomethyl)benzene (250 mg, 1.00 mmol, 1.00 equiv.) and tributyl phosphine (286 µL, 1.10 mmol, 1.10 equiv.). The title compound **9e** was obtained in 94% yield (425 mg, 940 µmol) as a white powder.

m.p. = 115 – 116 °C

^{1}H NMR (501 MHz, chloroform-*d*, 298 K): δ [ppm] = 8.00 (dt, *J* = 7.9, 2.1 Hz, 1H, Ar-*H*), 7.59 (dt, *J* =7.9, 1.1 Hz, 1H, Ar-*H*), 7.37 (tt, *J* = 7.6, 1.1 Hz, 1H, Ar-*H*), 7.25 – 7.18 (m, 1H, Ar-*H*), 4.47 (d, *J* = 15.4 Hz, 2H, C*H*$_2$), 2.59 – 2.40 (m, 6H, PBu$_3$-*H*), 1.55 – 1.32 (m, 12H, PBu$_3$-*H*), 0.92 (t, *J* = 7.1 Hz, 9H, PBu$_3$-*H*).

^{13}C{^{1}H} NMR (126 MHz, chloroform-*d*, 298 K): δ [ppm] = 133.5 (d, *J* = 3.1 Hz, *C*H-Ar), 133.4 (d, *J* = 4.6 Hz, *C*H-Ar), 130.4 (d, *J* = 3.5 Hz, *C*H-Ar), 129.4 (d, *J* = 9.0 Hz, *C*-Ar), 128.9 (d, *J* = 3.3 Hz, *C*H-Ar), 124.5 (d, *J* = 5.9 Hz, *C*-Ar), 27.4 (d, *J* = 45.8 Hz, *C*H$_2$), 24.1 (d, *J* = 15.5 Hz, *n*-Bu), 23.8 (d, *J* = 4.9 Hz, *n*-Bu), 19.4 (d, *J* = 45.9 Hz, *n*-Bu), 13.6 (*n*-Bu).

^{31}P NMR (203 MHz, chloroform-*d*, 298 K): δ [ppm] = 33.3.

IR (ATR) [cm^{-1}]: ṽ = 2963, 2933, 2874, 2167, 1568, 1471, 1432, 1401, 1383, 1318, 1293, 1262, 1237, 1195, 1157, 1101, 1078, 1048, 1025, 969, 916, 830, 797, 772, 722, 659

HRMS-ESI(+): calc. $C_{19}H_{33}BrP^+$ $[M]^+$ 371.1498; found 371.1491.

tributyl(3-cyanobenzyl)phosphonium bromide (9f)

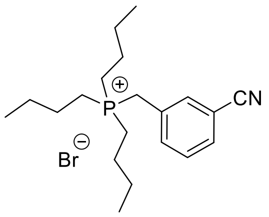

Synthesized according to the **GP4** employing 3-(bromomethyl)benzonitrile (196 mg, 1.00 mmol, 1.00 equiv.) and tributyl phosphine (286 µL, 1.10 mmol, 1.10 equiv.). The title compound **9f** was obtained in 94% yield (375 mg, 941 µmol) as a white powder.

m.p. = 171 – 172 °C

^{1}H NMR (501 MHz, chloroform-*d*, 298 K): δ [ppm] = 8.11 (d, *J* = 7.9 Hz, 1H, Ar-*H*), 7.70 (s, 1H, Ar-*H*), 7.62 (d, *J* = 7.7 Hz, 1H, Ar-*H*), 7.52 (t, *J* = 7.8 Hz, 1H, Ar-*H*), 4.66 (d, *J* = 15.7 Hz, 2H, CH_2), 2.47 – 2.39 (m, 6H, PBu_3-*H*), 1.51 – 1.41 (m, 12H, PBu_3-*H*), 0.96 – 0.87 (m, 9H, PBu_3-*H*).

^{13}C{^{1}H} NMR (126 MHz, chloroform-*d*, 298 K): δ [ppm] = 135.6 (d, *J* = 4.8 Hz, *C*-Ar), 133.0 (d, *J* = 5.1 Hz, *C*H-Ar), 132.2 (d, *J* = 3.3 Hz, *C*H-Ar), 131.2 (d, *J* = 8.6 Hz, *C*-Ar), 130.6 (d, *J* = 2.9 Hz, *C*H-Ar), 118.0 (*C*N), 113.5 (d, *J* = 3.2 Hz, *C*H-Ar), 26.7 (d, *J* = 45.0 Hz, *C*H_2), 24.1 (d, *J* = 15.3 Hz, *n*-Bu), 23.9 (d, *J* = 4.8 Hz, *n*-Bu), 18.9 (d, *J* = 46.3 Hz, *n*-Bu), 13.5 (*n*-Bu).

^{31}P NMR (203 MHz, chloroform-*d*, 298 K): δ [ppm] = 32.0.

IR (ATR) [cm^{-1}]: ṽ = 3017, 2964, 2936, 2919, 2875, 2234, 2166, 1603, 1583, 1487, 1463, 1436, 1415, 1383, 1349, 1269, 1227, 1179, 1096, 1002, 968, 913, 874, 860, 819, 697, 615

HRMS-ESI(+): calc. $C_{20}H_{33}NP^+$ $[M]^+$ 318.2345; found 318.2349.

tributyl(4-(trifluoromethyl)benzyl)phosphonium bromide (9g)

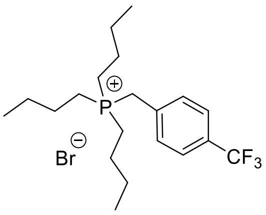

Synthesized according to the **GP4** employing 1-(bromomethyl)-4-(trifluoromethyl)benzene (239 mg, 1.00 mmol, 1.00 equiv.) and tributyl phosphine (286 µL, 1.10 mmol, 1.10 equiv.). The title compound **9g** was obtained in 97% yield (429 mg, 972 µmol) as a white powder.

m.p. = 158 – 159 °C

^{1}H NMR (501 MHz, chloroform-*d*, 298 K): δ [ppm] = 7.69 (dd, *J* = 8.2, 2.4 Hz, 2H, Ar-*H*), 7.59 (d, *J* = 8.0 Hz, 2H, Ar-*H*), 4.61 (d, *J* = 15.9 Hz, 2H, CH_2), 2.45 – 2.36 (m, 6H, PBu_3-*H*), 1.50 – 1.41 (m, 12H, PBu_3-*H*), 0.95 – 0.88 (m, 9H, PBu_3-*H*).

^{13}**C{^{1}H} NMR** (126 MHz, chloroform-*d*, 298 K): δ [ppm] = 133.3 (d, *J* = 8.6 Hz, *C*-Ar), 130.90 (*C*H-Ar), 130.87 (m, *C*-Ar), 126.33 (q, *J* = 3.7 Hz, *C*H-Ar), 123.8 (q, *J* = 273.5 Hz, *C*F_3), 27.0 (d, *J* = 45.0 Hz, *C*H_2), 24.1 (*n*-Bu), 23.9 (d, *J* = 4.9 Hz, *n*-Bu), 18.9 (d, *J* = 46.4 Hz, *n*-Bu), 13.5 (*n*-Bu).

^{31}**P NMR** (203 MHz, chloroform-*d*, 298 K): δ [ppm] = 31.9.

^{19}**F NMR** (565 MHz, chloroform-*d*, 298 K): δ [ppm] = –62.80 (d, *J* = 2.5 Hz).

IR (ATR) [cm^{-1}]: ṽ = 2966, 2939, 2878, 1620, 1464, 1419, 1384, 1325, 1264, 1198, 1159, 1123, 1109, 1069, 1023, 970, 916, 899, 858, 808, 718, 624

HRMS-ESI(+): calc. $C_{20}H_{33}F_3P^+$ [M]$^+$ 361.2266; found 361.2274.

General procedure for pyridyl triazole synthesis (GP5)

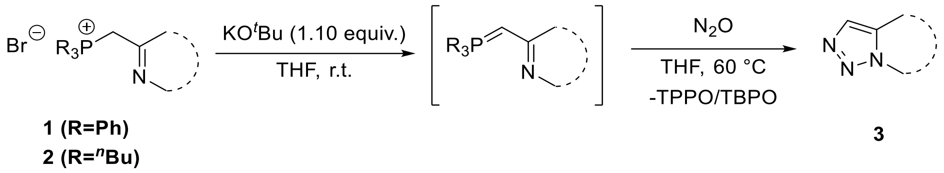

To a dried pressure Schlenk-flask equipped with a magnetic stirring bar, a suspension of phosphonium salt (1.00 equiv.) was prepared in anhydrous THF (5 mL) under argon atmosphere. To this suspension, potassium *tert*-butoxide (1.10 equiv.) was added under argon counterflow. The solution was stirred at ambient temperature for 30 min. The reaction mixture was then cooled to –78 °C and the inert gas was exchanged for 1 atmosphere of N_2O (Note: N_2O was directly used from the gas bottle; quality 5.0 obtained from Messer) by evacuating the pressure Schlenk-flask until gas evolution from the solution at –78 °C has ceased then repressurizing it with N_2O (1 bar). After gas exchange, the flask was sealed and allowed to warm slowly to the reaction temperature specified in Scheme 2, in the absence of light. The reaction was stirred at this temperature for 48 h (Caution: the actual pressure in the flask at room temperature is higher; use pressure glassware and explosion shield). The crude mixture was cooled down to ambient temperature and liberate the excess of nitrous oxide. The resulting mixture was filtered and directly adsorbed onto silica gel for further purification by flash chromatography to give the corresponding heterocyclic product.

[1,2,3]triazolo[1,5-a]pyridine (3a)

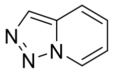

Synthesized according to the **GP5** employing triphenyl(pyridin-2-ylmethyl)phosphonium bromide **1a** (86.9 mg, 200 µmol) and potassium *tert*-butoxide (24.7 mg, 220 µmol). The title compound **3a** was

purified by flash column chromatography on silica gel (EtOAc/CyHex = 30/70, R_f = 0.32) and obtained in 99% yield (23.6 mg, 198 µmol) as a white powder.

The same product was also obtained using tributyl(pyridin-2-ylmethyl)phosphonium bromide **2a** (1.87 g, 5.00 mmol) and potassium *tert*-butoxide (617 mg, 5.50 mmol) in 10 mL of THF. The reaction was carried out at ambient temperature for 48 hours after introducing N_2O. Following the same workup and purification procedure, the desired compound **3a** was isolated in 89% yield (530 mg, 4.45 mmol).

^{1}H NMR (501 MHz, chloroform-*d*, 298 K): δ [ppm] = 8.74 (d, *J* = 7.0 Hz, 1H), 8.06 (s, 1H), 7.73 (d, *J* = 8.9 Hz, 1H), 7.24 (dt, *J* = 8.9, 6.7 Hz, 1H), 6.98 (t, *J* = 6.7 Hz, 1H).

^{13}C{^{1}H} NMR (126 MHz, chloroform-*d*, 298 K): δ [ppm] = 133.8, 125.7, 125.33, 125.26, 118.1, 115.3.

The analytical data are consistent with the literature data.[13]

6-bromo-[1,2,3]triazolo[1,5-a]pyridine (3b)

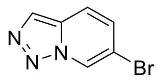

Synthesized according to the **GP5** employing ((5-bromopyridin-2-yl)methyl)triphenylphosphonium bromide **1b** (103 mg, 200 µmol) and potassium *tert*-butoxide (24.7 mg, 220 µmol). The title compound **3b** was purified by column chromatography on silica gel (EtOAc/CyHex = 30/70, R_f = 0.38) and obtained in 97% yield (38.4 mg, 194 µmol) as a white powder.

^{1}H NMR (501 MHz, chloroform-*d*, 298 K): δ [ppm] = 8.91 (dd, *J* = 1.5, 1.0 Hz, 1H), 8.07 (d, *J* = 1.0 Hz, 1H), 7.64 (dd, *J* = 9.3, 1.0 Hz, 1H), 7.32 (dd, *J* = 9.3, 1.5 Hz, 1H).

^{13}C{^{1}H} NMR (126 MHz, chloroform-*d*, 298 K): δ [ppm] = 132.4, 129.2, 126.4, 125.6, 118.4, 110.8.

The analytical data are consistent with the literature data.[14]

4-bromo-[1,2,3]triazolo[1,5-a]pyridine (3c)

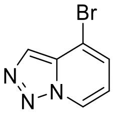

Synthesized according to the **GP5** employing ((3-bromopyridin-2-yl)methyl)triphenylphosphonium bromide **1c** (103 mg, 200 µmol) and potassium *tert*-butoxide (24.7 mg, 220 µmol). The title compound **3c** was purified by column chromatography on silica gel (EtOAc:CyHex = 30:70, R_f = 0.38) and obtained in 96% yield (38.0 mg, 192 µmol) as a white powder.

^{1}H NMR (501 MHz, chloroform-*d*, 298 K): δ [ppm] = 8.71 (d, *J* = 7.0 Hz, 1H), 8.11 (s, 1H), 7.43 (d, *J* = 7.1 Hz, 1H), 6.87 (t, *J* = 7.1 Hz, 1H).

^{13}C{^{1}H} NMR (126 MHz, chloroform-*d*, 298 K): δ [ppm] = 134.8, 127.8, 127.1, 124.4, 115.5, 111.6.

The analytical data are consistent with the literature data.[15]

5-methoxy-[1,2,3]triazolo[1,5-a]pyridine (3d)

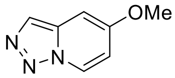

Synthesized according to the **GP5** employing ((4-methoxypyridin-2-yl)methyl)triphenylphosphonium bromide **1d** (46.4 mg, 100 µmol) and potassium *tert*-butoxide (12.3 mg, 110 µmol). The title compound **3d** was purified by column chromatography on silica gel (EtOAc/CyHex = 30/70, R_f = 0.21) and obtained in 95% yield (14.2 mg, 95.2 µmol) as a white powder.

^{1}H NMR (501 MHz, chloroform-*d*, 298 K): δ [ppm] = 8.55 (d, *J* = 7.6 Hz, 1H), 7.84 (s, 1H), 6.85 (d, *J* = 2.5 Hz, 1H), 6.66 (dd, *J* = 7.6, 2.5 Hz, 1H), 3.88 (s, 3H).

^{13}C{^{1}H} NMR (126 MHz, chloroform-*d*, 298 K): δ [ppm] = 157.6, 135.1, 126.1, 124.3, 110.8, 93.6, 55.9.

The analytical data are consistent with the literature data.[16]

7-methyl-[1,2,3]triazolo[1,5-a]pyridine (3e)

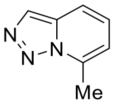

Synthesized according to the **GP5** employing ((6-methylpyridin-2-yl)methyl)triphenylphosphonium bromide **1e** (89.7 mg, 200 µmol) and potassium *tert*-butoxide (24.7 mg, 220 µmol). The title compound **3e** was purified by column chromatography on silica gel (EtOAc/CyHex = 20/80, R_f = 0.34) and obtained in 99% yield (26.6 mg, 199 µmol) as a white powder.

^{1}H NMR (501 MHz, chloroform-*d*, 298 K): δ [ppm] = 8.09 (s, 1H), 7.63 (d, *J* = 8.9 Hz, 1H), 7.19 (dd, *J* = 8.9, 6.7 Hz, 1H), 6.78 (dt, *J* = 6.7, 1.1 Hz, 1H), 2.89 (s, 3H).

^{13}C{^{1}H} NMR (126 MHz, chloroform-*d*, 298 K): δ [ppm] = 136.0, 134.1, 126.2, 125.3, 115.5, 114.1, 17.6.

The analytical data are consistent with the literature data.[14]

[1,2,3]triazolo[1,5-a]quinoline (3f)

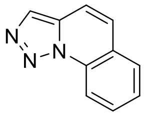

Synthesized according to the **GP5** employing triphenyl(quinolin-2-ylmethyl)phosphonium bromide **1f** (96.9 mg, 200 µmol) and potassium *tert*-butoxide (24.7 mg, 220 µmol). The title compound **3f** was purified by column chromatography on silica gel (EtOAc/CyHex = 20/80, R_f = 0.36) and obtained in 98% yield (33.2 mg, 196 µmol) as a white powder.

^{1}H NMR (501 MHz, chloroform-*d*, 298 K): δ [ppm] = 8.80 (d, *J* = 8.3 Hz, 1H), 8.11 (s, 1H), 7.85 (dd, *J* = 8.2, 1.3 Hz, 1H), 7.77 (ddd, *J* = 8.2, 7.3, 1.3 Hz, 1H), 7.61 (ddd, *J* = 8.2, 7.3, 1.3 Hz, 1H), 7.55 (d, *J* = 9.3 Hz, 1H), 7.52 (d, *J* = 9.3 Hz, 1H).

^{13}C{^{1}H} NMR (126 MHz, chloroform-*d*, 298 K): δ [ppm] = 132.0, 131.9, 130.2, 128.7, 127.7, 127.3, 126.8, 124.0, 116.5, 114.9.

The analytical data are consistent with the literature data.[17]

8-chloro-[1,2,3]triazolo[1,5-a]quinoline (3g)

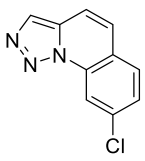

Synthesized according to the **GP5** employing ((7-chloroquinolin-2-yl)methyl)triphenylphosphonium bromide **1g** (104 mg, 200 µmol) and potassium *tert*-butoxide (24.7 mg, 220 µmol). The title compound

3g was purified by column chromatography on silica gel (EtOAc/CyHex = 20/80, R_f = 0.33) and obtained in 92% yield (37.5 mg, 184 µmol) as a white powder.

^{1}H NMR (501 MHz, chloroform-*d*, 298 K): δ [ppm] = 8.81 (d, *J* = 2.0 Hz, 1H), 8.12 (s, 1H), 7.78 (d, *J* = 8.5 Hz, 1H), 7.58 – 7.54 (m, 2H), 7.50 (d, *J* = 9.3 Hz, 1H).

^{13}C{^{1}H} NMR (126 MHz, chloroform-*d*, 298 K): δ [ppm] = 136.3, 132.4, 132.0, 129.8, 128.0, 127.9, 126.2, 122.4, 116.6, 115.2.

The analytical data are consistent with the literature data.[17]

[1,2,3]triazolo[1,5-b]isoquinoline (3h)

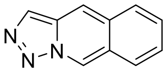

Synthesized according to the **GP5** employing (isoquinolin-3-ylmethyl)triphenylphosphonium bromide **1h** (96.9 mg, 200 µmol) and potassium *tert*-butoxide (24.7 mg, 220 µmol). The title compound **3h** was purified by column chromatography on silica gel (EtOAc/CyHex = 30/70, R_f = 0.26) and obtained in 86% yield (29.1 mg, 172 µmol) as a white powder.

m.p. = 156 – 157 °C.

^{1}H NMR (501 MHz, chloroform-*d*, 298 K): δ [ppm] = 9.41 (s, 1H, Ar-*H*), 9.05 (s, 1H, Ar-*H*), 8.40 (s, 1H, Ar-*H*), 8.07 (d, *J* = 8.2 Hz, 1H, Ar-*H*), 7.99 (d, *J* = 8.2 Hz, 1H, Ar-*H*), 7.81 (t, *J* = 7.6 Hz, 1H, Ar-*H*), 7.73 (t, *J* = 7.6 Hz, 1H, Ar-*H*).

^{13}C{^{1}H} NMR (126 MHz, chloroform-*d*, 298 K): δ [ppm] = 162.8 (*C*H-Ar), 153.1 (*C*H-Ar), 136.1 (*C*-Ar), 131.5 (*C*H-Ar), 129.4 (*C*-Ar), 129.1 (*C*H-Ar), 128.1 (*C*H-Ar), 127.8 (*C*H-Ar), 122.2 (*C*H-Ar).

IR (ATR) [cm^{-1}]: ṽ = 2924, 2874, 1656, 1608, 1592, 1532, 1504, 1480, 1458, 1422, 1386, 1330, 1276, 1258, 1219, 1106, 1052, 963, 934, 844, 798, 751, 689.

HRMS-ESI(+): calc. $C_{10}H_8N_3^+$ [M+H]$^+$ 170.0713; found 170.0715.

[1,2,3]triazolo[5,1-a]isoquinoline (3i)

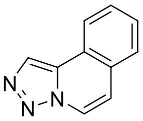

Synthesized according to the **GP5** employing (isoquinolin-1-ylmethyl)triphenylphosphonium bromide **1i** (96.9 mg, 200 µmol) and potassium *tert*-butoxide (24.7 mg, 220 µmol). The title compound **3i** was

purified by column chromatography on silica gel (EtOAc/CyHex = 30/70, R_f = 0.28) and obtained in 95% yield (32.1 mg, 190 µmol) as a white powder.

^{1}H NMR (501 MHz, chloroform-*d*, 298 K): δ [ppm] = 8.53 (d, *J* = 7.4 Hz, 1H), 8.43 (s, 1H), 8.17 (dd, *J* = 7.6, 1.5 Hz, 1H), 7.82 (dd, *J* = 7.6, 1.5 Hz, 1H), 7.68 (td, *J* = 7.5, 1.6 Hz, 1H), 7.65 (td, *J* = 7.5, 1.6 Hz, 1H), 7.21 (d, *J* = 7.5 Hz, 1H).

^{13}C{^{1}H} NMR (126 MHz, chloroform-*d*, 298 K): δ [ppm] = 132.7, 129.3, 129.1, 127.8, 126.0, 124.3, 123.2, 122.9, 116.2.

The analytical data are consistent with the literature data.[17]

[1,2,3]triazolo[1,5-a]quinoxaline (3j)

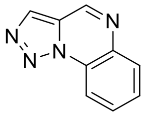

Synthesized according to the **GP5** employing triphenyl(quinoxalin-2-ylmethyl)phosphonium bromide **1j** (97.1 mg, 200 µmol) and potassium *tert*-butoxide (24.7 mg, 220 µmol). The diazo transfer was carried out at 70 °C. The title compound **3j** was purified by column chromatography on silica gel (EtOAc/CyHex = 20/80, R_f = 0.34) and obtained in 88% yield (29.8 mg, 175 µmol) as a colorless oil.

^{1}H NMR (501 MHz, chloroform-*d*, 298 K): δ [ppm] = 9.24 (s, 1H), 8.74 (dd, *J* = 8.2, 1.5 Hz, 1H), 8.41 (s, 1H), 8.20 (dd, *J* = 8.2, 1.5 Hz, 1H), 7.82 (ddd, *J* = 8.4, 7.3, 1.5 Hz, 1H), 7.77 (ddd, *J* = 8.4, 7.3, 1.5 Hz, 1H).

^{13}C{^{1}H} NMR (126 MHz, chloroform-*d*, 298 K): δ [ppm] = 143.3, 136.6, 130.7, 130.4, 129.6, 129.1, 127.2, 126.3, 116.0.

The analytical data are consistent with the literature data.[17]

[1,2,3]triazolo[1,5-a][1,5]naphthyridine (3k)

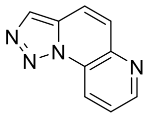

Synthesized according to the **GP5** employing ((1,5-naphthyridin-2-yl)methyl)tributylphosphonium bromide **2k** (85.1 mg, 200 µmol) and potassium *tert*-butoxide (24.7 mg, 220 µmol). The title compound **3k** was purified by column chromatography on silica gel (EtOAc/CyHex = 20/80, R_f = 0.34) and obtained in 76% yield (25.7 mg, 151 µmol) as a yellowish solid.

m.p. = 128 – 129 °C.

^{1}H NMR (501 MHz, chloroform-*d*, 298 K): δ [ppm] = 9.08 (dd, *J* = 8.4, 1.7 Hz, 1H, Ar-*H*), 8.95 (dd, *J* = 4.5, 1.6 Hz, 1H, Ar-*H*), 8.16 (s, 1H, Ar-*H*), 7.82 – 7.76 (m, 2H, Ar-*H*), 7.69 (dd, *J* = 8.4, 4.5 Hz, 1H, Ar-*H*).

^{13}C{^{1}H} NMR (126 MHz, chloroform-*d*, 298 K): δ [ppm] = 150.1 (*C*H-Ar), 141.5 (*C*-Ar), 131.5 (*C*-Ar), 128.8 (*C*H-Ar), 128.5 (*C*H-Ar), 128.0 (*C*H-Ar), 124.3 (*C*H-Ar), 118.5 (*C*H-Ar).

IR (ATR) [cm^{-1}]: ṽ = 3127, 3072, 2959, 2923, 2859, 1690, 1605, 1543, 1459, 1411, 1285, 1230, 1180, 1097, 1048, 1012, 964, 819, 762, 682, 610, 546, 465

HRMS-APCI(+): calc. $C_9H_7N_4^+$ [M+H]$^+$ 171.0665; found 171.0661.

[1,2,3]triazolo[1,5-a]pyrimidine (3l)

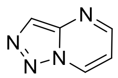

Synthesized according to the **GP5** employing tributyl(pyrimidin-2-ylmethyl)phosphonium chloride **2l** (66.2 mg, 200 µmol) and potassium *tert*-butoxide (24.7 mg, 220 µmol). The title compound **3l** was purified by column chromatography on silica gel (EtOAc/CyHex = 30/70, R_f = 0.23) and obtained in 63% yield (15.2 mg, 127 µmol) as a white powder.

m.p. = 165 – 166 °C

^{1}H NMR (600 MHz, chloroform-*d*, 300 K): δ [ppm] = 8.96 (ddd, *J* = 7.1, 1.7, 1.0 Hz, 1H, Ar-*H*), 8.67 (dd, *J* = 3.9, 1.7 Hz, 1H, Ar-*H*), 8.29 (d, *J* = 1.1 Hz, 1H, Ar-*H*), 7.03 (dd, *J* = 7.1, 3.9 Hz, 1H, Ar-*H*).

^{13}C{^{1}H} NMR (151 MHz, chloroform-*d*, 300 K): δ [ppm] = 151.7 (*C*H-Ar), 143.5 (*C*-Ar), 132.1 (*C*H-Ar), 126.1 (*C*H-Ar), 110.7 (*C*H-Ar).

IR (ATR) [cm^{-1}]: ṽ = 3735, 3649, 3102, 3078, 1615, 1558, 1534, 1521, 1457, 1407, 1337, 1170, 1143, 1099, 1029, 956, 826, 807, 767, 569, 434.

HRMS-ESI(+): calc. $C_5H_5N_4^+$ [M+H]$^+$ 121.0509; found 121.0510.

8,9-dihydro-7H-[1,2,3]triazolo[4,5,1-ij]quinoline (3m)

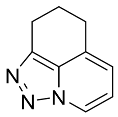

Synthesized according to the **GP5** employing tributyl(5,6,7,8-tetrahydroquinolin-8-yl)phosphonium bromide **2m** (82.9 mg, 200 µmol) and potassium *tert*-butoxide (24.7 mg, 220 µmol). The title compound **3m** was purified by column chromatography on silica gel (EtOAc/CyHex = 30/70, R_f = 0.31) and obtained in 67% yield (21.4 mg, 134 µmol) as a white powder.

^{1}H NMR (501 MHz, chloroform-*d*, 298 K): δ [ppm] = 8.46 (dq, *J* = 7.1, 0.9 Hz, 1H), 6.88 (t, *J* = 6.7 Hz, 1H), 6.83 (dq, *J* = 6.6, 1.1 Hz, 1H), 3.13 (t, *J* = 6.1 Hz, 2H), 2.95 (t, *J* = 6.1 Hz, 2H), 2.16 (p, *J* = 6.2 Hz, 2H).

^{13}C{^{1}H} NMR (126 MHz, chloroform-*d*, 298 K): δ [ppm] = 138.0, 132.5, 132.1, 122.1, 118.3, 116.7, 26.7, 23.9, 22.5.

The analytical data are consistent with the literature data.[18]

3-(pyridin-2-yl)-[1,2,3]triazolo[1,5-a]pyridine (3n)

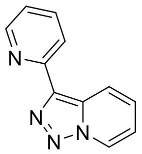

Synthesized according to the **GP5** employing tributyl(di(pyridin-2-yl)methyl)phosphonium bromide **2n** (90.3 mg, 200 µmol) and *n*-butyllithium (138 µL, 1.6 M, 220 µmol). The deprotonation was carried out at 0 °C. The title compound **3n** was purified by column chromatography on silica gel (EtOAc/CyHex = 30/70, R_f = 0.29) and obtained in 46% yield (18.1 mg, 92.3 µmol) as a white powder.

^{1}H NMR (600 MHz, chloroform-*d*, 298 K): δ [ppm] = 8.77 (dt, *J* = 7.0, 1.2 Hz, 1H), 8.74 (dt, *J* = 9.0, 1.2 Hz, 1H), 8.67 (ddd, *J* = 4.9, 1.9, 1.0 Hz, 1H), 8.36 (dt, *J* = 7.9, 1.2 Hz, 1H), 7.80 (td, *J* = 7.9, 1.9 Hz, 1H), 7.38 (ddd, *J* = 9.0, 6.7, 1.0 Hz, 1H), 7.22 (ddd, *J* = 7.5, 4.9, 1.2 Hz, 1H), 7.05 (td, *J* = 6.7, 1.2 Hz, 1H).

^{13}C{^{1}H} NMR (151 MHz, chloroform-*d*, 298 K): δ [ppm] = 152.2, 149.5, 137.6, 136.8, 132.2, 126.5, 125.4, 122.2, 121.5, 120.6, 116.0.

The analytical data are consistent with the literature data.[13]

7-phenyl-[1,2,3]triazolo[1,5-c]pyrimidine (3o)

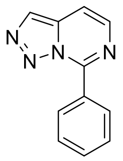

Synthesized according to the **GP5** employing triphenyl((2-phenylpyrimidin-4-yl)methyl)phosphonium bromide **1o** (102 mg, 200 µmol) and potassium *tert*-butoxide (24.7 mg, 220 µmol). The diazo transfer was carried out at 70 °C. The title compound **3o** was purified by column chromatography on silica gel (EtOAc/CyHex = 40:60, R_f = 0.28) and obtained in 83% yield (32.6 mg, 166 µmol) as a white powder.

m.p. = 190 – 191 °C.

^{1}H NMR (501 MHz, chloroform-*d*, 298 K): δ [ppm] = 8.87 (d, *J* = 5.0 Hz, 1H, Ar-*H*), 8.60 – 8.53 (m, 2H, Ar-*H*), 8.02 (s, 1H, Ar-*H*), 7.54 (dd, *J* = 5.2, 2.1 Hz, 3H, Ar-*H*), 7.33 (d, *J* = 5.0 Hz, 1H, Ar-*H*).

^{13}C{^{1}H} NMR (126 MHz, chloroform-*d*, 298 K): δ [ppm] = 164.9 (*C*-Ar), 161.1 (*C*-Ar), 158.6 (*C*H-Ar), 137.8 (*C*-Ar), 134.2 (*C*H-Ar), 131.0 (*C*H-Ar), 128.8 (*C*H-Ar), 128.5 (*C*H-Ar), 118.0 (*C*H-Ar).

IR (ATR) [cm^{-1}]: ṽ = 2961, 2923, 2853, 1587, 1545, 1486, 1458, 1427, 1385, 1337, 1312, 1258, 1197, 1162, 1087, 1024, 984, 930, 908, 847, 794, 759, 736, 687, 674, 644, 617, 567.

HRMS-ESI(+): calc. $C_{11}H_9N_2$ [M-N$_2$+H]$^+$ 169.0760; found 169.0753.

7-(pyridin-2-yl)-[1,2,3]triazolo[1,5-a]pyridine (3p)

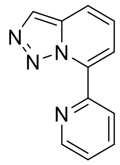

Synthesized according to the **GP5** employing ([2,2'-bipyridin]-6-ylmethyl)tributylphosphonium bromide **2p** (90.3 mg, 200 µmol) and potassium *tert*-butoxide (24.7 mg, 220 µmol). The diazo transfer was carried out at ambient temperature. The title compound **3p** was purified by column chromatography on silica gel (EtOAc/CyHex = 30/70, R_f = 0.24) and obtained in 79% yield (31.1 mg, 159 µmol) as a white powder.

^{1}H NMR (501 MHz, chloroform-*d*, 298 K): δ [ppm] = 9.01 (dt, *J* = 8.1, 1.1 Hz, 1H), 8.79 (ddd, *J* = 4.7, 1.8, 0.9 Hz, 1H), 8.21 (s, 1H), 7.92 (ddd, *J* = 9.3, 7.5, 1.6 Hz, 2H), 7.82 (dd, *J* = 8.8, 1.3 Hz, 1H), 7.46 – 7.39 (m, 2H).

^{13}C{^{1}H} NMR (126 MHz, chloroform-*d*, 298 K): δ [ppm] = 149.9, 149.3, 136.9, 136.8, 135.2, 126.2, 125.7, 125.3, 124.6, 118.1, 116.8.

The analytical data are consistent with the literature data.[19]

General procedure for pyridyl triazole synthesis (GP6)

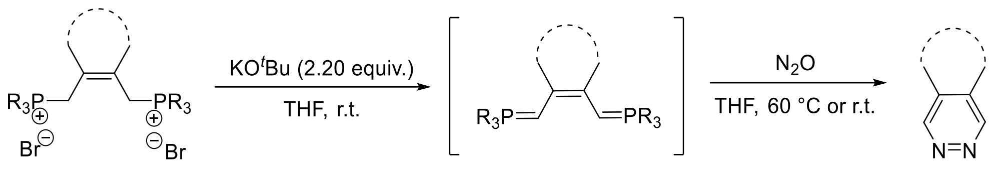

5

To a dried pressure Schlenk-flask equipped with a magnetic stirring bar, a suspension of phosphonium salt (1.00 equiv.) was prepared in anhydrous THF (5 mL) under argon atmosphere. To this suspension, potassium *tert*-butoxide (2.20 equiv.) was added under argon counterflow. The solution was stirred at ambient temperature for 3 hours. The solution was cooled to –78 °C and the inert gas was exchanged for 1 atmosphere of N_2O (Note: N_2O was directly used from the gas bottle; quality 5.0 obtained from Messer) by evacuating the pressure Schlenk-flask until gas evolution from the solution at –78 °C has ceased then repressurizing it with N_2O (1 bar). After repressurizing with N_2O at –78 °C, the flask was closed and slowly warm up to the reaction temperature specified in Scheme 3, in the dark and the reaction was stirred at this temperature for another 48 hours. (Caution: the actual pressure in the flask at room temperature is higher; use pressure glassware and explosion shield). The crude mixture was cooled down to ambient temperature and liberate the excess of nitrous oxide. The resulting mixture was filtered and directly adsorbed onto silica gel for further purification by flash chromatography to give the corresponding heterocyclic product.

pyrazino[2,3-d]pyridazine (5a)

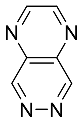

Synthesized according to the **GP6** employing (pyrazine-2,3-diylbis(methylene))bis(triphenylphosphonium) bromide **4a** (395 mg, 500 µmol) and potassium *tert*-butoxide (123 mg, 1.10 mmol). The title compound **5a** was purified by column chromatography on silica gel (EtOAc/CyHex = 50/50, R_f = 0.18) and obtained in 99% yield (65.7 mg, 497 µmol) as a white powder.

^{1}H NMR (501 MHz, chloroform-*d*, 298 K): δ [ppm] = 9.90 (s, 2H), 9.23 (s, 2H).

^{13}C{^{1}H} NMR (126 MHz, chloroform-*d*, 298 K): δ [ppm] = 153.1, 151.5, 137.2.

The analytical data are consistent with the literature data.[20]

phthalazine (5b)

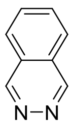

Synthesized according to the **GP6** employing (1,2-phenylenebis(methylene))bis(triphenylphosphonium) bromide **4b** (158 mg, 200 µmol) and potassium *tert*-butoxide (49.4 mg, 440 µmol). The title compound **5b** was purified by column chromatography on silica gel (EtOAc/CyHex = 50/50, R_f = 0.14) and obtained in 98% yield (25.4 mg, 195 µmol) as a white powder.

^{1}H NMR (501 MHz, chloroform-*d*, 298 K): δ [ppm] = 9.54 (s, 2H), 7.99 – 7.89 (m, 4H).

^{13}C{^{1}H} NMR (126 MHz, chloroform-*d*, 298 K): δ [ppm] = 151.2, 132.8, 126.7, 126.4.

The analytical data are consistent with the literature data.[21]

6-bromophthalazine (5c)

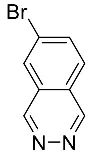

Synthesized according to the **GP6** employing ((4-bromo-1,2-phenylene)bis(methylene))bis(triphenylphosphonium) bromide **4c** (173 mg, 200 µmol) and potassium *tert*-butoxide (49.4 mg, 440 µmol). The title compound **5c** was purified by column chromatography on silica gel (EtOAc/CyHex = 50/50, R_f = 0.13) and obtained in 85% yield (35.6 mg, 170 µmol) as a white powder.

^{1}H NMR (501 MHz, chloroform-*d*, 298 K): δ [ppm] = 9.53 (dd, *J* = 1.6, 0.8 Hz, 1H), 9.49 (dd, *J* = 1.5, 0.8 Hz, 1H), 8.15 (dt, *J* = 1.6, 0.7 Hz, 1H), 8.03 (dd, *J* = 8.6, 1.8 Hz, 1H), 7.86 (dt, *J* = 8.6, 0.8 Hz, 1H).

^{13}C{^{1}H} NMR (126 MHz, chloroform-*d*, 298 K): δ [ppm] = 150.8, 150.0, 136.5, 128.9, 128.1, 127.6, 127.2, 125.1.

The analytical data are consistent with the literature data.[22]

6,7-dimethoxyphthalazine (5d)

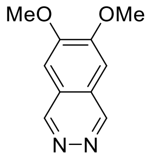

Synthesized according to the **GP6** employing ((4,5-dimethoxy-1,2-phenylene)bis(methylene))bis(triphenylphosphonium) bromide **4d** (170 mg, 200 µmol) and potassium *tert*-butoxide (49.4 mg, 440 µmol). The diazo transfer was carried out at ambient temperature. The title compound **5d** was purified by column chromatography on silica gel ($MeOH/CH_2Cl_2$ = 1/25, R_f = 0.32) and obtained in 76% yield (28.8 mg, 151 µmol) as a yellowish solid.

^{1}H NMR (501 MHz, chloroform-*d*, 298 K): δ [ppm] = 9.37 (s, 2H), 7.18 (s, 2H), 4.08 (s, 6H).

^{13}C{^{1}H} NMR (126 MHz, chloroform-*d*, 298 K): δ [ppm] = 154.4, 149.5, 123.4, 104.4, 56.6.

The analytical data are consistent with the literature data.[23]

methyl phthalazine-5-carboxylate (5e)

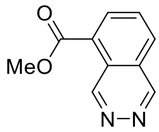

Synthesized according to the **GP6** employing ((3-(methoxycarbonyl)-1,2-phenylene)bis(methylene))bis(triphenylphosphonium) bromide **4e** (169 mg, 200 µmol) and potassium *tert*-butoxide (49.4 mg, 440 µmol). The title compound **5e** was purified by column chromatography on silica gel (EtOAc/CyHex = 50/50, R_f = 0.12) and obtained in 87% yield (32.7 mg, 174 µmol) as a white powder.

m.p. = 158 – 159 °C.

^{1}H NMR (501 MHz, chloroform-*d*, 298 K): δ [ppm] = 10.63 (dd, *J* = 1.5, 0.9 Hz, 1H, Ar-*H*), 9.58 (d, *J* = 1.5 Hz, 1H, Ar-*H*), 8.62 (dd, *J* = 7.4, 1.3 Hz, 1H, Ar-*H*), 8.16 (dt, *J* = 8.1, 1.1 Hz, 1H, Ar-*H*), 7.98 (dd, *J* = 8.1, 7.4 Hz, 1H, Ar-*H*), 4.08 (s, 3H, $COOCH_3$).

^{13}C{^{1}H} NMR (126 MHz, chloroform-*d*, 298 K): δ [ppm] = 165.7 (*C*OOCH$_3$), 151.0 (*C*H-Ar), 149.2 (*C*H-Ar), 136.3 (*C*H-Ar), 131.7 (*C*H-Ar), 131.6 (*C*H-Ar), 126.8 (*C*-Ar), 126.5 (*C*-Ar), 124.9 (*C*-Ar), 53.0 (COO*C*H$_3$).

IR (ATR) [cm^{-1}]: ṽ = 2960, 2929, 2855, 1716, 1612, 1584, 1562, 1448, 1372, 1298, 1255, 1206, 1147, 1045, 956, 930, 865, 776, 757.

HRMS-ESI(+): calc. $C_{10}H_9N_2O_2$ $[M+H]^+$ 189.0659; found 189.0659.

benzo[f]phthalazine (5f)

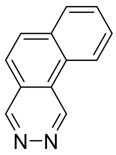

Synthesized according to the **GP6** employing (naphthalene-1,2-diylbis(methylene))bis(tributylphosphonium) bromide **4f** (144 mg, 200 µmol) and potassium *tert*-butoxide (49.4 mg, 440 µmol). The title compound **5f** was purified by column chromatography on silica gel (EtOAc/CyHex = 67/33, R_f = 0.12) and obtained in 86% yield (31.0 mg, 172 µmol) as a slightly yellowish solid.

^{1}H NMR (501 MHz, chloroform-*d*, 298 K): δ [ppm] = 10.33 (s, 1H), 9.61 (s, 1H), 8.80 (d, *J* = 7.9 Hz, 1H), 8.18 (d, *J* = 8.7 Hz, 1H), 8.03 (dd, *J* = 7.7, 1.7 Hz, 1H), 7.89 – 7.77 (m, 3H).

^{13}C{^{1}H} NMR (126 MHz, chloroform-*d*, 298 K): δ [ppm] = 150.9, 146.6, 134.5, 134.1, 129.7, 129.4, 128.8, 127.2, 126.0, 124.7, 122.8, 122.7.

The analytical data are consistent with the literature data.[24]

benzo[g]phthalazine (5g)

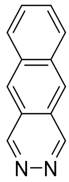

Synthesized according to the **GP6** employing ((naphthalene-2,3-diylbis(methylene))bis(triphenylphosphonium) bromide **4g** (168 mg, 200 µmol) and potassium *tert*-butoxide (49.4 mg, 440 µmol). The title compound **5g** was purified by column chromatography on silica gel (EtOAc, R_f = 0.16) and obtained in 95% yield (34.2 mg, 190 µmol) as a slightly yellowish solid.

^{1}H NMR (501 MHz, chloroform-*d*, 298 K): δ [ppm] = 9.63 (s, 2H), 8.55 (s, 2H), 8.20 – 8.15 (m, 2H), 7.72 (dd, *J* = 6.5 Hz, 3.3 Hz, 2H).

^{13}C{^{1}H} NMR (126 MHz, chloroform-*d*, 298 K): δ [ppm] = 151.9, 135.3, 129.2, 128.6, 126.7, 123.2.

The analytical data are consistent with the literature data.[25]

2,3-diphenylpyridazino[4,5-g]quinoxaline (5h)

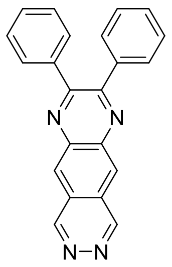

Synthesized according to the **GP6** employing ((2,3-diphenylquinoxaline-6,7-diyl)bis(methylene))bis(tributylphosphonium) bromide **4h** (175 mg, 200 µmol) and potassium *tert*-butoxide (49.4 mg, 440 µmol). The title compound **5h** was purified by column chromatography on silica gel (EtOAc/CyHex = 67/33, R_f = 0.12) and obtained in 86% yield (57.4 mg, 172 µmol) as a slightly yellowish solid.

m.p. = 84 – 86 °C.

^{1}H NMR (501 MHz, chloroform-*d*, 298 K): δ [ppm] = 9.74 (s, 2H, Ar-*H*), 8.85 (s, 2H, Ar-*H*), 7.61 (d, *J* = 7.5 Hz, 4H, Ar-*H*), 7.46 (t, *J* = 7.5 Hz, 2H, Ar-*H*), 7.40 (t, *J* = 7.5 Hz, 4H, Ar-*H*).

^{13}C{^{1}H} NMR (126 MHz, chloroform-*d* , 298 K): δ [ppm] = 156.8 (*C*-Ar), 151.7 (*C*H-Ar), 141.8 (*C*-Ar), 138.3 (*C*-Ar), 130.1 (*C*H-Ar), 130.1 (*C*H-Ar), 128.6 (*C*H-Ar), 128.2 (*C*H-Ar), 125.5 (*C*-Ar).

IR (ATR) [cm^{-1}]: ṽ = 3755, 3654, 3061, 2929, 2858, 1700, 1561, 1543, 1509, 1492, 1447, 1348, 1264, 1162, 1115, 1077, 1056, 1024, 977, 926, 866, 819, 777, 753, 735, 697, 594, 557, 497, 467

HRMS-ESI(+): calc. $C_{22}H_{15}N_4^+$ [M+H]$^+$ 335.1291; found 335.1306

1-(4-(trifluoromethyl)phenyl)phthalazine (5i)

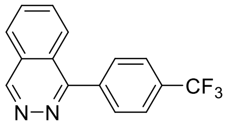

Synthesized according to the **GP6** employing tributyl(2-((tributylphosphonio)(4-(trifluoromethyl)phenyl)methyl)benzyl)phosphonium bromide **4i** (163 mg, 200 µmol) and potassium *tert*-butoxide (49.4 mg, 440 µmol). The title compound **5i** was purified by column chromatography on silica gel (EtOAc/CyHex = 4/3, R_f = 0.15) and obtained in 74% yield (40.6 mg, 148 µmol) as a slightly yellowish solid.

m.p. = 78 – 79 °C

^{1}**H NMR** (501 MHz, chloroform-*d*, 298 K): δ [ppm] = 9.57 (s, 1H, Ar-*H*), 8.07 (d, *J* = 8.1 Hz, 1H, Ar-*H*), 8.01 (d, *J* = 8.1 Hz, 1H, Ar-*H*), 7.96 (t, *J* = 7.4 Hz, 1H, Ar-*H*), 7.94 – 7.87 (m, 3H, Ar-*H*), 7.84 (d, *J* = 8.1 Hz, 2H, Ar-*H*).

^{13}**C{^{1}H} NMR** (126 MHz, chloroform-*d*, 298 K): δ [ppm] = 158.9 (*C*-Ar), 151.1(*C*H-Ar), 139.9(*C*-Ar), 133.1 (*C*H-Ar), 132.7 (*C*H-Ar), 131.6 (q, 32.6 Hz, *C*-Ar), 130.6 (*C*H-Ar), 127.2 (*C*-Ar), 127.0 (*C*H-Ar), 125.73 (*C*H-Ar), 125.68 (q, 3.8 Hz, *C*H-Ar), 125.3 (*C*-Ar), 124.1 (q, *J* = 272.3 Hz, CF_3).

^{19}**F NMR** (377 MHz, chloroform-*d*, 298 K): δ [ppm] = -62.65.

IR (ATR) [cm^{-1}]: ṽ = 1847, 1831, 1794, 1774, 1752, 1736, 1719, 1701, 1686, 1673, 1655, 1638, 1620, 1578, 1561, 1547, 1523, 1509, 1499, 1475, 1459, 1438, 1421, 1398, 1389, 1376, 1364, 1340, 1324, 1177, 1167, 1108, 1066, 1018, 998, 964, 851, 801, 763, 762, 747, 704, 669, 652, 611, 590, 554, 519.

HRMS-ESI(+): calc. $C_{15}H_{10}F_3N_2^+$ $[M+H]^+$ 275.0791; found 275.0793

1-(3-methoxyphenyl)phthalazine (5j)

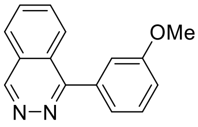

Synthesized according to the **GP6** employing **4j** (155 mg, 200 µmol) and potassium *tert*-butoxide (49.4 mg, 440 µmol). The title compound **5j** was purified by column chromatography on silica gel (EtOAc/CyHex = 4/3, R_f = 0.15) and obtained in 89% yield (42 mg, 178 µmol) as a white powder.

m.p. = 89 – 90 °C.

^{1}**H NMR** (501 MHz, chloroform-*d*, 298 K): δ [ppm] = 9.52 (s, 1H, Ar-*H*), 8.10 (d, *J* = 8.2 Hz, 1H, Ar-*H*), 8.02 (d, *J* = 7.8 Hz, 1H, Ar-*H*), 7.91 (t, *J* = 7.4 Hz, 1H, Ar-*H*), 7.86 (t, *J* = 7.6 Hz, 1H, Ar-*H*), 7.47 (t, *J* = 7.9 Hz, 1H, Ar-*H*), 7.35 – 7.28 (m, 2H, Ar-*H*), 7.09 (dd, *J* = 8.0, 3.1 Hz, 1H, Ar-*H*), 3.88 (s, 3H, O-CH_3).

^{13}**C{^{1}H} NMR** (126 MHz, chloroform-*d*, 298 K): δ [ppm] = 159.9 (*C*-Ar), 150.7(*C*H-Ar), 137.5 (*C*-Ar), 132.7(*C*H-Ar), 132.3(*C*H-Ar), 129.6(*C*H-Ar), 127.2(*C*-Ar), 126.7(*C*H-Ar), 126.4(*C*H-Ar), 125.5(*C*-Ar), 122.6(*C*-Ar), 115.6(*C*H-Ar), 115.3(*C*H-Ar), 55.6 (O-*C*H3).

IR (ATR) [cm^{-1}]: ṽ = 3079, 2991, 2965, 2838, 1602, 1587, 1543, 1484, 1468, 1432, 1356, 1297, 1245, 1166, 1132, 1079, 1038, 1004, 973, 922, 903, 865, 815, 799, 762, 703, 692, 672, 636, 589, 549, 534.

HRMS-ESI(+): calc. $C_{15}H_{13}N_2O^+$ $[M+H]^+$ 237.1022; found 237.1029

pyridazine (5k)

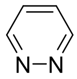

Synthesized according to the **GP6** employing (*Z*)-but-2-ene-1,4-diylbis(triphenylphosphonium) chloride **4k** (130 mg, 200 µmol) and potassium *tert*-butoxide (49.4 mg, 440 µmol). The title compound **5k** was purified by column chromatography on silica gel (EtOAc/pentane = 50/50, R_f = 0.12) and obtained in 92% yield (14.7 mg, 184 µmol) as a colorless oil.

^{1}**H NMR** (501 MHz, chloroform-*d*, 298 K): δ [ppm] = 9.16 (dq, *J* = 5.0, 3.3 Hz, 2H), 7.45 (q, *J* = 3.3 Hz, 2H).

^{13}**C{^{1}H} NMR** (126 MHz, chloroform-*d*, 298 K): δ [ppm] = 151.76, 126.45.

The analytical data are consistent with the literature data.[26]

6,7-dihydrodibenzo[e,k][1,4]dioxa[8,9]diazacyclododecine (5l)+ 6,7,22,23-tetrahydrotetrabenzo[e,k,q,w][1,4,13,16]tetraoxa[8,9,20,21]tetraazacyclotetracosine (5l’, trace)

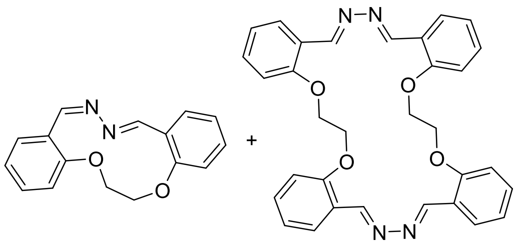

Synthesized according to the **GP6** employing **4l** (((ethane-1,2-diylbis(oxy))bis(2,1-phenylene))bis(methylene))bis(tributylphosphonium) bromide (161 mg, 200 µmol) and potassium *tert*-butoxide (49.4 mg, 440 µmol) in 20 mL THF. The title compound **5l** was purified by column chromatography on silica gel (EtOAc/CyHex = 20/80, R_f = 0.32) and obtained in 32% yield (17.0 mg, 63.8 µmol) as a wax-like solid contaminated with trace amount of dimerized compound **5l’** which can be observed with ^{1}H-NMR and HRMS. The desired compound is very unstable is solution thus decomposition can be observed during the measurement.

m.p. = 64-65 °C (decomp.)

^{1}**H NMR** (501 MHz, chloroform-*d*, 298 K): δ [ppm] = 8.43 (s, 1H, N=C*H*), 7.92 (s, 1H, N=C*H*), 7.83 (s, 4H’, N=C*H*), 7.51 (dd, *J* = 7.6, 1.7 Hz, 1H, Ar-*H*), 7.44 (dd, *J* = 7.6, 1.7 Hz, 4H’, Ar-*H*), 7.34 – 7.31 (m, 1H+4H’, Ar-*H*), 7.31 – 7.27 (m, 1H+4H’, Ar-*H*), 7.14 (td, *J* = 7.6, 1.1 Hz, 1H, Ar-*H*), 7.10 (dd, *J* = 8.2, 1.1 Hz, 1H, Ar-*H*), 7.06 (td, *J* = 7.6, 1.1 Hz, 4H’, Ar-*H*), 6.97 (td, *J* = 7.4, 1.0 Hz, 2H, Ar-*H*), 6.78 (d, *J* = 8.2 Hz, 1H, Ar-*H*), 4.39 – 4.36 (m, 4H’, H_2C-CH_2), 4.32 – 4.28 (m, 2H H_2C-CH_2), 4.23 –

4.20 (m, 4H’, *H*$_2$C-C*H*$_2$), 4.20 – 4.17 (m, 2H, *H*$_2$C-C*H*$_2$). Signal of monomer and dimer are both assigned.

^{13}C{^{1}H} NMR (126 MHz, chloroform-*d*, 298 K): δ [ppm] = 152.9 (N=*C*H), 150.4 (N=*C*H), 131.2 (*C*H-Ar), 131.1 (*C*H-Ar), 130.9 (*C*H-Ar), 129.7 (*C*-Ar), 129.6 (*C*-Ar), 129.1 (*C*H-Ar), 128.6 (*C*-Ar), 125.0 (*C*H-Ar), 123.5 (*C*-Ar), 122.0 (*C*H-Ar), 120.7 (*C*H-Ar), 112.3 (*C*H-Ar), 72.6 (H$_2$*C*-CH$_2$), 65.9 (H$_2$C-*C*H$_2$). Only signal of monomer are assigned.

IR (ATR) [cm^{-1}]: ṽ = 2929, 2870, 1687, 1620, 1602, 1580, 1490, 1448, 1284, 1242, 1104, 1045, 914, 799, 754.

HRMS-ESI(+): calc. $C_{16}H_{15}N_2O_2^+$ [M+H]$^+$ 267.1128; found 267.1127 (major); calc. $C_{32}H_{29}N_4O_4^+$ [M+H]$^+$ 533.2183; found 533.2188 (minor).

(5*E*,7*E*,22*E*,24*E*)-15,16,32,33-tetrahydro-14H,31H-tetrabenzo[f,l,s,y][1,5,14,18]tetraoxa[9,10,22,23]tetraazacyclohexacosine (5m’)

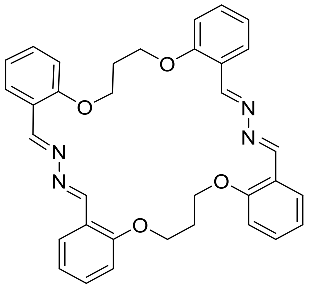

Synthesized according to the **GP6** employing **4m** (((ethane-1,2-diylbis(oxy))bis(2,1-phenylene))bis(methylene))bis(tributylphosphonium) bromide (163 mg, 200 µmol) and potassium *tert*-butoxide (49.4 mg, 440 µmol) in 20 mL THF. The diazo transfer was carried out at ambient temperature. The title compound **5m’** was purified by column chromatography on silica gel (EtOAc/CyHex = 20/80, R$_f$ = 0.32) and obtained in 48% yield (26.9 mg, 48.0 µmol) as a slightly yellowish solid.

m.p. = 168-169 °C (decomp.).

^{1}H NMR (600 MHz, methylene chloride-*d*$_2$, 298 K): δ [ppm] = 8.61 (s, 4H, N=C*H*), 7.79 (dd, *J* = 7.4, 1.8 Hz, 4H, Ar-*H*) 7.44 (ddd, *J* = 8.3, 7.4, 1.8 Hz, 4H, Ar-*H*), 7.04 (dd, *J* = 8.3, 1.0 Hz, 4H, Ar-*H*), 7.00 (t, *J* = 7.4 Hz, 4H, Ar-*H*), 4.44 – 4.27 (m, 8H, *H*$_2$C-H$_2$C-C*H*$_2$), 2.35 (p, *J* = 4.4 Hz, 4H, H$_2$C-*H*$_2$C-CH$_2$).

^{13}C{^{1}H} NMR (151 MHz, methylene chloride-*d*$_2$, 298 K): δ [ppm] = 158.7 (*C*-Ar), 155.7 (N=*C*H), 132.4 (*C*H-Ar), 128.1 (*C*H-Ar), 124.1 (*C*-Ar), 121.3 (*C*H-Ar), 113.6 (*C*H-Ar), 68.3 (H$_2$*C*-H$_2$C-*C*H$_2$), 29.2(H$_2$C-H$_2$*C*-CH$_2$).

IR (ATR) [cm^{-1}]: $\tilde{v}$ = 2958, 2930, 2882, 1618, 1601, 1488, 1456, 1397, 1325, 1297, 1247, 1162, 1110, 1058, 964, 862, 753, 674.

HRMS-ESI(+): calc. $C_{34}H_{33}N_4O_4^+$ $[M+H]^+$ 561.2496; found 561.2505.

General procedure for triazole synthesis (GP7)

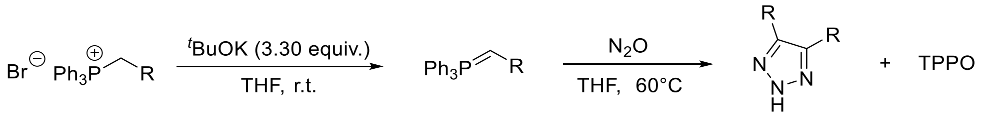

To a dried pressure Schlenk-flask equipped with a magnetic stirring bar, a suspension of phosphonium salt (1.00 equiv.) was prepared in anhydrous THF (5 mL) under argon atmosphere. To this suspension mixture, potassium *tert*-butoxide (3.30 equiv.) was added under argon counter flow. The solution was stirred at ambient temperature for 30 min. The solution was cooled to –78 °C and the inert gas was exchanged for 1 atmosphere of N_2O (Note: N_2O was directly used from the gas bottle; quality 5.0 obtained from Messer) by evacuating the pressure Schlenk-flask until gas evolution from the solution at –78 °C has ceased then repressurizing it with N_2O (1 bar). After repressurizing with N_2O, the flask was closed and stirred at 60 °C in the dark (Caution: the actual pressure in the flask at room temperature is higher; use pressure glassware and explosion shield). The crude mixture was directly adsorbed onto silica gel and purified by flash chromatography to give the corresponding heterocyclic product **12**.

4,5-diphenyl-2*H*-1,2,3-triazole (12b)

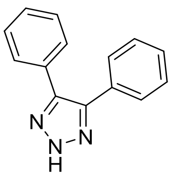

Synthesized according to the **GP7** employing benzyltriphenylphosphonium bromide **7b** (173 mg, 400 µmol) and potassium *tert*-butoxide (148 mg, 1.32 mmol). The title compound **12b** was purified by column chromatography on silica gel (EtOAc/CyHex = 20/80, R_f = 0.32) and obtained in 78% yield (22.8 mg, 103 µmol) as a slightly yellowish solid.

^{1}H NMR (501 MHz, chloroform-*d*, 298 K): δ [ppm] = 7.61 – 7.51 (m, 4H), 7.38 – 7.34 (m, 6H).

^{13}C{^{1}H} NMR (126 MHz, chloroform-*d*, 298 K): δ [ppm] = 142.8 (br), 130.3, 128.8, 128.7, 128.4.

The analytical data are consistent with the literature data.[27]

4,5-bis(4-(*tert*-butyl)phenyl)-2*H*-1,2,3-triazole (12c)

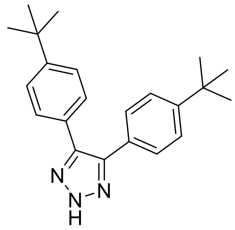

Synthesized according to the **GP7** employing (4-(*tert*-butyl)benzyl)triphenylphosphonium bromide **7c** (196 mg, 400 µmol) and potassium tert-butoxide (148 mg, 1.32 mmol). The title compound **12c** was purified by column chromatography on silica gel (EtOAc/CyHex = 20/80, $R_f = 0.34$) and obtained in 86% yield (37.8 mg, 113 µmol) as a slightly yellowish solid.

m.p. = 219 – 220 °C

^{1}H NMR (501 MHz, chloroform-*d*, 298 K): δ [ppm] = 12.74 (s, br, 1H, NH), 7.54 (d, *J* = 8.4 Hz, 4H, Ar-*H*), 7.40 (d, *J* = 8.5 Hz, 4H, Ar-*H*), 1.35 (s, 18H, *t*-Bu).

^{13}C{^{1}H} NMR (126 MHz, chloroform-*d*, 298 K): δ [ppm] = 151.7 (*C*-Ar), 142.4 (br, *C*-Ar), 128.0 (*C*H-Ar), 127.5 (*C*H-Ar), 125.7 (*C*-Ar), 34.9 (*C*(CH_3)$_3$), 31.4 (C(*C*H_3)$_3$).

IR (ATR) [cm^{-1}]: ṽ = 2957, 2917, 2867, 1516, 1466, 1400, 1363, 1269, 1202, 1114, 997, 909, 836, 731, 645, 563.

HRMS-APCI(+): calc. $C_{22}H_{28}N_3^+$ $[M+H]^+$ 334.2278; found 334.2278.

4,5-di((*E*)-styryl)-2*H*-1,2,3-triazole (12d)

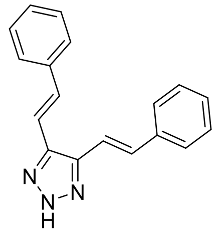

Synthesized according to the **GP7** employing cinnamyltriphenylphosphonium bromide **7d** (184 mg, 400 µmol) and potassium *tert*-butoxide (148 mg, 1.32 mmol). The title compound **12d** was purified by column chromatography on silica gel (EtOAc/CyHex = 20/80, $R_f = 0.22$) and obtained in 54% yield (19.6 mg, 71.7 µmol) as a slightly yellowish oil (slow decomposition can be observed at room temperature).

^{1}H NMR (501 MHz, chloroform-*d*, 298 K): δ [ppm] = 7.77 – 7.73 (m, 3H, Ar-*H*), 7.63 (br d, *J* = 2.3 Hz, 2H, C*H*=CH-Ph), 7.46 – 7.40 (m, 4H, Ar-*H*), 7.36 – 7.32 (m, 3H, Ar-*H*), 6.63 (br d, *J* = 2.2 Hz, 2H, CH=C*H*-Ph).

^{13}C{^{1}H} NMR (126 MHz, chloroform-*d*, 298 K): δ [ppm] = 133.3 (*C*H=CH-Ph), 132.2 (*C*-Ar), 129.0 (*C*H-Ar), 128.3 (*C*H-Ar), 126.8(*C*-Ar), 125.8 (*C*H-Ar), 102.9 (CH=*C*H-Ph).

IR (ATR) [cm^{-1}]: ṽ = 3264, 2924, 1688, 1454, 1259, 1155, 1098, 1032, 912, 824,765, 726, 637.

HRMS-APCI(+): calc. $C_{18}H_{16}N_3^+$ [M+H]$^+$ 274.1339; found 274.1339.

General procedure for azine synthesis (GP8)

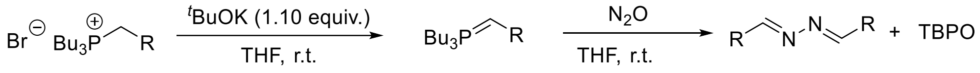

To a dried pressure Schlenk-flask equipped with a magnetic stirring bar, a suspension of phosphonium salt (1.00 equiv.) was prepared in anhydrous THF (5 mL) under argon atmosphere. To this suspension, potassium *tert*-butoxide (1.10 equiv.) was added under argon counterflow. The solution was stirred at ambient temperature for 30 min. The solution was cooled to –78 °C and the inert gas was exchanged for 1 atmosphere of N_2O (Note: N_2O was directly used from the gas bottle; quality 5.0 obtained from Messer) by evacuating the pressure Schlenk-flask until gas evolution from the solution at –78 °C has ceased then repressurizing it with N_2O (1 bar). After repressurizing with N_2O at –78 °C, the flask was closed and slowly warm up to room temperature in the dark and the reaction was stirred at this temperature for another 24 hours (Caution: the actual pressure in the flask at room temperature is higher; use pressure glassware and explosion shield). After liberate the excess of nitrous oxide, the resulting mixture was filtered and directly adsorbed onto silica gel for further purification by flash chromatography to give the corresponding azines.

1,2-di(benzylidene)hydrazine (13a)

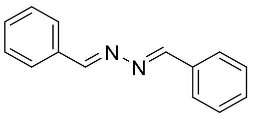

Synthesized according to the **GP8** employing benzyltributylphosphonium bromide **9a** (74.7 mg, 200 µmol) and potassium *tert*-butoxide (24.7 mg, 220 µmol). The title compound **13a** was purified by column chromatography on silica gel (EtOAc/CyHex = 5/95, R_f = 0.56) and obtained in 86% yield (17.9 mg, 86.0 µmol) as a yellowish solid.

^{1}H NMR (501 MHz, chloroform-*d*, 298 K): δ [ppm] = 8.68 (s, 2H), 7.98 – 7.78 (m, 4H), 7.51 – 7.43 (m, 6H).

^{13}C{^{1}H} NMR (126 MHz, chloroform-*d*, 298 K): δ [ppm] = 162.2, 134.2, 131.3, 128.9, 128.7.

The analytical data are consistent with the literature data.[28]

1,2-bis(4-(tert-butyl)benzylidene)hydrazine (13b)

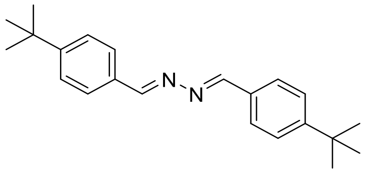

Synthesized according to the **GP8** employing tributyl(4-(*tert*-butyl)benzyl)phosphonium bromide **9b** (85.9 mg, 200 µmol) and potassium *tert*-butoxide (24.7 mg, 220 µmol). The title compound **13b** was purified by column chromatography on silica gel (EtOAc/CyHex = 5/95, R_f = 0.57) and obtained in 92% yield (29.5 mg, 92.1 µmol) as a yellowish solid.

m.p. = 204 – 206 °C.

^{1}H NMR (501 MHz, chloroform-*d*, 298 K): δ [ppm] = 8.65 (s, 2H, N=C*H*), 7.78 (d, *J* = 8.4 Hz, 4H, Ar-*H*), 7.48 (d, *J* = 8.4 Hz, 4H, Ar-*H*), 1.36 (s, 18H, *t*-Bu).

^{13}C{^{1}H} NMR (126 MHz, chloroform-*d*, 298 K): δ [ppm] = 161.8(N=*C*H), 154.8 (*C*-Ar), 131.6 (*C*-Ar), 128.5 (*C*H-Ar), 125.9 (*C*H-Ar), 35.2 (*C*(CH_3)$_3$), 31.3 (C(*C*H_3)$_3$).

IR (ATR) [cm^{-1}]: ṽ = 2951, 2903, 2868, 1627, 1563, 1506, 1463, 1415, 1365, 1294, 1265, 1222, 1205, 1186, 1120, 1105, 1019, 970, 947, 868, 833, 721.

HRMS-ESI(+): calc. $C_{22}H_{29}N_2^+$ $[M+H]^+$ 321.2325; found 321.2323.

1,2-bis(4-methoxybenzylidene)hydrazine (13c)

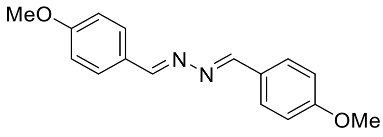

Synthesized according to the **GP8** employing tributyl(4-methoxybenzyl)phosphonium chloride **9c** (71.8 mg, 200 µmol) and potassium *tert*-butoxide (24.7 mg, 220 µmol). The title compound **13c** was purified by column chromatography on silica gel (EtOAc/CyHex = 5/95, R_f = 0.51) and obtained in 96% yield (25.8 mg, 96.2 µmol) as a yellowish solid.

^{1}H NMR (501 MHz, chloroform-*d*, 298 K): δ [ppm] = 8.61 (s, 2H), 7.78 (d, *J* = 8.8 Hz, 4H), 6.96 (d, *J* = 8.8 Hz, 4H), 3.86 (s, 6H).

^{13}C{^{1}H} NMR (126 MHz, chloroform-*d*, 298 K): δ [ppm] = 162.1, 161.2, 130.3, 127.2, 114.4, 55.5.

The analytical data are consistent with the literature data.[24]

1,2-bis((*E*)-3-bromobenzylidene)hydrazine (13d)

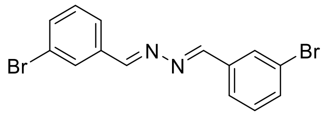

Synthesized according to the **GP8** employing (3-bromobenzyl)tributylphosphonium bromide **9d** (90.5 mg, 200 µmol) and potassium *tert*-butoxide (24.7 mg, 220 µmol). The title compound **13d** was purified by column chromatography on silica gel (EtOAc/CyHex = 5/95, R_f = 0.54) and obtained in 54% yield (19.8 mg, 54.1 µmol) as a yellowish solid.

^{1}H NMR (501 MHz, chloroform-*d*, 298 K): δ [ppm] = 8.57 (s, 2H), 8.04 (t, *J* = 1.8 Hz, 2H), 7.73 (dt, *J* = 7.7, 1.4 Hz, 2H), 7.60 (ddd, *J* = 8.0, 2.1, 1.1 Hz, 2H), 7.33 (t, *J* = 7.8 Hz, 2H).

^{13}C{^{1}H} NMR (126 MHz, chloroform-*d*, 298 K): δ [ppm] = 161.2, 136.1, 134.4, 131.2, 130.5, 127.6, 123.2.

The analytical data are consistent with the literature data.[29]

1,2-bis(2-bromobenzylidene)hydrazine (13e)

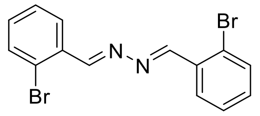

Synthesized according to the **GP8** employing (2-bromobenzyl)tributylphosphonium bromide **9e** (90.5 mg, 200 µmol) and potassium *tert*-butoxide (24.7 mg, 220 µmol). The title compound **13e** was purified by column chromatography on silica gel (EtOAc/CyHex = 5/95, R_f = 0.56) and obtained in 65% yield (23.7 mg, 64.7 µmol) as a yellowish solid.

^{1}H NMR (501 MHz, chloroform-*d*, 298 K): δ [ppm] = 9.03 (s, 2H), 8.22 (dd, *J* = 7.8, 1.8 Hz, 2H), 7.63 (d, *J* = 7.8 Hz, 2H), 7.40 (t, *J* = 7.6 Hz, 2H), 7.32 (td, *J* = 7.6, 1.8 Hz, 2H).

^{13}C{^{1}H} NMR (126 MHz, chloroform-*d* , 298 K): δ [ppm] = 161.5, 133.5, 133.1, 132.6, 128.9, 127.8, 126.0.

The analytical data are consistent with the literature data.[29]

1,2-bis(3-cyanobenzylidene)hydrazine (13f) +(*E*)-3,3'-(ethene-1,2-diyl)dibenzonitrile (14f)

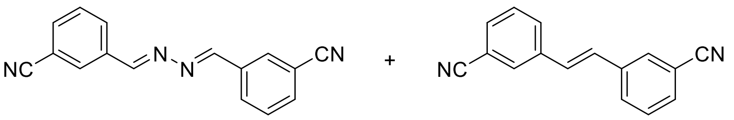

Synthesized according to the **GP8** employing tributyl(3-cyanobenzyl)phosphonium bromide **9f** (79.7 mg, 200 µmol) and potassium *tert*-butoxide (24.7 mg, 220 µmol). The title compound was purified by column chromatography on silica gel (EtOAc/CyHex = 5/95, R_f = 0.48) and obtained a mixture of compound **13f** (21%, 5.4 mg, 21.0 µmol) and **14f** (75%, 19.4 mg, 75.1 µmol) in total 21.9 mg as a yellowish solid. The yield was calculated by ^{1}H NMR integration.

(*E*)-3,3'-(ethene-1,2-diyl)dibenzonitrile (14f)

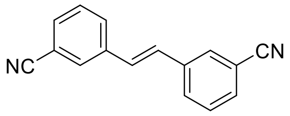

m.p. = 196 – 197 °C

^{1}H NMR (501 MHz, chloroform-*d*, 298 K): δ [ppm] = 7.79 (t, *J* = 1.7 Hz, 2H, Ar- *H*), 7.73 (dt, *J* = 7.8, 1.5 Hz, 2H, Ar-*H*), 7.58 (dt, *J* = 7.7, 1.4 Hz, 2H, Ar-*H*), 7.50 (t, *J* = 7.8 Hz, 2H, Ar-*H*), 7.12 (s, 2H, *H*C=C*H*).

^{13}C{^{1}H} NMR (126 MHz, chloroform-*d*, 298 K): δ [ppm] = 137.8 (*C*-Ar), 131.6 (*C*H-Ar), 130.9 (*C*H-Ar), 130.2 (*C*H-Ar), 129.8 (*C*H-Ar), 129.0 (H*C*=*C*H), 118.7 (*C*-Ar), 113.3 (CN).

IR (ATR) [cm^{-1}]: ṽ = 2227, 1598, 1581, 1488, 1432, 1328, 1262, 1095, 966, 891, 795, 683, 617, 545, 484, 436.

HRMS-APCI(+): calc. $C_{16}H_{10}N_2Na^+$ $[M+Na]^+$ 253.0736.; found 253.0737.

3,3'-(hydrazine-1,2-diylidenebis(methaneylylidene))dibenzonitrile (13f)

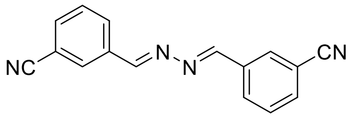

m.p. = 250 – 251 °C

^{1}H NMR (501 MHz, chloroform-*d*, 298 K): δ [ppm] = 8.64 (s, 2H, N=C*H*), 8.18 (t, *J* = 1.7 Hz, 2H, Ar-*H*), 8.06 (dt, *J* = 7.8, 1.5 Hz, 2H, Ar-*H*), 7.76 (dt, *J* = 7.6, 1.4 Hz, 2H, Ar-*H*), 7.60 (t, *J* = 7.8 Hz, 2H, Ar-*H*).

^{13}C{^{1}H} NMR (126 MHz, chloroform-*d* , 298 K): δ [ppm] = 160.7 (N=*C*H), 135.2 (*C*-Ar), 134.6 (*C*H-Ar), 132.8 (*C*H-Ar), 132.1 (*C*H-Ar), 129.9 (*C*H-Ar), 118.2 (*C*-Ar), 113.5 (CN).

IR (ATR) [cm^{-1}]: ṽ = 3062, 2923, 2229, 1628, 1581, 1486, 1419, 1312, 1290, 1241, 1177, 1159, 1093, 1002, 957, 930, 898, 798, 680, 596, 557, 485, 461, 430.

HRMS-APCI(+): calc. $C_{16}H_{11}N_4^+$ $[M+H]^+$ 259.0979.; found 259.0983.

1,2-bis(4-(trifluoromethyl)benzylidene)hydrazine (13g) (trace) +
(*E*)-1,2-bis(4-(trifluoromethyl)phenyl)ethene (14g)

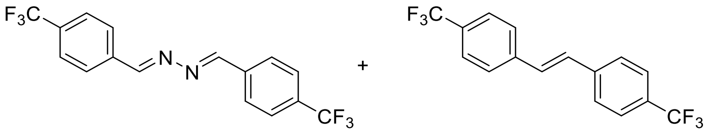

Synthesized according to the **GP8** employing tributyl(4-(trifluoromethyl)benzyl)phosphonium bromide **9g** (88.3 mg, 200 µmol) and potassium *tert*-butoxide (24.7 mg, 220 µmol). The title compound was purified by column chromatography on silica gel (EtOAc/CyHex = 5/95, R_f = 0.51) and obtained (*E*)-1,2-bis(4-(trifluoromethyl)phenyl)ethene **14g** in 88% yield (27.7 mg, 87.6 µmol) as a white powder with trace impurity of 1,2-bis(4-(trifluoromethyl)benzylidene)hydrazine **13g**.

^{1}H NMR (501 MHz, chloroform-*d*, 298 K): δ [ppm] = 7.63 (m, 8H), 7.20 (s, 2H).

^{13}C{^{1}H} NMR (126 MHz, chloroform-*d* , 298 K): δ [ppm] = 140.4 (d, *J* = 1.6 Hz), 130.3 (q, *J* = 32.5 Hz), 130.0, 127.2, 126.1 (q, *J* = 3.8 Hz), 124.5 (q, *J* = 271.8 Hz).

^{19}F NMR (565 MHz, chloroform-*d*, 298 K): δ [ppm] = –62.6.

The analytical data are consistent with the literature data.[30]

Procedure for intermolecular trapping employing norbornene

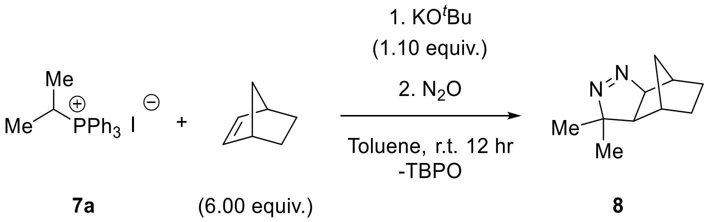

To a dried pressure Schlenk-flask equipped with a magnetic stirring bar, a suspension of commercially available phosphonium salt **7a** (1.00 equiv., 0.50 mmol, 216 mg) was prepared in anhydrous THF (5 mL) under argon atmosphere. To this suspension, potassium *tert*-butoxide (1.10 equiv. 0.55 mmol, 61.7 mg) was added under argon counterflow. The solution was stirred at ambient temperature for 30 min. To the solution, norbornene (6.00 equiv., 3.00 mmol, 283 mg) was added under argon counterflow. The reaction mixture was then cooled to –78 °C and the inert gas was exchanged for 1 atmosphere of N_2O (Note: N_2O was directly used from the gas bottle; quality 5.0 obtained from Messer) by evacuating the pressure Schlenk-flask until gas evolution from the solution at –78 °C has ceased then repressurizing it with N_2O (1 bar). After gas exchange, the flask was sealed and allowed to warm slowly

to 60 °C, in the absence of light. The reaction was stirred at this temperature for 12 h (Caution: the actual pressure in the flask at room temperature is higher; use pressure glassware and explosion shield). All the volatiles were removed under reduced pressure with careful temperature control at 20 °C. The crude product was absorbed on silica gel and purified by flash column chromatography (EtOAc/CyHex = 10/90, R_f = 0.55) to yield 3,3-dimethyl-3a,4,5,6,7,7a-hexahydro-3*H*-4,7-methanoindazole (**8**) as a colorless oil in 76% (62.7 mg, 382 µmol).

^{1}H NMR (501 MHz, chloroform-*d*, 298 K): δ [ppm] = 4.64 (d, *J* = 6.7 Hz, 1H), 2.81 (d, *J* = 4.3 Hz, 1H), 2.03 (d, *J* = 4.1 Hz, 1H), 1.58 (tq, *J* = 12.1, 4.1 Hz, 1H), 1.44 (dt, *J* = 12.1, 4.6 Hz, 1H), 1.40 (s, 3H), 1.36 (dd, *J* = 6.7, 1.8 Hz, 1H), 1.34 – 1.26 (m, 1H), 1.12 (s, 3H), 1.10 – 1.03 (m, 1H), 0.95 (dt, *J* = 10.6, 1.5 Hz, 1H), 0.56 (dt, *J* = 10.6, 2.1 Hz, 1H).

^{13}C{^{1}H} NMR (126 MHz, chloroform-*d*, 298 K): δ [ppm] = 97.4, 89.9, 48.7, 37.8, 37.2, 32.8, 28.8, 28.7, 25.5, 20.3.

The analytical data are consistent with the literature data.[31]

Procedure for intermolecular trapping employing Cyclooctyne

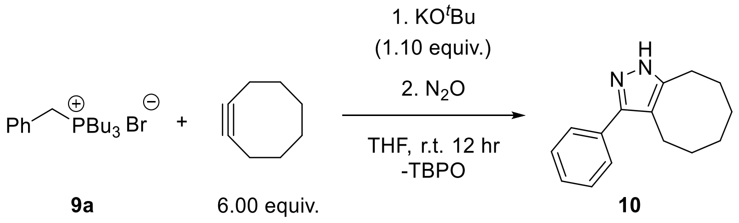

To a dried pressure Schlenk-flask equipped with a magnetic stirring bar, a suspension of phosphonium salt **9a** (1.00 equiv. 0.40 mmol, 149 mg) was prepared in anhydrous THF (5 mL) under argon atmosphere. To this suspension, potassium *tert*-butoxide (1.10 equiv. 0.44 mmol, 49.4 mg) was added under argon counterflow. The solution was stirred at ambient temperature for 30 min. To the solution, cyclooctyne (6.00 equiv., 1.20 mmol, 300 µL) was added under argon counterflow. The reaction mixture was then cooled to –78 °C and the inert gas was exchanged for 1 atmosphere of N_2O (Note: N_2O was directly used from the gas bottle; quality 5.0 obtained from Messer) by evacuating the pressure Schlenk-flask until gas evolution from the solution at –78 °C has ceased then repressurizing it with N_2O (1 bar). After gas exchange, the flask was sealed and allowed to warm slowly to the room temperature, in the absence of light. The reaction was stirred at this temperature for 12 hours (Caution: the actual pressure in the flask at room temperature is higher; use pressure glassware and explosion shield). All the volatiles were removed under reduced pressure. The crude product was absorbed on silica gel and directly purified by flash column chromatography (EtOAc/CyHex = 20/80, R_f = 0.34) to yield

3-phenyl-4,5,6,7,8,9-hexahydro-1*H*-cycloocta[c]pyrazole (**10**) as a colorless oil in 45% (20.5 mg, 90.6 µmol).

^{1}H NMR (501 MHz, chloroform-*d*, 298 K): δ [ppm] = 7.56 – 7.51 (m, 2H, Ar-*H*), 7.45 – 7.39 (m, 2H, Ar-*H*), 7.38 – 7.31 (m, 1H, Ar-*H*), 2.79 – 2.74 (m, 2H, C*H*$_2$), 2.72 – 2.67 (m, 2H, C*H*$_2$), 1.77 – 1.69 (m, 4H, C*H*$_2$), 1.57 – 1.48 (m, 4H, C*H*$_2$).

^{13}C{^{1}H} NMR (126 MHz, chloroform-*d*, 298 K): δ [ppm] = 148.6 (found in HMBC, *C*-Ar), 145.1 (found in HMBC, *C*-Ar), 132.6 (*C*-Ar), 128.8 (*C*H-Ar), 127.8 (*C*H-Ar), 127.7 (*C*H-Ar), 115.5 (*C*-Ar), 30.5 (*C*H$_2$), 29.8 (*C*H$_2$), 26.0 (*C*H$_2$), 25.8 (*C*H$_2$), 25.2 (*C*H$_2$), 21.9 (*C*H$_2$).

IR (ATR) [cm^{-1}]: ṽ = 3190, 3128, 3063, 2929, 2854, 1573, 1509, 1458, 1360, 1256, 1161, 1138, 1073, 1032, 982, 947, 912, 770, 732, 698, 548.

HRMS-ESI(+): calc. $C_{15}H_{19}N_2^+$ [M+H]$^+$ 227.1543; found 227.1542.

Procedure for one-pot transformation from phosphonium ylide to 1,3,4-oxadiazole

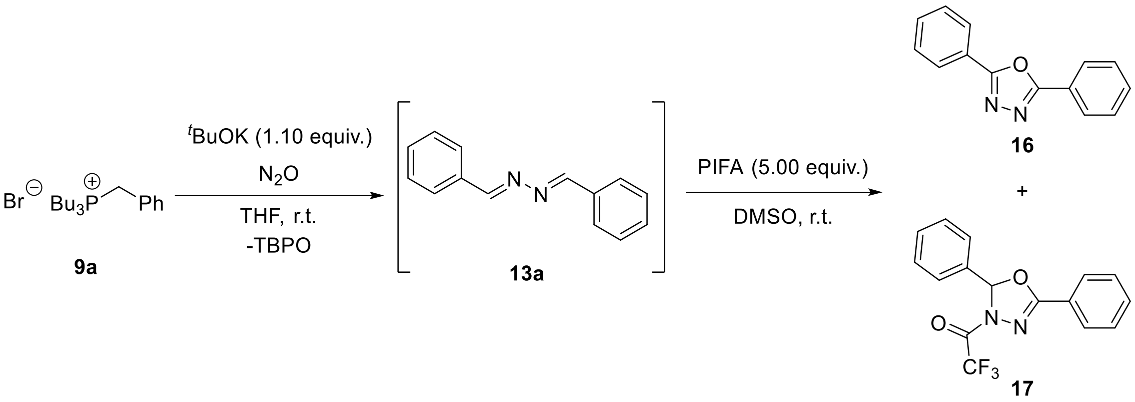

To a dried pressure Schlenk-flask equipped with a magnetic stirring bar, a suspension of phosphonium salt **9a** (1.00 equiv. 0.20 mmol, 74.7 mg) was prepared in anhydrous THF (5 mL) under argon atmosphere. To this suspension, potassium *tert*-butoxide (1.10 equiv. 0.22 mmol, 24.7 mg) was added under argon counterflow. The solution was stirred at ambient temperature for 30 min. The reaction mixture was then cooled to –78 °C and the inert gas was exchanged for 1 atmosphere of N_2O (Note: N_2O was directly used from the gas bottle; quality 5.0 obtained from Messer) by evacuating the pressure Schlenk-flask until gas evolution from the solution at –78 °C has ceased then repressurizing it with N_2O (1 bar). After gas exchange, the flask was sealed and allowed to warm slowly to the room temperature, in the absence of light. The reaction was stirred at this temperature for 12 hours (Caution: the actual pressure in the flask at room temperature is higher; use pressure glassware and explosion shield). All the volatiles were removed under reduced pressure. To the crude mixture dimethyl sulfoxide (2 mL) and (Bis(trifluoroacetoxy)iodo)benzene (5.00 equiv. refer to resulting aldazine, 0.50 mmol, 219 mg) were

added under an argon atmosphere. The reaction was stirred at this temperature for another 48 hours to afford a full consumption of aldazine was observed by TLC analysis. Aqueous workup was performed and the reaction mixture was extracted with dichloromethane (3 × 10 mL). Combined organic phases was collected and concentrated under reduced pressure. The crude product was purified by flash column chromatography (EtOAc/CyHex = 10/90 to 20/80) to yield compound **16** in 42% (9.4 mg, 42.3 µmol) and compound **17** in 37% (9.9 mg, 37.2 µmol).

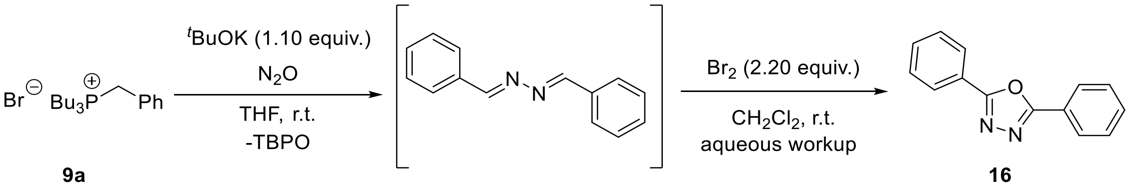

9a **16**

To a dried pressure Schlenk-flask equipped with a magnetic stirring bar, a suspension of phosphonium salt (1.00 equiv. 0.20 mmol, 74.7 mg) was prepared in anhydrous THF (5 mL) under argon atmosphere. To this suspension, potassium *tert*-butoxide (1.10 equiv. 0.22 mmol, 24.7 mg) was added under argon counterflow. The solution was stirred at ambient temperature for 30 min. The reaction mixture was then cooled to –78 °C and the inert gas was exchanged for 1 atmosphere of N_2O (Note: N_2O was directly used from the gas bottle; quality 5.0 obtained from Messer) by evacuating the pressure Schlenk-flask until gas evolution from the solution at –78 °C has ceased then repressurizing it with N_2O (1 bar). After gas exchange, the flask was sealed and allowed to warm slowly to the room temperature, in the absence of light. The reaction was stirred at this temperature for another 12 hours (Caution: the actual pressure in the flask at room temperature is higher; use pressure glassware and explosion shield). All the volatiles were removed under reduced pressure. To the crude mixture, a solution of elemental bromine (2.20 equiv. 0.22 mmol, 11.3 µL) in dichloromethane (2 mL) was added dropwise at 0 °C under an argon atmosphere. The reaction was allowed to warm to room temperature and stirred for another 24 hours at this temperature. An aqueous workup was performed when a full consumption of aldazine was observed by TLC analysis. Upon cooling at 0 °C saturated sodium thiosulfate solution in water was added to the reaction mixture slowly until the yellowish color vanished. The mixture was extracted with dichloromethane (3 × 10 mL) and the organic layer was collected. After removal of volatiles under reduced pressure, the crude product was further purified by flash column chromatography (EtOAc/CyHex = 10/90 to 20/80) to yield **16** in 78% (17.4 mg, 78.3 µmol).

2,5-diphenyl-1,3,4-oxadiazole (16)

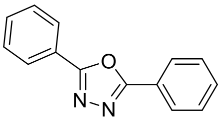

R_f = 0.52 (EtOAc/CyHex = 20/80).

^{1}H NMR (501 MHz, chloroform-*d*, 298 K): δ [ppm] = 8.18 – 8.13 (m, 4H), 7.59 – 7.52 (m, 6H).

^{13}C{^{1}H} NMR (126 MHz, chloroform-*d*, 298 K): δ [ppm] = 164.8, 131.9, 129.3, 127.1, 124.1.

The analytical data are consistent with the literature data.[32]

1-(2,5-diphenyl-1,3,4-oxadiazol-3(2*H*)-yl)-2,2,2-trifluoroethan-1-one (17)

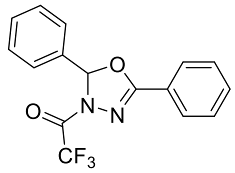

R$_f$ = 0.36 (EtOAc/CyHex = 20/80).

^{1}H NMR (600 MHz, chloroform-*d*, 300 K): δ [ppm] = 7.96 - 7.93 (m, 2H, Ar-*H*), 7.59 – 7.55 (m, 1H, Ar-*H*), 7.52 – 7.42 (m, 7H, Ar-*H*), 7.10 (s, 1H, OC*H*N).

^{13}C{^{1}H} NMR (126 MHz, chloroform-*d*, 298 K): δ [ppm] = 158.4 (N=*C*(O)Ph), 152.6 (q, *J* = 39.6 Hz, *C*OCF$_3$), 134.3 (*C*-Ar), 132.7 (*C*H-Ar), 130.9 (*C*H-Ar), 129.2 (*C*H-Ar), 129.0 (*C*H-Ar), 127.7 (*C*H-Ar), 126.8 (*C*H-Ar), 123.6 (*C*-Ar), 115.9 (q, *J* = 286.6 Hz, CO*C*F$_3$), 93.2 (O*C*HN).

^{19}F NMR (565 MHz, chloroform-*d*, 298 K): δ [ppm] = –72.5.

IR [cm^{-1}]: ṽ = 1695, 1634, 1581, 1471, 1453, 1343, 1286, 1204, 1156, 1067, 1027, 972, 845, 762, 744, 734, 689, 671, 646, 626.

HRMS-APCI(+): calc. $C_{14}H_{12}N_2NaO^+$ $[M\text{-}COCF_3\text{+}Na]^+$ 247.0842; found 247.0836.

Procedure for one-pot transformation from phosphonium ylide to afford 3,5-diphenyl-4*H*-1,2,4-triazole

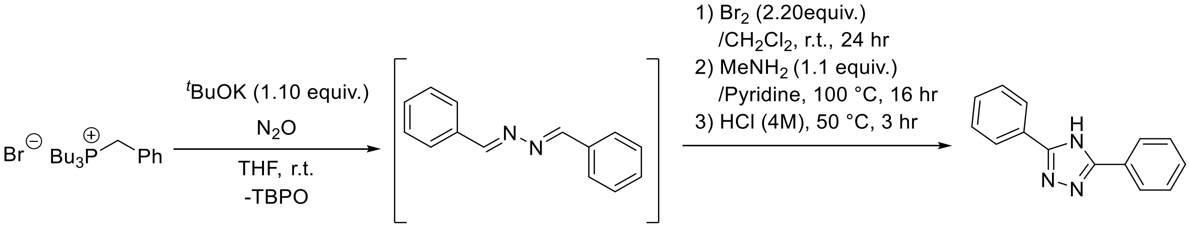

9a **18**

To a dried pressure Schlenk-flask equipped with a magnetic stirring bar, a suspension of phosphonium salt **9a** (1.00 equiv. 0.20 mmol, 74.7 mg) was prepared in anhydrous THF (5 mL) under argon atmosphere. To this suspension, potassium *tert*-butoxide (1.10 equiv. 0.22 mmol, 24.7 mg) was added under argon counterflow. The solution was stirred at ambient temperature for 30 min. The reaction

mixture was then cooled to –78 °C and the inert gas was exchanged for 1 atmosphere of N_2O (Note: N_2O was directly used from the gas bottle; quality 5.0 obtained from Messer) by evacuating the pressure Schlenk-flask until gas evolution from the solution at –78 °C has ceased then repressurizing it with N_2O (1 bar). After gas exchange, the flask was sealed and allowed to warm slowly to the room temperature, in the absence of light. The reaction was stirred at this temperature for 12 hours (Caution: the actual pressure in the flask at room temperature is higher; use pressure glassware and explosion shield). All the volatiles were removed under reduced pressure. To the crude mixture, a solution of elemental bromine (2.20 equiv. 0.22 mmol, 11.3 µL) in dichloromethane (2 mL) was added dropwise at 0 °C under an argon atmosphere. The reaction was allowed to warm to room temperature and stirred for another 24 hours at this temperature. All the volatiles were removed under reduced pressure. To this crude mixture, anhydrous pyridine (2 mL) and methylamine (0.55 equiv. 2.0 M in THF, 0.11 mmol, 55 µL) was added sequentially. The reaction was sealed and heated to 100 °C for 16 hours. The resulting crude mixture was cooled to room temperature. Acidic work-up was carried out with hydrochloric acid (4N) at 50 °C for additional 3 hours to fulfill demethylation. The resulting crude mixture was extracted by dichloromethane (3 × 10 mL) and the combined organic phases were concentrated under reduced pressure. The residue was further purified by flash column chromatography on silica gel to afford 3,5-diphenyl-4*H*-1,2,4-triazole **18** in 42% yield (9.3 mg, 42.0 µmol).

^{1}H NMR (500 MHz, chloroform-*d*, 300 K): δ [ppm] = 8.10 – 8.03 (m, 4H), 7.50 – 7.41 (m, 6H).

^{13}C{^{1}H} NMR (126 MHz, chloroform-*d*, 300 K): δ [ppm] = 159.8 (br), 130.2, 129.0, 126.7.
(quaternary carbon on the phenyl ring are missing which is typical for this type of molecules.)

The analytical data are consistent with the literature data.[33]

Procedure for diazoalkane 15a synthesis

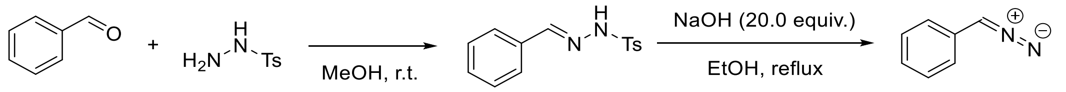

15a

The diazoalkane **15a** was prepared according to the slightly modified literature-known procedure reported by Zhang et. al.[30]To a stirred solution of 4-toluenesulfonyl hydrazide (1.00 equiv., 745 mg, 4.00 mmol) in MeOH (10.0 mL) at 0 °C, benzaldehyde (1.00 equiv., 406 µL, 4.00 mmol) was added dropwise. After approximately 3 hours the crude suspension was directly recrystallized in methanol to afford quantitative conversion to *N'*-benzylidene-4-methylbenzenesulfonohydrazide (1.10 g, 4.00 mmol). The suspension of benzylidene tosylhydrazone (1.10 g, 4.00 mmol) in ethanol (5 mL) was prepared. To the suspension, a solution of sodium hydroxide (400 mg, 8.00 mmol) in ethanol (1 mL) was added. The solution was heated until reflux and stirred at this temperature until no starting material

remains as indicated by TLC analysis. The crude mixture was then cooled, diluted with ice water (50 mL), and the resulting mixture was extracted with *n*-pentane (5×). The combined extracts were washed with saturated aqueous sodium bicarbonate and dried over $MgSO_4$. The volatiles were removed under reduced pressure carefully upon cooling (–20 °C) to yield the phenyldiazomethane **14a** (50%, determined by ^{1}H NMR), which could be used directly without further purification but quantified by adding Tetrakis(trimethylsilyl)silane (TTMSS) as an internal standard.

^{1}H NMR (501 MHz, C_6D_6) δ 7.02 (dd, *J* = 8.3, 7.4 Hz, 2H), 6.82 (t, *J* = 7.4 Hz, 1H), 6.62 – 6.52 (m, 2H), 4.11 (s, 1H). *Note: Due to instability, further characterization data were not recorded, and the mixture was used directly in the subsequent transformation.*

Procedure for diazoalkane 15b synthesis

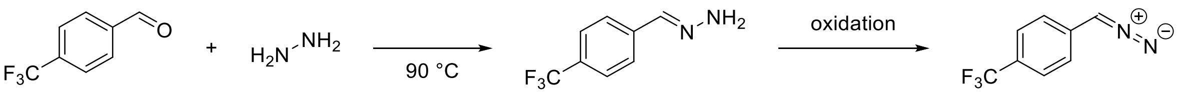

15b

1-(diazomethyl)-4-(trifluoromethyl)benzene **15b** was synthesized based on another literature reports.[31] In a dried Schlenk-pressure tube equipped with magnetic stirring bar, 4-(Trifluormethyl)benzaldehyde (1.00 equiv., 0.68 mL, 5.00 mmol) was added. To the vigorously stirred aldehyde, hydrazine hydrate (16.5 equiv.,4 mL, 65%, 82.4 mmol) was added dropwise. The pressure tube was sealed and heated to 90 °C for 12 hours. The reaction mixture was poured into 10 mL of brine, extracted with CH_2Cl_2 (3 × 10 mL), dried over anhydrous Na_2SO_4 and concentrated to a colorless oil in a 250 mL round bottom flask. The crude hydrazone was flushed with argon and kept cold (−20 °C) until use in the oxidation step. In a separate flask, dimethyl sulfoxide (390 µL, 5.5 mmol, 1.10 equiv.) in 10 mL of CH_2Cl_2 was cooled to −78 °C and oxalyl chloride (450 µL, 5.25 mmol, 1.05 equiv.) was added dropwise via syringe pump over 15 minutes. The oxidant solution was stirred for an additional 15 minutes. During this time, the crude hydrazone was dissolved in 90 mL of Et_2O, cooled to −78 °C and triethylamine (2.10 equiv., 1.47 mL, 10.5 mmol,) was added to the stirred solution. The oxidant was transferred via cannula to the solution of hydrazone and triethylamine which immediately formed a pink solution. After 45 minutes the reaction mixture was quickly extracted in a separatory funnel with ice cold 50% aq. NH_4Cl (100 mL), H_2O (100 mL), and saturated $NaHCO_3$. The combined organic phases were dried by rapidly swirling over K_2CO_3 on an ice bath for 1 minute. The clear red solution was filtered through a sintered glass funnel and then immediately concentrated under high vacuum on a brine/ice bath to yield the desired diazoalkane **14b** as a red oil. The resulting oil was dissolved in benzene-*d*$_6$ and quantify by ^{1}H NMR with internal standard. To provide further proof of the existence of diazoalkane IR spectrum is presented here with a characteristic peak at 2059 cm^{-1}.

^{1}H NMR (501 MHz, C_6D_6) δ 7.23 – 7.15 (m, 1H), 6.38 – 6.32 (m, 1H), 4.13 (s, 1H). *Note: Due to instability, further characterization data were not recorded, and the mixture was used directly in the subsequent transformation.*

4. X-ray characterization data

General information

Single crystal X-ray diffraction analysis

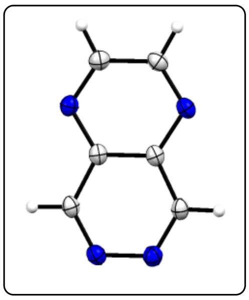

Fig. S1: X-ray crystal structure of compound **5a**. Atomic displacement ellipsoids are shown at 50% probability level.

Crystals of **5a** were grown by slow vapor diffusion of pentane into solution of compound in dichloromethane.

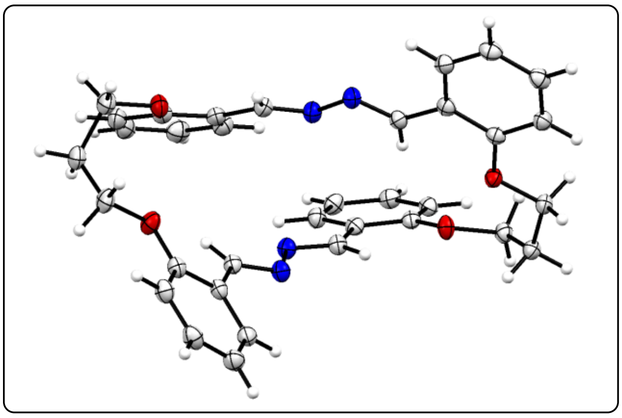

Fig. S2: X-ray crystal structure of compound **5h'**. Atomic displacement ellipsoids are shown at 50% probability level.

Crystals of **5h'** were grown by slow cooling of saturated solution of **5h'** in a THF/benzene mixture to -40 °C in the glove box.

Diffraction data of single-crystals grown from compounds **5e** and **5h'** were collected from flash cooled single crystals at 100(2) K on a Bruker D8 VENTURE dual wavelength Mo/Cu or Ag/Cu four circle diffractometer with a microfocus sealed X-ray tube using mirror optics as monochromator and a Bruker PHOTON II or PHOTON III detector. The diffractometer was equipped with an Oxford Cryostream 800 or 1000 low temperature device. Special Utilities included a SMZ1270 stereomicroscope from Nikon Metrology and a Leica M205M Stereomicroscope which were used for sample preparation. Crystals were mounted on MicroMounts or MicroLoops from MiTeGen using NVH (noise, vibration and harshness) immersion oil. The software used was the APEX5 (2023.9-2) suite and therein integrated

programs SAINT(integration) und SADABS (absorption correction) from Bruker AXS. Structure solution was accomplished with SHELXT.[34] Unless otherwise noted SHELXL (2019/3) was used for refinement against F^2 until convergence using the full-matrix least-squares method. OLEX2[35] was used as the graphical user interface and FinalCIF (https://www.xs3.uni-freiburg.de/research/finalcif) was used for data finalization. All non-hydrogen atoms were refined with anisotropic atomic displacement parameters. The hydrogen atoms were refined isotropically on calculated positions using a riding model with $U_H = 1.5U_C$ for methyl groups, otherwise $U_H = 1.2U_C$.

Structure Tables

	**cu_OCM_PA_YU_513_TBenz_0m-finalcif.cif**	**cu_OCM_PA_YUJL_65_0m-finalcif.cif**
CCDC number	2522765	2522764
Empirical formula	$C_{41}H_{43}N_4O_5$	$C_6H_4N_4$
Formula weight	671.79	132.13
Temperature [K]	100.00	100.00
Crystal system	monoclinic	triclinic
Space group (number)	$P2_1/n$ (14)	$P\overline{1}$ (2)
a [Å]	12.2767(3)	3.7110(3)
b [Å]	10.1749(3)	7.2949(7)
c [Å]	28.6713(8)	11.2452(12)
α [°]	90	94.849(6)
β [°]	100.1880(10)	99.083(8)
γ [°]	90	94.410(6)
Volume [Å^3]	3524.98(17)	298.27(5)
Z	4	2
ρ_{calc} [gcm^{-3}]	1.266	1.471
μ [mm^{-1}]	0.672	0.827
F(000)	1428	136
Crystal size [mm^3]	0.3×0.03×0.02	0.02×0.01×0.005

Crystal colour	clear colourless	colourless
Crystal shape	needle	plate
Radiation	CuK_α (λ=1.54178 Å)	CuK_α (λ=1.54178 Å)
2Θ range [°]	6.26 to 158.41 (0.78 Å)	8.00 to 136.98 (0.83 Å)
Index ranges	$-15 \le h \le 15$<br>$-12 \le k \le 12$<br>$-36 \le l \le 36$	$-4 \le h \le 4$<br>$-8 \le k \le 8$<br>$-13 \le l \le 13$
Reflections collected	48136	3663
Independent reflections	7565<br>R_{int} = 0.0532<br>R_{sigma} = 0.0342	1096<br>R_{int} = 0.0309<br>R_{sigma} = 0.0286
Completeness	100.0 %	99.1 %
Data / Restraints / Parameters	7565/0/451	1096/0/91
Goodness-of-fit on F^2	1.011	1.159
Final R indexes [$I \geq 2\sigma(I)$]	R_1 = 0.0376<br>wR_2 = 0.0863	R_1 = 0.0357<br>wR_2 = 0.1115
Final R indexes [all data]	R_1 = 0.0486<br>wR_2 = 0.0927	R_1 = 0.0419<br>wR_2 = 0.1159
Largest peak/hole [eÅ^{-3}]	0.22/-0.21	0.20/-0.15
Flack X parameter		
Extinction coefficient		

5. Mechanism study

To provide further mechanistic insight, we present key control experiments that support our proposed mechanism concerning the reactivity of diazoalkanes.

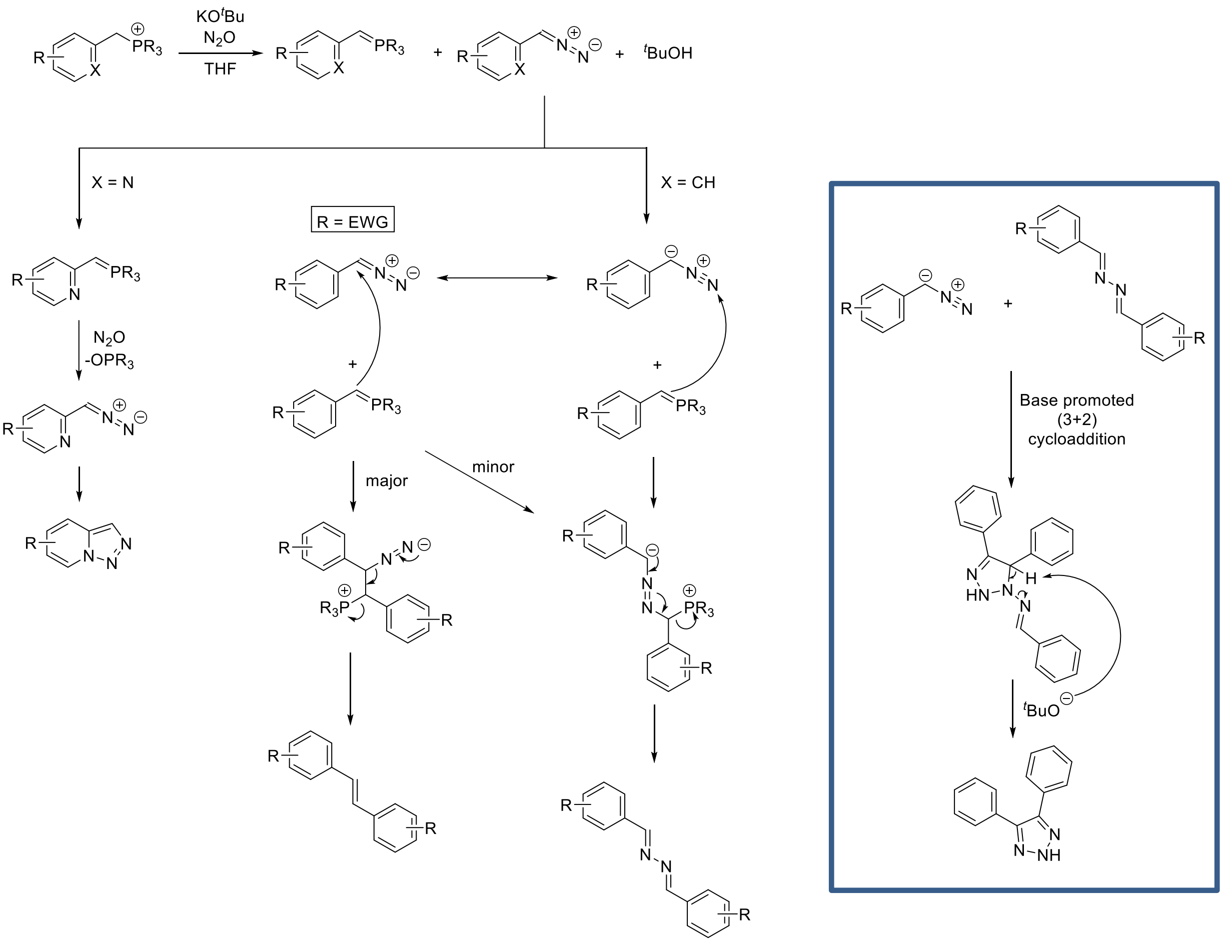

Scheme S1. Plausible over-all reaction mechanisms.

Here, we illustrate the overall mechanisms of each synthetic transformation involving diazoalkanes described in this work. Among these, the rearrangement of 2-(diazomethyl)pyridines to triazolopyridines **3** is well-established in the literature. Similarly, the base-promoted pyrolysis of diazoalkanes leading to symmetric triazoles has also been reported.

Procedure for mechanistic experiment A

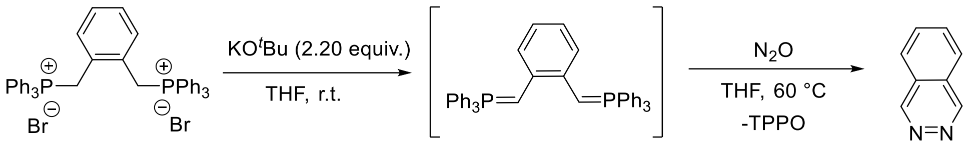

According to **GP6**, (1,2-phenylenebis(methylene))bis(triphenylphosphonium) bromide **4b** (158 mg, 200 µmol) and potassium *tert*-butoxide (49.4 mg, 440 µmol) were employed. After completion of the reaction, volatiles were removed under reduced pressure, and the residue was directly analyzed by NMR spectroscopy in $CDCl_3$. Both triphenylphosphine and triphenylphosphine oxide were observed in the ^1H and ^{31}P NMR spectra in an approximate ratio of 1:1.5. (Fig. S3-S4).

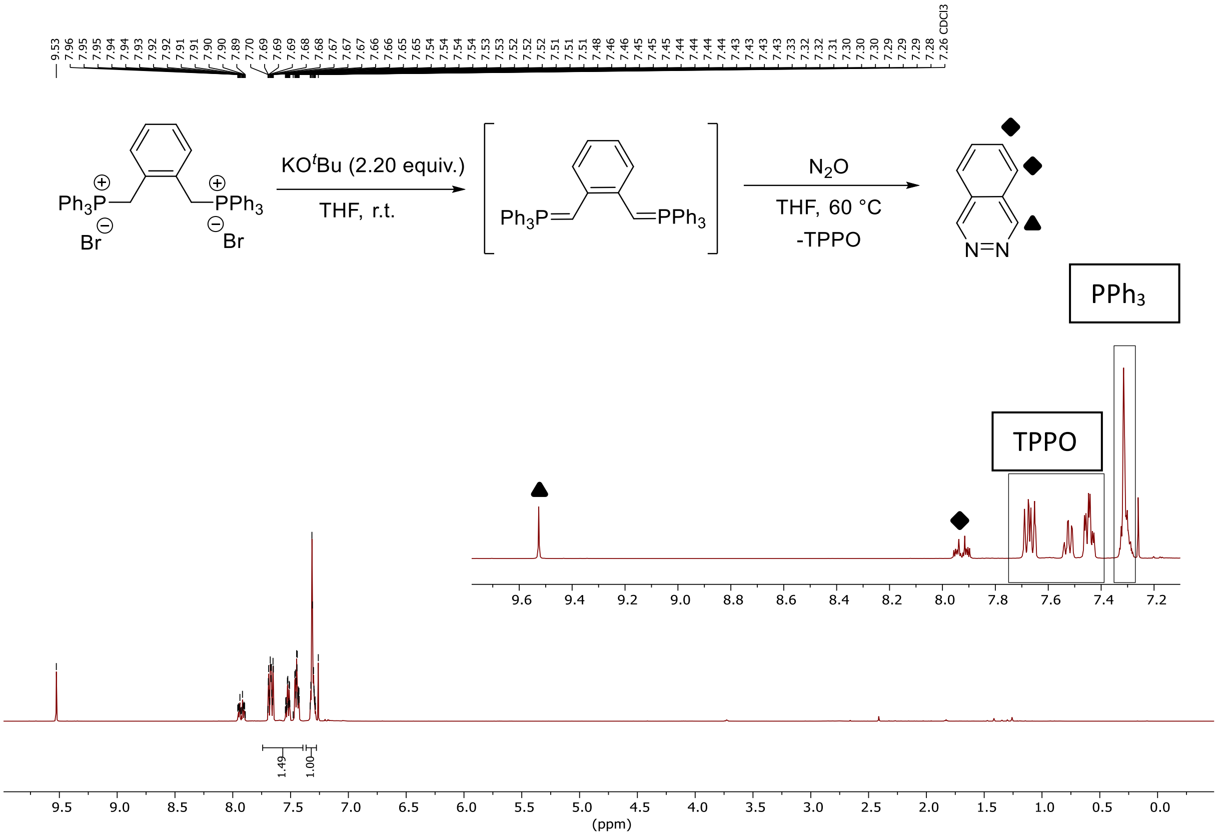

Fig. S3 ^1H NMR of crude reaction mixture of phthalazine **5b** formation

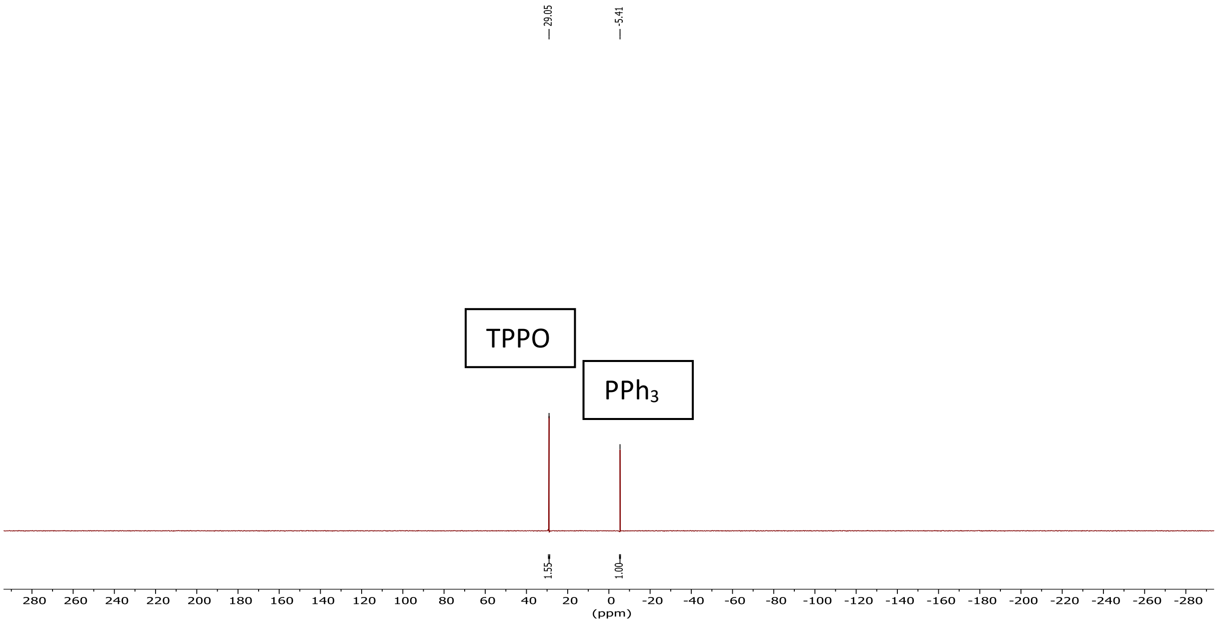

Fig. S4 ^{31}P NMR of crude reaction mixture of phthalazine **5b** formation

Procedure for mechanistic experiment B

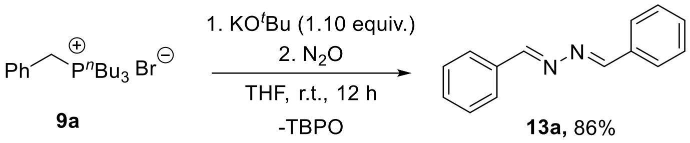

According to the **GP8** employing benzyltributylphosphonium bromide **9a** (74.7 mg, 200 µmol) and potassium *tert*-butoxide (24.7 mg, 220 µmol) were employed. After completion of the reaction, volatiles were removed under reduced pressure, and the residue was directly analyzed by NMR spectroscopy in d_8-THF Only triphenylphosphine oxide were observed in the ^{1}H and ^{31}P NMR spectra (Fig. S5-S6). The desired compound can be isolated according to the previously described method to afford an 86% yield (86.2 µmol, 18.0 mg).

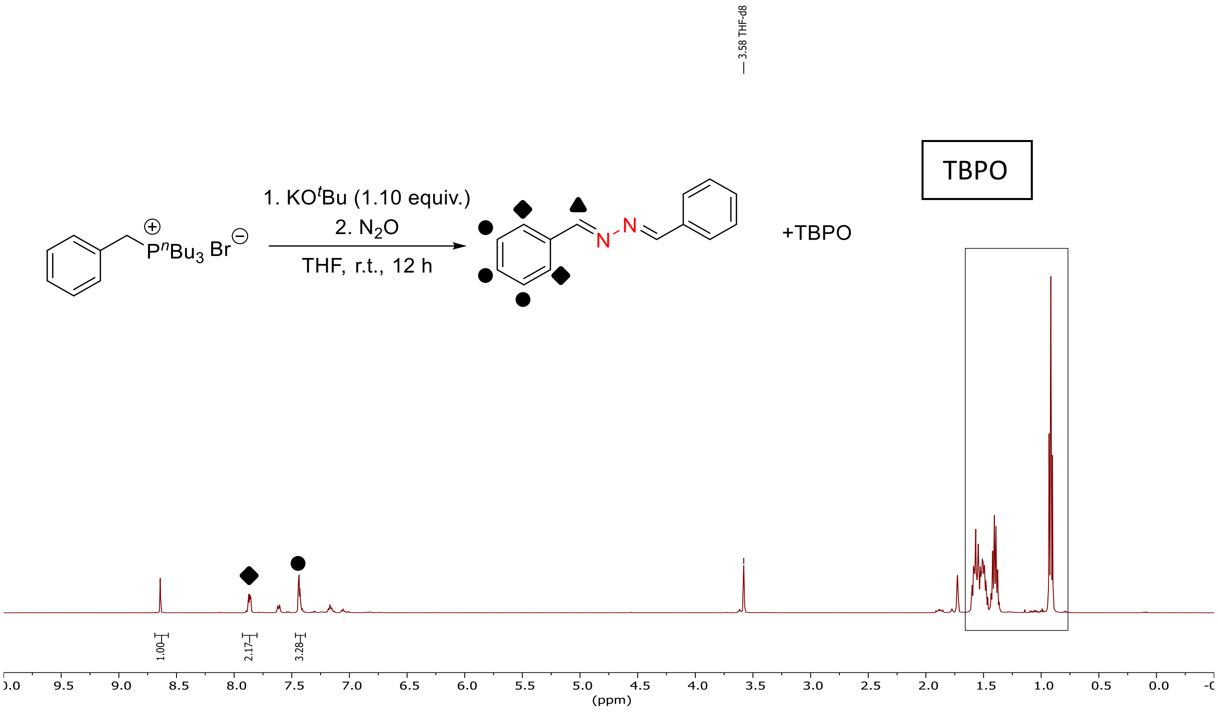

Fig. S5 ^{1}H NMR of crude reaction mixture of aldazine **13a** formation prior to air exposure

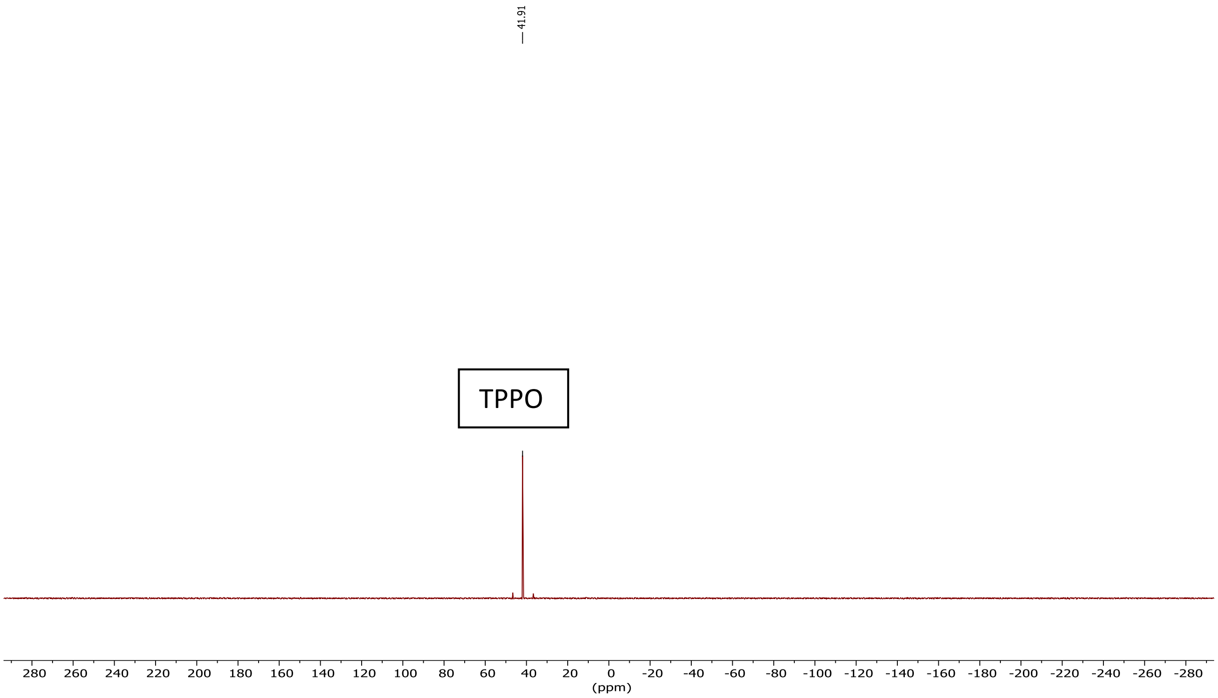

Fig. S6 ^{31}P NMR of crude reaction mixture of aldazine **13a** formation prior to air exposure

Procedure for mechanistic experiment C

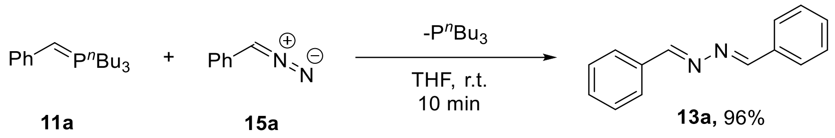

In the glove box both phosphorus ylide **11a** (1.00 equiv., 0.50 mmol) and phenyldiazomethane **15a** (1.00 equiv., 0.50 mmol) were dissolved in 0.25 mL THF-d^8 to mimic the optimized condition, and directly mixed in a J.Young valve NMR tube. The reaction mixture was directly submitted to the NMR measurement (Fig. S7-S8). From the resulting spectrum, we can see the major phosphorus related species is tributyl phosphine which align with the literature and the observation in the diazine synthesis. In addition, quantification of the substance by the internal standard results in an almost quantitative transformation, showing the great efficiency of this conversion. The desired azine 1**3a** could be isolated in 96% yield in this reaction.

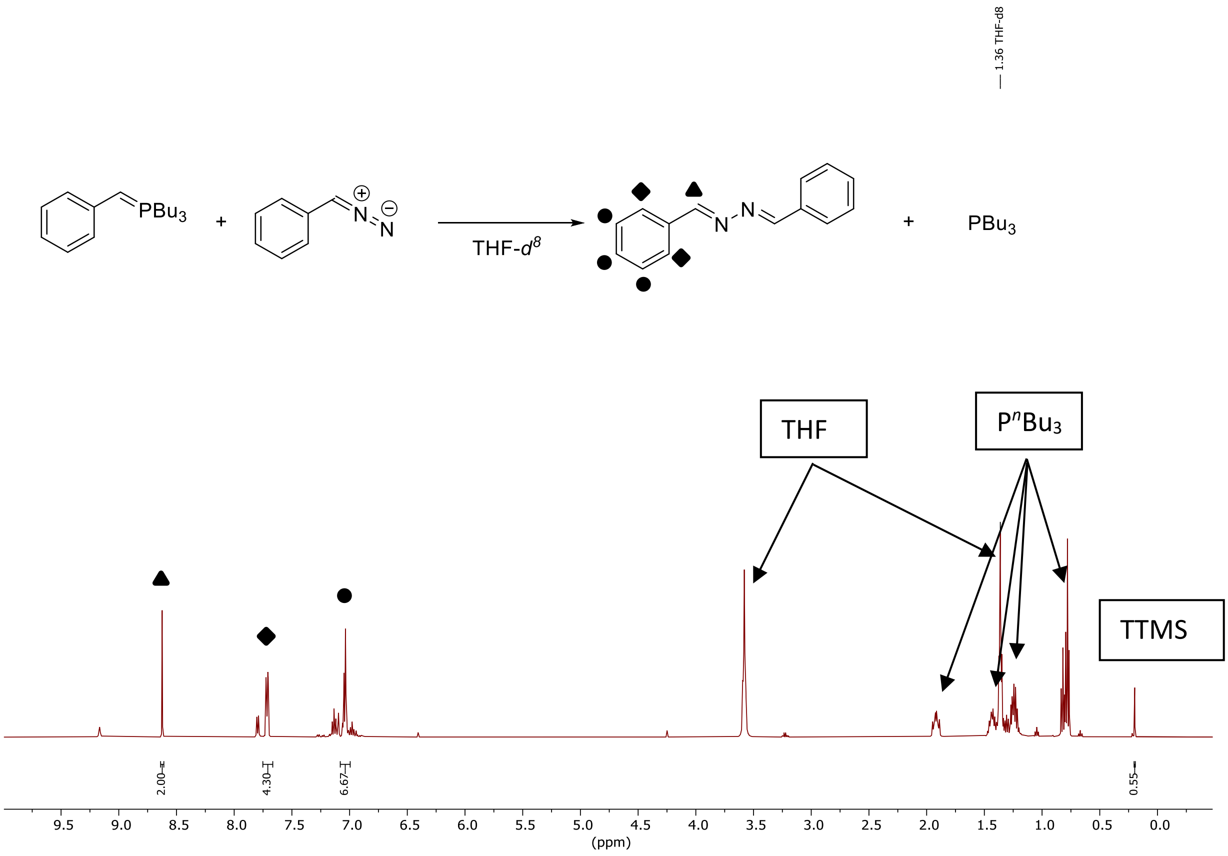

Fig. S7 Crude ^{1}H NMR of reaction between phosphorus ylide **11a** and phenyldiazomethane **15a**

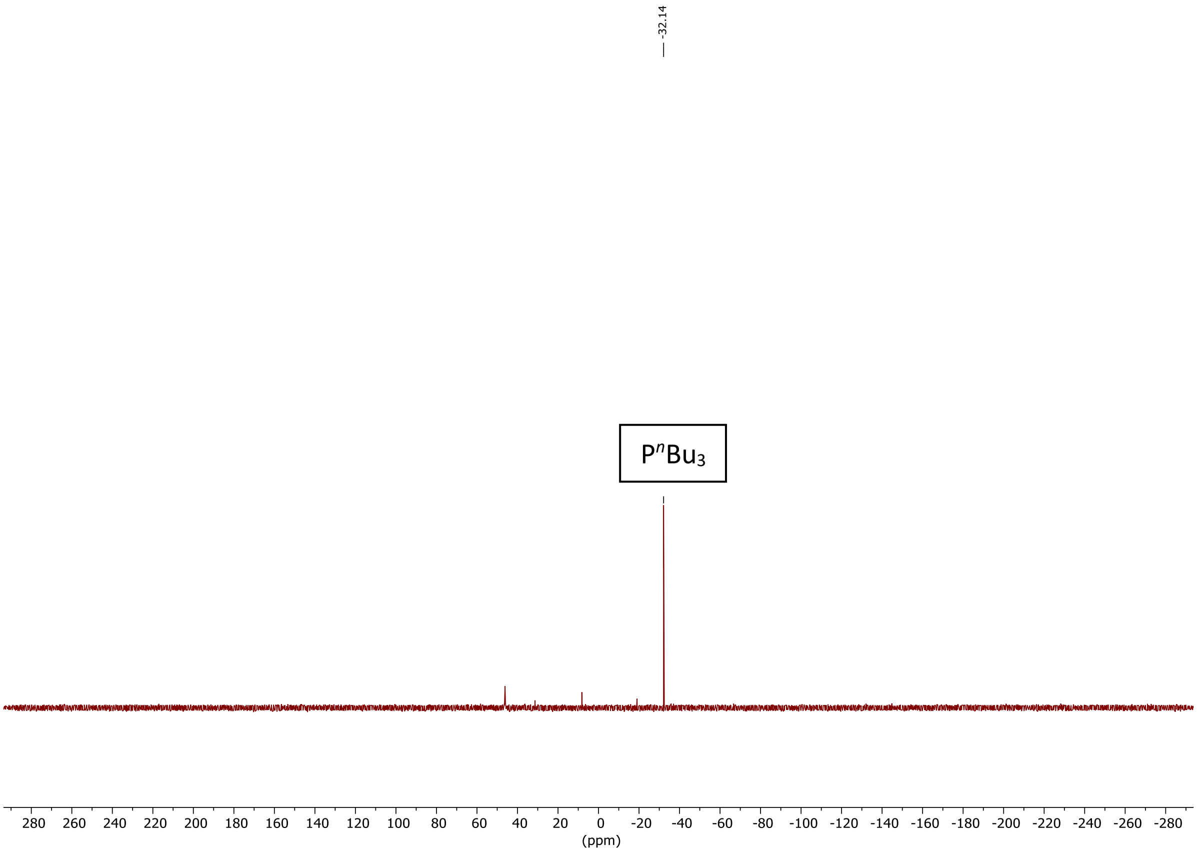

Fig. S8 Crude ^{31}P NMR of reaction between phosphorus ylide **11a** and phenyldiazomethane **15a**

Procedure for mechanistic experiment D

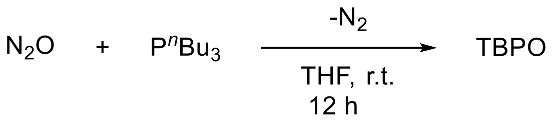

In a dried pressure Schlenk flask, tributylphosphine (1.0 mmol, 249 µL) was dissolved in THF. At −78 °C, the solution was evacuated to remove the inert gas and then repressurized with N_2O. The mixture was slowly warmed to room temperature and stirred for 12 h. Volatiles were removed under reduced pressure, and the residue was submitted to NMR analysis (Fig. S9). The resulting spectra showed complete conversion of the phosphine to the corresponding phosphine oxide.

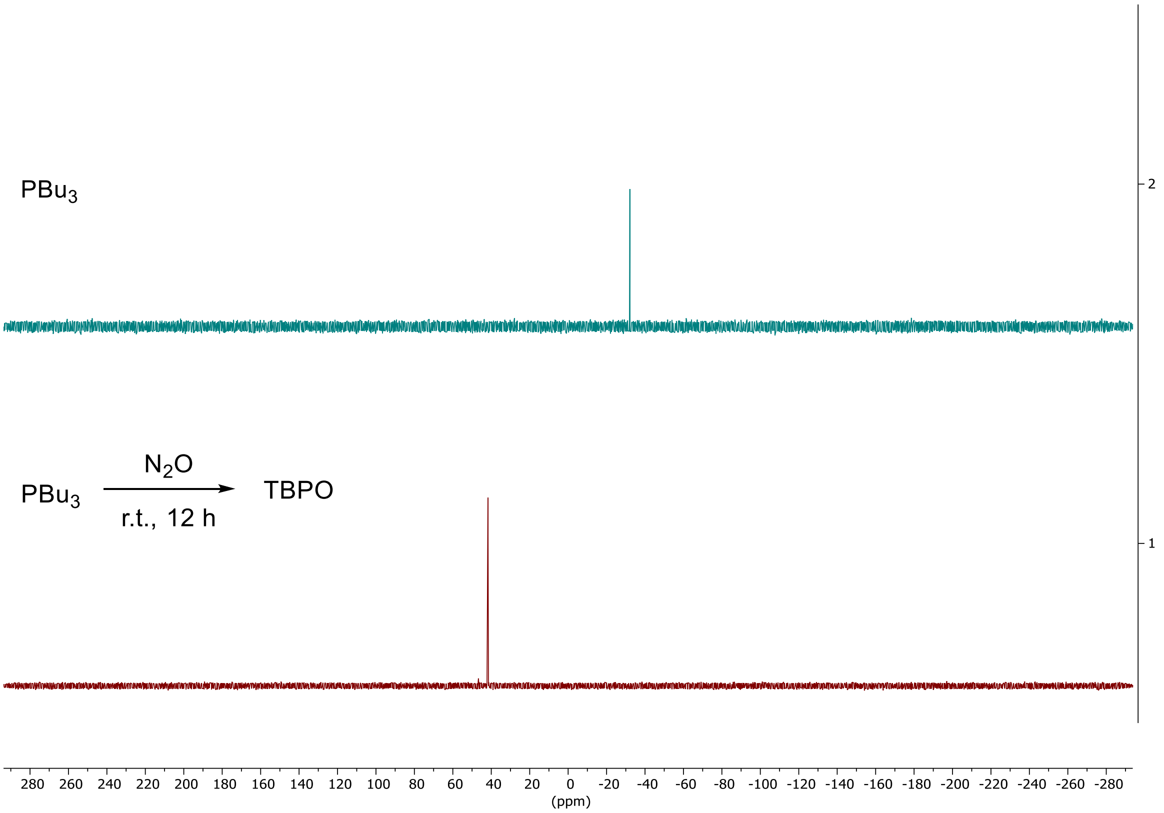

Fig. S9 ^{31}P NMR of Tributylphosphine before and after treatment with N_2O

Procedure for mechanistic experiment E

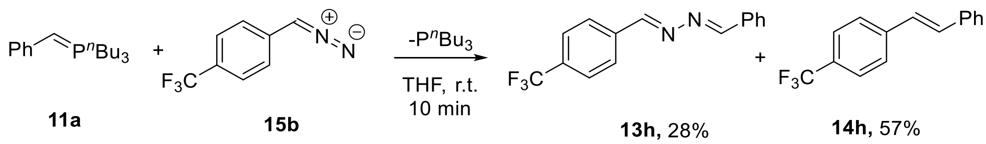

The solution of diazoalkane **15b** (1.00 mmol)in benzene was transferred to a flask containing the freshly prepared phosphorus ylide **11a** (1.00 mmol) under a nitrogen atmosphere. The reaction mixture was stirred at ambient temperature for 10 minutes. The crude reaction mixture was then analyzed by both ^{1}H and ^{31}P NMR spectroscopy (Fig. S10-S11). The resulting spectra indicated complete consumption of the phosphorus ylide, as tributylphosphine was the only phosphorus-containing species observed. However, the ^{1}H NMR spectrum appeared complex; therefore, we proceeded to isolate the product from this reaction in order to quantify the species formed during the transformation. To our delight, the (*E*) -styryl-4-(trifluoromethyl)benzene **14h** can be isolated in 57% (142 mg, 0.57mmol). On the other hand, the asymmetric azine **13h** could also be found in 28% isolated yield (77.4 mg, 0.28 mmol).

1-benzylidene-2-(4-(trifluoromethyl)benzylidene)hydrazine (13h)

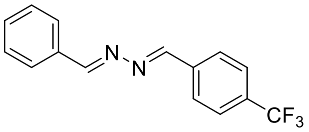

m.p. = 70 – 71 °C

^{1}H NMR (501 MHz, chloroform-*d*, 298 K): δ [ppm] = 8.70 – 8.65 (m, 2H, N=C*H*), 8.00 – 7.93 (m, 1H, Ar-*H*), 7.89 – 7.83 (m, 3H, Ar-*H*), 7.75 – 7.69 (m, 1H, Ar-*H*), 7.50 – 7.43 (m, 4H, Ar-*H*).

^{13}C{^{1}H} NMR (126 MHz, chloroform-*d*, 298 K): δ [ppm] = 162.2 (N=*C*H), 134.2 (*C*H-Ar), 131.7 (*C*-Ar), 131.4 (*C*H-Ar), 129.0 (*C*-Ar), 128.94 (*C*H-Ar), 128.87 (*C*H-Ar), 128.8 (*C*H-Ar), 128.7 (*C*H-Ar), 125.87 (q, *J* = 4.0 Hz, *C*H-Ar).

^{19}F NMR (565 MHz, chloroform-*d*, 298 K): δ [ppm] = -62.8 (m)

IR (ATR) [cm^{-1}]: ṽ = 2932, 2857, 1626, 1576, 1492, 1449, 1413, 1324, 1212, 1163, 1119, 1108, 1066, 1015, 954, 841, 753, 691, 674, 646, 600, 523, 497, 448, 430

HRMS-ESI(+): calc. $C_{15}H_{12}F_3N_2$ $[M+H]^+$ 277.0947; found 277.0939

Note: Due to the presence of multiple conformers, the NMR spectra appear as a mixture, resulting in inconsistent integration values that do not correspond to the expected proton count. In the ^{13}C{^{1}H} NMR spectrum, the signals of the major conformer were assigned and verified by 2D NMR experiments.

However, due to spectral complexity and concentration effects, the signal corresponding to the CF_3 group could not be unambiguously assigned.

(*E*)-1-styryl-4-(trifluoromethyl)benzene (14h)

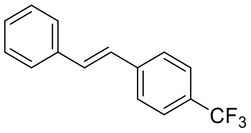

^{1}H NMR (501 MHz, chloroform-*d*, 298 K): δ [ppm] = 7.65 – 7.59 (m, 4H), 7.57 – 7.54 (m, 2H), 7.44 – 7.39 (m, 2H), 7.36 – 7.31 (m, 1H), 7.21 (d, *J* = 16.4 Hz, 1H), 7.13 (d, *J* = 16.3 Hz, 1H).

^{13}C{^{1}H} NMR (126 MHz, chloroform-*d*, 298 K): δ [ppm] = 141.0, 136.8, 131.3, 129.4 (q, *J* = 32.5 Hz), 128.9, 128.4, 127.3, 126.9, 126.7, 125.7 (q, *J* = 3.8 Hz), 124.4 (q, *J* = 271.8 Hz).

The analytical data are consistent with the literature data.[36]

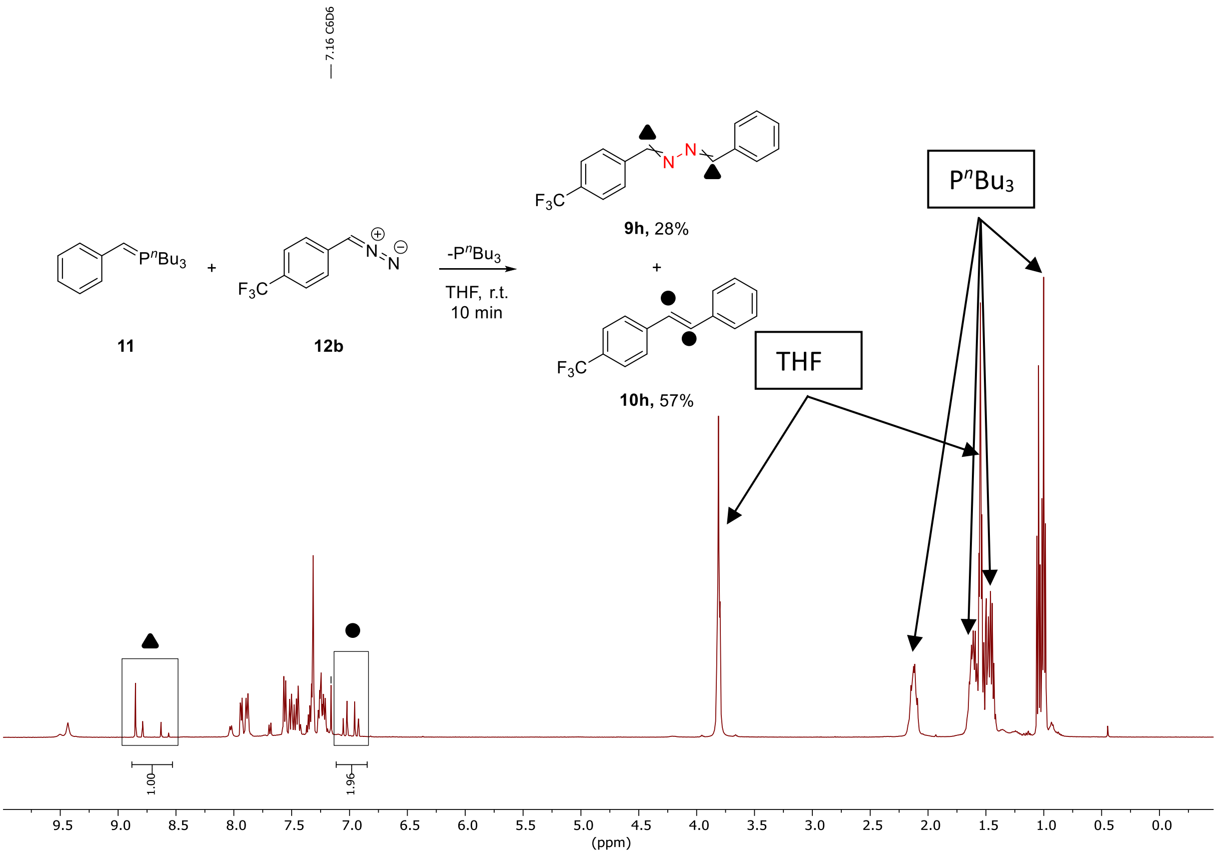

Fig. S10 Crude 1H NMR of reaction between phosphorus ylide **11a** and 1-(diazomethyl)-4-(trifluoromethyl)benzene **15b**

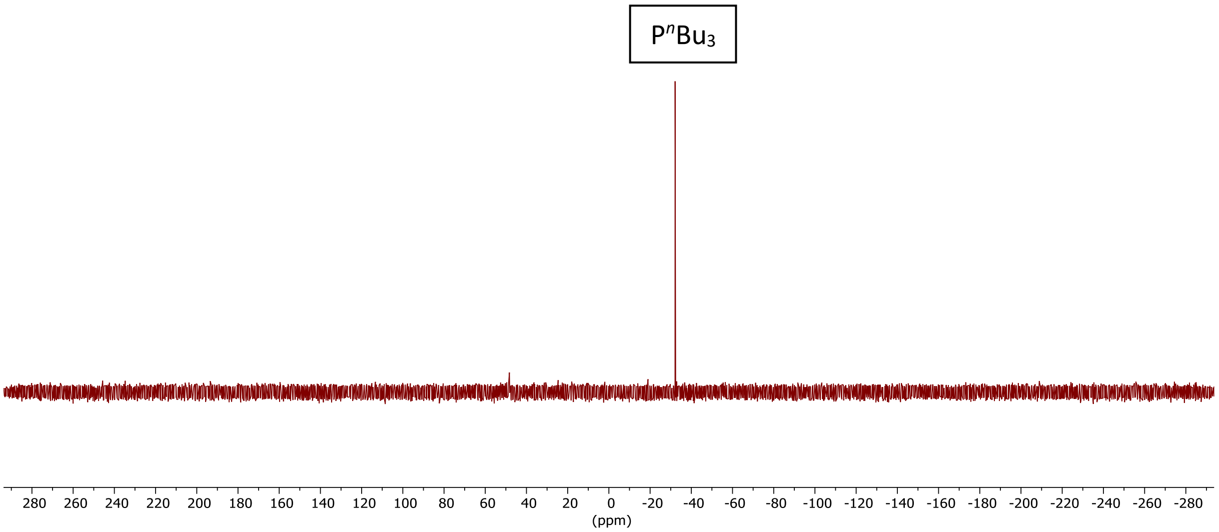

Fig. S11 Crude ^{31}P NMR of reaction between phosphorus ylide **11a** and 1-(diazomethyl)-4-(trifluoromethyl)benzene **15b**

6. Computational details

All density functional theory (DFT) calculations were performed using the Gaussian 16 program package[37] (Gaussian 16 Rev. C.01) employing the hybrid functional PBE0 with the def2-TZVP[38] basis set, defined by Weigend and Ahlrichs. In order to take dispersion effects into account, Grimme's D3 dispersion correction along with Becke-Johnson damping was used.[39–42] Solvents effects were considered implicitly with the SMD solvation model for THF. Gibbs free reaction energies and enthalpies were calculated for standard conditions (p = 1 atm, T = 298 K). Geometries of stationary points were fully optimized without any symmetry constraints. To test for minima or saddle points, harmonic vibrational frequency calculations were performed. The absence of imaginary frequencies (NIMAG = 0) confirmed a minimum, while a transition state was confirmed by an imaginary frequency (NIMAG = 1). To check whether a transition state connects related minima, the intrinsic reaction coordinate (IRC) was calculated. XYZ-data were generated employing GaussView.[43] For the visualization of computed IboView was used.[44]

The computational results reveal all key transition states and intermediates involved in the cycloaddition/retro-cycloaddition sequence. Starting from the trialkyl phosphorus ylide (***P*-ylide 1**), a rapid equilibrium exists between the *s-cis* and *s-trans* conformers. The thermodynamically favored ***s-cis P*-ylide 1** undergoes addition to nitrous oxide via **TS1** to generate zwitterionic **Int1**, which corresponds to the rate-determining step. The calculated activation barrier (ΔG* = 21.1 kcal/mol) is consistent with the experimental observation that the reaction proceeds at room temperature over 12 hours. Subsequent cyclization affords **Int2.1**, which then undergoes flipping of the pyridine ring to give the dynamically more favorable **Int2.2**. This intermediate is kinetically favored over the **Int2.1** for the subsequent cycloreversion via **TS4.1** (ΔG* = 19.4 kcal/mol), producing ***s*-trans-2-(diazomethyl)pyridine**. Finally, spontaneous isomerization yields ***s*-cis-2-(diazomethyl)pyridine**, which can undergo the subsequent cyclization.

In the case of triphenyl phosphorus ylide (***P*-ylide 2**), the reaction pathway is essentially the same but proceeds with a higher activation barrier (ΔG^{*} = 24.0 kcal/mol), rationalizing the need for heating and a longer reaction time.

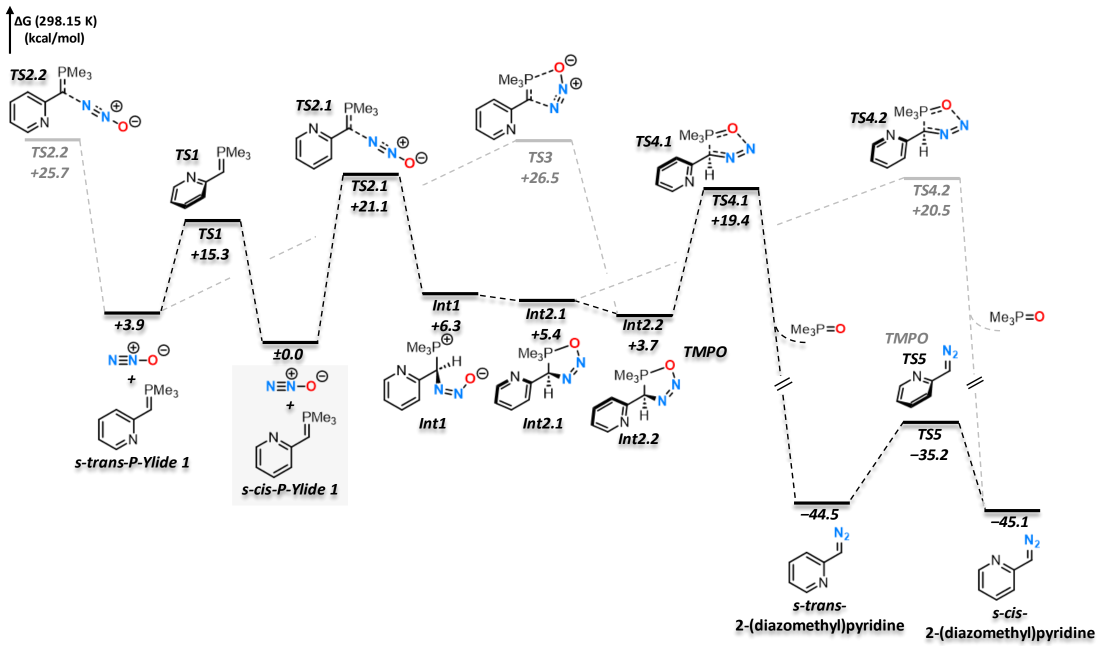

Fig. S12: Computed energy profile at the PBE0-D3(BJ)/def2-TZVP/SMD(THF) level of theory for reaction of ***P*-ylide 1** with N_2O forming **2-(diazomethyl)pyridine** along with trimethylphosphine oxide.

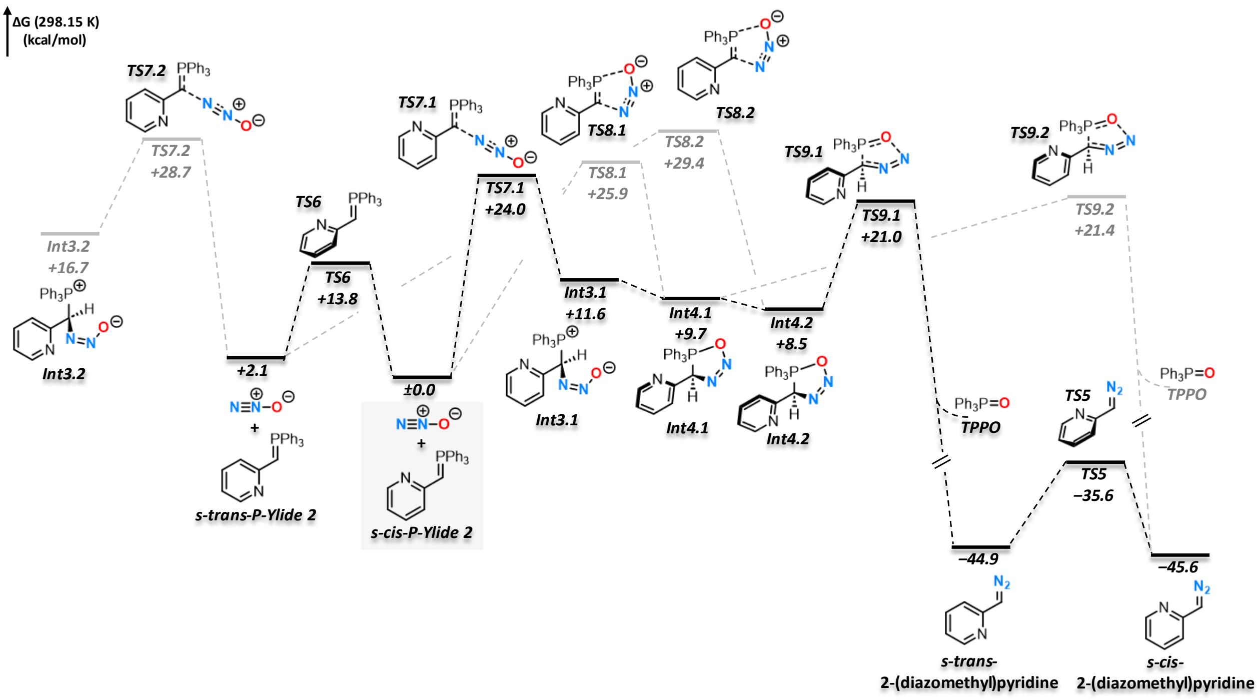

Fig. S13: Computed energy profile at the PBE0-D3(BJ)/def2-TZVP/SMD(THF) level of theory for reaction of ***P*-ylide 2** with N_2O forming **2-(diazomethyl)pyridine** along with triphenylphosphine oxide.

***s-cis-P*-Ylide-1**

```
# opt freq PBE0-D3(BJ)/def2-TZVP SMD(THF)
```

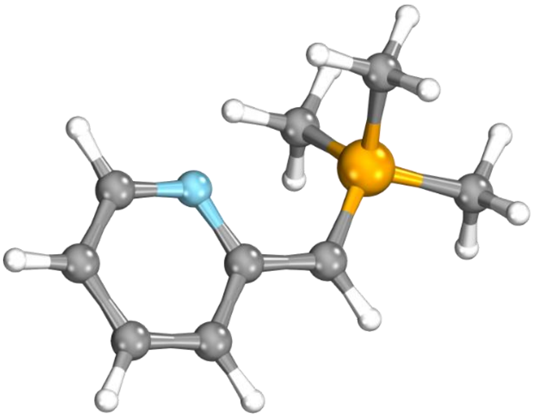

Fig. S14: Computed structure of ***s-cis-P*-Ylide-1**.

```
0 1
 C                 -2.32620800   -1.31626000    0.00004100
 N                 -1.09708500   -0.81218700    0.00001600
 C                 -0.93789400    0.53751700   -0.00002200
 C                 -2.08171000    1.37932700   -0.00003300
 C                 -3.33814400    0.82777800   -0.00000700
 C                 -3.48590100   -0.56252500    0.00003200
 C                  0.37515700    1.05762600   -0.00004800
 P                  1.75557400    0.05296200   -0.00000800
 C                  3.20356900    1.11483700   -0.00004800
 C                  1.92756300   -1.03619000    1.42771500
 C                  1.92757000   -1.03630700   -1.42764100
 H                 -2.38867900   -2.40342400    0.00007100
 H                 -1.94735800    2.45562900   -0.00006200
 H                 -4.21215600    1.47198800   -0.00001500
 H                 -4.45932400   -1.03655600    0.00005300
 H                  0.54708300    2.12680100   -0.00007700
 H                  4.11006000    0.50715000   -0.00002600
 H                  3.19311900    1.74641900   -0.89014600
 H                  3.19312100    1.74648500    0.89000400
 H                  2.84210600   -1.62866200    1.35006300
 H                  1.95737500   -0.43005900    2.33506700
 H                  1.06356400   -1.70028700    1.46968300
 H                  2.84211500   -1.62876800   -1.34993800
 H                  1.95738300   -0.43024900   -2.33504300
 H                  1.06357300   -1.70041000   -1.46955700

         Item               Value     Threshold  Converged?
 Maximum Force            0.000041     0.000450     YES
 RMS     Force            0.000004     0.000300     YES
 Maximum Displacement     0.000099     0.001800     YES
 RMS     Displacement     0.000020     0.001200     YES
 Predicted change in Energy=-3.006840D-09
 Optimization completed.
    -- Stationary point found.

SCF Done:  E(RPBE1PBE) =  -747.061744696     A.U. after    1 cycles

-------------------
 - Thermochemistry -
 -------------------

Zero-point correction=                           0.208663 (Hartree/Particle)
 Thermal correction to Energy=                    0.221186
 Thermal correction to Enthalpy=                  0.222130
 Thermal correction to Gibbs Free Energy=         0.170372
 Sum of electronic and zero-point Energies=           -746.853082
```

Sum of electronic and thermal Energies= -746.840559
Sum of electronic and thermal Enthalpies= -746.839615
Sum of electronic and thermal Free Energies= -746.891373

	E (Thermal)	CV	S
	KCal/Mol	Cal/Mol-Kelvin	Cal/Mol-Kelvin
Total	138.796	47.483	108.934

2,-0.5904262,-0.00005,-0.0000919\PG=C01 [X(C9H14N1P1)]\NImag=0\\0.6848

***s-trans-P*-Ylide-1**

opt freq PBE0-D3(BJ)/def2-TZVP SMD(THF)

0 1

C	3.23327000	-0.96587500	-0.00001300
N	1.97170800	-1.37700800	-0.00007400
C	0.97322600	-0.45452300	-0.00003200
C	1.30776600	0.92626500	0.00007200
C	2.62378000	1.32023000	0.00013600
C	3.63473100	0.35913200	0.00009400
C	-0.35525500	-0.94655300	-0.00009500
P	-1.80498700	-0.05138900	-0.00003300
C	-3.16550800	-1.21977500	-0.00010600
C	-2.06677300	1.02600900	1.42614500
C	-2.06677900	1.02620200	-1.42606500
H	3.98324100	-1.75563700	-0.00005100
H	0.52693000	1.67787800	0.00010200
H	2.86964800	2.37773100	0.00021600
H	4.68398600	0.62615600	0.00014100
H	-0.48991900	-2.02245100	-0.00017300
H	-4.11385800	-0.67976800	-0.00007800
H	-3.10599300	-1.84780600	-0.89048500
H	-3.10599700	-1.84790800	0.89020300
H	-3.02065200	1.55119100	1.33965800
H	-2.06130600	0.41685700	2.33193500
H	-1.26099900	1.75911800	1.49304900
H	-3.02064700	1.55139000	-1.33949900
H	-2.06133300	0.41716900	-2.33193500
H	-1.26099000	1.75930300	-1.49288300

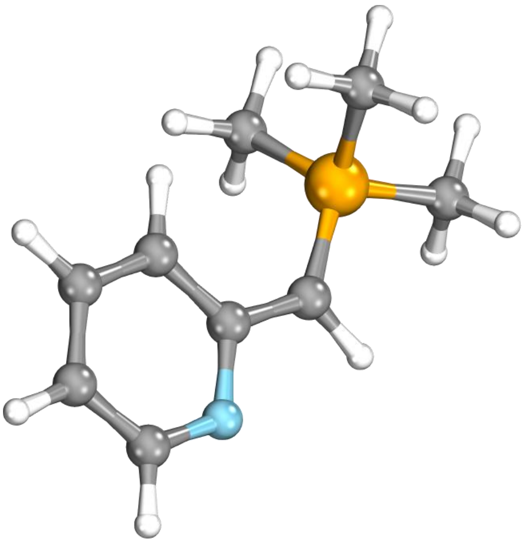

Fig. S15: Computed structure of ***s-trans-P*-Ylide 1.**

Item	Value	Threshold	Converged?
Maximum Force	0.000044	0.000450	YES
RMS Force	0.000004	0.000300	YES
Maximum Displacement	0.000081	0.001800	YES
RMS Displacement	0.000022	0.001200	YES

Predicted change in Energy=-3.437691D-09
Optimization completed.

```
    -- Stationary point found.

SCF Done:  E(RPBE1PBE) =  -747.056409600     A.U. after    1 cycles

-------------------
 - Thermochemistry -
 -------------------

Zero-point correction=                           0.209206 (Hartree/Particle)
 Thermal correction to Energy=                    0.221522
 Thermal correction to Enthalpy=                  0.222466
 Thermal correction to Gibbs Free Energy=         0.171185
 Sum of electronic and zero-point Energies=           -746.847203
 Sum of electronic and thermal Energies=              -746.834888
 Sum of electronic and thermal Enthalpies=            -746.833944
 Sum of electronic and thermal Free Energies=         -746.885224

                     E (Thermal)             CV                S
                      KCal/Mol        Cal/Mol-Kelvin    Cal/Mol-Kelvin
 Total                  139.007             47.228           107.929

01 [X(C9H14N1P1)]\NImag=0\\0.71577219,-0.03493470,0.72582748,0.0000115
```

N_2O

opt freq PBE0-D3(BJ)/def2-TZVP SMD(THF)

```
N                 0.00000000    0.00000000   -1.18855300
N                 0.00000000    0.00000000   -0.07159400
O                 0.00000000    0.00000000    1.10262800
```

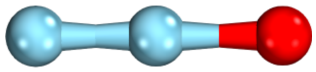

Fig. S16: Computed structure of **N_2O**.

```
       Item               Value        Threshold     Converged?
 Maximum Force            0.000018     0.000450      YES
 RMS     Force            0.000009     0.000300      YES
 Maximum Displacement     0.000012     0.001800      YES
 RMS     Displacement     0.000008     0.001200      YES
 Predicted change in Energy=-2.148275D-10
 Optimization completed.
    -- Stationary point found.

SCF Done:  E(RPBE1PBE) =  -184.544017808     A.U. after    1 cycles

-------------------
 - Thermochemistry -
 -------------------

 Zero-point correction=                           0.011507 (Hartree/Particle)
 Thermal correction to Energy=                    0.014156
```

```
Thermal correction to Enthalpy=                  0.015100
Thermal correction to Gibbs Free Energy=        -0.009766
Sum of electronic and zero-point Energies=           -184.532510
Sum of electronic and thermal Energies=              -184.529862
Sum of electronic and thermal Enthalpies=            -184.528918
Sum of electronic and thermal Free Energies=         -184.553784

                    E (Thermal)           CV                S
                     KCal/Mol        Cal/Mol-Kelvin    Cal/Mol-Kelvin
Total                    8.883            6.988            52.335

=C*V [C*(N1N1O1)]|NImag=0||0.03849231,0.,0.03849231,0.,0.,1.32769300,-
```

Transition state TS1

opt freq PBE0-D3(BJ)/def2-TZVP SMD(THF)

```
0 1
 C                -2.57714800    1.28417000   -0.03982000
 N                -1.33471600    1.03470400   -0.44459200
 C                -0.91971500   -0.24279200   -0.53963900
 C                -1.78940400   -1.28827400   -0.20982700
 C                -3.08091300   -1.01994200    0.21001800
 C                -3.49196800    0.30035200    0.29909400
 C                 0.46297700   -0.53528600   -0.98992200
 P                 1.74985200   -0.03684500   -0.00542700
 H                -2.85967000    2.33295700    0.01878800
 H                -1.43101000   -2.30739400   -0.28955300
 H                -3.75609900   -1.82990900    0.46540500
 H                -4.49160600    0.56561100    0.62223800
 H                 0.65422000   -0.34407000   -2.04757700
 C                 1.58871300   -0.71684100    1.65406900
 H                 0.65292600   -0.36618500    2.09555600
 H                 1.56887700   -1.80627400    1.60159900
 H                 2.41874300   -0.39006600    2.28360800
 C                 2.04235000    1.73311700    0.30276300
 H                 2.21703900    2.23399700   -0.65228900
 H                 1.14893900    2.16461700    0.75881000
 H                 2.90011200    1.89410000    0.96108800
 C                 3.30211300   -0.61953600   -0.69969500
 H                 3.41239500   -0.21985300   -1.71029900
 H                 4.14102200   -0.27850200   -0.08986000
 H                 3.29730400   -1.70909600   -0.74621100
```

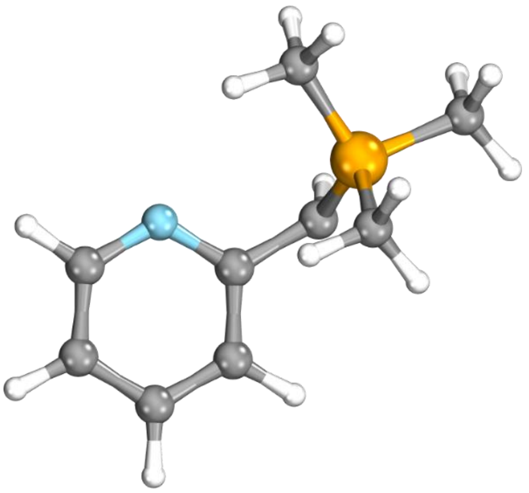

Fig. S17: Computed structure of **TS1**.

```
        Item               Value     Threshold  Converged?
Maximum Force            0.000004     0.000450     YES
RMS     Force            0.000001     0.000300     YES
Maximum Displacement     0.000848     0.001800     YES
```

```
 RMS     Displacement     0.000162     0.001200     YES
 Predicted change in Energy=-1.948603D-09
 Optimization completed.
    -- Stationary point found.

SCF Done:  E(RPBE1PBE) =  -747.036561951     A.U. after    1 cycles

-------------------
 - Thermochemistry -
 -------------------

 Zero-point correction=                           0.207642 (Hartree/Particle)
 Thermal correction to Energy=                    0.219679
 Thermal correction to Enthalpy=                  0.220623
 Thermal correction to Gibbs Free Energy=         0.169580
 Sum of electronic and zero-point Energies=           -746.828920
 Sum of electronic and thermal Energies=              -746.816883
 Sum of electronic and thermal Enthalpies=            -746.815939
 Sum of electronic and thermal Free Energies=         -746.866982

                   E (Thermal)             CV                S
                    KCal/Mol        Cal/Mol-Kelvin    Cal/Mol-Kelvin
 Total                137.850            45.779           107.429

1 [X(C9H14N1P1)]|NImag=1||0.62902197,0.00474902,0.74229942,-0.15919363
```

Transition state TS2.1

\# opt freq PBE0-D3(BJ)/def2-TZVP SMD(THF)

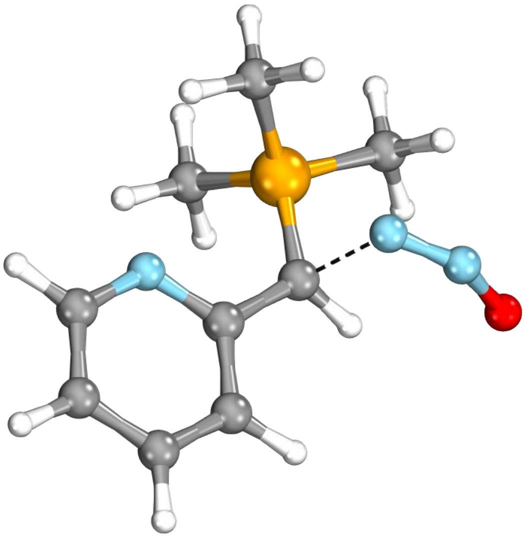

Fig. S18: Computed structure of **TS2.1**.

```
0 1
 C                 2.83983700   -1.12023400    0.58144600
 N                 1.53610200   -0.95532200    0.38270700
 C                 1.11374100    0.14945900   -0.26273400
 C                 2.03165400    1.11373600   -0.72033600
 C                 3.37788300    0.92041700   -0.50771600
 C                 3.80663400   -0.22393100    0.16124600
 C                -0.30049800    0.33135300   -0.43547700
 O                -1.77109500    2.96469700   -0.41601400
 N                -1.37402500    2.54498600    0.66716600
 N                -0.82362300    1.62838600    1.14242600
 P                -1.39151600   -0.99200400   -0.13902100
 C                -3.03293500   -0.44408800   -0.60266000
 C                -1.46301300   -1.50261200    1.57800600
 C                -1.04065200   -2.46946800   -1.10204600
 H                 3.12917300   -2.02728500    1.10739000
 H                 1.66680200    1.99899500   -1.22880400
 H                 4.09551900    1.65538200   -0.85691900
 H                 4.85571500   -0.41320600    0.35150000
 H                -0.63412900    1.00375000   -1.22122100
 H                -3.74004400   -1.26529300   -0.47191900
 H                -3.33441300    0.39518300    0.02547500
 H                -3.03251500   -0.13041600   -1.64793900
 H                -0.48294500   -1.86868200    1.88233500
 H                -1.74067800   -0.64962600    2.19779800
 H                -2.20713500   -2.29503500    1.68475400
 H                -1.07855000   -2.22209300   -2.16461200
 H                -0.04263600   -2.82897000   -0.84942200
 H                -1.77773500   -3.24437000   -0.87945500

         Item               Value     Threshold  Converged?
 Maximum Force            0.000031     0.000450     YES
 RMS     Force            0.000004     0.000300     YES
 Maximum Displacement     0.001196     0.001800     YES
 RMS     Displacement     0.000303     0.001200     YES
 Predicted change in Energy=-1.337277D-08
 Optimization completed.
    -- Stationary point found.

SCF Done:  E(RPBE1PBE) =  -931.583989665     A.U. after    1 cycles

-------------------
 - Thermochemistry -
 -------------------

 Zero-point correction=                           0.220455 (Hartree/Particle)
 Thermal correction to Energy=                    0.236125
```

Thermal correction to Enthalpy= 0.237069
Thermal correction to Gibbs Free Energy= 0.176894
Sum of electronic and zero-point Energies= -931.363534
Sum of electronic and thermal Energies= -931.347865
Sum of electronic and thermal Enthalpies= -931.346921
Sum of electronic and thermal Free Energies= -931.407096

	E (Thermal)	CV	S
	KCal/Mol	Cal/Mol-Kelvin	Cal/Mol-Kelvin
Total	148.170	56.781	126.649

095,12.3884617,0.4095939,-1.7393838\PG=C01 [X(C9H14N3O1P1)]\NImag=1\\0

Transition state TS2.2

opt freq PBE0-D3(BJ)/def2-TZVP SMD(THF)

0 1

C	3.09289100	-1.00307600	0.74312200
C	1.16694400	0.07893900	-0.21944500
C	3.28029600	0.83653400	-0.73921600
C	3.90591100	-0.11150000	0.05485300
C	-0.25121500	0.28408000	-0.38846100
O	-1.81381500	2.87826300	-0.34451800
N	-1.30496700	2.51152900	0.71169800
N	-0.68804500	1.62221600	1.15955400
P	-1.46351400	-0.94381500	-0.15743100
C	-2.98032700	-0.34003600	-0.88816800
C	-1.82235500	-1.32426700	1.56059700
C	-1.07561100	-2.51395900	-0.94487200
H	3.52578600	-1.76505600	1.38267300
H	3.87532800	1.55760100	-1.29541700
H	4.98552100	-0.14585200	0.13124100
H	-0.51464200	0.94370600	-1.21190600
H	-3.76452800	-1.08866900	-0.76281600
H	-3.27796700	0.58983500	-0.40227700
H	-2.82037200	-0.15366100	-1.95156500
H	-0.92985000	-1.68895700	2.07019900
H	-2.17415800	-0.42364700	2.06434000
H	-2.59555800	-2.09439600	1.59947100
H	-0.91649400	-2.34816000	-2.01191100
H	-0.16560700	-2.93028300	-0.50943700
H	-1.89846500	-3.21728900	-0.79906300
C	1.72317700	-0.90987300	0.61199500
H	1.08687500	-1.59784500	1.15442900
N	1.96574000	0.93950300	-0.88307900

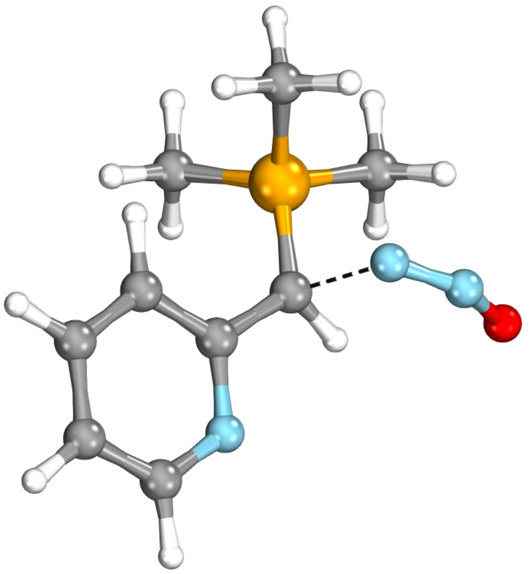

Fig. S19: Computed structure of **TS2.2**.

Item Value Threshold Converged?

```
 Maximum Force            0.000015     0.000450     YES
 RMS     Force            0.000001     0.000300     YES
 Maximum Displacement     0.000891     0.001800     YES
 RMS     Displacement     0.000190     0.001200     YES
 Predicted change in Energy=-1.192244D-09
 Optimization completed.
    -- Stationary point found.

SCF Done:  E(RPBE1PBE) =  -931.577531594     A.U. after    1 cycles

-------------------
 - Thermochemistry -
 -------------------

 Zero-point correction=                           0.220926 (Hartree/Particle)
 Thermal correction to Energy=                    0.236383
 Thermal correction to Enthalpy=                  0.237327
 Thermal correction to Gibbs Free Energy=         0.177728
 Sum of electronic and zero-point Energies=           -931.356606
 Sum of electronic and thermal Energies=              -931.341149
 Sum of electronic and thermal Enthalpies=            -931.340204
 Sum of electronic and thermal Free Energies=         -931.399803

                     E (Thermal)             CV                S
                      KCal/Mol        Cal/Mol-Kelvin    Cal/Mol-Kelvin
 Total                  148.333             56.572           125.437

.3749109,2.5989058,-1.2458978\PG=C01 [X(C9H14N3O1P1)]\NImag=1\\0.70123
```

Intermediate Int1

opt freq PBE0-D3(BJ)/def2-TZVP SMD(THF)

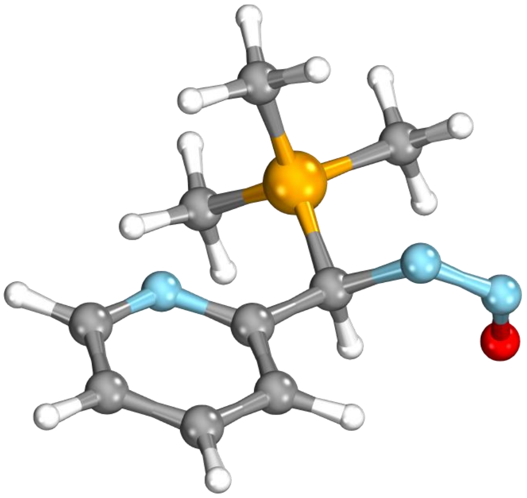

Fig. S20: Computed structure of **Int1**.

```
0 1
 C                  2.84007000   -1.25087100   -0.11770800
 N                  1.53330400   -0.99862200   -0.16699400
 C                  1.12794600    0.26811500   -0.11539600
 C                  2.02006100    1.33195200   -0.00182100
 C                  3.37413300    1.06010500    0.04408600
 C                  3.79993700   -0.26036600   -0.01468000
 C                 -0.34149500    0.54520100   -0.20277600
 O                 -1.92434100    2.44543200   -0.82725100
 N                 -1.53056300    2.45850200    0.36454700
 N                 -0.74881500    1.53670000    0.80074400
 P                 -1.37308500   -0.93480800    0.01842400
 C                 -3.07882300   -0.39966200    0.03408300
 C                 -1.04498400   -1.71938500    1.59033100
 C                 -1.21308900   -2.12036000   -1.31325100
 H                  3.12865300   -2.29747800   -0.15898800
 H                  1.64360700    2.34571300    0.05275300
 H                  4.09201800    1.86848700    0.12817200
 H                  4.85143300   -0.51749000    0.02304300
 H                 -0.60695400    0.92781500   -1.20272100
 H                 -3.71324900   -1.27362100    0.19583000
 H                 -3.23154800    0.31731100    0.84101400
 H                 -3.32828200    0.06894300   -0.91746500
 H                 -0.04406300   -2.14806900    1.59091300
 H                 -1.12699700   -0.96918700    2.37912700
 H                 -1.78765600   -2.50313000    1.75377100
 H                 -1.39259400   -1.61247900   -2.26323700
 H                 -0.21226700   -2.54748900   -1.30778500
 H                 -1.96111200   -2.90509000   -1.17806300

         Item               Value     Threshold  Converged?
 Maximum Force            0.000008     0.000450     YES
 RMS     Force            0.000002     0.000300     YES
 Maximum Displacement     0.001087     0.001800     YES
 RMS     Displacement     0.000261     0.001200     YES
 Predicted change in Energy=-2.176555D-09
 Optimization completed.
    -- Stationary point found.

SCF Done:  E(RPBE1PBE) =  -931.611680902     A.U. after    1 cycles

-------------------
 - Thermochemistry -
 -------------------

 Zero-point correction=                           0.223584 (Hartree/Particle)
 Thermal correction to Energy=                    0.238727
```

```
 Thermal correction to Enthalpy=                  0.239671
 Thermal correction to Gibbs Free Energy=         0.180979
 Sum of electronic and zero-point Energies=           -931.388097
 Sum of electronic and thermal Energies=              -931.372954
 Sum of electronic and thermal Enthalpies=            -931.372009
 Sum of electronic and thermal Free Energies=         -931.430702

                     E (Thermal)             CV                S
                      KCal/Mol        Cal/Mol-Kelvin    Cal/Mol-Kelvin
 Total                  149.804             55.939           123.529
```

403313,16.6703342,-0.4389453,-0.6454968\PG=C01 [X(C9H14N3O1P1)]\NImag=
0\\0.68158970,-0.02060839,0.77013058,0.03330865,0.02909713,0.16497453

Intermediate Int2.1

opt freq PBE0-D3(BJ)/def2-TZVP SMD(THF)

0 1

C	-2.72566800	-0.75329800	1.08716400
N	-1.48184200	-0.84583100	0.62962100
C	-1.15639700	-0.14497200	-0.45590500
C	-2.06619200	0.67141300	-1.11883500
C	-3.35889200	0.76369800	-0.63164600
C	-3.70075300	0.03655100	0.49702800
C	0.25617400	-0.28390400	-0.95295800
O	1.94326600	-1.42891900	0.45761700
N	1.42397900	-2.18648200	-0.45092500
N	0.56990700	-1.68303600	-1.21336700
P	1.46232900	0.55655700	0.17053500
C	3.14973900	0.68702200	-0.46325600
C	1.29328300	0.54937100	1.96576500
C	0.89949000	2.28118400	-0.15114200
H	-2.95610900	-1.34016600	1.97208800
H	-1.76051700	1.21695800	-2.00370500
H	-4.08992600	1.39035800	-1.13016100
H	-4.70055100	0.07349300	0.91208400
H	0.34182500	0.25396400	-1.90125000
H	3.45869100	1.73113000	-0.51791500
H	3.83155100	0.12120500	0.16887700
H	3.17580500	0.25430400	-1.46633600
H	0.30105300	0.16084000	2.20552800
H	2.03827600	-0.10203500	2.41716100
H	1.38633700	1.56526600	2.35052600
H	0.92860900	2.50697800	-1.22014400
H	-0.12012400	2.43148300	0.21156500
H	1.55500300	2.98428000	0.36811800

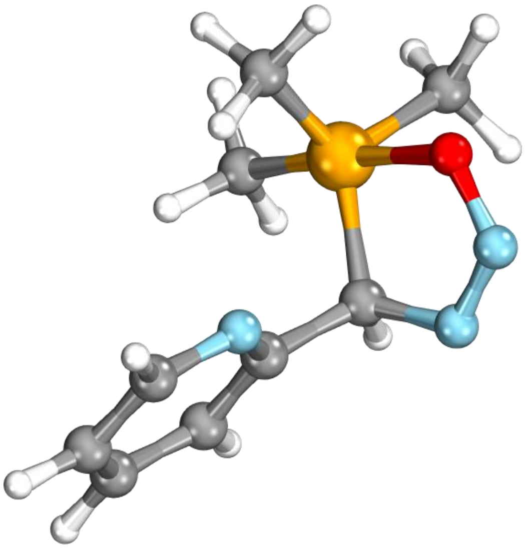

Fig. S21: Computed structure of **Int2.1.**

```
        Item               Value     Threshold  Converged?
 Maximum Force            0.000001     0.000450     YES
 RMS     Force            0.000000     0.000300     YES
 Maximum Displacement     0.000094     0.001800     YES
 RMS     Displacement     0.000023     0.001200     YES
 Predicted change in Energy=-1.073800D-11
 Optimization completed.
    -- Stationary point found.

SCF Done:  E(RPBE1PBE) =  -931.614788139     A.U. after    1 cycles

-------------------
 - Thermochemistry -
 -------------------

 Zero-point correction=                           0.224807 (Hartree/Particle)
 Thermal correction to Energy=                    0.239527
 Thermal correction to Enthalpy=                  0.240471
 Thermal correction to Gibbs Free Energy=         0.182756
 Sum of electronic and zero-point Energies=           -931.389981
 Sum of electronic and thermal Energies=              -931.375261
 Sum of electronic and thermal Enthalpies=            -931.374317
 Sum of electronic and thermal Free Energies=         -931.432032

                    E (Thermal)             CV                S
                     KCal/Mol        Cal/Mol-Kelvin    Cal/Mol-Kelvin
 Total                 150.305             55.132            121.471

8674,-0.7014998,-4.5898138|PG=C01 [X(C9H14N3O1P1)]|NImag=0||0.64680985
```

Intermediate Int2.2

opt freq PBE0-D3(BJ)/def2-TZVP SMD(THF)

0 1

C	-3.14733400	0.73746700	-0.90495400
N	-1.86440200	0.49701000	-1.16001400
C	-1.20591400	-0.33269300	-0.35144800
C	-1.81142600	-0.95384900	0.73642700
C	-3.14537200	-0.69318200	0.99441300
C	-3.83292000	0.17377200	0.15876100
C	0.24284900	-0.54716700	-0.65741900
O	2.58957000	-1.04941500	-0.53787600
N	1.86622600	-2.10263400	-0.37500300
N	0.62356300	-1.92579500	-0.40264900
P	1.40049900	0.64370400	0.18150600
C	2.06197900	0.14674100	1.78488000
C	0.27780800	1.99352700	0.68116000
C	2.54243200	1.56318900	-0.86082200
H	-3.65336200	1.41410100	-1.58802000
H	-1.24005800	-1.63328900	1.35626800
H	-3.64323100	-1.16231600	1.83567500
H	-4.87878600	0.40493500	0.32037400
H	0.42122200	-0.29801800	-1.71312200
H	1.86907000	0.92136500	2.52738500
H	1.57071200	-0.77730300	2.09834800
H	3.13005000	-0.04451600	1.69986600
H	-0.21665500	2.42492300	-0.19123700
H	-0.48435900	1.63243000	1.37414300
H	0.86296800	2.77038800	1.17831300
H	3.49041500	1.04315100	-0.96779200
H	2.08269100	1.68404300	-1.84473300
H	2.69495000	2.55297500	-0.42737200

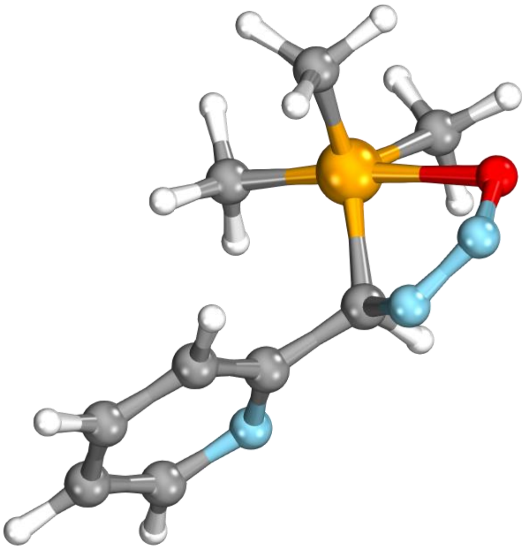

Fig. S22: Computed structure of **Int2.2.**

Item		Value	Threshold	Converged?
Maximum	Force	0.000002	0.000450	YES
RMS	Force	0.000000	0.000300	YES
Maximum	Displacement	0.000254	0.001800	YES
RMS	Displacement	0.000045	0.001200	YES

Predicted change in Energy=-1.633265D-10

Optimization completed.

-- Stationary point found.

SCF Done: E(RPBE1PBE) = -931.617389793 A.U. after 1 cycles

- Thermochemistry -

Zero-point correction= 0.224645 (Hartree/Particle)

Thermal correction to Energy= 0.239414

```
Thermal correction to Enthalpy=                  0.240358
Thermal correction to Gibbs Free Energy=         0.182586
Sum of electronic and zero-point Energies=           -931.392745
Sum of electronic and thermal Energies=              -931.377976
Sum of electronic and thermal Enthalpies=            -931.377032
Sum of electronic and thermal Free Energies=         -931.434803

                    E (Thermal)             CV                S
                     KCal/Mol        Cal/Mol-Kelvin    Cal/Mol-Kelvin
Total                 150.234            55.244           121.590

-0.3758297,-3.0077368\PG=C01 [X(C9H14N3O1P1)]\NImag=0\\0.66604003,-0.1
```

Transition state TS3

opt freq PBE0-D3(BJ)/def2-TZVP SMD(THF)

```
0 1
 C                 3.34177100   -0.57151600   -0.90115800
 N                 2.06168000   -0.54277600   -1.25647700
 C                 1.14099400   -0.16406300   -0.34743400
 C                 1.53567600    0.21814700    0.94834700
 C                 2.86593000    0.15965500    1.30244500
 C                 3.80583600   -0.24996900    0.36246800
 C                -0.23040200   -0.10314900   -0.81175700
 O                -1.22940100    2.39625500    0.78148200
 N                -0.89244000    2.54475400   -0.38656400
 N                -0.51811000    1.97140600   -1.32213000
 P                -1.57688200   -0.79525500    0.04638100
 C                -3.10702200   -0.05073300   -0.51805000
 C                -1.52505000   -0.69029800    1.83335700
 C                -1.74274000   -2.55528700   -0.31596000
 H                 4.04131800   -0.87483800   -1.67700000
 H                 0.80069000    0.59280800    1.64778700
 H                 3.17461700    0.45144000    2.30085400
 H                 4.86238900   -0.30047700    0.59470600
 H                -0.32000700   -0.39397400   -1.85774400
 H                -3.95614500   -0.62466300   -0.14139400
 H                -3.16925900    0.97639700   -0.15801600
 H                -3.12475900   -0.05578800   -1.60983100
 H                -0.66314400   -1.23981500    2.21422600
 H                -1.48041800    0.34976700    2.15133200
 H                -2.43674900   -1.15361500    2.21748400
 H                -1.85055500   -2.68921700   -1.39426000
 H                -0.84207100   -3.07543500    0.01641200
 H                -2.61533500   -2.97421100    0.19053000
```

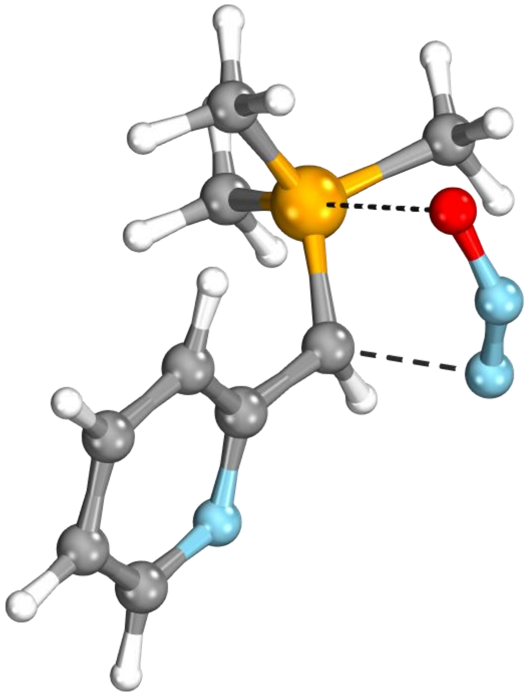

Fig. S23: Computed structure of **TS3**.

```
        Item               Value     Threshold  Converged?
```

```
 Maximum Force            0.000042     0.000450     YES
 RMS     Force            0.000005     0.000300     YES
 Maximum Displacement     0.001448     0.001800     YES
 RMS     Displacement     0.000455     0.001200     YES
 Predicted change in Energy=-1.071011D-08
 Optimization completed.
    -- Stationary point found.

SCF Done:  E(RPBE1PBE) =  -931.576343103     A.U. after    1 cycles

-------------------
 - Thermochemistry -
 -------------------


 Zero-point correction=                           0.221000 (Hartree/Particle)
 Thermal correction to Energy=                    0.236438
 Thermal correction to Enthalpy=                  0.237382
 Thermal correction to Gibbs Free Energy=         0.177933
 Sum of electronic and zero-point Energies=           -931.355343
 Sum of electronic and thermal Energies=              -931.339905
 Sum of electronic and thermal Enthalpies=            -931.338961
 Sum of electronic and thermal Free Energies=         -931.398410

                    E (Thermal)            CV                 S
                     KCal/Mol        Cal/Mol-Kelvin    Cal/Mol-Kelvin
 Total                 148.367             56.546            125.121

1P1)]\NImag=1\\0.70713138,-0.06425314,0.21509788,-0.02323894,0.1576951
```

Transition state TS4.1

\# opt freq PBE0-D3(BJ)/def2-TZVP SMD(THF)

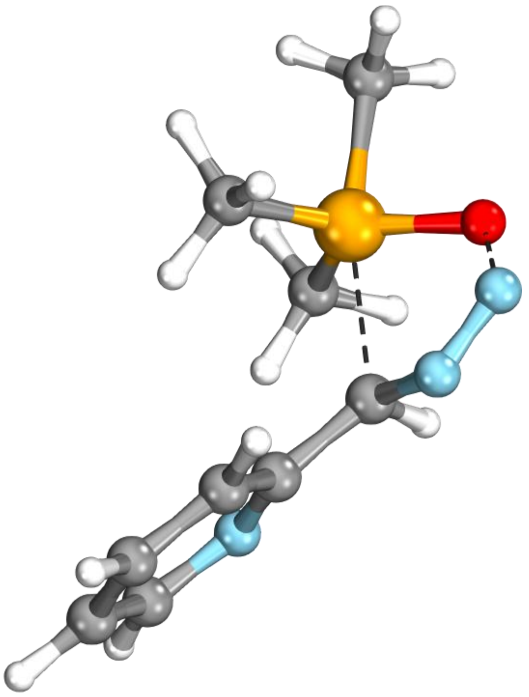

Fig. S24: Computed structure of **TS4.1.**

0 1

C	3.19005400	-1.23369000	-0.53479100
N	1.96176900	-0.87431500	-0.89437600
C	1.44554600	0.25334200	-0.37684400
C	2.17202700	1.03713000	0.53295900
C	3.45005600	0.65445600	0.88106700
C	3.98237400	-0.51116700	0.34065400
C	0.08102900	0.56687400	-0.74697300
O	-2.35517900	0.85904000	-0.74150800
N	-1.53927400	2.10795000	-0.59122700
N	-0.34386300	1.86565200	-0.57302700
P	-1.84063800	-0.40744500	0.16633800
C	-3.54385800	-0.90018100	0.59280800
C	-1.15612300	-0.10325300	1.80681600
C	-1.26635500	-1.92519600	-0.61342300
H	3.56504200	-2.15349400	-0.97732700
H	1.72394300	1.93215500	0.94712900
H	4.02954100	1.25506900	1.57418200
H	4.97943200	-0.85140900	0.59169000
H	-0.26717000	0.11593000	-1.67305300
H	-3.52757200	-1.79833300	1.21419000
H	-4.03881000	-0.09389100	1.13728900
H	-4.11011800	-1.10969100	-0.31773300
H	-0.11976000	-0.43577800	1.84699200
H	-1.18922000	0.97133500	2.00329300
H	-1.74977900	-0.61597600	2.56591500
H	-1.59535400	-1.90901800	-1.65636400
H	-0.18203400	-2.02074600	-0.58504200
H	-1.74605900	-2.77169500	-0.11738500

Item	Value	Threshold	Converged?
Maximum Force	0.000010	0.000450	YES
RMS Force	0.000003	0.000300	YES
Maximum Displacement	0.001724	0.001800	YES
RMS Displacement	0.000360	0.001200	YES

Predicted change in Energy=-1.060974D-08
Optimization completed.
-- Stationary point found

SCF Done: E(RPBE1PBE) = -931.592089350 A.U. after 1 cycles

- Thermochemistry -

Zero-point correction= 0.222769 (Hartree/Particle)
Thermal correction to Energy= 0.236850

```
Thermal correction to Enthalpy=                  0.237794
Thermal correction to Gibbs Free Energy=         0.182364
Sum of electronic and zero-point Energies=           -931.369321
Sum of electronic and thermal Energies=              -931.355239
Sum of electronic and thermal Enthalpies=            -931.354295
Sum of electronic and thermal Free Energies=         -931.409725

                    E (Thermal)             CV                S
                     KCal/Mol        Cal/Mol-Kelvin    Cal/Mol-Kelvin
Total                 148.626            54.581           116.662
```

]\NImag=1\\0.62566382,-0.12738174,0.57021625,0.12273306,0.25375664,0.4

Transition state TS4.2

opt freq PBE0-D3(BJ)/def2-TZVP SMD(THF)

```
0 1
 C                 3.19767600    0.62002600    1.05132500
 N                 1.96573100    0.92152100    0.65516400
 C                 1.44488300    0.24833300   -0.38333000
 C                 2.16599200   -0.76846400   -1.02712800
 C                 3.43702100   -1.07937500   -0.59011300
 C                 3.97741700   -0.36945800    0.47558000
 C                 0.08022700    0.54532800   -0.77096900
 O                -2.36468400    0.81526900   -0.77922400
 N                -1.53665900    2.09470300   -0.64500200
 N                -0.35063500    1.85082500   -0.61085500
 P                -1.83639800   -0.40861700    0.16215300
 C                -3.53420500   -0.92503300    0.59594200
 C                -1.19274300   -0.04644000    1.80619400
 C                -1.22515800   -1.94545400   -0.55642700
 H                 3.58323300    1.19900200    1.88733000
 H                 1.72506800   -1.28871700   -1.87010900
 H                 4.00857600   -1.85995000   -1.08076700
 H                 4.97246100   -0.57523200    0.85043600
 H                -0.24632000    0.12364500   -1.71917300
 H                -3.50685900   -1.80605500    1.24105400
 H                -4.04932300   -0.11426500    1.11443400
 H                -4.09056800   -1.16988200   -0.31212500
 H                -0.10640600   -0.10346200    1.83476100
 H                -1.49737900    0.96955700    2.07088600
 H                -1.63626800   -0.73751800    2.52611600
 H                -1.39572100   -1.91208400   -1.63536400
 H                -0.16256600   -2.08061100   -0.36379800
 H                -1.79020800   -2.78343700   -0.14376600
```

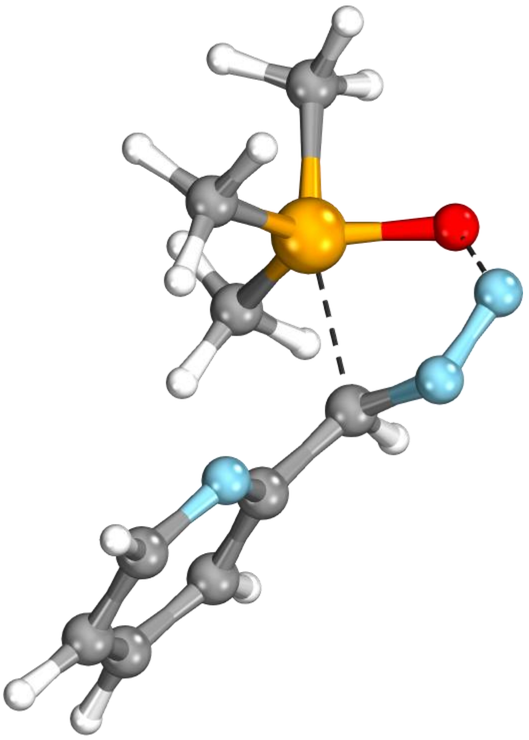

Fig. S25: Computed structure of **TS4.2**.

```
         Item               Value     Threshold  Converged?
```

```
 Maximum Force            0.000002     0.000450     YES
 RMS     Force            0.000000     0.000300     YES
 Maximum Displacement     0.000342     0.001800     YES
 RMS     Displacement     0.000052     0.001200     YES
 Predicted change in Energy=-2.962641D-10
 Optimization completed.
    -- Stationary point found.

SCF Done:  E(RPBE1PBE) =  -931.589471503     A.U. after    1 cycles

-------------------
 - Thermochemistry -
 -------------------

 Zero-point correction=                           0.222487 (Hartree/Particle)
 Thermal correction to Energy=                    0.236769
 Thermal correction to Enthalpy=                  0.237713
 Thermal correction to Gibbs Free Energy=         0.181580
 Sum of electronic and zero-point Energies=           -931.366984
 Sum of electronic and thermal Energies=              -931.352703
 Sum of electronic and thermal Enthalpies=            -931.351758
 Sum of electronic and thermal Free Energies=         -931.407891

                    E (Thermal)             CV                S
                     KCal/Mol        Cal/Mol-Kelvin    Cal/Mol-Kelvin
 Total                148.575             54.796           118.141

N3O1P1)]\NImag=1\\0.62439778,-0.11830574,0.43287471,0.13342053,0.25636
```

Trimethylphosphine oxide

opt freq PBE0-D3(BJ)/def2-TZVP SMD(THF)

0 1

P	0.00017800	-0.00011900	0.17471800
C	-0.97112700	1.33555600	-0.54363900
C	-0.67150000	-1.50838400	-0.54458800
C	1.64191500	0.17351200	-0.54499400
H	-0.57032600	2.29332100	-0.20362300
H	-2.00620900	1.24822800	-0.20518600
H	-0.94555900	1.30189700	-1.63504700
H	-1.70132400	-1.63973900	-0.20426000
H	-0.07851200	-2.36154000	-0.20680900
H	-0.65543500	-1.46880500	-1.63597800
H	1.59917900	0.16983400	-1.63637600
H	2.08395800	1.11310100	-0.20535600
H	2.27120300	-0.65300500	-0.20664600
O	0.00081900	-0.00080500	1.66493100

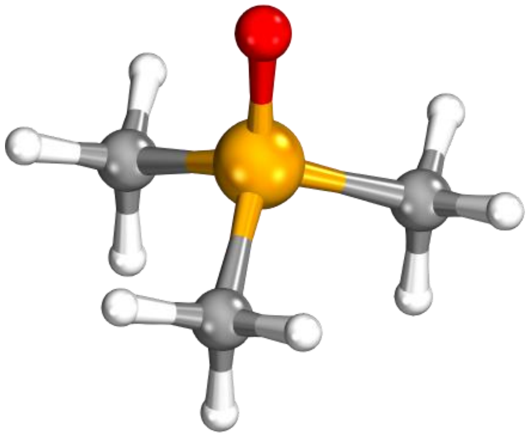

Fig. S26: Computed structure of **trimethylphosphine oxide.**

```
        Item               Value     Threshold  Converged?
 Maximum Force            0.000001     0.000450     YES
 RMS     Force            0.000000     0.000300     YES
 Maximum Displacement     0.000001     0.001800     YES
 RMS     Displacement     0.000000     0.001200     YES
 Predicted change in Energy=-1.477088D-12
 Optimization completed.
    -- Stationary point found.

SCF Done:  E(RPBE1PBE) =  -536.123554565     A.U. after    1 cycles

-------------------
 - Thermochemistry -
 -------------------

 Zero-point correction=                           0.117210 (Hartree/Particle)
 Thermal correction to Energy=                    0.124601
 Thermal correction to Enthalpy=                  0.125545
 Thermal correction to Gibbs Free Energy=         0.086879
 Sum of electronic and zero-point Energies=           -536.006344
 Sum of electronic and thermal Energies=              -535.998953
 Sum of electronic and thermal Enthalpies=            -535.998009
 Sum of electronic and thermal Free Energies=         -536.036675

                     E (Thermal)             CV                S
                      KCal/Mol        Cal/Mol-Kelvin    Cal/Mol-Kelvin
 Total                   78.188             26.523            81.379

9O1P1)]|NImag=0||0.41264613,-0.00005626,0.41245266,0.00008558,-0.00008
```

***s-trans*-Diazoalkane**

opt freq PBE0-D3(BJ)/def2-TZVP SMD(THF)

0 1

C	-2.12558100	-0.89783800	-0.00002900
N	-0.85998600	-1.30206500	-0.00001600
C	0.10017900	-0.36860300	-0.00000800
C	-0.19100300	1.00145400	-0.00002100
C	-1.51237200	1.39694300	-0.00003300
C	-2.51167200	0.43198400	-0.00003600
C	1.45103800	-0.88346000	0.00000900
N	3.34390100	0.62372100	0.00008900
N	2.46742900	-0.08540200	0.00005100
H	-2.87479300	-1.68522700	-0.00003700
H	0.60453200	1.73756900	-0.00002400
H	-1.76278900	2.45197900	-0.00004400
H	-3.56049700	0.70080500	-0.00004500
H	1.67059700	-1.94178300	-0.00001500

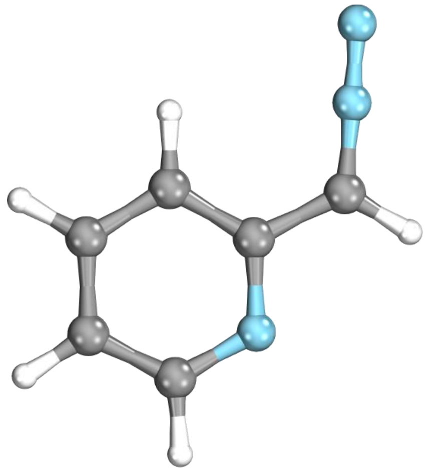

Fig. S27: Computed structure of ***s-trans*-2-(diazomethyl)pyridine**

Item	Value	Threshold	Converged?
Maximum Force	0.000173	0.000450	YES
RMS Force	0.000025	0.000300	YES
Maximum Displacement	0.000227	0.001800	YES
RMS Displacement	0.000071	0.001200	YES

Predicted change in Energy=-2.064682D-08
Optimization completed.
-- Stationary point found.

SCF Done: E(RPBE1PBE) = -395.546183569 A.U. after 1 cycles

- Thermochemistry -

Zero-point correction= 0.103346 (Hartree/Particle)
Thermal correction to Energy= 0.110468
Thermal correction to Enthalpy= 0.111412
Thermal correction to Gibbs Free Energy= 0.071326
Sum of electronic and zero-point Energies= -395.442838
Sum of electronic and thermal Energies= -395.435716
Sum of electronic and thermal Enthalpies= -395.434771
Sum of electronic and thermal Free Energies= -395.474858

	E (Thermal)	CV	S
	KCal/Mol	Cal/Mol-Kelvin	Cal/Mol-Kelvin
Total	69.320	26.642	84.369

983487,-0.0000714,-0.0000773|PG=C01 [X(C6H5N3)]|NImag=0||0.72400564,0.

***s-cis*-Diazoalkane**

opt freq PBE0-D3(BJ)/def2-TZVP SMD(THF)

0 1

C	-1.31144200	-1.39669500	-0.00005500
N	-0.06906300	-0.92702700	-0.00001300
C	0.10144800	0.40042300	-0.00000400
C	-0.97608800	1.29413700	-0.00003800
C	-2.25769100	0.78369300	-0.00008200
C	-2.44028000	-0.59372200	-0.00008700
C	1.45316000	0.90934900	0.00009400
N	3.28623100	-0.66926800	0.00011700
N	2.44287800	0.07566700	0.00008900
H	-1.41026100	-2.47902600	-0.00008100
H	-0.79497400	2.36252200	-0.00004600
H	-3.11018700	1.45401000	-0.00010500
H	-3.42901800	-1.03499500	-0.00011500
H	1.70947100	1.95877500	0.00002500

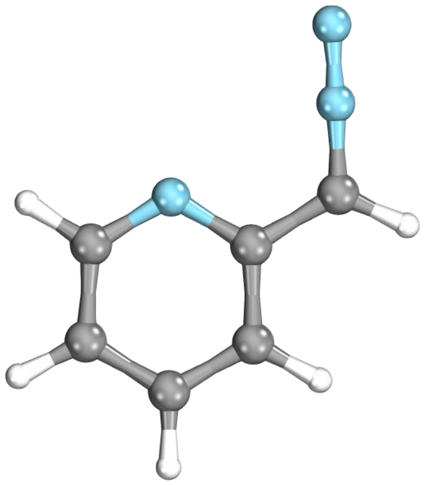

Fig. **S28**: Computed structure of ***s-cis*-2-(diazomethyl)pyridine**

Item	Value	Threshold	Converged?
Maximum Force	0.000178	0.000450	YES
RMS Force	0.000025	0.000300	YES
Maximum Displacement	0.000154	0.001800	YES
RMS Displacement	0.000049	0.001200	YES

Predicted change in Energy=-2.023286D-08
Optimization completed.
-- Stationary point found.

SCF Done: E(RPBE1PBE) = -395.547434465 A.U. after 1 cycles

- Thermochemistry -

Zero-point correction=	0.103420 (Hartree/Particle)
Thermal correction to Energy=	0.110537
Thermal correction to Enthalpy=	0.111482
Thermal correction to Gibbs Free Energy=	0.071509
Sum of electronic and zero-point Energies=	-395.444014
Sum of electronic and thermal Energies=	-395.436897
Sum of electronic and thermal Enthalpies=	-395.435953
Sum of electronic and thermal Free Energies=	-395.475926

	E (Thermal)	CV	S
	KCal/Mol	Cal/Mol-Kelvin	Cal/Mol-Kelvin
Total	69.363	26.634	84.130

.0000154,0.000005|PG=C01 [X(C6H5N3)]|NImag=0||0.68262894,-0.00868971,0

Transition state TS5

opt freq PBE0-D3(BJ)/def2-TZVP SMD(THF)

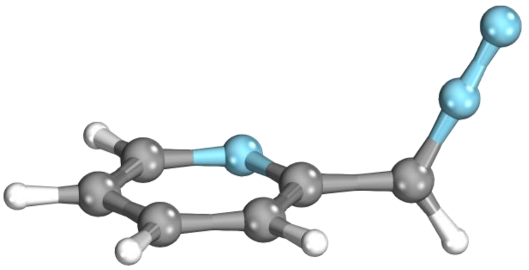

Fig. S29: Computed structure of **TS5**.

```
0 1
 C              -1.74189300   -1.21693000   -0.11474400
 N              -0.46158200   -1.17799200    0.24094700
 C               0.11523900    0.01620500    0.38414500
 C              -0.57246500    1.20724500    0.17722300
 C              -1.90667800    1.15527800   -0.19382900
 C              -2.50685600   -0.08297700   -0.34378300
 C               1.54025300    0.03743800    0.80240700
 N               3.26082600   -0.03949200   -0.89110600
 N               2.46107600   -0.00699400   -0.09121600
 H              -2.17983900   -2.20525300   -0.22304000
 H              -0.06211000    2.15385000    0.30642800
 H              -2.46572900    2.06856200   -0.36370100
 H              -3.54692700   -0.17545100   -0.63237600
 H               1.86677800    0.03208400    1.83380500

         Item               Value     Threshold  Converged?
 Maximum Force            0.000009     0.000450     YES
 RMS     Force            0.000001     0.000300     YES
 Maximum Displacement     0.000178     0.001800     YES
 RMS     Displacement     0.000045     0.001200     YES
 Predicted change in Energy=-1.463003D-10
 Optimization completed.
    -- Stationary point found.

SCF Done:  E(RPBE1PBE) =  -395.531444519     A.U. after    1 cycles

-------------------
 - Thermochemistry -
 -------------------

 Zero-point correction=                           0.102728 (Hartree/Particle)
 Thermal correction to Energy=                    0.109303
 Thermal correction to Enthalpy=                  0.110248
 Thermal correction to Gibbs Free Energy=         0.071410
 Sum of electronic and zero-point Energies=           -395.428717
 Sum of electronic and thermal Energies=              -395.422141
 Sum of electronic and thermal Enthalpies=            -395.421197
 Sum of electronic and thermal Free Energies=         -395.460035

                     E (Thermal)             CV                S
                      KCal/Mol        Cal/Mol-Kelvin    Cal/Mol-Kelvin
 Total                   68.589            24.986            81.741

444262,5.7876785,0.6603209|PG=C01 [X(C6H5N3)]|NImag=1||0.65600020,0.03
```

***s-cis P*-Ylide 2**

opt freq PBE0-D3(BJ)/def2-TZVP SMD(THF)

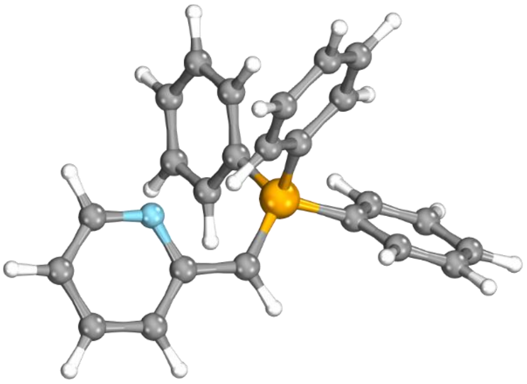

Fig. S30: Computed structure of ***s-cis-P*-Ylide 2**

0 1

C	3.94134400	0.38767200	0.03213400
N	2.63173800	0.30906800	-0.17162800
C	2.17102500	0.10763700	-1.42944000
C	3.08272000	-0.00395300	-2.50765500
C	4.43263500	0.08827100	-2.26908500
C	4.89414900	0.28716300	-0.96603300
C	0.76942200	0.00730200	-1.61271300
P	-0.30836600	-0.01264400	-0.29702200
H	4.24907200	0.54614900	1.06410000
H	2.70412300	-0.16373700	-3.51121000
H	5.13498000	0.00398500	-3.09255100
H	5.94980900	0.36339900	-0.73767200
H	0.34251600	-0.15494400	-2.59315200
C	-1.92956100	-0.45180600	-0.95912200
C	-2.60744600	-1.59758100	-0.55707500
C	-2.50358600	0.39588900	-1.90725600
C	-3.84539700	-1.89978200	-1.10756700
H	-2.17240500	-2.25681000	0.18454800
C	-3.73664700	0.08703800	-2.45695400
H	-1.98615600	1.29962100	-2.21087500
C	-4.40884900	-1.06146700	-2.05767200
H	-4.36929800	-2.79452200	-0.79087900
H	-4.17699000	0.74738400	-3.19544200
H	-5.37576100	-1.29987400	-2.48654100
C	0.10331500	-1.23006800	0.97999400
C	-0.05632800	-0.98526700	2.33961600
C	0.59403900	-2.46244200	0.55323100
C	0.26463900	-1.96931700	3.26357300
H	-0.41656100	-0.02384500	2.68424000
C	0.91015800	-3.44374600	1.47813700
H	0.73693100	-2.64120900	-0.50723300
C	0.74474700	-3.19838600	2.83519600
H	0.14391200	-1.77082100	4.32262000
H	1.29423300	-4.39945400	1.13970700
H	0.99806200	-3.96438900	3.55963600
C	-0.58788900	1.55901800	0.56451900
C	0.33756800	2.58986300	0.43562300
C	-1.74597800	1.75907900	1.31457300
C	0.11515700	3.80437400	1.06716000
H	1.22966400	2.43485300	-0.15831600
C	-1.96580800	2.97517400	1.94266500
H	-2.48347200	0.96825500	1.40147400
C	-1.03378600	3.99731200	1.82180900
H	0.84045800	4.60385600	0.96563000
H	-2.86993500	3.12720700	2.52141400
H	-1.20771700	4.94921600	2.31127500

```
        Item               Value     Threshold  Converged?
 Maximum Force            0.000004     0.000450     YES
 RMS     Force            0.000001     0.000300     YES
 Maximum Displacement     0.000589     0.001800     YES
 RMS     Displacement     0.000127     0.001200     YES
 Predicted change in Energy=-4.605890D-10
 Optimization completed.
    -- Stationary point found.

SCF Done:  E(RPBE1PBE) =  -1321.82210694     A.U. after    1 cycles

-------------------
 - Thermochemistry -
 -------------------

 Zero-point correction=                           0.370589 (Hartree/Particle)
 Thermal correction to Energy=                    0.392554
 Thermal correction to Enthalpy=                  0.393498
 Thermal correction to Gibbs Free Energy=         0.316829
 Sum of electronic and zero-point Energies=          -1321.451518
 Sum of electronic and thermal Energies=             -1321.429553
 Sum of electronic and thermal Enthalpies=           -1321.428609
 Sum of electronic and thermal Free Energies=        -1321.505278

                     E (Thermal)             CV                S
                      KCal/Mol        Cal/Mol-Kelvin    Cal/Mol-Kelvin
 Total                  246.331             86.546            161.364

0.0349592,-0.3688568,2.3682463,0.0440404|PG=C01 [X(C24H20N1P1)]|NImag=
0||0.68727234,0.02406837,0.17584683,0.01357979,0.08950765,0.74284519,-
```

s-*trans* *P*-Ylide 2

opt freq PBE0-D3(BJ)/def2-TZVP SMD(THF)

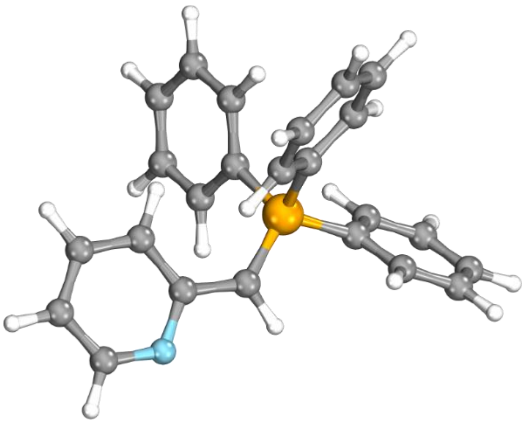

Fig. S31: Computed structure of **s-trans P-Ylide 2**

0 1

C	4.31874800	0.14883700	-2.36742500
N	2.99720800	0.07255300	-2.46523500
C	2.24926200	0.12219500	-1.33500600
C	2.89030000	0.25668800	-0.07756600
C	4.26130800	0.32783500	-0.00741300
C	5.01607400	0.27396700	-1.17798400
C	0.83964500	0.02572000	-1.49595200
P	-0.33126100	-0.01266000	-0.26901900
H	4.86235500	0.10638800	-3.30981000
H	2.30265100	0.30709300	0.83186400
H	4.74729100	0.42966000	0.95781600
H	6.09737900	0.32974500	-1.16818000
H	0.44877200	-0.06210000	-2.50199100
C	-1.89648600	-0.50108400	-1.01596100
C	-2.55718300	-1.66613900	-0.64184300
C	-2.44416400	0.32530000	-1.99748400
C	-3.75407400	-2.00906900	-1.25494000
H	-2.14153700	-2.30769900	0.12597100
C	-3.63575800	-0.02561700	-2.60962000
H	-1.93955500	1.24311100	-2.27956300
C	-4.29160500	-1.19290500	-2.23889300
H	-4.26623300	-2.91819300	-0.96099400
H	-4.05653100	0.61655500	-3.37494300
H	-5.22626900	-1.46366000	-2.71714600
C	0.05798800	-1.19142400	1.05092900
C	-0.15577400	-0.92470700	2.39994300
C	0.61079300	-2.41286900	0.66734200
C	0.16972900	-1.87693700	3.35487100
H	-0.56364200	0.02872400	2.71218700
C	0.92934800	-3.36281800	1.62346100
H	0.79850800	-2.60631700	-0.38339300
C	0.70845300	-3.09562300	2.96835900
H	0.00580200	-1.66224000	4.40479700
H	1.35982500	-4.30997700	1.31897500
H	0.96512500	-3.83646000	3.71731700
C	-0.68525500	1.56232900	0.56098100
C	0.22219500	2.60940500	0.43608800
C	-1.86286500	1.74874300	1.28389400
C	-0.03324400	3.82671500	1.05040700
H	1.12047700	2.47257800	-0.15560400
C	-2.11856000	2.96860000	1.88965200
H	-2.58617500	0.94422100	1.36496600
C	-1.20139500	4.00594500	1.77772000
H	0.67791200	4.63910000	0.95233200
H	-3.03860300	3.11275000	2.44481300
H	-1.40398500	4.96022100	2.25115200

```
        Item               Value     Threshold  Converged?
 Maximum Force            0.000001     0.000450     YES
 RMS     Force            0.000000     0.000300     YES
 Maximum Displacement     0.000326     0.001800     YES
 RMS     Displacement     0.000086     0.001200     YES
 Predicted change in Energy=-9.841039D-11
 Optimization completed.
    -- Stationary point found.

SCF Done:  E(RPBE1PBE) =  -1321.81861157     A.U. after    1 cycles

-------------------
 - Thermochemistry -
 -------------------

 Zero-point correction=                           0.370641 (Hartree/Particle)
 Thermal correction to Energy=                    0.392586
 Thermal correction to Enthalpy=                  0.393530
 Thermal correction to Gibbs Free Energy=         0.316744
 Sum of electronic and zero-point Energies=          -1321.447971
 Sum of electronic and thermal Energies=             -1321.426026
 Sum of electronic and thermal Enthalpies=           -1321.425082
 Sum of electronic and thermal Free Energies=        -1321.501868

                     E (Thermal)             CV                S
                      KCal/Mol        Cal/Mol-Kelvin    Cal/Mol-Kelvin
 Total                  246.351             86.504           161.610

59792,0.0814005,10.6981204,-0.7252809\PG=C01 [X(C24H20N1P1)]\NImag=0\\
```

Transition state TS6

opt freq PBE0-D3(BJ)/def2-TZVP SMD(THF)

0 1

C	-3.91487700	1.09541900	-1.32769100
N	-2.59739100	0.97322600	-1.46395800
C	-2.06084000	-0.25688700	-1.53829300
C	-2.87816200	-1.38979900	-1.47420300
C	-4.24809100	-1.25390600	-1.33139000
C	-4.78621400	0.02066000	-1.25397900
C	-0.59472500	-0.40659800	-1.66630600
P	0.40835200	-0.07189900	-0.36146100
H	-4.29735200	2.11189000	-1.27000800
H	-2.41834200	-2.36911600	-1.52689100
H	-4.88541000	-2.13003400	-1.27532900
H	-5.85124400	0.18292500	-1.13790400
H	-0.14954000	-0.27558300	-2.64969900
C	2.06562200	-0.70573400	-0.71465600
C	3.21266500	-0.06282800	-0.25442400
C	2.18934700	-1.88304000	-1.45202000
C	4.46516200	-0.59201000	-0.52650300
H	3.13283200	0.85872400	0.30885700
C	3.44336900	-2.40685200	-1.72491000
H	1.29415700	-2.37796600	-1.80951500
C	4.58259500	-1.76361400	-1.26081400
H	5.35237900	-0.08258600	-0.16766000
H	3.53026700	-3.32087600	-2.30158800
H	5.56328900	-2.17417800	-1.47441200
C	0.68216500	1.65074300	0.19126100
C	1.05854200	1.97656100	1.49333500
C	0.50751300	2.66464500	-0.74438600
C	1.26641700	3.30075200	1.84842400
H	1.18834500	1.19466500	2.23379100
C	0.71943500	3.98904000	-0.39042100
H	0.18504400	2.40855200	-1.74860600
C	1.09910200	4.30755300	0.90609500
H	1.55626400	3.54857100	2.86361600
H	0.57880500	4.77425200	-1.12494400
H	1.25744000	5.34322400	1.18613700
C	-0.24358900	-0.86874700	1.12754900
C	-1.35924700	-0.31635000	1.75610600
C	0.28829500	-2.06565500	1.59795000
C	-1.94050400	-0.96326500	2.83560900
H	-1.77379500	0.62134600	1.40293100
C	-0.28810600	-2.70276200	2.68702600
H	1.15600600	-2.50191800	1.11595300
C	-1.40442700	-2.15520300	3.30387100
H	-2.81229700	-0.53193700	3.31466000
H	0.13677600	-3.63037200	3.05398700
H	-1.85584500	-2.65642300	4.15287900

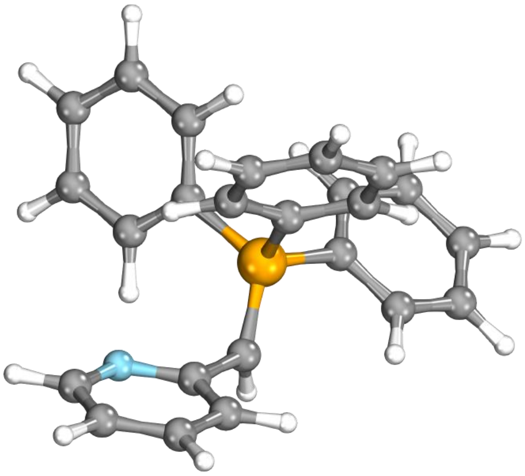

Fig. S32: Computed structure of **TS6**.

```
        Item               Value     Threshold  Converged?
 Maximum Force            0.000007     0.000450     YES
 RMS     Force            0.000001     0.000300     YES
 Maximum Displacement     0.000928     0.001800     YES
 RMS     Displacement     0.000198     0.001200     YES
 Predicted change in Energy=-8.026117D-10
 Optimization completed.
    -- Stationary point found.

SCF Done:  E(RPBE1PBE) =  -1321.79997878     A.U. after    1 cycles

-------------------
 - Thermochemistry -
 -------------------

 Zero-point correction=                           0.369703 (Hartree/Particle)
 Thermal correction to Energy=                    0.391156
 Thermal correction to Enthalpy=                  0.392100
 Thermal correction to Gibbs Free Energy=         0.316742
 Sum of electronic and zero-point Energies=          -1321.430276
 Sum of electronic and thermal Energies=             -1321.408823
 Sum of electronic and thermal Enthalpies=           -1321.407878
 Sum of electronic and thermal Free Energies=        -1321.483237

                     E (Thermal)             CV                S
                      KCal/Mol        Cal/Mol-Kelvin    Cal/Mol-Kelvin
 Total                  245.454             84.914           158.606

G=C01 [X(C24H20N1P1)]|NImag=1||0.68183463,-0.02675863,0.75625195,-0.04
```

Transition state TS7.1

\# opt freq PBE0-D3(BJ)/def2-TZVP SMD(THF)

0 1

C	-3.87916100	-0.02958400	0.61590200
N	-2.56780400	-0.16168000	0.44964100
C	-2.11494100	-1.00345200	-0.49373100
C	-3.00182400	-1.74742800	-1.28981800
C	-4.35878100	-1.59726000	-1.10344800
C	-4.82128100	-0.71643600	-0.12962900
C	-0.68587200	-1.13493700	-0.65020300
O	0.19491800	-3.80404300	-1.51893700
N	0.07723900	-3.64264300	-0.30505200
N	-0.19687700	-2.78783200	0.45600100
P	0.35838500	0.12960800	-0.05025000
H	-4.19287100	0.66585800	1.39055900
H	-2.61019900	-2.43046800	-2.03463300
H	-5.05748300	-2.16221800	-1.71126800
H	-5.87936000	-0.57008100	0.04847200
H	-0.33195600	-1.54663000	-1.59215000
C	1.92806700	-0.06578400	-0.91179500
C	2.65501200	-1.24136200	-0.71832200
C	2.41088000	0.91565400	-1.77182100
C	3.84850600	-1.43290300	-1.39327200
H	2.29453400	-1.99952800	-0.03324800
C	3.60943900	0.71793700	-2.44214300
H	1.85590500	1.83400100	-1.92036100
C	4.32598700	-0.45471500	-2.25618300
H	4.40812000	-2.34887500	-1.24288900
H	3.98227800	1.48493600	-3.11123200
H	5.26178800	-0.60728800	-2.78208600
C	-0.26394800	1.78275800	-0.41553400
C	-0.09277300	2.83984900	0.47185200
C	-0.91080100	1.99086400	-1.63227700
C	-0.56572400	4.10095800	0.14052400
H	0.39900700	2.68093600	1.42401400
C	-1.37951400	3.25234700	-1.95816500
H	-1.05288500	1.16302900	-2.31849700
C	-1.20759600	4.30795800	-1.07182600
H	-0.43583800	4.92240900	0.83581700
H	-1.88578700	3.41084200	-2.90345800
H	-1.58011000	5.29398300	-1.32593700
C	0.74228700	0.06618700	1.70973000
C	-0.19459200	-0.39399900	2.63192900
C	1.99104600	0.50613900	2.14720100
C	0.11986000	-0.41106600	3.98113100
H	-1.16407200	-0.72841000	2.28804900
C	2.29644800	0.49053900	3.49919200
H	2.72700000	0.85936000	1.43406400
C	1.36193100	0.03104900	4.41614200

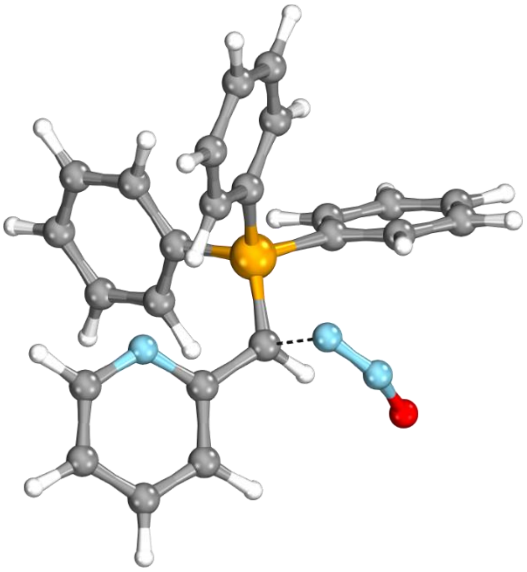

Fig. S33: Computed structure of **TS7.1**.

```
 H              -0.60918800   -0.77547500    4.69582200
 H               3.26851000    0.83365900    3.83442400
 H               1.60365900    0.01342300    5.47303500

         Item               Value     Threshold  Converged?
 Maximum Force            0.000018     0.000450     YES
 RMS     Force            0.000001     0.000300     YES
 Maximum Displacement     0.000749     0.001800     YES
 RMS     Displacement     0.000164     0.001200     YES
 Predicted change in Energy=-1.922601D-09
 Optimization completed.
    -- Stationary point found.

SCF Done:  E(RPBE1PBE) =  -1506.34057795     A.U. after    1 cycles

-------------------
 - Thermochemistry -
 -------------------

 Zero-point correction=                           0.382561 (Hartree/Particle)
 Thermal correction to Energy=                    0.407604
 Thermal correction to Enthalpy=                  0.408549
 Thermal correction to Gibbs Free Energy=         0.324155
 Sum of electronic and zero-point Energies=          -1505.958017
 Sum of electronic and thermal Energies=             -1505.932973
 Sum of electronic and thermal Enthalpies=           -1505.932029
 Sum of electronic and thermal Free Energies=        -1506.016423

                     E (Thermal)             CV                S
                      KCal/Mol        Cal/Mol-Kelvin    Cal/Mol-Kelvin
 Total                  255.776            95.689            177.621

0066,1.8765493,-3.9734979\PG=C01 [X(C24H20N3O1P1)]\NImag=1\\0.68794992
```

Transition state TS7.2

opt freq PBE0-D3(BJ)/def2-TZVP SMD(THF)

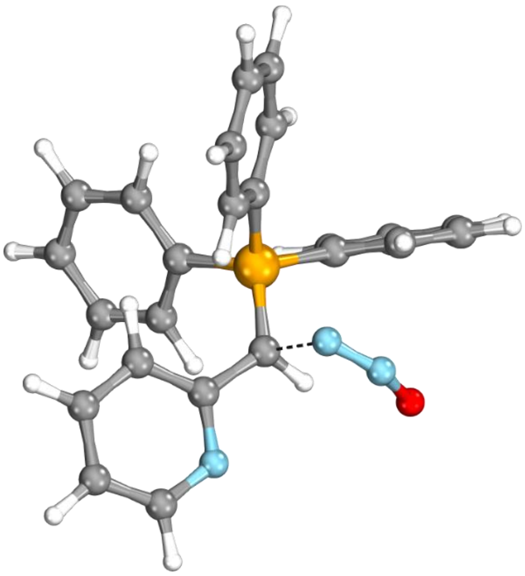

Fig. S34: Computed structure of **TS7.2**.

0 1

C	-4.23188300	-1.84888800	-0.94275100
N	-2.91024500	-1.87404800	-1.05235100
C	-2.17911400	-1.03751500	-0.29225200
C	-2.80319400	-0.14758300	0.59653500
C	-4.17978700	-0.13273000	0.69242900
C	-4.92511600	-1.00348400	-0.09065900
C	-0.74096000	-1.15178300	-0.45431500
O	0.47745400	-3.70979500	-1.23567400
N	0.20498200	-3.57770600	-0.04347400
N	-0.22687600	-2.74819300	0.67630800
P	0.36558900	0.13713100	-0.05304100
H	-4.77365000	-2.54621300	-1.57764400
H	-2.21727700	0.53406700	1.20104400
H	-4.66804600	0.55503200	1.37443400
H	-6.00685300	-1.02815000	-0.04602400
H	-0.43769200	-1.63150700	-1.38437400
C	1.86537900	-0.11834600	-1.01088600
C	2.68113500	-1.20393400	-0.69185400
C	2.20661100	0.72666400	-2.06220700
C	3.82638300	-1.44344600	-1.43214800
H	2.42799100	-1.85350300	0.13777700
C	3.35719800	0.48161000	-2.79670800
H	1.58130300	1.57640400	-2.30767000
C	4.16436400	-0.60236800	-2.48428500
H	4.45674400	-2.28985400	-1.18547200
H	3.62219000	1.14190000	-3.61438100
H	5.06311500	-0.79201200	-3.06032600
C	-0.31146500	1.75730900	-0.47344400
C	-0.14462600	2.86959700	0.34616800
C	-1.03464100	1.86989100	-1.66121300
C	-0.68944100	4.08912600	-0.02699100
H	0.39851300	2.78712200	1.27953200
C	-1.57127200	3.09164300	-2.03059600
H	-1.18247500	0.99857600	-2.28991400
C	-1.39916700	4.20152700	-1.21373600
H	-0.56201700	4.95254600	0.61580700
H	-2.13267700	3.17495700	-2.95393100
H	-1.82675500	5.15573100	-1.50036300
C	0.88388900	0.20253800	1.67101400
C	0.12877300	-0.39219100	2.67812900
C	2.06339100	0.87579700	1.99123100
C	0.54549500	-0.30047500	3.99721300
H	-0.76874200	-0.94403400	2.43348400
C	2.47349100	0.96391600	3.31118100
H	2.66488400	1.32602800	1.20904200
C	1.71363300	0.37747400	4.31454700

```
 H               -0.04157600   -0.77034700    4.77777800
 H                3.39127200    1.48626200    3.55556400
 H                2.03879500    0.44190600    5.34685100

         Item               Value     Threshold  Converged?
 Maximum Force            0.000002     0.000450     YES
 RMS     Force            0.000000     0.000300     YES
 Maximum Displacement     0.001193     0.001800     YES
 RMS     Displacement     0.000305     0.001200     YES
 Predicted change in Energy=-1.677803D-09
 Optimization completed.
    -- Stationary point found.

SCF Done:  E(RPBE1PBE) =  -1506.33464513     A.U. after    1 cycles

-------------------
 - Thermochemistry -
 -------------------

Zero-point correction=                           0.383006 (Hartree/Particle)
 Thermal correction to Energy=                    0.407817
 Thermal correction to Enthalpy=                  0.408761
 Thermal correction to Gibbs Free Energy=         0.325756
 Sum of electronic and zero-point Energies=          -1505.951639
 Sum of electronic and thermal Energies=             -1505.926828
 Sum of electronic and thermal Enthalpies=           -1505.925884
 Sum of electronic and thermal Free Energies=        -1506.008889

                   E (Thermal)             CV                S
                    KCal/Mol        Cal/Mol-Kelvin    Cal/Mol-Kelvin
 Total                255.909             95.475            174.700

,7.0730133,-3.6752859,-1.2547212,-6.6487147\PG=C01 [X(C24H20N3O1P1)]\N
 Imag=1\\0.70217777,0.04877438,0.46809912,0.00299570,0.29096491,0.44166
```

Intermediate Int3.1

opt freq PBE0-D3(BJ)/def2-TZVP SMD(THF)

0 1

C	-3.81147300	-0.26064500	0.31534100
N	-2.49870300	-0.24859500	0.09807000
C	-2.04630400	-0.62592700	-1.09398600
C	-2.89335300	-1.04903300	-2.11579800
C	-4.25525600	-1.05902900	-1.88172600
C	-4.73076500	-0.65416500	-0.64085900
C	-0.56910500	-0.59569100	-1.32510700
O	0.68287600	-0.97002200	-3.51407000
N	0.58090800	-1.98313600	-2.78560600
N	-0.02181200	-1.92175100	-1.65012600
P	0.35839400	0.09321900	0.07562600
H	-4.13749700	0.05533500	1.30223300
H	-2.48117000	-1.36136000	-3.06788000
H	-4.94130500	-1.37950500	-2.65780100
H	-5.79054700	-0.64856700	-0.41726200
H	-0.32519200	0.06872300	-2.17295500
C	2.06483300	0.25940100	-0.47039400
C	2.83277300	-0.89767700	-0.60904000
C	2.61661300	1.50184700	-0.77001500
C	4.13721400	-0.80592700	-1.06289300
H	2.40909100	-1.86487400	-0.36900100
C	3.92611400	1.58469100	-1.21789300
H	2.03437600	2.40664400	-0.64919100
C	4.68420100	0.43318800	-1.36868000
H	4.72876500	-1.70685600	-1.17678400
H	4.35332900	2.55390900	-1.44789600
H	5.70710300	0.50072800	-1.72174400
C	-0.25864600	1.72612800	0.50329400
C	-0.44949900	2.10248400	1.82894200
C	-0.55858800	2.61833300	-0.52591700
C	-0.93351100	3.36739500	2.12264900
H	-0.23833000	1.40762900	2.63210600
C	-1.04284800	3.88045300	-0.22349200
H	-0.42085700	2.33244600	-1.56256600
C	-1.23001100	4.25646500	1.09943700
H	-1.08492500	3.65556200	3.15652500
H	-1.27769500	4.56998200	-1.02599700
H	-1.61214500	5.24375600	1.33293100
C	0.41000600	-0.98178300	1.50926600
C	-0.42650700	-2.08681000	1.63457700
C	1.34984900	-0.69881900	2.50142500
C	-0.33038700	-2.89412500	2.75697900
H	-1.13672500	-2.32710600	0.85655800
C	1.43040300	-1.50340400	3.62538800
H	2.02256400	0.14490400	2.39278800
C	0.58950600	-2.60065000	3.75340800

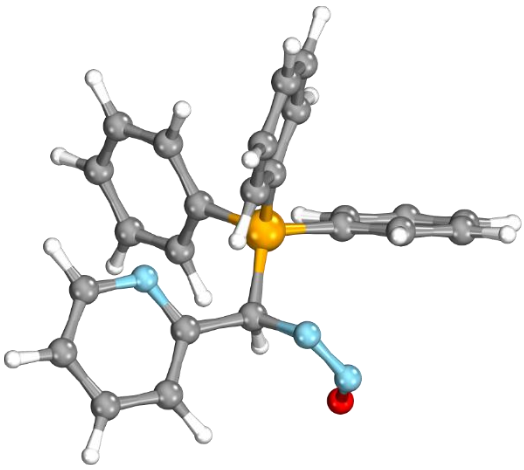

Fig. S35: Computed structure of **Int3.1.**

```
 H               -0.97670400   -3.75918000    2.84901700
 H                2.15707700   -1.27803700    4.39715500
 H                0.65833300   -3.23517000    4.62987800

         Item               Value     Threshold  Converged?
 Maximum Force            0.000001     0.000450     YES
 RMS     Force            0.000000     0.000300     YES
 Maximum Displacement     0.000059     0.001800     YES
 RMS     Displacement     0.000011     0.001200     YES
 Predicted change in Energy=-1.021649D-11
 Optimization completed.
    -- Stationary point found.

SCF Done:  E(RPBE1PBE) =  -1506.36441987     A.U. after    1 cycles

-------------------
 - Thermochemistry -
 -------------------

 Zero-point correction=                           0.385498 (Hartree/Particle)
 Thermal correction to Energy=                    0.410103
 Thermal correction to Enthalpy=                  0.411047
 Thermal correction to Gibbs Free Energy=         0.328352
 Sum of electronic and zero-point Energies=          -1505.978922
 Sum of electronic and thermal Energies=             -1505.954317
 Sum of electronic and thermal Enthalpies=           -1505.953373
 Sum of electronic and thermal Free Energies=        -1506.036067

                    E (Thermal)             CV                S
                     KCal/Mol        Cal/Mol-Kelvin    Cal/Mol-Kelvin
 Total                 257.344             95.171            174.046

[X(C24H20N3O1P1)]|NImag=0||0.68430645,0.02321117,0.22630877,-0.0347000
```

Intermediate Int3.2

opt freq PBE0-D3(BJ)/def2-TZVP SMD(THF)

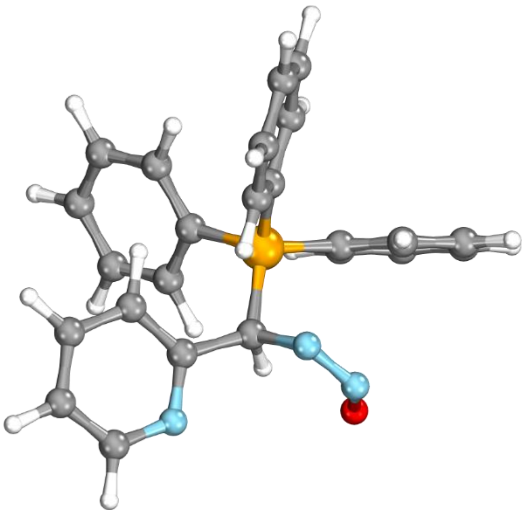

Fig. S36: Computed structure of **Int3.2.**

0 1

C	4.14341000	-1.72480200	-1.39294500
N	2.82177500	-1.82681500	-1.34057500
C	2.11592800	-0.78748100	-0.88836600
C	2.73060800	0.38698800	-0.46636800
C	4.11180000	0.48103400	-0.51772100
C	4.84119200	-0.59459500	-0.99289100
C	0.63228400	-1.02784600	-0.90444800
O	-0.68331600	-3.02976800	-1.79629400
N	-0.54386100	-2.08811900	-2.60676100
N	0.08361100	-1.01454300	-2.27440200
P	-0.40422400	0.05533200	0.11472100
H	4.67587300	-2.59379200	-1.77031700
H	2.15331900	1.22514700	-0.09804800
H	4.60779600	1.38811500	-0.19102000
H	5.92225700	-0.56572500	-1.05307300
H	0.43506600	-2.03222200	-0.49097900
C	-2.02790200	-0.69626400	0.24328000
C	-2.89337800	-0.59324300	-0.84548800
C	-2.41484400	-1.39226200	1.38464100
C	-4.13534500	-1.20153600	-0.79118300
H	-2.59441800	-0.04882900	-1.73286800
C	-3.66326000	-1.99238500	1.43170500
H	-1.75166900	-1.46430900	2.23772200
C	-4.52070000	-1.90027200	0.34508200
H	-4.80484600	-1.12858600	-1.64024500
H	-3.96525400	-2.53238000	2.32157400
H	-5.49564600	-2.37288700	0.38408700
C	0.32300700	0.19770200	1.75260400
C	0.42890100	1.42448600	2.40208300
C	0.82434800	-0.95718500	2.35628900
C	1.02220000	1.49240800	3.65297700
H	0.06400900	2.32864000	1.93119100
C	1.41290700	-0.88033700	3.60711300
H	0.76318500	-1.91412000	1.85022000
C	1.51141200	0.34295200	4.25620500
H	1.10646900	2.44955300	4.15433500
H	1.80160500	-1.77868800	4.07213400
H	1.97743500	0.40069400	5.23327700
C	-0.65467900	1.68095100	-0.59807600
C	0.07099600	2.11488100	-1.70442900
C	-1.61829900	2.51169700	-0.02218300
C	-0.15311500	3.38394000	-2.21554100
H	0.78266400	1.45723800	-2.18473200
C	-1.83125900	3.77883600	-0.53668600
H	-2.20736100	2.16701700	0.82079200
C	-1.09617600	4.21621000	-1.63093800

```
 H                0.40704000    3.71700400   -3.08138100
 H               -2.57784600    4.42290500   -0.08697700
 H               -1.26833900    5.20695700   -2.03619500

         Item               Value     Threshold  Converged?
 Maximum Force            0.000007     0.000450     YES
 RMS     Force            0.000001     0.000300     YES
 Maximum Displacement     0.000485     0.001800     YES
 RMS     Displacement     0.000117     0.001200     YES
 Predicted change in Energy=-5.224514D-10
 Optimization completed.
    -- Stationary point found.

SCF Done:  E(RPBE1PBE) =  -1506.35683672     A.U. after    1 cycles

-------------------
 - Thermochemistry -
 -------------------

 Zero-point correction=                           0.385589 (Hartree/Particle)
 Thermal correction to Energy=                    0.410144
 Thermal correction to Enthalpy=                  0.411088
 Thermal correction to Gibbs Free Energy=         0.328792
 Sum of electronic and zero-point Energies=          -1505.971248
 Sum of electronic and thermal Energies=             -1505.946693
 Sum of electronic and thermal Enthalpies=           -1505.945749
 Sum of electronic and thermal Free Energies=        -1506.028045

                    E (Thermal)             CV                S
                     KCal/Mol        Cal/Mol-Kelvin    Cal/Mol-Kelvin
 Total                 257.369             95.152            173.207

C24H20N3O1P1)]\NImag=0\\0.70515552,-0.02672979,0.66842799,-0.04733364,
```

Transition state TS8.1

opt freq PBE0-D3(BJ)/def2-TZVP SMD(THF)

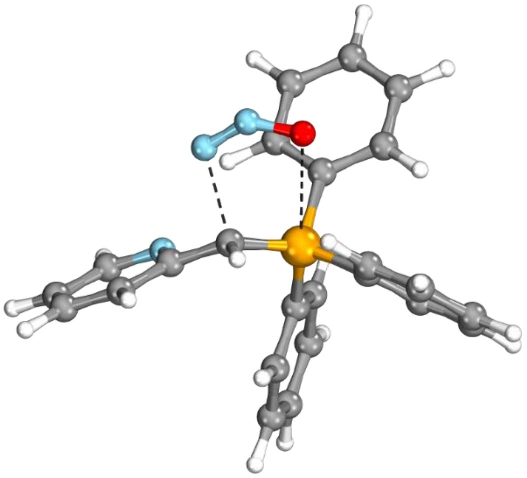

Fig. S37: Computed structure of **TS8.1.**

0 1

C	3.90645100	0.58391200	0.17950800
N	2.59249900	0.60927900	-0.01201600
C	2.11197200	0.37001000	-1.24167800
C	2.97324400	0.11678000	-2.32004000
C	4.33543000	0.10245000	-2.10550100
C	4.82593300	0.33604900	-0.82501600
C	0.67796900	0.42808700	-1.43266300
O	-1.67034300	2.21579400	-1.92632500
N	-0.57280400	2.76454700	-1.88204500
N	0.54611900	2.48863400	-1.69288800
P	-0.36158200	-0.08294100	-0.11830900
H	4.24290700	0.77600400	1.19550600
H	2.56136000	-0.06782100	-3.30550200
H	5.01446500	-0.09376100	-2.92844600
H	5.88778000	0.33041200	-0.61256600
H	0.31635000	0.09692500	-2.40018300
C	0.45943600	-1.29799300	0.94245200
C	1.06805700	-2.38797600	0.32287200
C	0.47202500	-1.19105100	2.32772300
C	1.69242900	-3.35821100	1.08658300
H	1.05797600	-2.47158200	-0.75889700
C	1.09905700	-2.16721600	3.08993600
H	-0.00055100	-0.34698200	2.81502200
C	1.71020700	-3.24752000	2.47206000
H	2.16936500	-4.20195600	0.60104300
H	1.11068200	-2.07885200	4.17034900
H	2.20270000	-4.00669000	3.06932200
C	-1.80481700	-0.95999900	-0.75734900
C	-2.45164700	-1.85750600	0.09254200
C	-2.26947000	-0.79016400	-2.05925500
C	-3.55306900	-2.57075000	-0.35356900
H	-2.09270100	-2.00709400	1.10425000
C	-3.36411800	-1.51466900	-2.50280900
H	-1.79694200	-0.07605500	-2.71876700
C	-4.00879700	-2.40270900	-1.65276000
H	-4.04941800	-3.26414400	0.31552700
H	-3.71774900	-1.37871400	-3.51844700
H	-4.86696600	-2.96453700	-2.00419900
C	-0.91462500	1.24227000	0.97145100
C	-2.26114800	1.40553000	1.28185300
C	0.03604000	2.11528500	1.49980200
C	-2.65472700	2.43562200	2.12220700
H	-3.00522700	0.74037000	0.86096000
C	-0.36601400	3.14272200	2.33750000
H	1.08208100	1.97952100	1.24687500
C	-1.70946600	3.30347400	2.64996600

```
 H                -3.70489700    2.56301200    2.35902400
 H                 0.37335200    3.82294600    2.74486400
 H                -2.02091000    4.11098400    3.30324300

         Item               Value     Threshold  Converged?
 Maximum Force            0.000004     0.000450     YES
 RMS     Force            0.000001     0.000300     YES
 Maximum Displacement     0.002961     0.001800     NO
 RMS     Displacement     0.000570     0.001200     YES
 Predicted change in Energy=-1.127329D-08
 Optimization completed on the basis of negligible forces.
    -- Stationary point found.

SCF Done:  E(RPBE1PBE) =  -1506.33873682     A.U. after    1 cycles

-------------------
 - Thermochemistry -
 -------------------

 Zero-point correction=                           0.382545 (Hartree/Particle)
 Thermal correction to Energy=                    0.407518
 Thermal correction to Enthalpy=                  0.408462
 Thermal correction to Gibbs Free Energy=         0.325347
 Sum of electronic and zero-point Energies=          -1505.956192
 Sum of electronic and thermal Energies=             -1505.931219
 Sum of electronic and thermal Enthalpies=           -1505.930275
 Sum of electronic and thermal Free Energies=        -1506.013390

                     E (Thermal)             CV                S
                      KCal/Mol        Cal/Mol-Kelvin    Cal/Mol-Kelvin
 Total                  255.721             95.881            174.930

G=C01 [X(C24H20N3O1P1)]\NImag=1\\0.68861037,-0.01969233,0.20010403,-0.
```

Transition state TS8.2

opt freq PBE0-D3(BJ)/def2-TZVP SMD(THF)

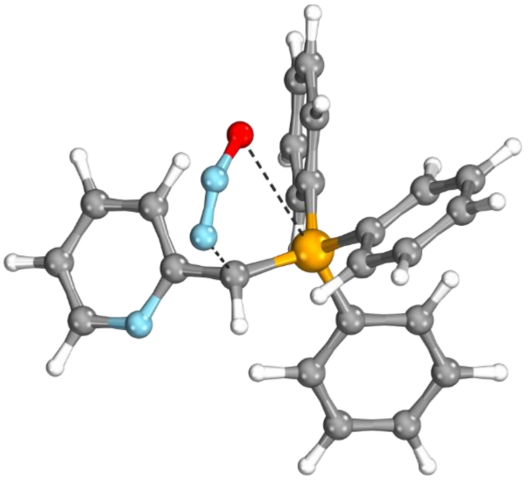

Fig. S38: Computed structure of **TS8.2**.

0 1

C	-4.15202800	0.42873400	-1.93242100
N	-2.82855200	0.39358800	-2.05673200
C	-2.11298200	-0.32281300	-1.17178600
C	-2.74834200	-1.03912100	-0.14462400
C	-4.11923700	-0.97068100	-0.01786800
C	-4.85153500	-0.21660900	-0.92805700
C	-0.67071800	-0.35954300	-1.39132700
O	-0.04657700	-3.12805400	-0.24192800
N	-0.18657000	-3.03541900	-1.45435400
N	-0.38258200	-2.24976700	-2.28781900
P	0.43708100	0.12024900	-0.13316200
H	-4.68330700	1.01336200	-2.67979700
H	-2.16075700	-1.65788700	0.52019700
H	-4.61841100	-1.51678400	0.77546400
H	-5.93066300	-0.14391400	-0.87128200
H	-0.39320900	0.16079900	-2.30617500
C	0.72055900	1.91390500	-0.22290000
C	-0.17827600	2.70397200	-0.93430700
C	1.79874800	2.50883500	0.43067700
C	-0.00127100	4.07962800	-0.98731100
H	-1.01695100	2.24580600	-1.44720000
C	1.97073600	3.88162400	0.37423400
H	2.50911700	1.90354400	0.98170800
C	1.07070000	4.66841400	-0.33419300
H	-0.70402500	4.68976600	-1.54324100
H	2.81208300	4.33966400	0.88182500
H	1.21003600	5.74275500	-0.37891500
C	2.06765200	-0.61209400	-0.34579800
C	2.94183900	-0.76182100	0.72896500
C	2.48237100	-0.95845200	-1.63031100
C	4.21401400	-1.27129400	0.51952300
H	2.63376600	-0.48812400	1.73089700
C	3.75613700	-1.46377000	-1.83259300
H	1.80972300	-0.84242900	-2.47127800
C	4.62128300	-1.62417000	-0.75889800
H	4.88732000	-1.39455400	1.36004700
H	4.07174900	-1.73640500	-2.83307900
H	5.61555400	-2.02568700	-0.91919100
C	-0.15871600	-0.17623200	1.53726700
C	0.09239400	-1.37262600	2.20647400
C	-0.93953100	0.80748500	2.14255200
C	-0.43867700	-1.57922500	3.46957100
H	0.68693500	-2.14292800	1.73577400
C	-1.47605600	0.58923400	3.40095500
H	-1.13514100	1.74206000	1.63025400
C	-1.22605900	-0.60337000	4.06495400

```
 H               -0.23957300   -2.51027600    3.98773700
 H               -2.08851500    1.35460600    3.86330400
 H               -1.64344900   -0.77154000    5.05147200

         Item               Value     Threshold  Converged?
 Maximum Force            0.000009     0.000450     YES
 RMS     Force            0.000001     0.000300     YES
 Maximum Displacement     0.000172     0.001800     YES
 RMS     Displacement     0.000032     0.001200     YES
 Predicted change in Energy=-1.609305D-10
 Optimization completed.
    -- Stationary point found.

SCF Done:  E(RPBE1PBE) =  -1506.33346603     A.U. after    1 cycles

-------------------
 - Thermochemistry -
 -------------------

 Zero-point correction=                           0.382947 (Hartree/Particle)
 Thermal correction to Energy=                    0.407842
 Thermal correction to Enthalpy=                  0.408787
 Thermal correction to Gibbs Free Energy=         0.325683
 Sum of electronic and zero-point Energies=          -1505.950519
 Sum of electronic and thermal Energies=             -1505.925624
 Sum of electronic and thermal Enthalpies=           -1505.924679
 Sum of electronic and thermal Free Energies=        -1506.007783

                     E (Thermal)             CV                S
                      KCal/Mol        Cal/Mol-Kelvin    Cal/Mol-Kelvin
 Total                  255.925             95.676            174.905

-6.4777133,-6.4218569\PG=C01 [X(C24H20N3O1P1)]\NImag=1\\0.69354392,-0.
```

Intermediate Int4.1

opt freq PBE0-D3(BJ)/def2-TZVP SMD(THF)

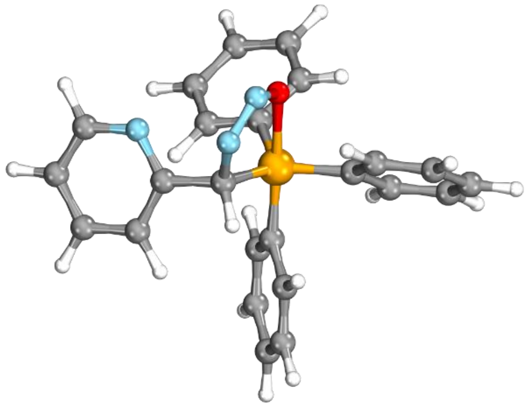

Fig. S39: Computed structure of **Int4.1.**

0 1

C	-4.01776600	-0.66897900	-1.20295800
N	-2.73454600	-0.71375000	-1.54314400
C	-2.01834400	0.40234100	-1.43965800
C	-2.55948100	1.60699500	-1.00140000
C	-3.89471100	1.63865800	-0.63712800
C	-4.64356700	0.47648300	-0.73500200
C	-0.55715600	0.30287700	-1.79801400
O	0.44095000	-1.90391500	-1.38886500
N	0.13158300	-1.69417100	-2.63582500
N	-0.36051200	-0.58586400	-2.92400800
P	0.42746000	-0.22673500	-0.30617200
H	-4.57296800	-1.59729200	-1.30345100
H	-1.94212600	2.49502400	-0.94559600
H	-4.34425900	2.56058100	-0.28599800
H	-5.69212300	0.45554700	-0.46409900
H	-0.19372900	1.29236700	-2.07376100
C	-0.27399700	-1.30532000	0.96700600
C	-1.57061700	-1.12588200	1.44252200
C	0.49798000	-2.35580700	1.45838800
C	-2.08889300	-1.99288500	2.39235700
H	-2.18268600	-0.30913400	1.08261300
C	-0.01892800	-3.20868000	2.42060100
H	1.50281400	-2.51377400	1.08605800
C	-1.31447900	-3.03243000	2.88584700
H	-3.10240400	-1.84929500	2.74934400
H	0.59166900	-4.02029100	2.79973900
H	-1.72054300	-3.70622300	3.63198500
C	2.24338200	-0.27970200	-0.40312600
C	2.92194400	-0.49689100	-1.59803700
C	2.96840600	-0.05317100	0.76585500
C	4.30833800	-0.47350500	-1.62365100
H	2.37995100	-0.68235300	-2.51634800
C	4.35349100	-0.06991400	0.74301200
H	2.45164500	0.13662000	1.69962300
C	5.02640500	-0.27055300	-0.45422000
H	4.82739700	-0.62707600	-2.56292800
H	4.90686100	0.08603000	1.66207300
H	6.11047500	-0.26808700	-0.47544500
C	0.36585400	1.42915400	0.56871100
C	0.89288200	2.53036000	-0.11446100
C	-0.12864000	1.63785800	1.85279500
C	0.89552000	3.79427500	0.44963100
H	1.32939300	2.40367200	-1.10077900
C	-0.12861200	2.90558100	2.42389600
H	-0.51201200	0.80915400	2.43279700
C	0.37505500	3.98861600	1.72337500

```
 H                 1.31106400    4.62954000   -0.10326400
 H                -0.52239700    3.03947400    3.42555500
 H                 0.37442000    4.97747800    2.16818500

         Item               Value     Threshold  Converged?
 Maximum Force            0.000009     0.000450     YES
 RMS     Force            0.000001     0.000300     YES
 Maximum Displacement     0.000882     0.001800     YES
 RMS     Displacement     0.000166     0.001200     YES
 Predicted change in Energy=-1.167918D-09
 Optimization completed.
    -- Stationary point found.

SCF Done:  E(RPBE1PBE) =  -1506.37157602     A.U. after    1 cycles

-------------------
 - Thermochemistry -
 -------------------

 Zero-point correction=                           0.387055 (Hartree/Particle)
 Thermal correction to Energy=                    0.410910
 Thermal correction to Enthalpy=                  0.411855
 Thermal correction to Gibbs Free Energy=         0.332430
 Sum of electronic and zero-point Energies=          -1505.984521
 Sum of electronic and thermal Energies=             -1505.960666
 Sum of electronic and thermal Enthalpies=           -1505.959721
 Sum of electronic and thermal Free Energies=        -1506.039146

                     E (Thermal)             CV                S
                      KCal/Mol        Cal/Mol-Kelvin    Cal/Mol-Kelvin
 Total                  257.850             94.347            167.162

5235,-7.0289312,-11.4741611\PG=C01 [X(C24H20N3O1P1)]\NImag=0\\0.677630
```

Intermediate Int4.2

\# opt freq PBE0-D3(BJ)/def2-TZVP SMD(THF)

0 1

C	-3.80824700	1.11951500	-1.51491700
N	-2.50613500	0.96097800	-1.73232700
C	-1.97228700	-0.24135600	-1.53197900
C	-2.72746100	-1.33572200	-1.12268100
C	-4.08065500	-1.15870400	-0.89640900
C	-4.63813600	0.09538600	-1.09029000
C	-0.48346000	-0.35131300	-1.77130100
O	0.19119400	-2.29892000	-0.44859800
N	0.19489000	-2.53128700	-1.72007300
N	-0.12388300	-1.58027100	-2.46816800
P	0.41945100	-0.21717300	-0.15829200
H	-4.20558400	2.11506400	-1.69144100
H	-2.26141800	-2.30015800	-0.97406400
H	-4.69186300	-1.99245500	-0.57006800
H	-5.69271900	0.27787400	-0.92364900
H	-0.18007100	0.47886900	-2.40531000
C	-0.51269500	-0.50940900	1.36559600
C	-1.41199900	0.47805200	1.77011400
C	-0.40363900	-1.68117700	2.11504700
C	-2.19873300	0.29145000	2.89487500
H	-1.50807400	1.39443200	1.20109000
C	-1.16915900	-1.84655700	3.25840600
H	0.26313800	-2.47050100	1.80167100
C	-2.07178000	-0.86696700	3.64700200
H	-2.90637600	1.05914800	3.18578800
H	-1.06641600	-2.75483100	3.84117300
H	-2.67723300	-1.00770100	4.53544400
C	2.17316100	-0.65815900	-0.22041900
C	2.87514100	-0.32936300	-1.37710200
C	2.83111600	-1.27092600	0.84095000
C	4.22427800	-0.63426100	-1.47691900
H	2.37842000	0.17157300	-2.20091900
C	4.18437000	-1.55344300	0.74146300
H	2.29789200	-1.52299900	1.74902000
C	4.88151800	-1.24365800	-0.41830400
H	4.76303800	-0.38612300	-2.38433200
H	4.69458000	-2.02361100	1.57458000
H	5.93806500	-1.47420400	-0.49500800
C	0.68694400	1.61178900	0.01559900
C	0.16613600	2.58303600	-0.83314800
C	1.48554800	2.02403200	1.08649700
C	0.44267800	3.92962600	-0.62225100
H	-0.49013200	2.31144200	-1.64922700
C	1.75749400	3.36336100	1.30022800
H	1.90395900	1.28803700	1.76524600
C	1.23770500	4.32394300	0.44041500

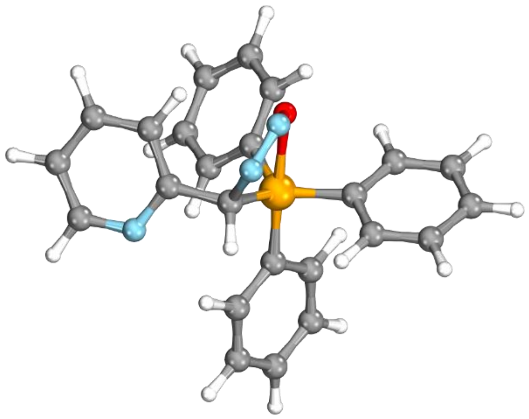

Fig. S40: Computed structure of **Int4.2**.

```
 H                 0.02597900    4.67044600   -1.29578600
 H                 2.37966200    3.65931300    2.13759700
 H                 1.45270600    5.37434500    0.60249600

         Item               Value     Threshold  Converged?
 Maximum Force            0.000006     0.000450     YES
 RMS     Force            0.000001     0.000300     YES
 Maximum Displacement     0.001744     0.001800     YES
 RMS     Displacement     0.000212     0.001200     YES
 Predicted change in Energy=-5.741287D-10
 Optimization completed.
    -- Stationary point found.

SCF Done:  E(RPBE1PBE) =  -1506.37211429     A.U. after    1 cycles

-------------------
 - Thermochemistry -
 -------------------

 Zero-point correction=                           0.387000 (Hartree/Particle)
 Thermal correction to Energy=                    0.411068
 Thermal correction to Enthalpy=                  0.412012
 Thermal correction to Gibbs Free Energy=         0.331059
 Sum of electronic and zero-point Energies=          -1505.985115
 Sum of electronic and thermal Energies=             -1505.961046
 Sum of electronic and thermal Enthalpies=           -1505.960102
 Sum of electronic and thermal Free Energies=        -1506.041055

                     E (Thermal)             CV                S
                      KCal/Mol        Cal/Mol-Kelvin    Cal/Mol-Kelvin
 Total                  257.949             94.517            170.379

,-2.4637205,-11.8531997\PG=C01 [X(C24H20N3O1P1)]\NImag=0\\0.67425433,-
```

Transition state TS9.1

opt freq PBE0-D3(BJ)/def2-TZVP SMD(THF)

0 1

C	3.91384200	1.66346800	-0.61075800
N	2.82423200	1.57191900	0.14310100
C	2.58154500	0.41295800	0.76992800
C	3.44081700	-0.68380500	0.65372000
C	4.57830600	-0.56492100	-0.12016600
C	4.82497500	0.63218700	-0.77790900
C	1.32706900	0.33549500	1.51237600
O	-0.96346400	-0.27045400	2.19988500
N	0.23513700	-0.96991900	2.97857900
N	1.28447700	-0.62015200	2.53731200
P	-0.67307000	-0.09045400	0.62552200
H	4.07191800	2.61814500	-1.10617000
H	3.20555700	-1.61110900	1.16211300
H	5.26120300	-1.40162600	-0.21968800
H	5.69970300	0.76732100	-1.40223700
H	1.00636500	1.29606700	1.91100300
C	-0.59080200	1.56816600	-0.09538800
C	-0.68664000	2.68106200	0.73563500
C	-0.53223500	1.74032800	-1.47465600
C	-0.69359300	3.95468600	0.19113200
H	-0.76259100	2.56064000	1.81105400
C	-0.51948200	3.01902800	-2.01221400
H	-0.50378100	0.88506100	-2.13840600
C	-0.59780200	4.12757700	-1.18286200
H	-0.77132800	4.81554200	0.84544600
H	-0.45556000	3.14530500	-3.08711300
H	-0.59258500	5.12556000	-1.60615000
C	-2.45538300	-0.38541600	0.15869800
C	-2.79094900	-0.92706700	-1.08058400
C	-3.48395200	0.05723500	0.99026800
C	-4.11728200	-1.03226800	-1.47625500
H	-2.01383700	-1.28135100	-1.74839200
C	-4.80991000	-0.04878000	0.59789900
H	-3.24898800	0.48543700	1.95750200
C	-5.13140800	-0.59406500	-0.63763100
H	-4.35696600	-1.46303300	-2.44241300
H	-5.59580900	0.29587200	1.26131900
H	-6.16816300	-0.67796500	-0.94431700
C	0.06813100	-1.46153000	-0.28451900
C	-0.29477400	-2.74484100	0.12461000
C	1.00786100	-1.29927200	-1.29897100
C	0.28051400	-3.85349200	-0.47593600
H	-1.03334800	-2.87841700	0.90789800
C	1.55431100	-2.41351000	-1.91749200
H	1.31885600	-0.31073100	-1.61120700
C	1.19752700	-3.68953600	-1.50516300

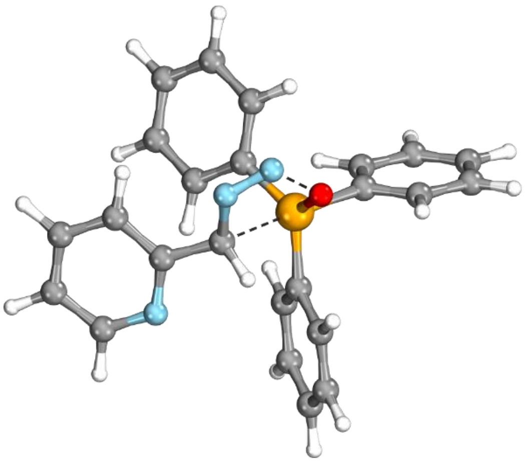

Fig. S41: Computed structure of **TS9.1**.

```
 H                 0.00172100   -4.84779300   -0.14587700
 H                 2.27392100   -2.28020400   -2.71731700
 H                 1.63645100   -4.55734200   -1.98445800

         Item               Value     Threshold  Converged?
 Maximum Force            0.000013     0.000450     YES
 RMS     Force            0.000001     0.000300     YES
 Maximum Displacement     0.000667     0.001800     YES
 RMS     Displacement     0.000143     0.001200     YES
 Predicted change in Energy=-1.768213D-09
 Optimization completed.
    -- Stationary point found.

SCF Done:  E(RPBE1PBE) =  -1506.35045764     A.U. after    1 cycles

-------------------
 - Thermochemistry -
 -------------------

 Zero-point correction=                           0.383984 (Hartree/Particle)
 Thermal correction to Energy=                    0.407812
 Thermal correction to Enthalpy=                  0.408756
 Thermal correction to Gibbs Free Energy=         0.329322
 Sum of electronic and zero-point Energies=          -1505.966474
 Sum of electronic and thermal Energies=             -1505.942646
 Sum of electronic and thermal Enthalpies=           -1505.941701
 Sum of electronic and thermal Free Energies=        -1506.021136

                     E (Thermal)             CV                S
                      KCal/Mol        Cal/Mol-Kelvin    Cal/Mol-Kelvin
 Total                  255.906             94.404           167.185

1,-5.7307948,-6.6649012,3.6795502\PG=C01 [X(C24H20N3O1P1)]\NImag=1\\0.
```

Transition state TS9.2

\# opt freq PBE0-D3(BJ)/def2-TZVP SMD(THF)

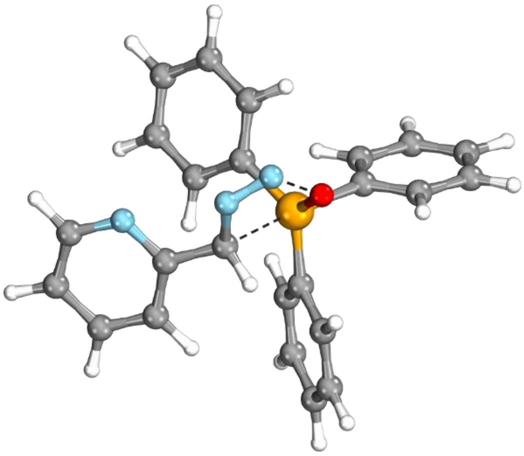

Fig. S42: Computed structure of **TS9.2**.

0 1

C	4.40658300	-0.56792900	-0.03056500
N	3.31392900	-0.60725200	0.72500800
C	2.52979600	0.47427600	0.76141300
C	2.82707900	1.63032800	0.03263800
C	3.95607300	1.64828900	-0.76169900
C	4.77050700	0.52371300	-0.80121200
C	1.28387900	0.39301100	1.52747300
O	-0.97470000	-0.31498100	2.21237000
N	0.27308900	-0.91821100	3.04293500
N	1.29839700	-0.53180000	2.59639300
P	-0.64252900	-0.10023400	0.65362800
H	5.02128100	-1.46443900	-0.02687200
H	2.17293700	2.49214400	0.09310600
H	4.20428700	2.53168500	-1.33982000
H	5.66769100	0.49423100	-1.40736000
H	0.97900900	1.35300300	1.94322000
C	-0.61759800	1.55976500	-0.08337900
C	-0.73420700	2.67737500	0.74000300
C	-0.56327500	1.72695100	-1.46372000
C	-0.76407500	3.94829000	0.18849900
H	-0.81188300	2.56216500	1.81609600
C	-0.57135000	3.00286400	-2.00907700
H	-0.52175200	0.86833100	-2.12239300
C	-0.66844700	4.11521600	-1.18650300
H	-0.86154700	4.81114200	0.83762800
H	-0.50969800	3.12386800	-3.08472000
H	-0.68085600	5.11061300	-1.61566200
C	-2.42008400	-0.43084600	0.15192200
C	-2.73168400	-1.00542900	-1.07877600
C	-3.46888100	0.02284200	0.95262300
C	-4.05031100	-1.13186600	-1.49524500
H	-1.94097200	-1.36919700	-1.72516800
C	-4.78712100	-0.10439900	0.54112700
H	-3.25516600	0.47731800	1.91304700
C	-5.08308500	-0.68273500	-0.68597200
H	-4.26904200	-1.58841300	-2.45466100
H	-5.58730900	0.24929700	1.18253600
H	-6.11361400	-0.78358300	-1.00829300
C	0.12280200	-1.45824300	-0.26249000
C	-0.19337700	-2.74478800	0.17234300
C	1.02762500	-1.28704000	-1.30598000
C	0.39274900	-3.84664800	-0.42997900
H	-0.90416600	-2.88675800	0.97962300
C	1.58863200	-2.39440900	-1.92345100
H	1.30530100	-0.29587500	-1.64015900
C	1.27794100	-3.67346400	-1.48480800

```
 H                 0.15002000   -4.84306600   -0.07850300
 H                 2.28384600   -2.25303300   -2.74332600
 H                 1.72823200   -4.53598300   -1.96315400

         Item               Value     Threshold  Converged?
 Maximum Force            0.000002     0.000450     YES
 RMS     Force            0.000000     0.000300     YES
 Maximum Displacement     0.000575     0.001800     YES
 RMS     Displacement     0.000091     0.001200     YES
 Predicted change in Energy=-3.909394D-10
 Optimization completed.
    -- Stationary point found.

SCF Done:  E(RPBE1PBE) =  -1506.34984685     A.U. after    1 cycles

-------------------
 - Thermochemistry -
 -------------------

 Zero-point correction=                           0.384146 (Hartree/Particle)
 Thermal correction to Energy=                    0.407940
 Thermal correction to Enthalpy=                  0.408885
 Thermal correction to Gibbs Free Energy=         0.329378
 Sum of electronic and zero-point Energies=          -1505.965701
 Sum of electronic and thermal Energies=             -1505.941906
 Sum of electronic and thermal Enthalpies=           -1505.940962
 Sum of electronic and thermal Free Energies=        -1506.020469

                     E (Thermal)             CV                S
                      KCal/Mol        Cal/Mol-Kelvin    Cal/Mol-Kelvin
 Total                  255.987            94.283            167.336

,1.4420226,-8.6679867,5.9822365|PG=C01 [X(C24H20N3O1P1)]|NImag=1||0.56
```

Triphenylphosphine oxide

opt freq PBE0-D3(BJ)/def2-TZVP SMD(THF)

0 1

P	-0.01131100	-0.02388800	0.93547900
O	-0.04247600	-0.09144700	2.42116600
C	0.37700000	1.61807000	0.28830900
C	1.65411500	2.13158500	0.52038600
C	-0.56962000	2.40433100	-0.36166200
C	1.97845000	3.41038400	0.10015600
H	2.39747300	1.52682000	1.02948600
C	-0.24209500	3.68635800	-0.78134700
H	-1.56582300	2.01778100	-0.54274600
C	1.03020300	4.18857900	-0.55306100
H	2.97312900	3.80203700	0.28095400
H	-0.98445100	4.29281500	-1.28794100
H	1.28564000	5.18936200	-0.88330900
C	-1.59314400	-0.49680000	0.19917900
C	-1.73514300	-0.78866300	-1.15598100
C	-2.70098700	-0.56302100	1.03893200
C	-2.97608300	-1.13621100	-1.66466600
H	-0.87635800	-0.74809500	-1.81689100
C	-3.94270800	-0.91224700	0.52700900
H	-2.57860400	-0.34407300	2.09361500
C	-4.08061100	-1.19729400	-0.82365600
H	-3.08223900	-1.36441300	-2.71928400
H	-4.80280400	-0.96362900	1.18521000
H	-5.05031100	-1.47231500	-1.22362100
C	1.22326900	-1.12731600	0.21231600
C	1.57959900	-2.26018200	0.94063500
C	1.79702300	-0.90240500	-1.03714900
C	2.49597300	-3.16170900	0.42132100
H	1.14074600	-2.42384200	1.91859700
C	2.71171500	-1.80693200	-1.55490800
H	1.53864200	-0.01472400	-1.60383000
C	3.06045400	-2.93662100	-0.82702100
H	2.77270800	-4.04019700	0.99340700
H	3.15842800	-1.62613200	-2.52616900
H	3.77882900	-3.64092700	-1.23176000

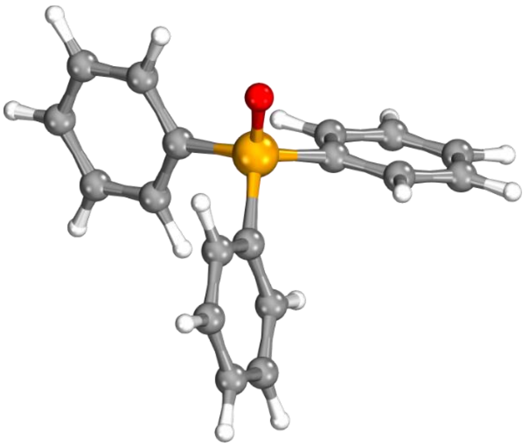

Fig. S43: Computed structure of **Triphenylphosphine oxide.**

Item	Value	Threshold	Converged?
Maximum Force	0.000001	0.000450	YES
RMS Force	0.000000	0.000300	YES
Maximum Displacement	0.000044	0.001800	YES
RMS Displacement	0.000010	0.001200	YES

Predicted change in Energy=-6.337510D-12
Optimization completed.
-- Stationary point found.

SCF Done: E(RPBE1PBE) = -1110.88410241 A.U. after 1 cycles

```
-------------------
 - Thermochemistry -
 -------------------

 Zero-point correction=                           0.279559 (Hartree/Particle)
 Thermal correction to Energy=                    0.296285
 Thermal correction to Enthalpy=                  0.297230
 Thermal correction to Gibbs Free Energy=         0.232793
 Sum of electronic and zero-point Energies=          -1110.604543
 Sum of electronic and thermal Energies=             -1110.587817
 Sum of electronic and thermal Enthalpies=           -1110.586873
 Sum of electronic and thermal Free Energies=        -1110.651310

                     E (Thermal)             CV                S
                      KCal/Mol        Cal/Mol-Kelvin    Cal/Mol-Kelvin
 Total                  185.922             65.493            135.618

933047|PG=C01 [X(C18H15O1P1)]|NImag=0||0.39065710,-0.00039781,0.375292
```

7. NMR spectra

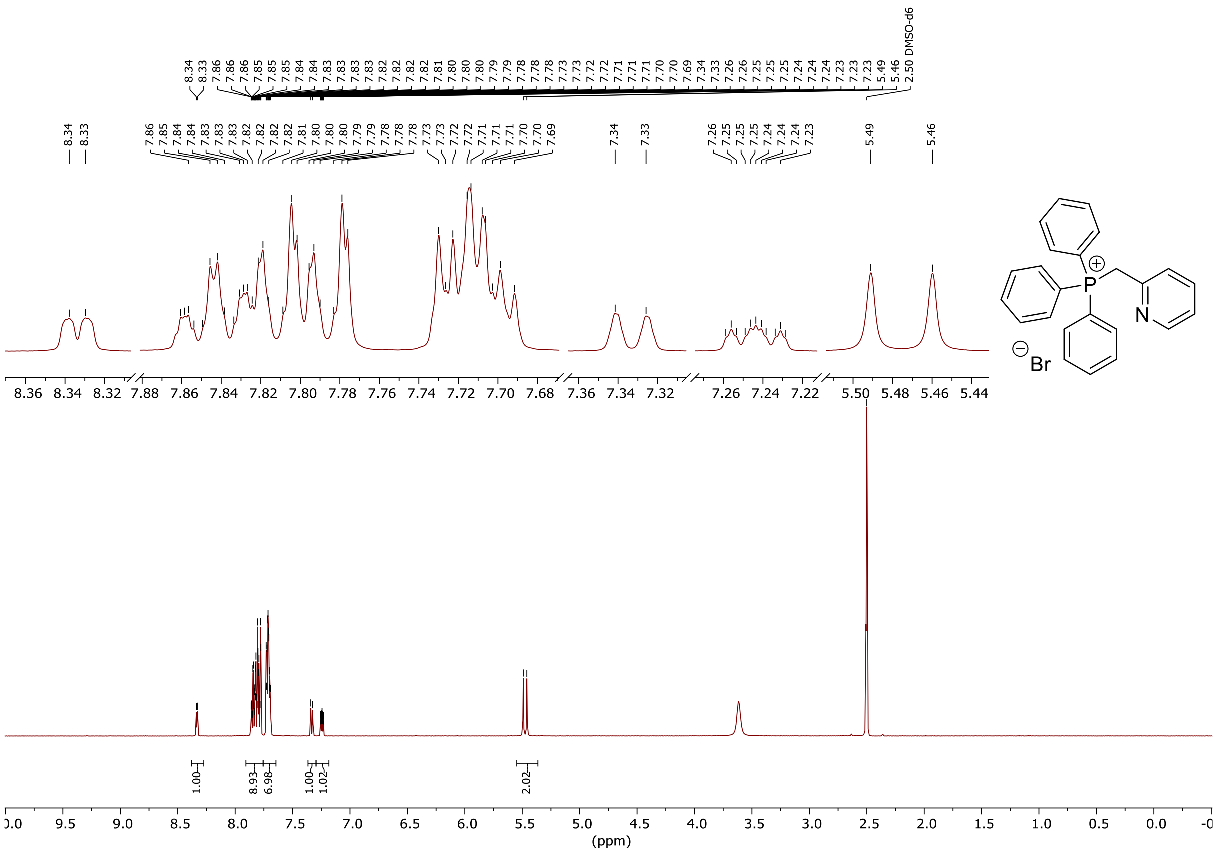

Fig. S44 ^{1}H NMR (501 MHz, dimethyl sulfoxide-d_6, 298 K) spectrum of compound **1a**

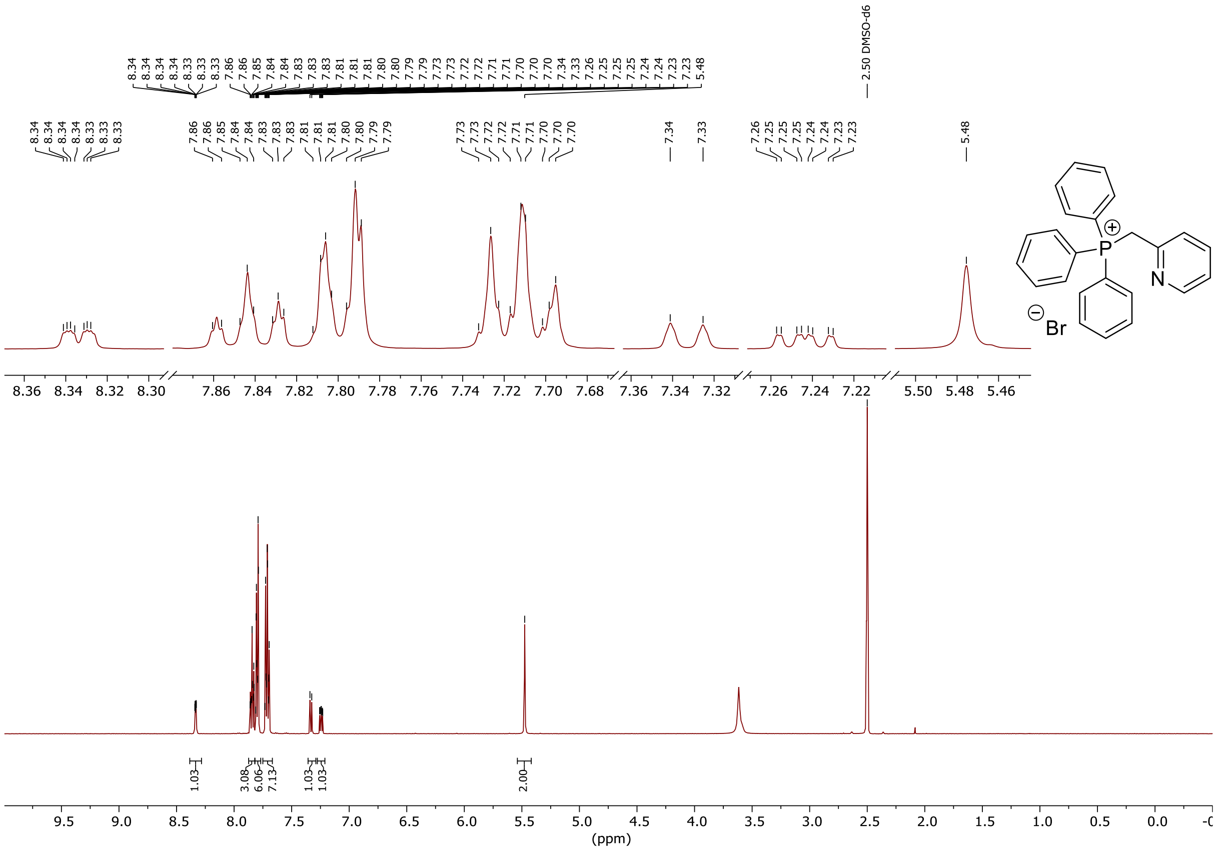

Fig. S45 $^{1}H\{^{31}P\}$ NMR (501 MHz, dimethyl sulfoxide-d_6, 298 K) spectrum of compound **1a**

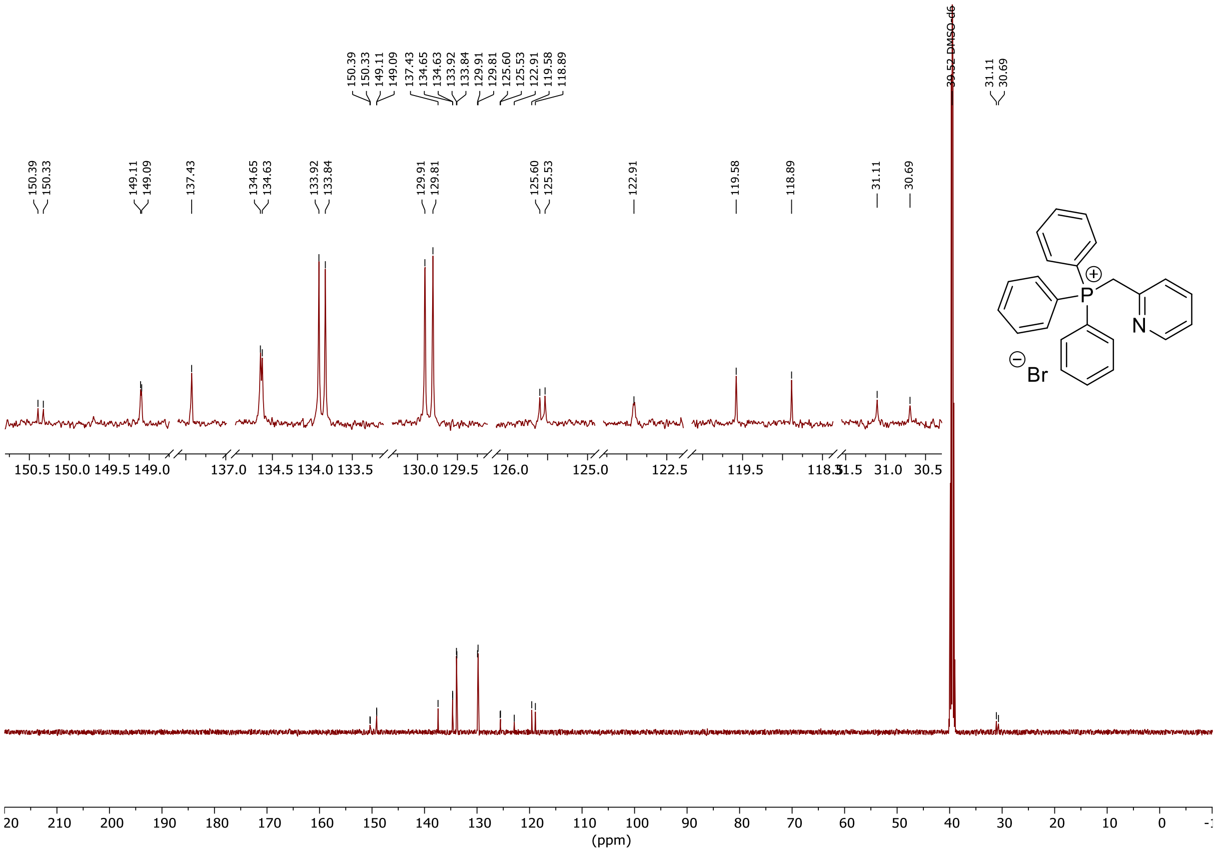

Fig. S46 $^{13}C\{^1H\}$ NMR (126 MHz, dimethyl sulfoxide-d_6, 298 K) spectrum of compound **1a**

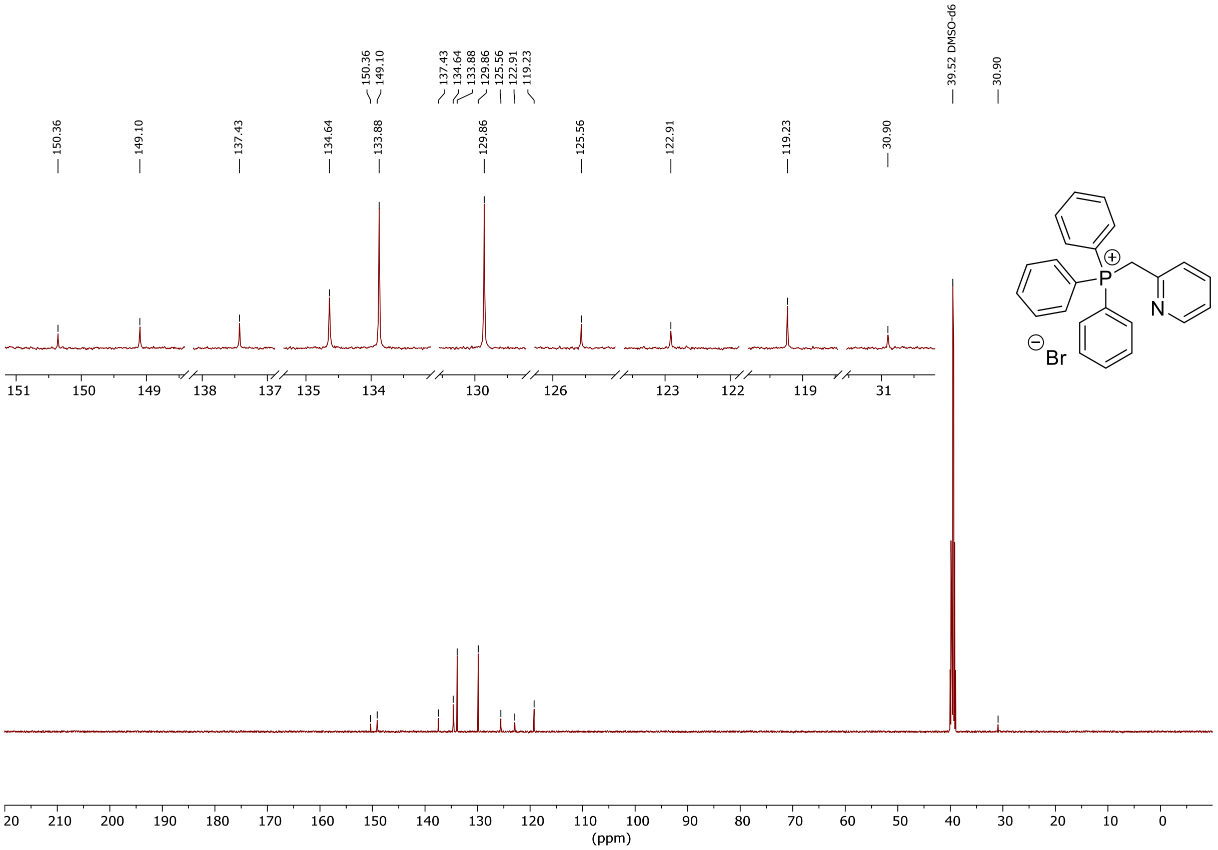

Fig. S47 $^{13}C\{^1H, ^{31}P\}$ NMR (126 MHz, dimethyl sulfoxide-d_6, 298 K) spectrum of compound **1a**

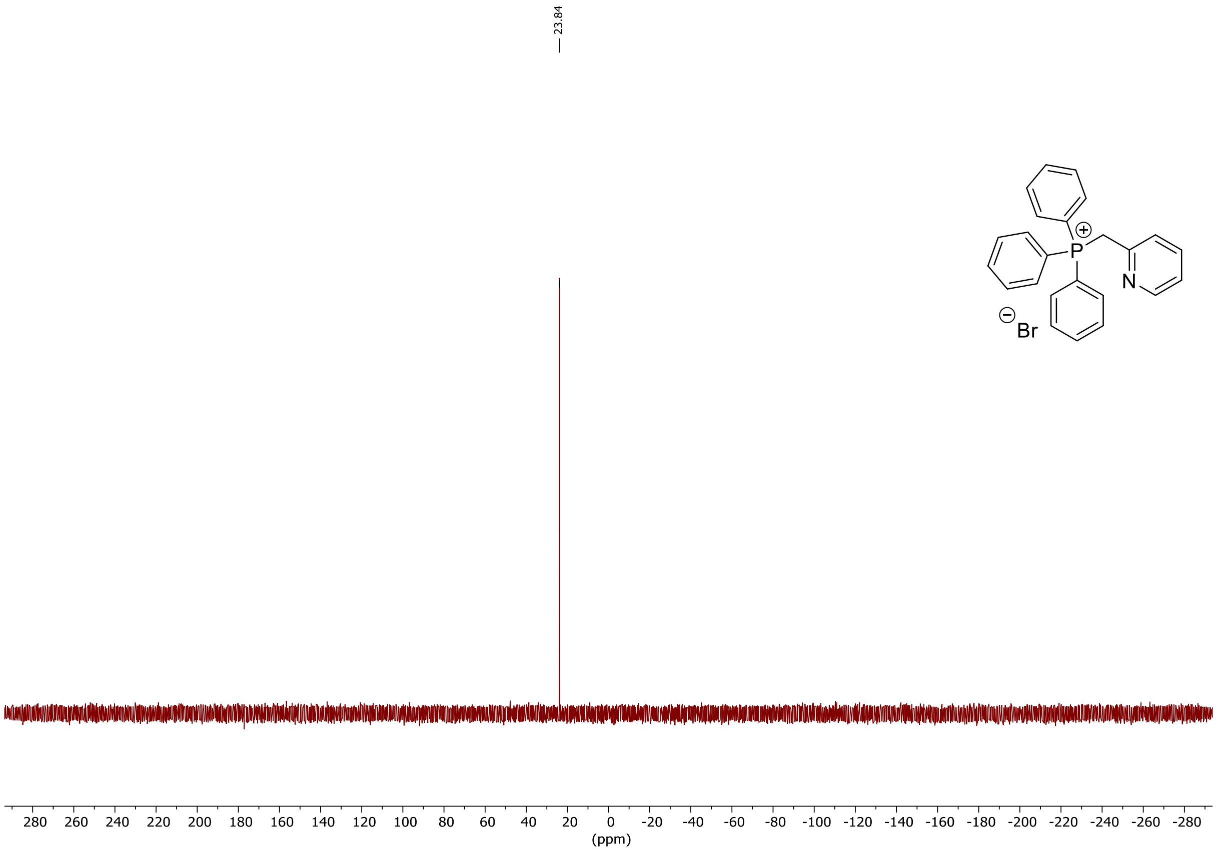

Fig. S48 ^{31}P NMR (202 MHz, dimethyl sulfoxide-*d*$_6$, 298 K) spectrum of compound **1a**

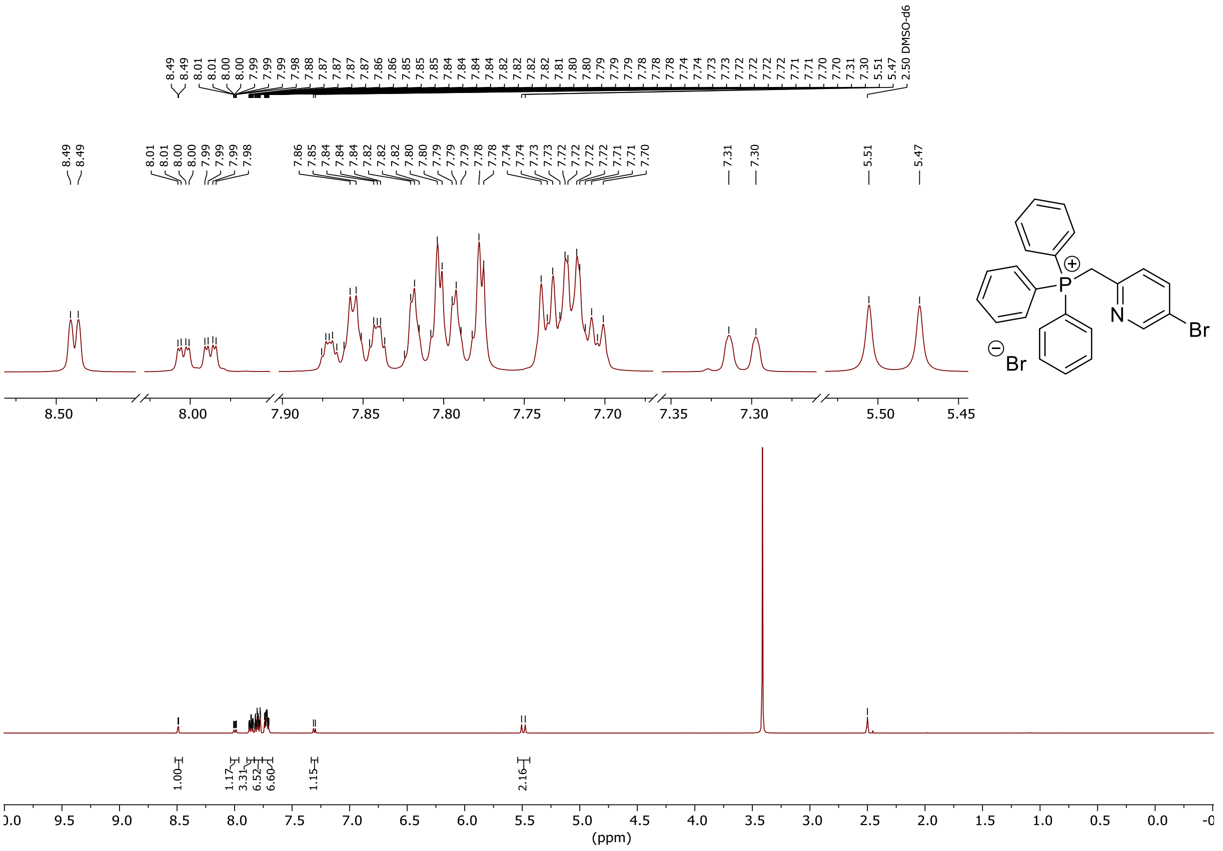

Fig. S49 ^{1}H NMR (501 MHz, dimethyl sulfoxide-d_6, 298 K) spectrum of compound **1b**

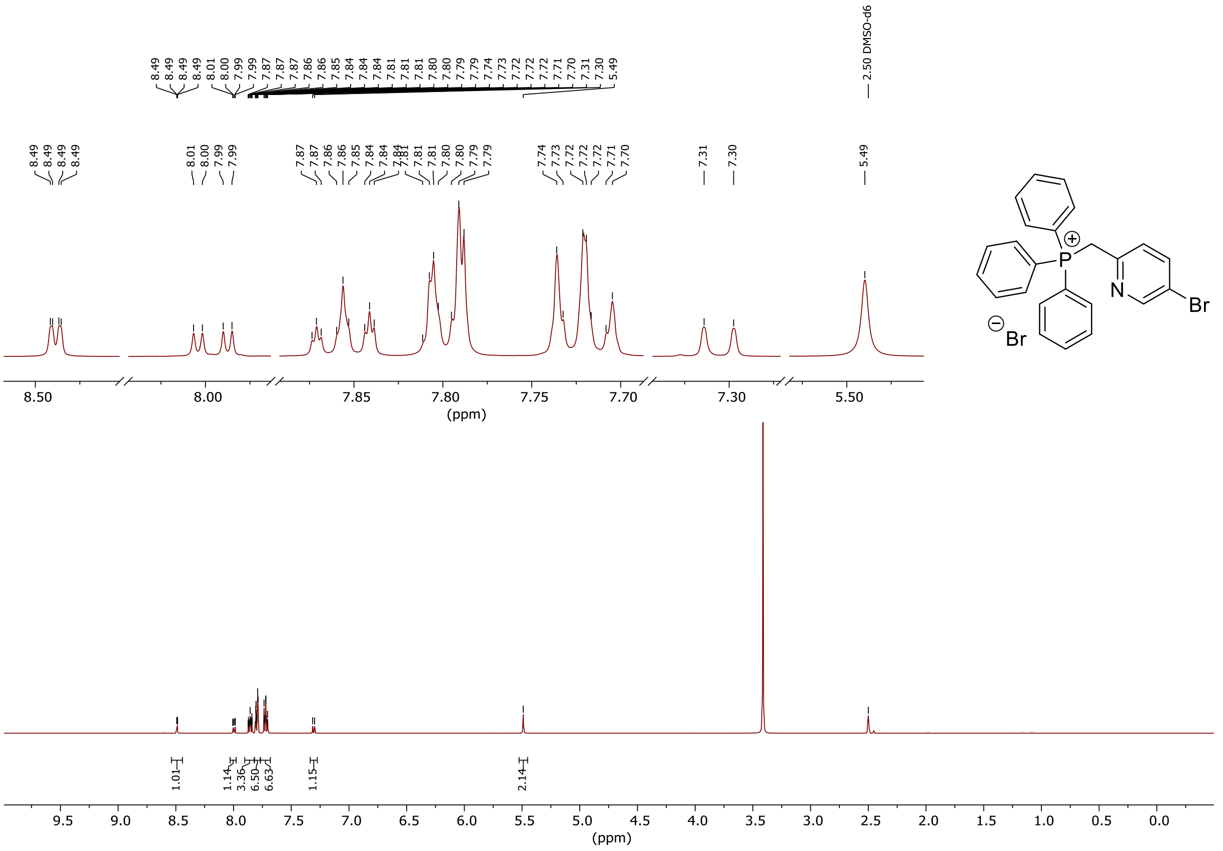

Fig. S50 ^{1}H{^{31}P} NMR (501 MHz, dimethyl sulfoxide-d_6, 298 K) spectrum of compound **1b**

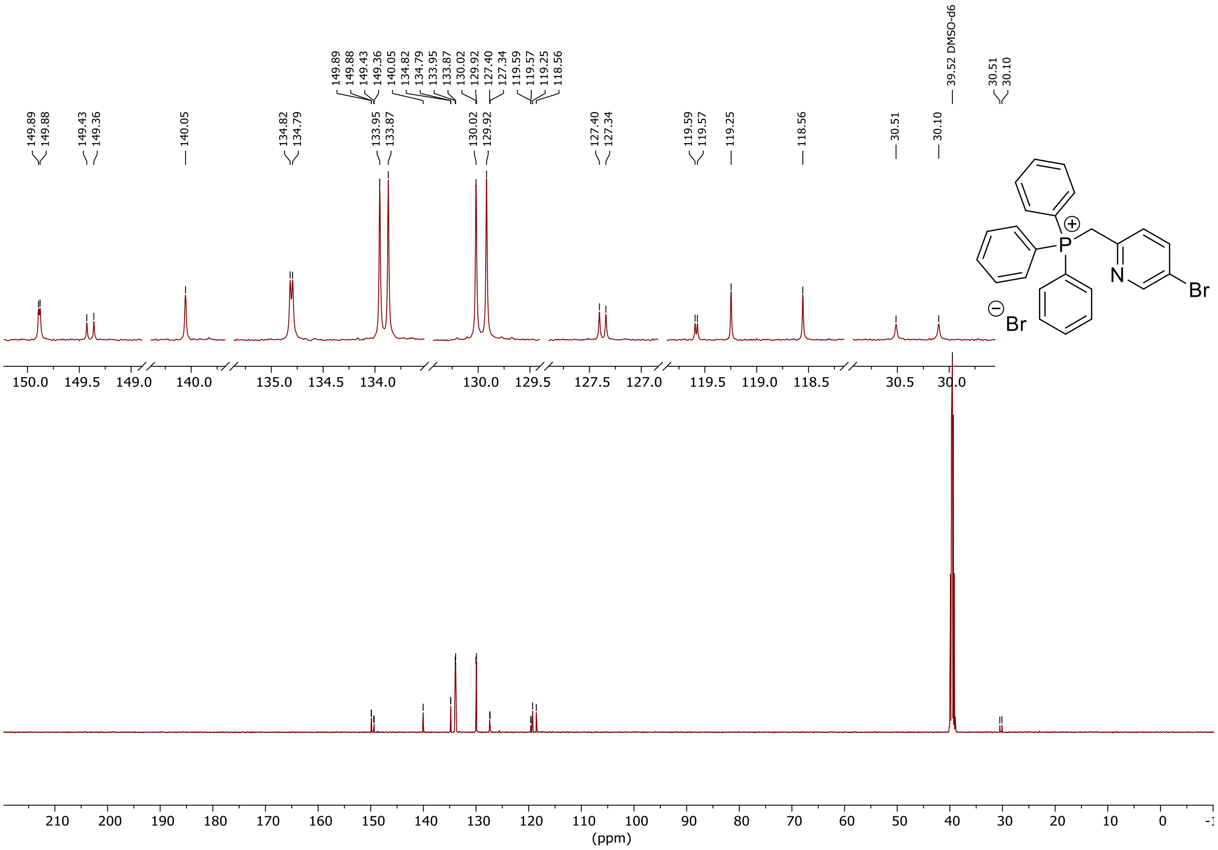

Fig. S51 $^{13}C\{^{1}H\}$ NMR (126 MHz, dimethyl sulfoxide-d_6, 298 K) spectrum of compound **1b**

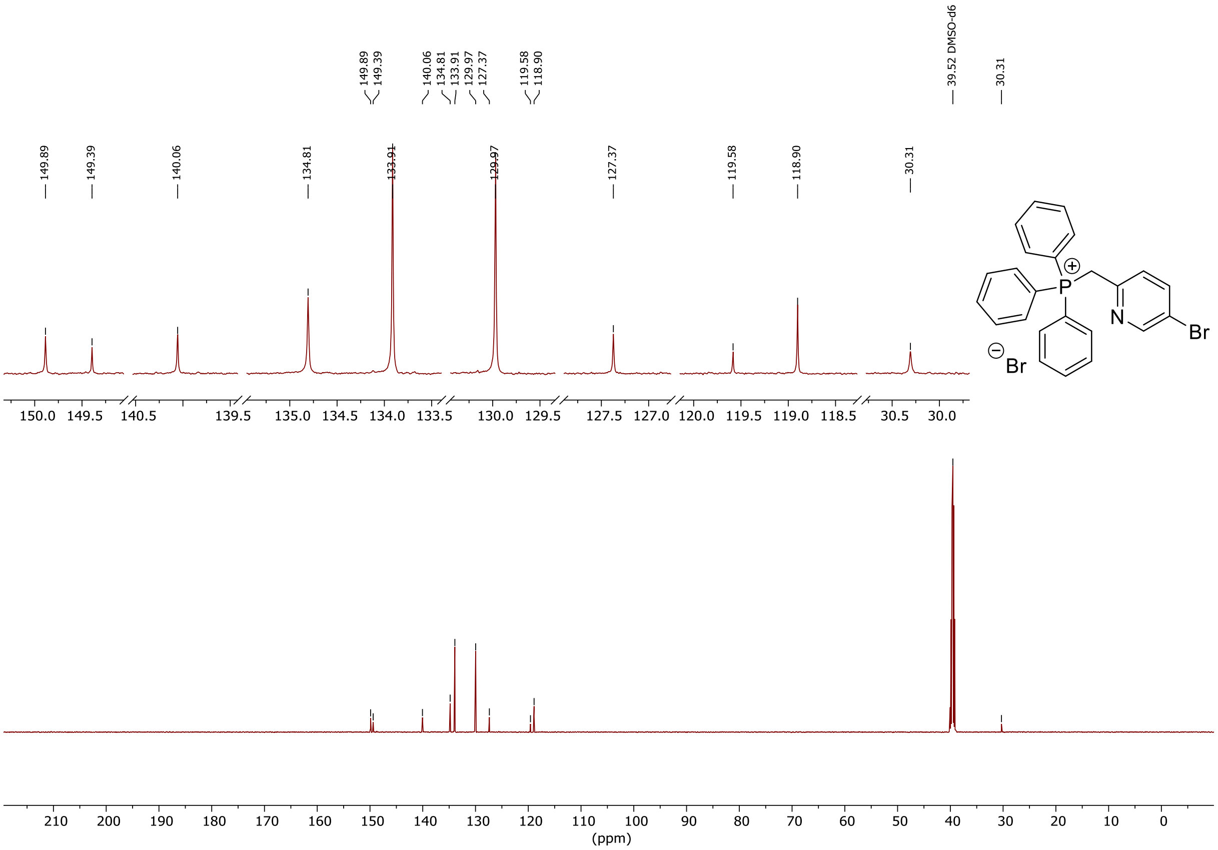

Fig. S52 $^{13}C\{^{1}H,^{31}P\}$ NMR (126 MHz, dimethyl sulfoxide-d_6, 298 K) spectrum of compound **1b**

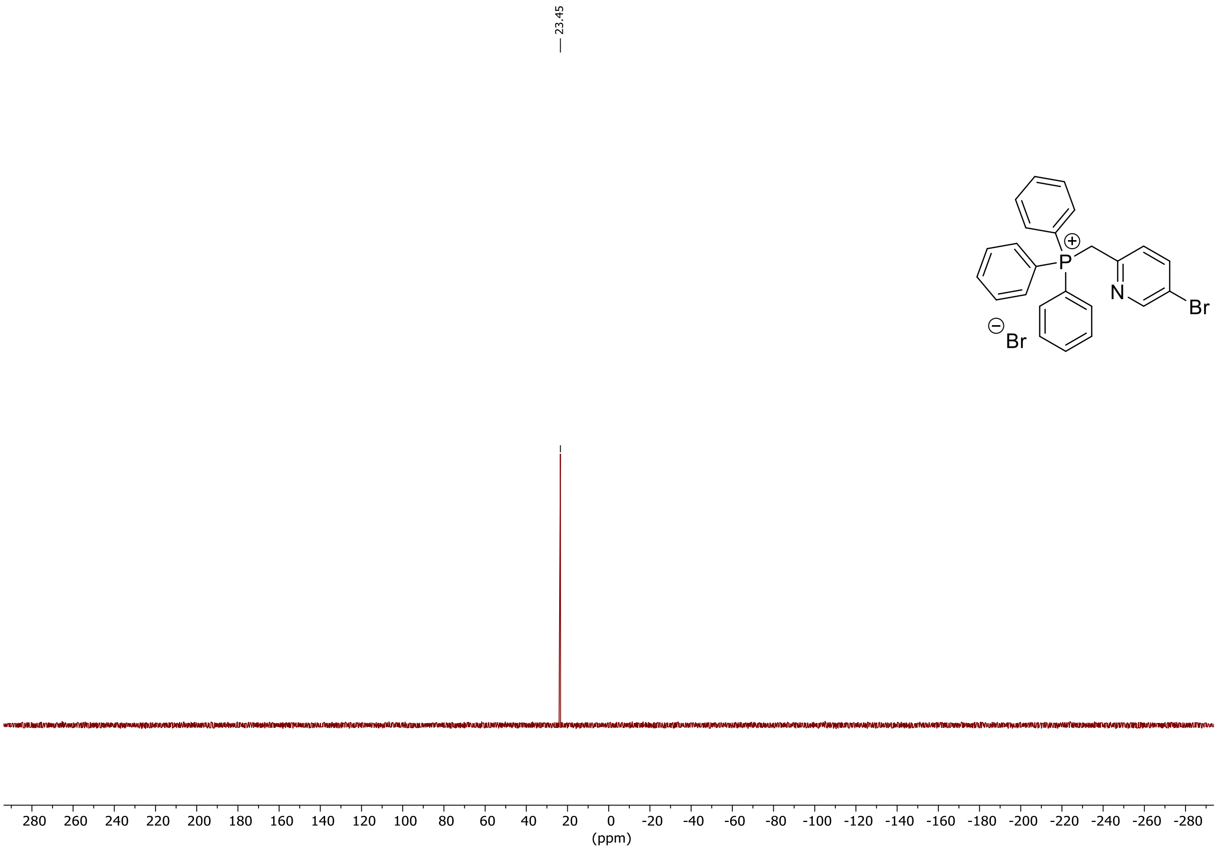

Fig. S53 ^{31}P NMR (202 MHz, dimethyl sulfoxide-d_6, 298 K) spectrum of compound **1b**

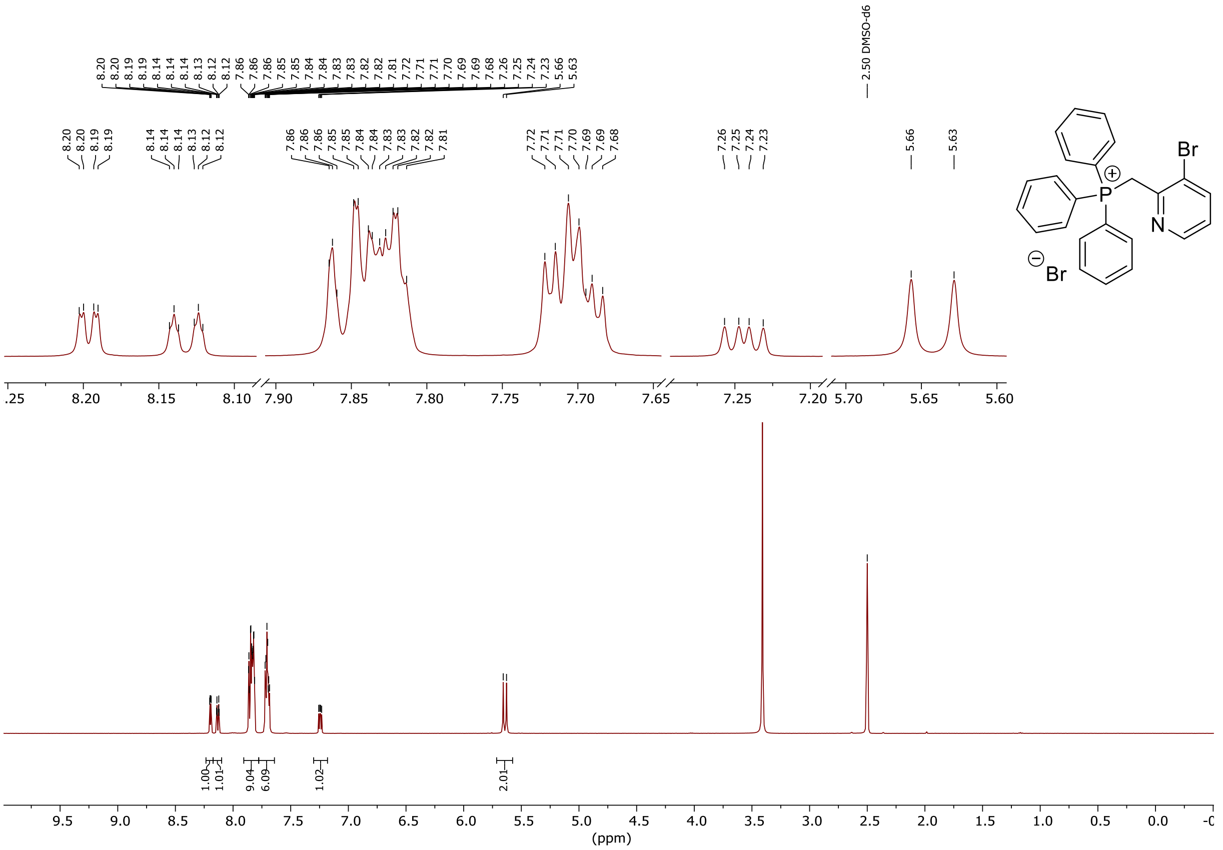

Fig. S54 ^{1}H NMR (501 MHz, dimethyl sulfoxide-d_6, 298 K) spectrum of compound **1c**

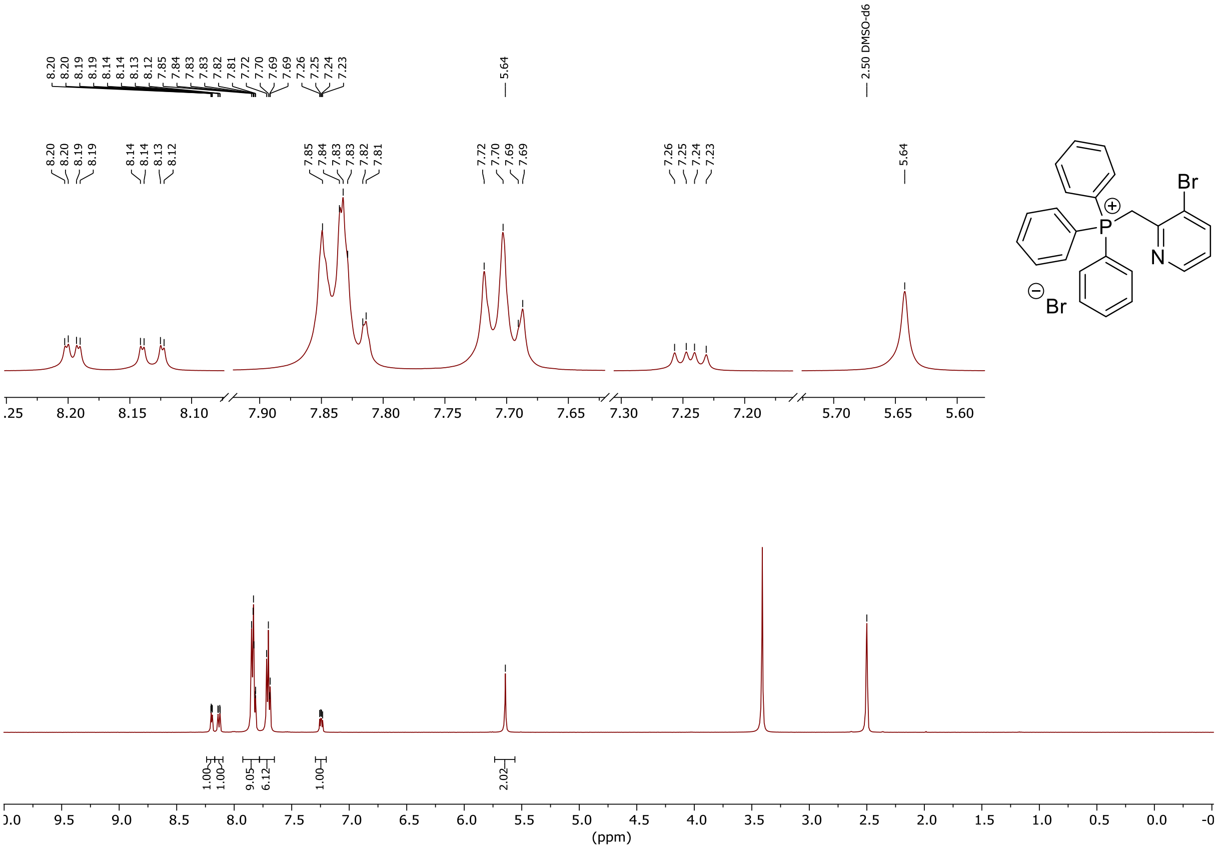

Fig. S55 ^{1}H{^{31}P} NMR (501 MHz, dimethyl sulfoxide-d_6, 298 K) spectrum of compound **1c**

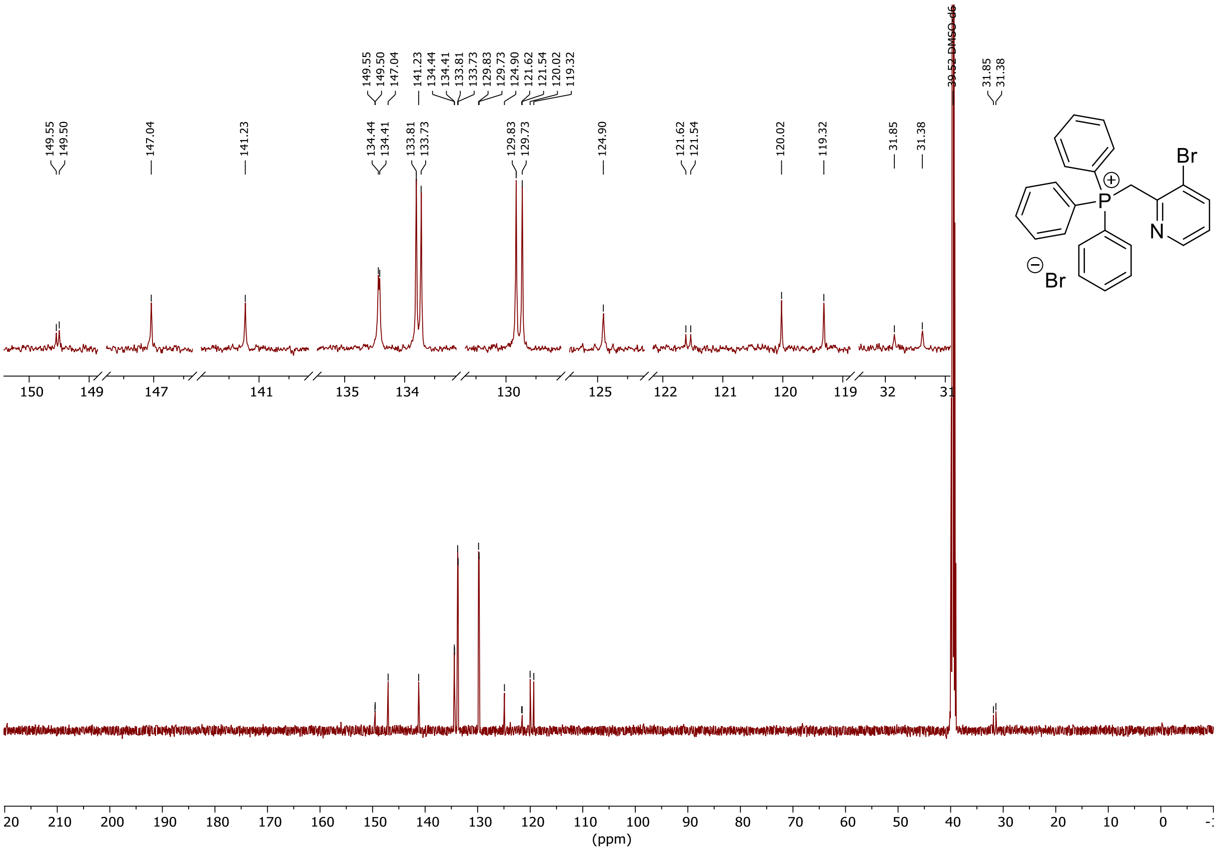

Fig. S56 $^{13}C\{^1H\}$ NMR (126 MHz, dimethyl sulfoxide-d_6, 298 K) spectrum of compound **1c**

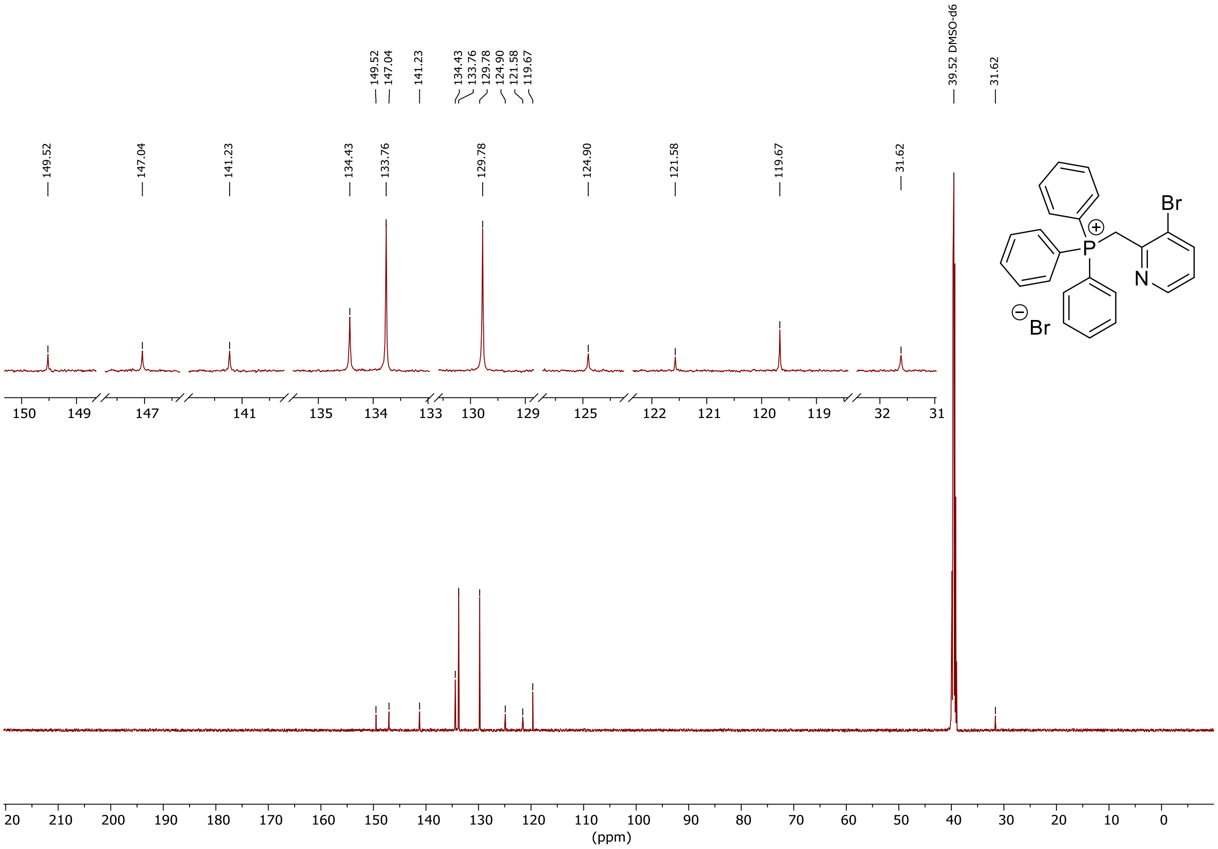

Fig. S57 $^{13}C\{^1H,^{31}P\}$ NMR (126 MHz, dimethyl sulfoxide-d_6, 298 K) spectrum of compound **1c**.

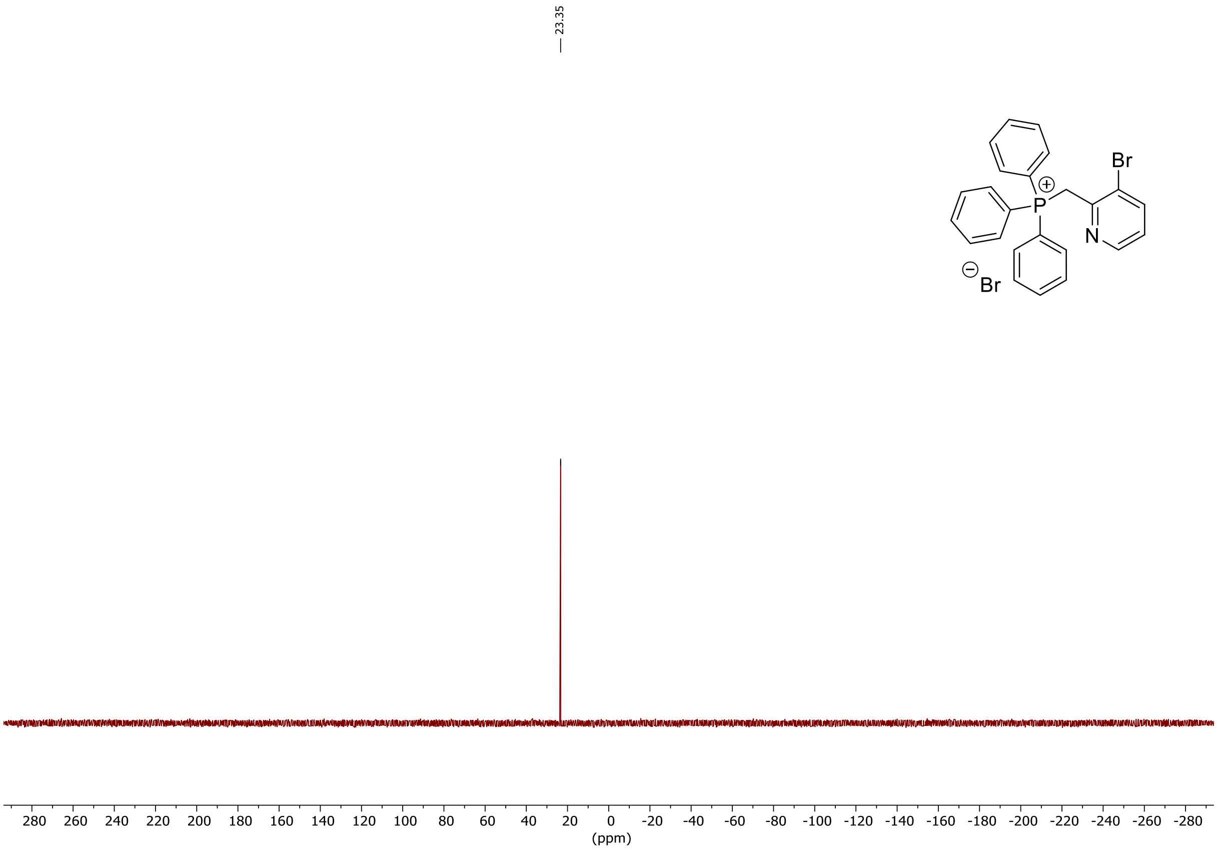

Fig. S58 ^{31}P NMR (202 MHz, dimethyl sulfoxide-d_6, 298 K) spectrum of compound **1c**

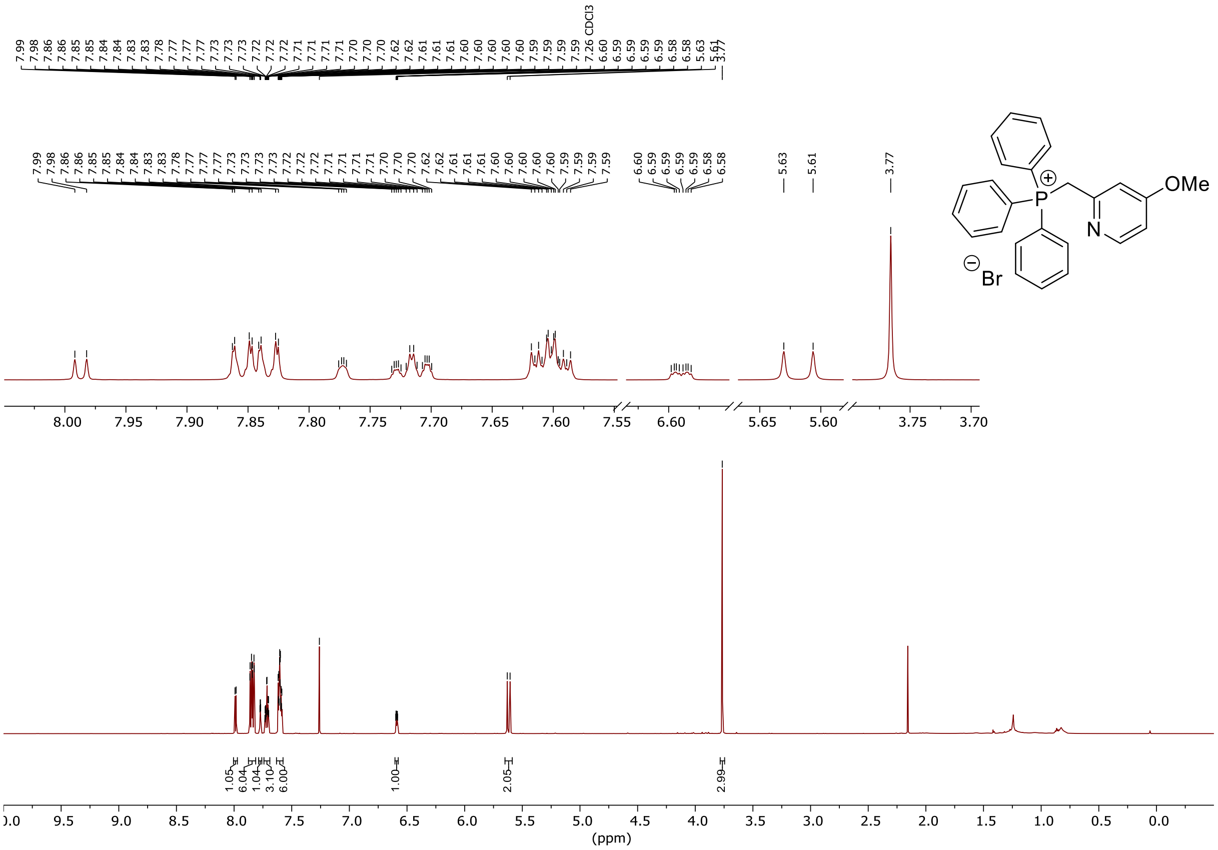

Fig. S59 ^{1}H NMR (600 MHz, chloroform-*d*, 300 K) spectrum of compound **1d**

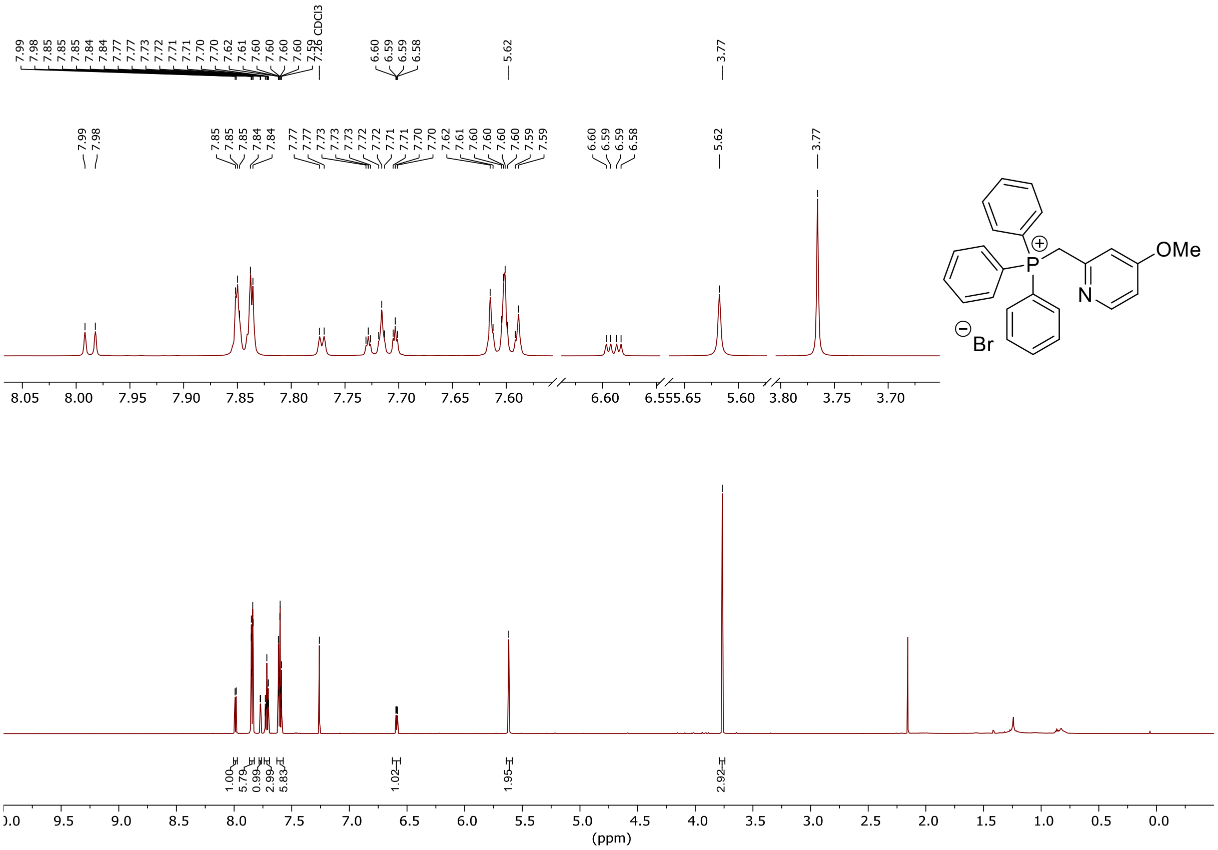

Fig. S60 $^{1}H\{^{31}P\}$ NMR (600 MHz, chloroform-*d*, 300 K) spectrum of compound **1d**

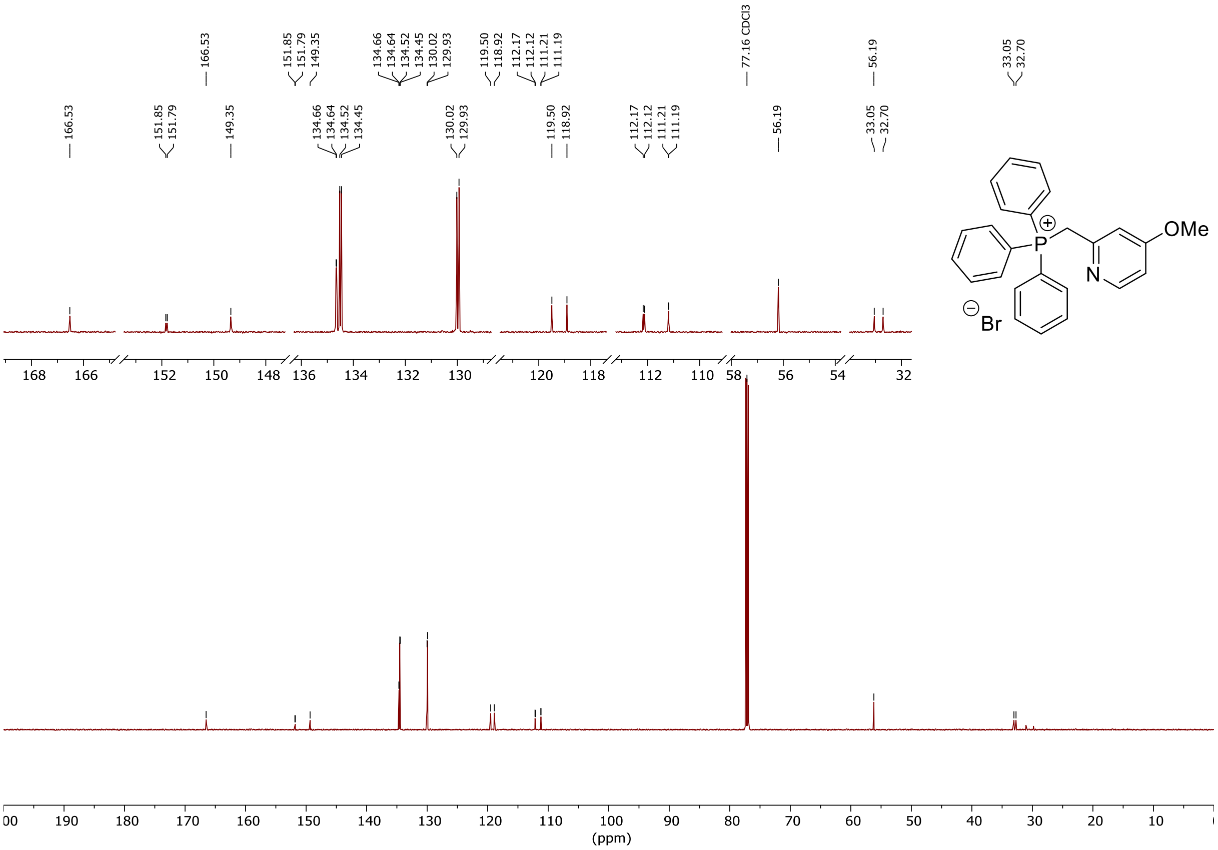

Fig. S61 $^{13}C\{^{1}H\}$ NMR (151 MHz, chloroform-*d*, 300 K) spectrum of compound **1d**

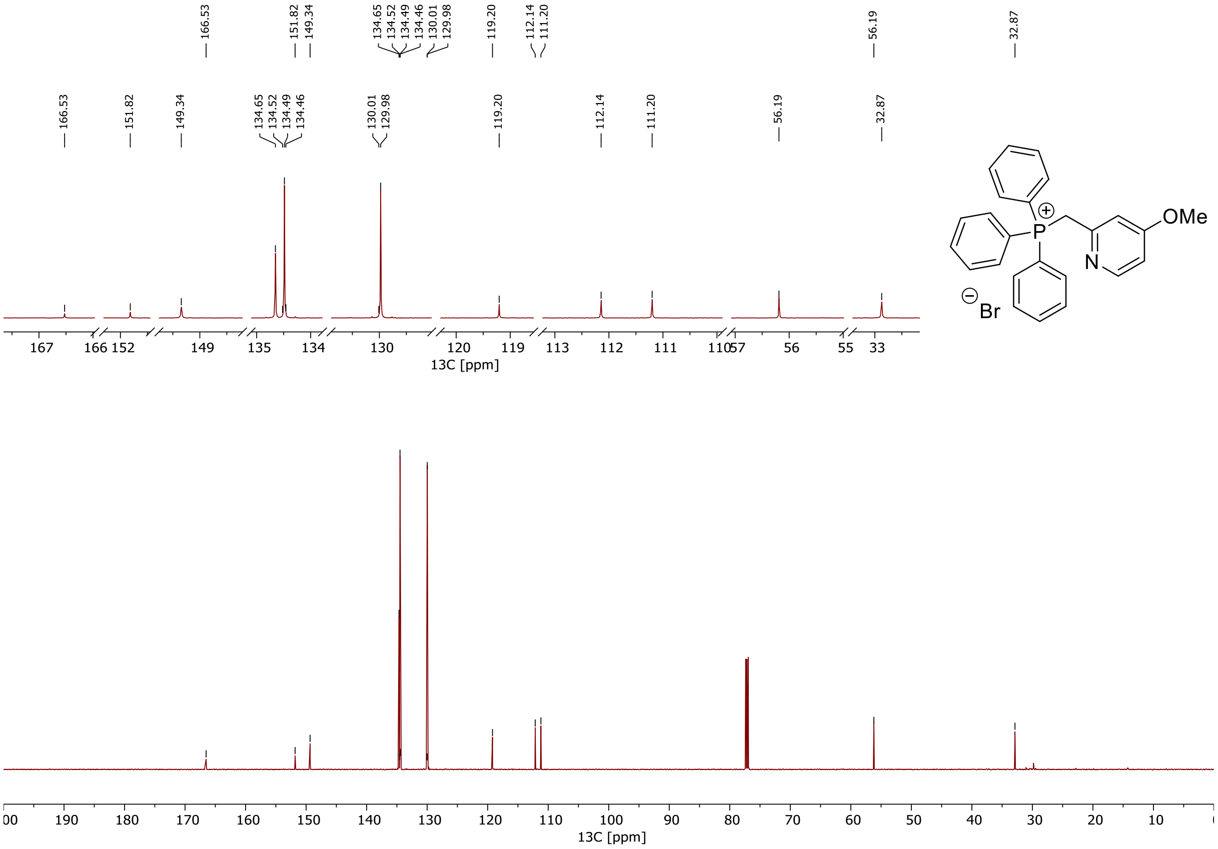

Fig. S62 $^{13}C\{^{1}H,^{31}P\}$ NMR (151 MHz, chloroform-*d*, 300 K) spectrum of compound **1d**

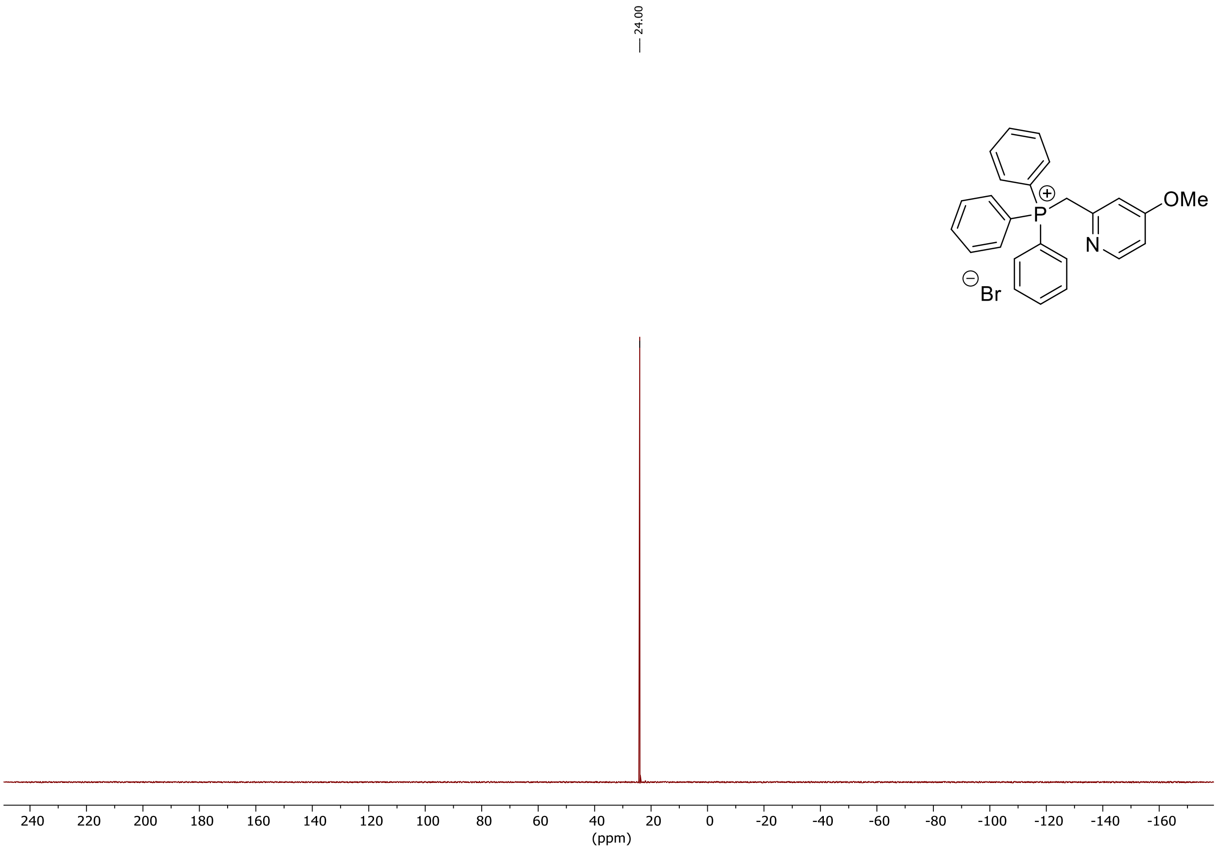

Fig. S63 ^{31}P NMR (243 MHz, chloroform-*d*, 300 K) spectrum of compound **1d**

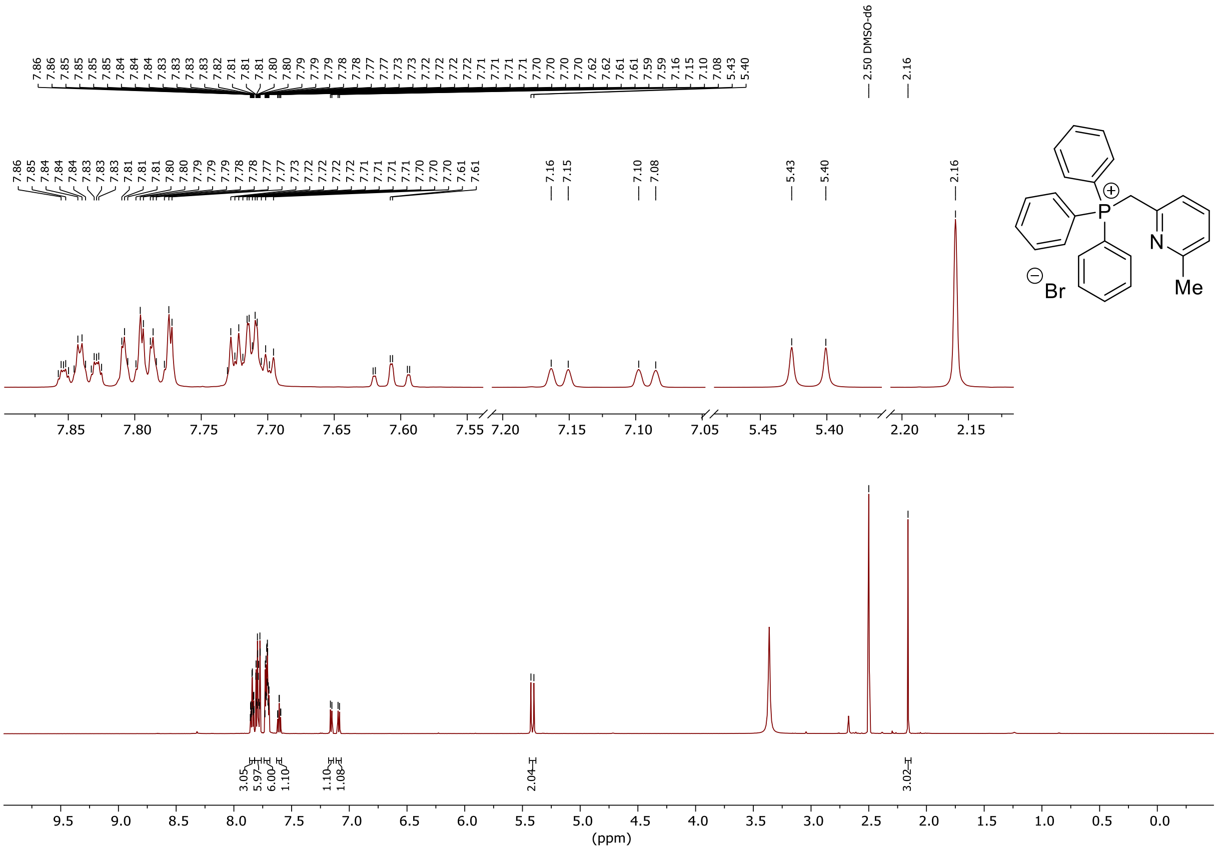

Fig. S64 ¹H NMR (501 MHz, dimethyl sulfoxide-d_6, 298 K) spectrum of compound **1e**

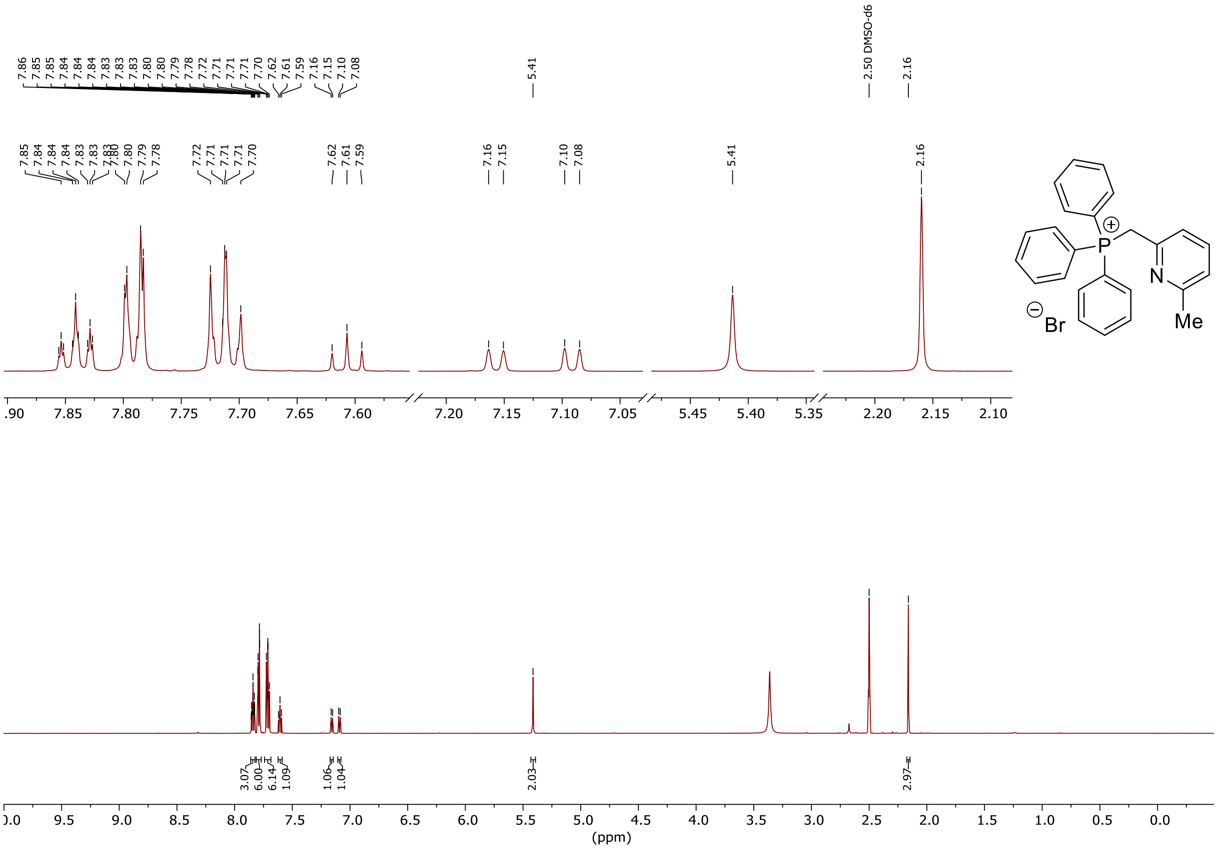

Fig. S65 ¹H{³¹P} NMR (501 MHz, dimethyl sulfoxide-d_6, 298 K) spectrum of compound **1e**

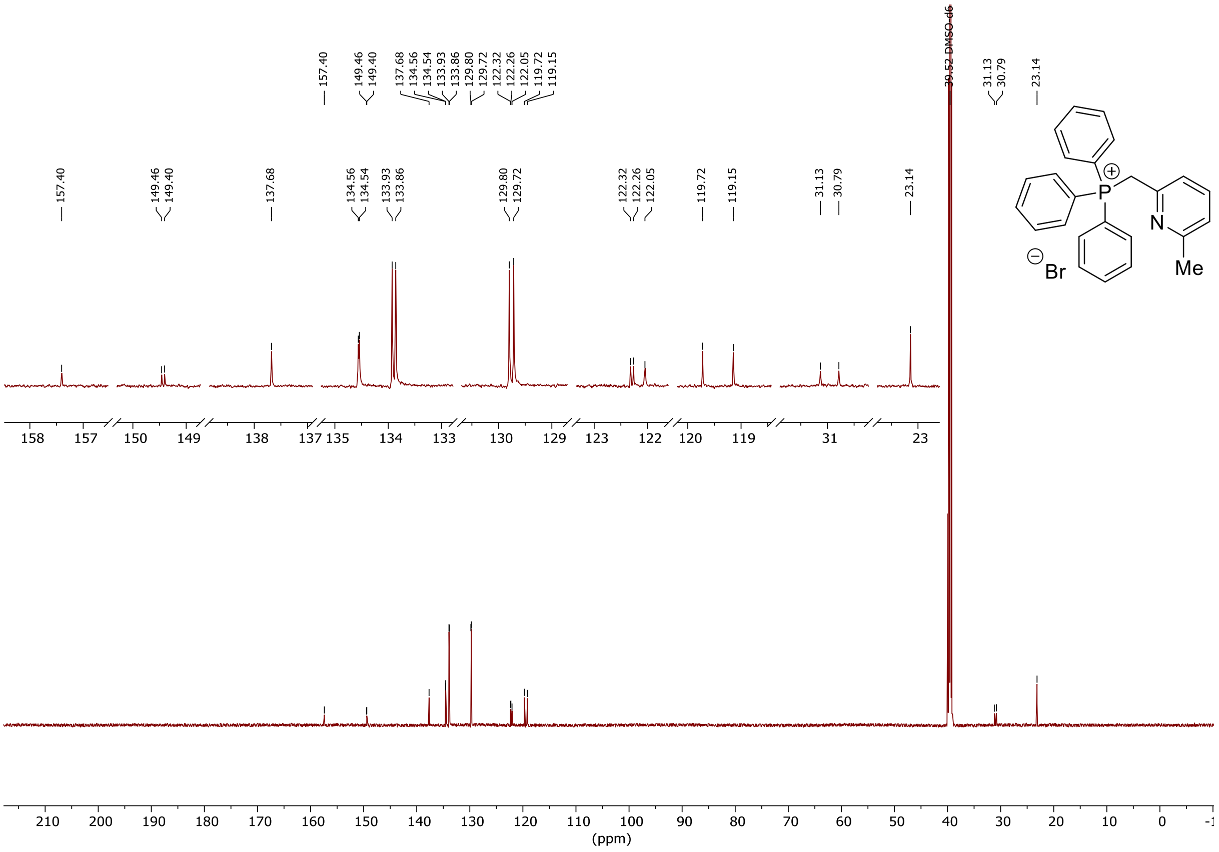

Fig. S66 $^{13}C\{^{1}H\}$ NMR (126 MHz, dimethyl sulfoxide-d_6, 298 K) spectrum of compound **1e**

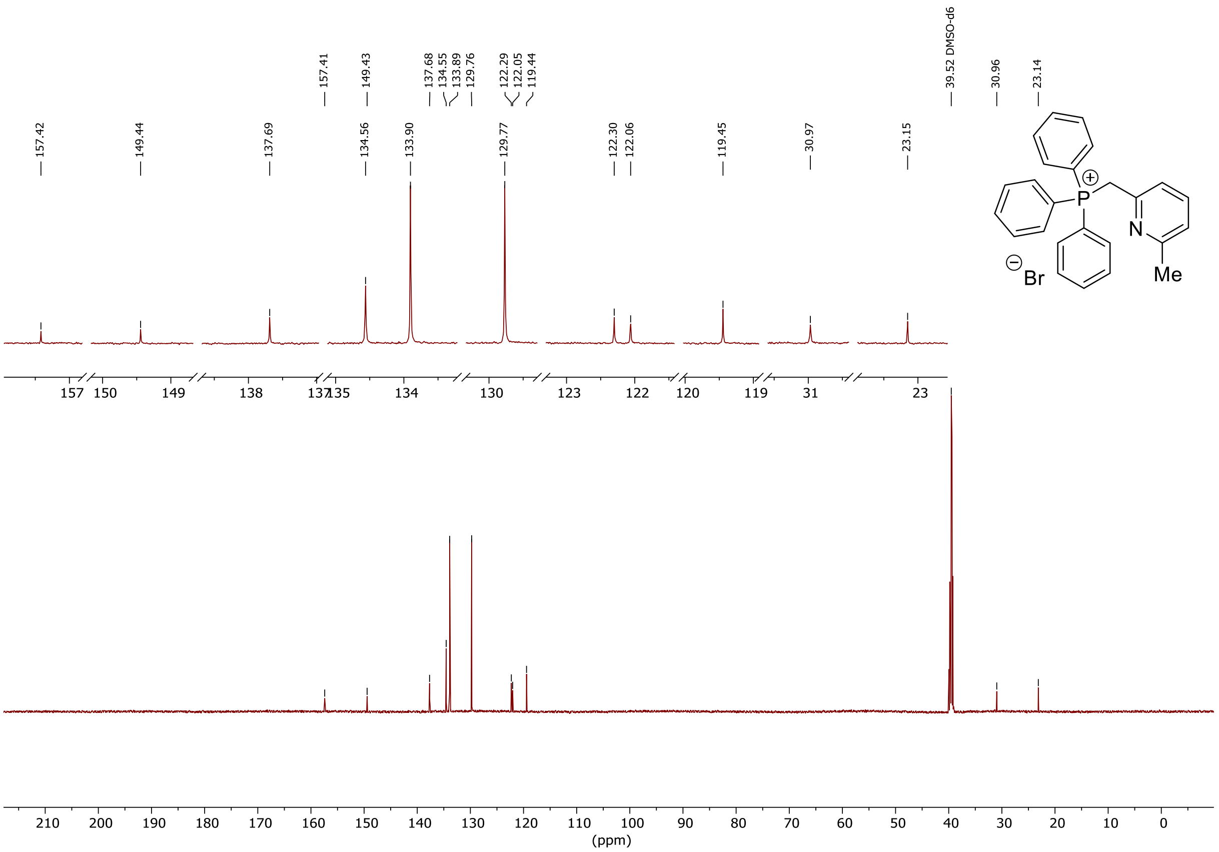

Fig. S67 $^{13}C\{^{1}H,^{31}P\}$ NMR (126 MHz, dimethyl sulfoxide-d_6, 298 K) spectrum of compound **1e**

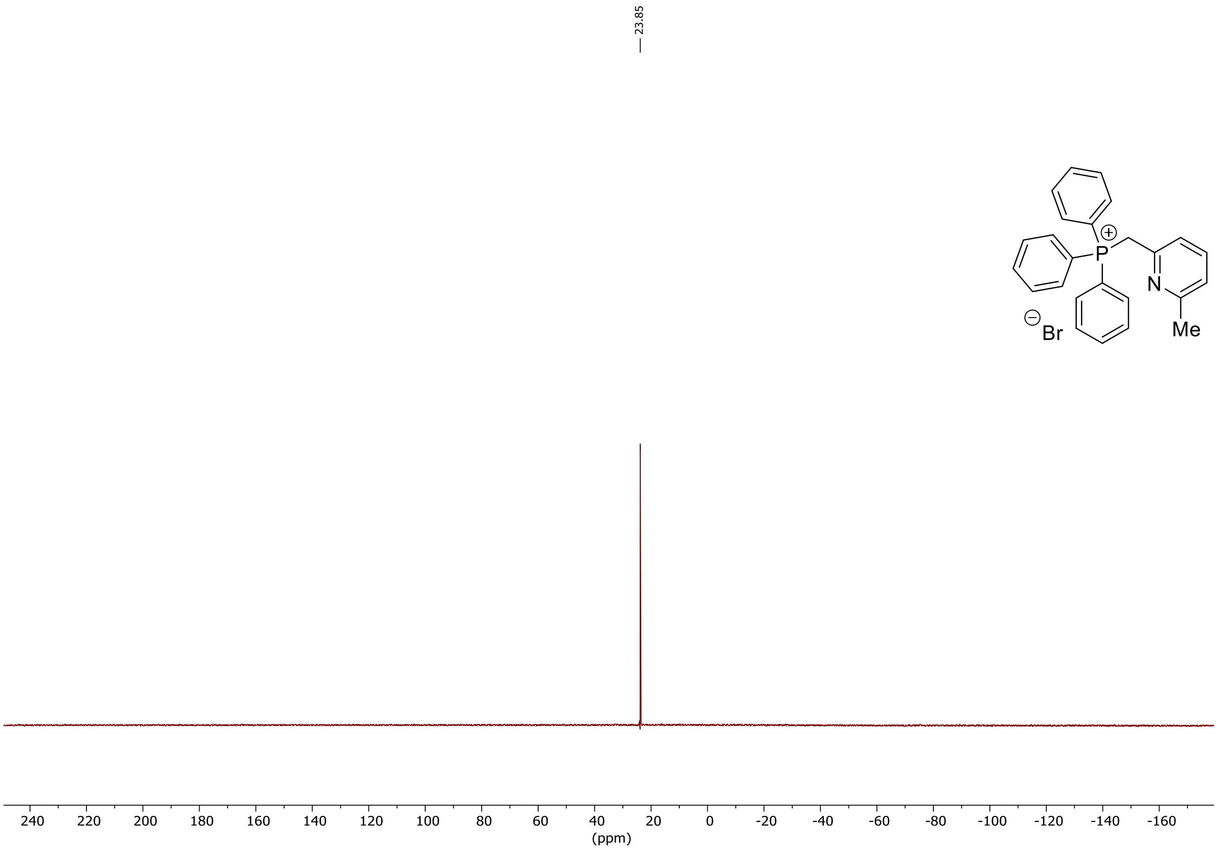

Fig. S68 ^{31}P NMR (202 MHz, dimethyl sulfoxide-d_6, 298 K) spectrum of compound **1e**

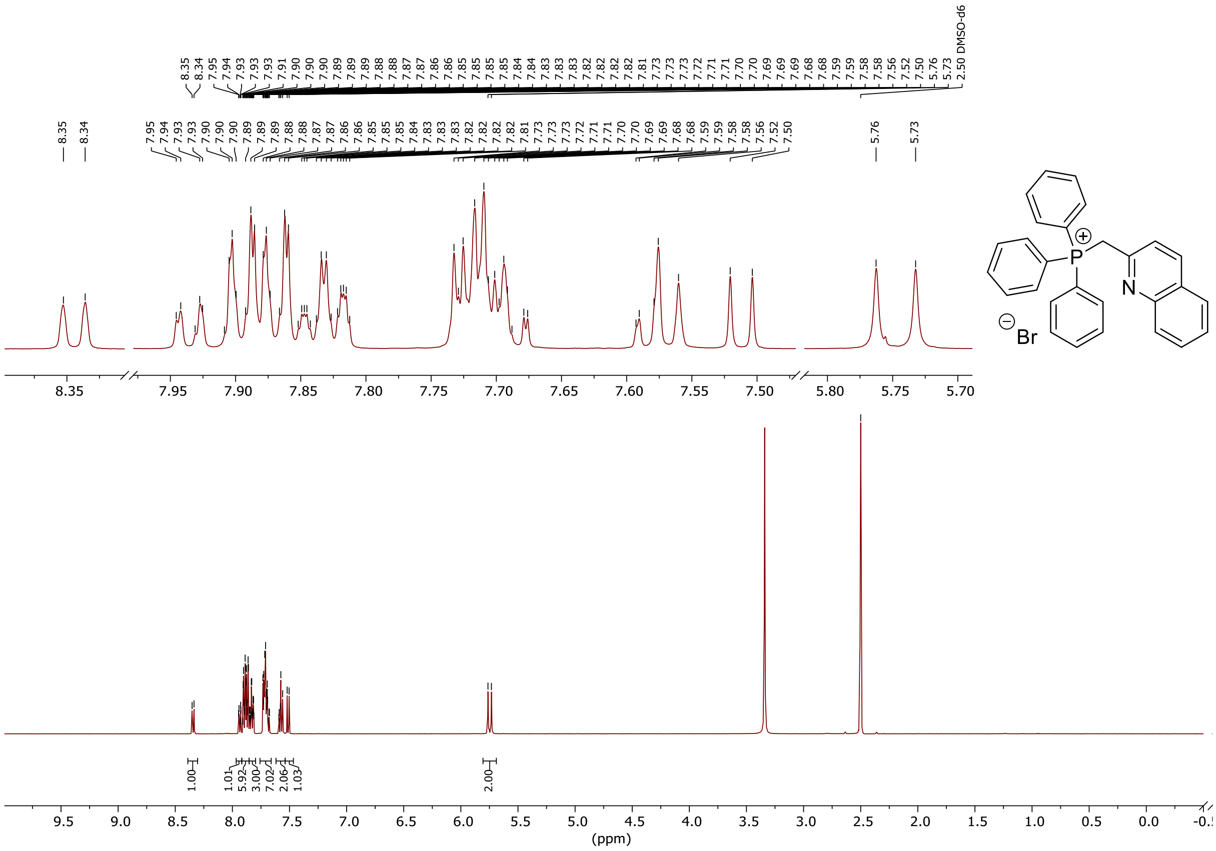

Fig. S69 ^{1}H NMR (501 MHz, dimethyl sulfoxide-d_6, 298 K) spectrum of compound **1f**

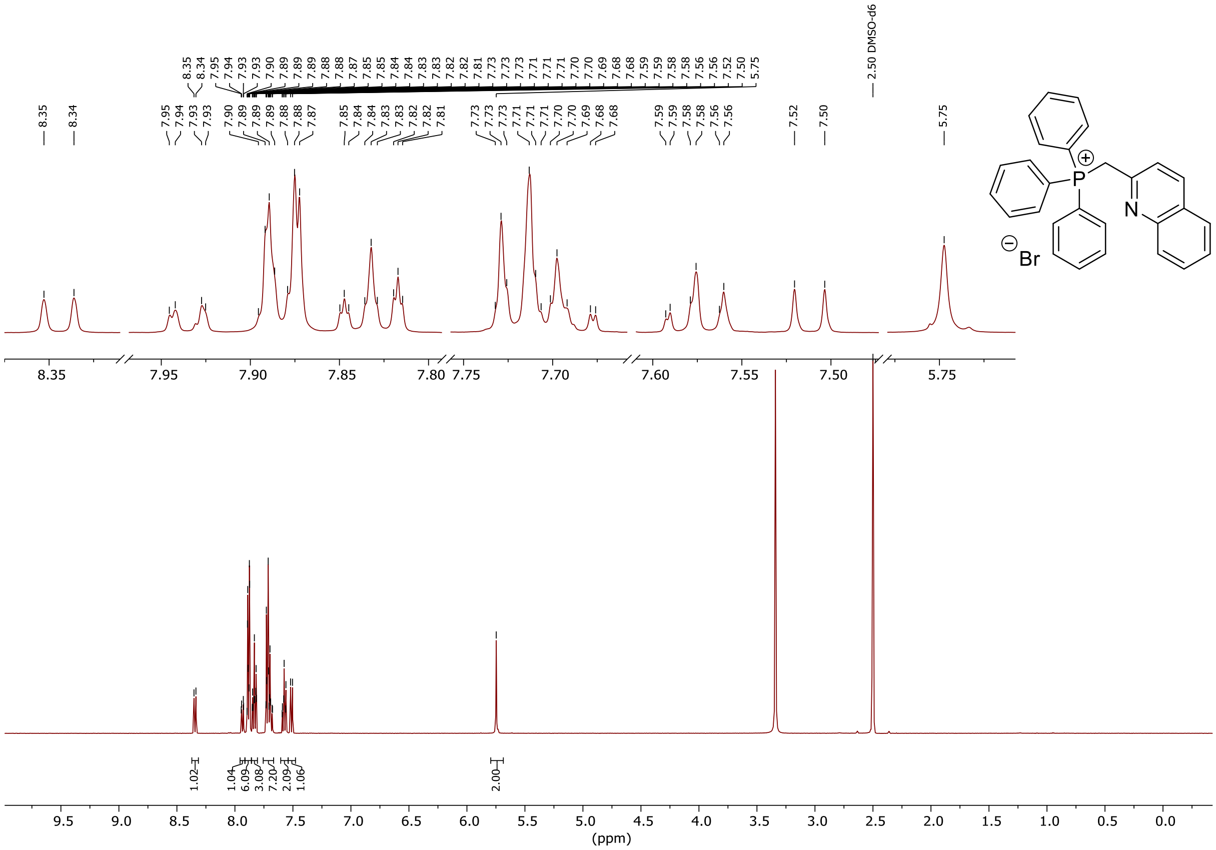

Fig. S70 $^{1}H\{^{31}P\}$ NMR (501 MHz, dimethyl sulfoxide-d_6, 298 K) spectrum of compound **1f**

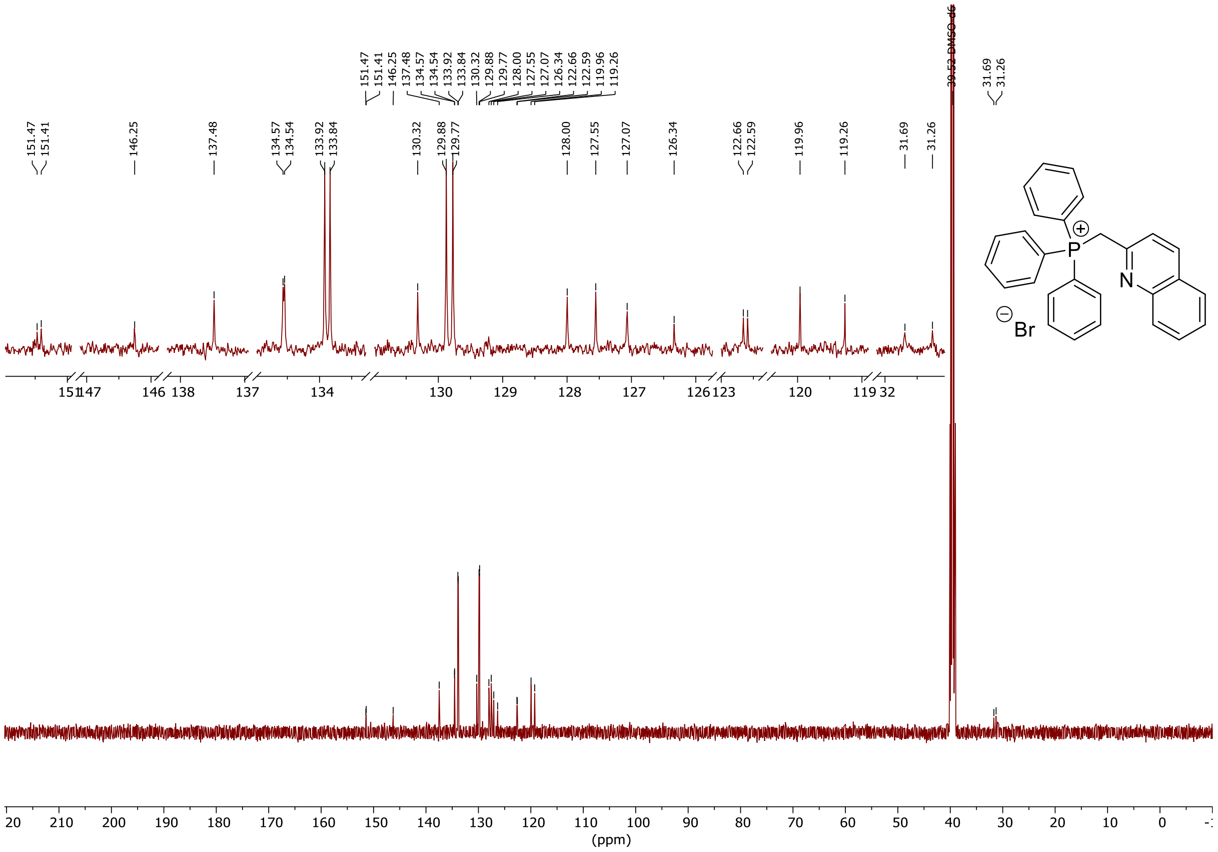

Fig. S71 $^{13}C\{^1H\}$ NMR (126 MHz, dimethyl sulfoxide-d_6, 298 K) spectrum of compound **1f**

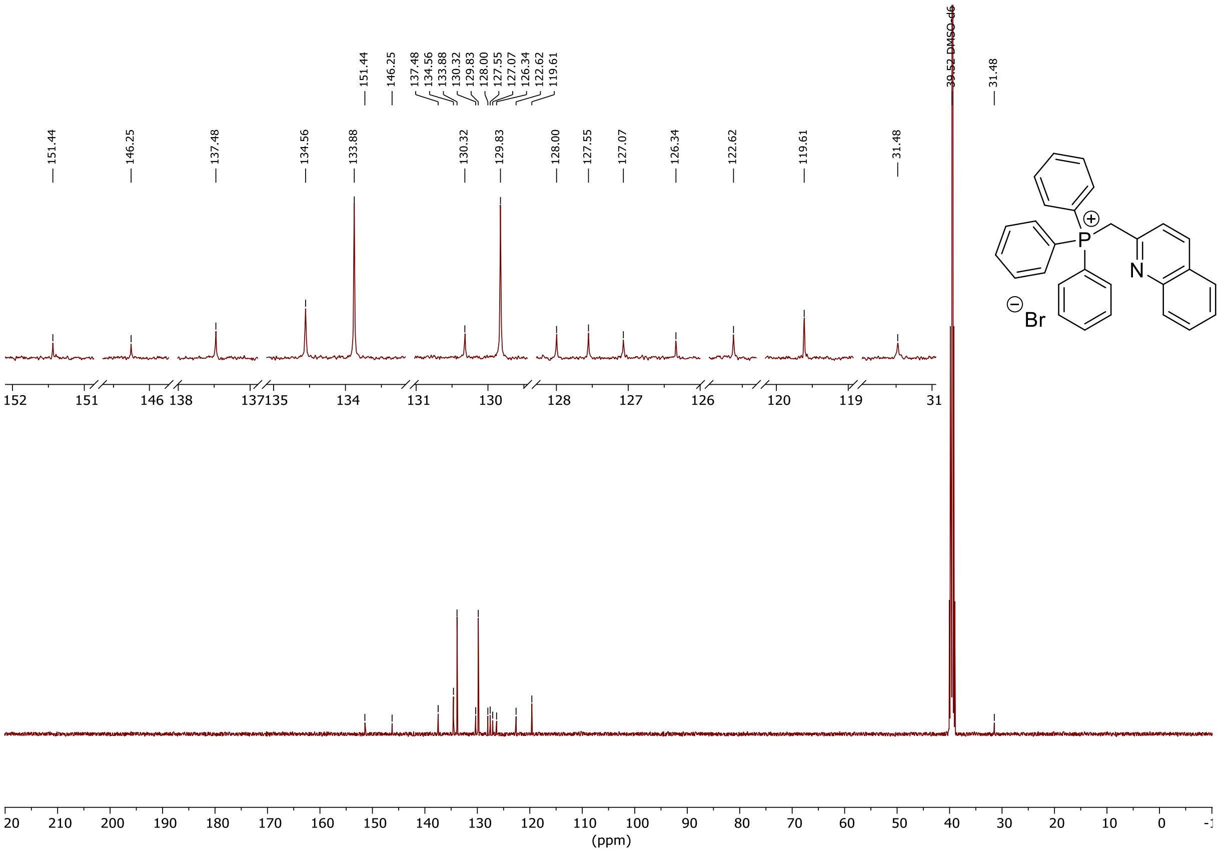

Fig. S72 $^{13}C\{^1H, ^{31}P\}$ NMR (126 MHz, dimethyl sulfoxide-d_6, 298 K) spectrum of compound **1f**

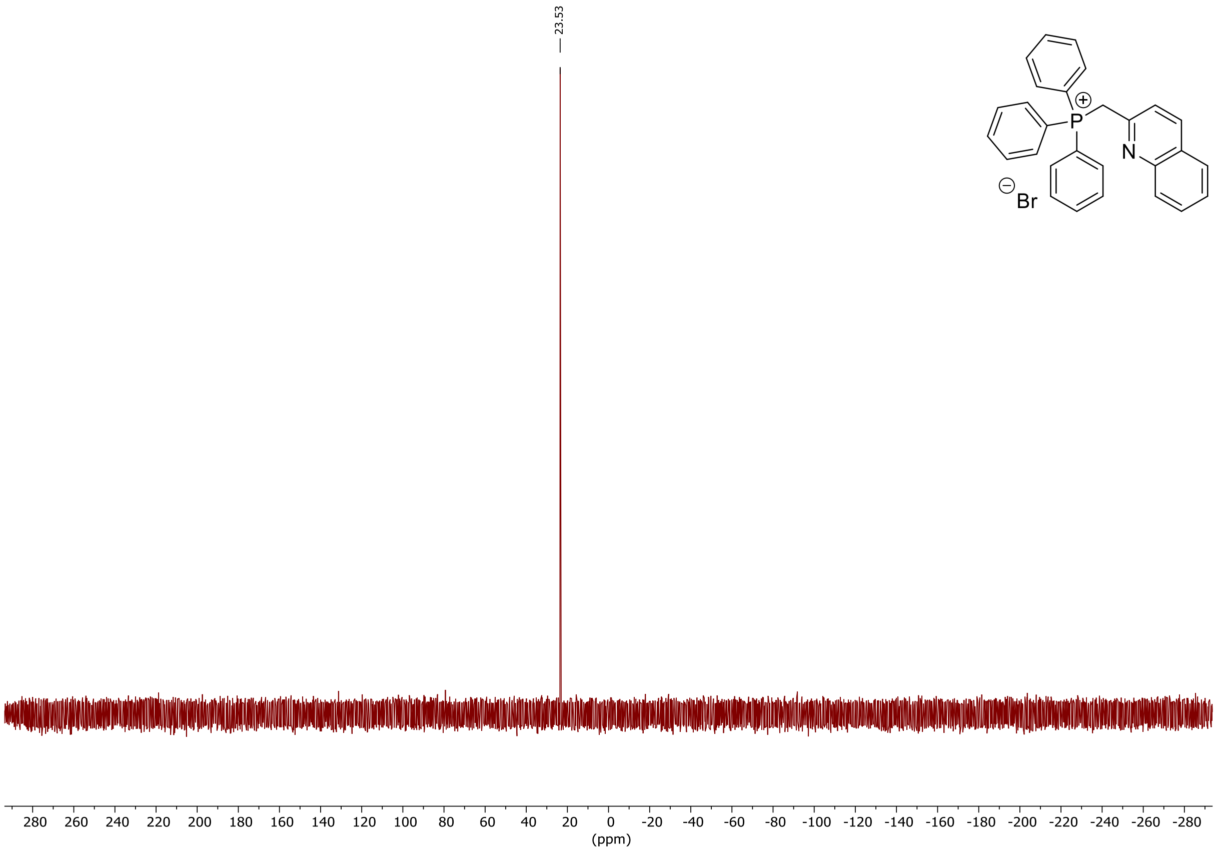

Fig. S73 ^{31}P NMR (202 MHz, dimethyl sulfoxide-d_6, 298 K) spectrum of compound **1f**

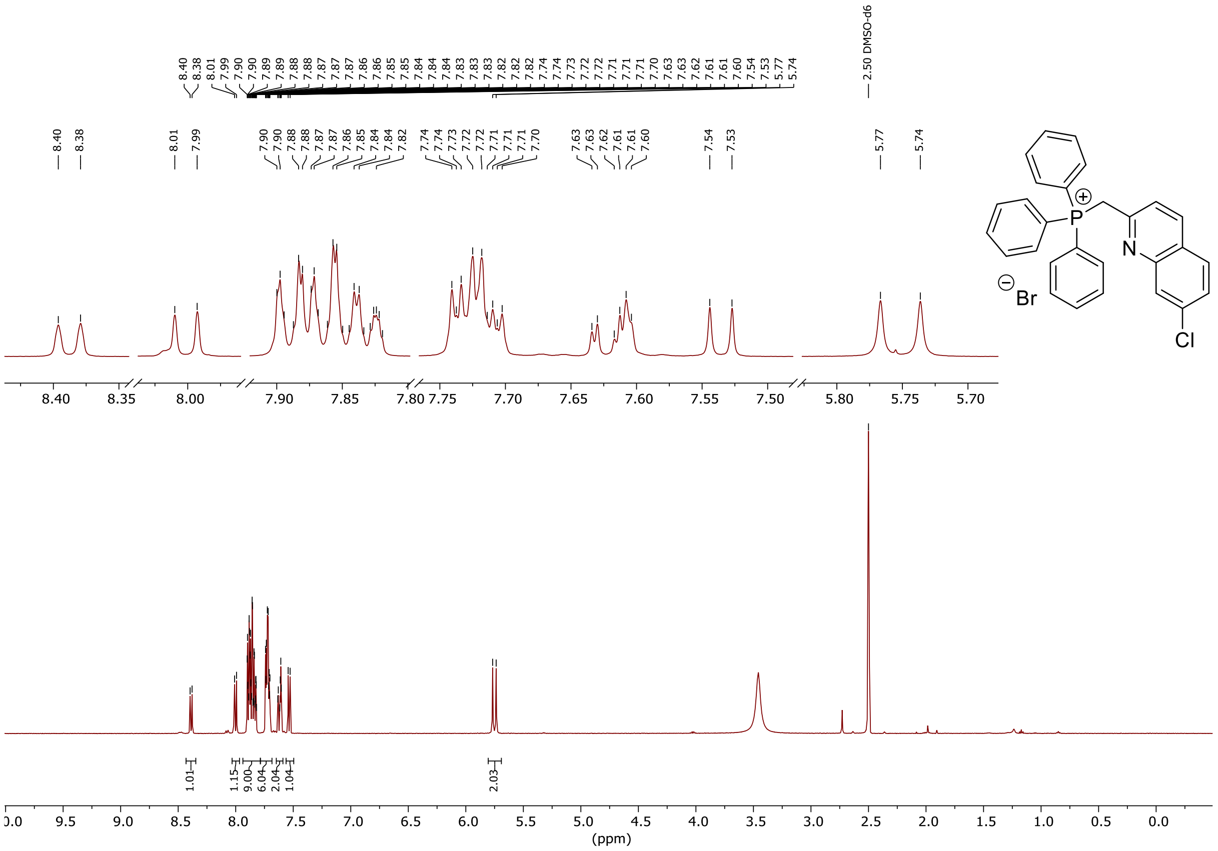

Fig. S74 ^{1}H NMR (501 MHz, dimethyl sulfoxide-d_6, 298 K) spectrum of compound **1g**

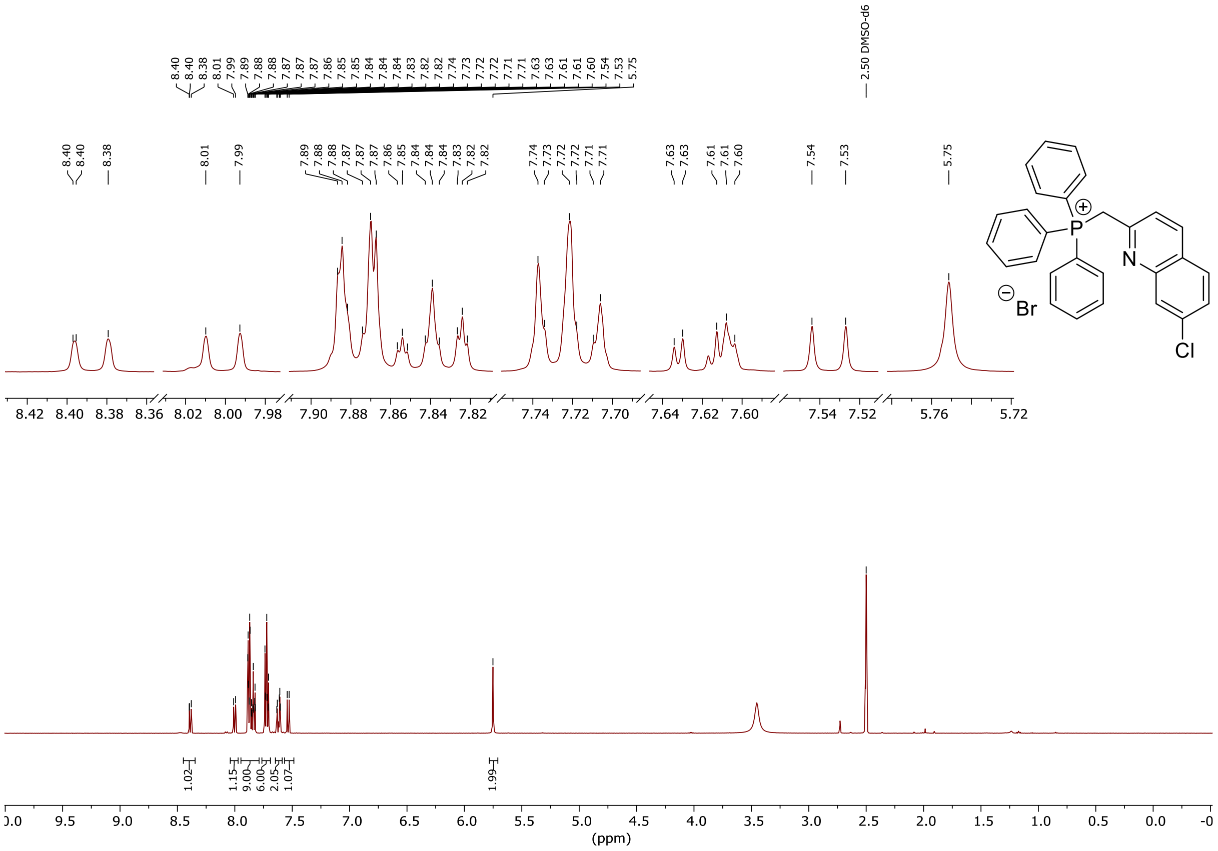

Fig. S75 ^{1}H{^{31}P} NMR (501 MHz, dimethyl sulfoxide-d_6, 298 K) spectrum of compound **1g**

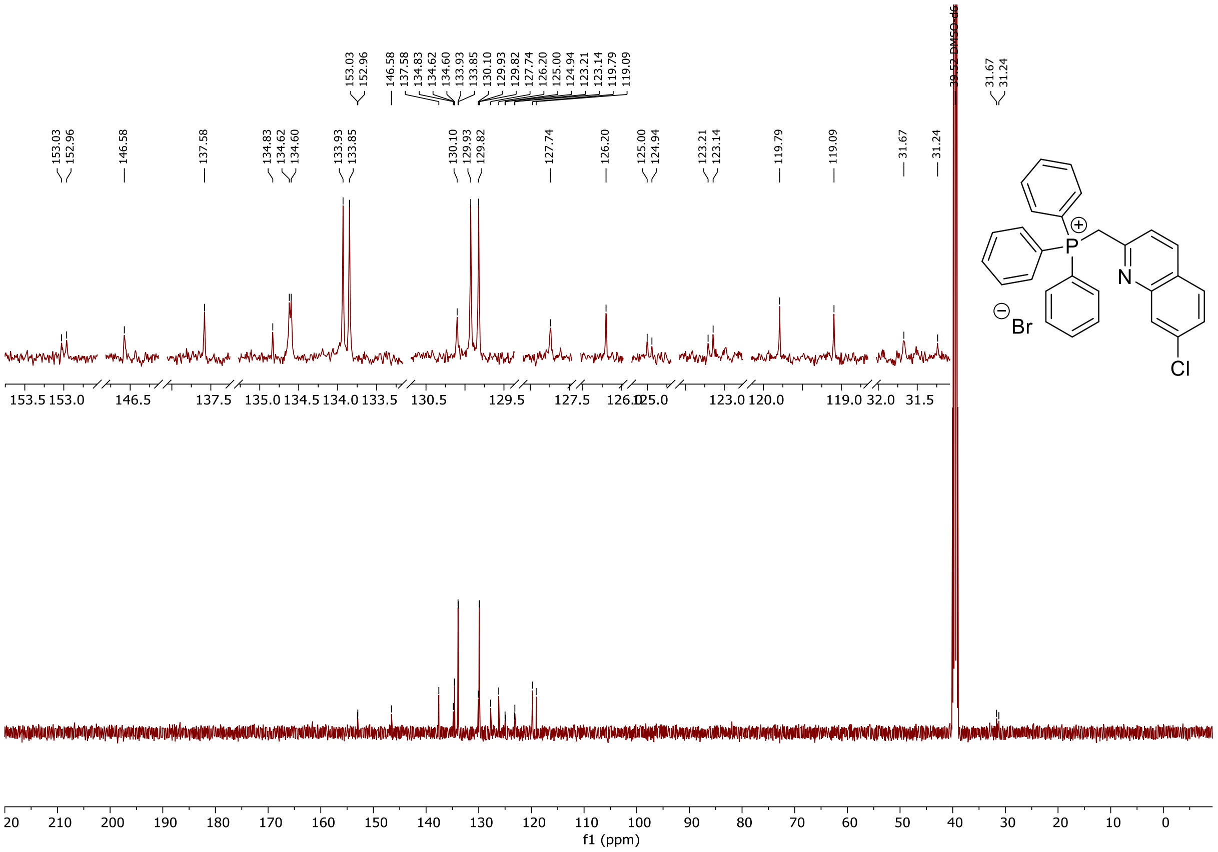

Fig. S76 $^{13}C\{^{1}H\}$ NMR (126 MHz, dimethyl sulfoxide-d_6, 298 K) spectrum of compound **1g**

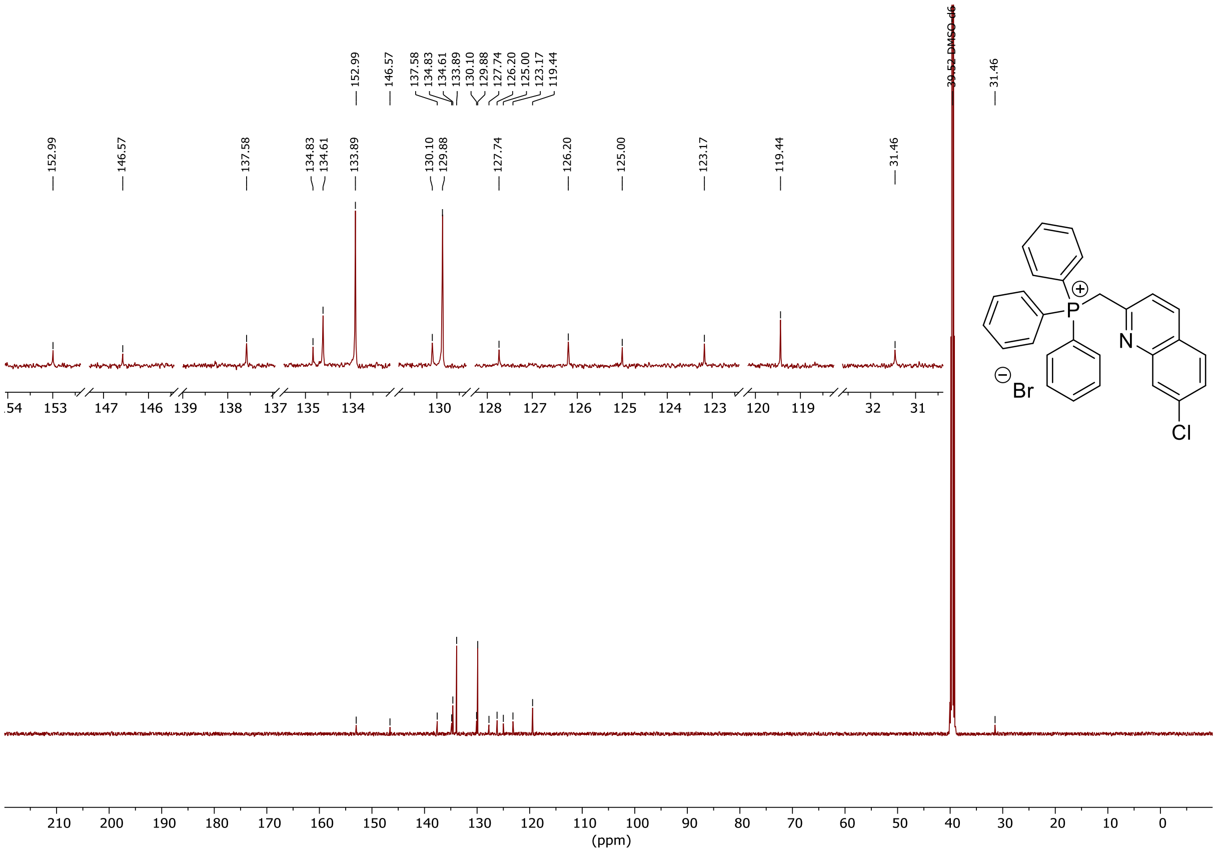

Fig. S77 $^{13}C\{^{1}H, ^{31}P\}$ NMR (126 MHz, dimethyl sulfoxide-d_6, 298 K) spectrum of compound **1g**

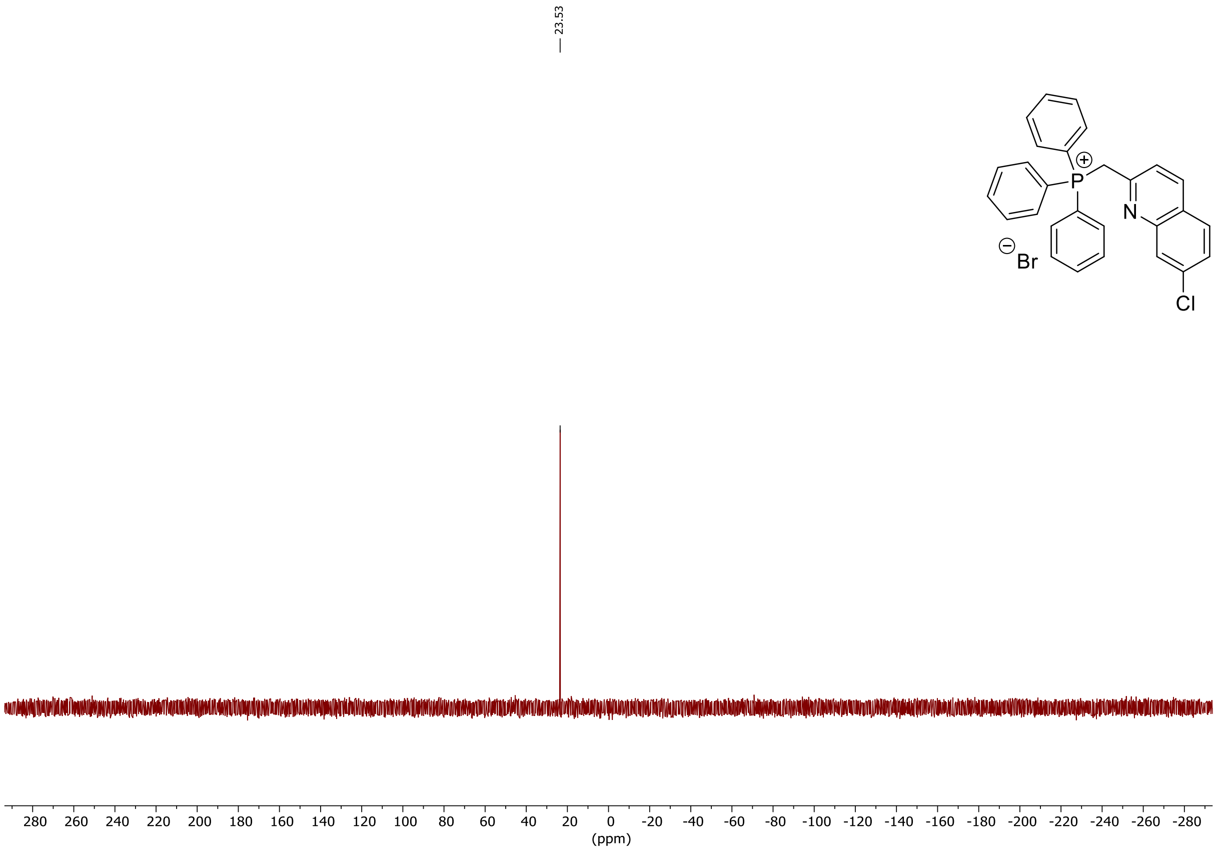

Fig. S78 ^{31}P NMR (202 MHz, dimethyl sulfoxide-d_6, 298 K) spectrum of compound **1g**

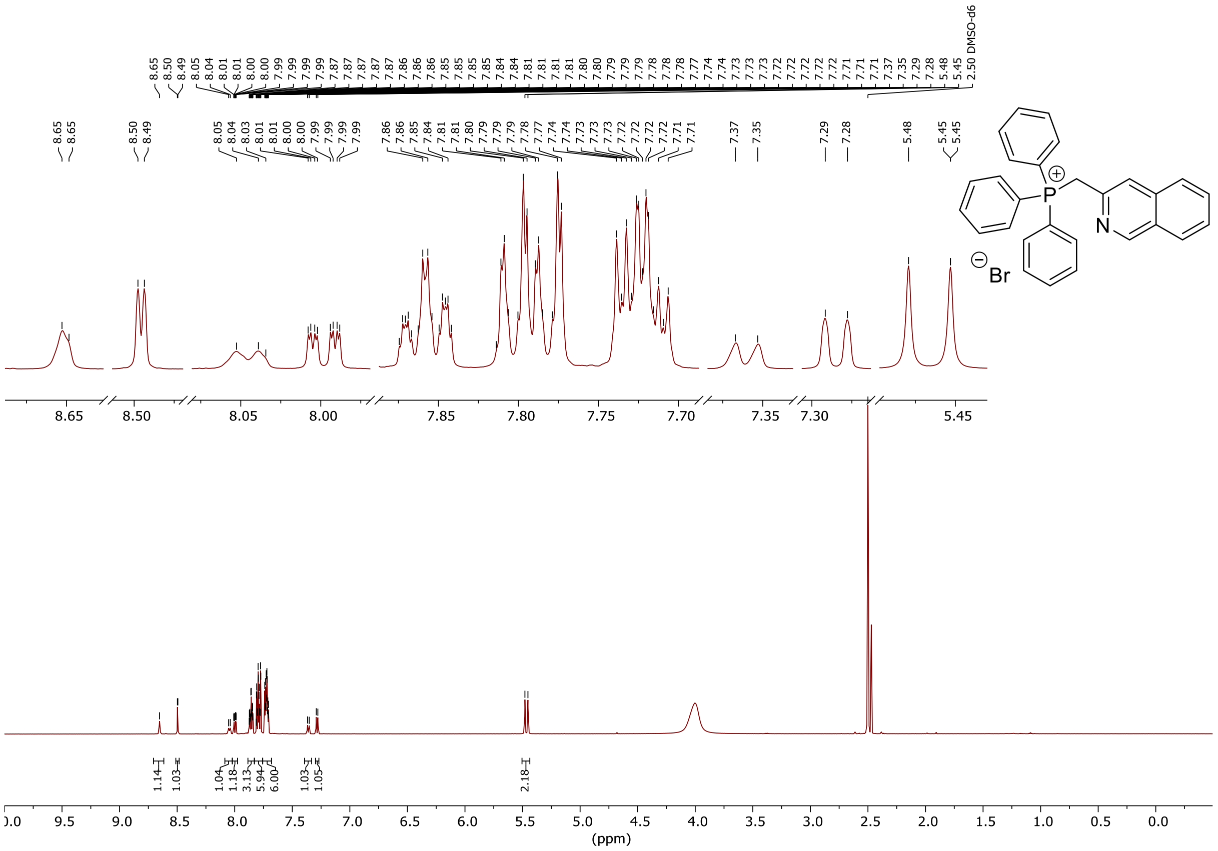

Fig. S79 ^{1}H NMR(501 MHz, dimethyl sulfoxide-d_6, 298 K) spectrum of compound **1h**

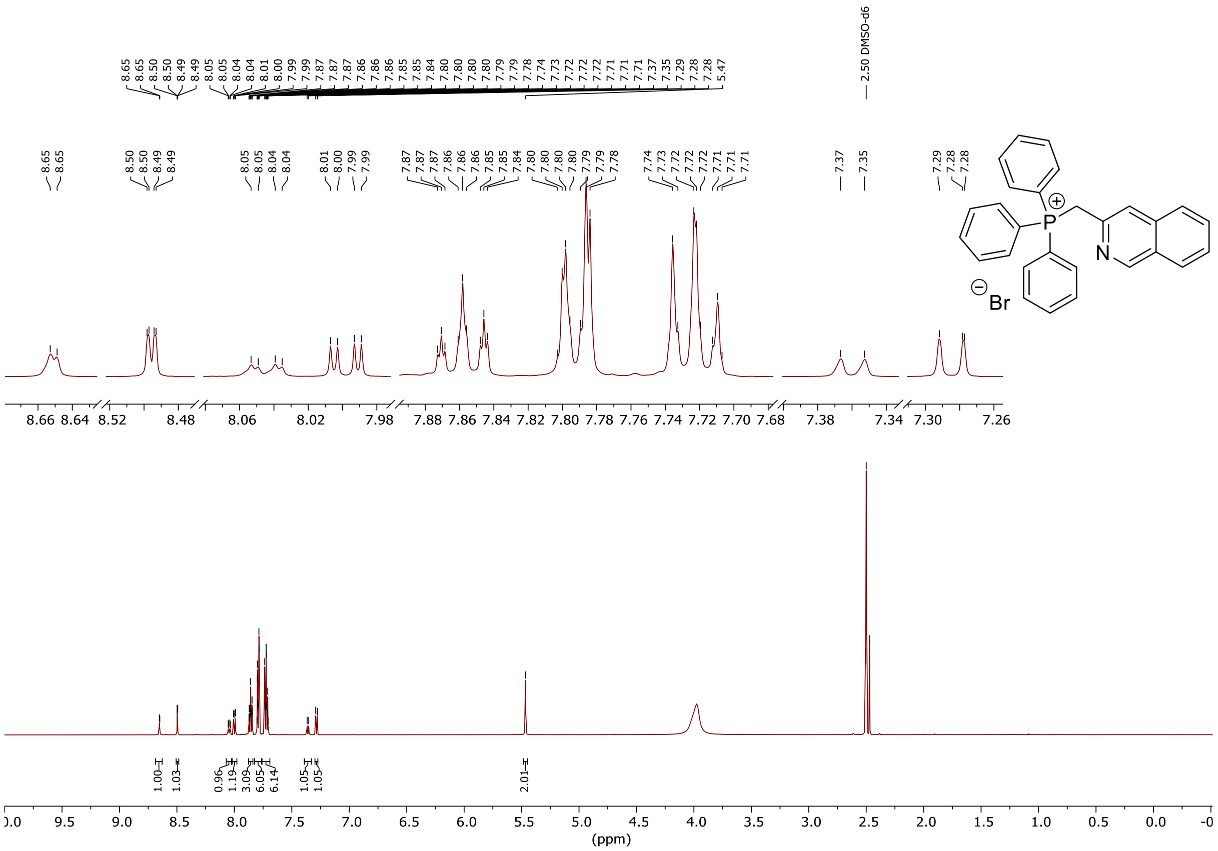

Fig. S80 ^{1}H{^{31}P} NMR (501 MHz, dimethyl sulfoxide-d_6, 298 K) spectrum of compound **1h**

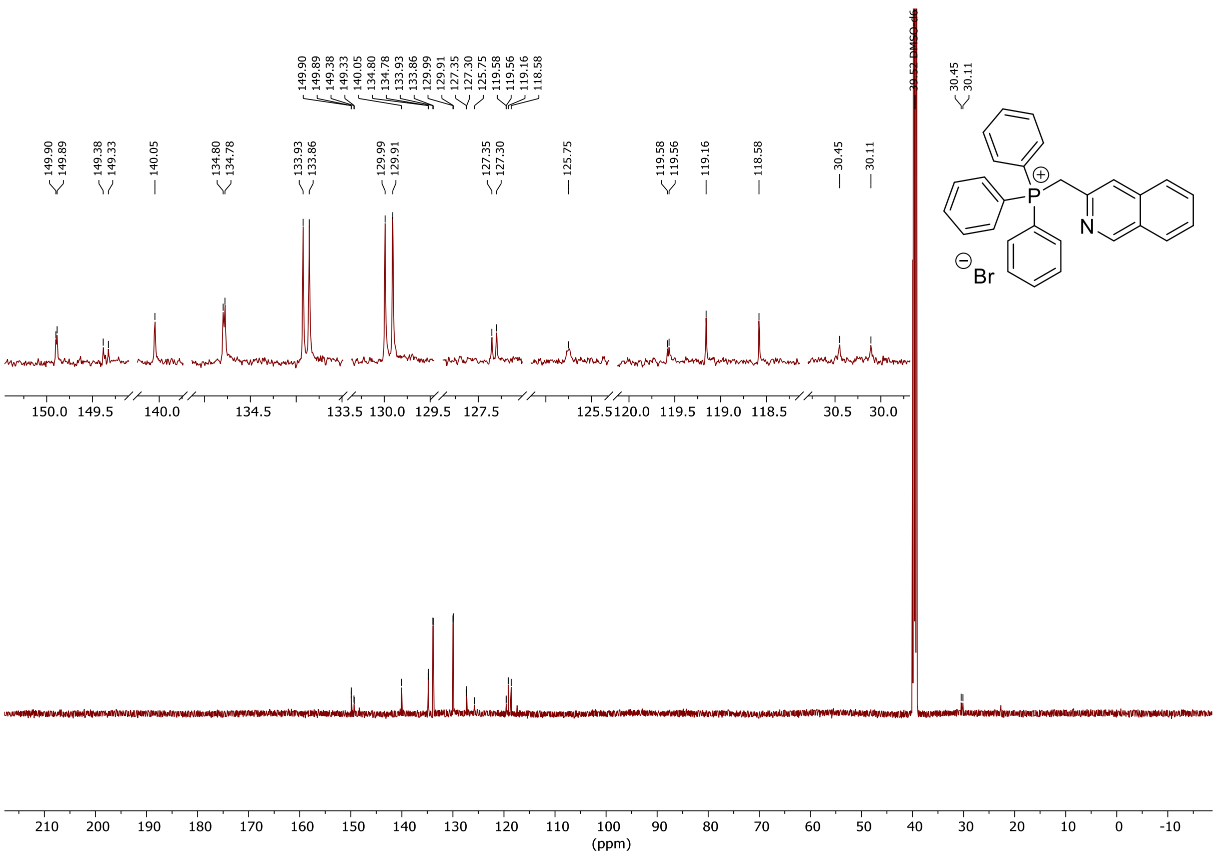

Fig. S81 $^{13}C\{^{1}H\}$ NMR (126 MHz, dimethyl sulfoxide-d_6, 298 K) spectrum of compound **1h**

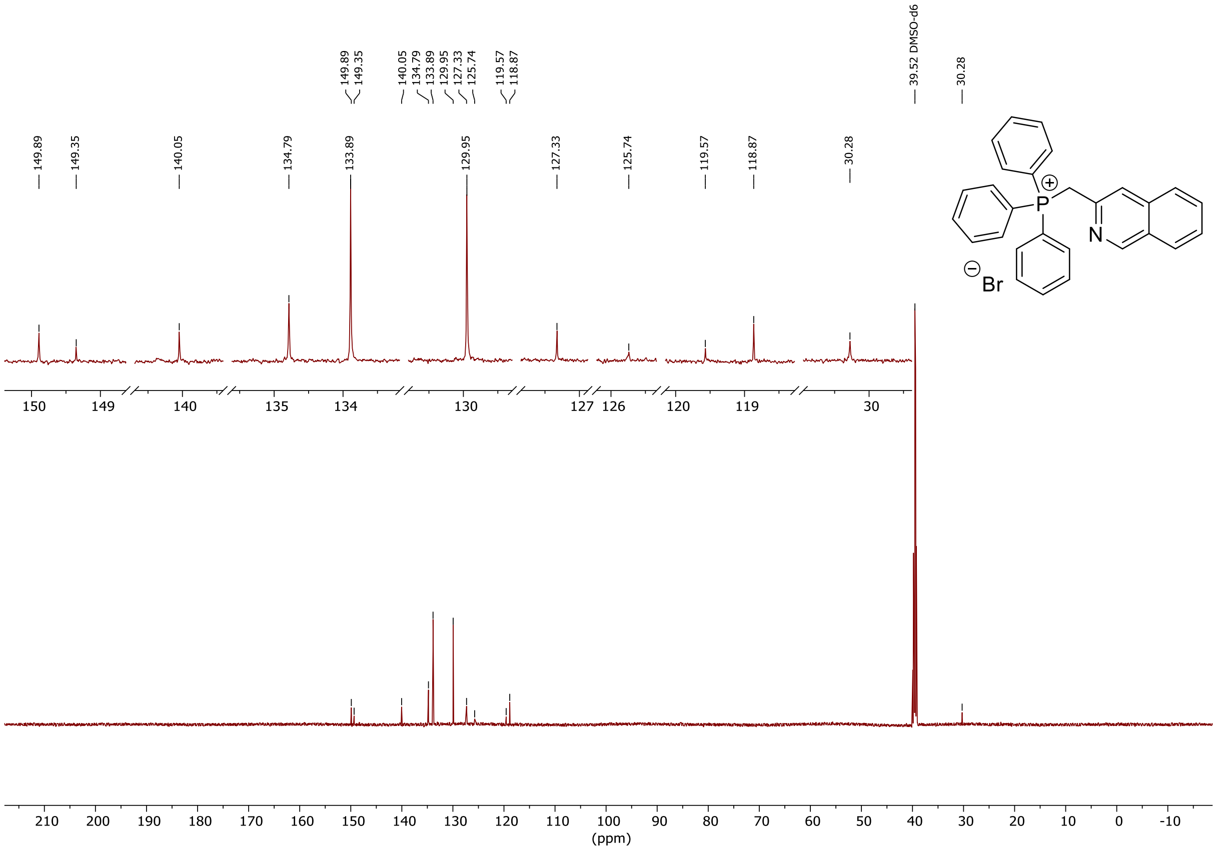

Fig. S82 $^{13}C\{^{31}P\}$ NMR (126 MHz, dimethyl sulfoxide-d_6, 298 K) spectrum of compound **1h**

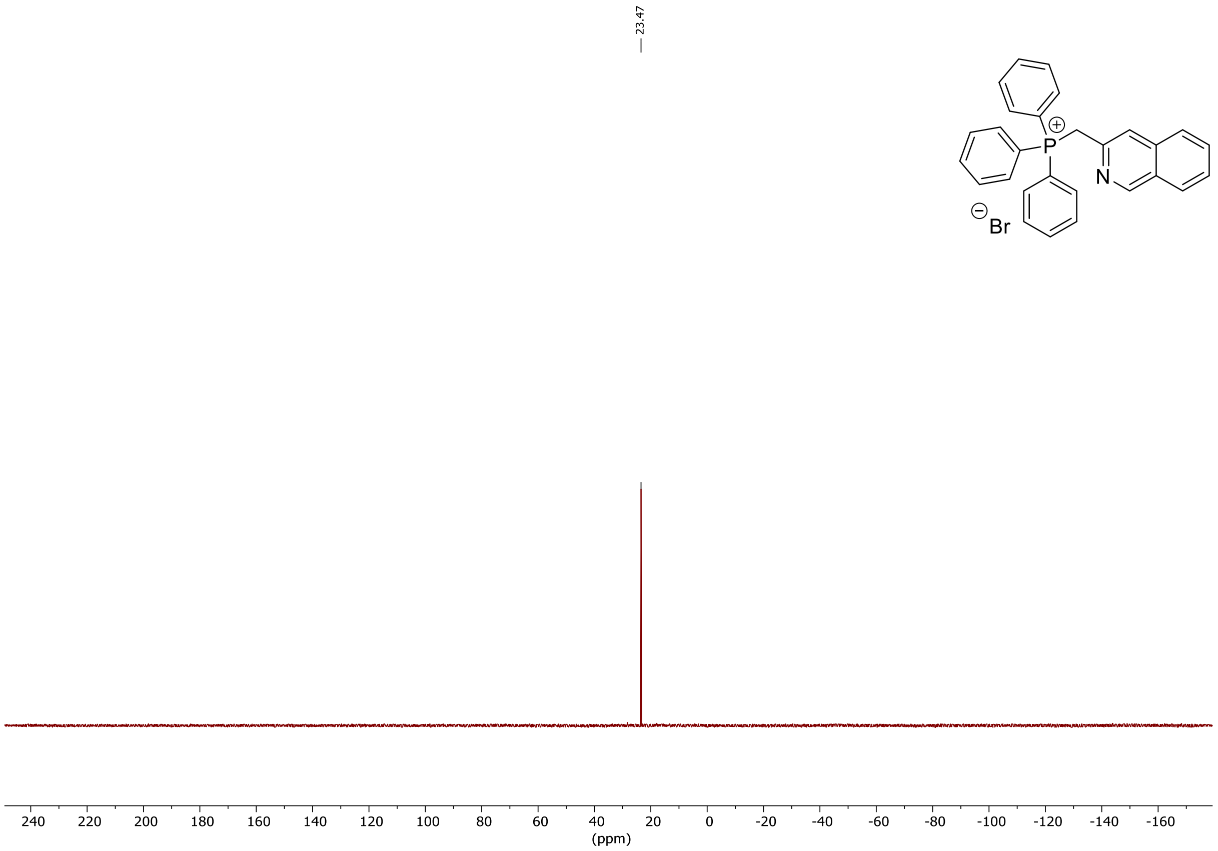

Fig. S83 ^{31}P NMR (202 MHz, dimethyl sulfoxide-d_6, 298 K) spectrum of compound **1h**

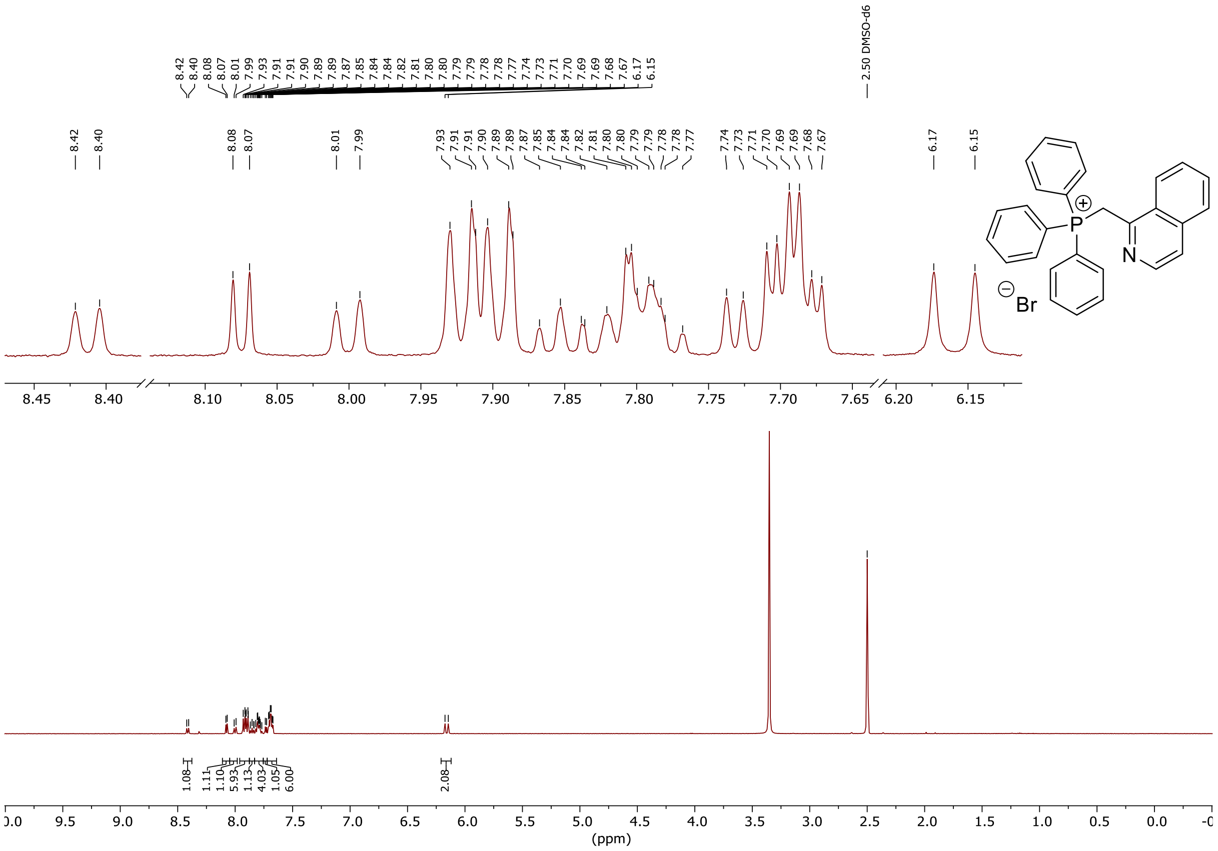

Fig. S84 ^{1}H NMR (501 MHz, dimethyl sulfoxide-d_6, 298 K) spectrum of compound **1i**

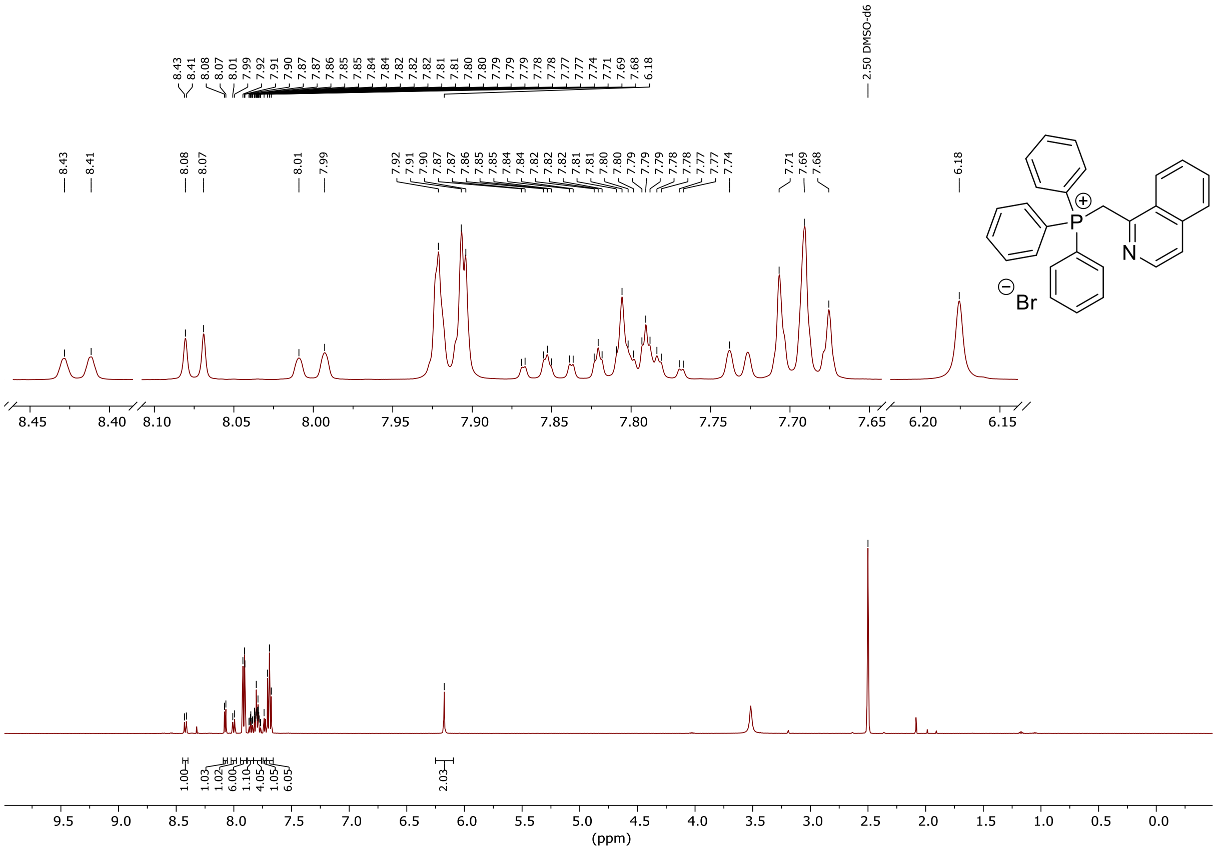

Fig. S85 ^{1}H{^{31}P} NMR (501 MHz, dimethyl sulfoxide-d_6, 298 K) spectrum of compound **1i**

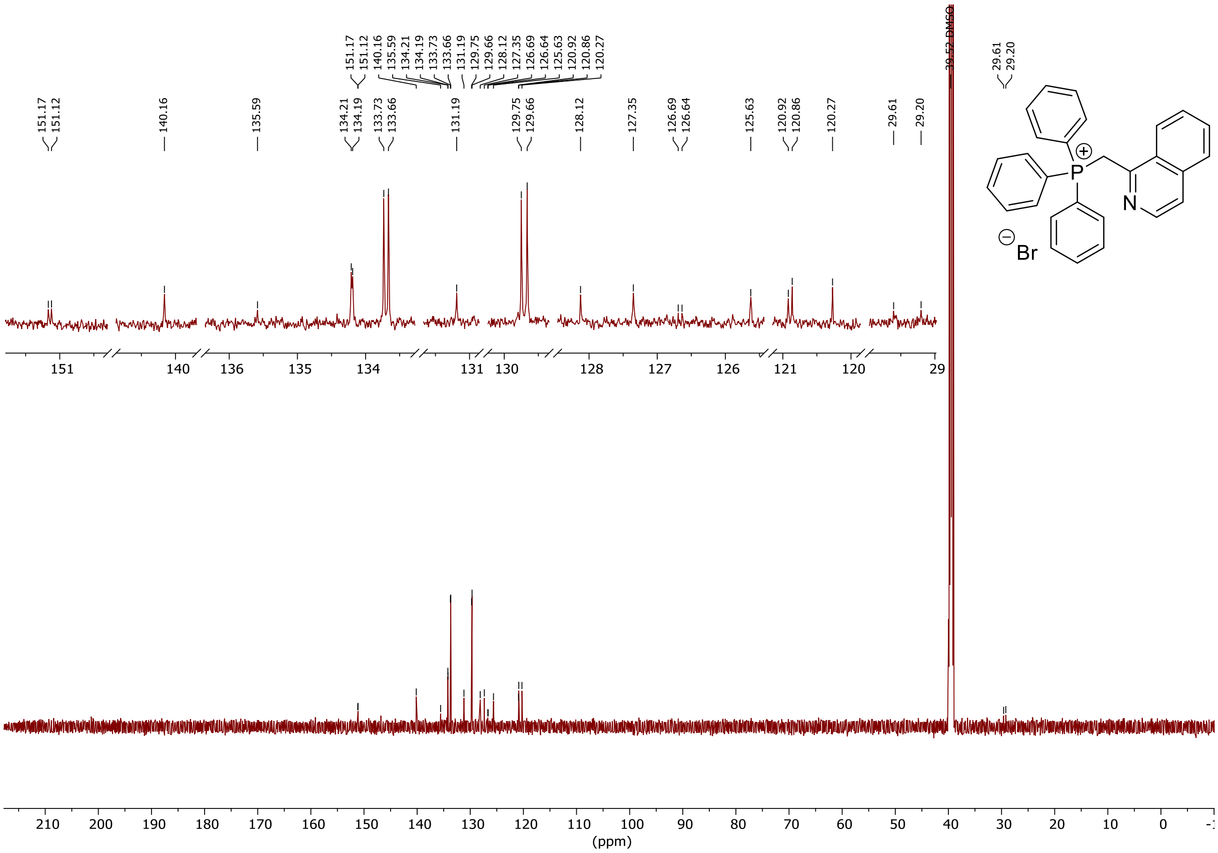

Fig. S86 $^{13}C\{^{1}H\}$ NMR (126 MHz, dimethyl sulfoxide-d_6, 298 K) spectrum of compound **1i**

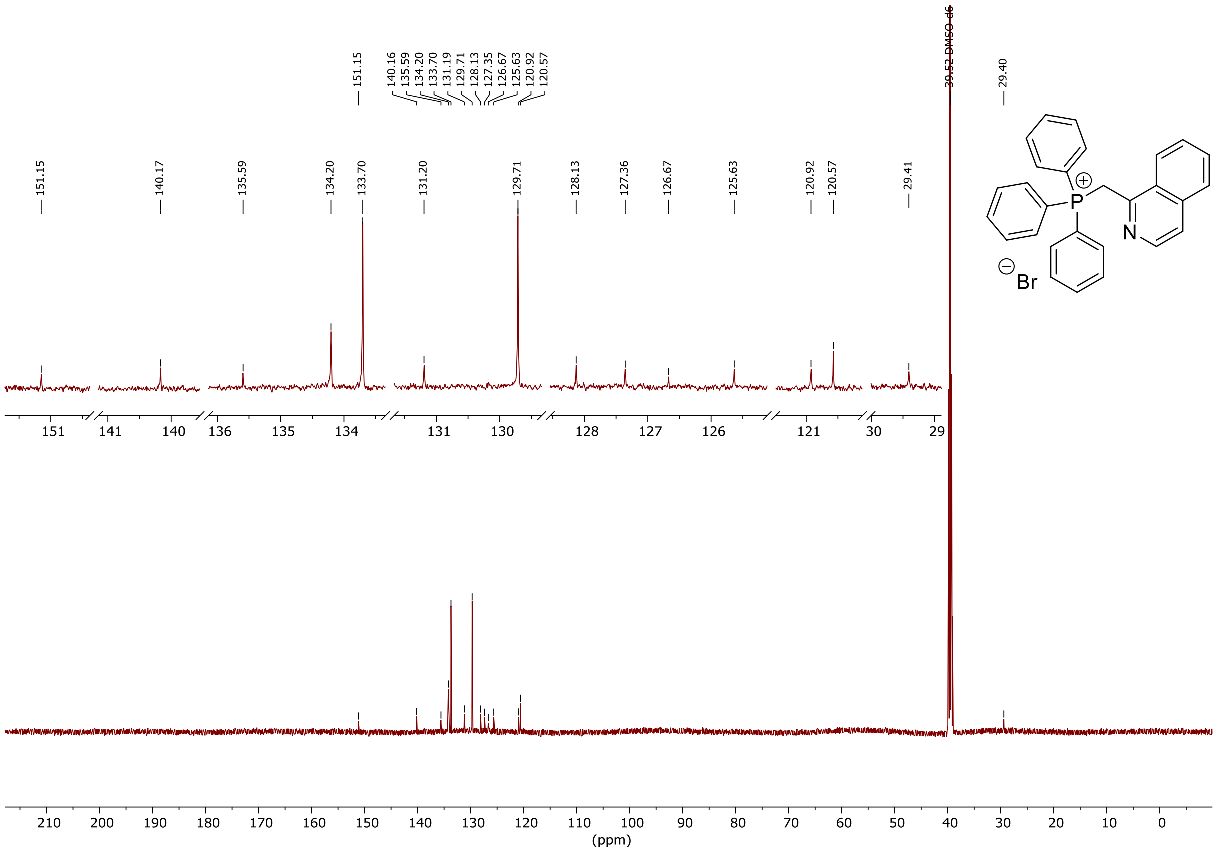

Fig. S87 $^{13}C\{^{1}H,^{31}P\}$ NMR (126 MHz, dimethyl sulfoxide-d_6, 298 K) spectrum of compound **1i**

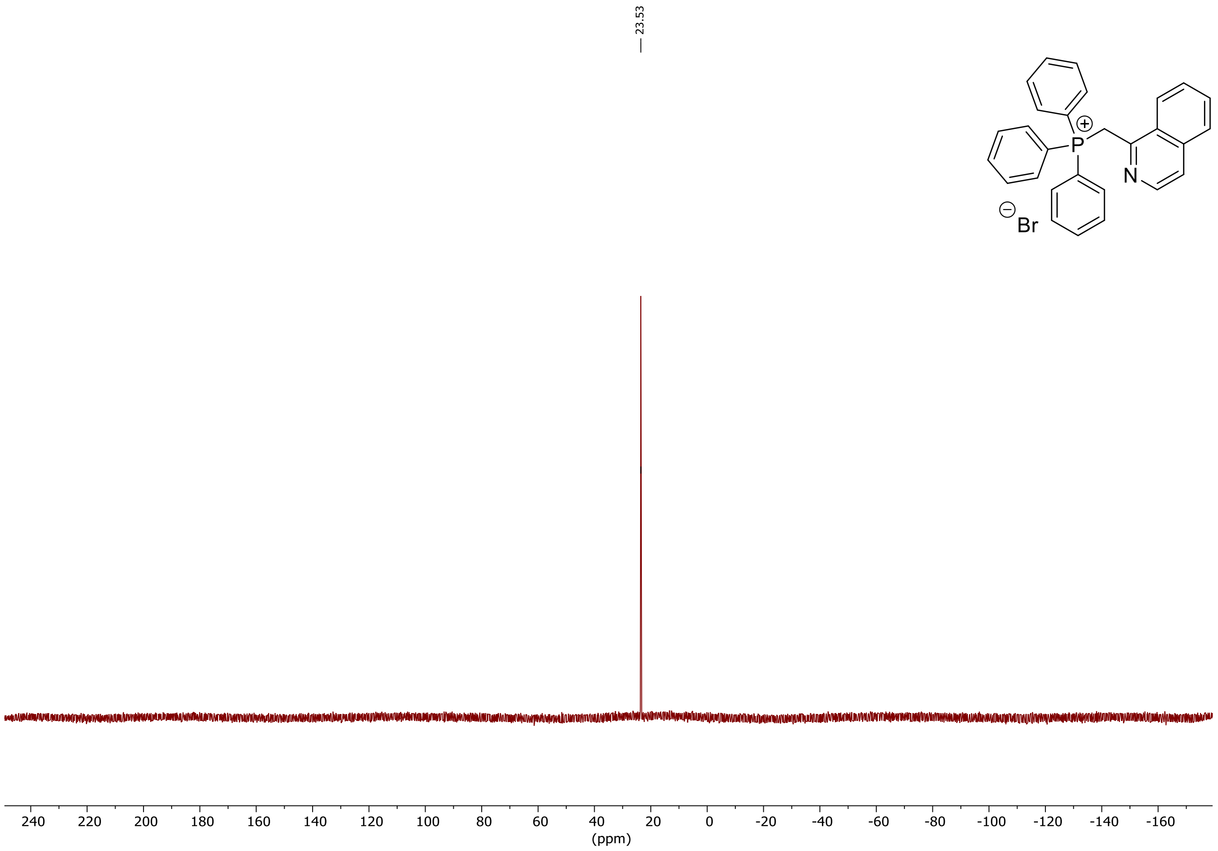

Fig. S88 ^{31}P NMR (202 MHz, dimethyl sulfoxide-d_6, 298 K) spectrum of compound **1i**

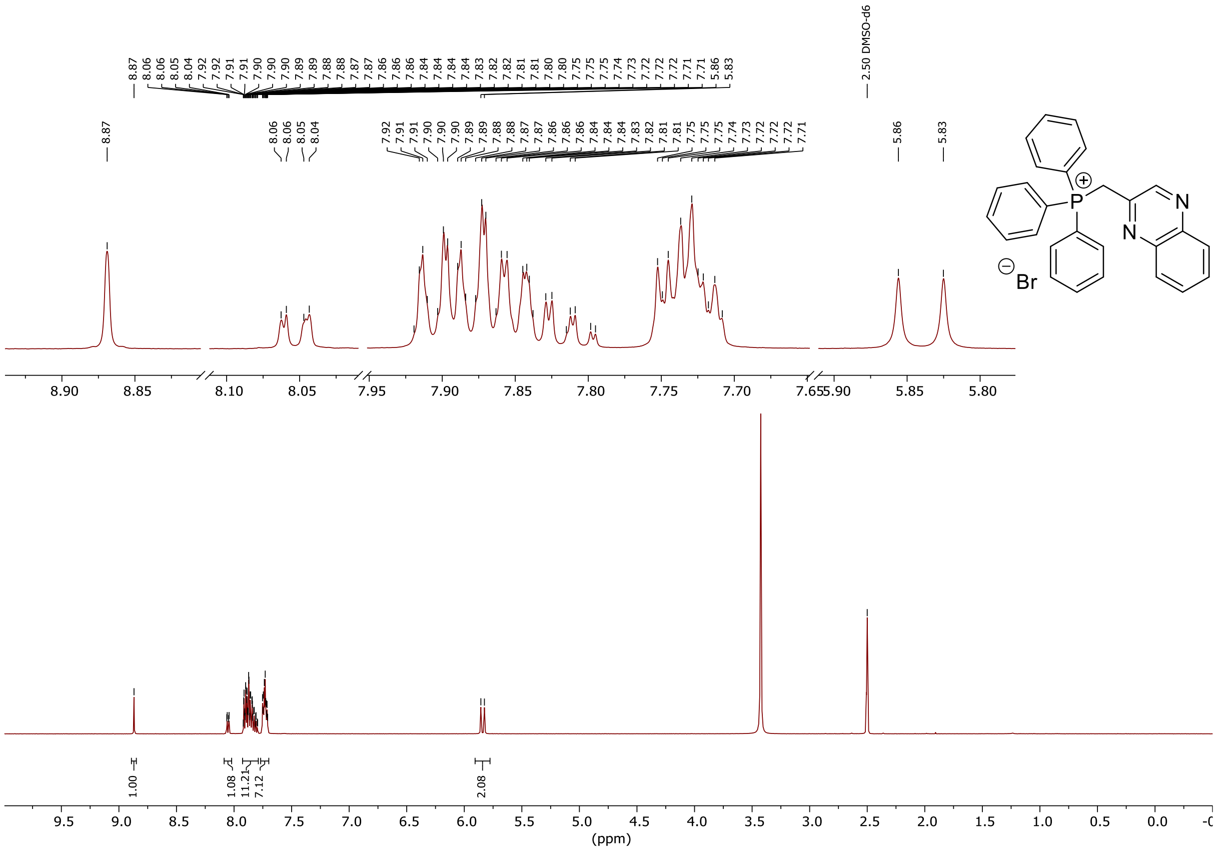

Fig. S89 ^{1}H NMR (501 MHz, dimethyl sulfoxide-d_6, 298 K) spectrum of compound **1j**

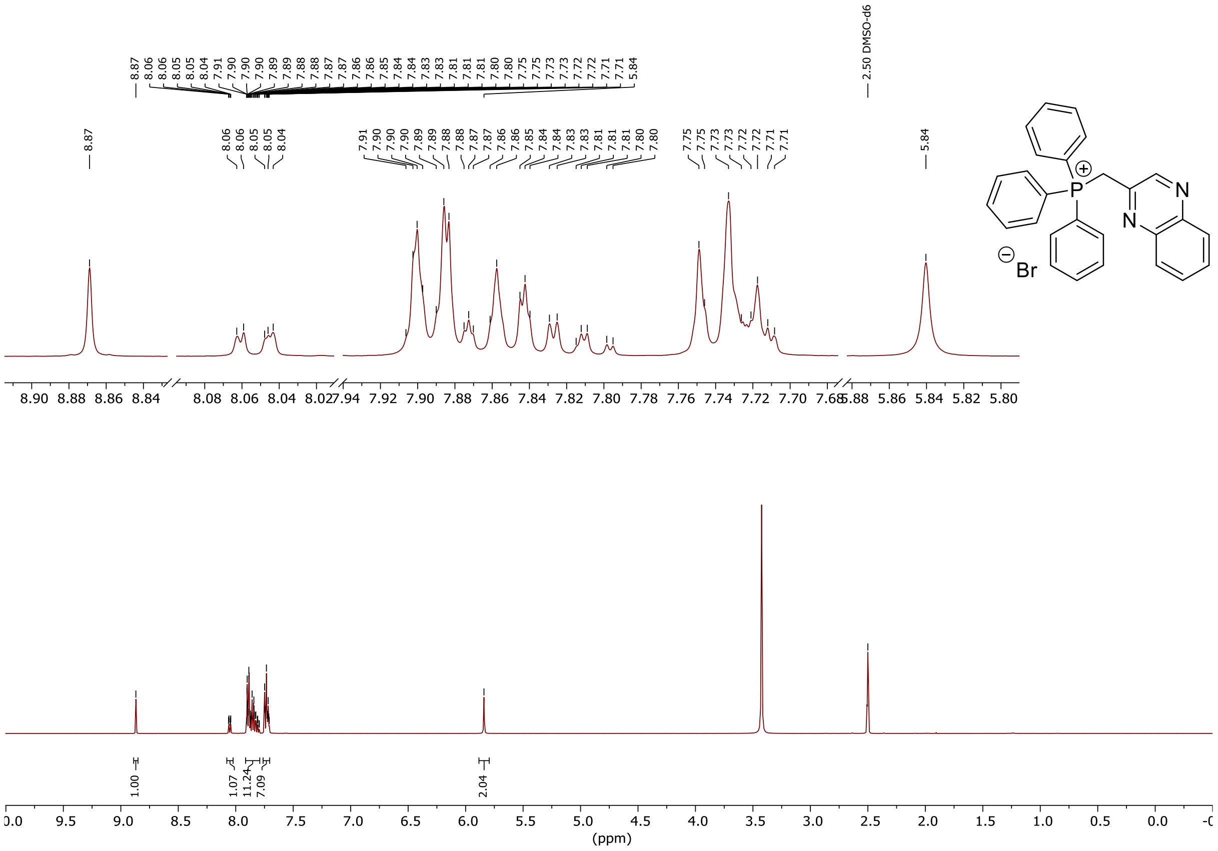

Fig. S90 ^{1}H{^{31}P} NMR (501 MHz, dimethyl sulfoxide-d_6, 298 K) spectrum of compound **1j**

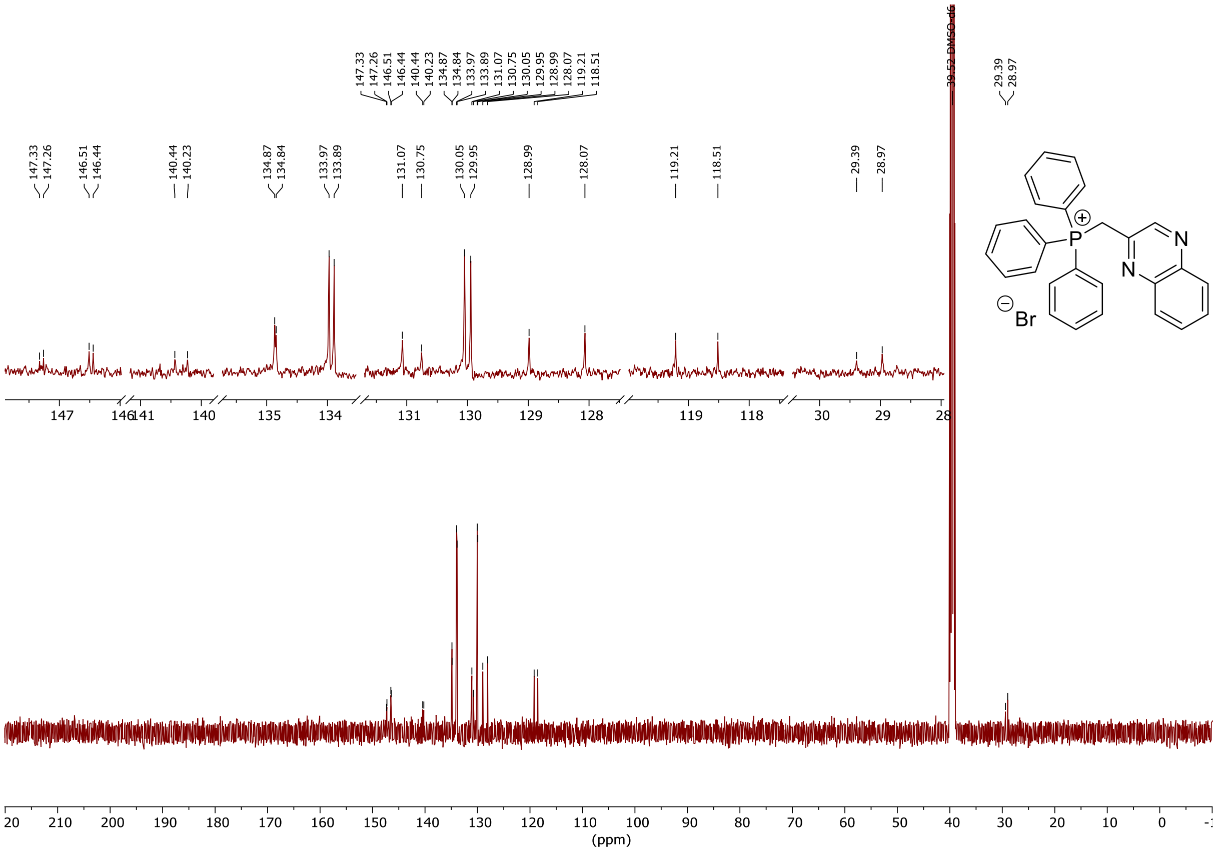

Fig. S91 $^{13}C\{^1H\}$ NMR (126 MHz, dimethyl sulfoxide-d_6, 298 K) spectrum of compound **1j**

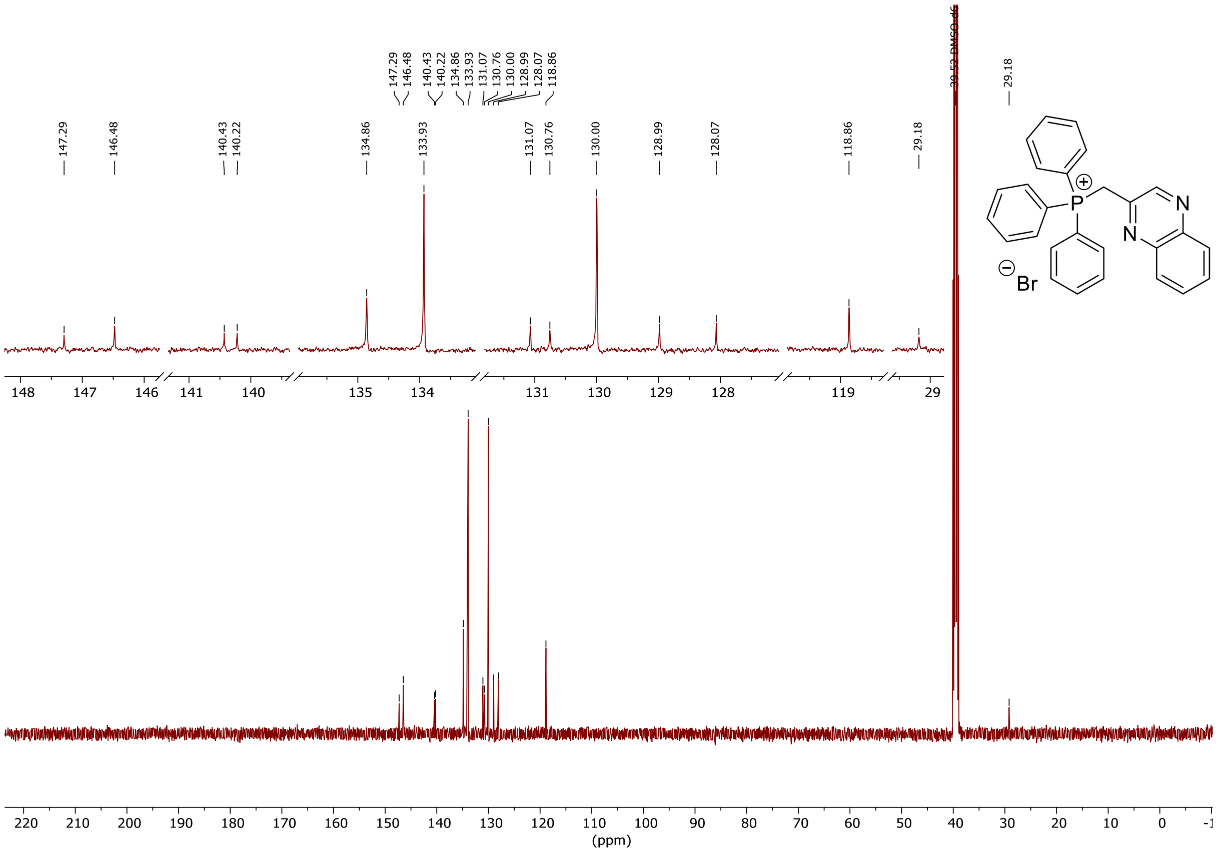

Fig. S92 $^{13}C\{^1H,^{31}P\}$ NMR (126 MHz, dimethyl sulfoxide-d_6, 298 K) spectrum of compound **1j**

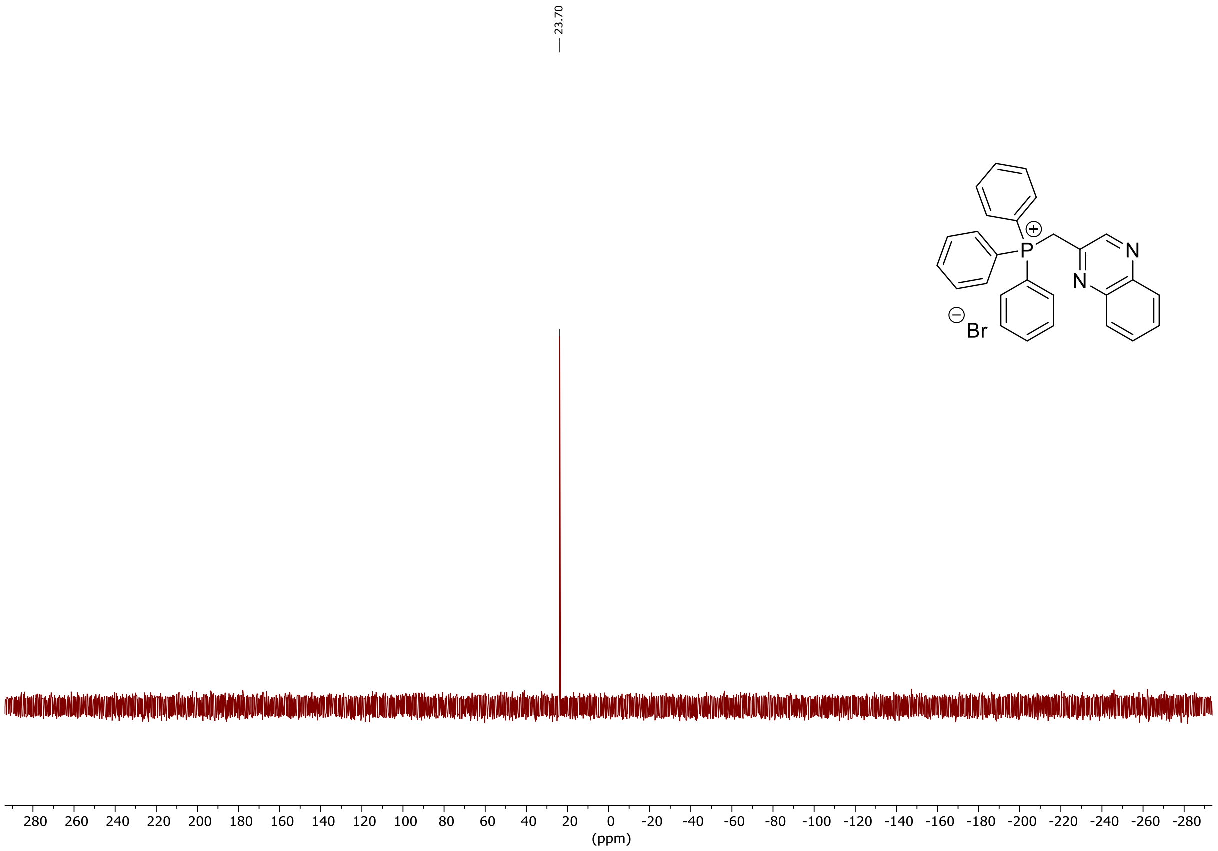

Fig. S93 ^{31}P NMR (202 MHz, dimethyl sulfoxide-d_6, 298 K) spectrum of compound **1j**

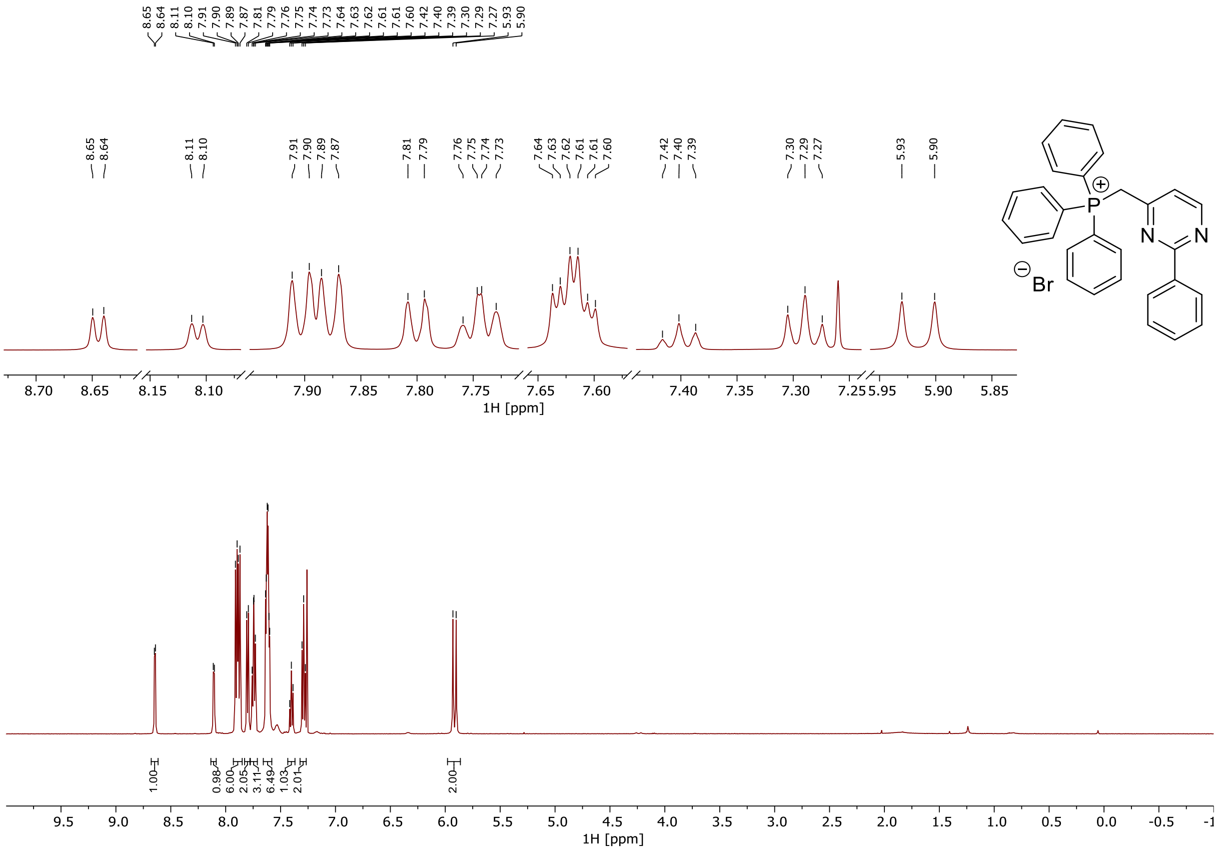

Fig. S94 ^{1}H NMR (501 MHz, chloroform-*d*, 298 K) spectrum of compound **1o**

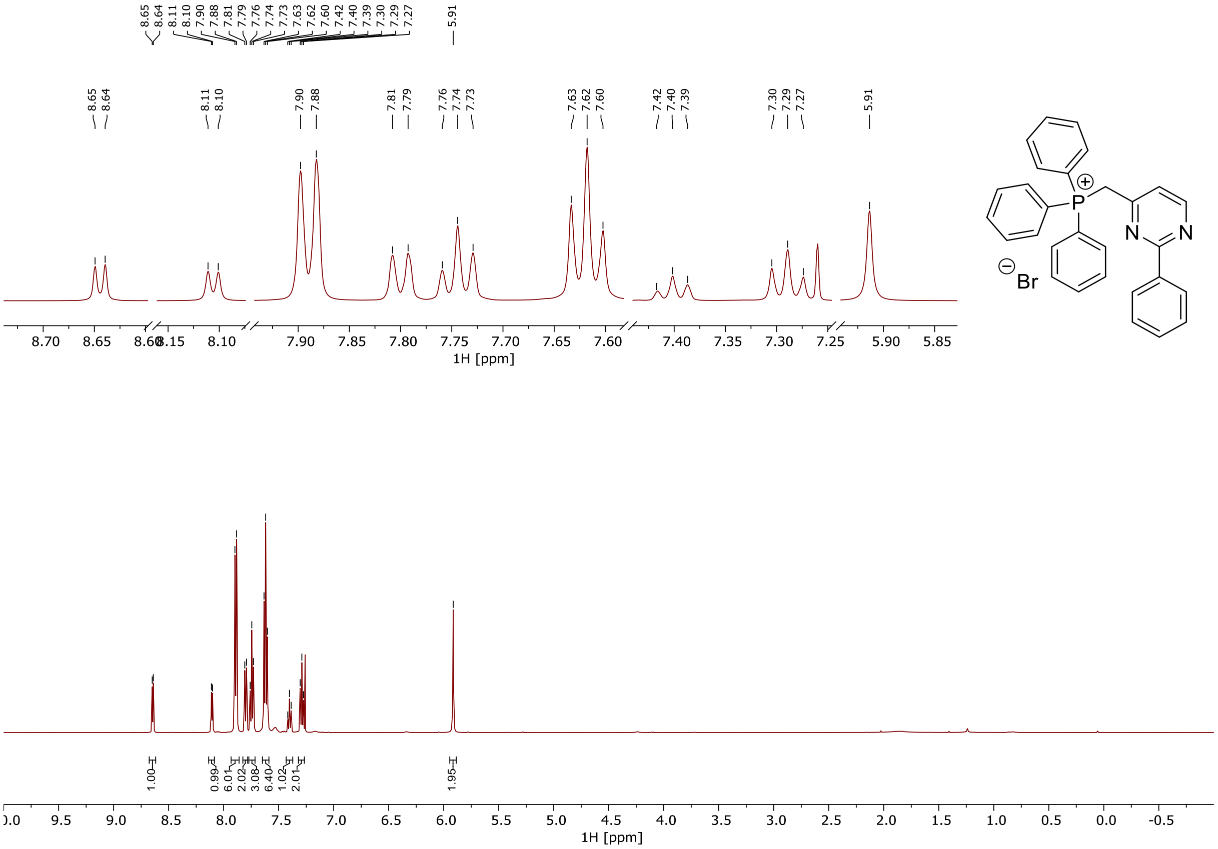

Fig. S95 ^{1}H{^{31}P} NMR (501 MHz, chloroform-*d*, 298 K) spectrum of compound **1o**

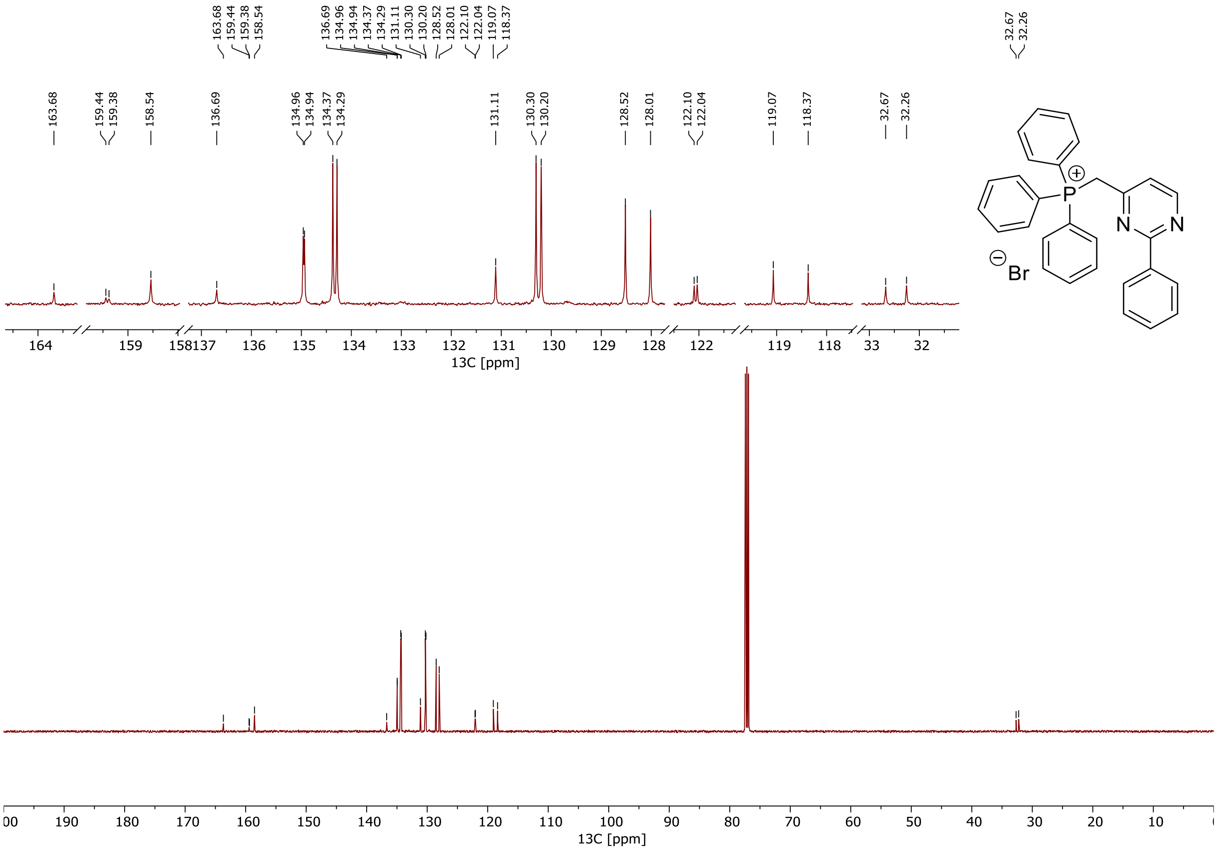

Fig. S96 $^{13}C\{^{1}H\}$ NMR (126 MHz, chloroform-*d*, 298 K) spectrum of compound **1o**

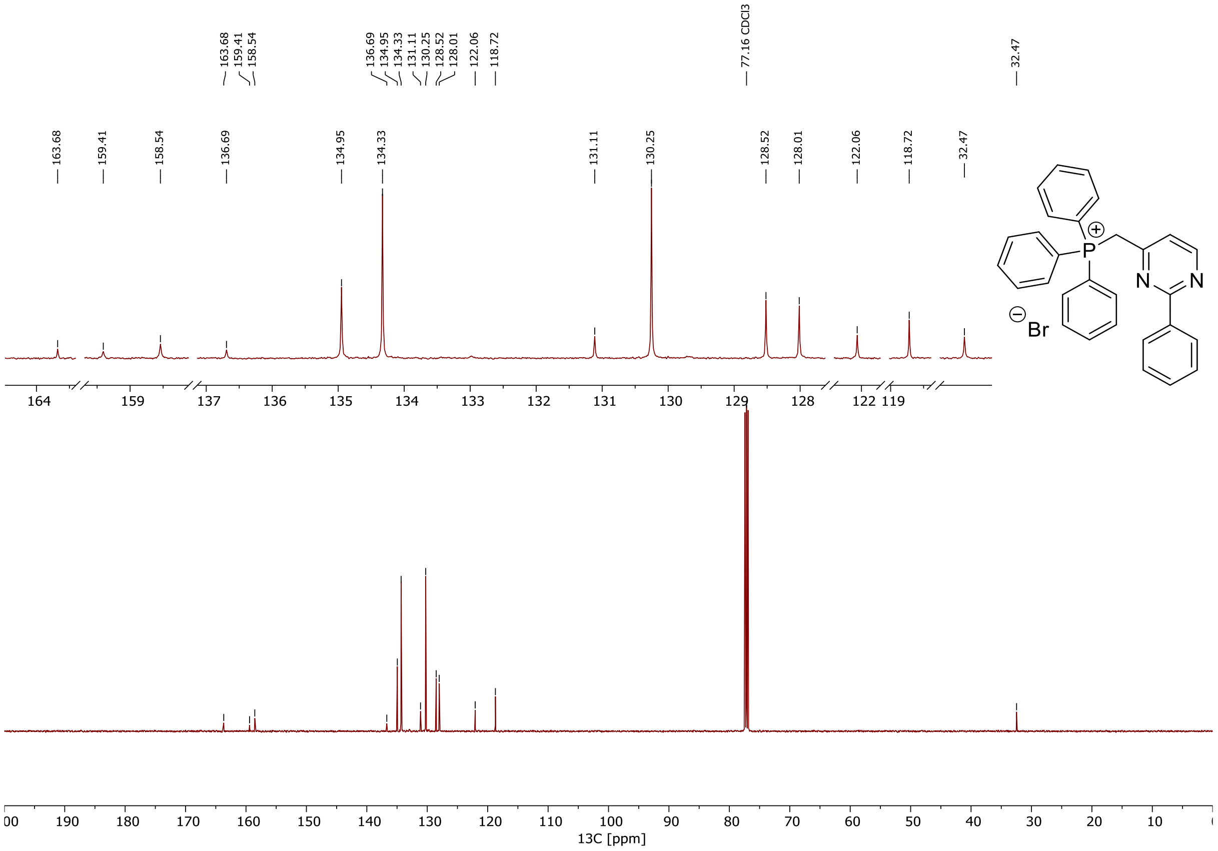

Fig. S97 $^{13}C\{^{1}H,^{31}P\}$ NMR (126 MHz, chloroform-*d*, 298 K) spectrum of compound **1o**

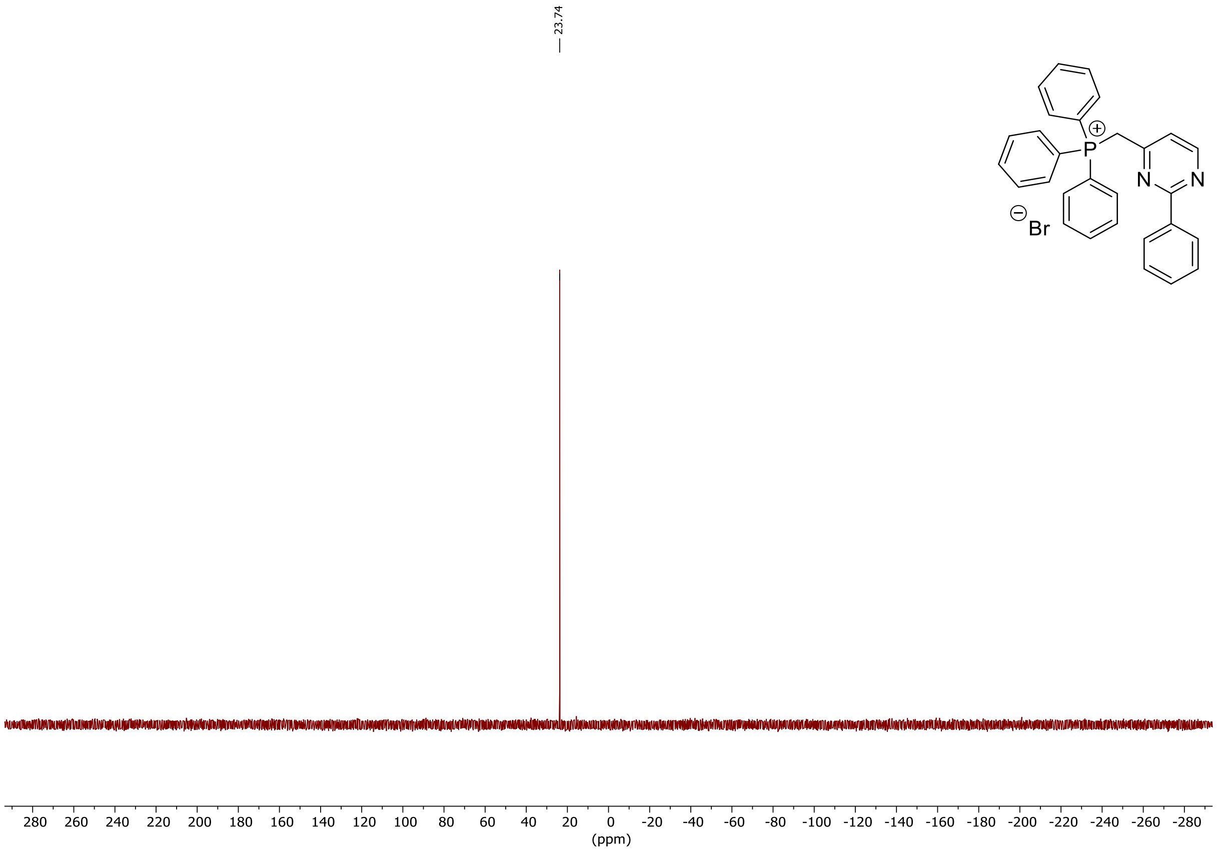

Fig. S98 ^{31}P NMR (203 MHz, chloroform-*d*, 298 K) spectrum of compound **1o**

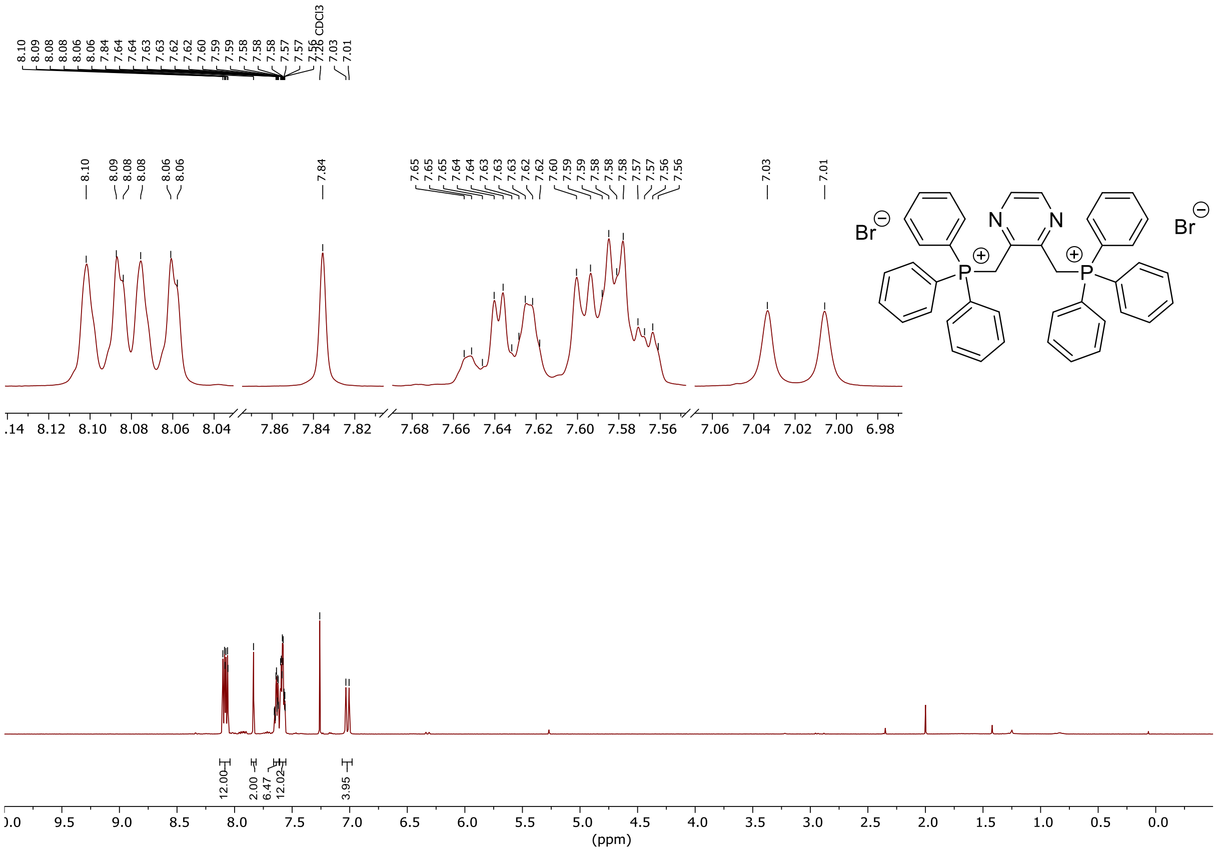

Fig. S99 ^{1}H NMR (501 MHz, chloroform-*d*, 298 K) spectrum of compound **4a**

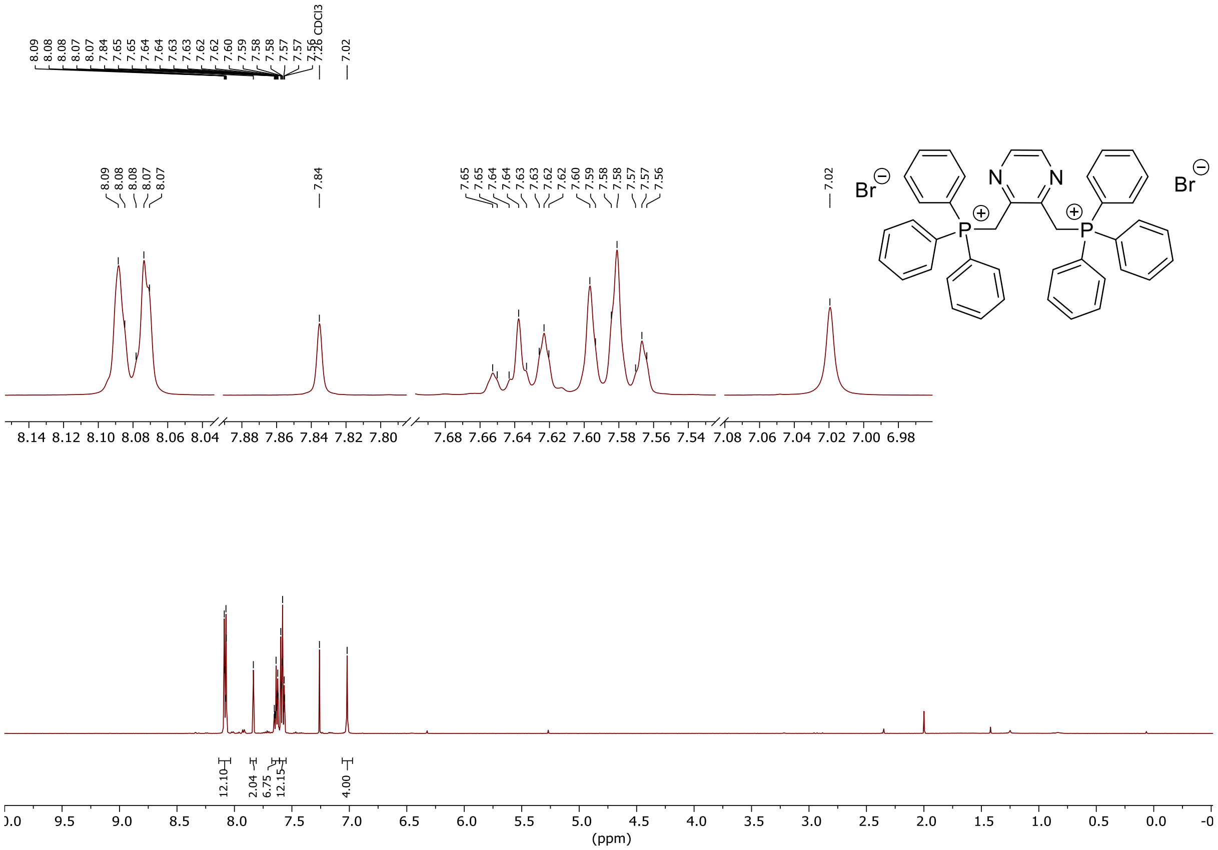

Fig. S100 ^{1}H{^{31}P} NMR (501 MHz, chloroform-*d*, 298 K) spectrum of compound **4a**

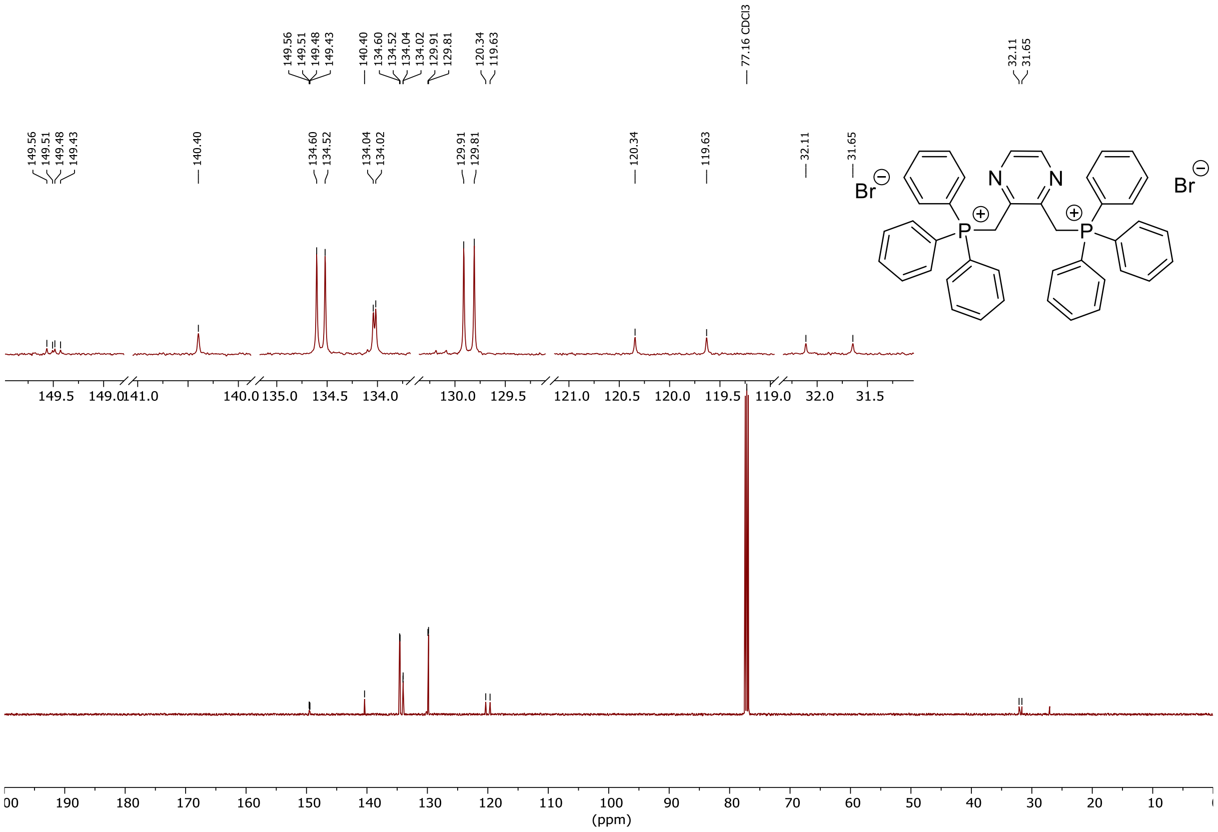

Fig. S101 $^{13}C\{^{1}H\}$ NMR (126 MHz, chloroform-*d*, 298 K) spectrum of compound **4a**

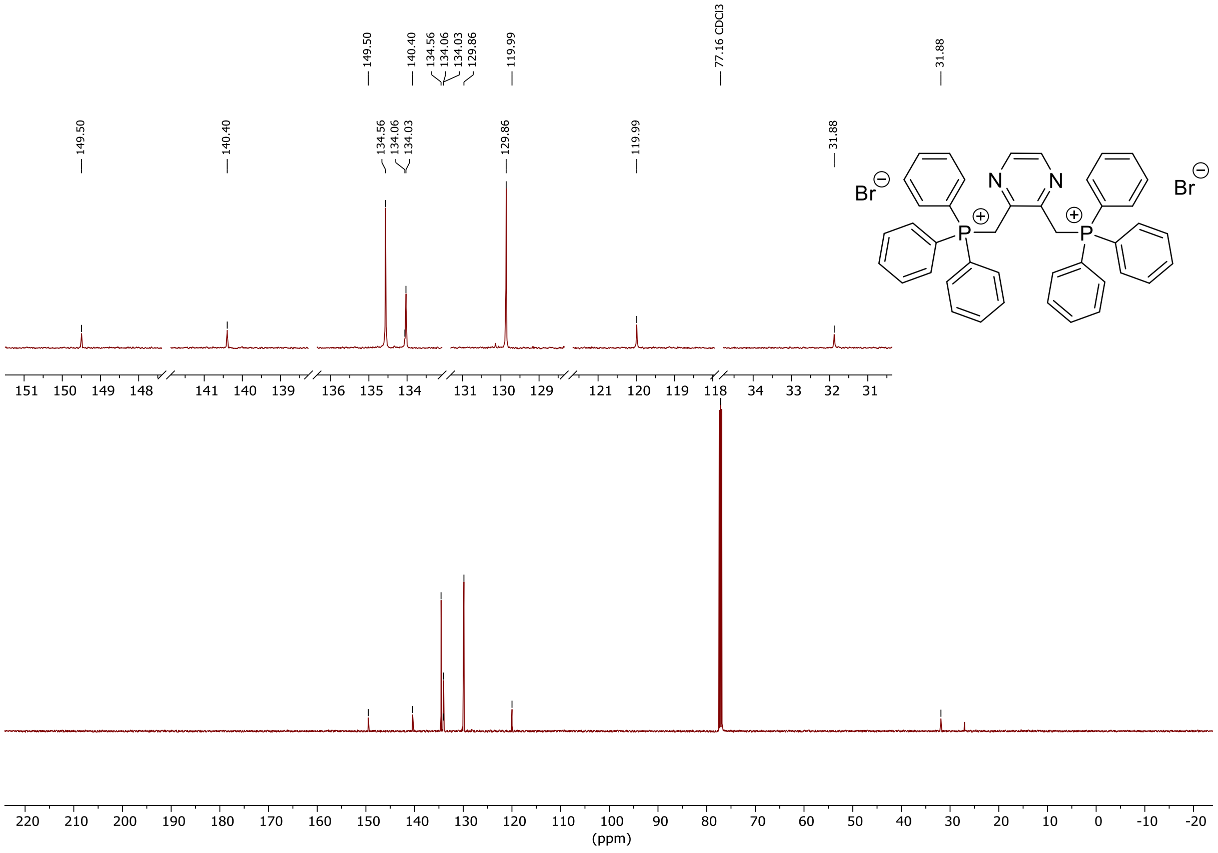

Fig. S102 $^{13}C\{^{1}H,^{31}P\}$ NMR (126 MHz, chloroform-*d*, 298 K) spectrum of compound **4a**

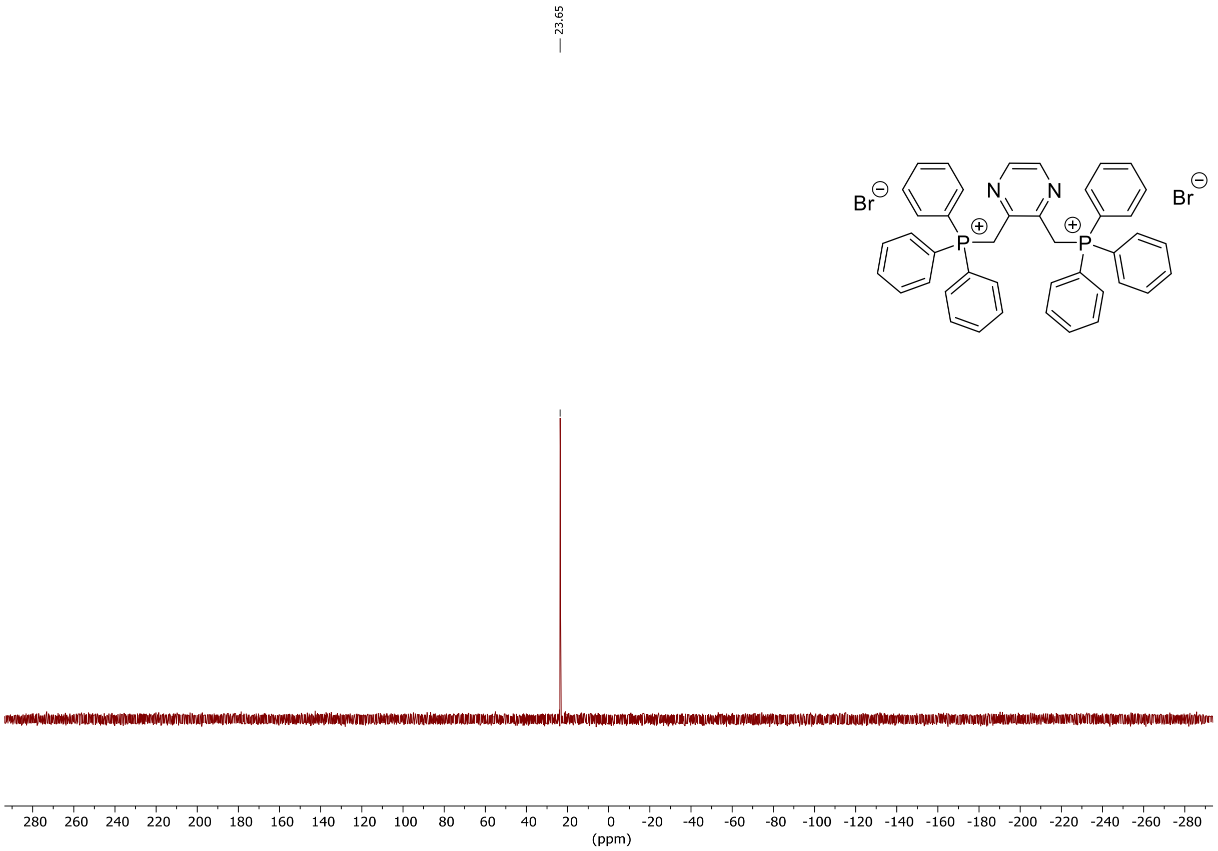

Fig. S103 ^{31}P NMR (203 MHz, chloroform-*d*, 298 K) spectrum of compound **4a**

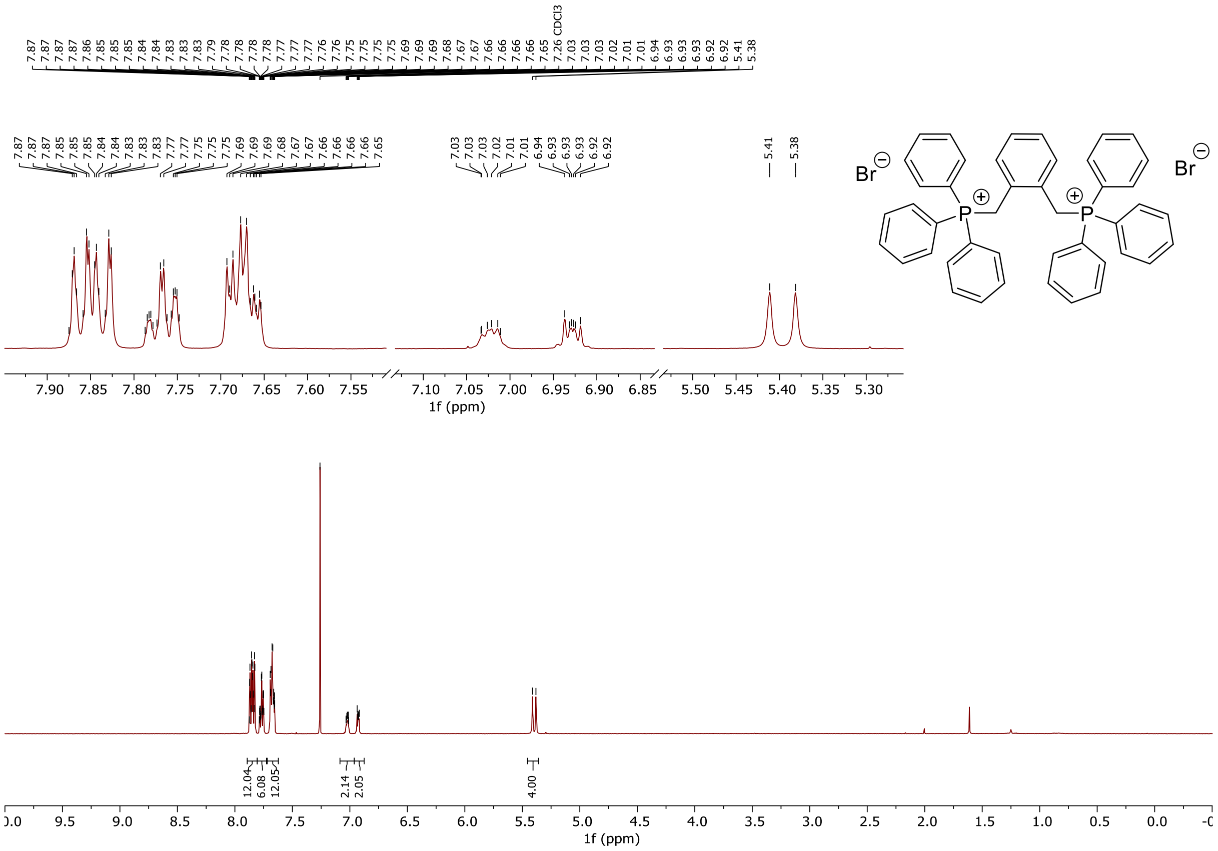

Fig. S104 ^{1}H NMR (501 MHz, chloroform-*d*, 298 K) spectrum of compound **4b**

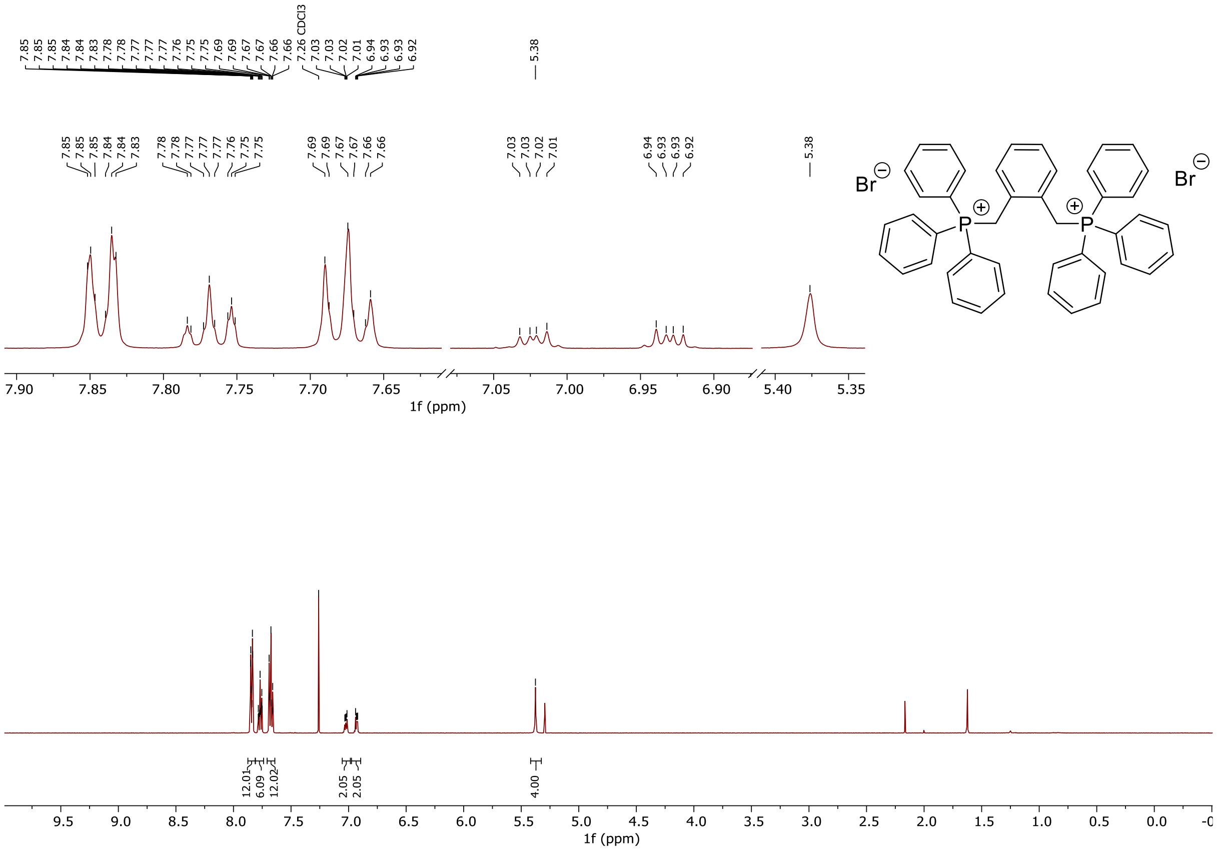

Fig. S105 ^{1}H{^{31}P} NMR (501 MHz, chloroform-*d*, 298 K) spectrum of compound **4b**

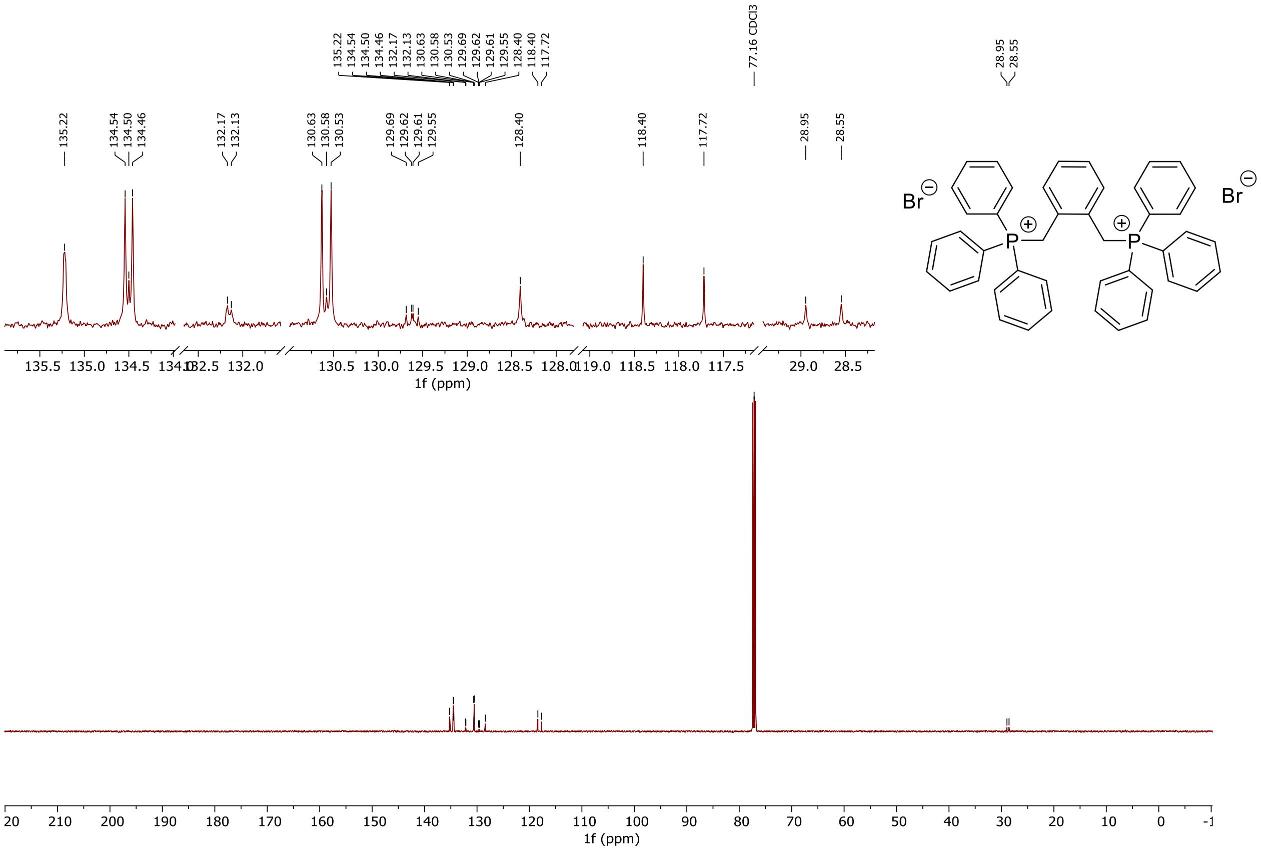

Fig. S106 $^{13}C\{^{1}H\}$ NMR (126 MHz, chloroform-*d*, 298 K) spectrum of compound **4b**

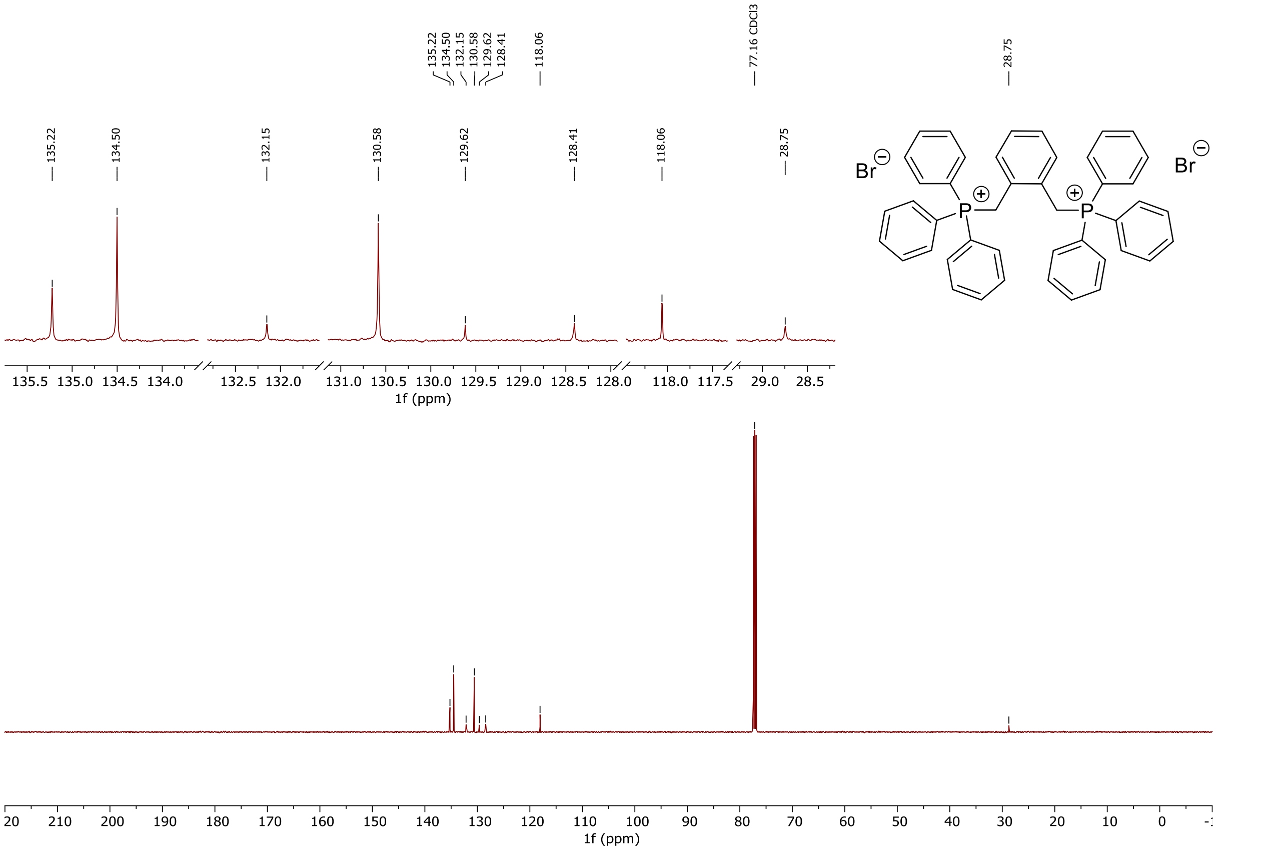

Fig. S107 $^{13}C\{^{1}H,^{31}P\}$ NMR (126 MHz, chloroform-*d*, 298 K) spectrum of compound **4b**

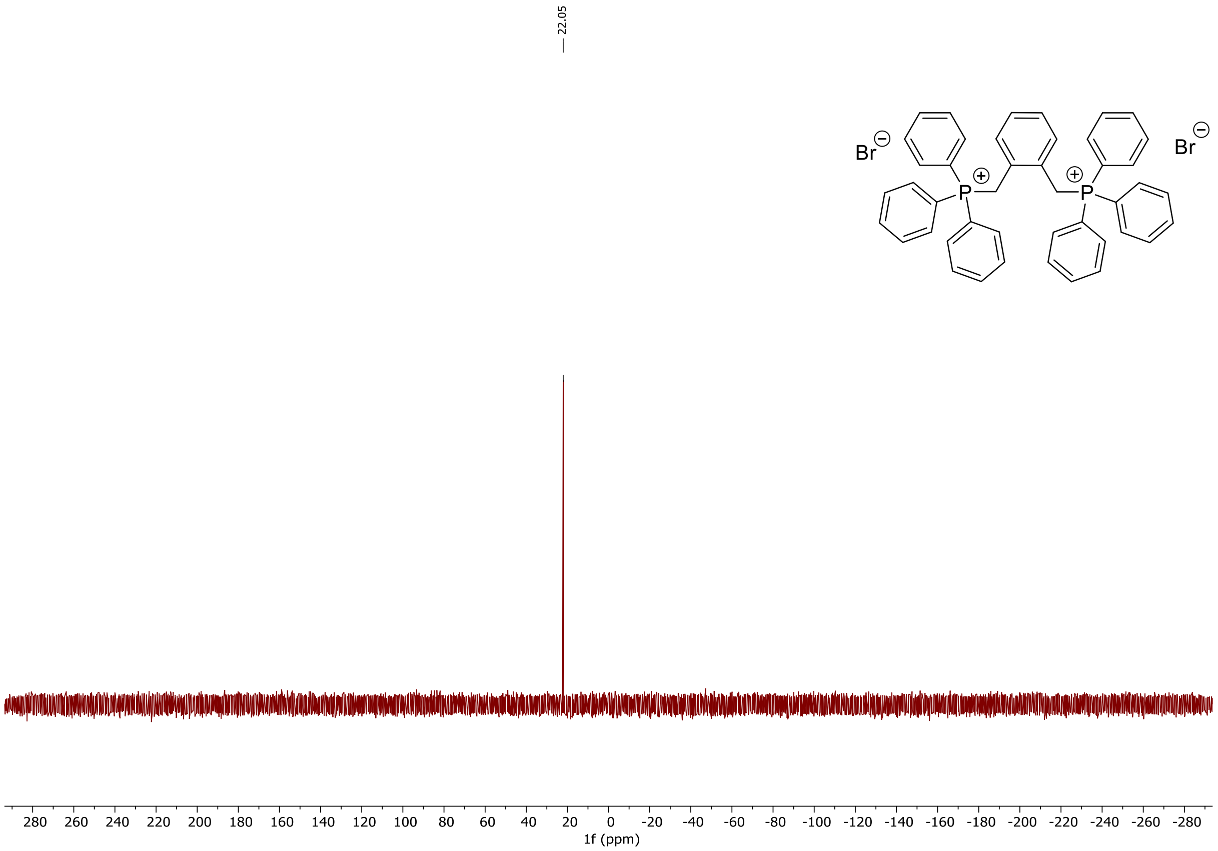

Fig. S108 ^{31}P NMR (203 MHz, chloroform-*d*, 298 K) spectrum of compound **4b**

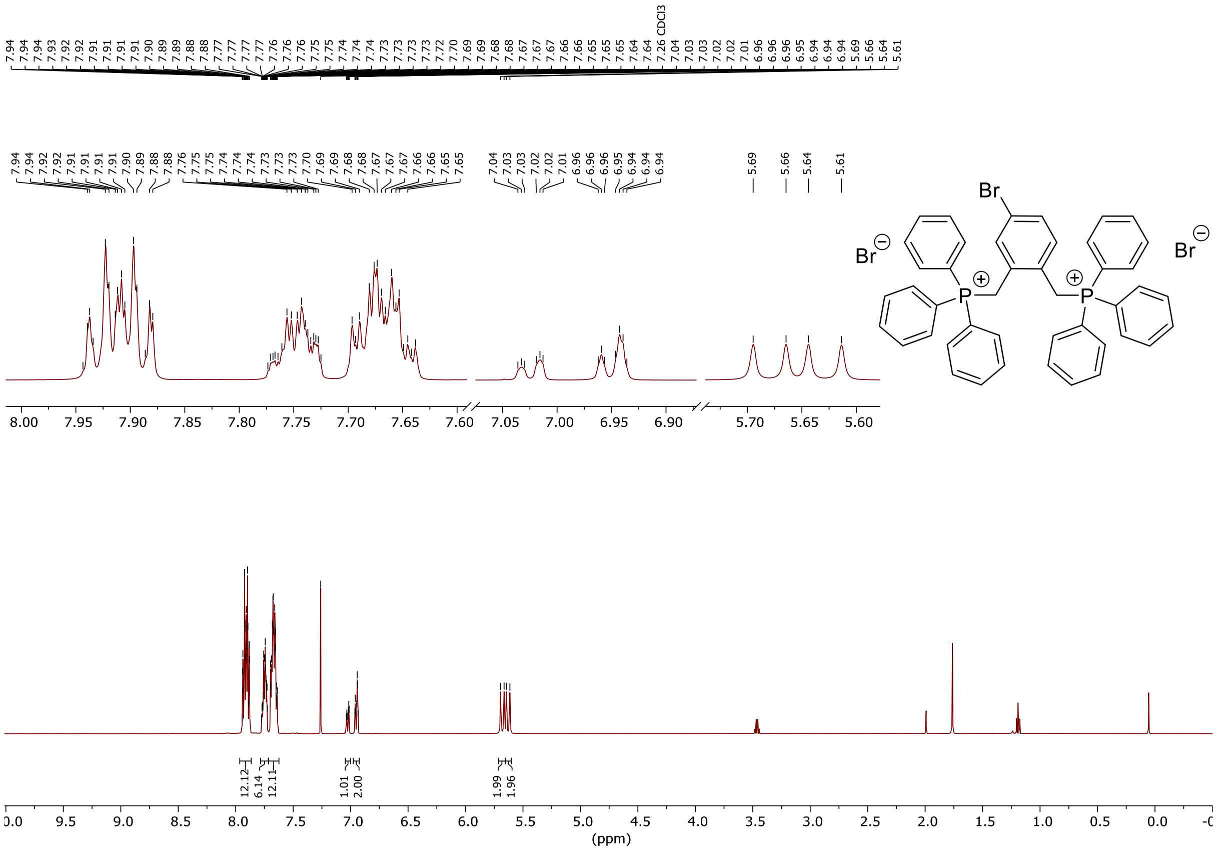

Fig. S109 ^{1}H NMR (501 MHz, chloroform-*d*, 298 K) spectrum of compound **4c**

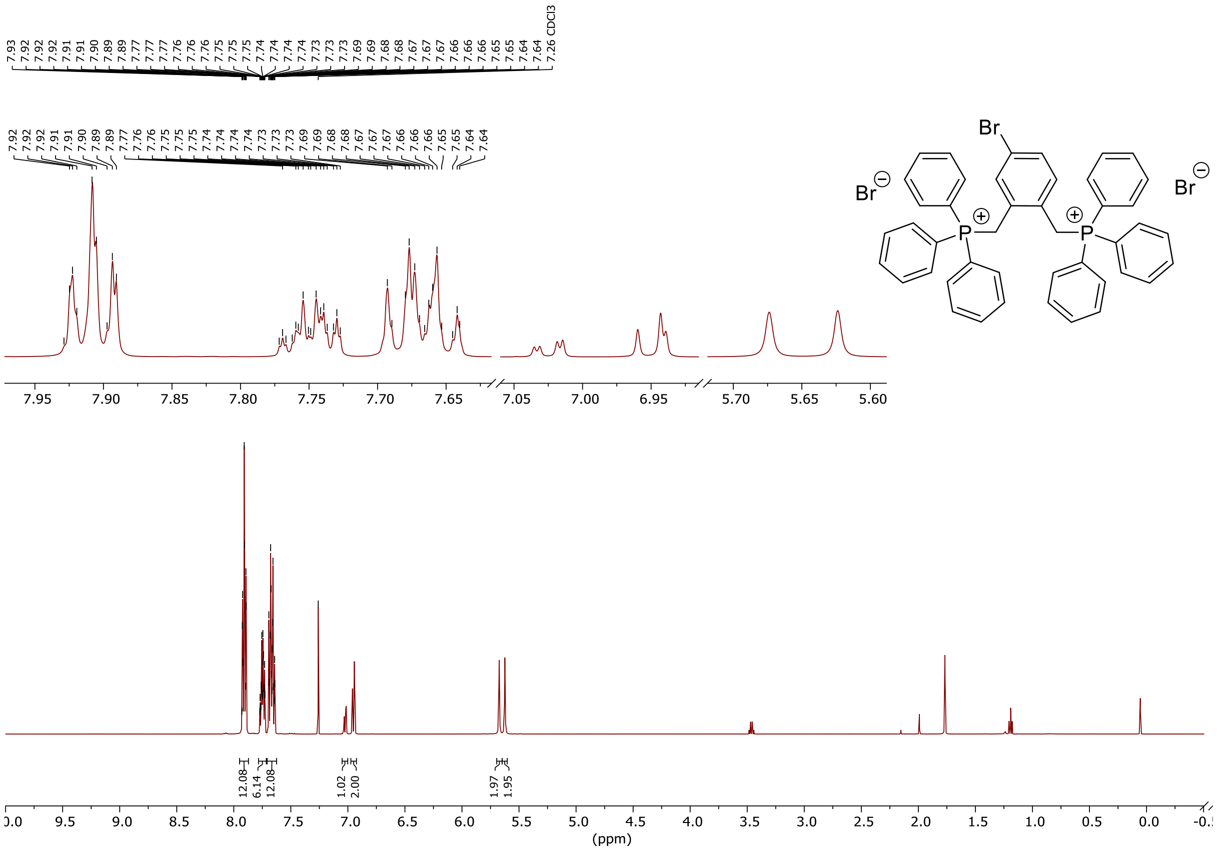

Fig. S110 ^{1}H{^{31}P} NMR (501 MHz, chloroform-*d*, 298 K) spectrum of compound **4c**

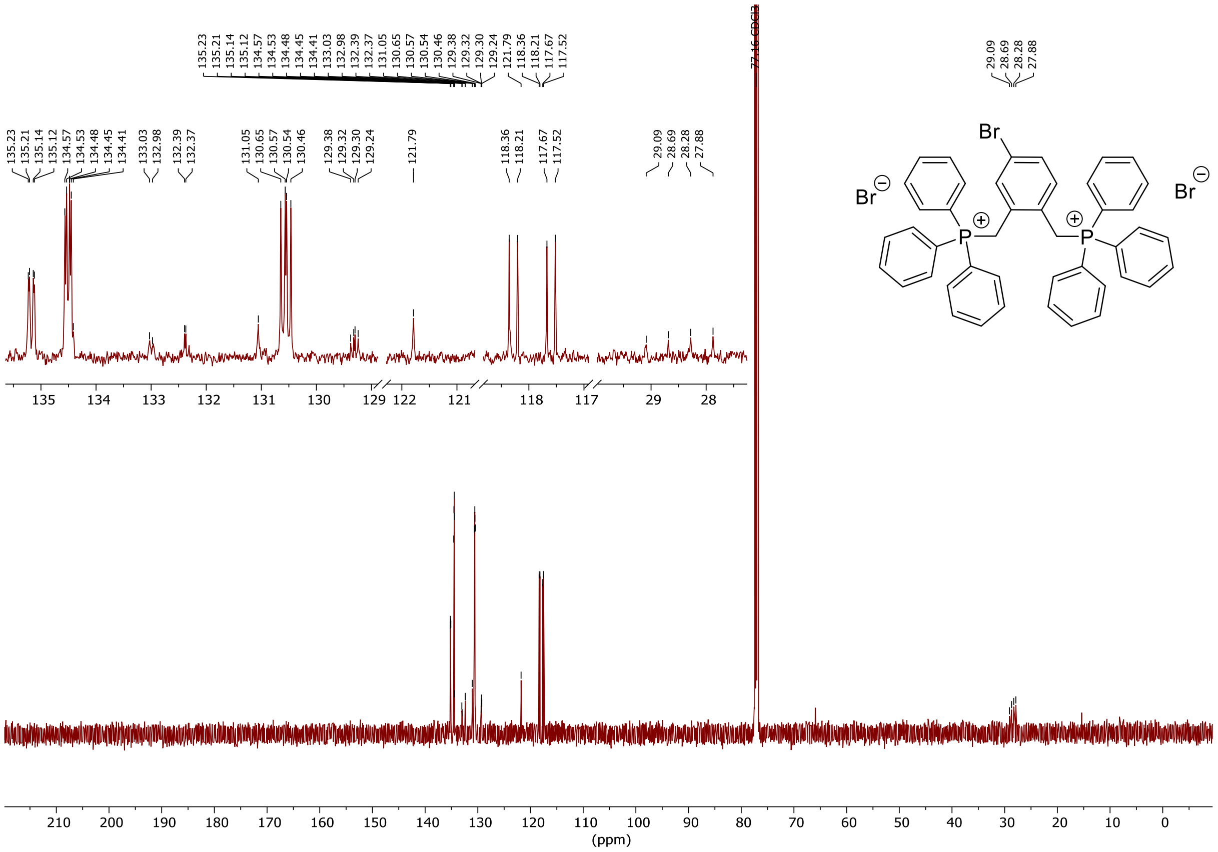

Fig. S111 $^{13}C\{1H\}$ NMR (126 MHz, chloroform-*d*, 298 K) spectrum of compound **4c**

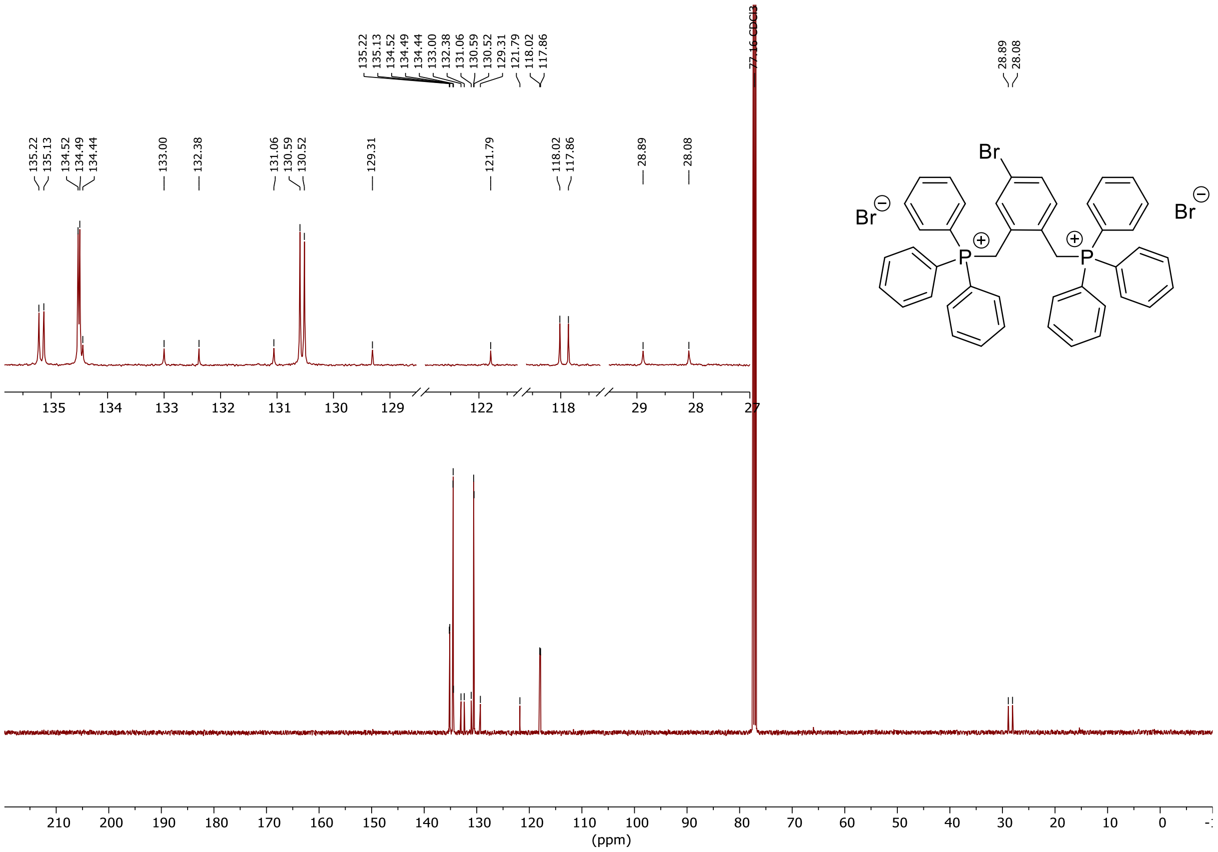

Fig. S112 $^{13}C\{^1H,^{31}P\}$ NMR (126 MHz, chloroform-*d*, 298 K) spectrum of compound **4c**

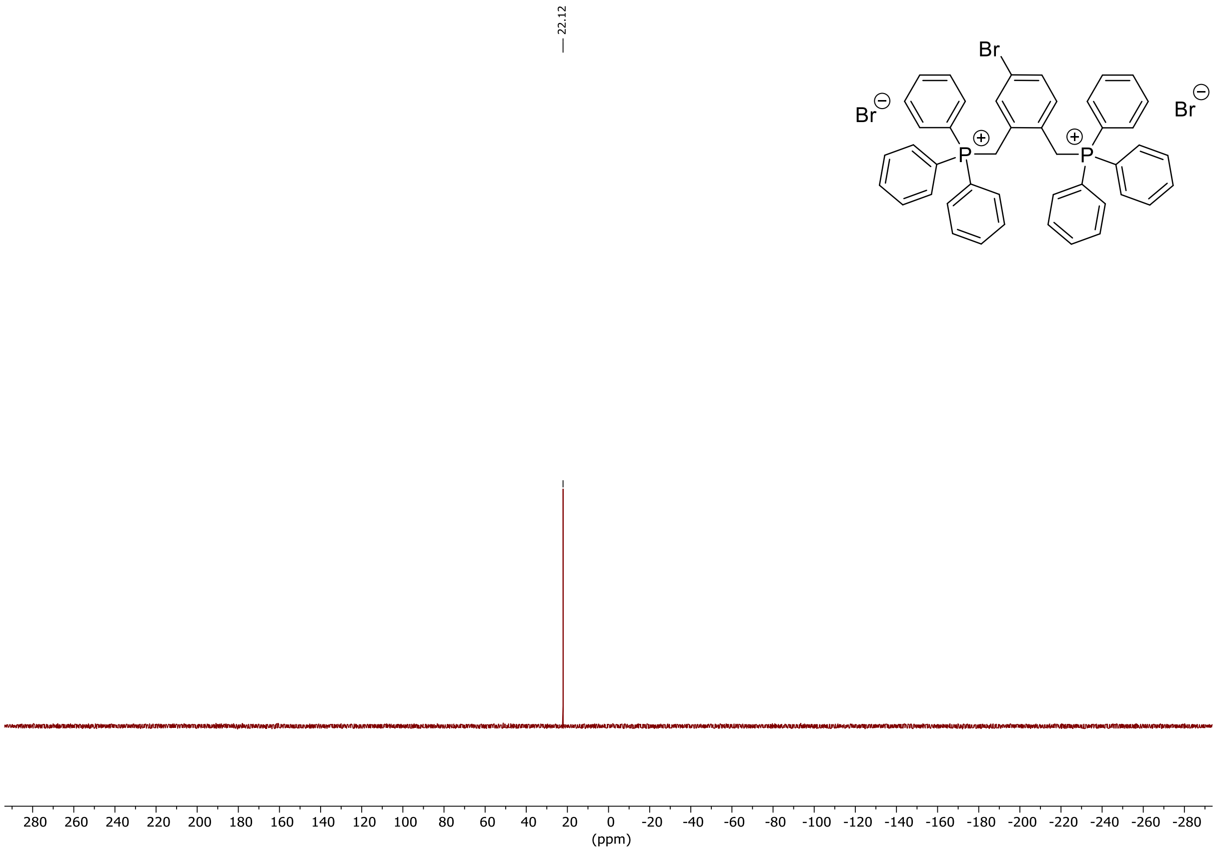

Fig. S113 ^{31}P NMR (203 MHz, chloroform-*d*, 298 K) spectrum of compound **4c**

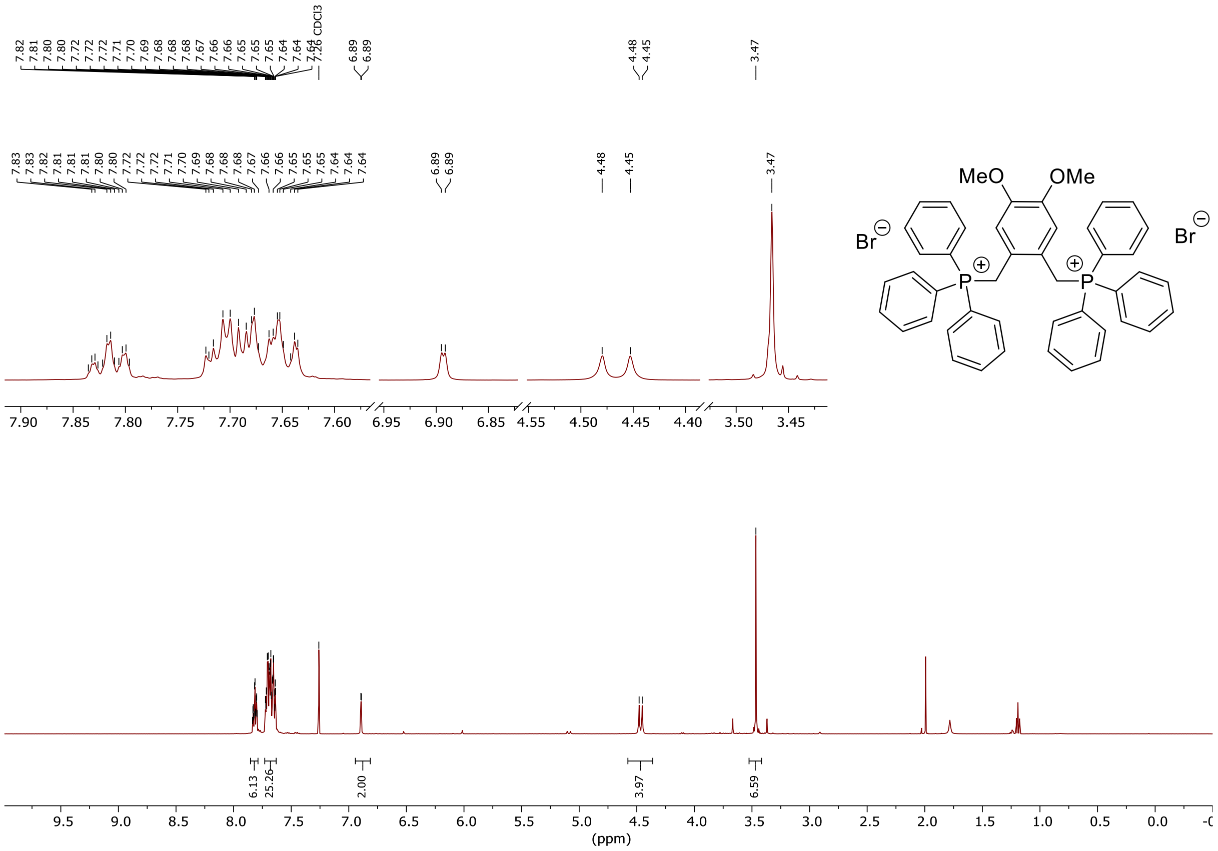

Fig. S114 ^{1}H NMR (501 MHz, chloroform-*d*, 298 K) spectrum of compound **4d**

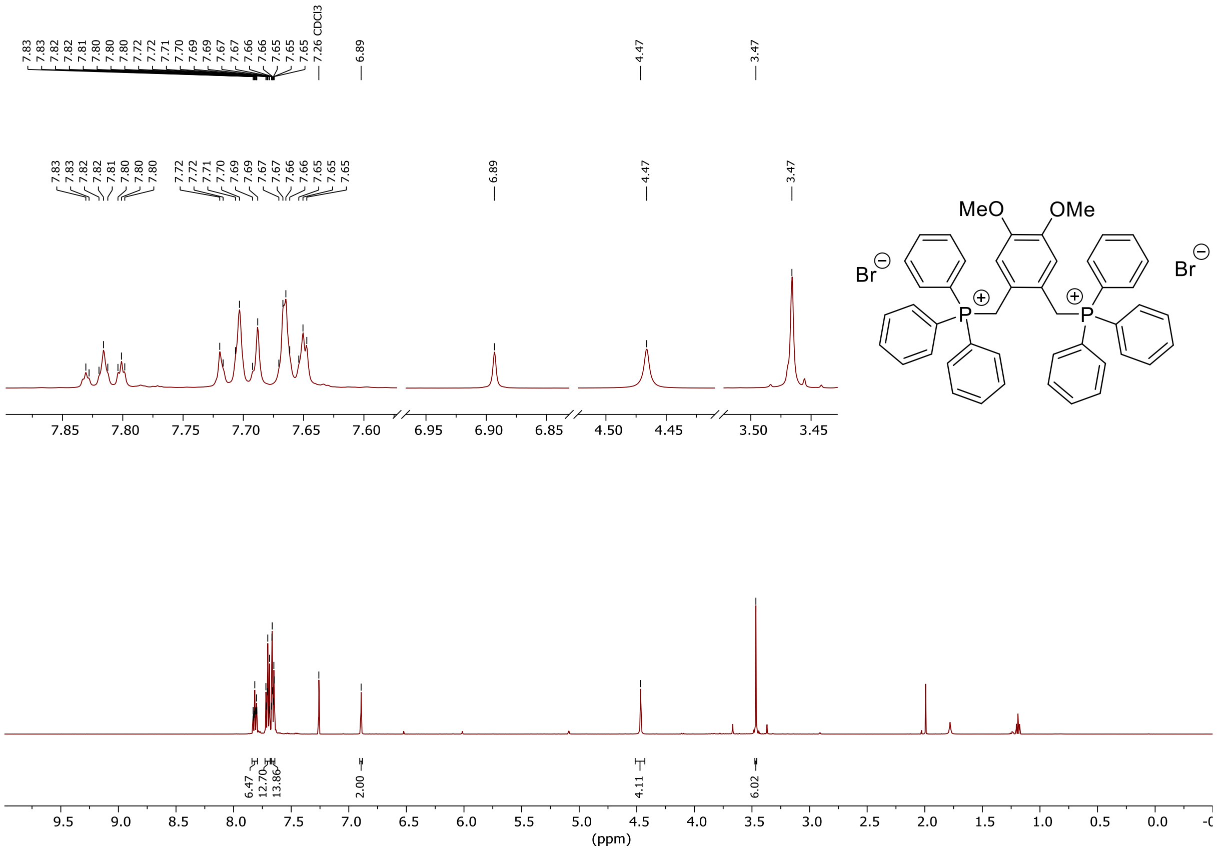

Fig. S115 $^{1}H\{^{31}P\}$ NMR (501 MHz, chloroform-*d*, 298 K) spectrum of compound **4d**

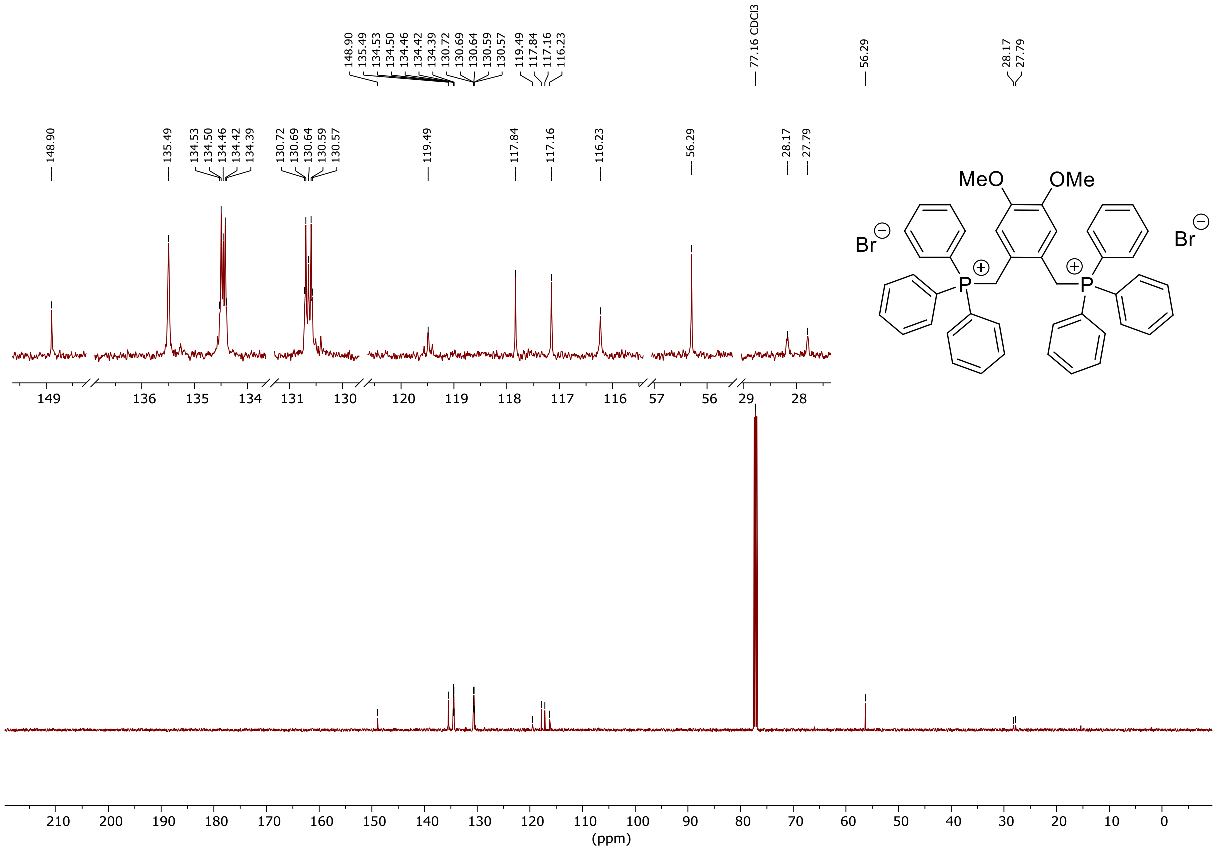

Fig. S116 $^{13}C\{^{1}H\}$ NMR (126 MHz, chloroform-*d*, 298 K) spectrum of compound **4d**

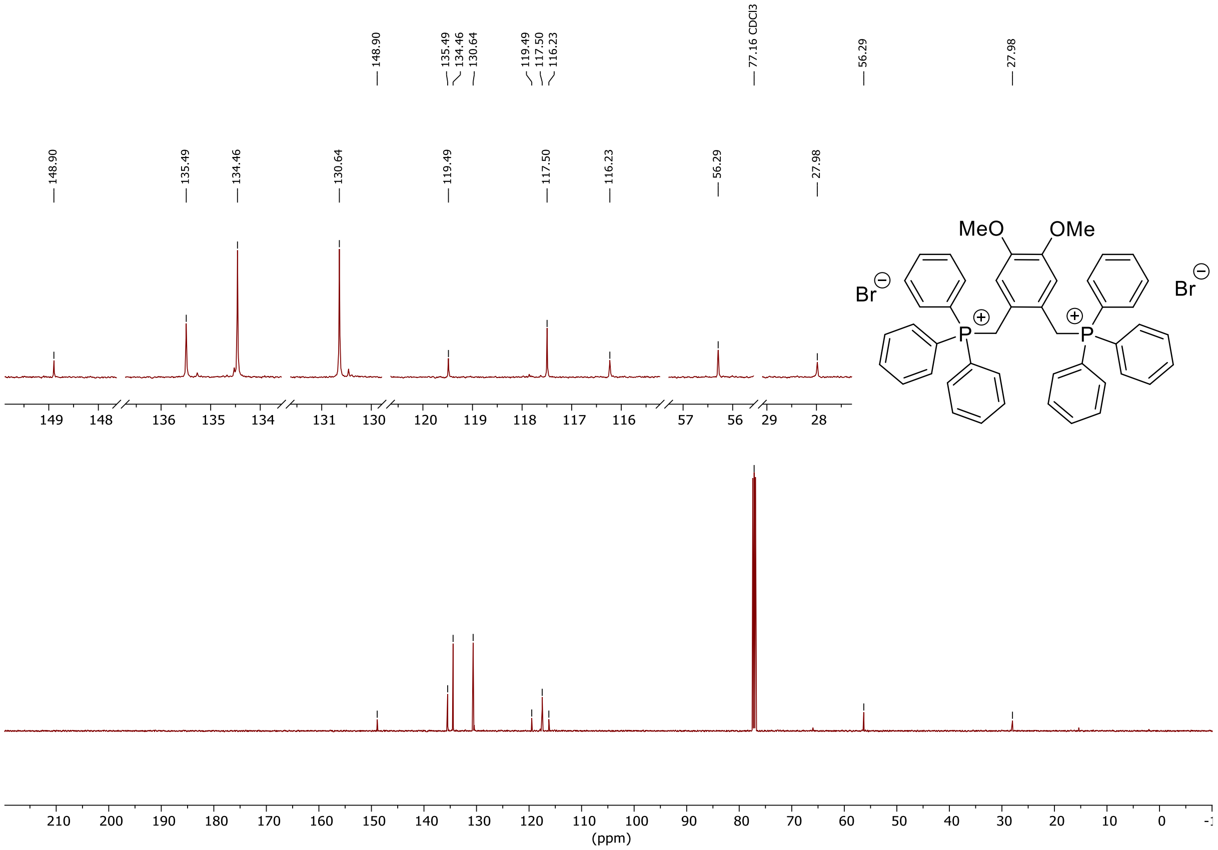

Fig. S117 $^{13}C\{^{1}H,^{31}P\}$ NMR (126 MHz, chloroform-*d*, 298 K) spectrum of compound **4d**

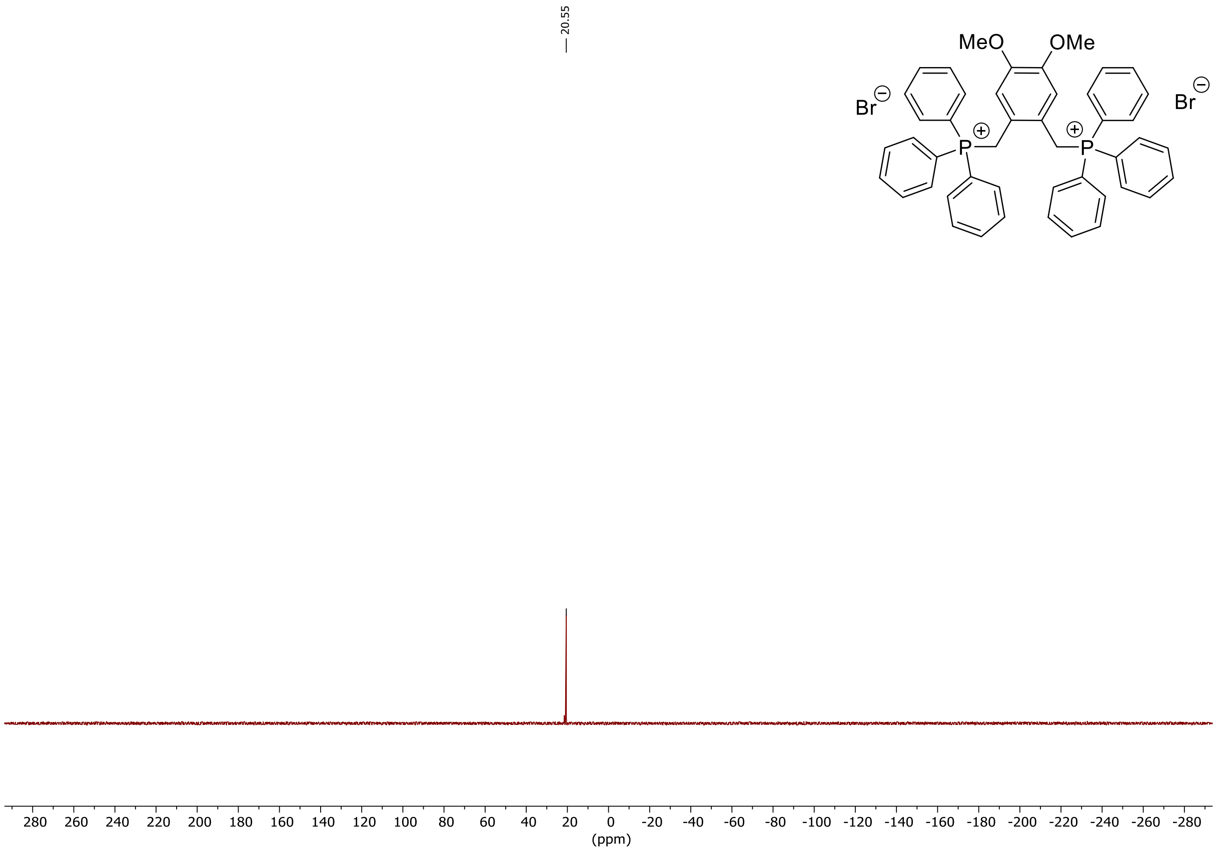

Fig. S118 ^{31}P NMR (203 MHz, chloroform-*d*, 298 K) spectrum of compound **4d**

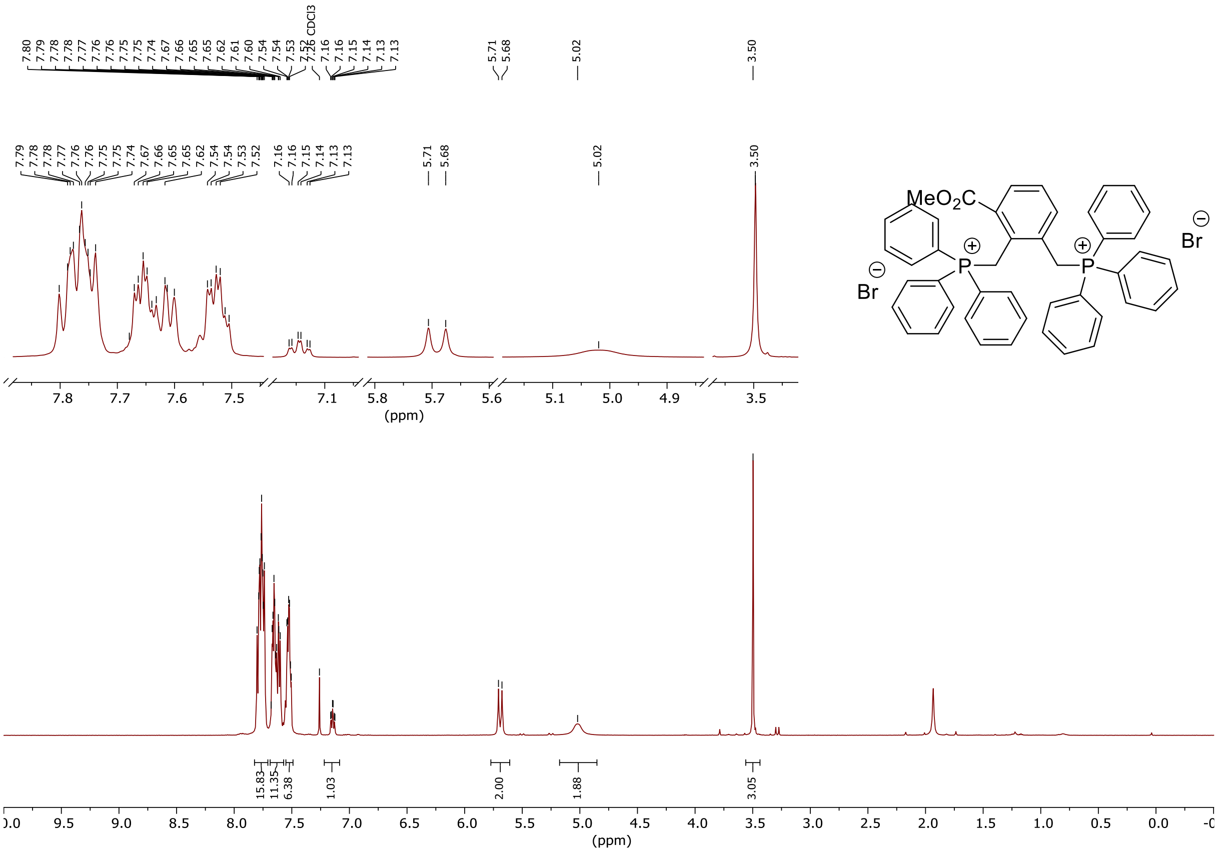

Fig. S119 ^{1}H NMR (501 MHz, chloroform-*d*, 298 K) spectrum of compound **4e**

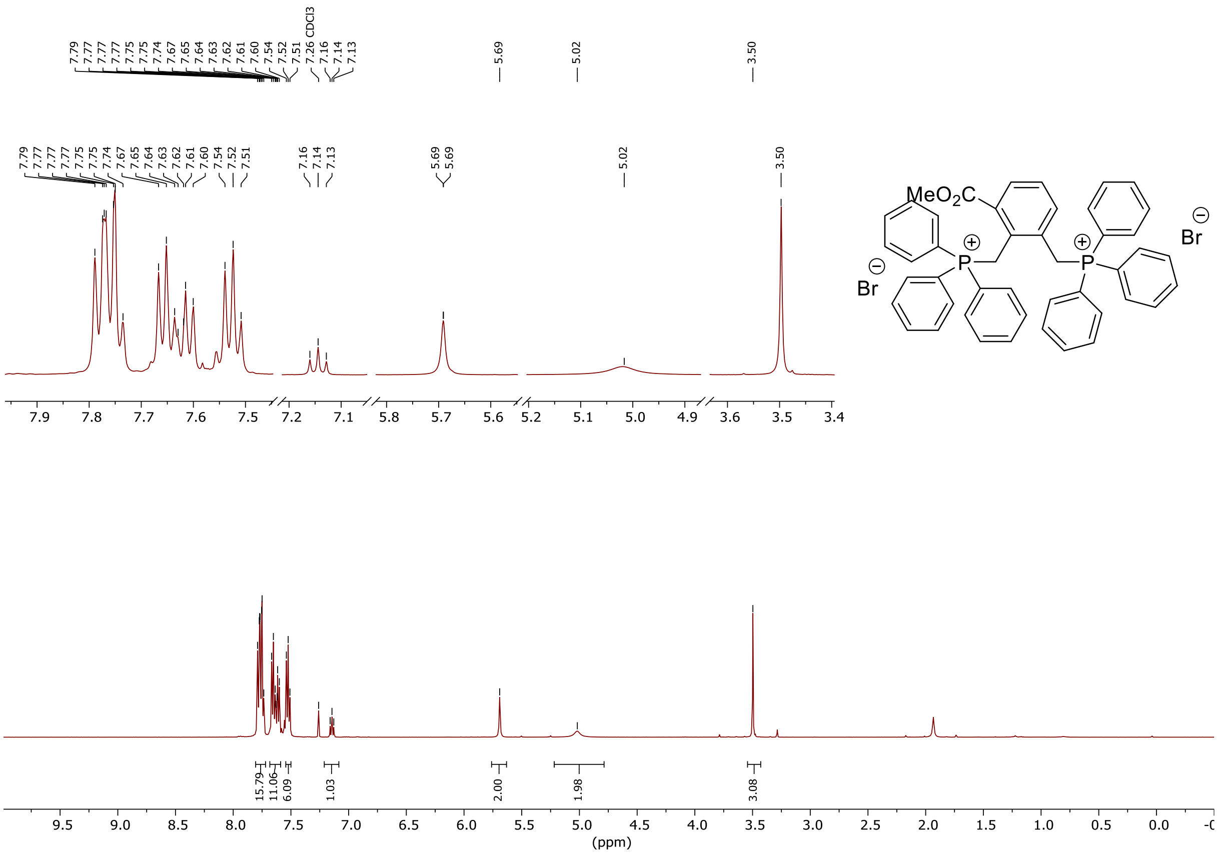

Fig. S120 ^{1}H{^{31}P} NMR (501 MHz, chloroform-*d*, 298 K) spectrum of compound **4e**

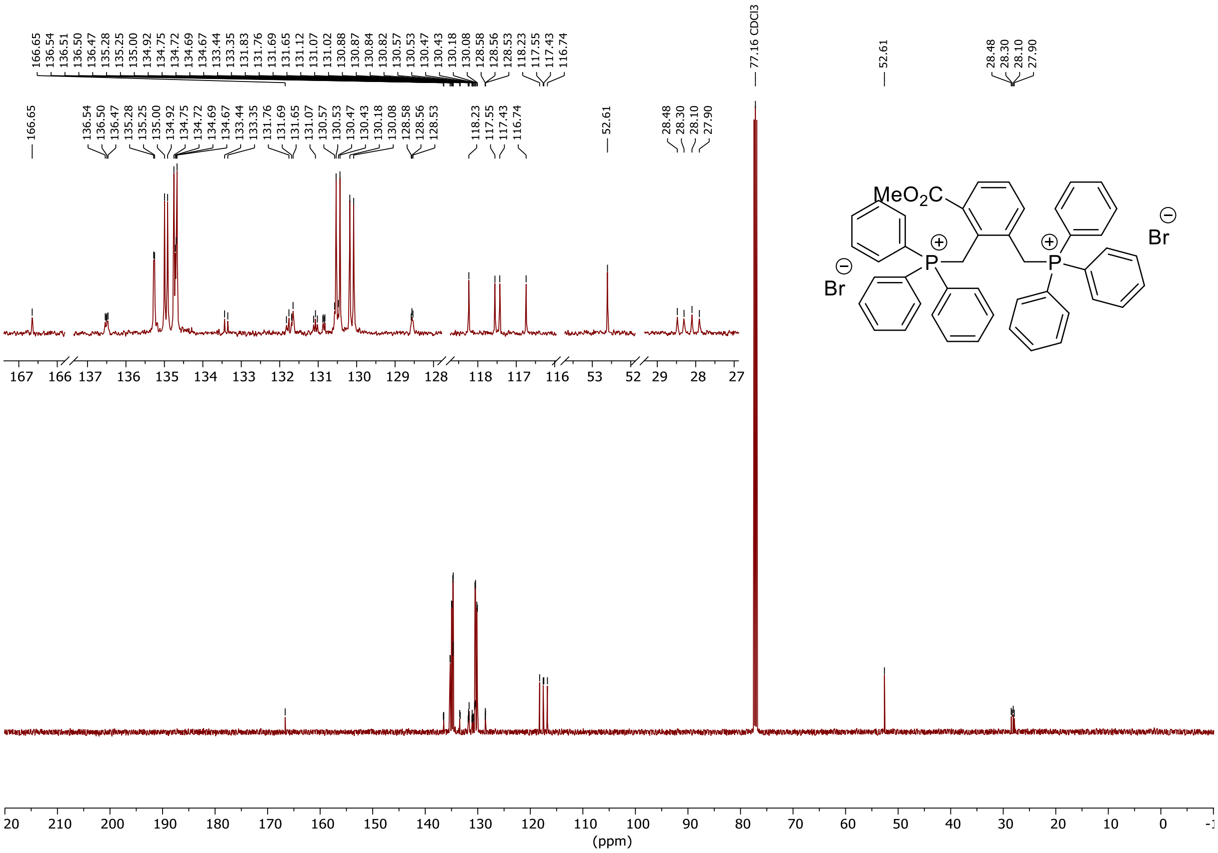

Fig. S121 $^{13}C\{^1H\}$ NMR (126 MHz, chloroform-*d*, 298 K) spectrum of compound **4e**

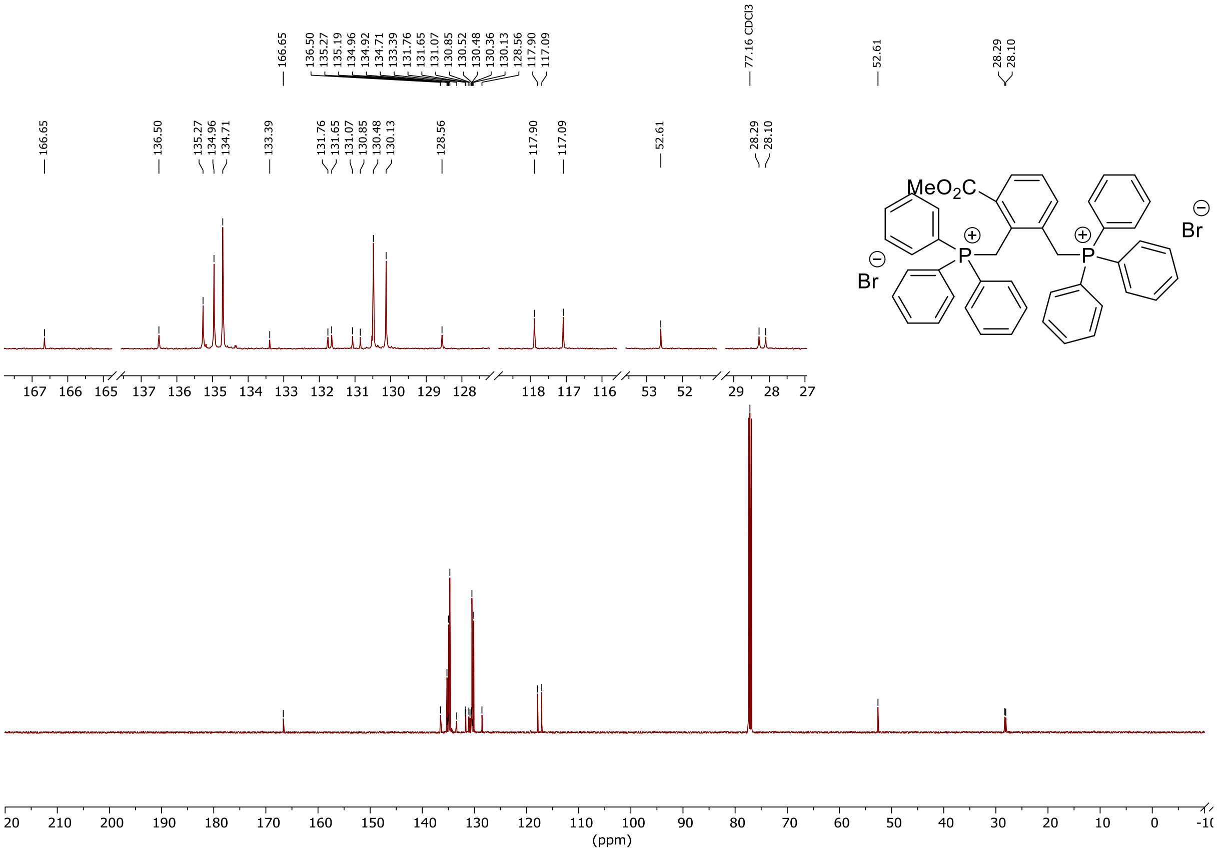

Fig. S122 $^{13}C\{^1H,^{31}P\}$ NMR (126 MHz, chloroform-*d*, 298 K) spectrum of compound **4e**

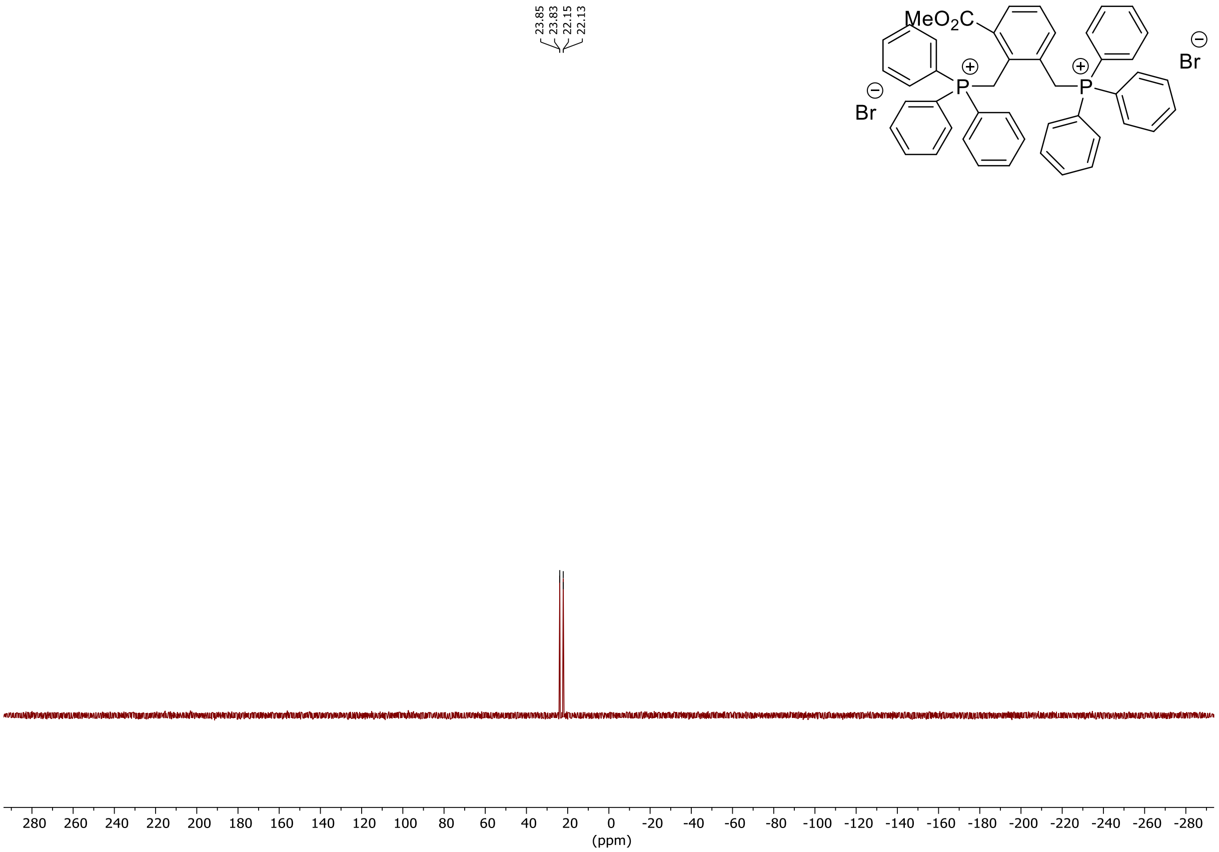

Fig. S123 ^{31}P NMR (203 MHz, chloroform-*d*, 298 K) spectrum of compound **4e**

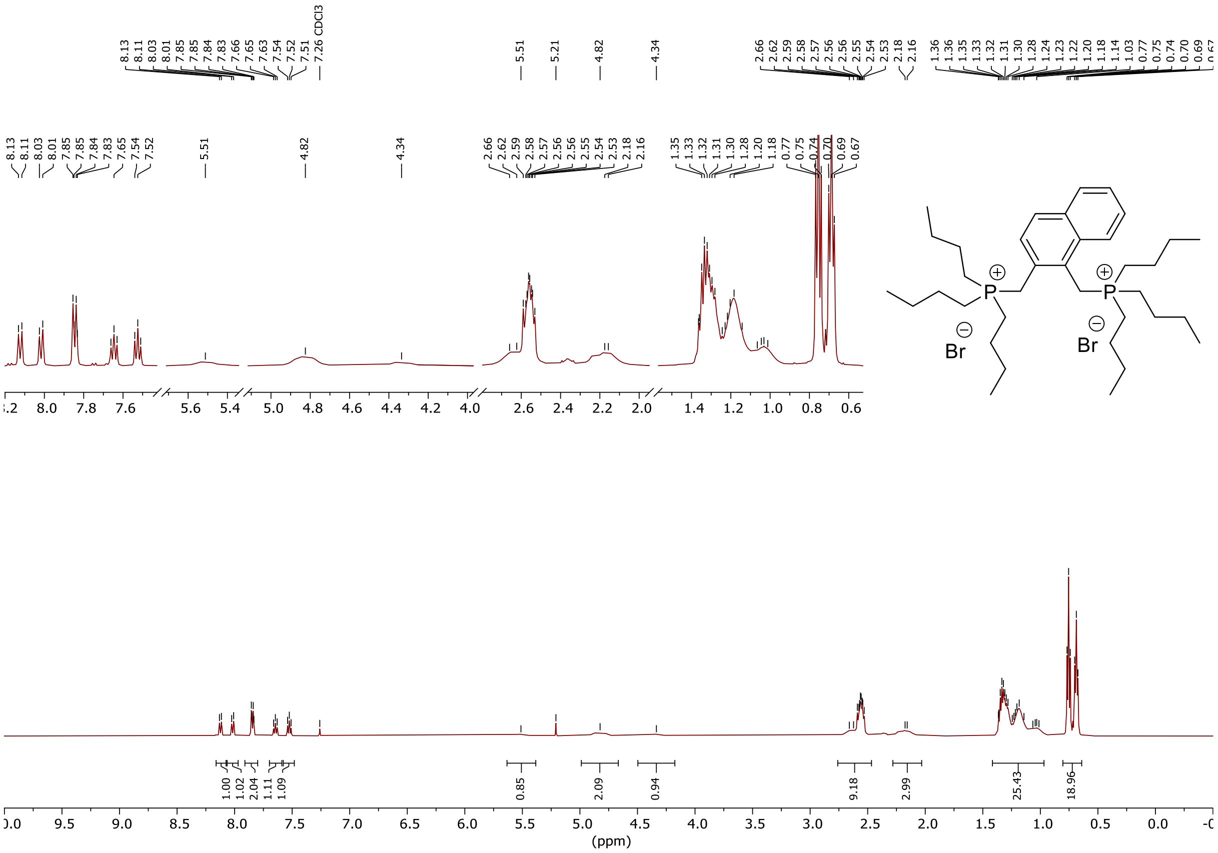

Fig. S124 ^{1}H NMR (600 MHz, chloroform-*d*, 298 K) spectrum of compound **4f**

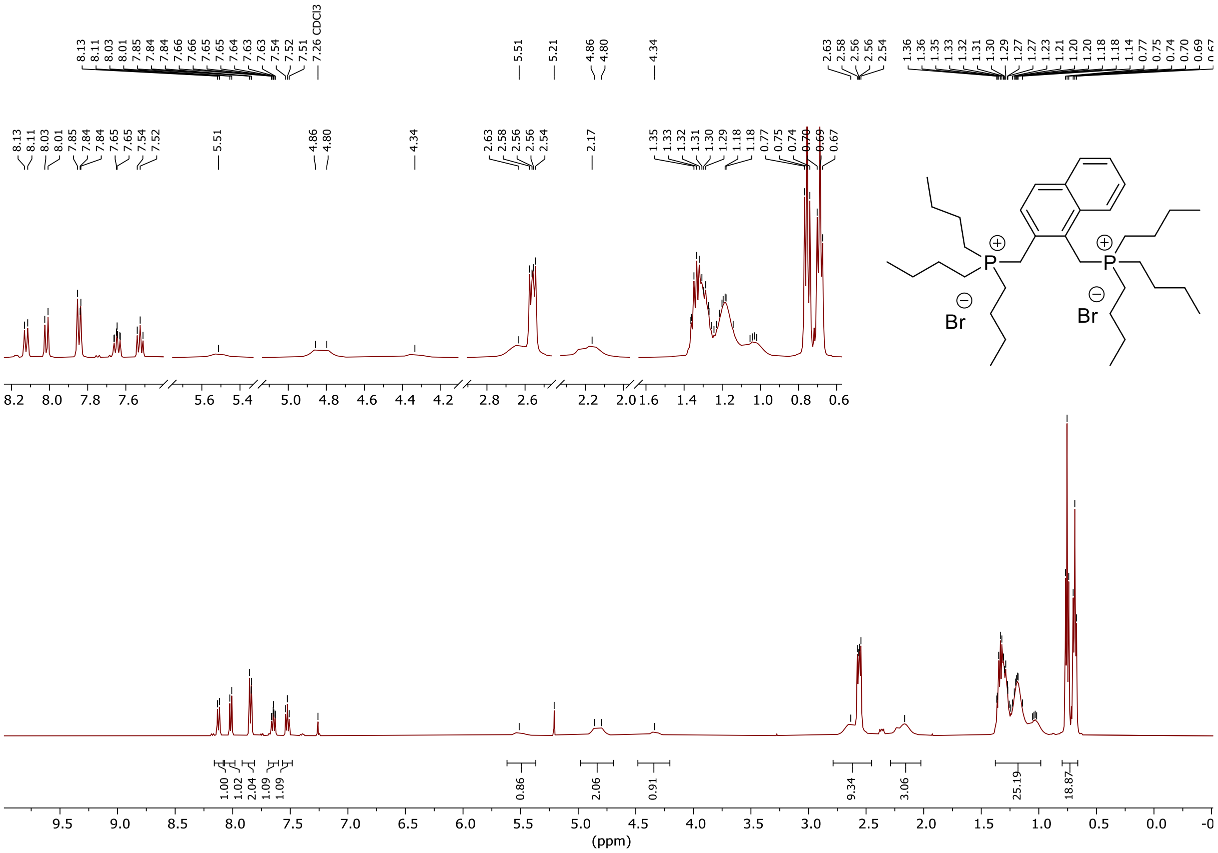

Fig. S125 ^{1}H{^{31}P} NMR (600 MHz, chloroform-*d*, 298 K) spectrum of compound **4f**

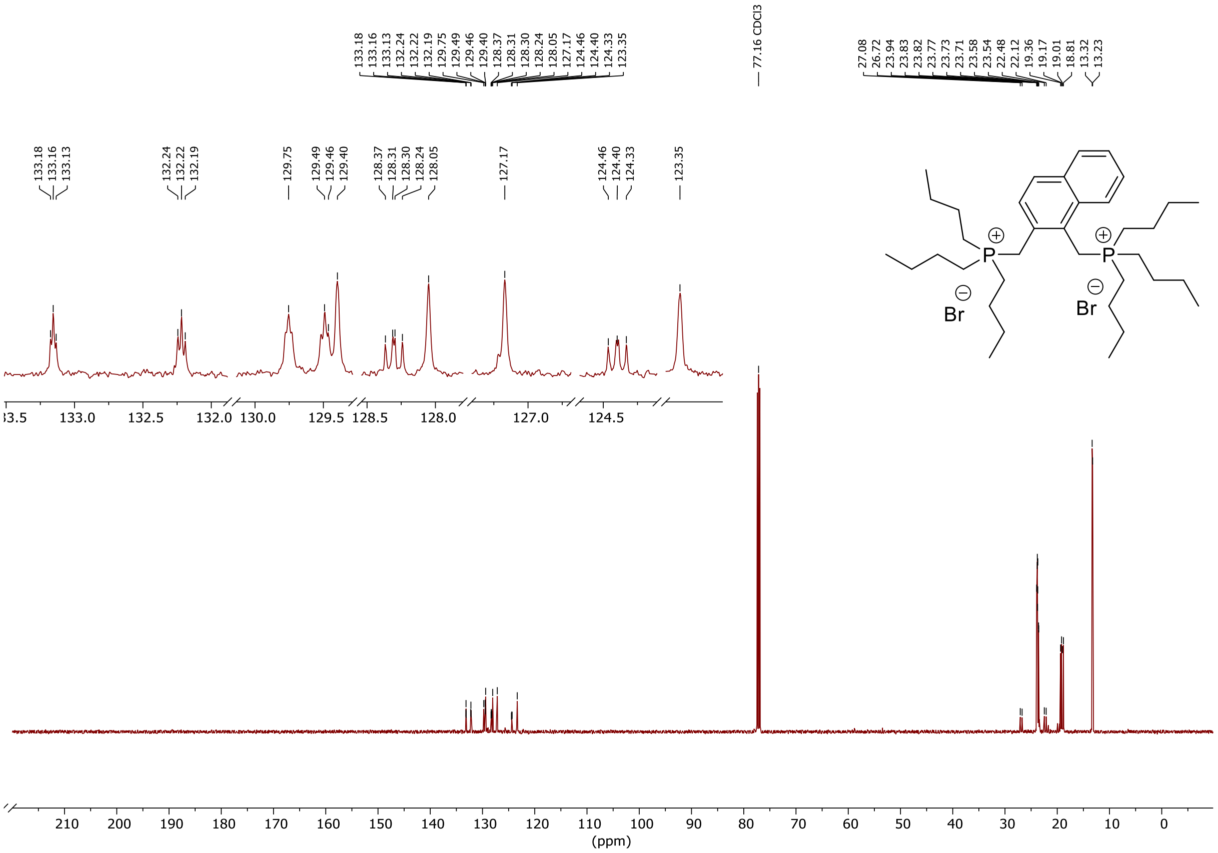

Fig. S126 $^{13}C\{^{1}H\}$ NMR (151 MHz, chloroform-*d*, 298 K) spectrum of compound **4f**

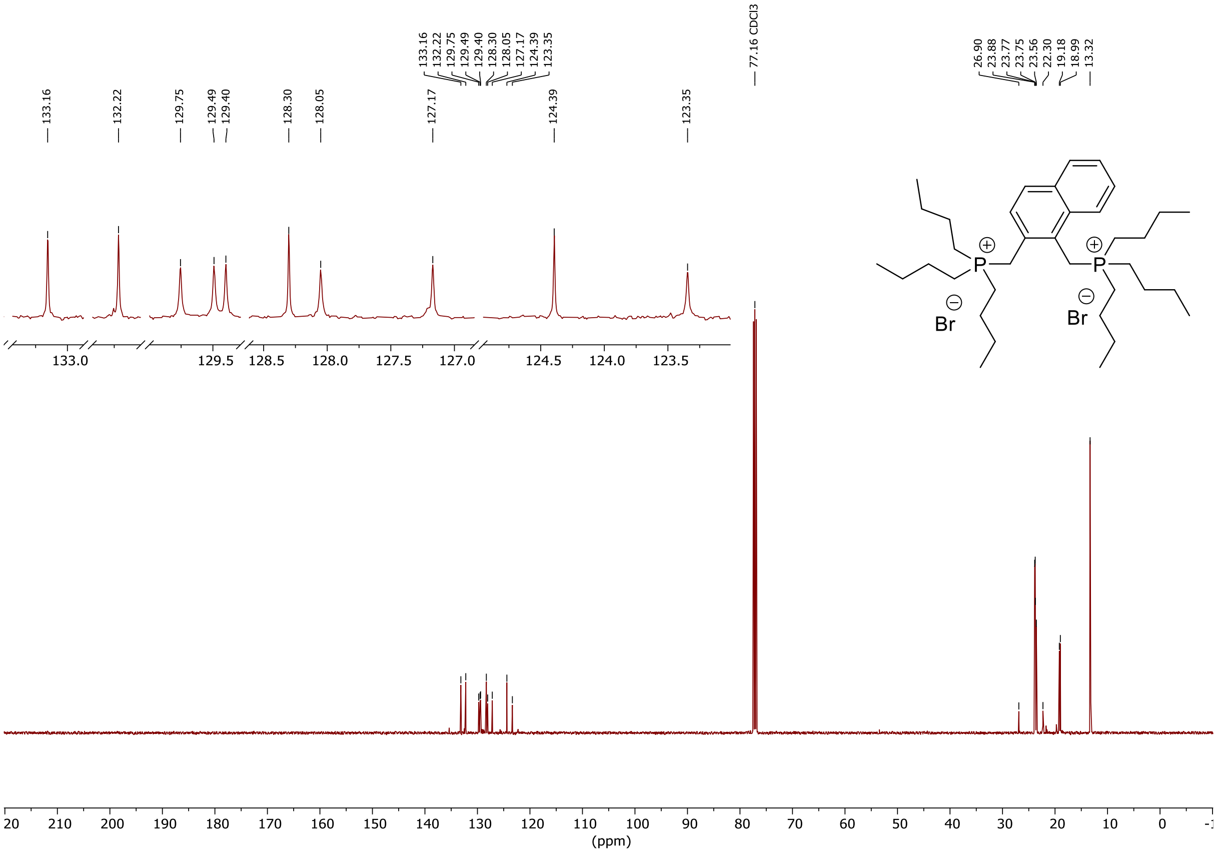

Fig. S127 $^{13}C\{^{1}H,^{31}P\}$ NMR (151 MHz, chloroform-*d*, 298 K) spectrum of compound **4f**

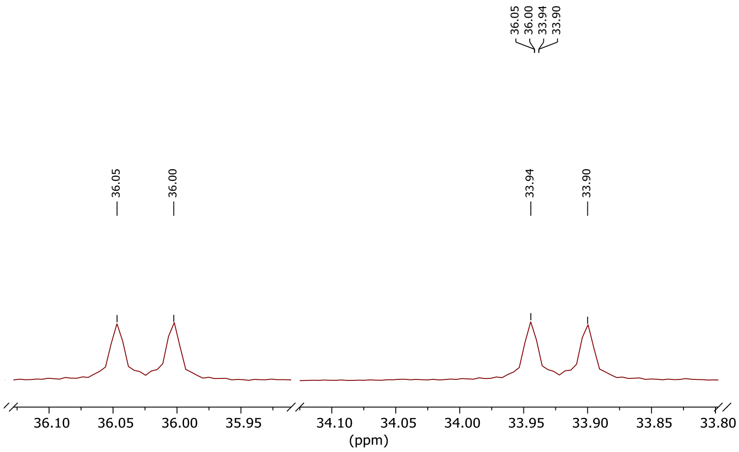

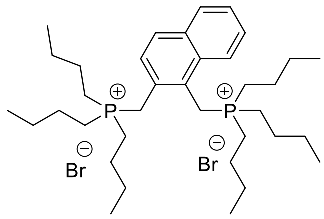

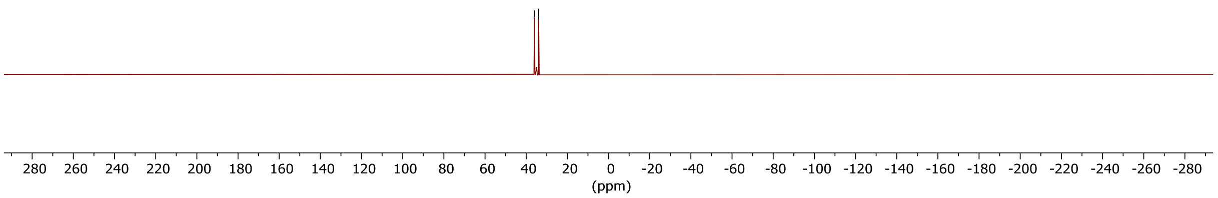

Fig. S128 ^{31}P NMR (243 MHz, chloroform-*d*, 298 K) spectrum of compound **4f**

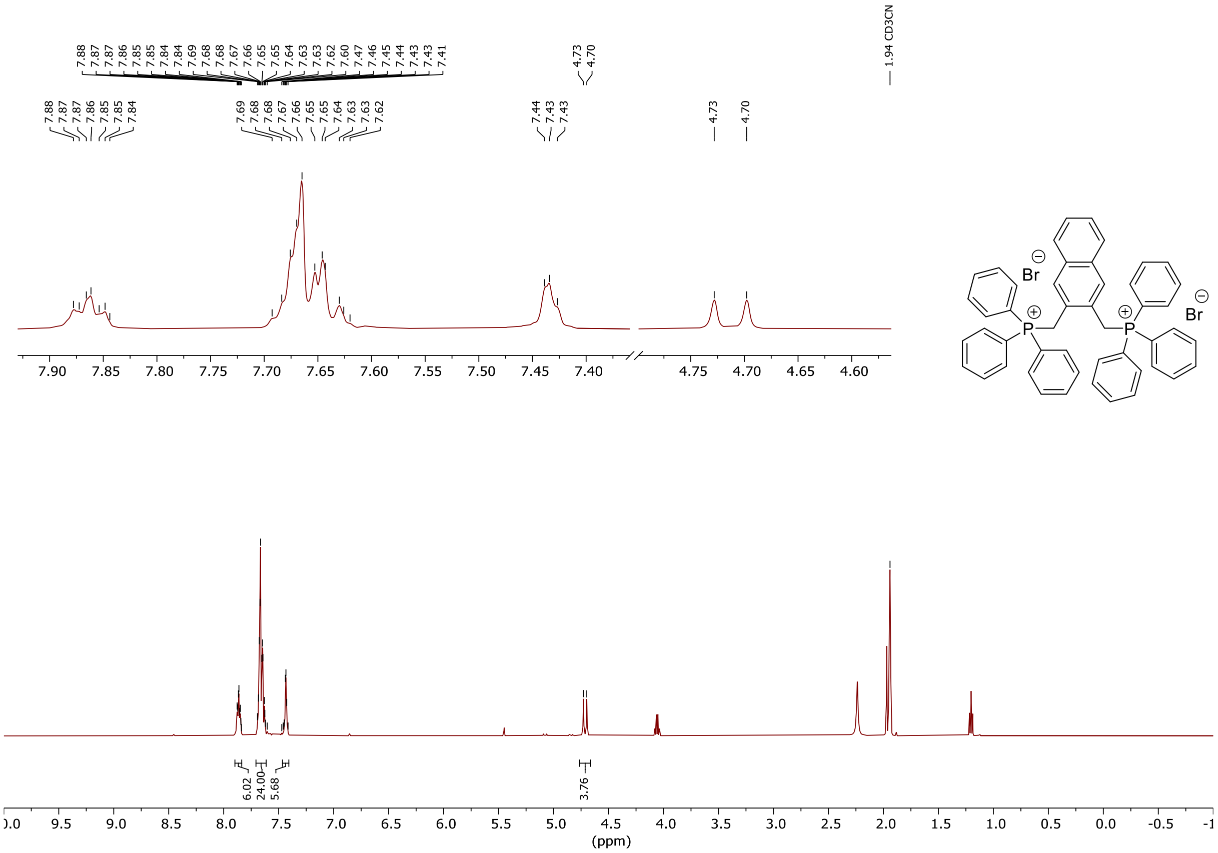

Fig. S129 ^{1}H NMR (501 MHz, acetonitrile-d_3, 298 K) spectrum of compound **4g**

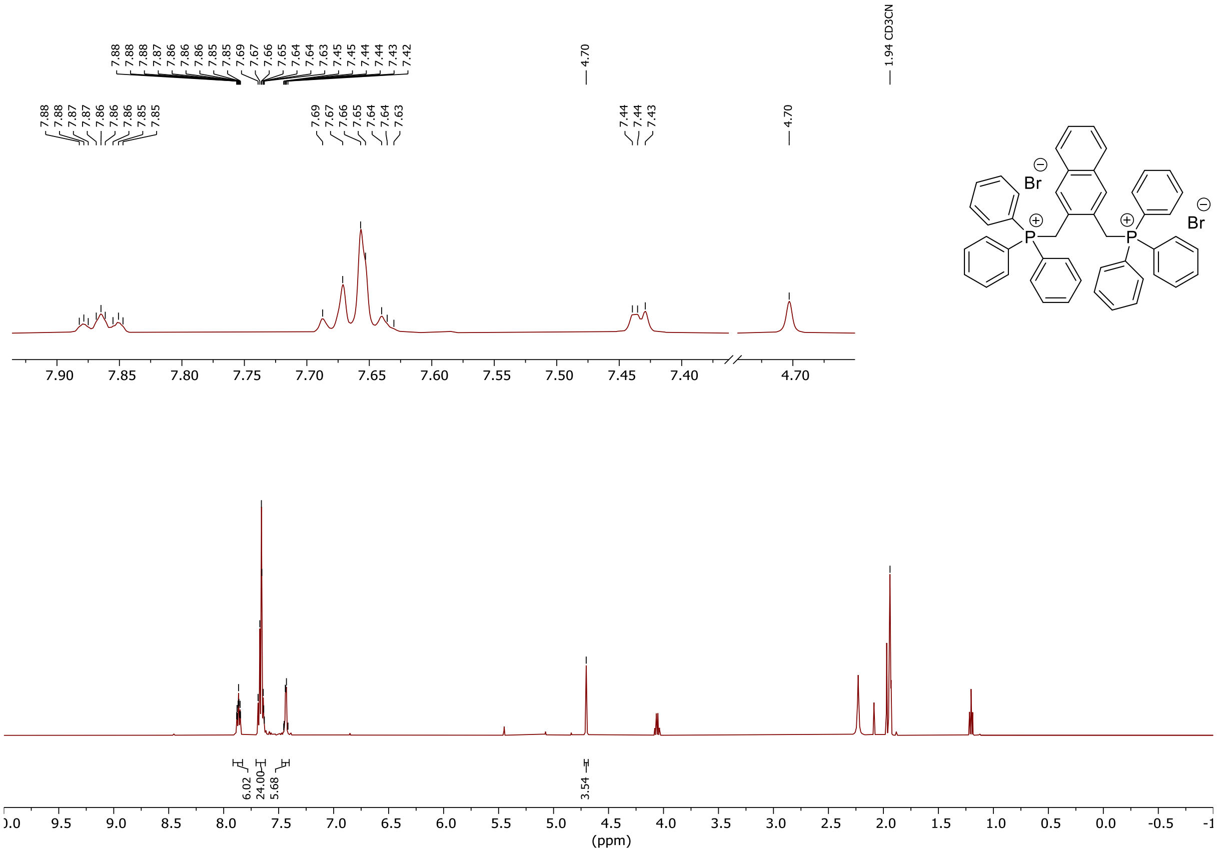

Fig. S130 ^{1}H{^{31}P} NMR (501 MHz, acetonitrile-d_3, 298 K) spectrum of compound **4g**

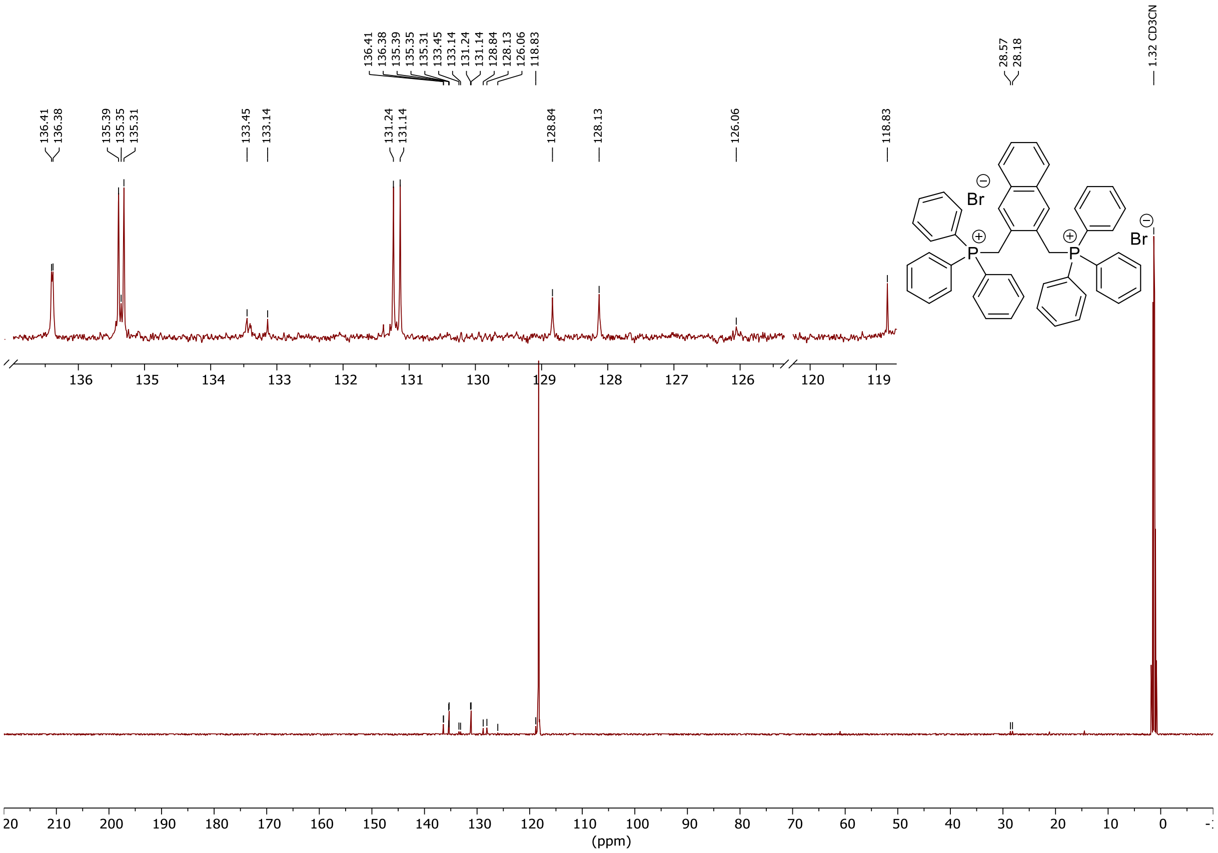

Fig. S131 $^{13}C\{^{1}H\}$ NMR (126 MHz, acetonitrile-d_3, 298 K) spectrum of compound **4g**

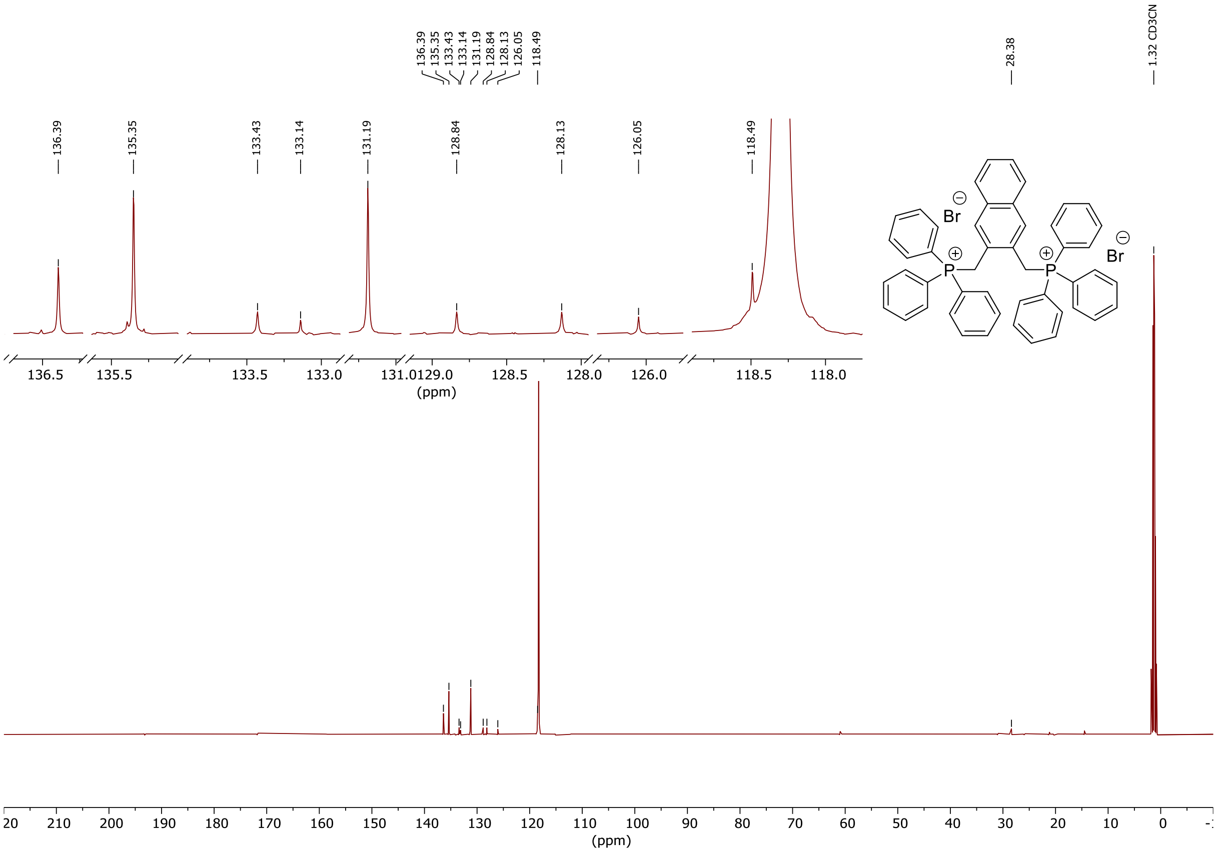

Fig. S132 $^{13}C\{^{1}H,^{31}P\}$ NMR (126 MHz, acetonitrile-d_3, 298 K) spectrum of compound **4g**

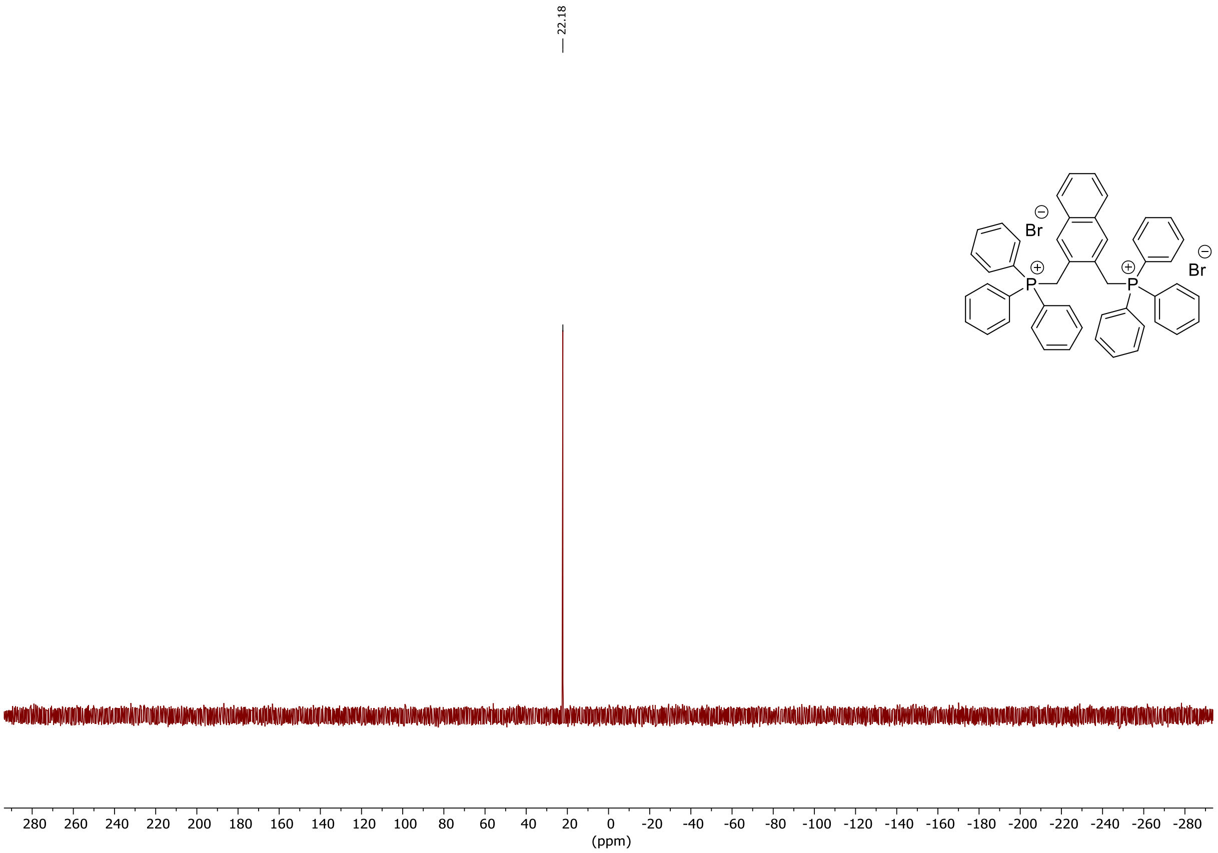

Fig. S133 ^{31}P NMR (203 MHz, acetonitrile-d_3, 298 K) spectrum of compound **4g**

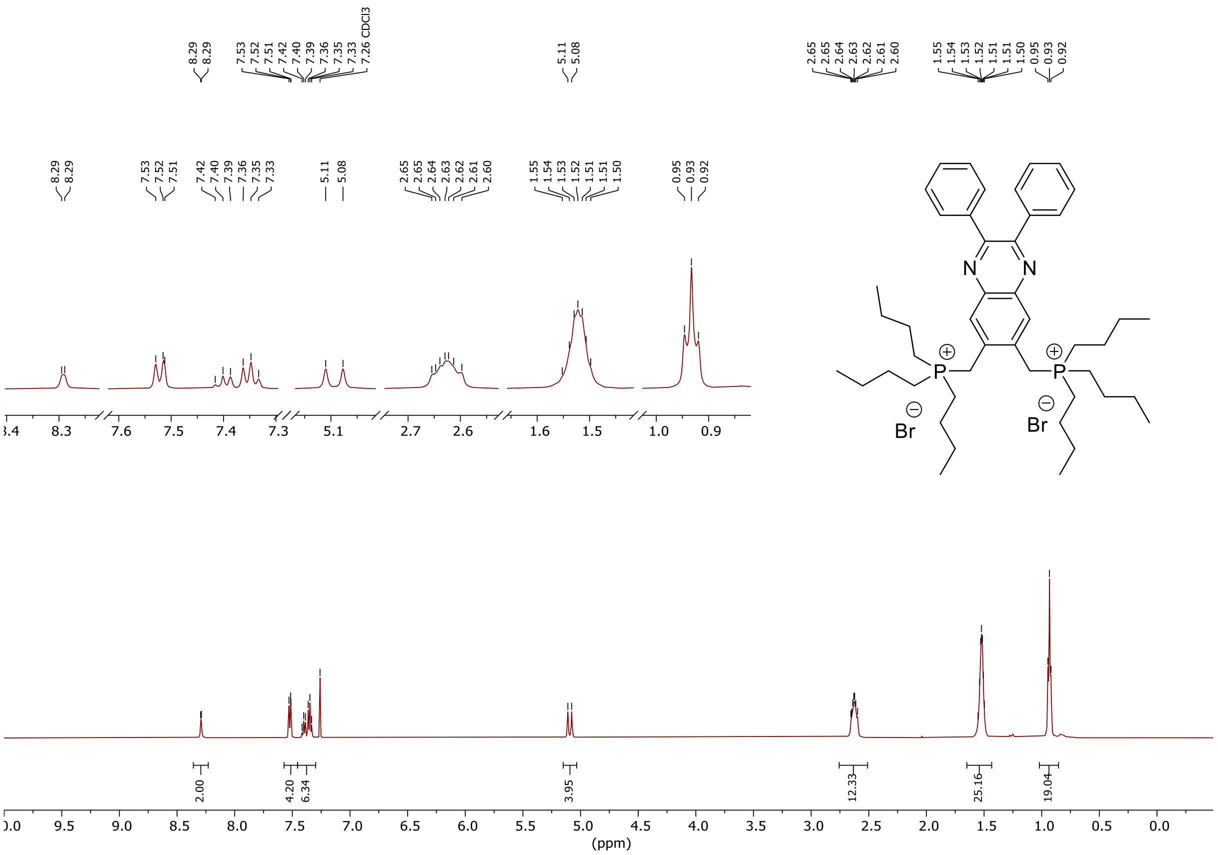

Fig. S134 ^{1}H NMR (501 MHz, chloroform-*d*, 298 K) spectrum of compound **4h**

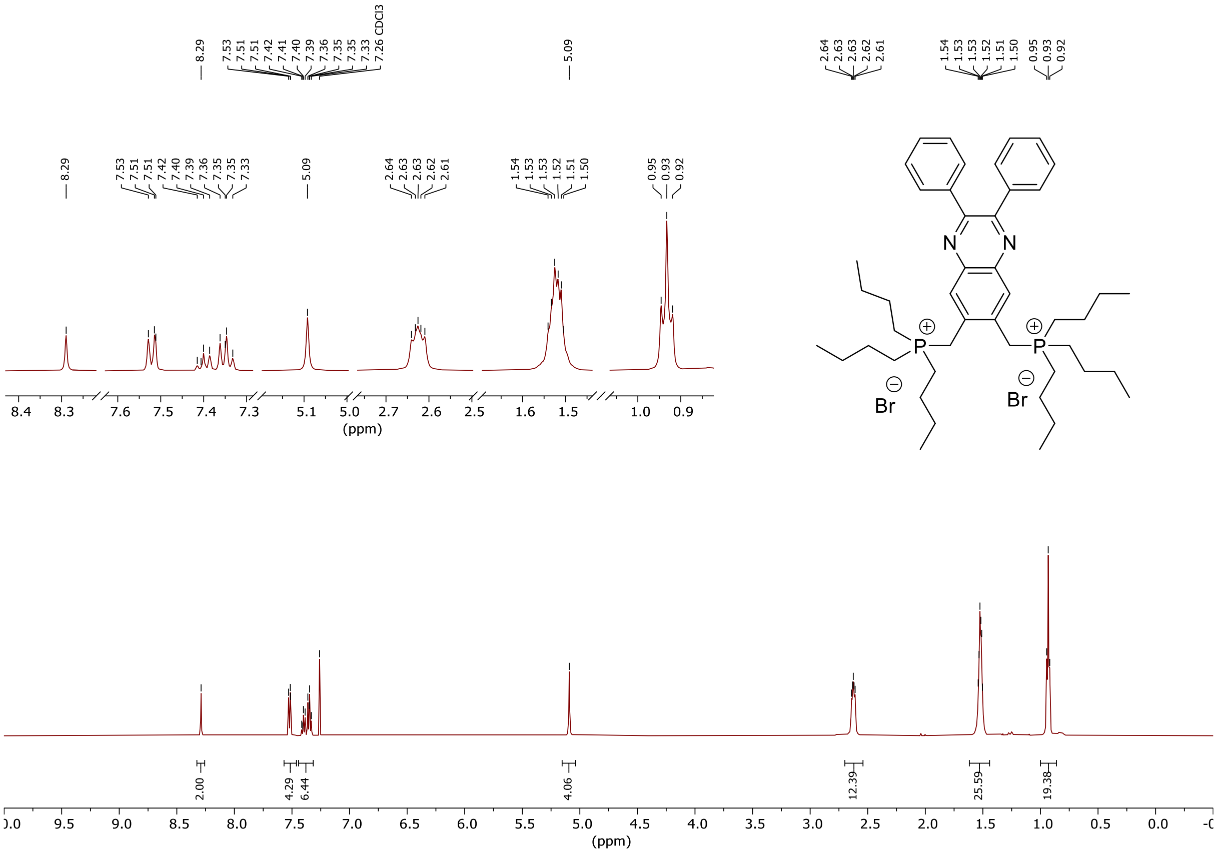

Fig. S135 ^{1}H{^{31}P} NMR (501 MHz, chloroform-*d*, 298 K) spectrum of compound **4h**

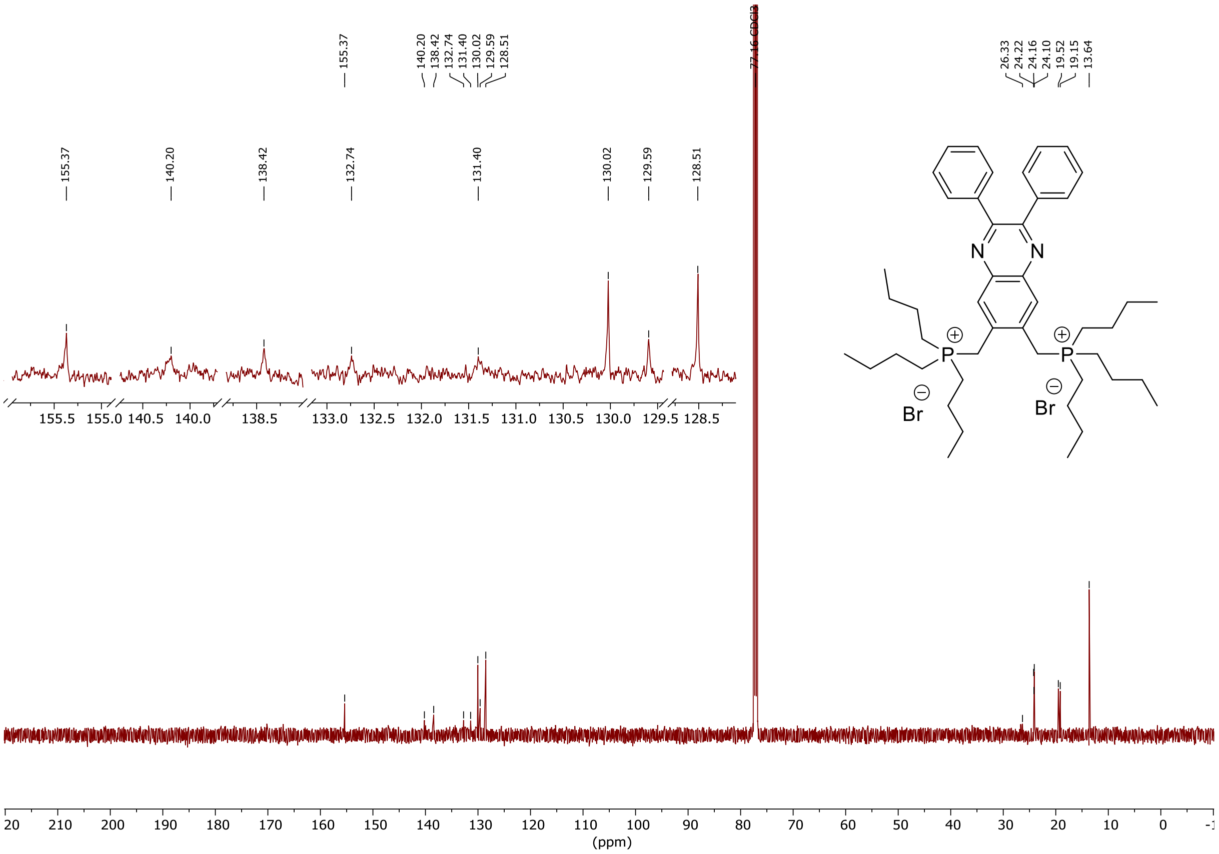

Fig. S136 $^{13}C\{^{1}H\}$ NMR (125 MHz, chloroform-*d*, 298 K) spectrum of compound **4h**

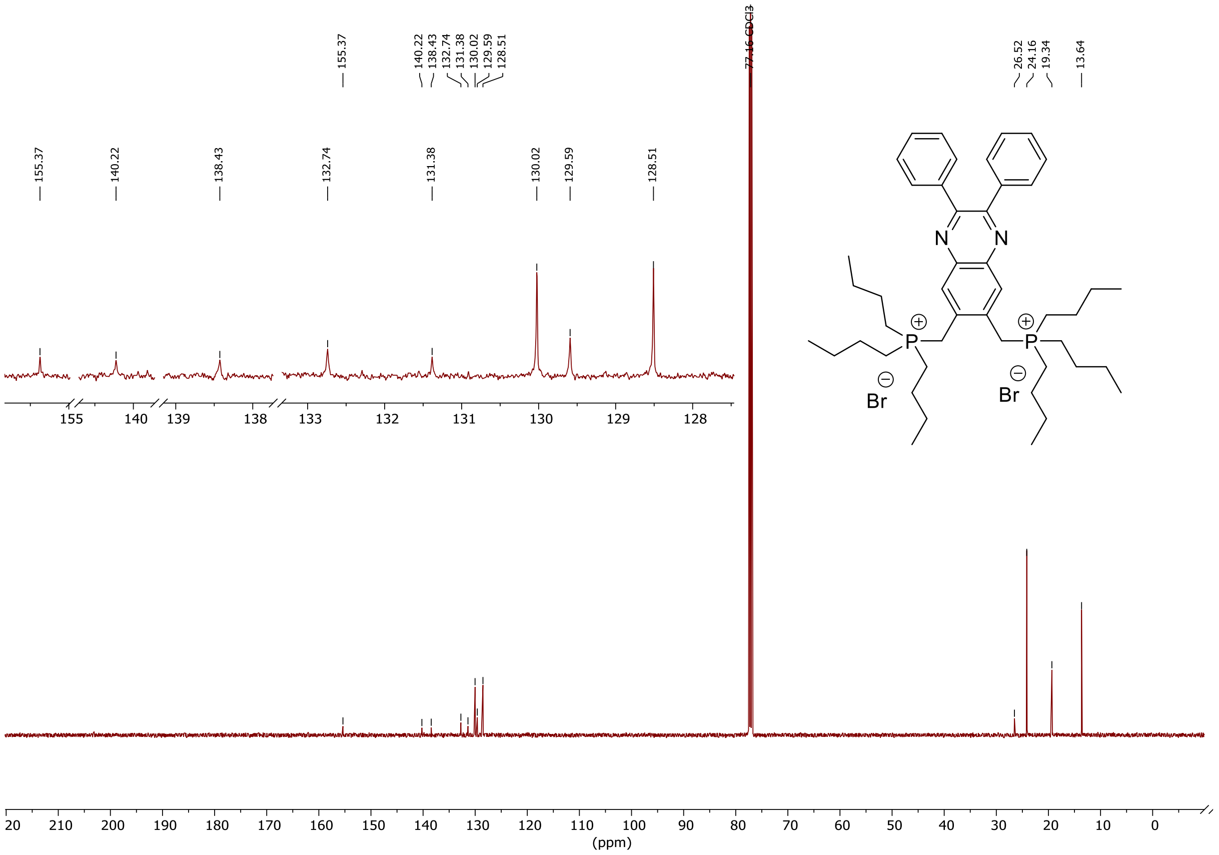

Fig. S137 $^{13}C\{^{1}H,^{31}P\}$ NMR (125 MHz, chloroform-*d*, 298 K) spectrum of compound **4h**

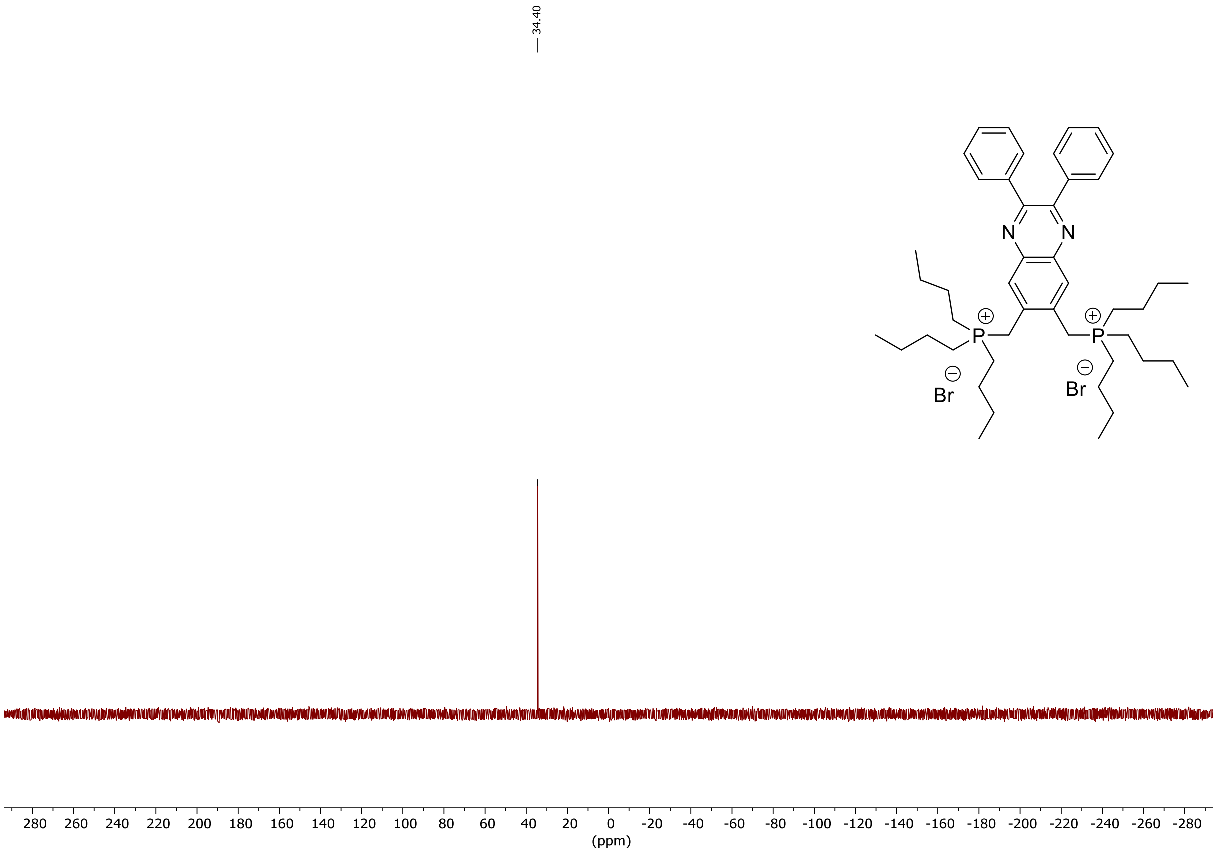

Fig. S138 ^{31}P NMR (203 MHz, chloroform-*d*, 298 K) spectrum of compound **4h**

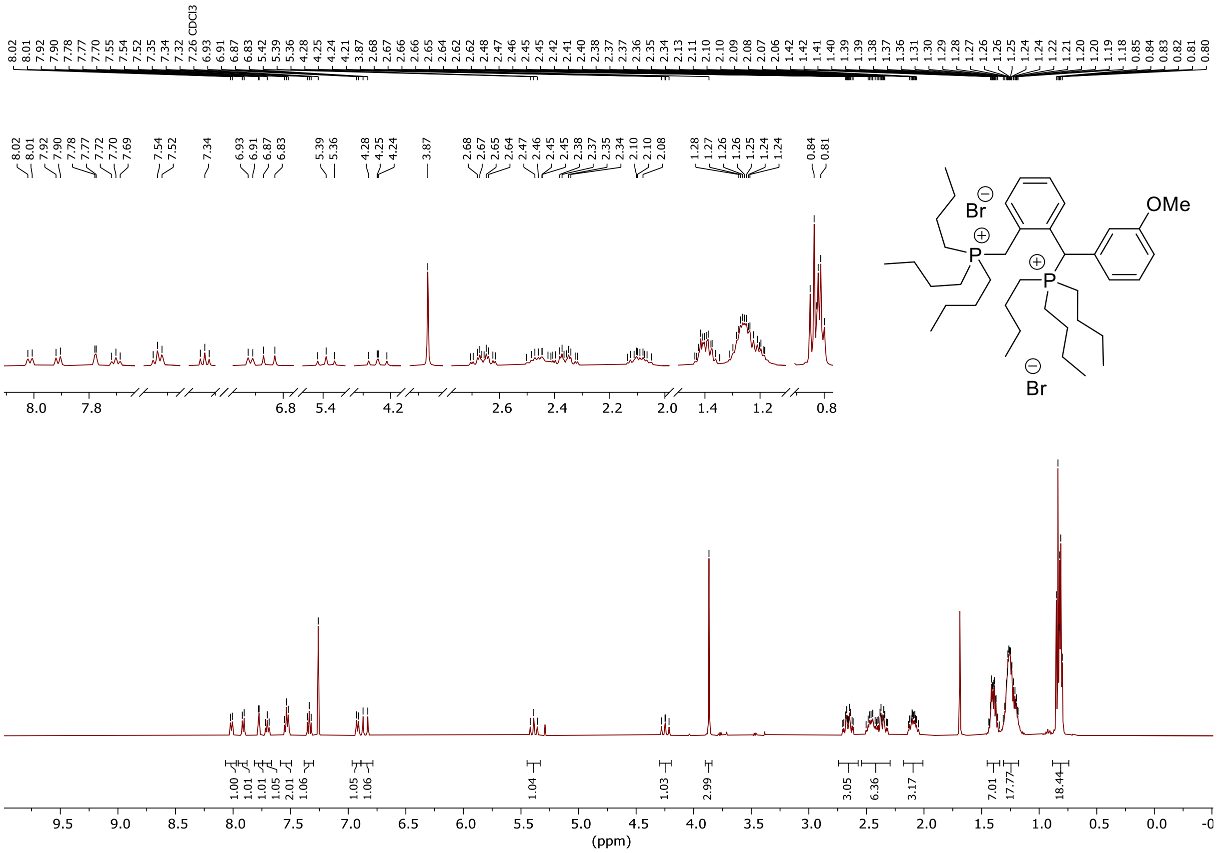

Fig. S139 ^{1}H NMR (501 MHz, chloroform-*d*, 298 K) spectrum of compound **4h**

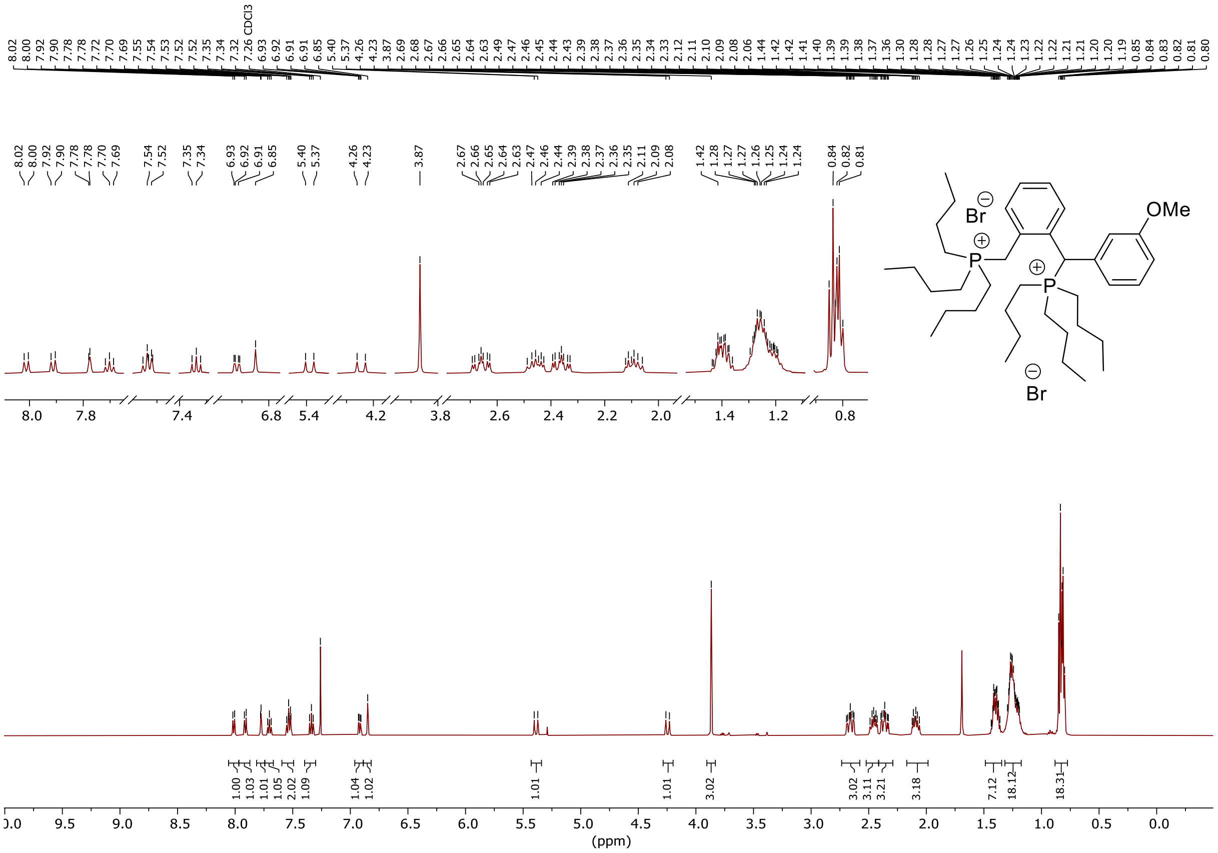

Fig. S140 ^{1}H{^{31}P} NMR (501 MHz, chloroform-*d*, 298 K) spectrum of compound **4h**

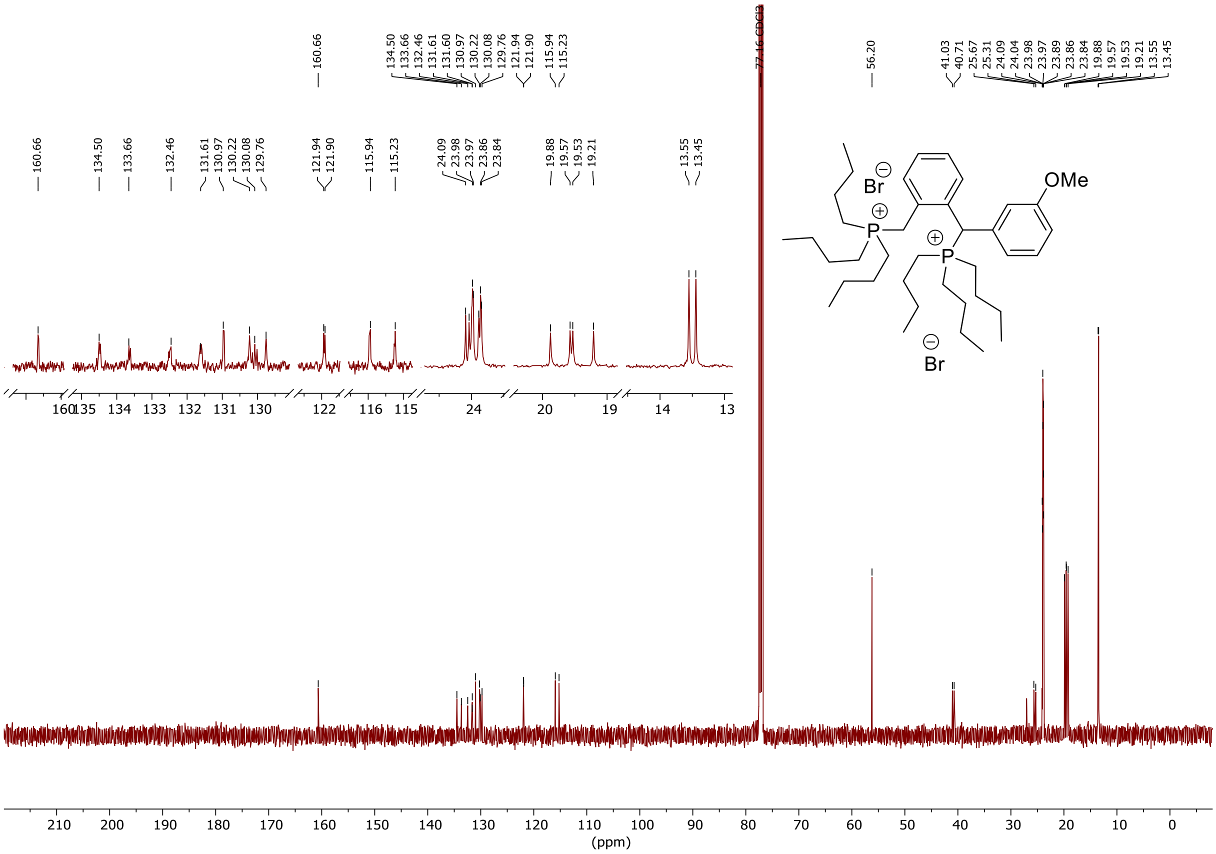

Fig. S141 $^{13}C\{^{1}H\}$ NMR (126 MHz, chloroform-*d*, 298 K) spectrum of compound **4h**

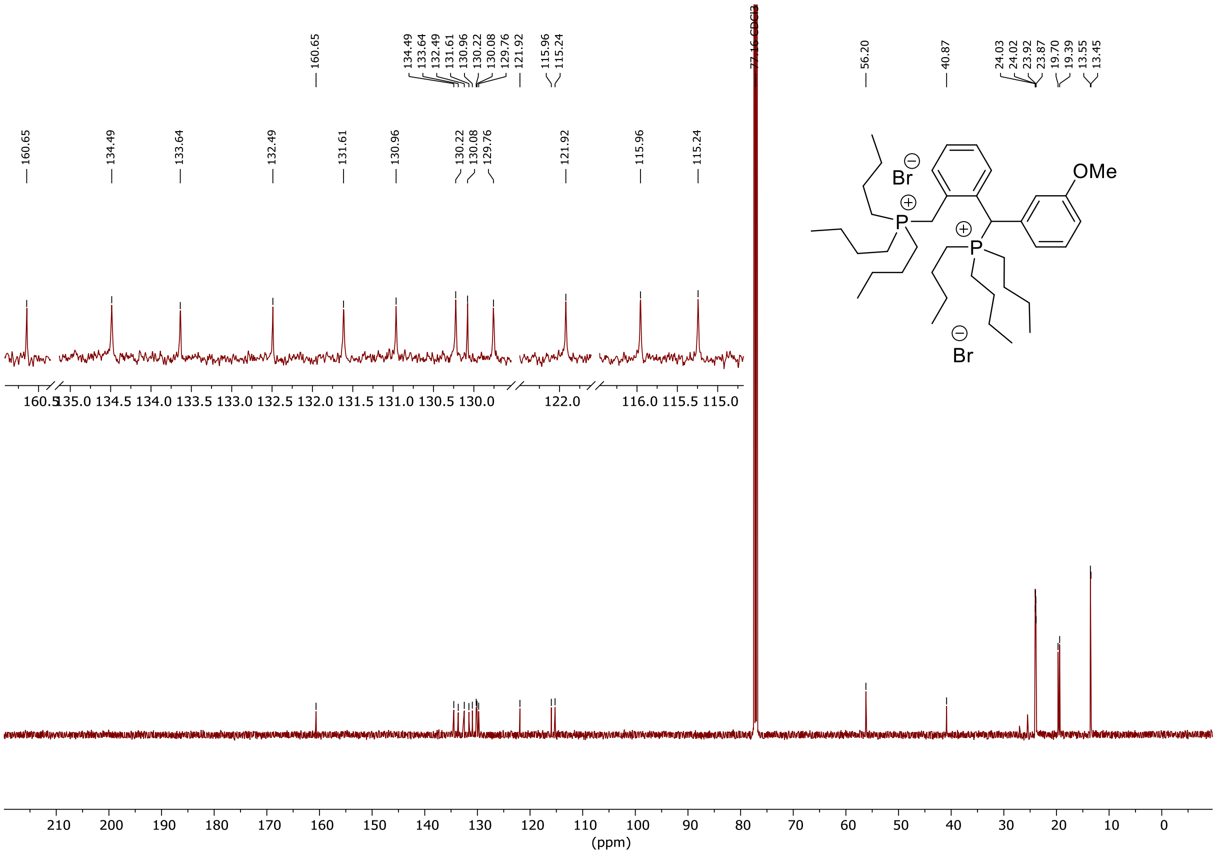

Fig. S142 $^{13}C\{^{1}H,^{31}P\}$ NMR (126 MHz, chloroform-*d*, 298 K) spectrum of compound **4h**

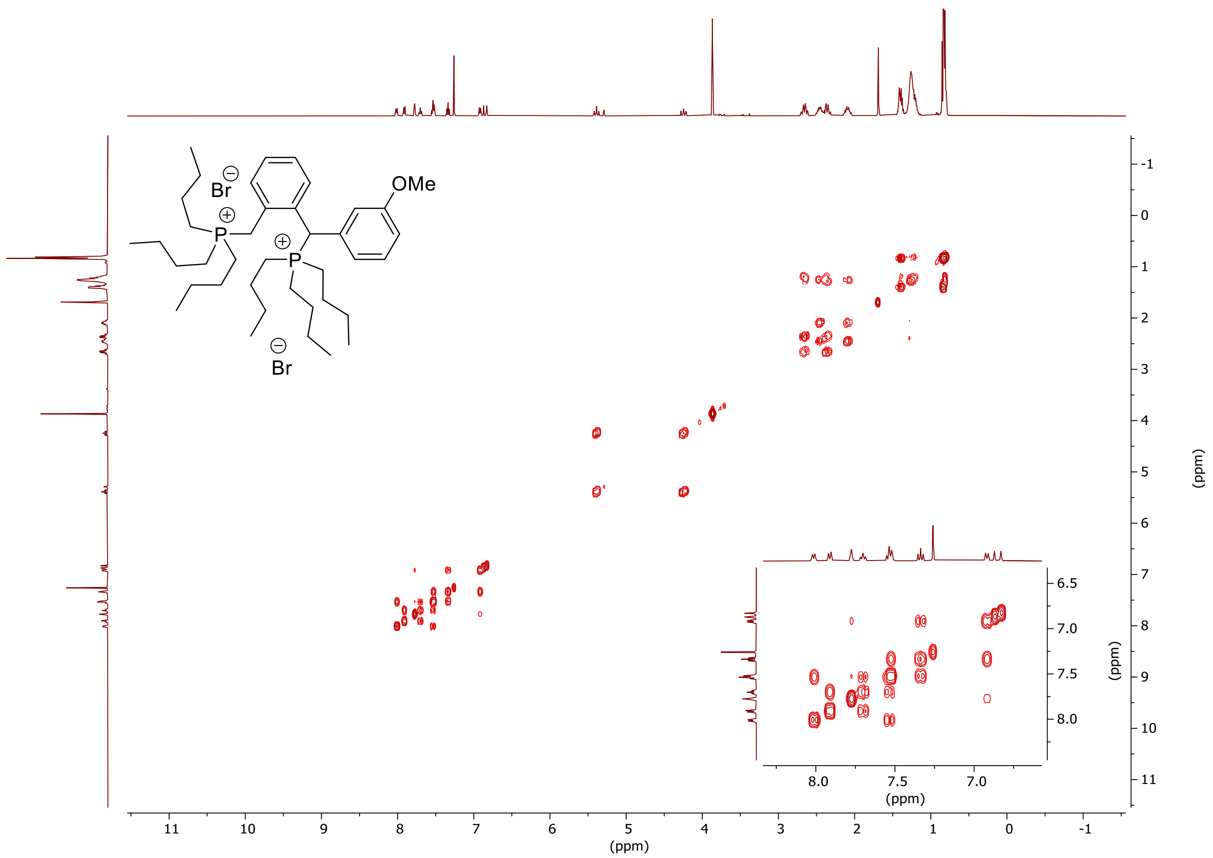

Fig. S143 COSY (501 MHz, 126 MHz, chloroform-*d*, 298 K) spectrum of compound **4h**

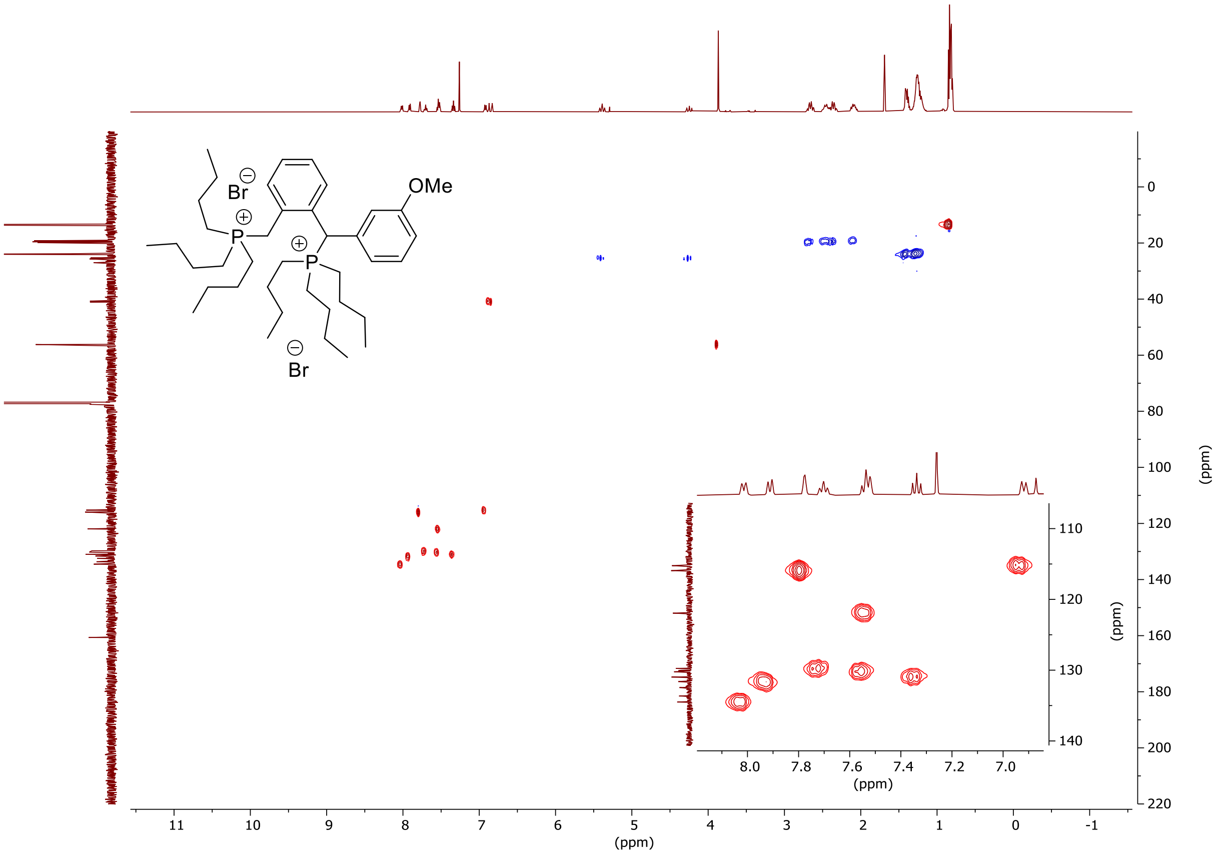

Fig. S144 HSQC (501 MHz, 126 MHz, chloroform-*d*, 298 K) spectrum of compound **4h**

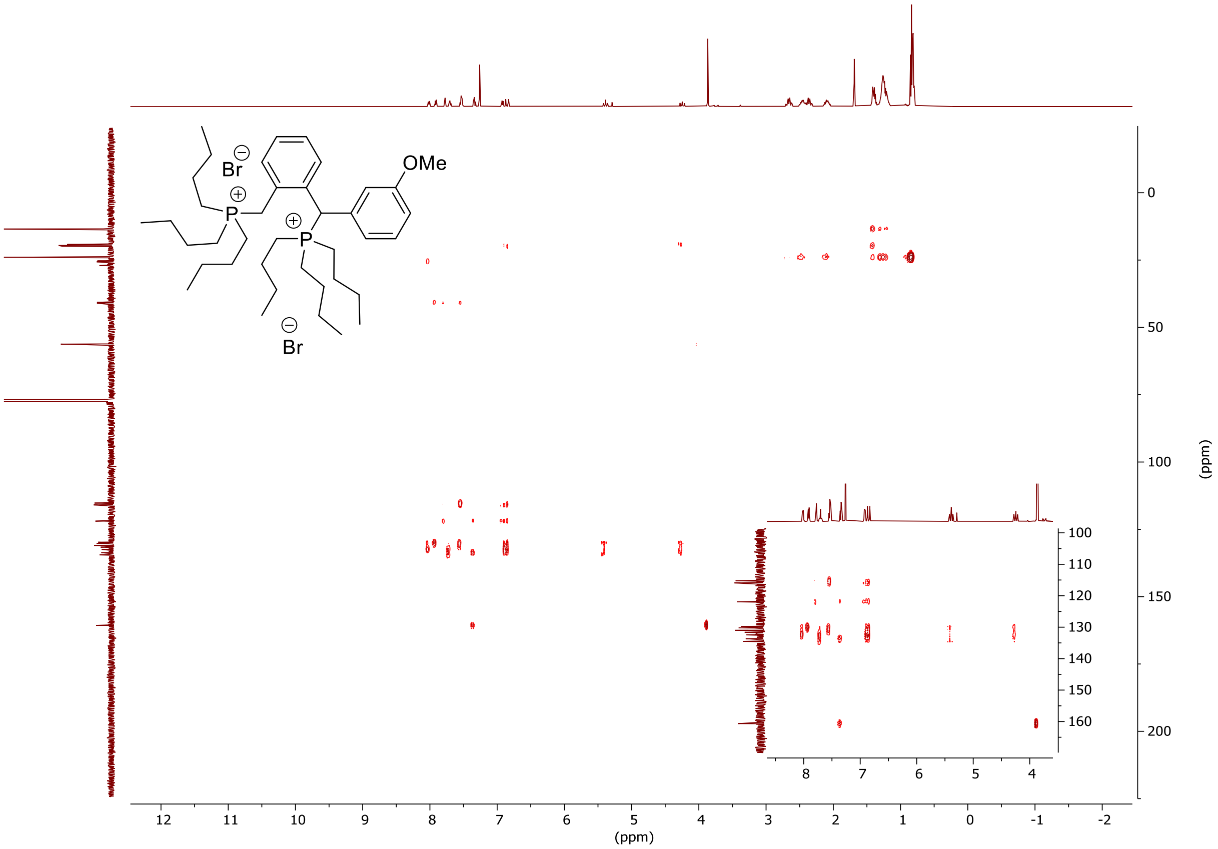

Fig. S145 HMBC (501 MHz, 126 MHz, chloroform-*d*, 298 K) spectrum of compound **4h**

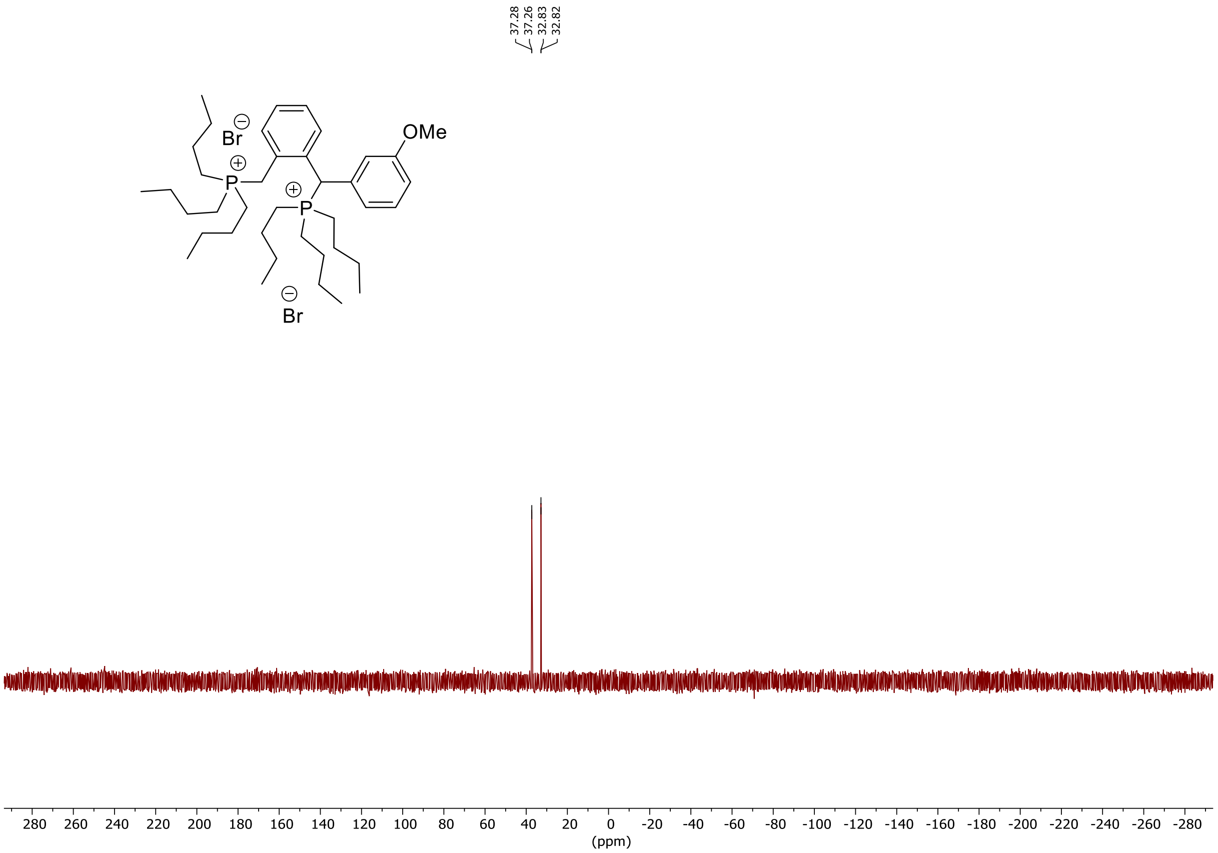

Fig. S146 ^{31}P NMR (203 MHz, chloroform-*d*, 298 K) spectrum of compound **4h**

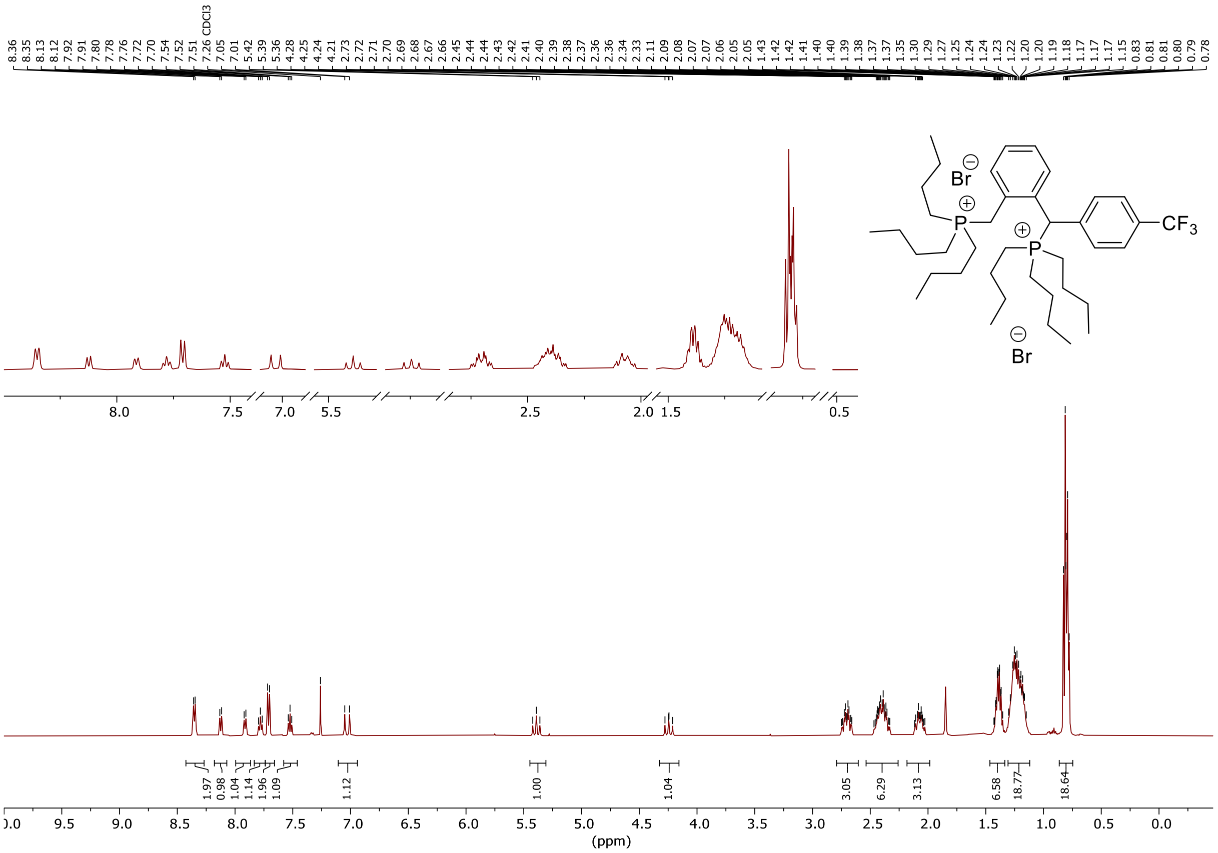

Fig. S147 ^{1}H NMR (501 MHz, chloroform-*d*, 298 K) spectrum of compound **4i**

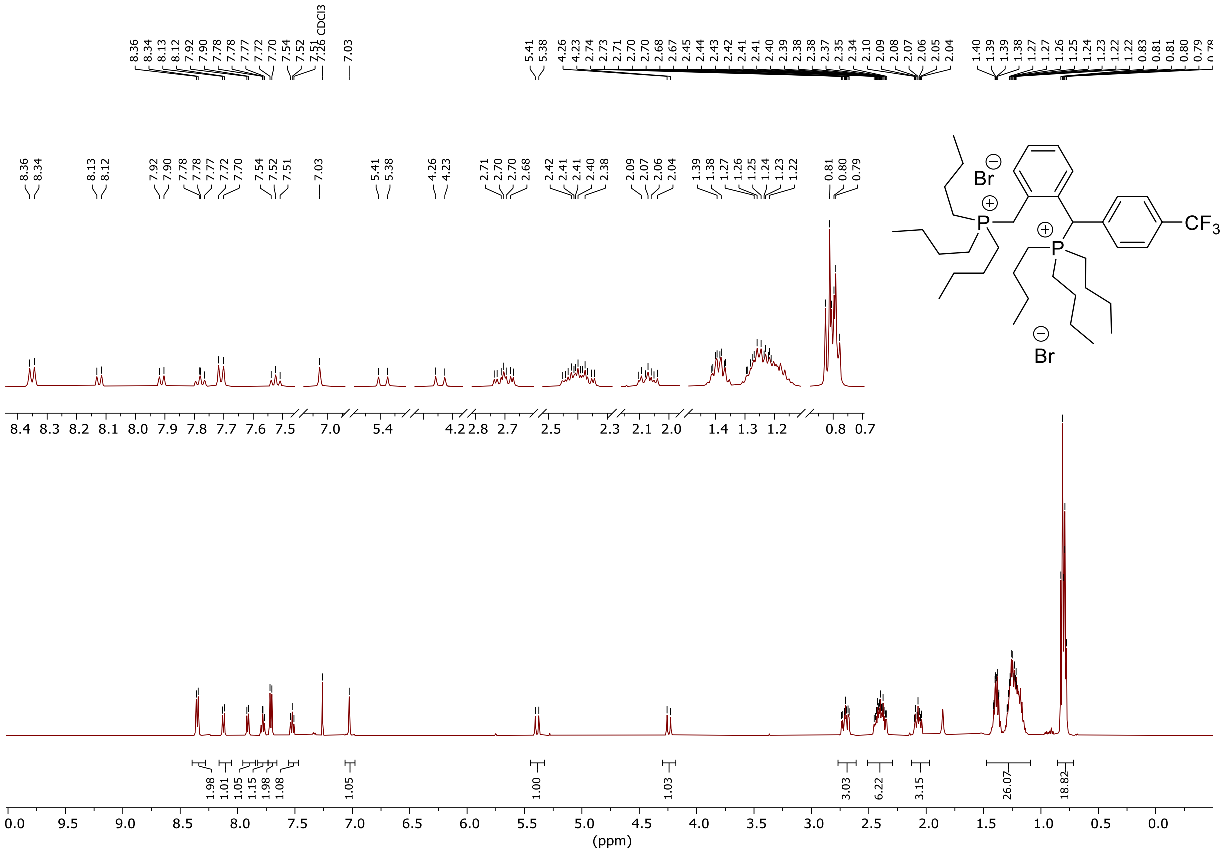

Fig. S148 $^{1}H\{^{31}P\}$ NMR (501 MHz, chloroform-*d*, 298 K) spectrum of compound **4i**

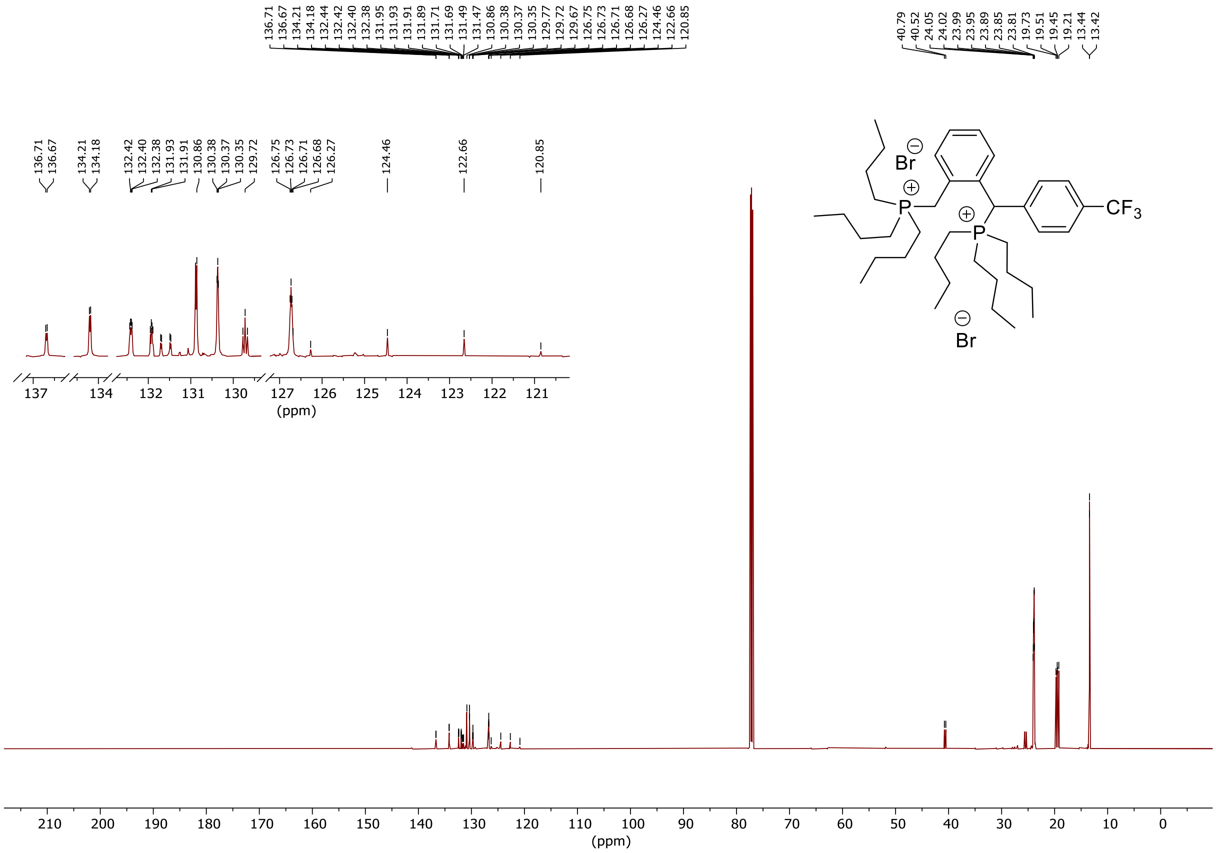

Fig. S149 $^{13}C\{^{1}H\}$ NMR (126 MHz, chloroform-*d*, 298 K) spectrum of compound **4i**

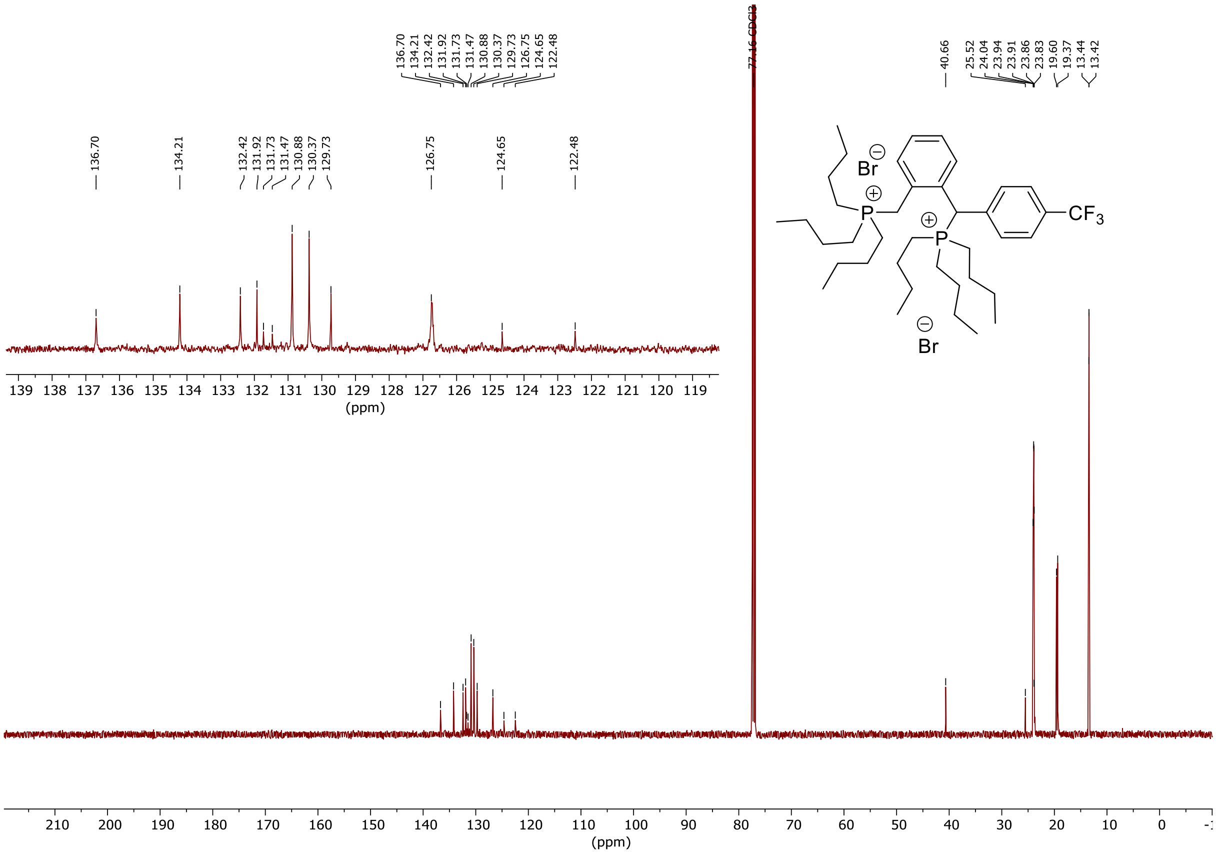

Fig. S150 $^{13}C\{^{1}H,^{31}P\}$ NMR (126 MHz, chloroform-*d*, 298 K) spectrum of compound **4i**

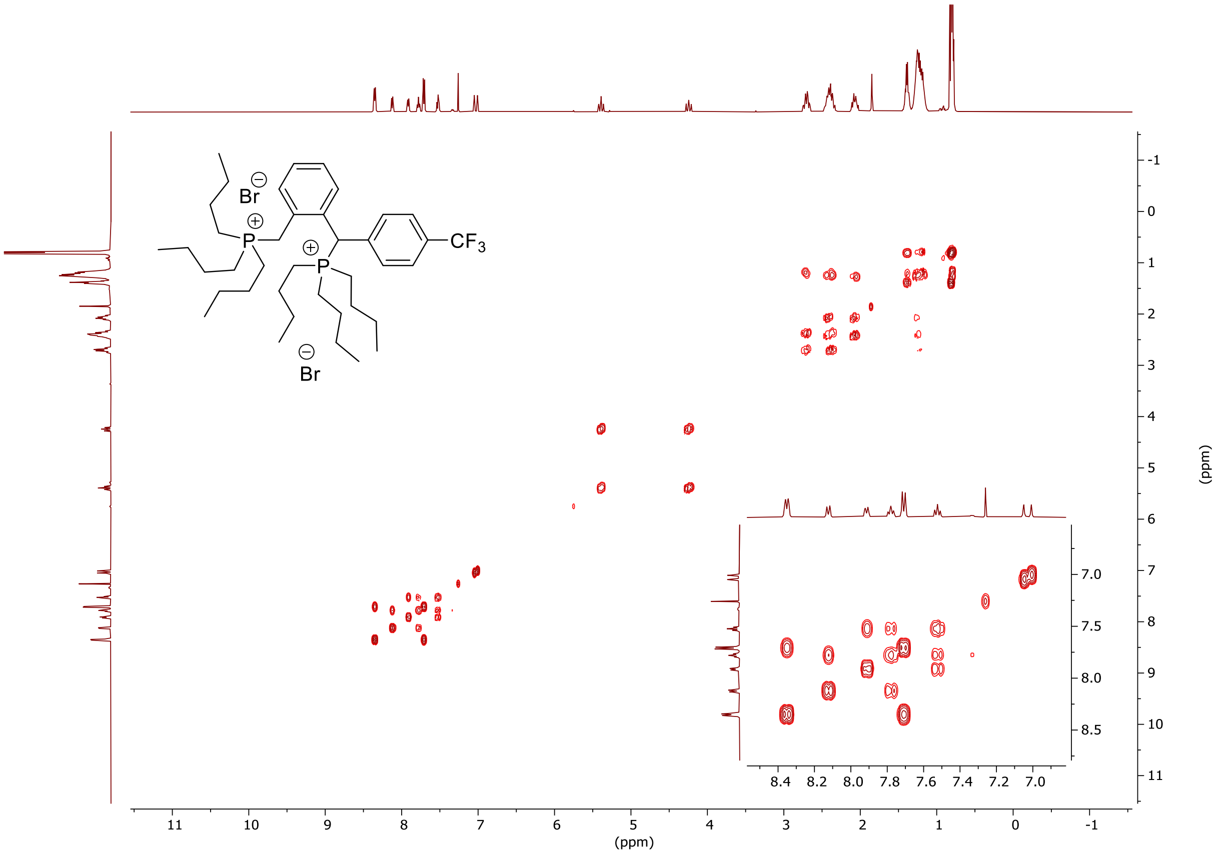

Fig. S151 COSY (501 MHz, 501 MHz, chloroform-*d*, 298 K) spectrum of compound **4i**

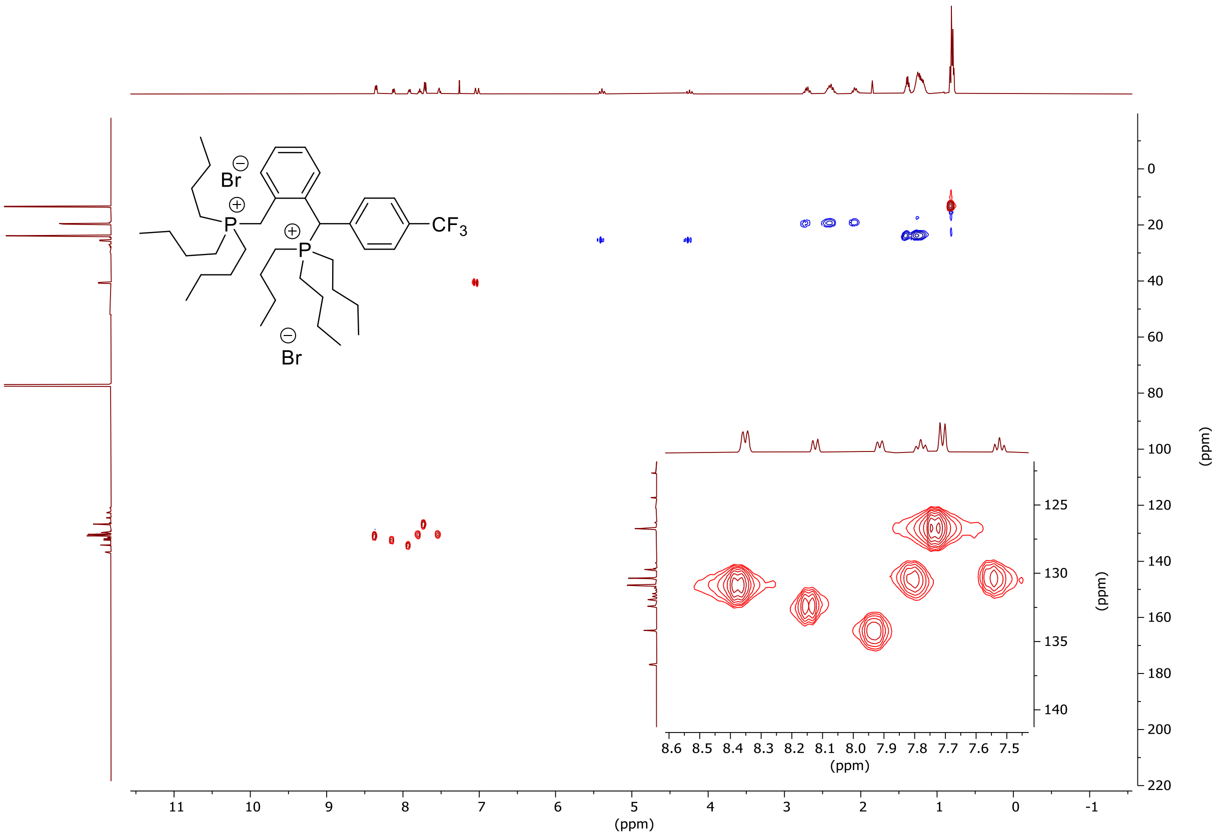

Fig. S152 HSQC (501 MHz, 126 MHz, chloroform-*d*, 298 K) spectrum of compound **4i**

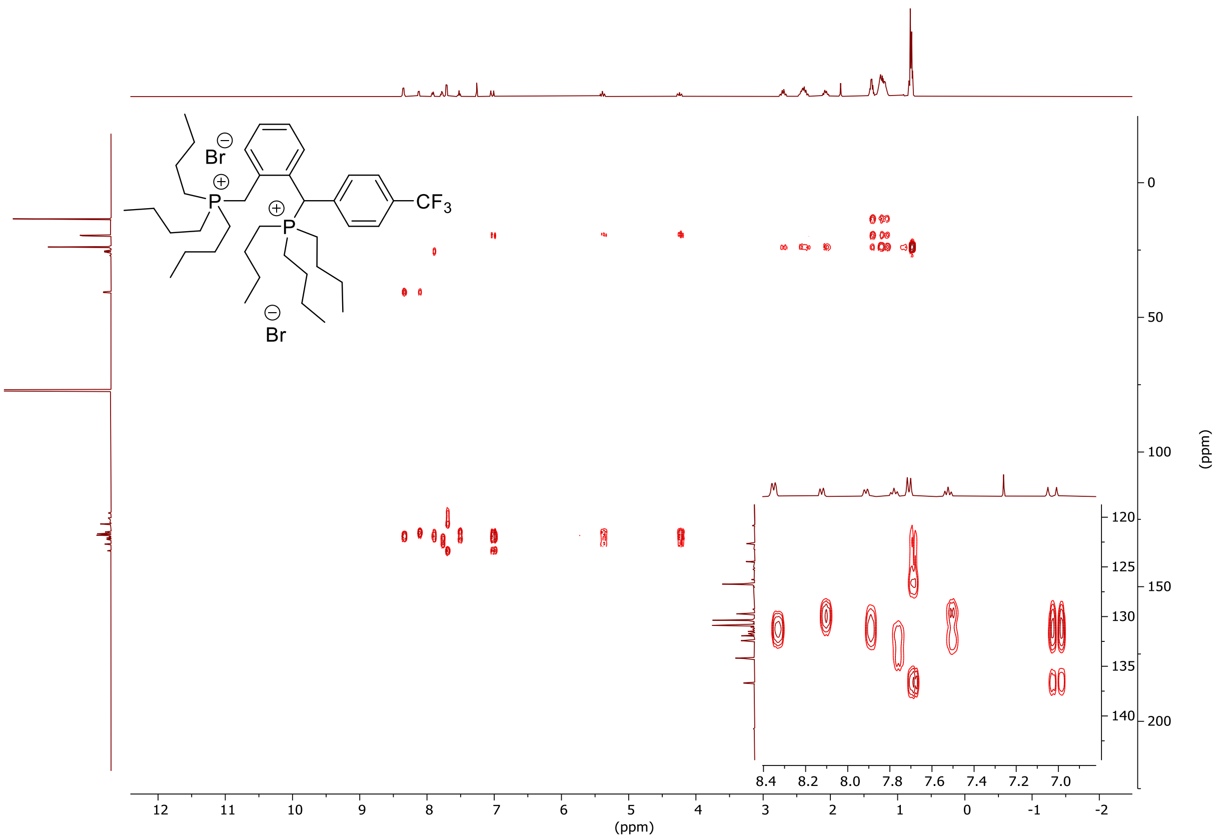

Fig. S153 HMBC (500 MHz, 126 MHz, chloroform-*d*, 298 K) spectrum of compound **4i**

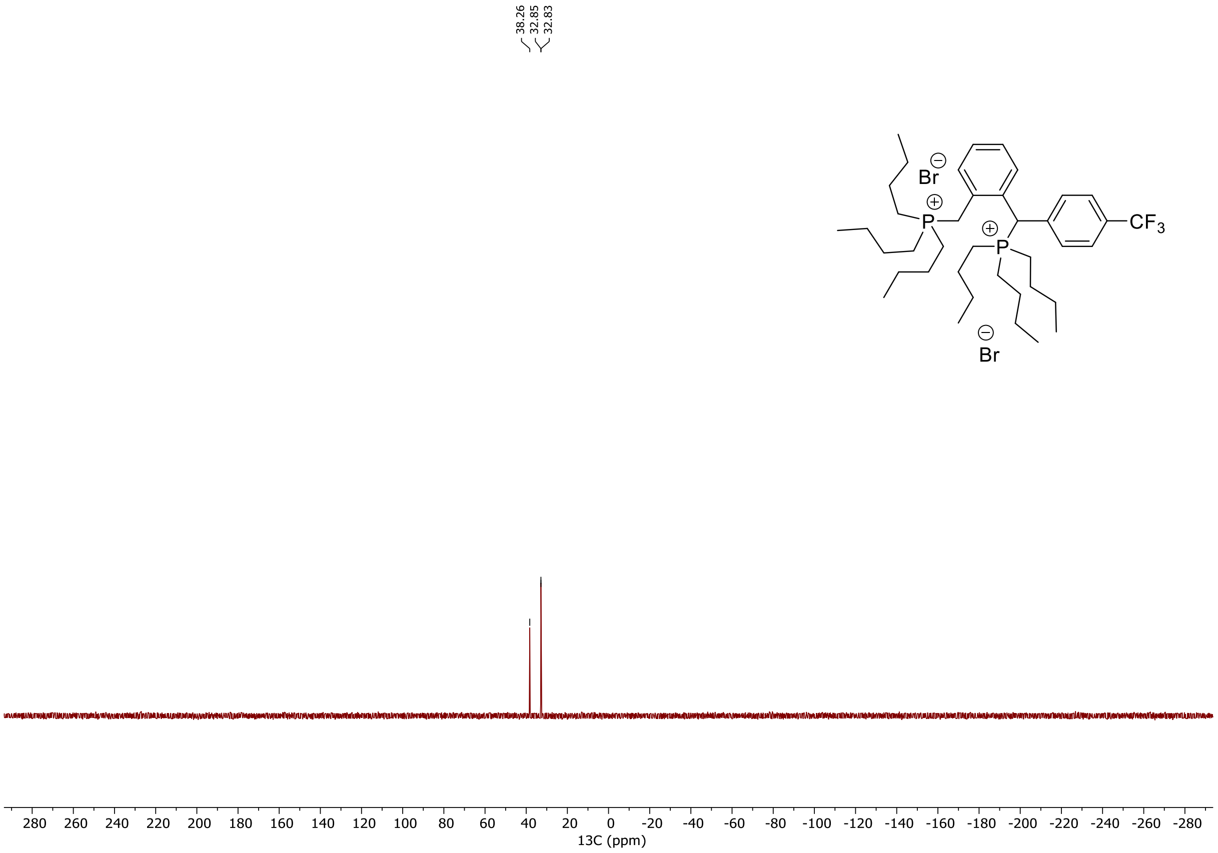

Fig. S154 ^{31}P NMR (203 MHz, chloroform-*d*, 298 K) spectrum of compound **4i**

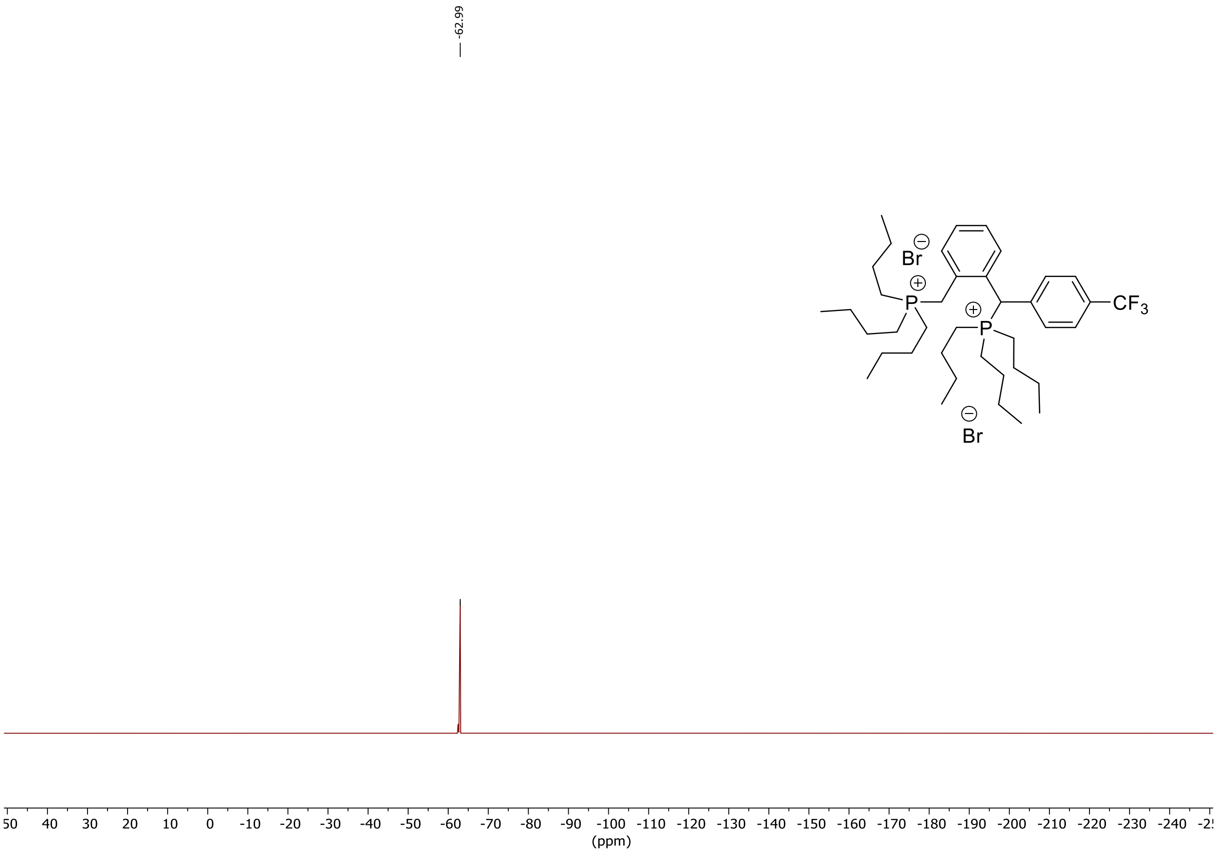

Fig. S155 ^{19}F NMR (565 MHz, chloroform-*d*, 298 K) spectrum of compound **4i**

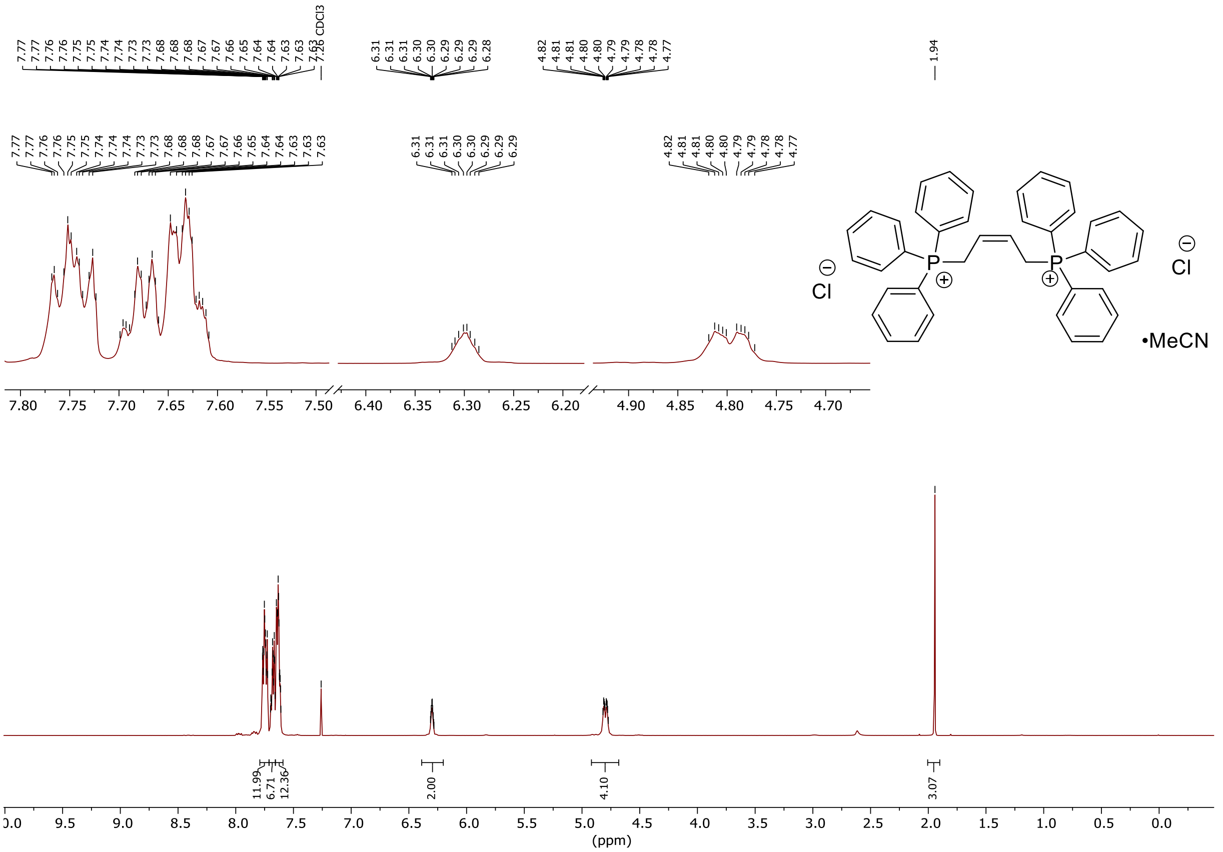

Fig. S156 ^{1}H NMR (501 MHz, chloroform-*d*, 298 K) spectrum of compound **4j**

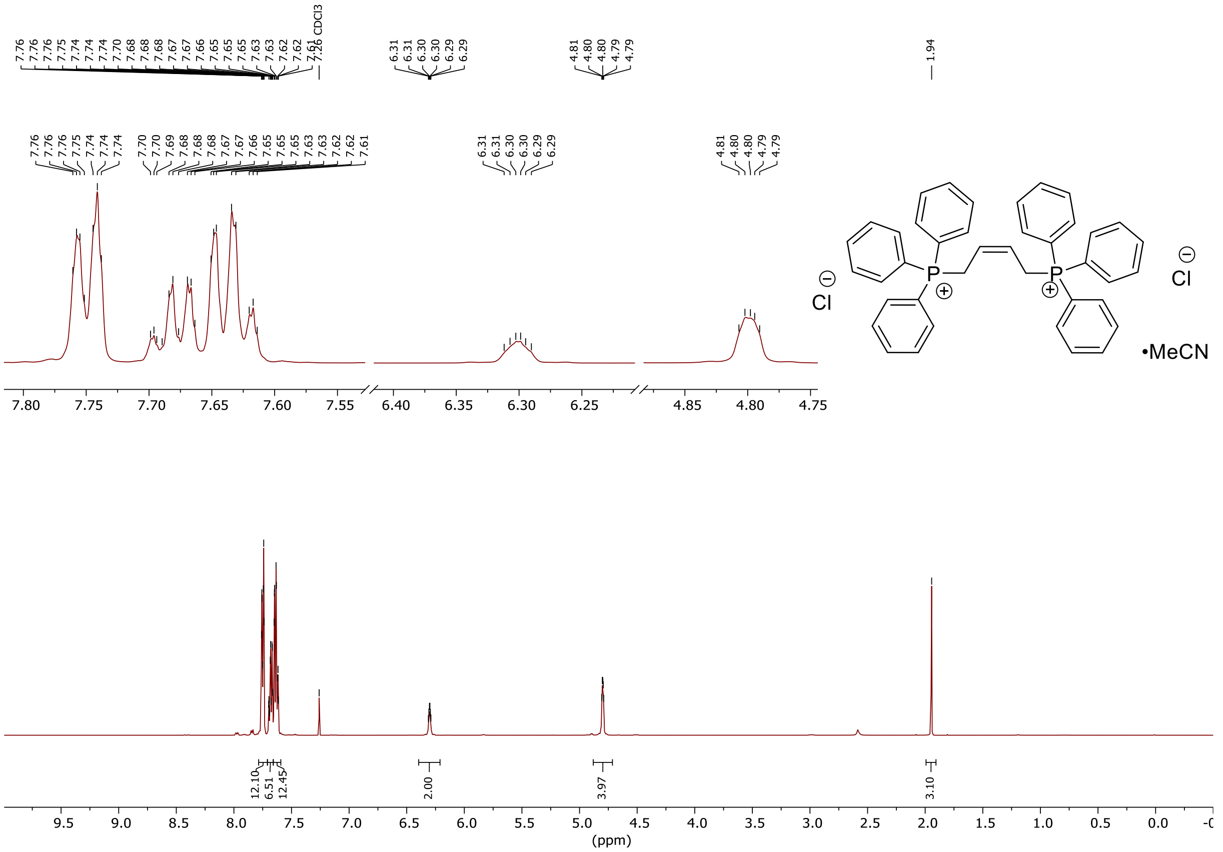

Fig. S157 ^{1}H{^{31}P} NMR (501 MHz, chloroform-*d*, 298 K) spectrum of compound **4j**

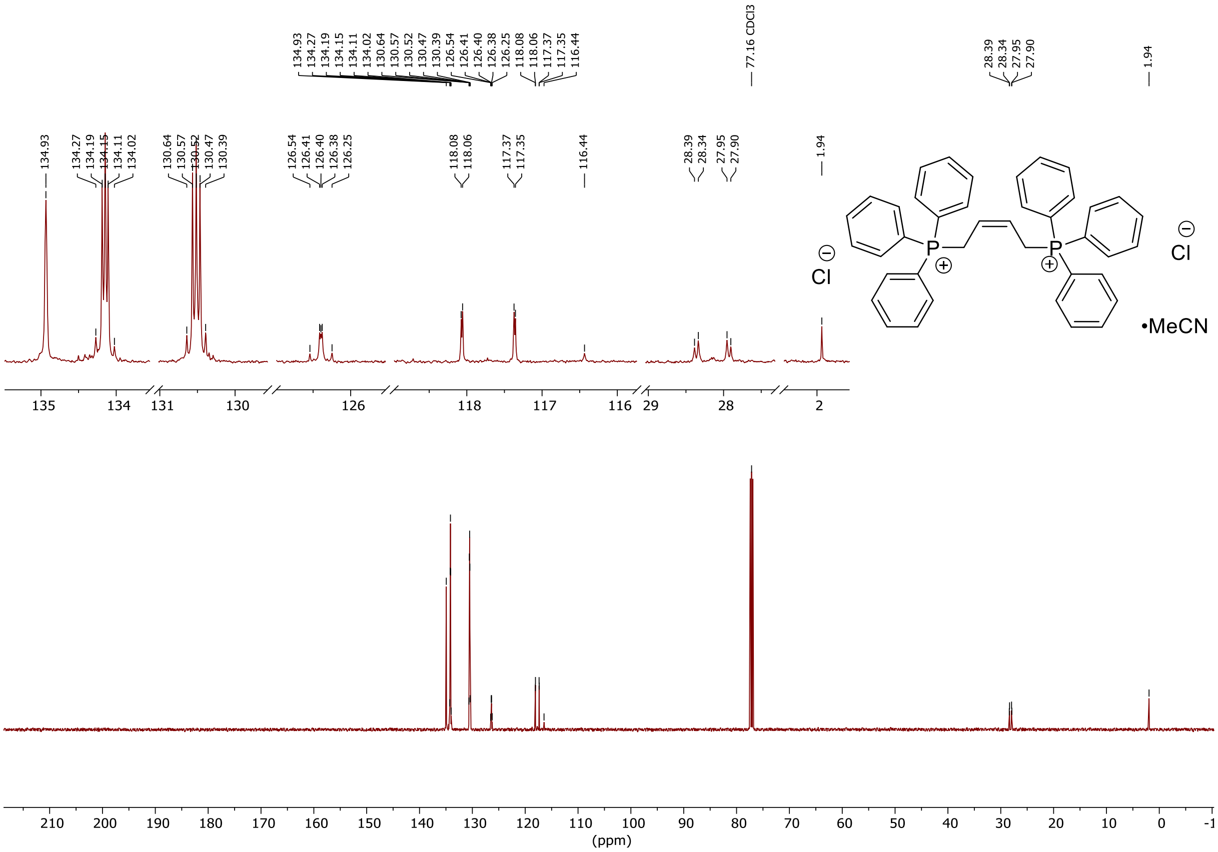

Fig. S158 $^{13}C\{^1H\}$ NMR (126 MHz, chloroform-*d*, 298 K) spectrum of compound **4j**

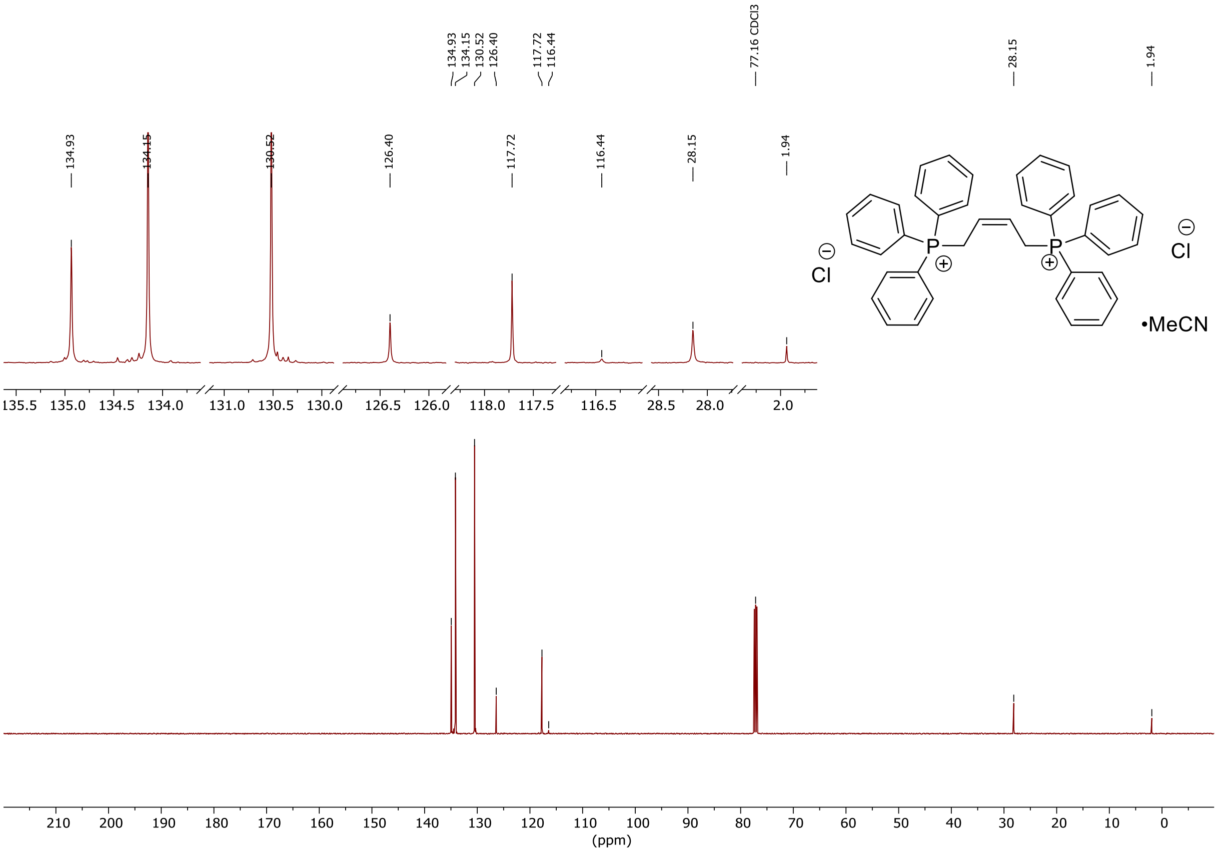

Fig. S159 $^{13}C\{^1H,^{31}P\}$ NMR (126 MHz, chloroform-*d*, 298 K) spectrum of compound **4j**

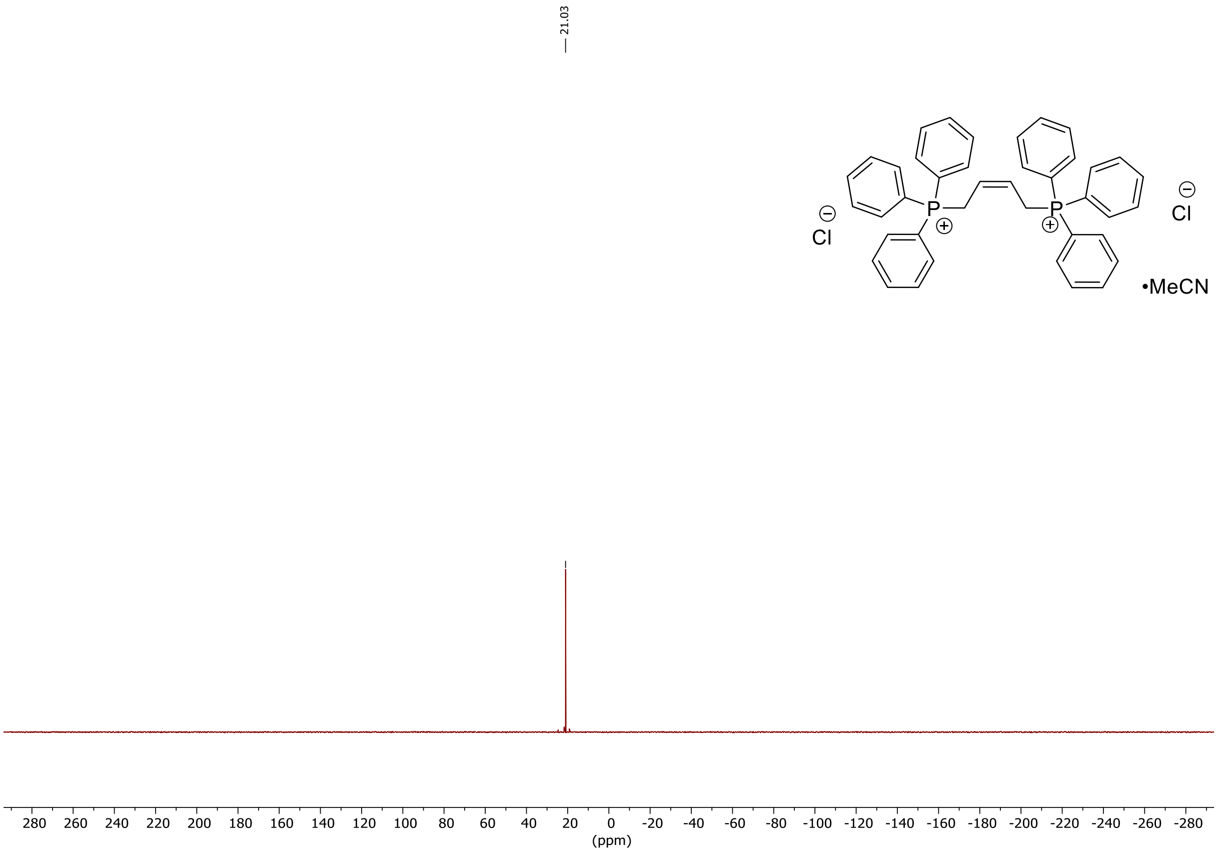

Fig. S160 ^{31}P NMR (203 MHz, chloroform-*d*, 298 K) spectrum of compound **4j**

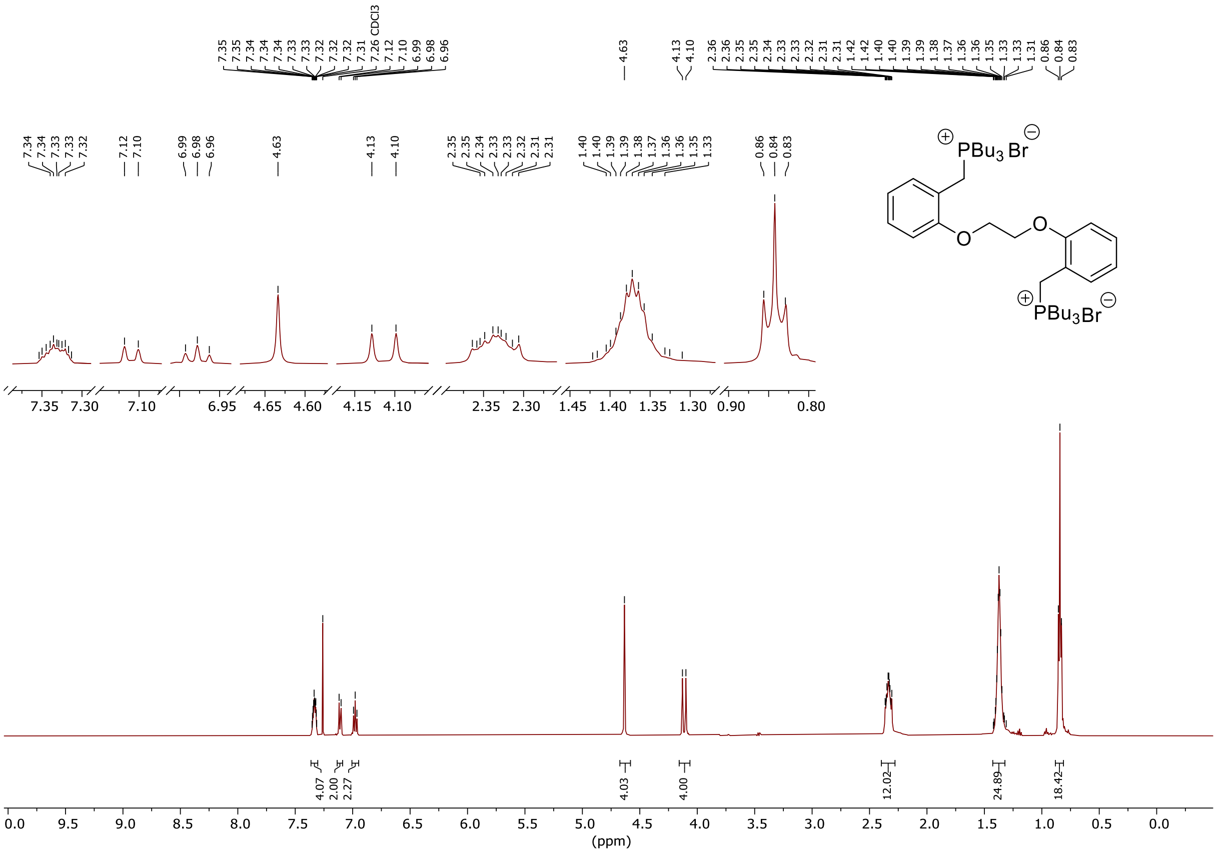

Fig. S161 ^{1}H NMR (501 MHz, chloroform-*d*, 298 K) spectrum of compound **4k**

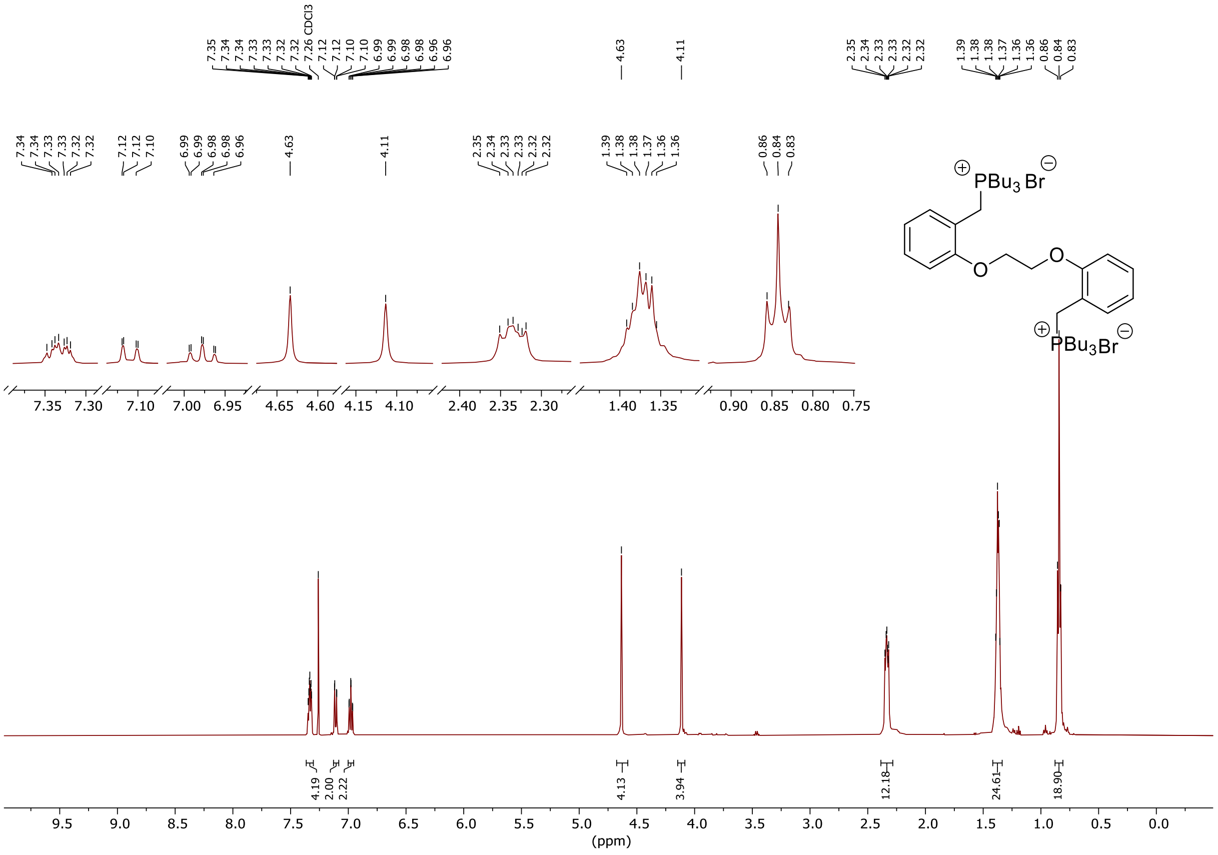

Fig. S162 ^{1}H{^{31}P} NMR (501 MHz, chloroform-*d*, 298 K) spectrum of compound **4k**

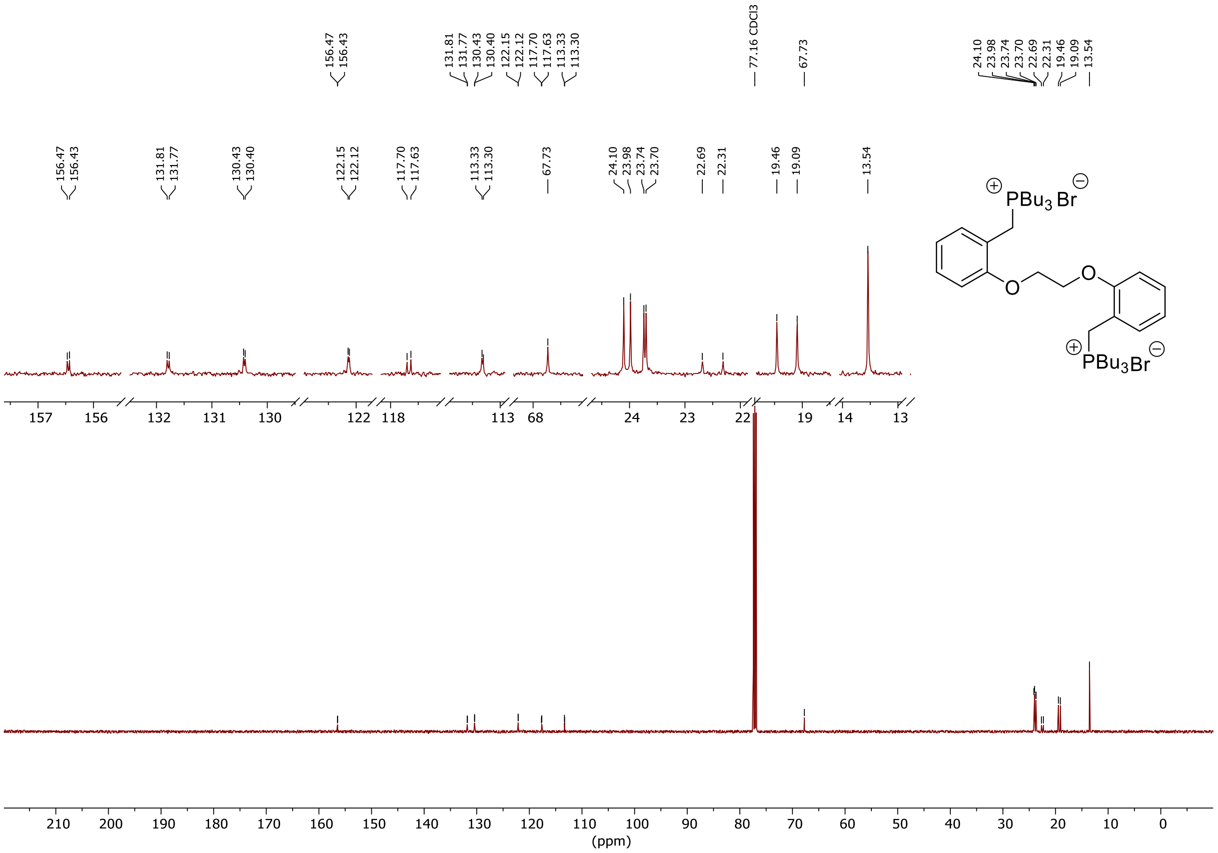

Fig. S163 $^{13}C\{^{1}H\}$ NMR (126 MHz, chloroform-*d*, 298 K) spectrum of compound **4k**

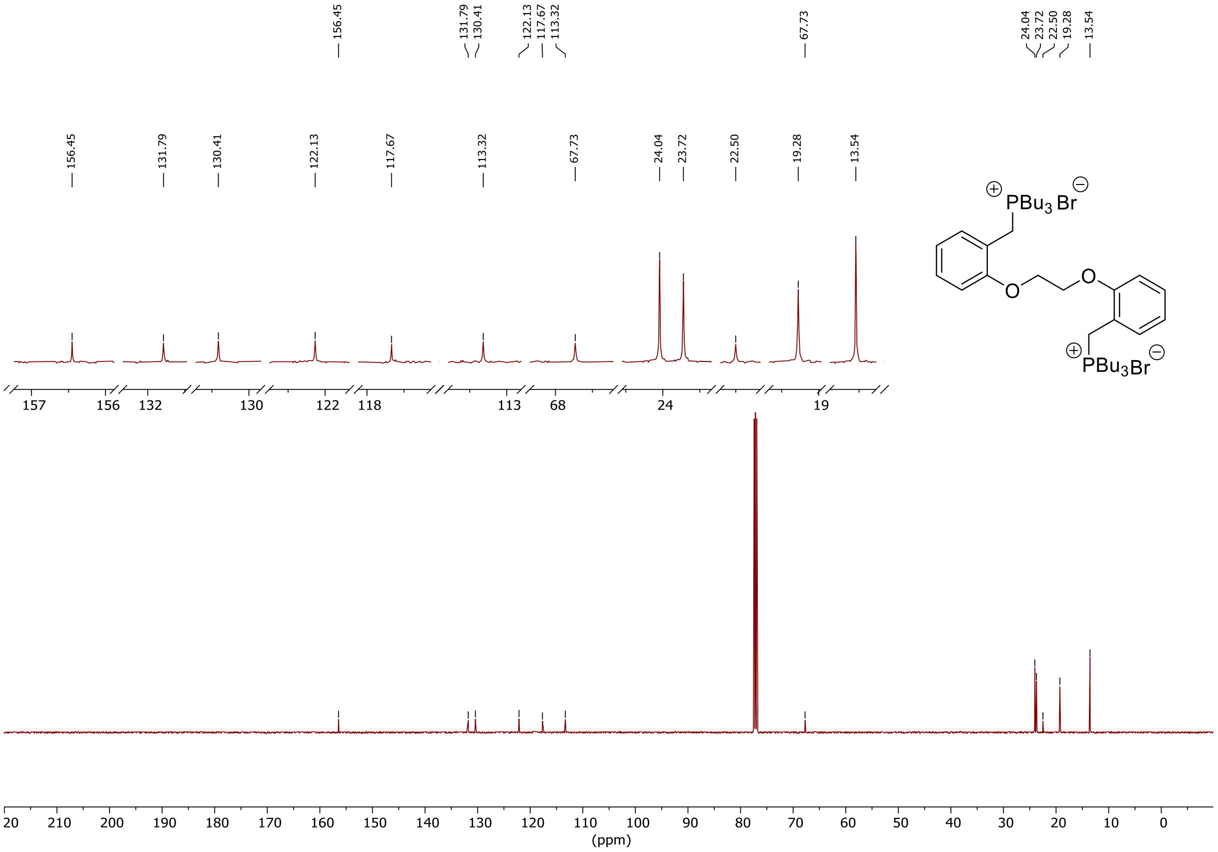

Fig. S164 $^{13}C\{^{1}H,^{31}P\}$(126 MHz, chloroform-*d*, 298 K) spectrum of compound **4k**

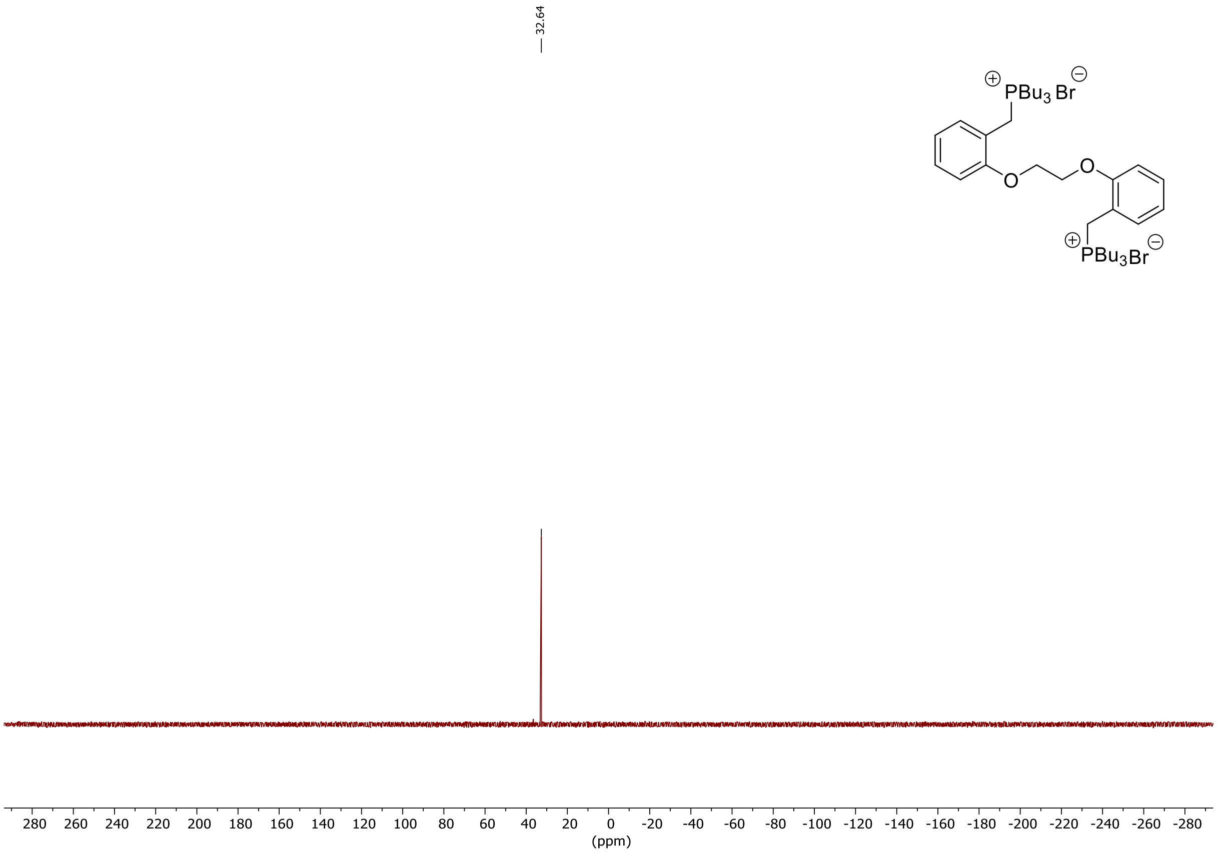

Fig. S165 ^{31}P NMR (203 MHz, chloroform-*d*, 298 K) spectrum of compound **4k**

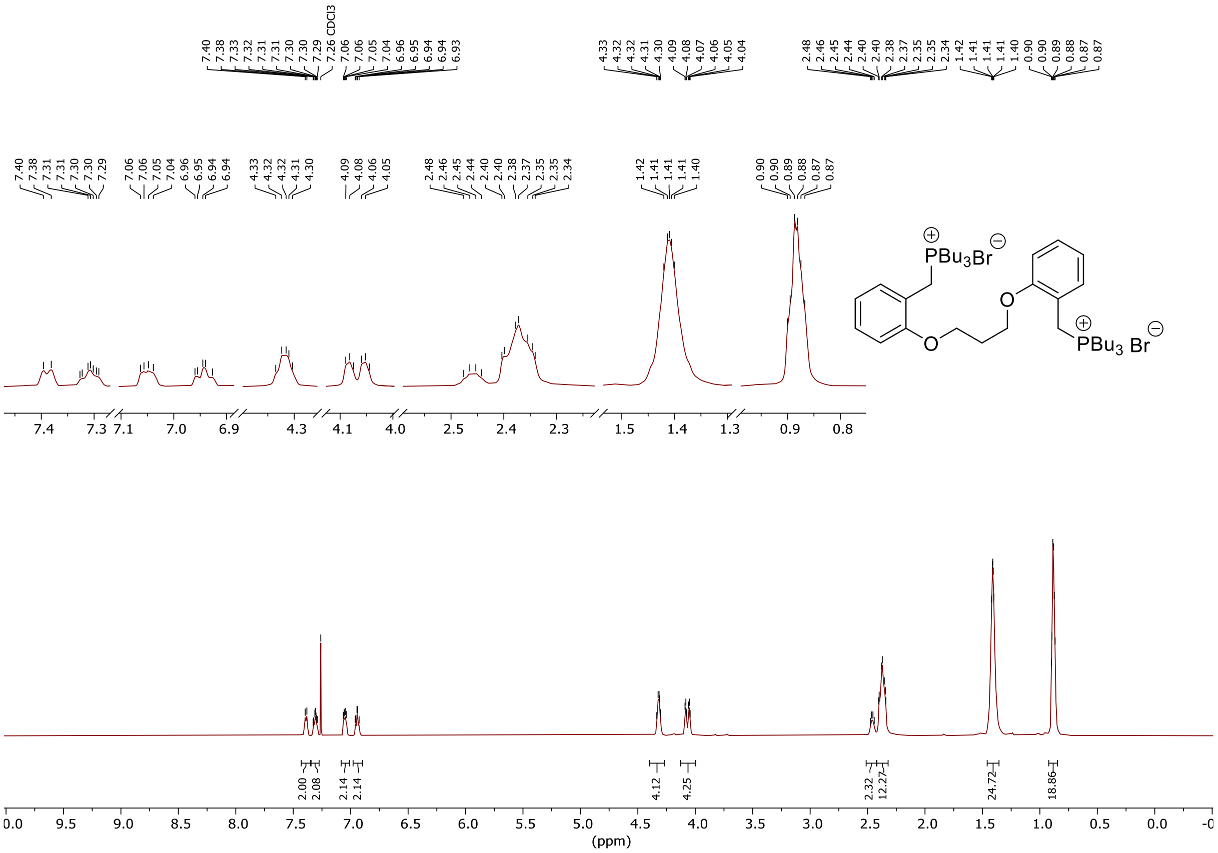

Fig. S166 ^{1}H NMR (501 MHz, chloroform-*d*, 298 K) spectrum of compound **4l**

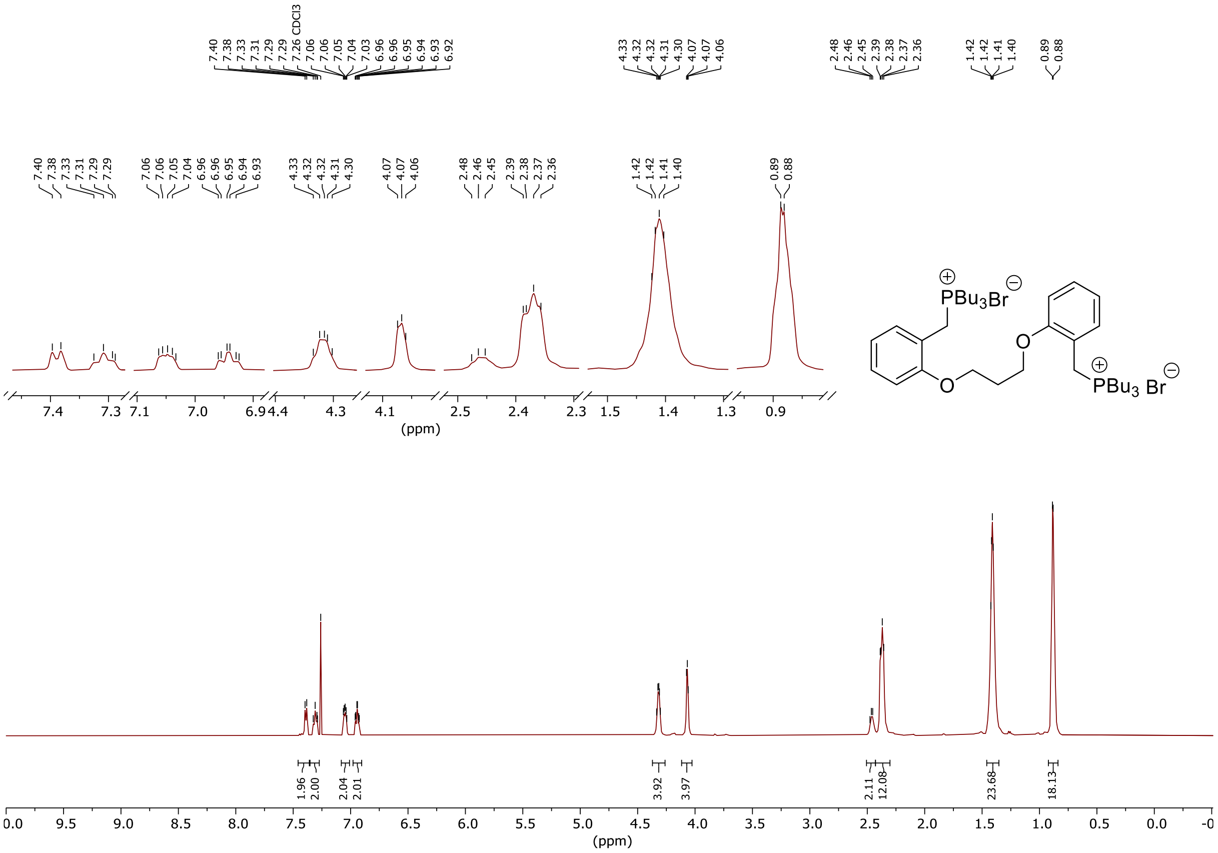

Fig. S167 $^{1}H\{^{31}P\}$ NMR (501 MHz, chloroform-*d*, 298 K) spectrum of compound **4l**

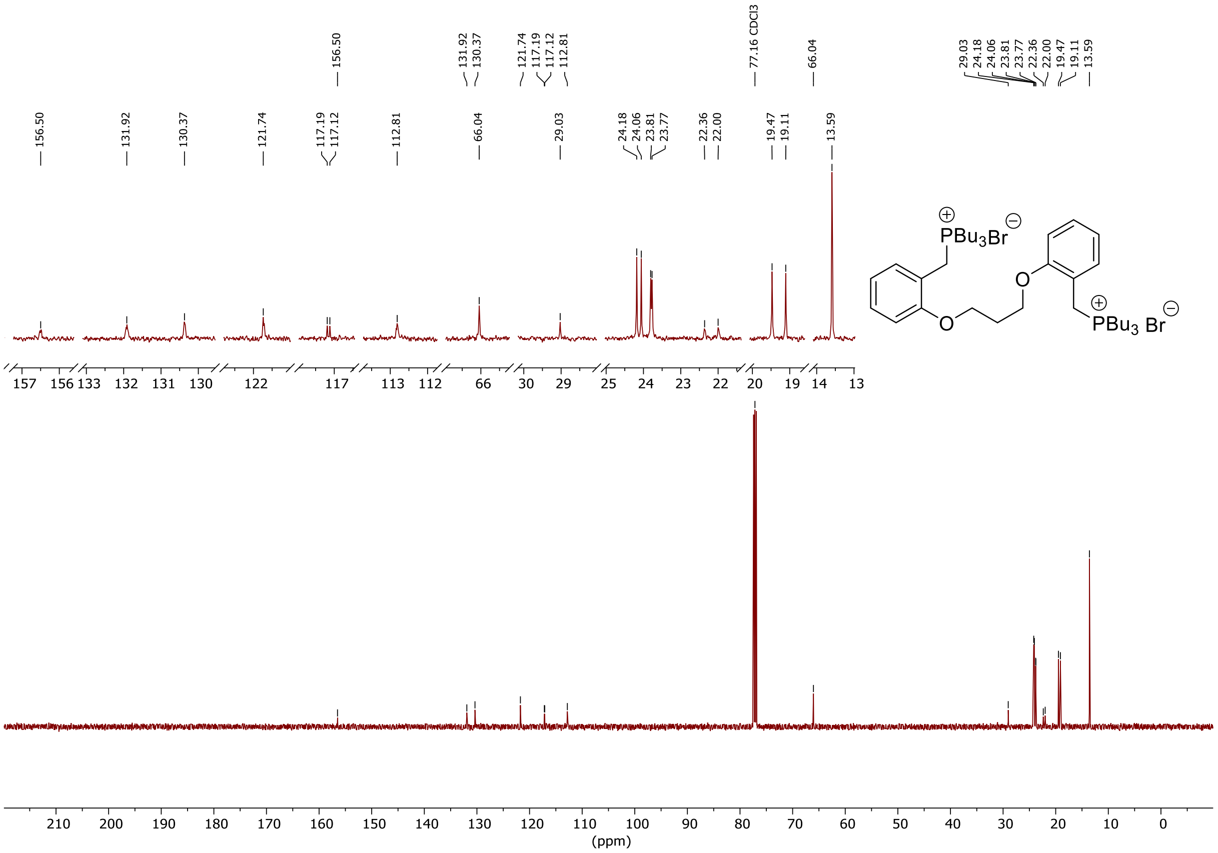

Fig. S168 $^{13}C\{^{1}H\}$ NMR (126 MHz, chloroform-*d*, 298 K) spectrum of compound **4l**

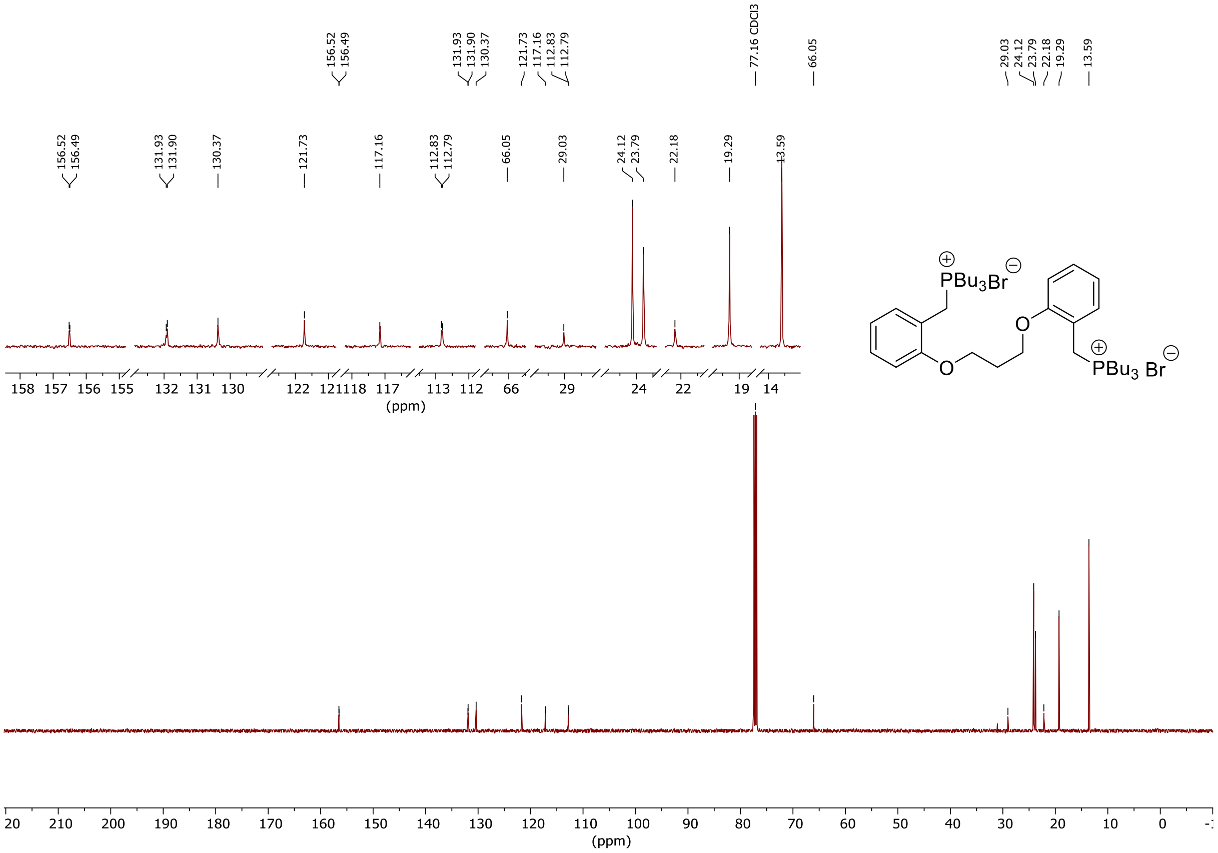

Fig. S169 $^{13}C\{^{1}H,^{31}P\}$(126 MHz, chloroform-*d*, 298 K) spectrum of compound **4l**

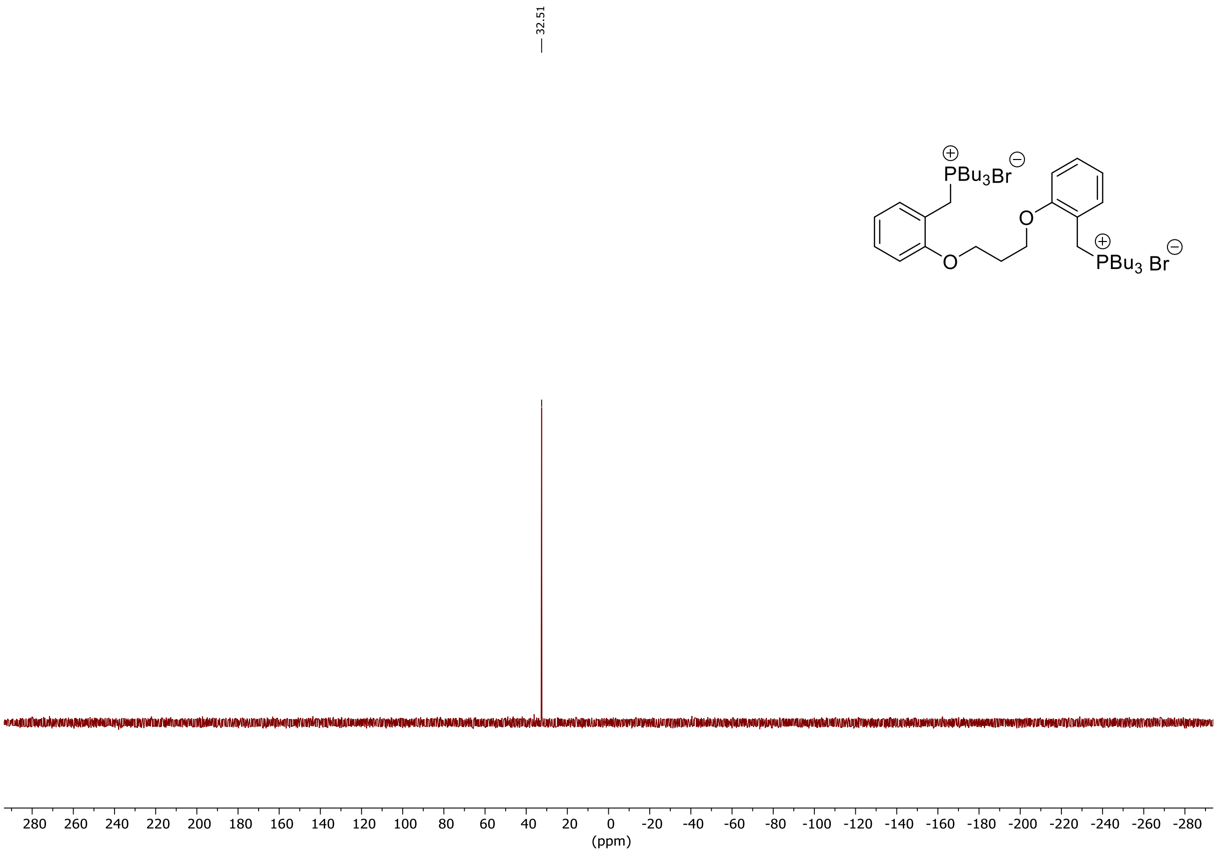

Fig. S170 ^{31}P NMR (203 MHz, chloroform-*d*, 298 K) spectrum of compound **4l**

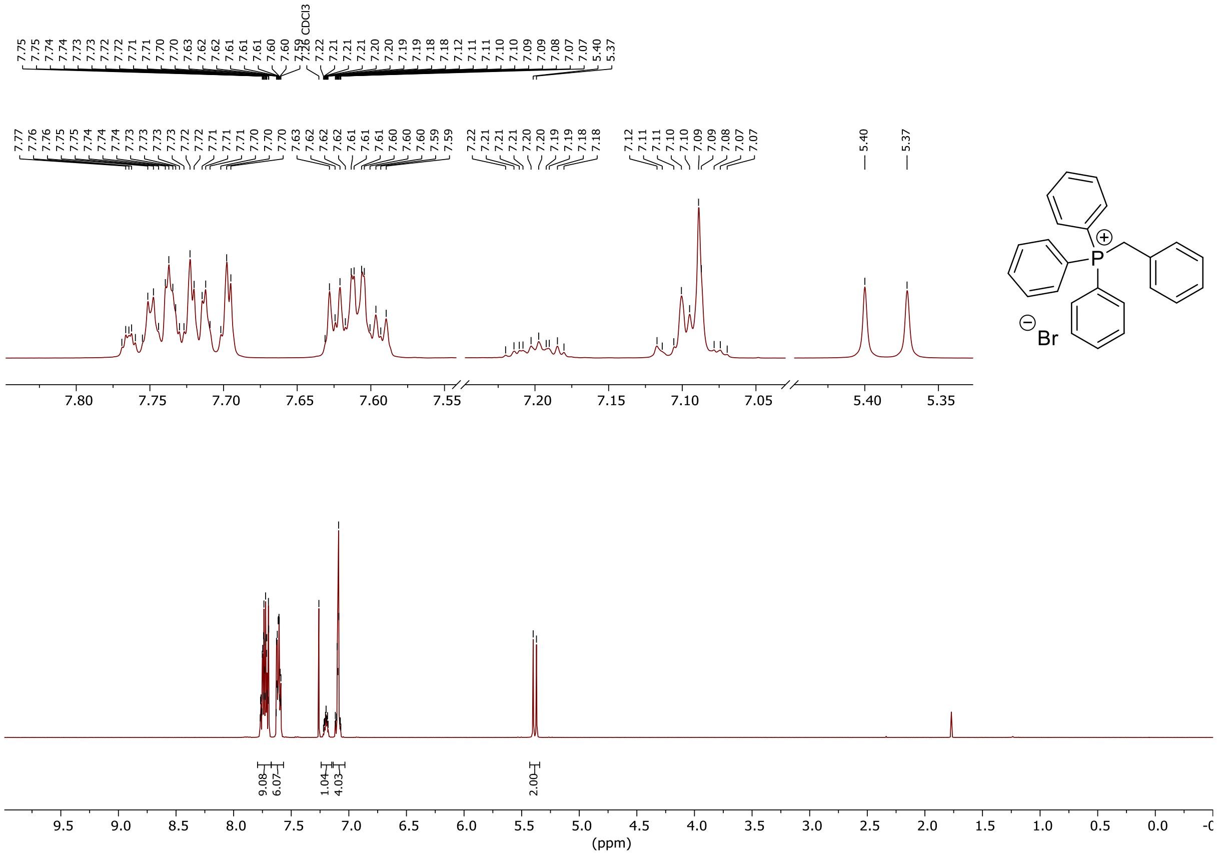

Fig. S171 ^{1}H NMR (501 MHz, chloroform-*d*, 298 K) spectrum of compound **7b**

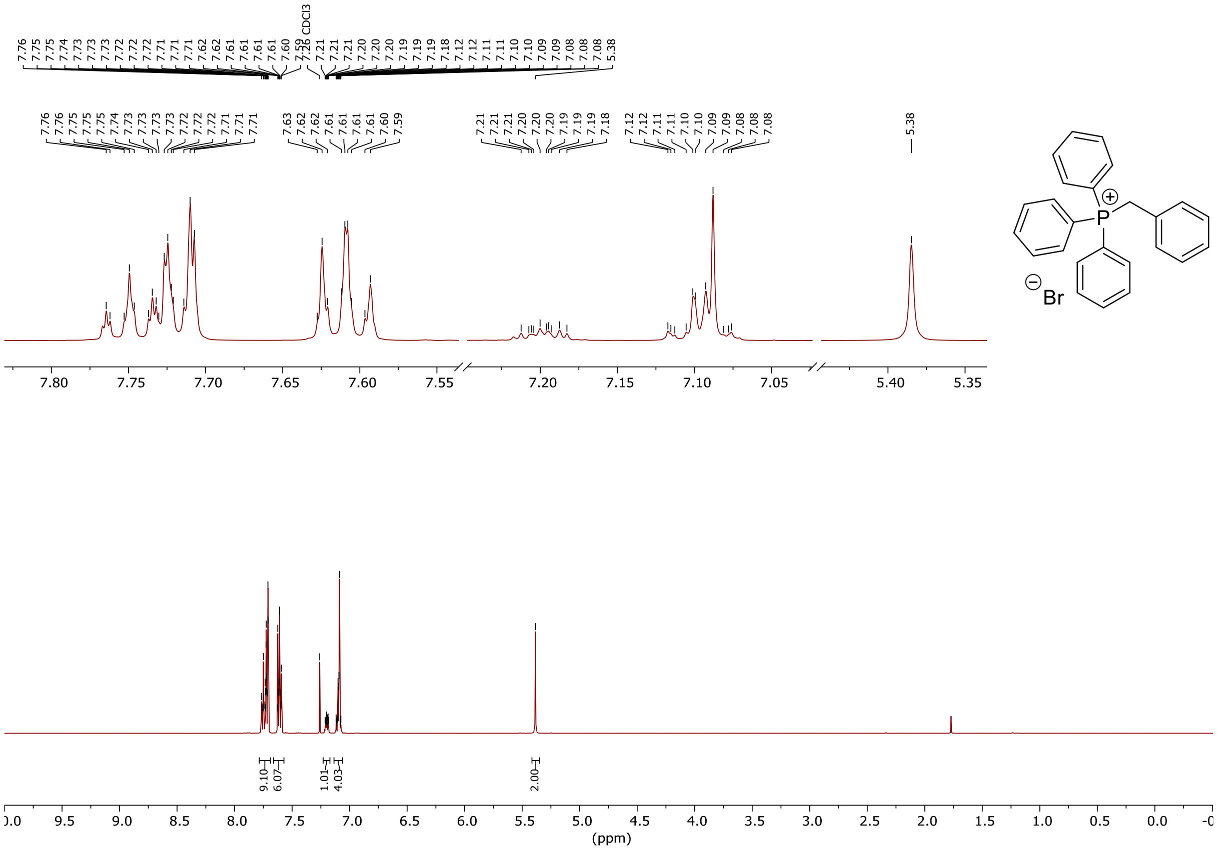

Fig. S172 ^{1}H{^{31}P} NMR (501 MHz, chloroform-*d*, 298 K) spectrum of compound **7b**

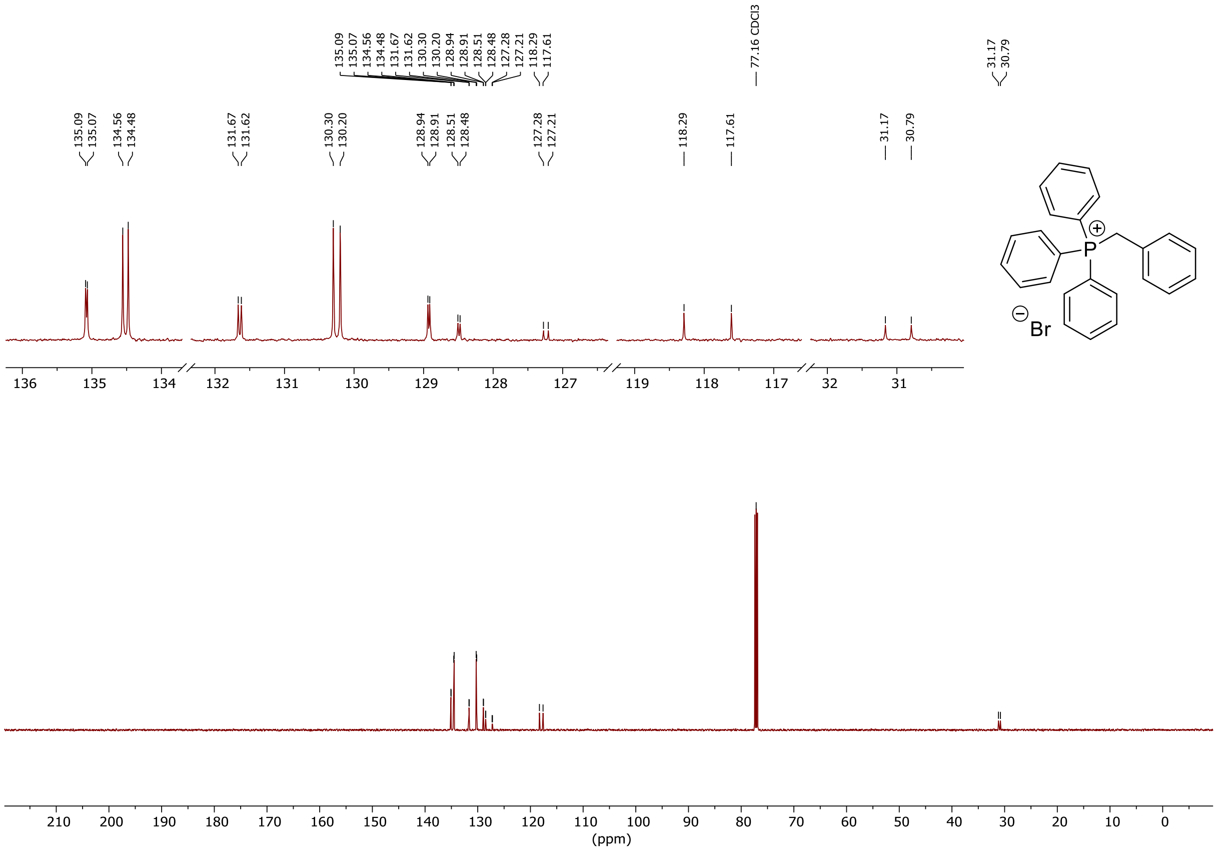

Fig. S173 $^{13}C\{^{1}H\}$ NMR (126 MHz, chloroform-*d*, 298 K) spectrum of compound **7b**

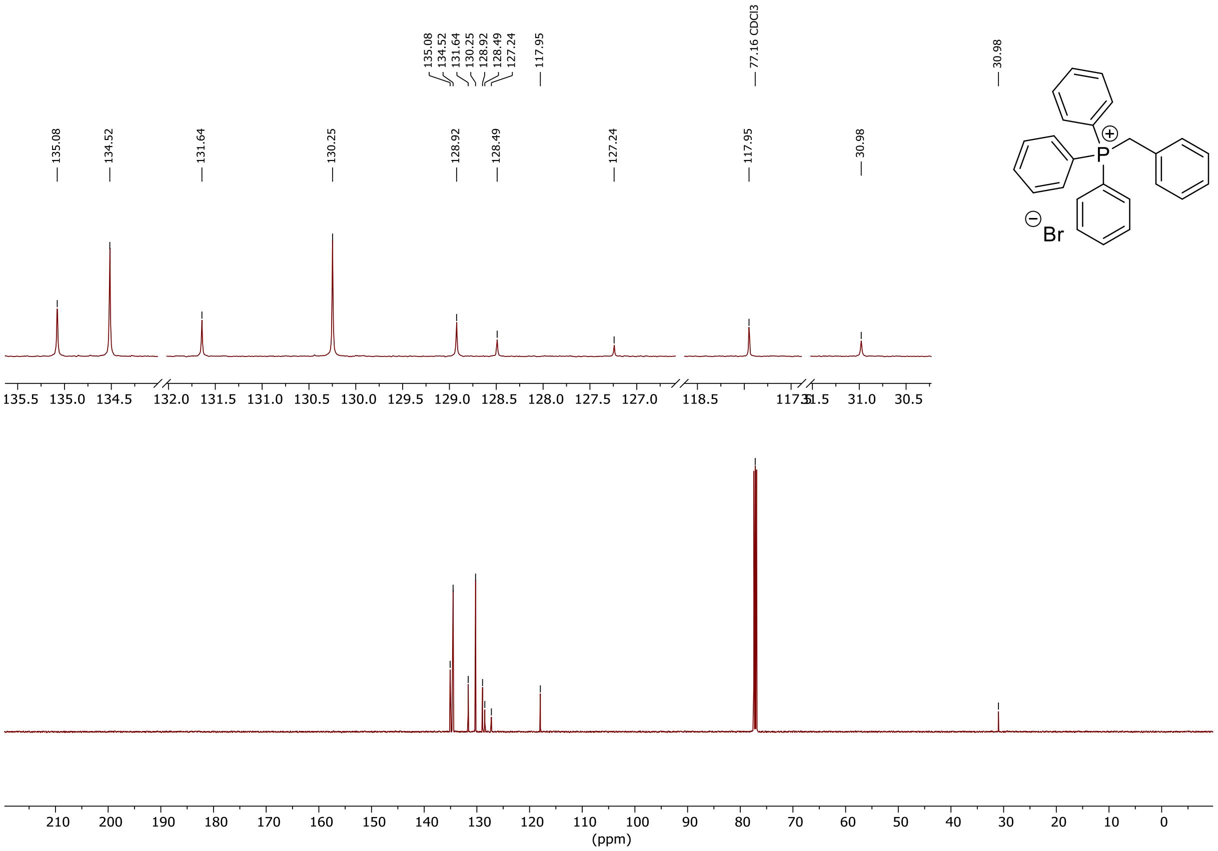

Fig. S174 $^{13}C\{^{1}H,^{31}P\}$ NMR (126 MHz, chloroform-*d*, 298 K) spectrum of compound **7b**

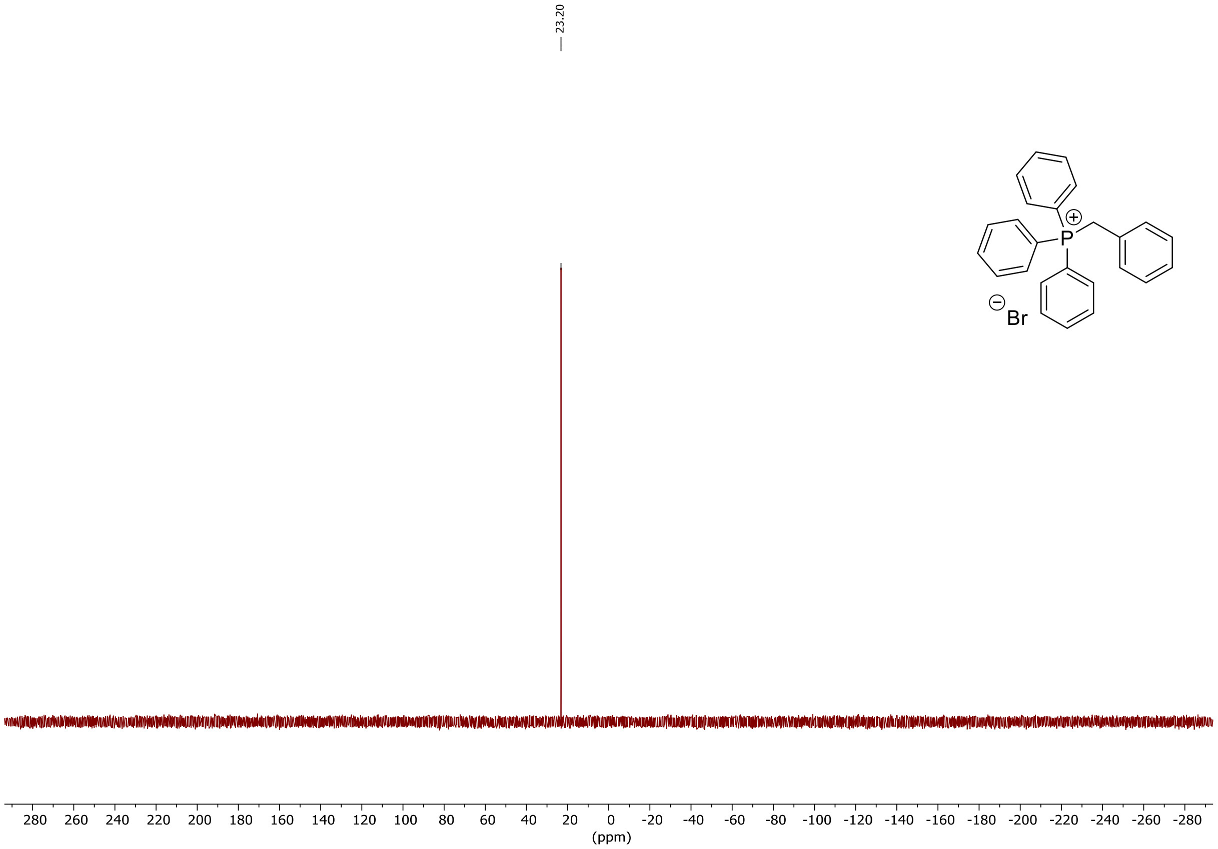

Fig. S175 ^{31}P NMR (203 MHz, chloroform-*d*, 298 K) spectrum of compound **7b**

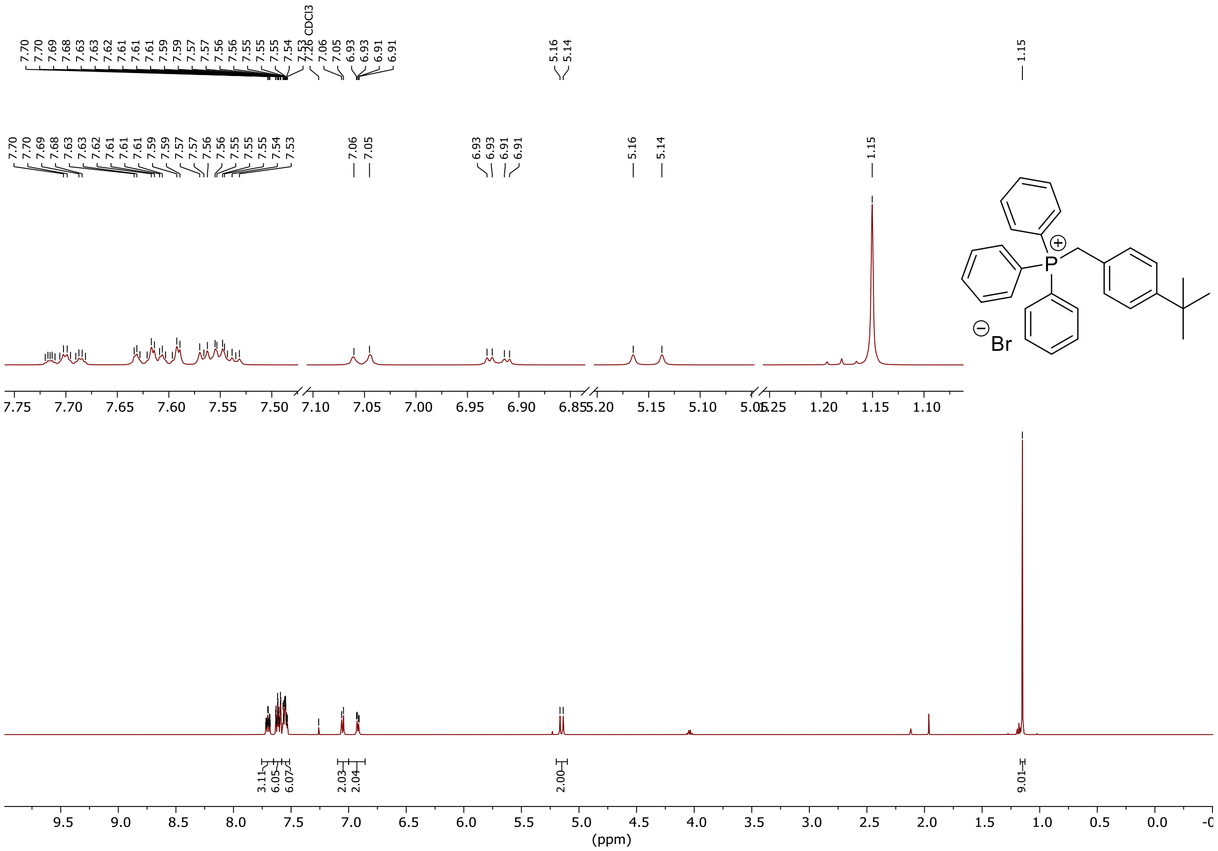

Fig. S176 ^{1}H NMR (501 MHz, chloroform-*d*, 298 K) spectrum of compound **7c**

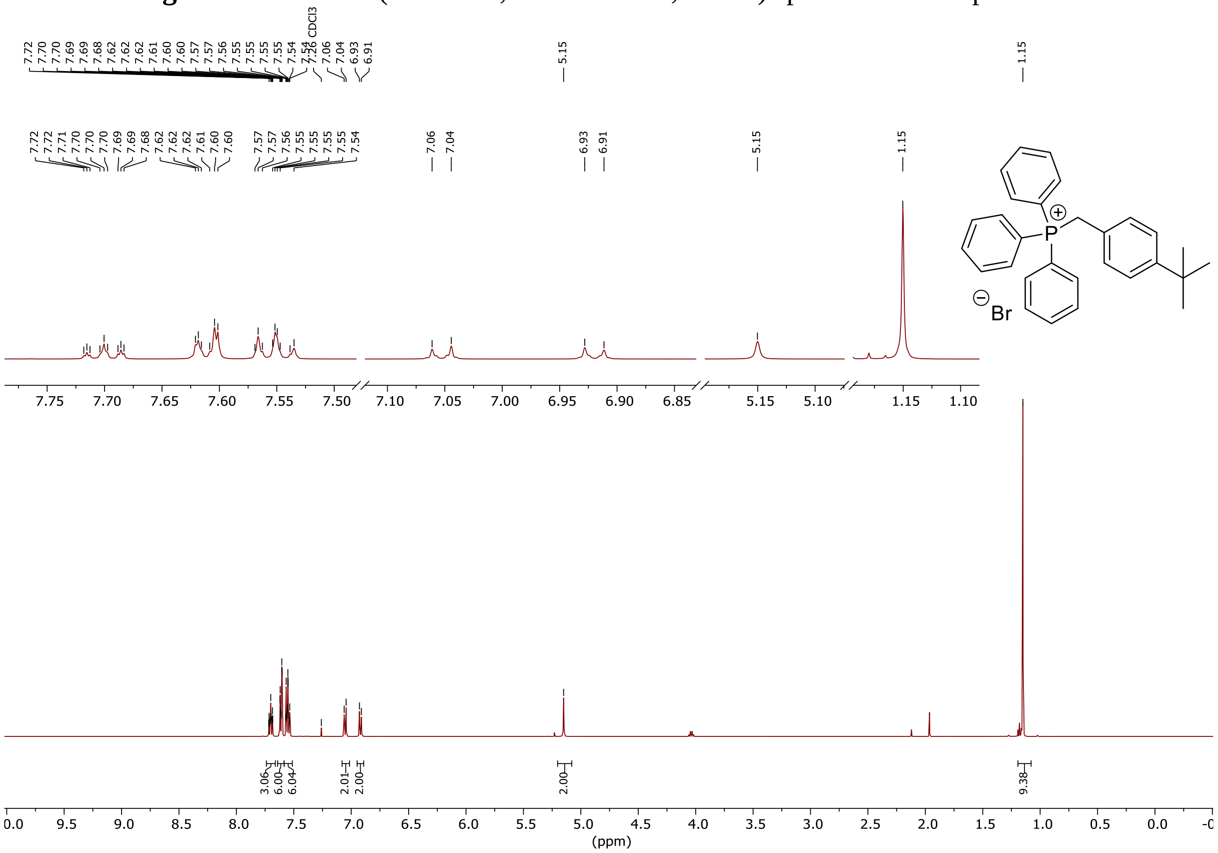

Fig. S177 $^{1}H\{^{31}P\}$ NMR (501 MHz, chloroform-*d*, 298 K) spectrum of compound **7c**

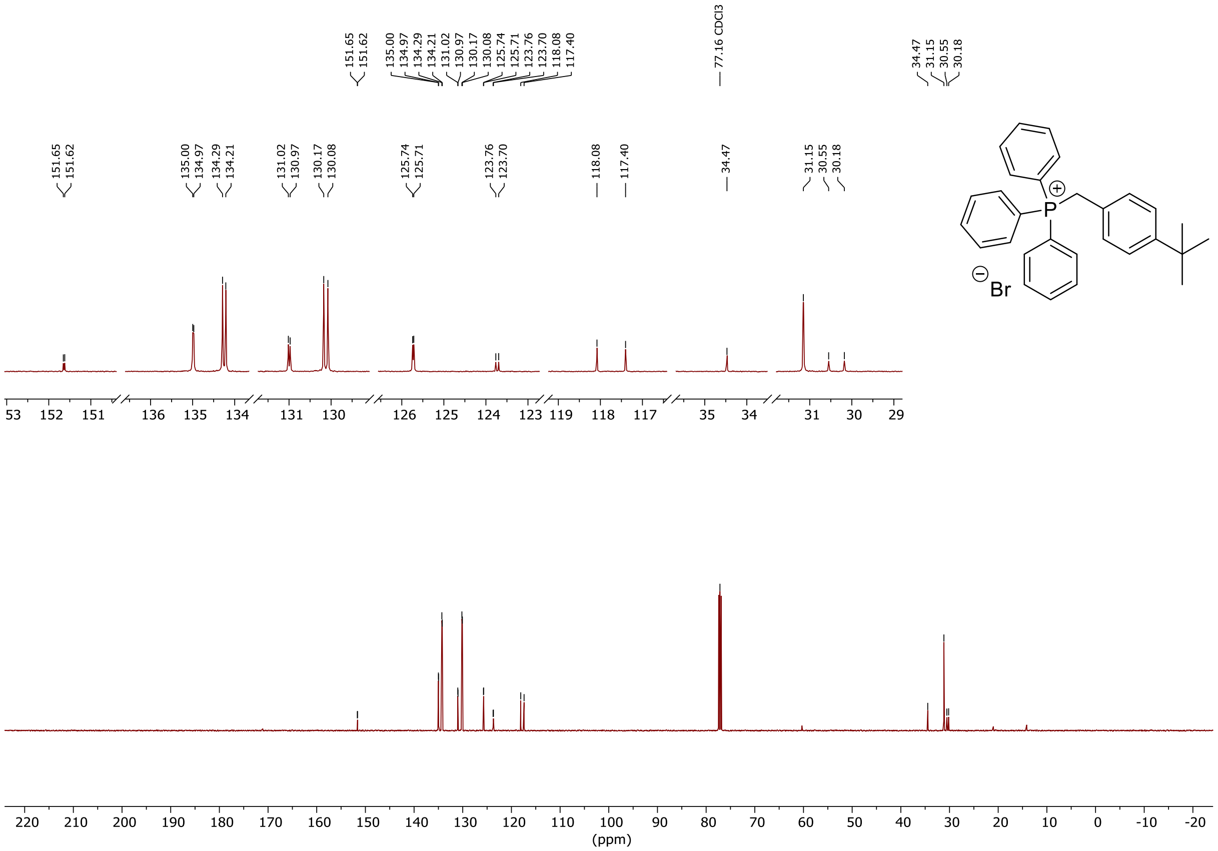

Fig. S178 $^{13}C\{^1H\}$ NMR (126 MHz, chloroform-*d*, 298 K) spectrum of compound **7c**

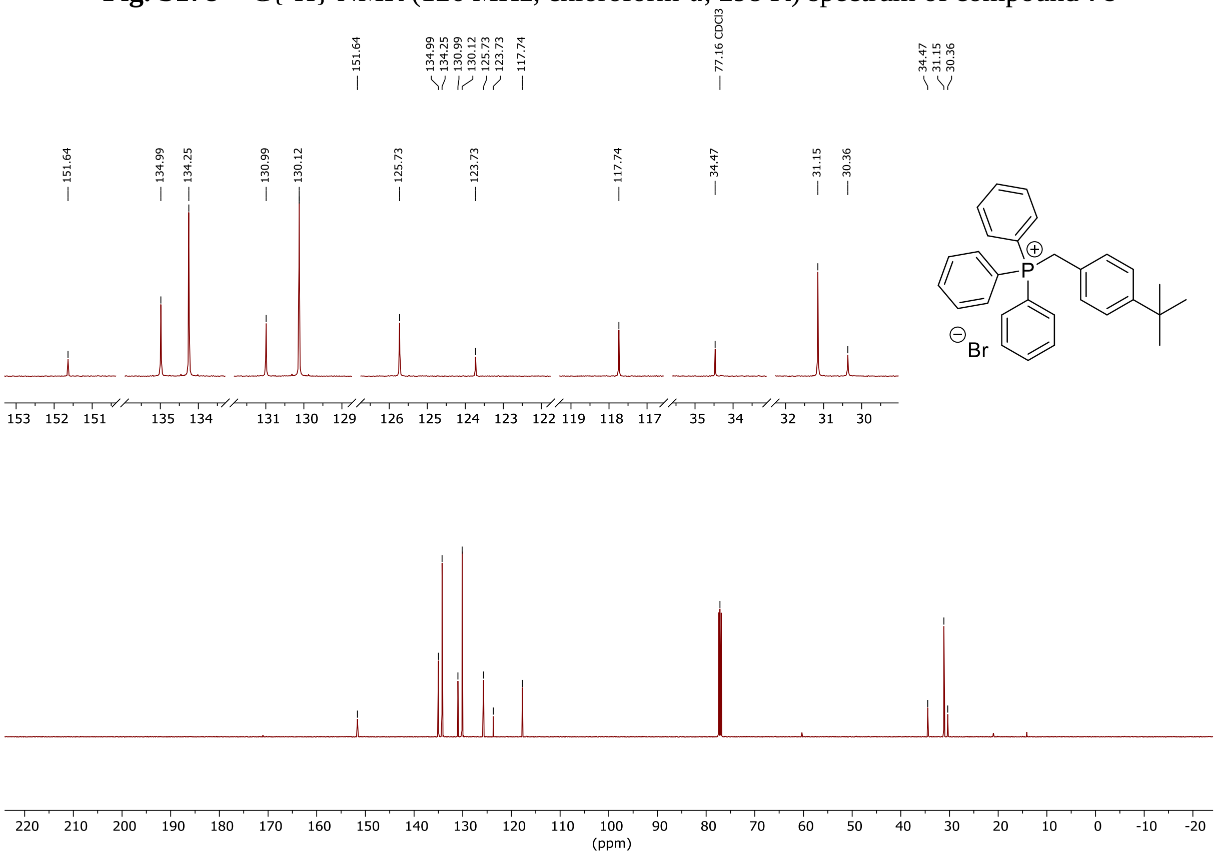

Fig. S179 $^{13}C\{^1H,^{31}P\}$ NMR (126 MHz, chloroform-*d*, 298 K) spectrum of compound **7c**

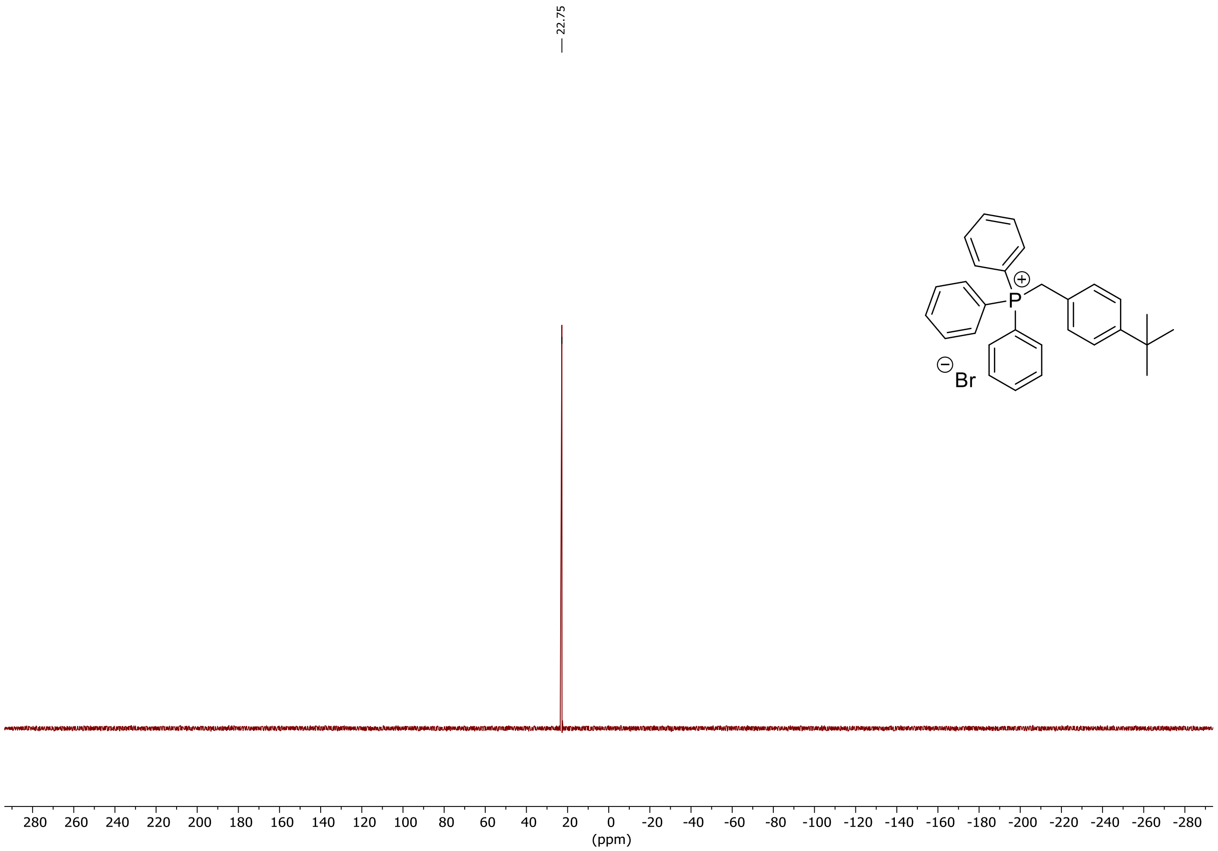

Fig. S180 ^{31}P NMR (203 MHz, chloroform-*d*, 298 K) spectrum of compound **7c**

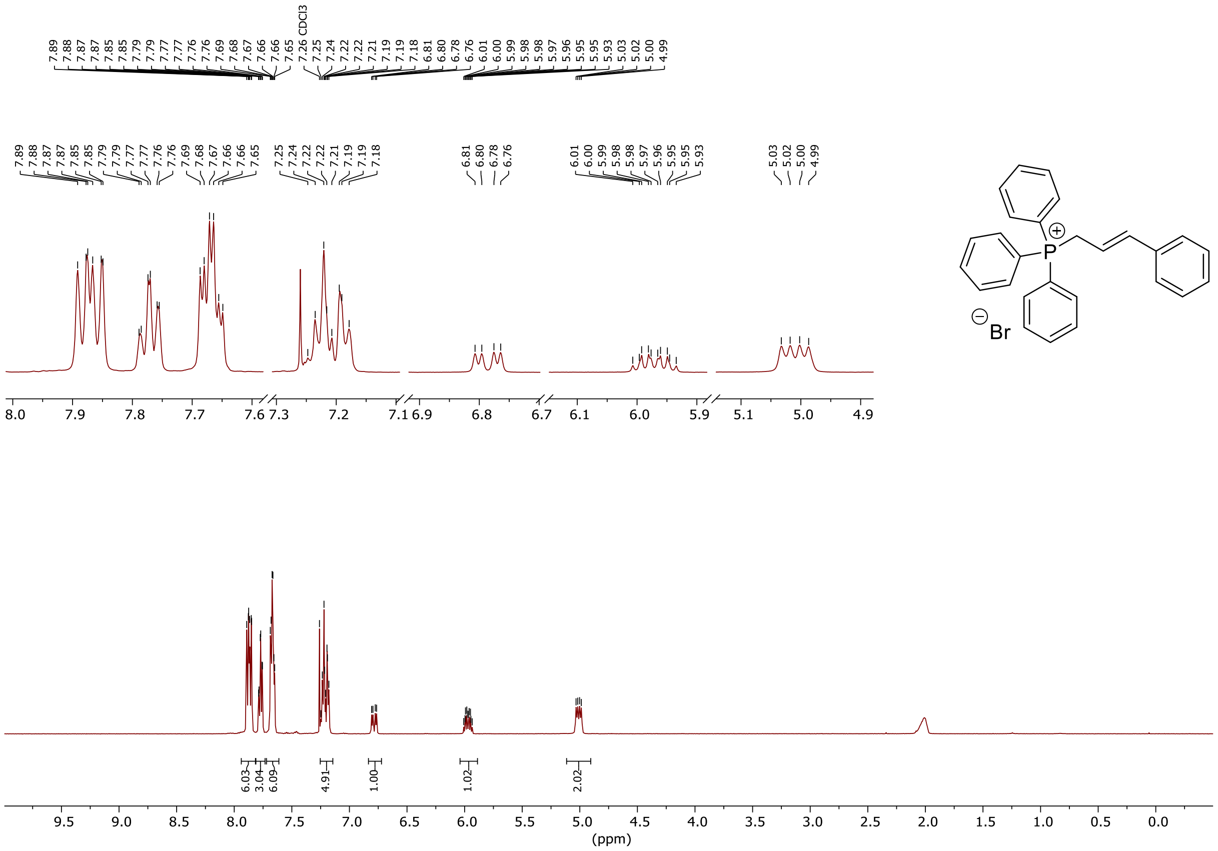

Fig. S181 ^{1}H NMR (501 MHz, chloroform-*d*, 298 K) spectrum of compound **7d**

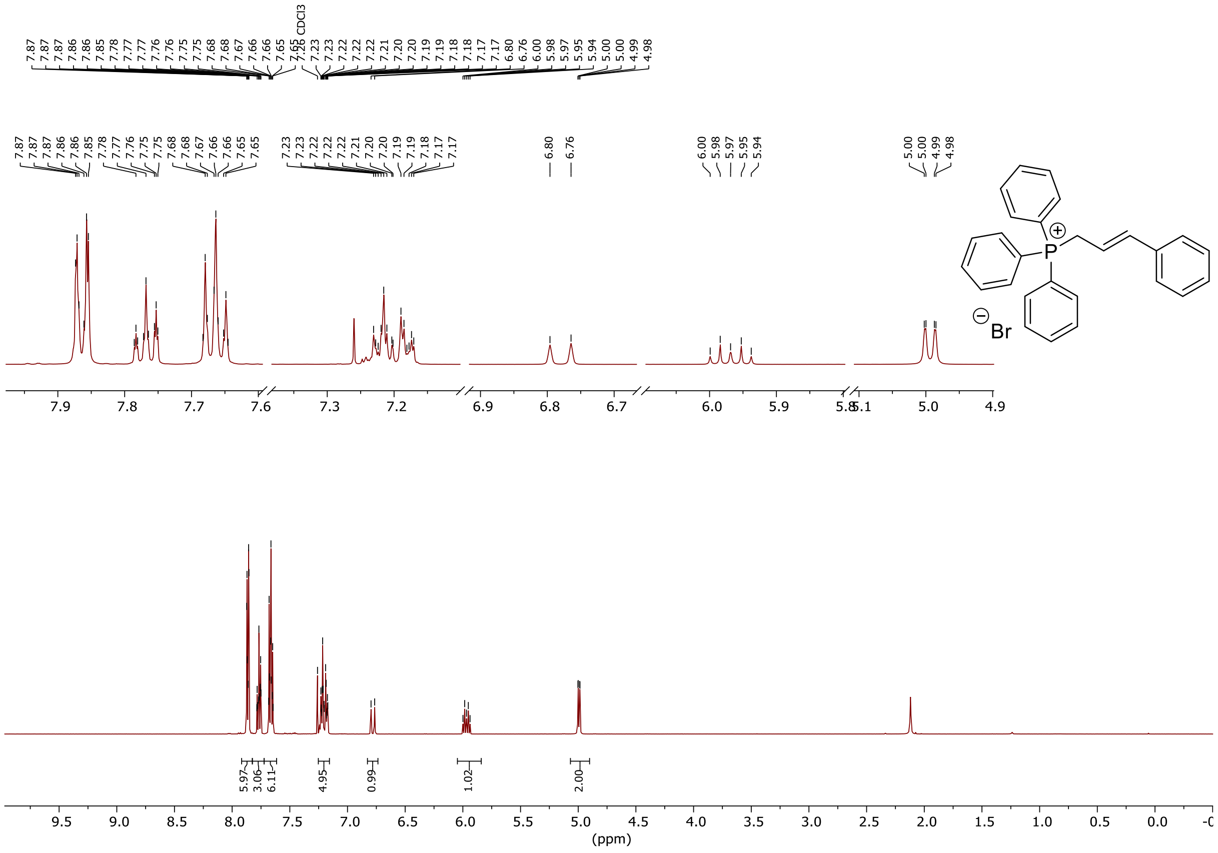

Fig. S182 ^{1}H{^{31}P} NMR (501 MHz, chloroform-*d*, 298 K) spectrum of compound **7d**

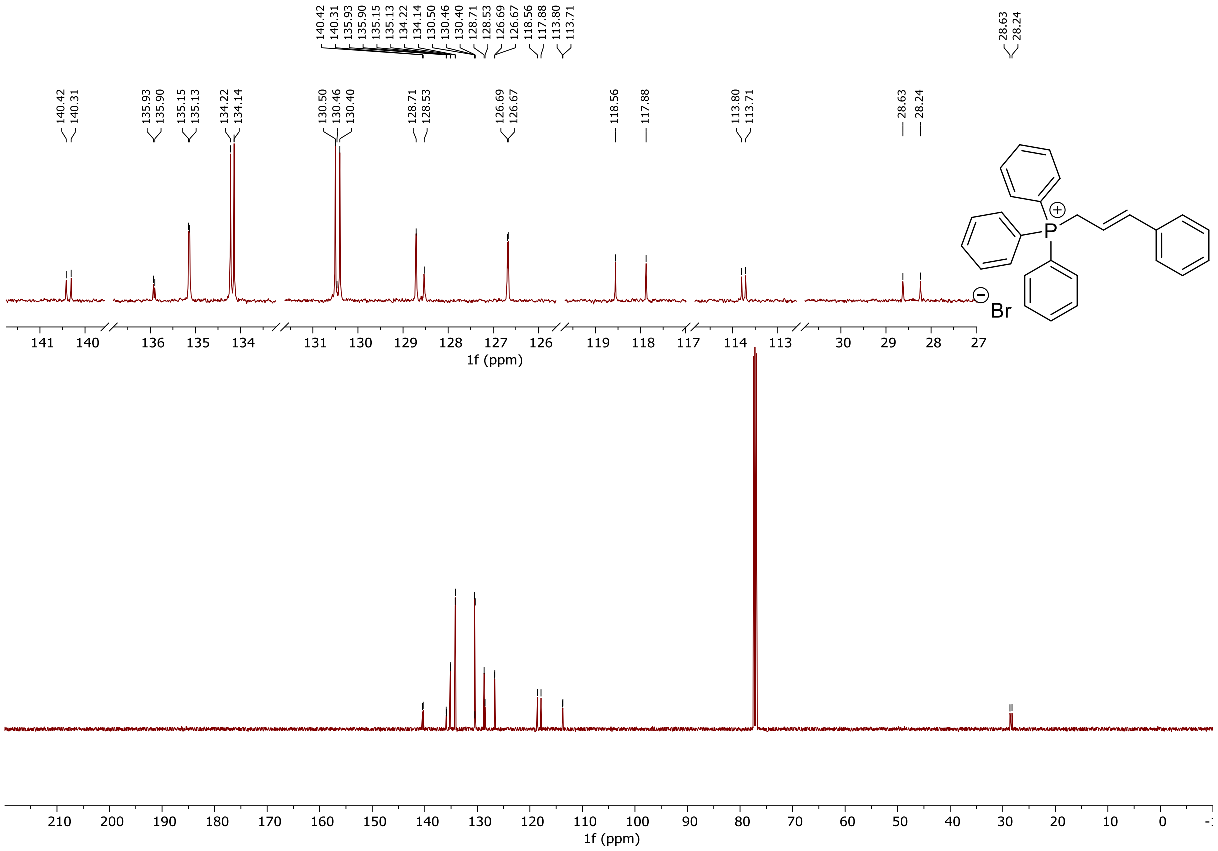

Fig. S183 $^{13}C\{^{1}H\}$ NMR (126 MHz, chloroform-*d*, 298 K) spectrum of compound **7d**

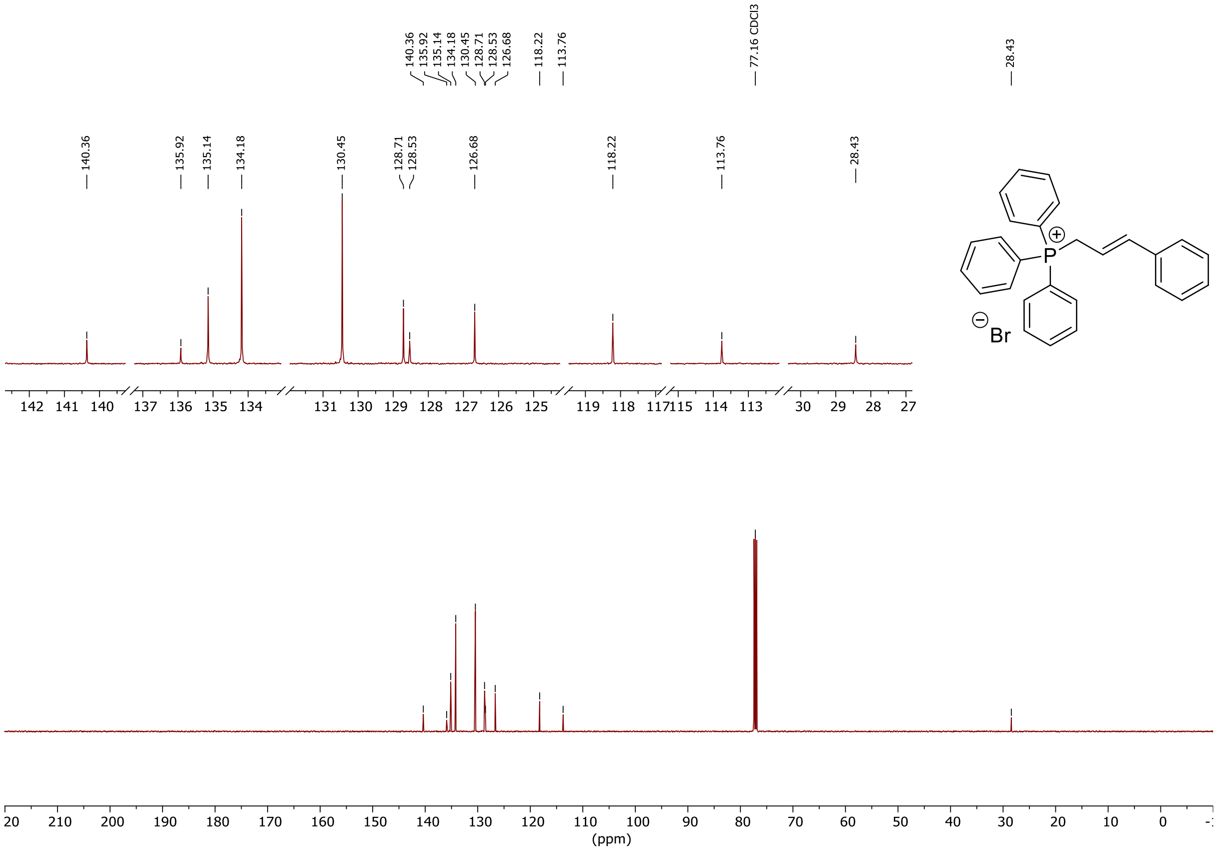

Fig. S184 $^{13}C\{^{1}H,^{31}P\}$ NMR (126 MHz, chloroform-*d*, 298 K) spectrum of compound **7d**

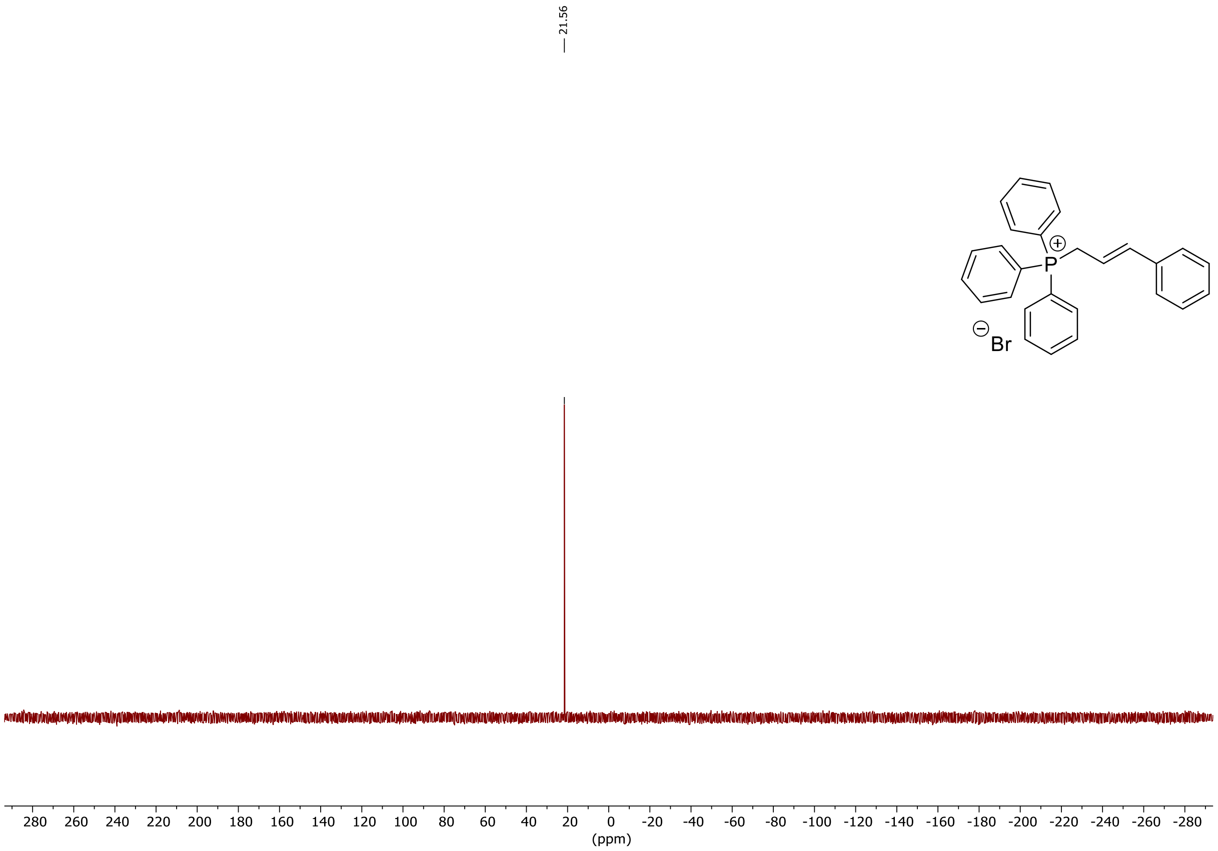

Fig. S185 ^{31}P NMR (203 MHz, chloroform-*d*, 298 K) spectrum of compound **7d**

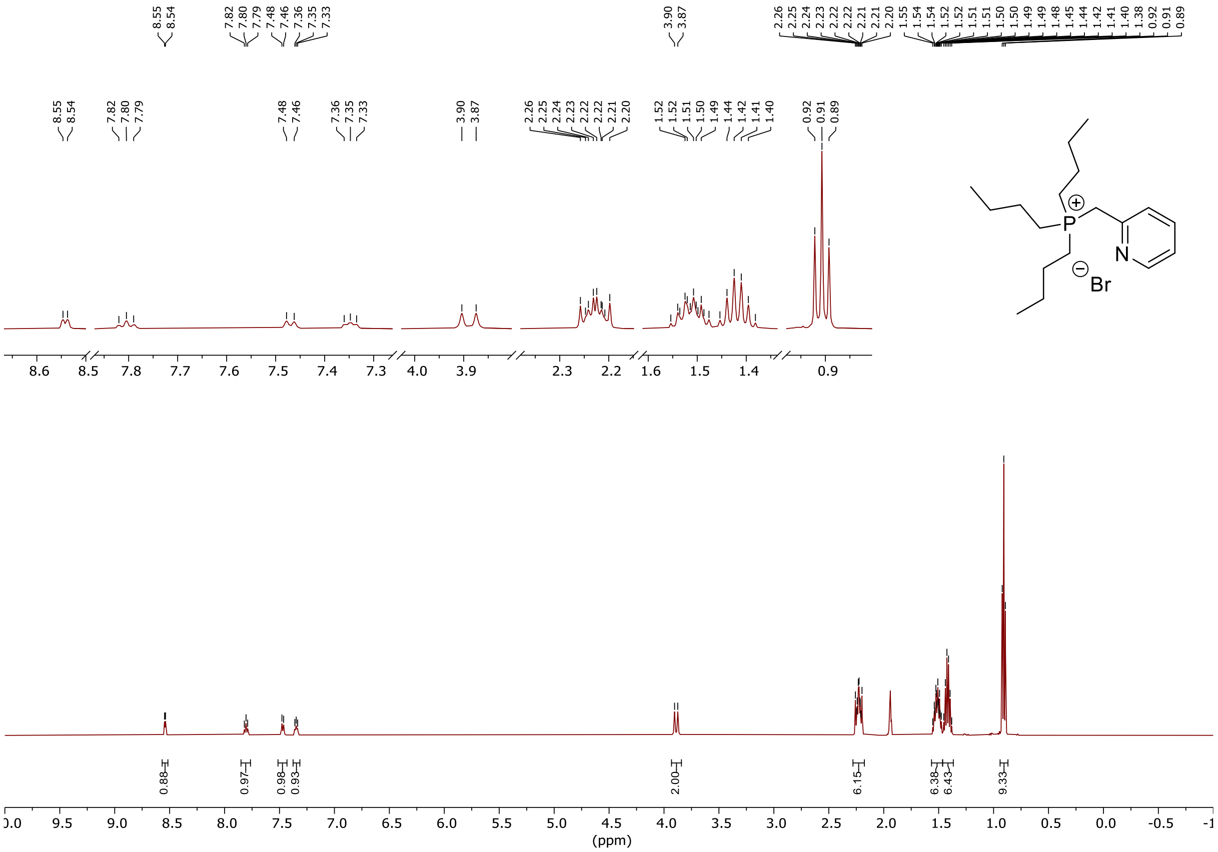

Fig. S186 ^{1}H NMR (501 MHz, acetonitrile-d_3, 298 K) spectrum of compound **2a**

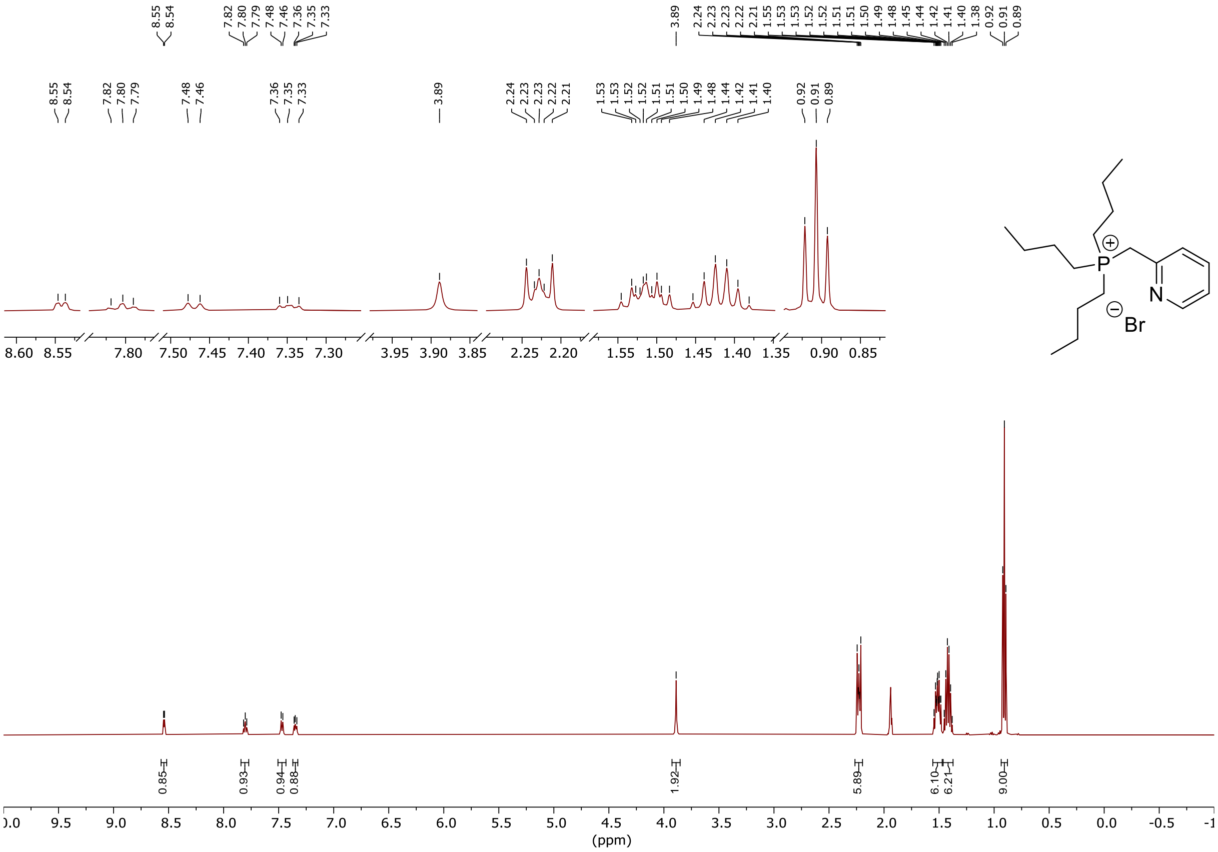

Fig. S187 ^{1}H{^{31}P} NMR (501 MHz, acetonitrile-d_3, 298 K) spectrum of compound **2a**

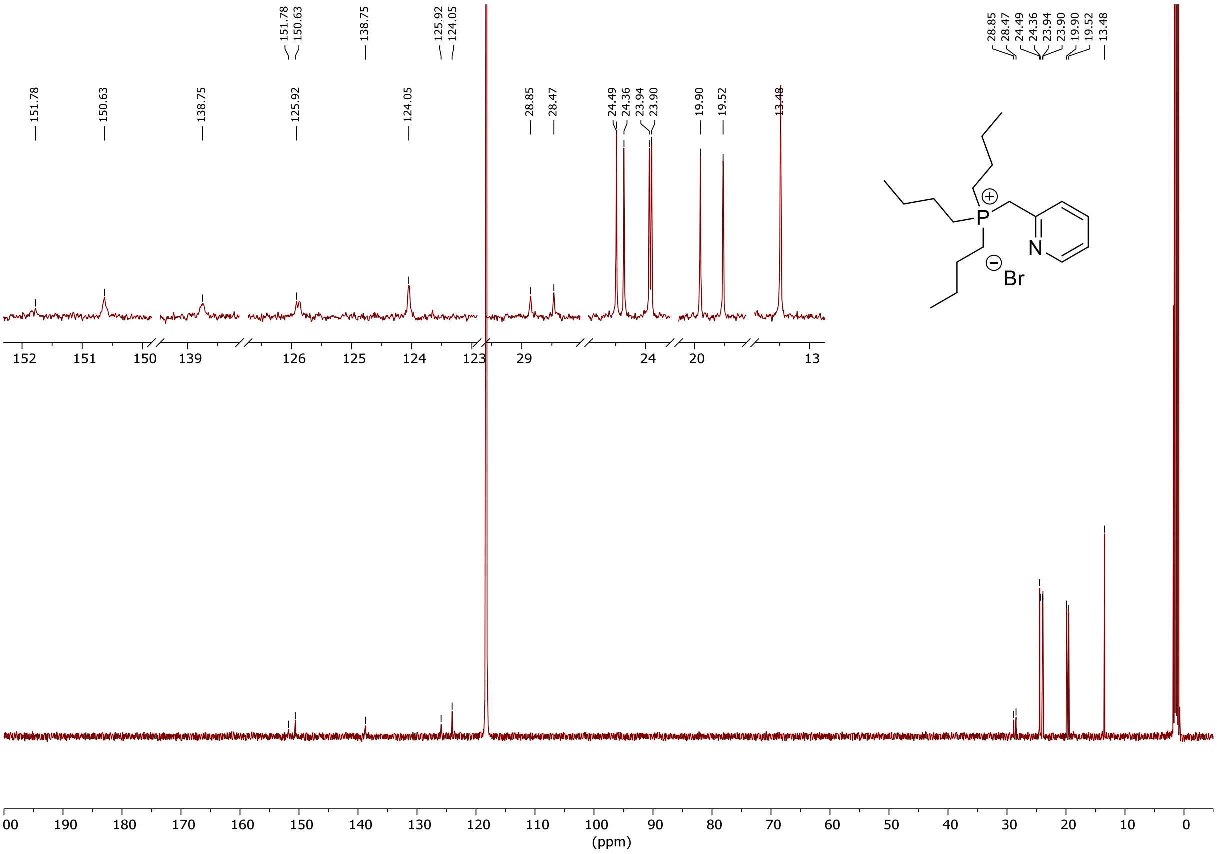

Fig. S188 $^{13}C\{^1H\}$ NMR (126 MHz, acetonitrile-d_3, 298 K) spectrum of compound **2a**

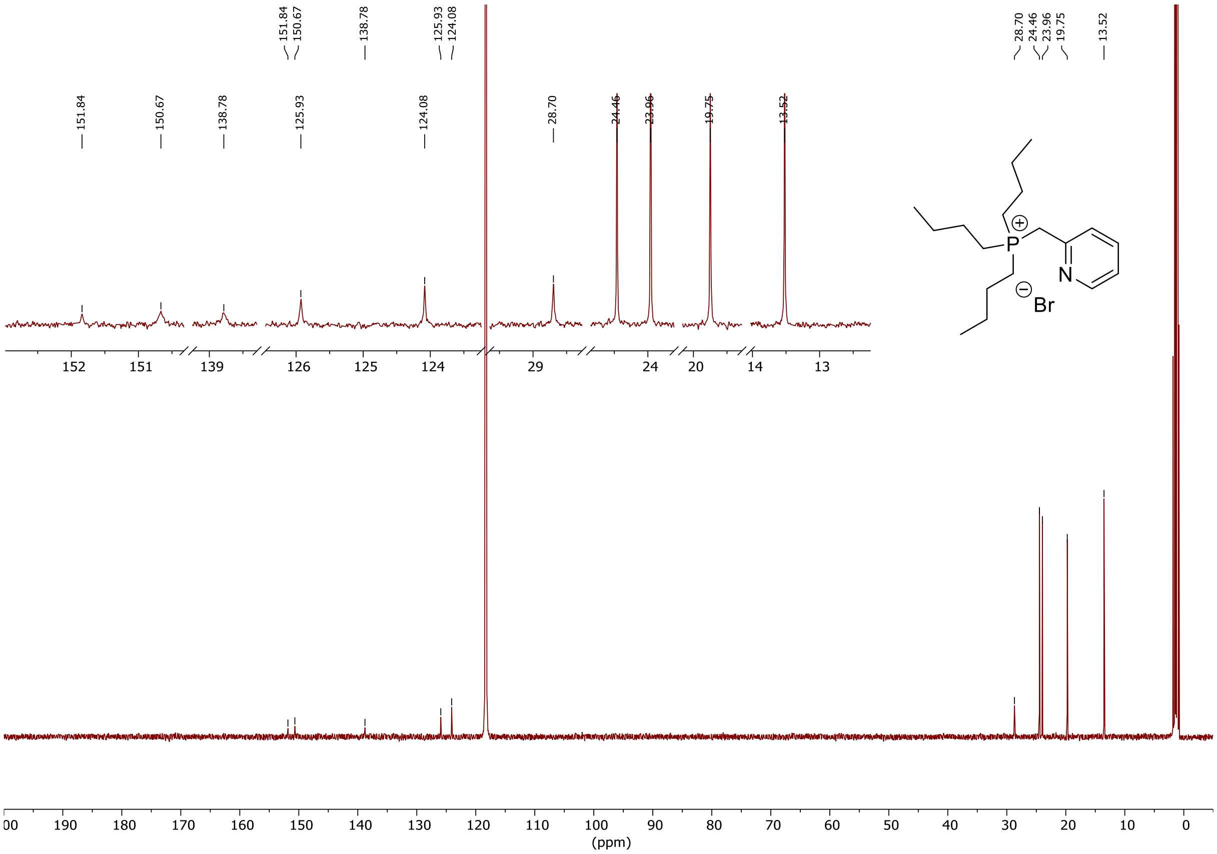

Fig. S189 $^{13}C\{^1H, ^{31}P\}$ NMR (126 MHz, acetonitrile-d_3, 298 K) spectrum of compound **2a**

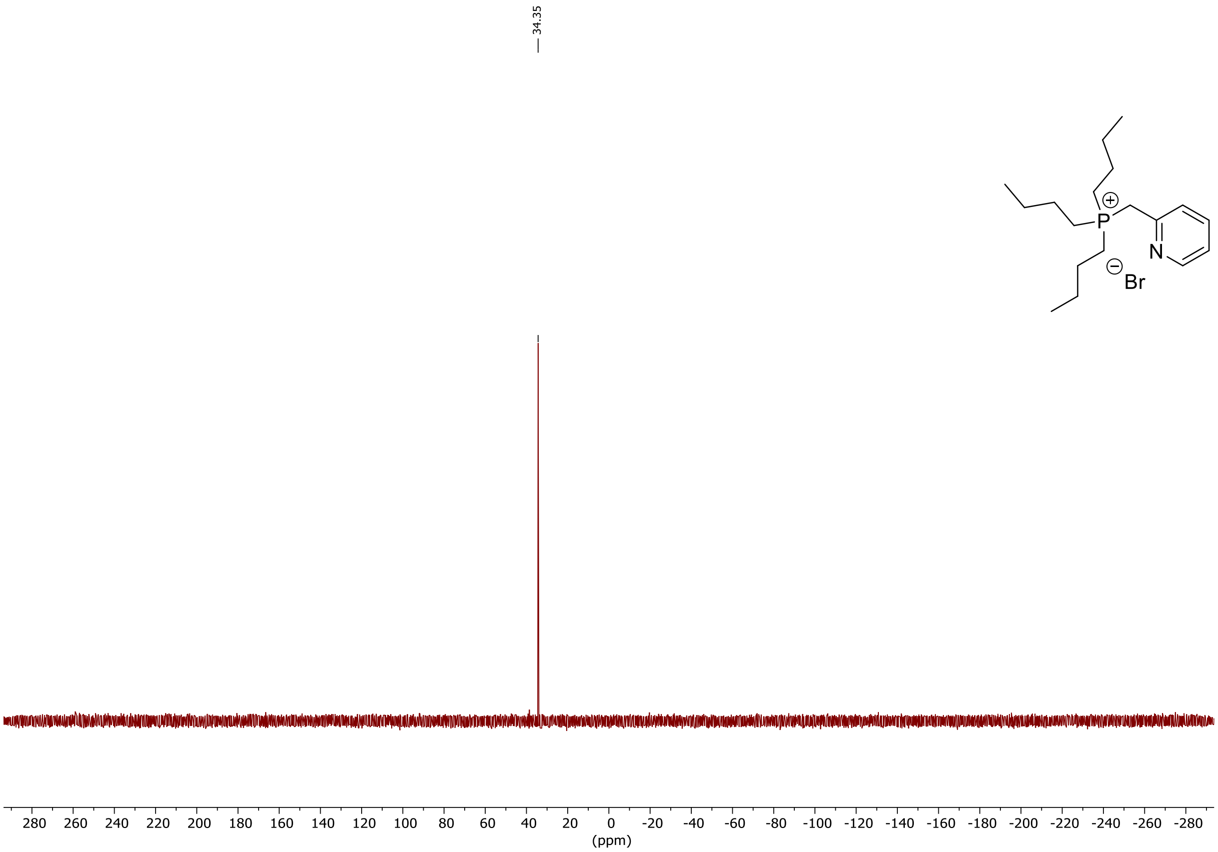

Fig. S190 ^{31}P NMR (203 MHz, acetonitrile-d_3, 298 K) spectrum of compound **2a**

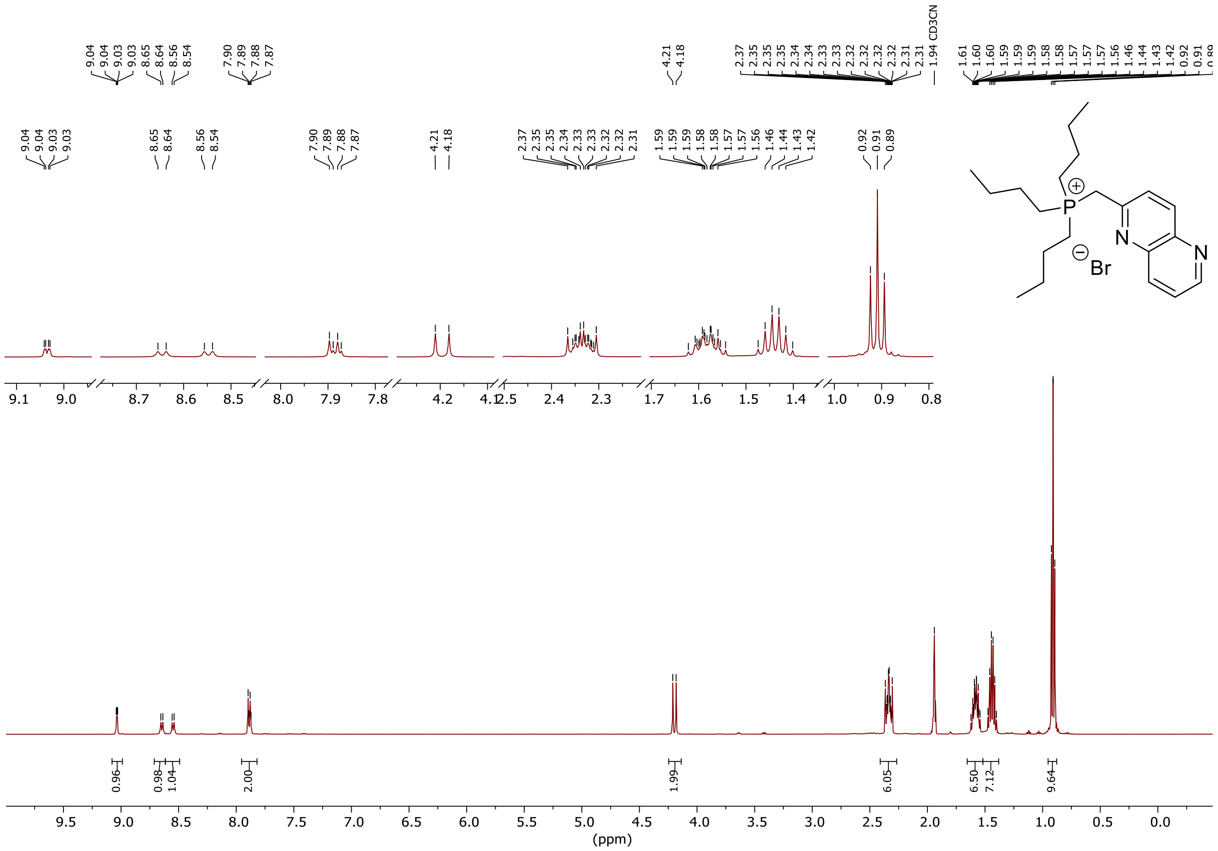

Fig. S191 ^{1}H NMR (501 MHz, acetonitrile-d_3, 298 K) spectrum of compound **2k**

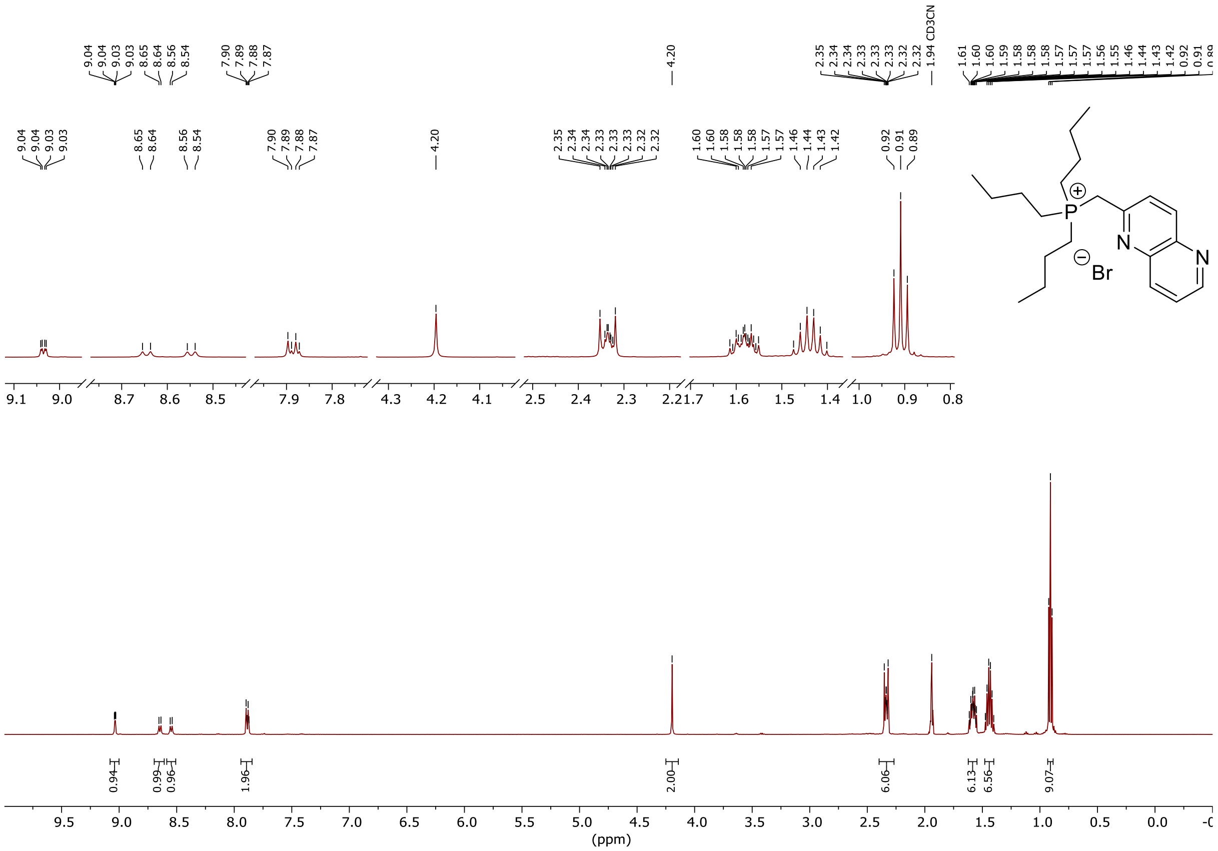

Fig. S192 ^{1}H{^{31}P} NMR (501 MHz, acetonitrile-d_3, 298 K) spectrum of compound **2k**

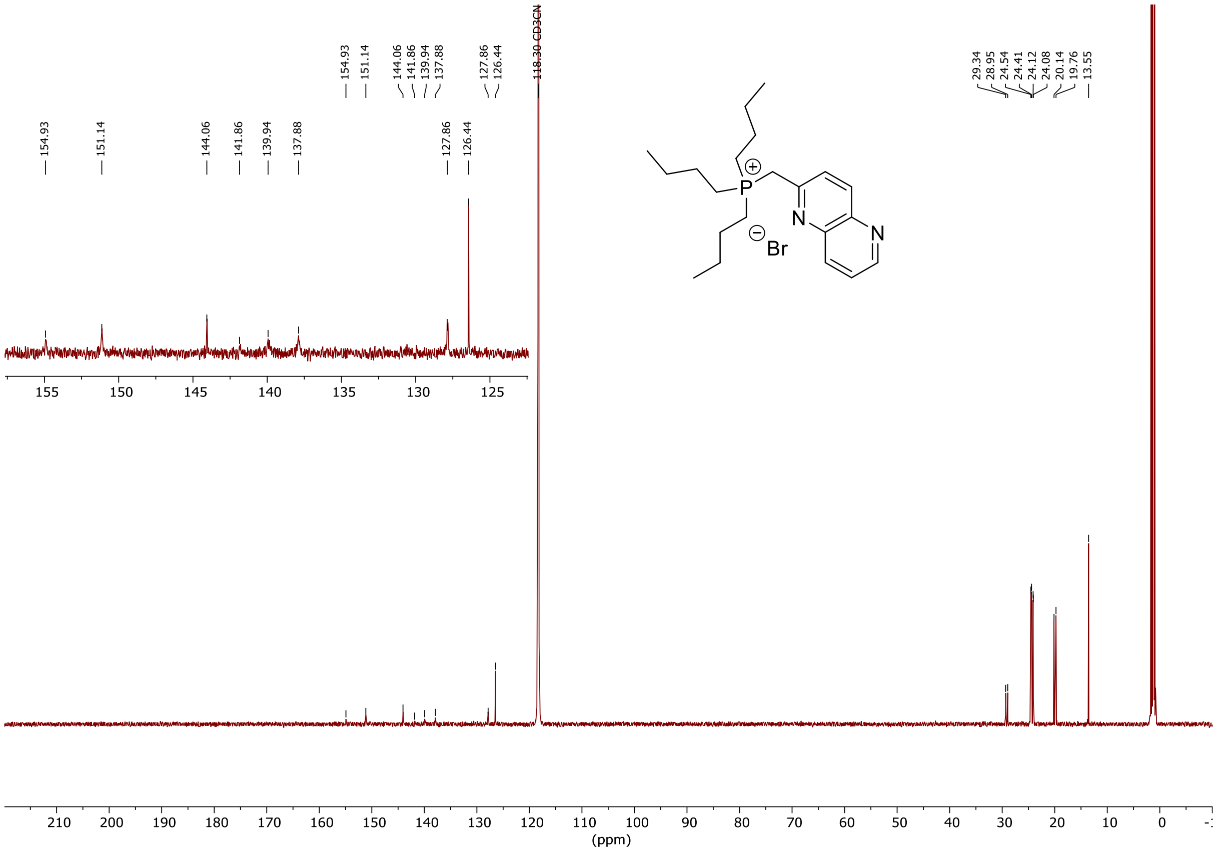

Fig. S193 $^{13}C\{^1H\}$ NMR (126 MHz, acetonitrile-d_3, 298 K) spectrum of compound **2k**

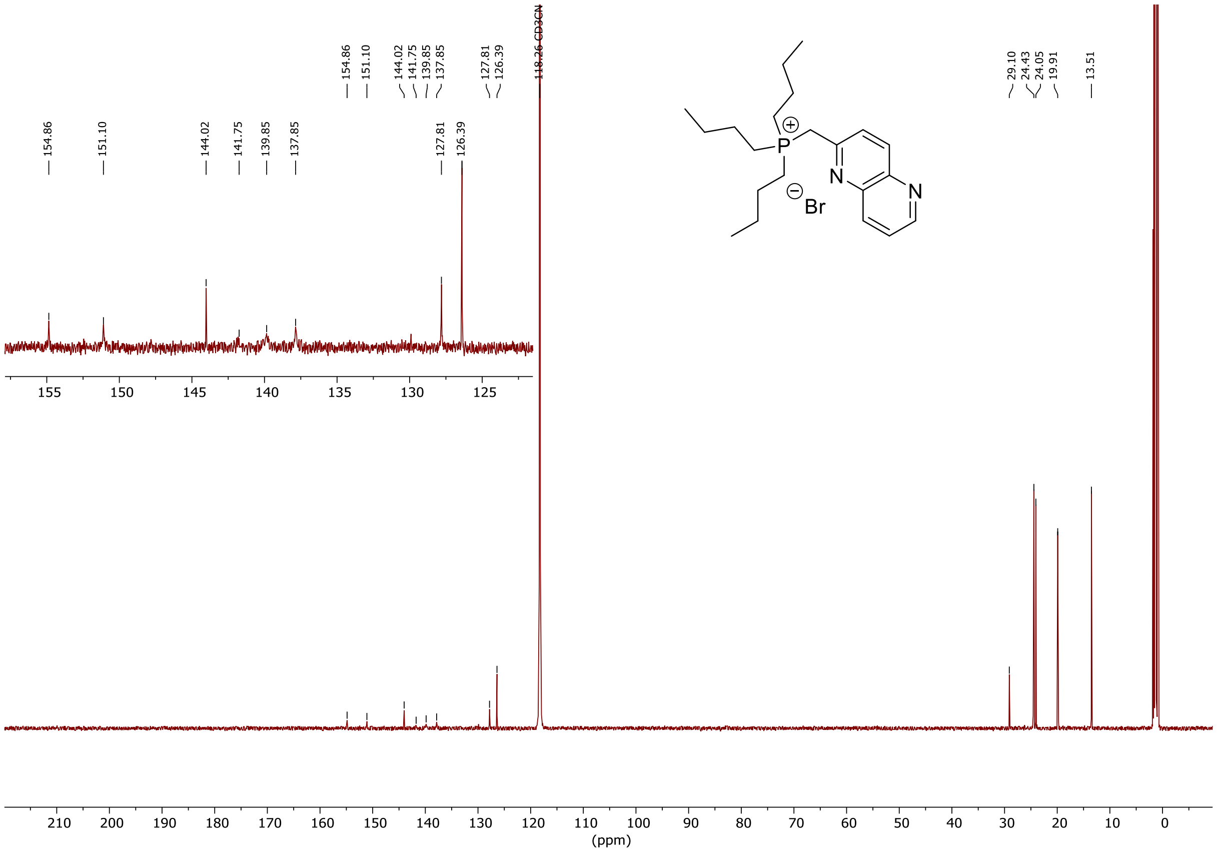

Fig. S194 $^{13}C\{^1H,^{31}P\}$ NMR (126 MHz, acetonitrile-d_3, 298 K) spectrum of compound **2k**

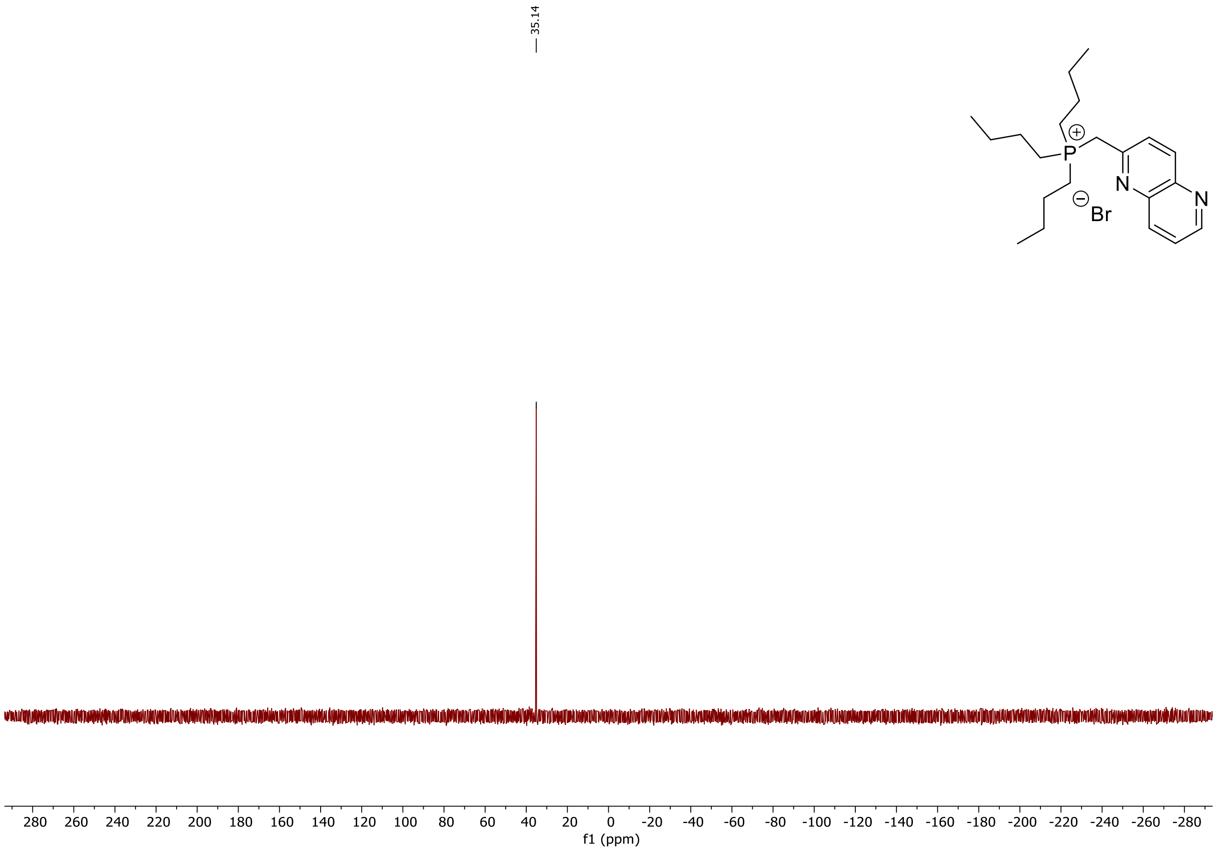

Fig. S195 ^{31}P NMR (203 MHz, acetonitrile-d_3, 298 K) spectrum of compound **2k**

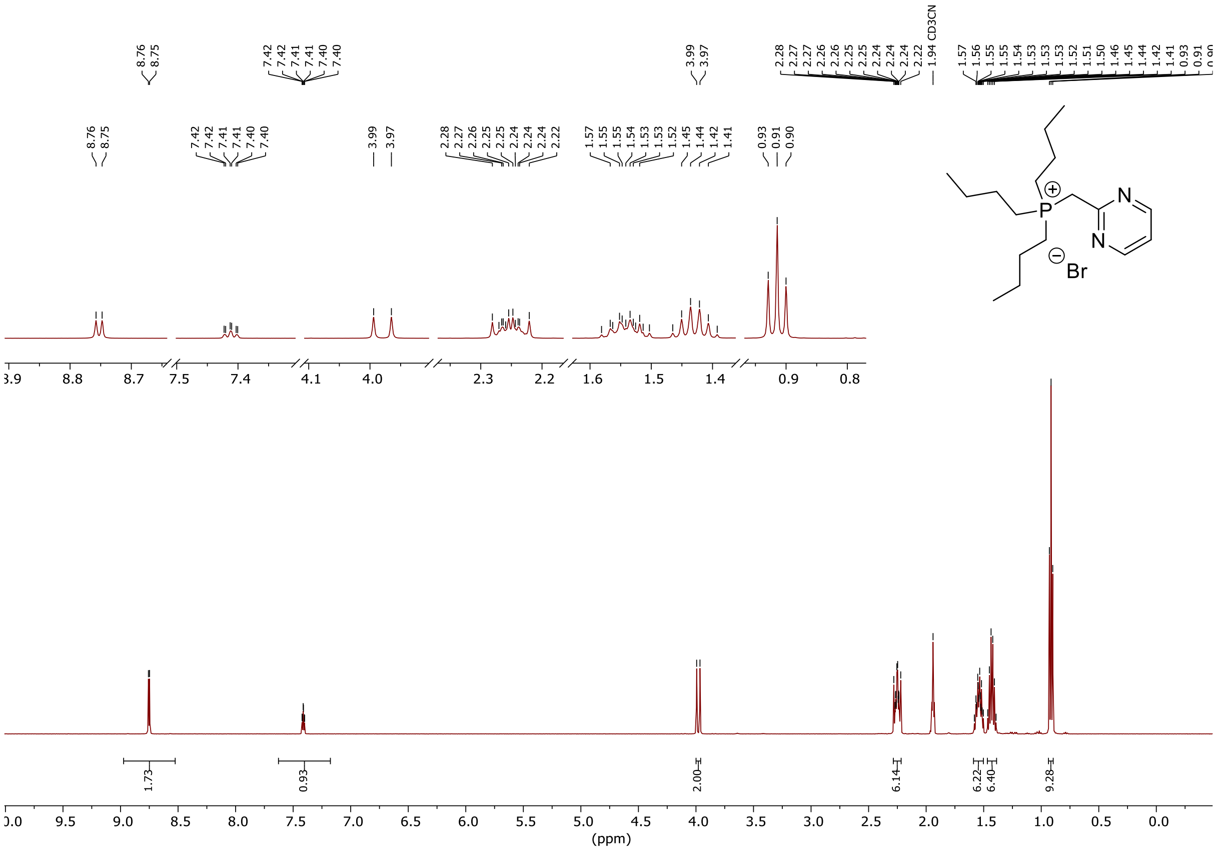

Fig. S196 ^{1}H NMR (501 MHz, acetonitrile-d_3, 298 K) spectrum of compound **2l**

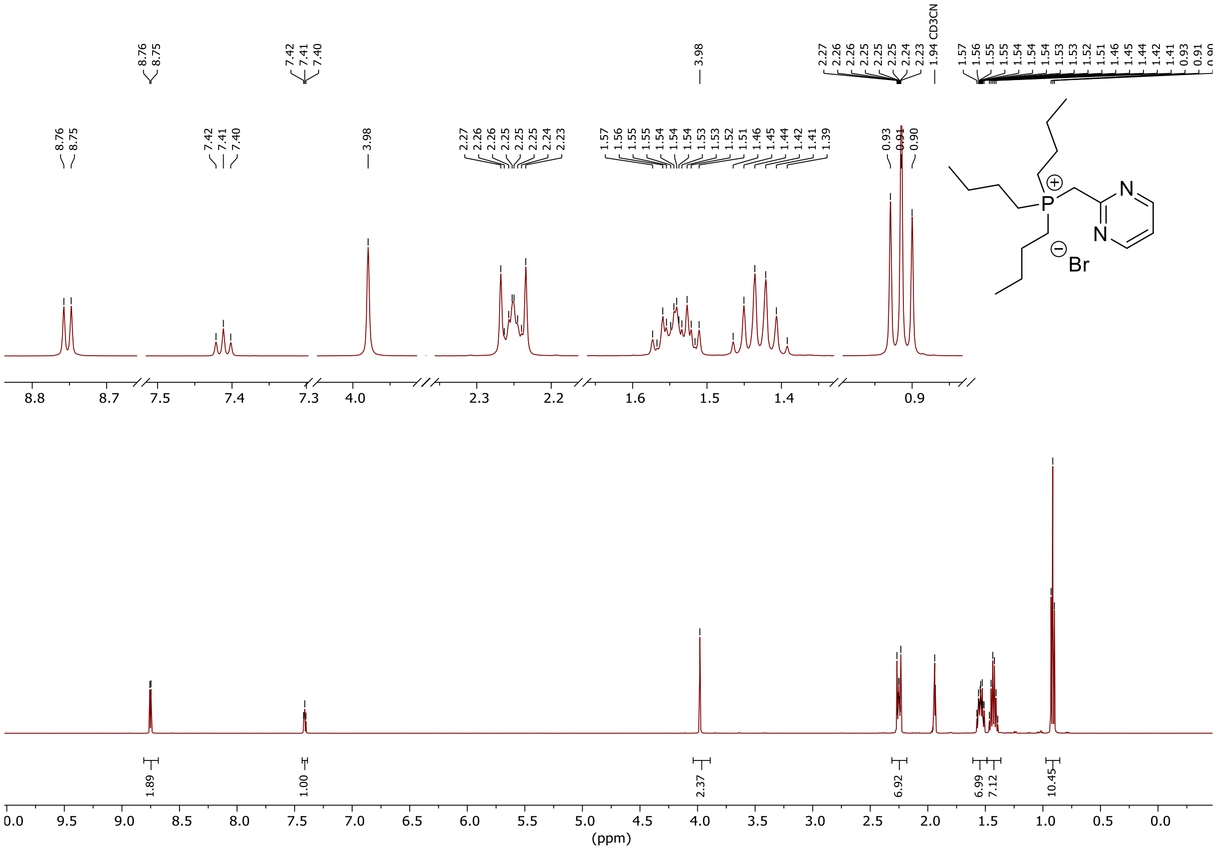

Fig. S197 ^{1}H{^{31}P} NMR (501 MHz, acetonitrile-d_3, 298 K) spectrum of compound **2l**

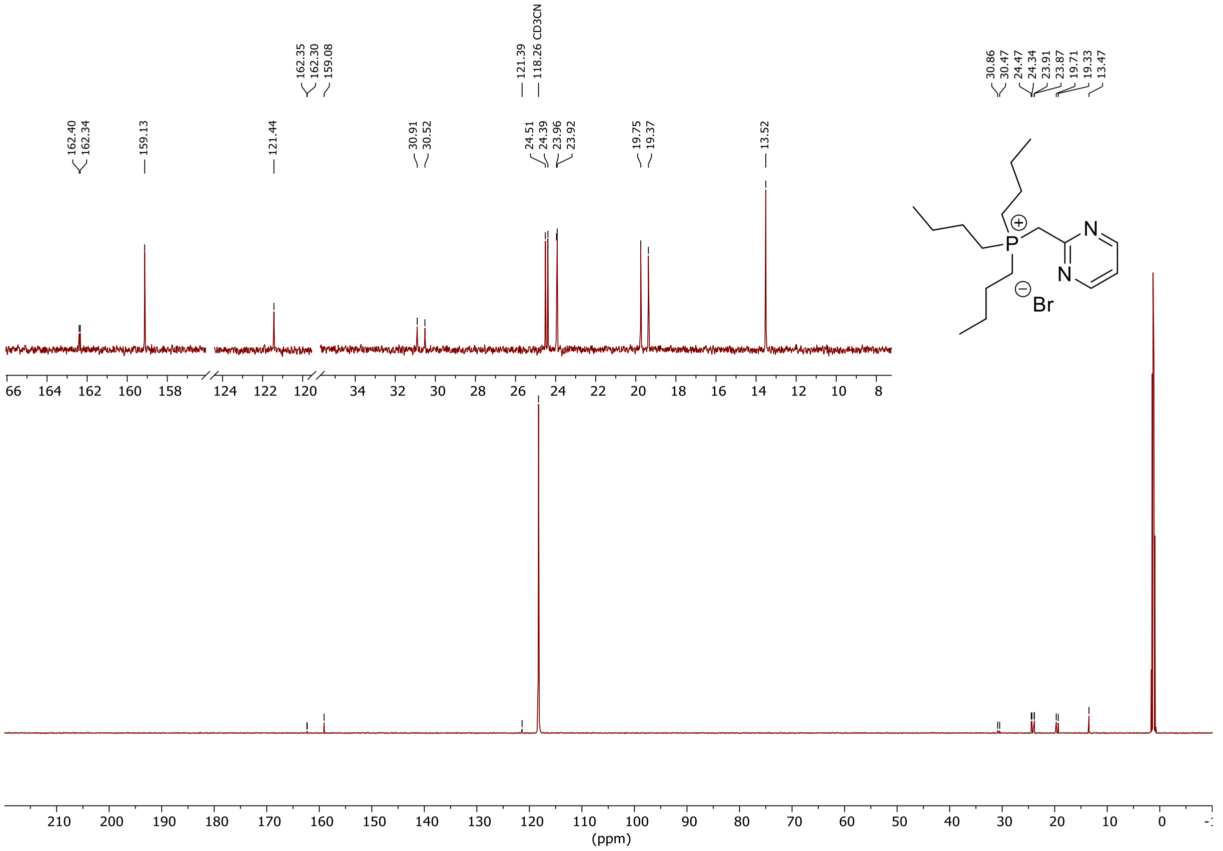

Fig. S198 $^{13}C\{^{1}H\}$ NMR (126 MHz, acetonitrile-d_3, 298 K) spectrum of compound **2l**

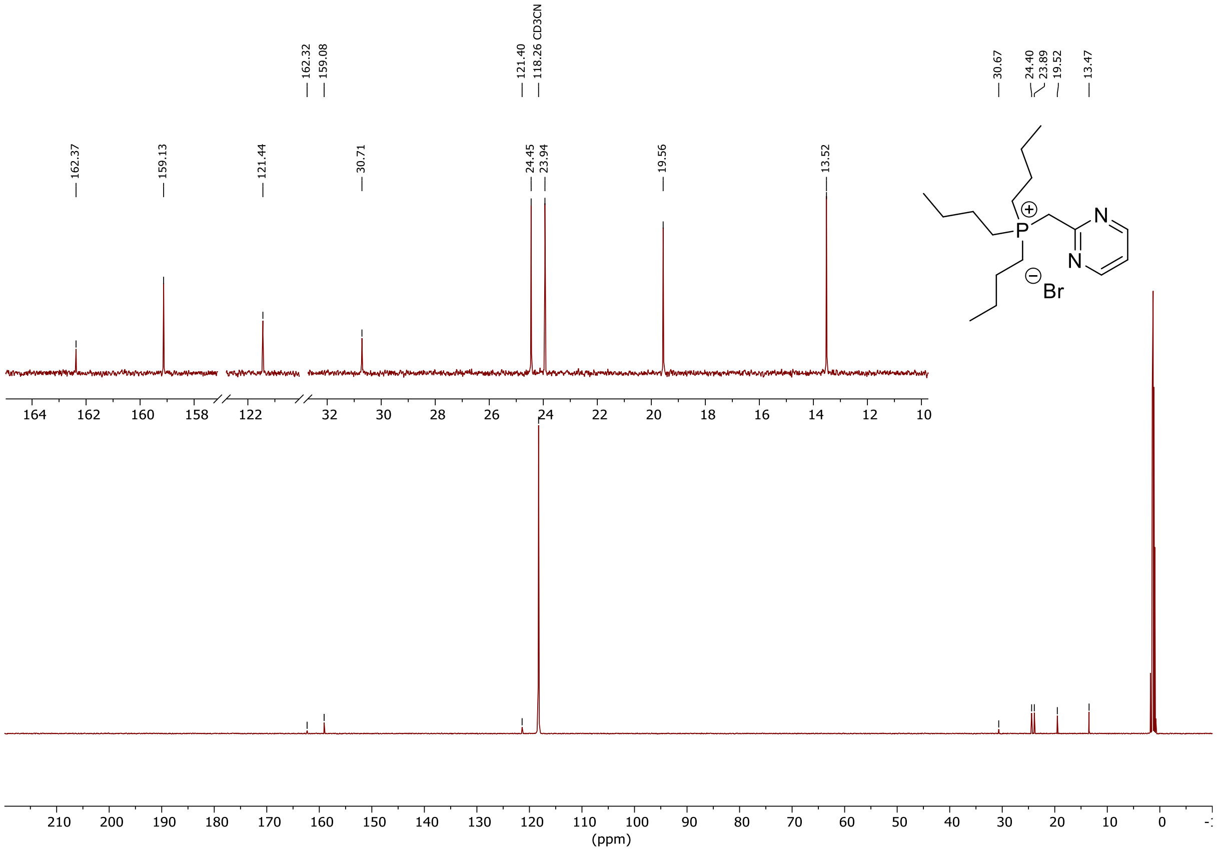

Fig. S199 $^{13}C\{^{1}H,^{31}P\}$ NMR (126 MHz, acetonitrile-d_3, 298 K) spectrum of compound **2l**

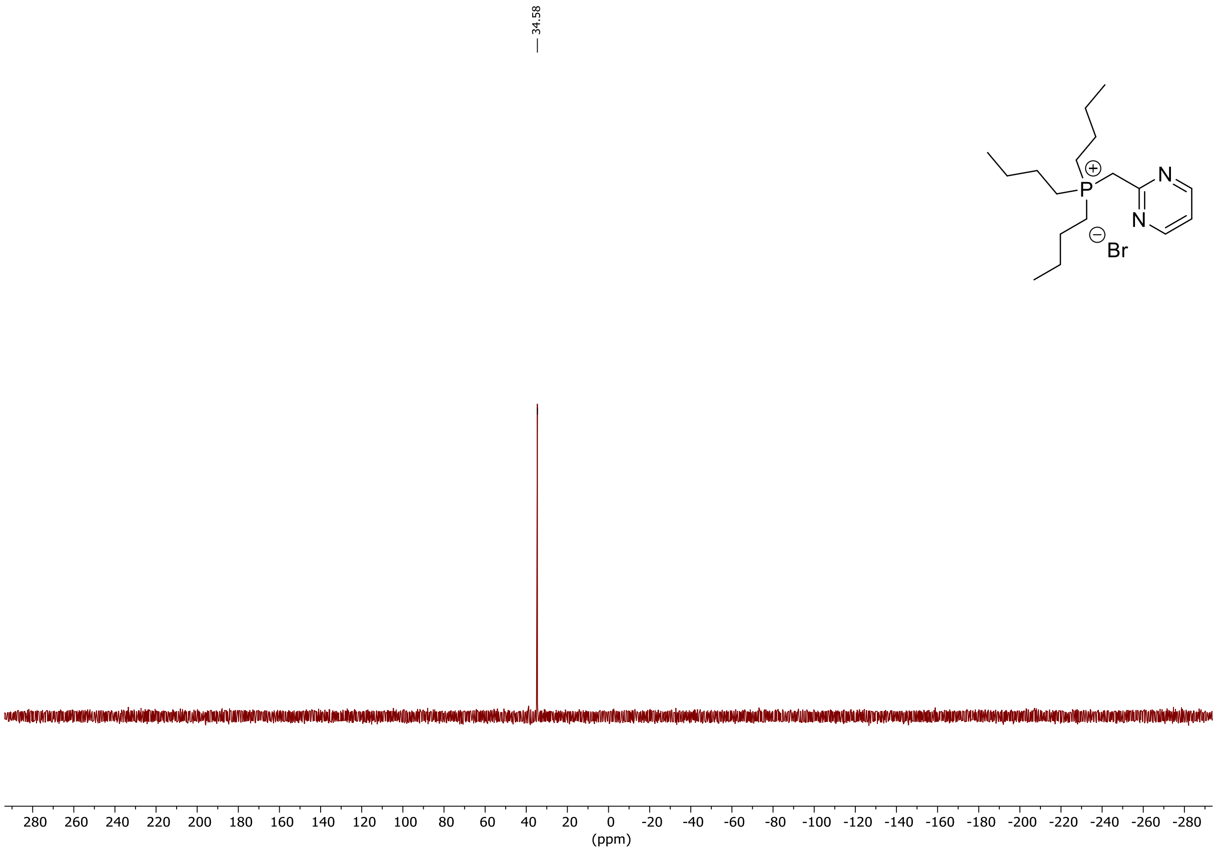

Fig. S200 ^{31}P NMR (203 MHz, acetonitrile-d_3, 298 K) spectrum of compound **2l**

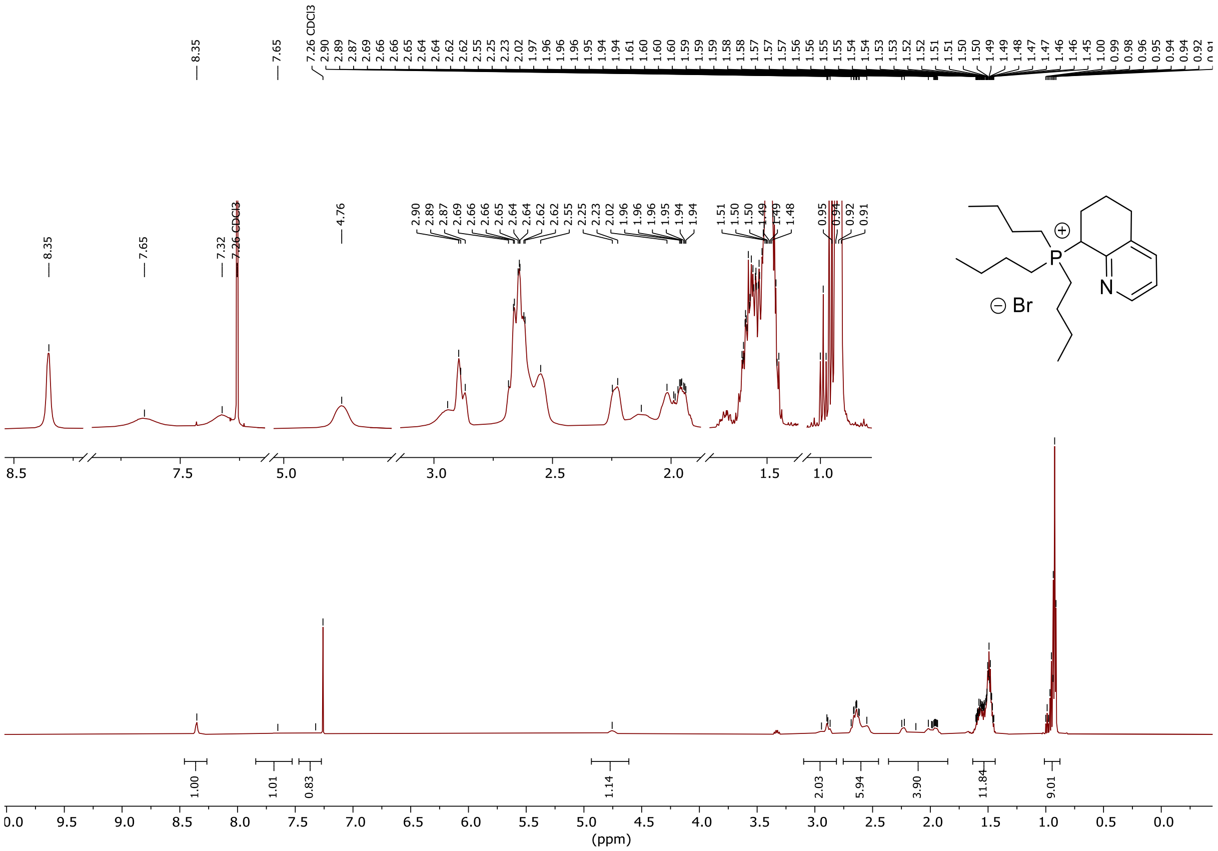

Fig. S201 ^{1}H NMR (600 MHz, chloroform-*d*, 298 K) spectrum of compound **2m**

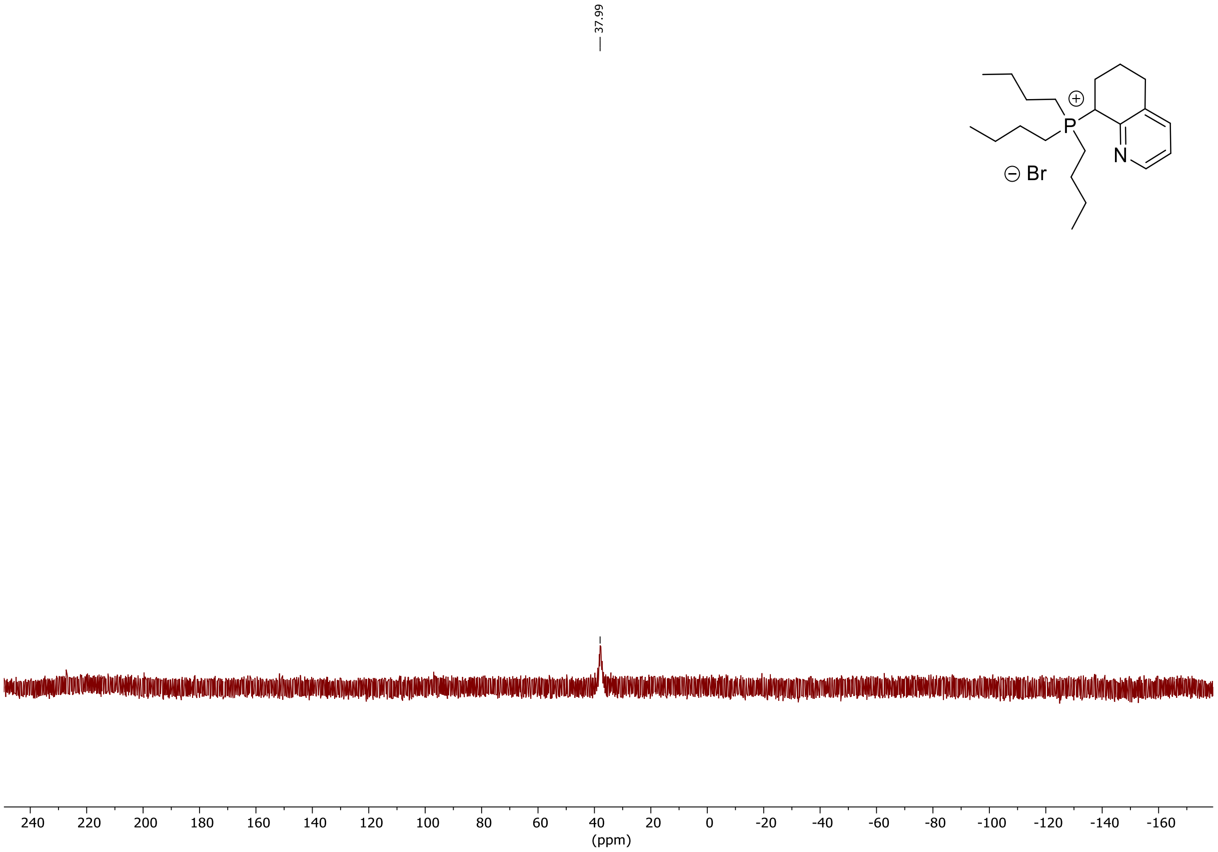

Fig. S202 ^{31}P NMR (243 MHz, chloroform-*d*, 298 K) spectrum of compound **2m**

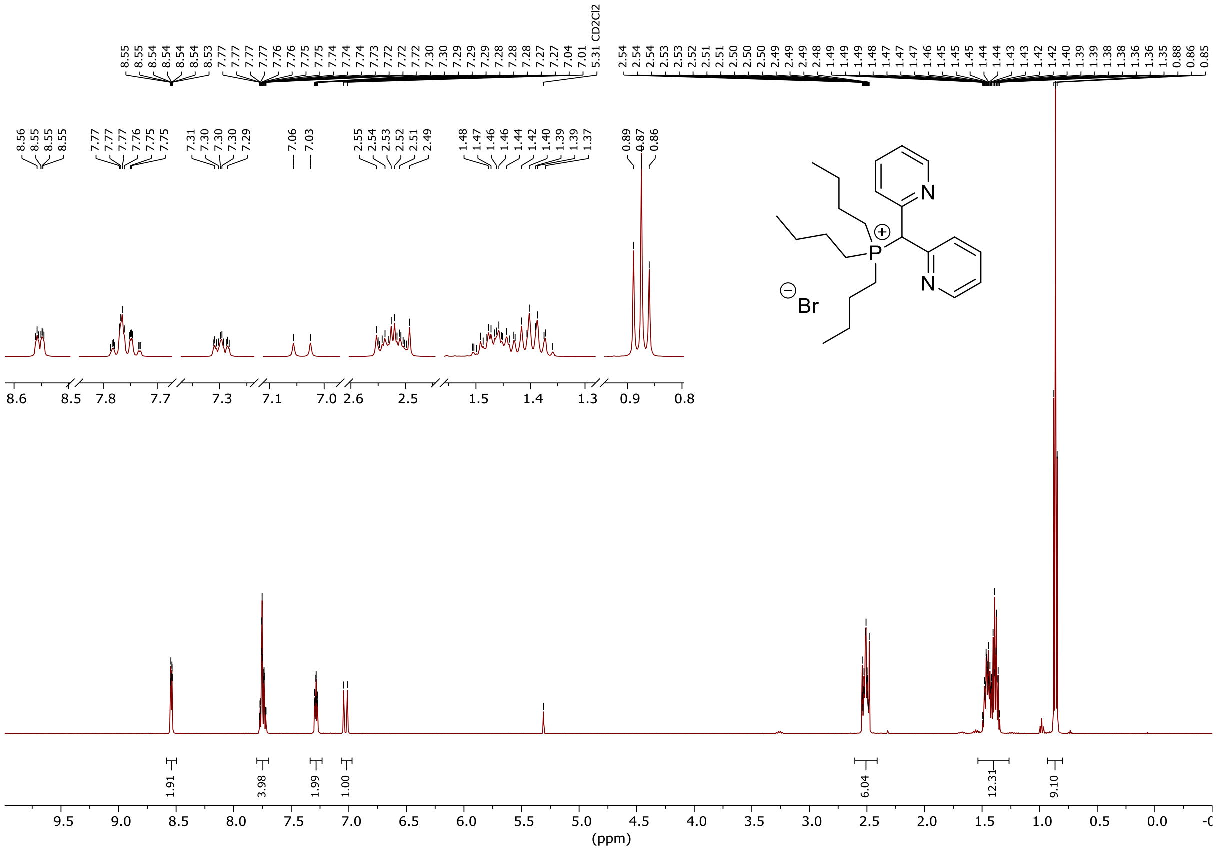

Fig. S203 ^{1}H NMR (501 MHz, methylene chloride-d_2, 298 K) spectrum of compound **2n**

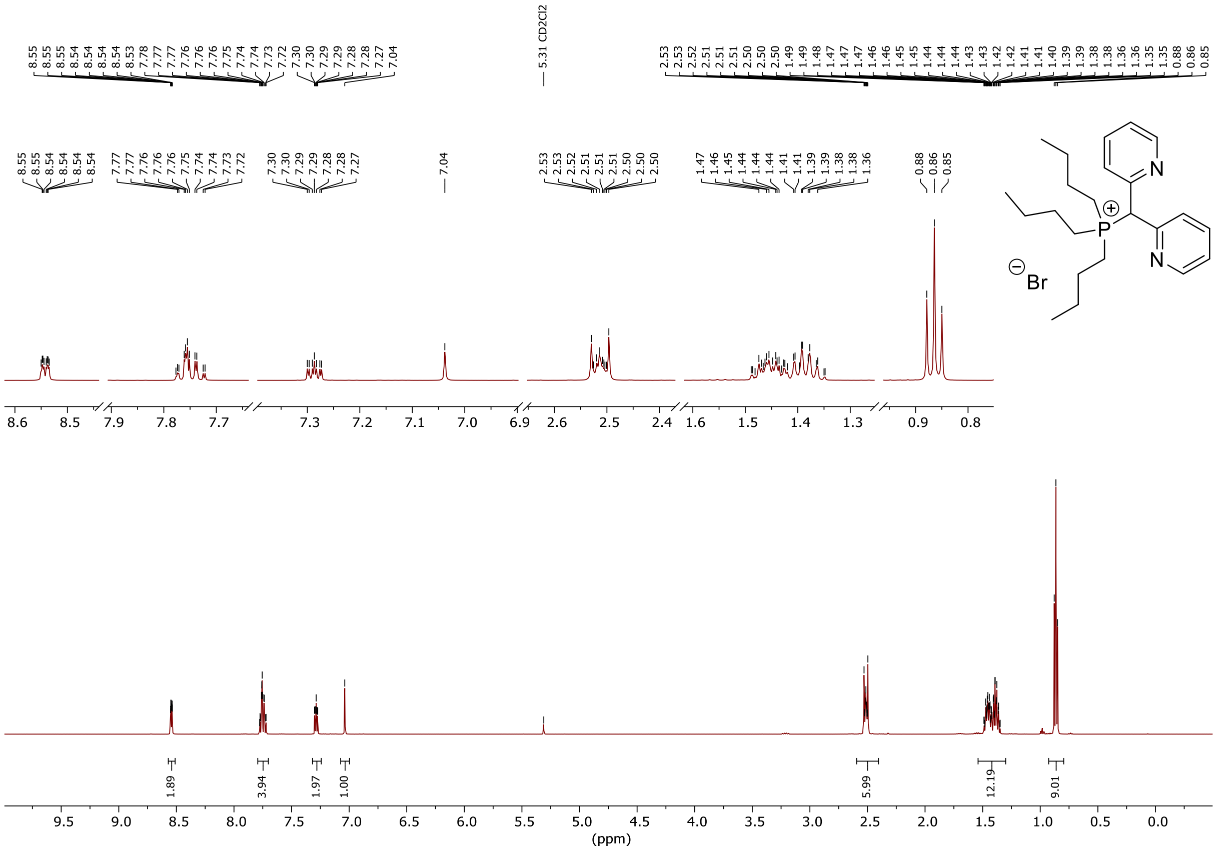

Fig. S204 $^{1}H\{^{31}P\}$ NMR (501 MHz, methylene chloride-d_2, 298 K) spectrum of compound **2n**

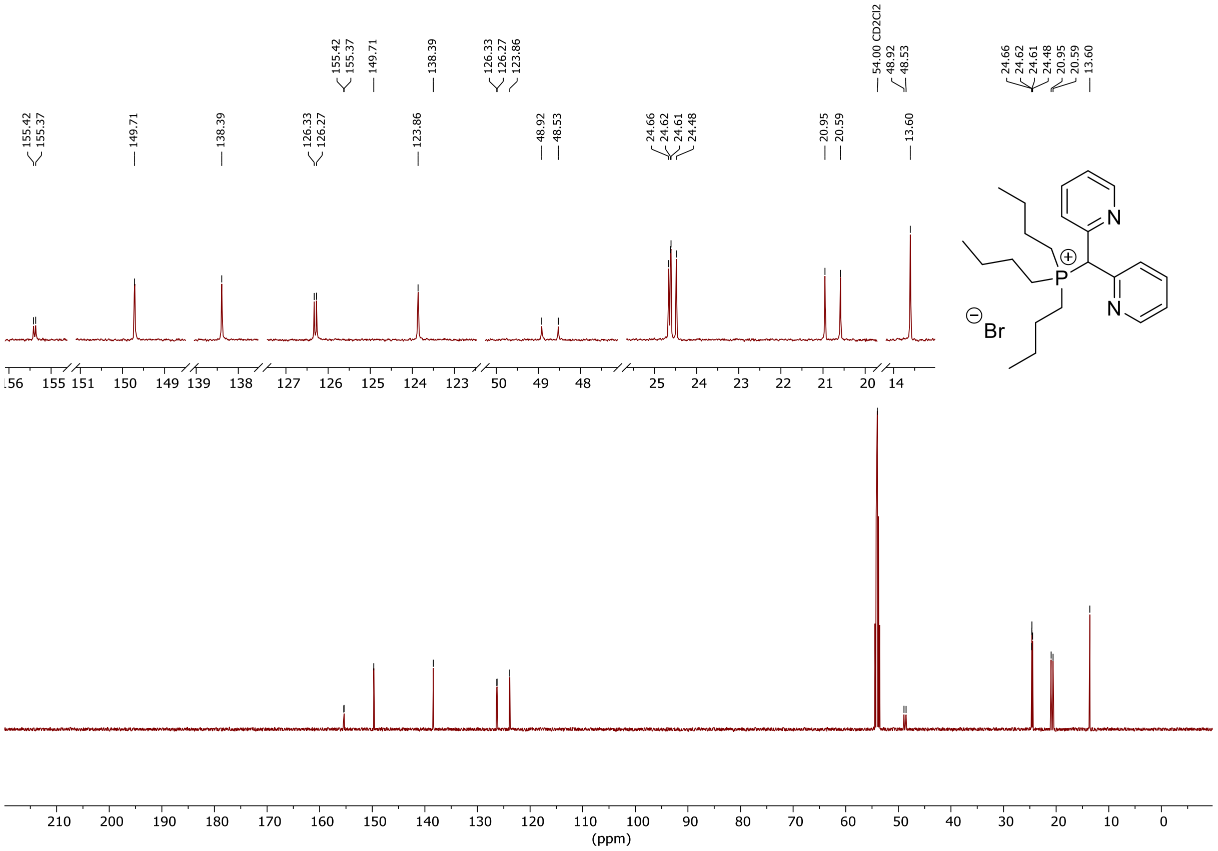

Fig. S205 $^{13}C\{^{1}H\}$ NMR (126 MHz, methylene chloride-d_2, 298 K) spectrum of compound **2n**

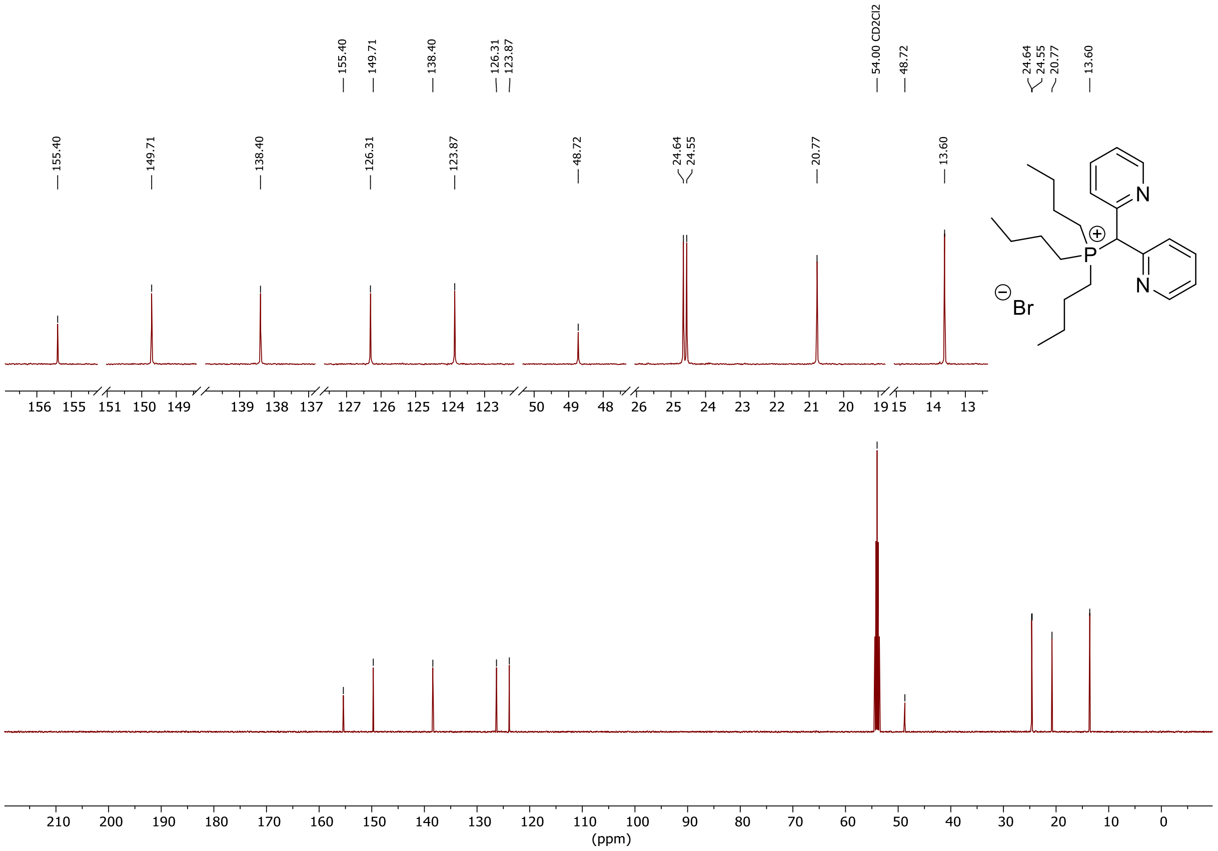

Fig. S206 $^{13}C\{^{1}H,^{31}P\}$ NMR (126 MHz, methylene chloride-d_2, 298 K) spectrum of compound **2n**

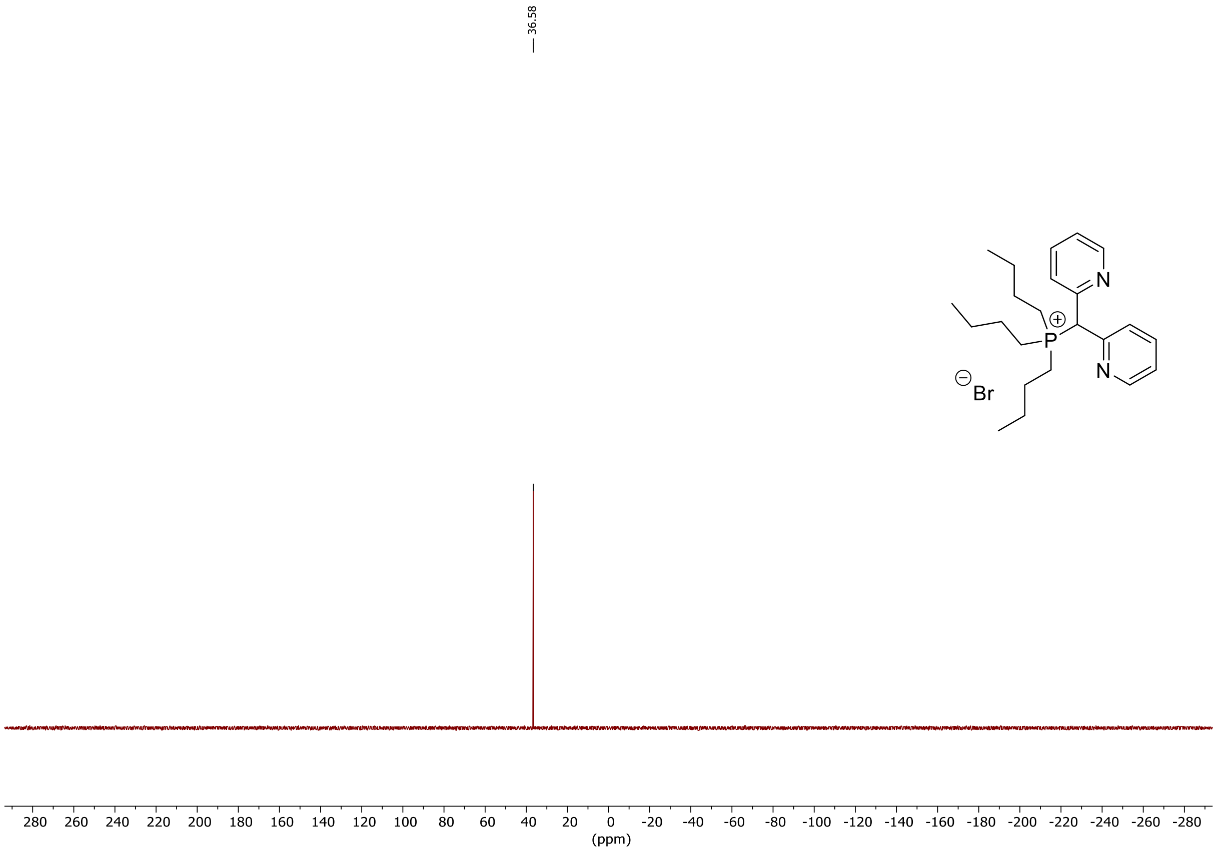

Fig. S207 ^{31}P NMR (203 MHz, methylene chloride-d_2, 298 K) spectrum of compound **2n**

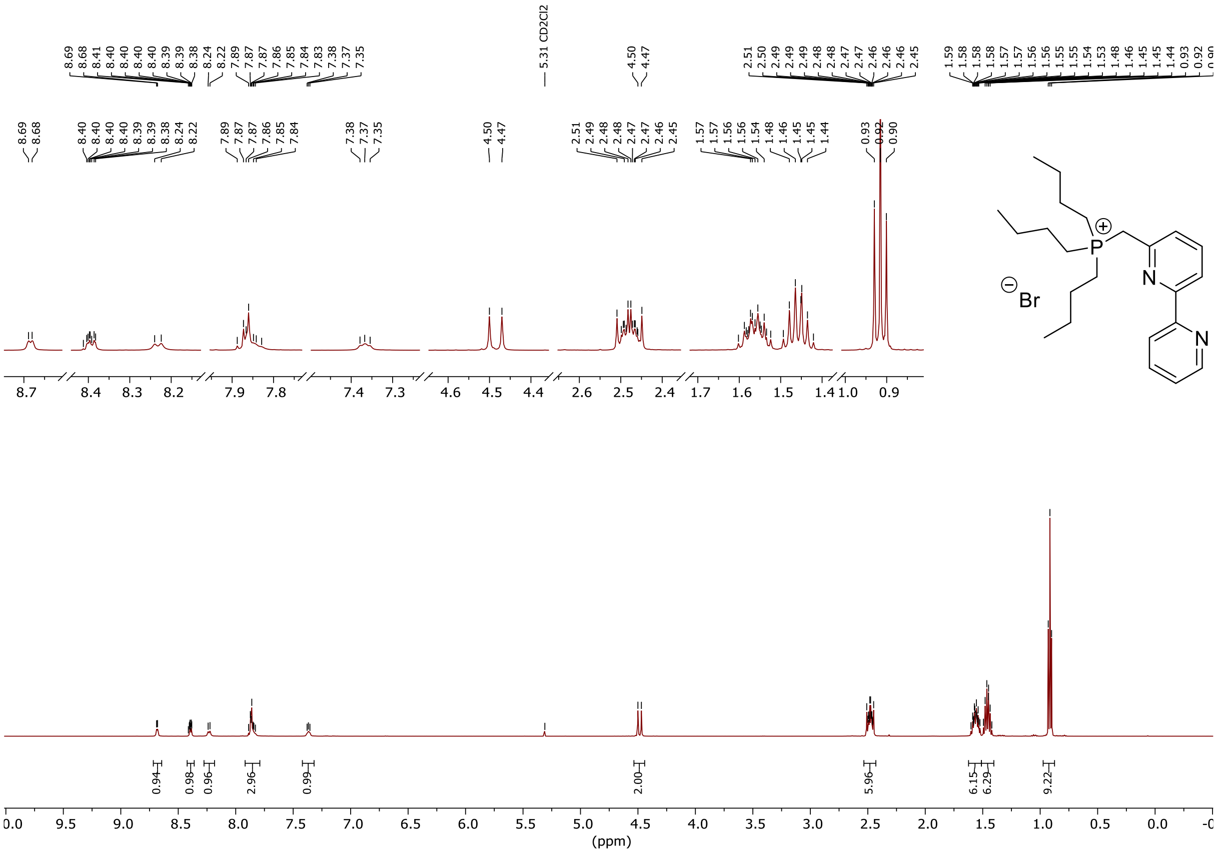

Fig. S208 ^{1}H NMR (501 MHz, methylene chloride-d_2, 298 K) spectrum of compound **2p**

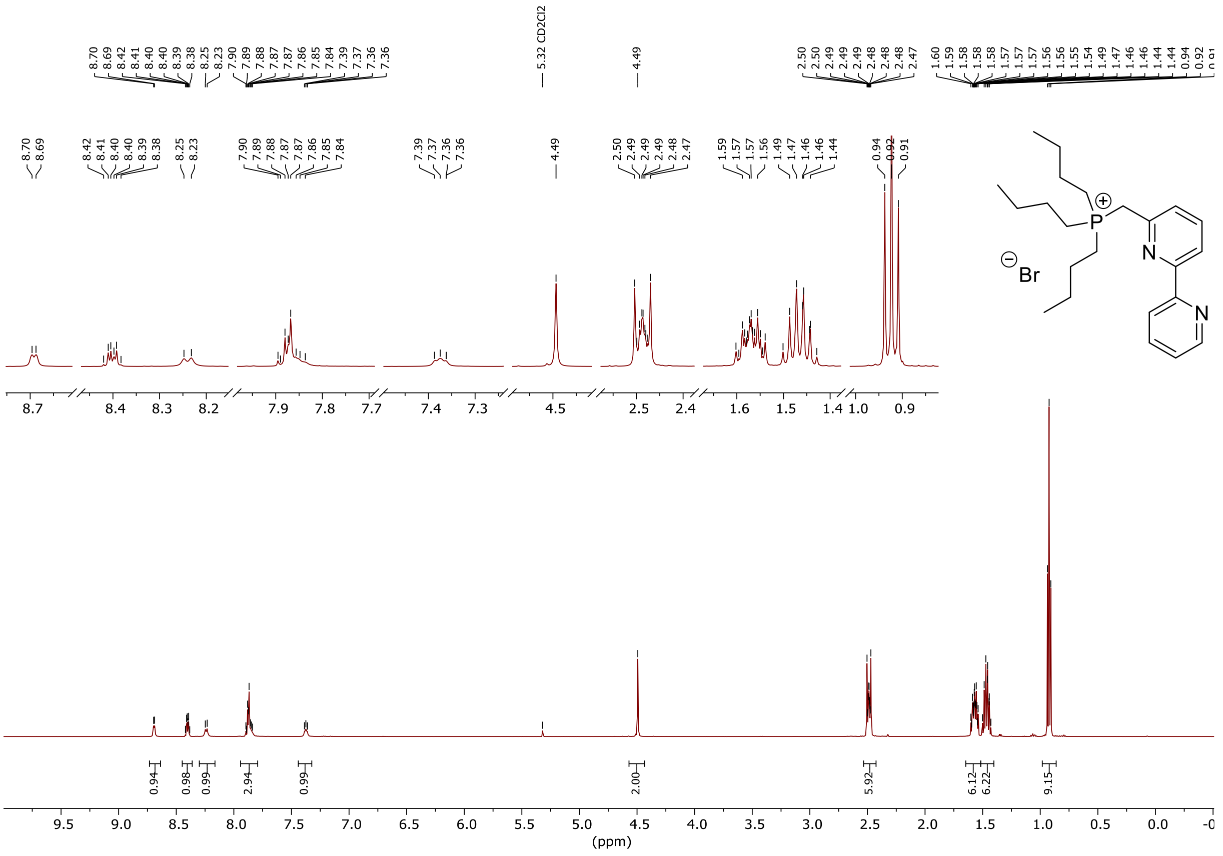

Fig. S209 ^{1}H{^{31}P} NMR (501 MHz, methylene chloride-d_2, 298 K) spectrum of compound **2p**

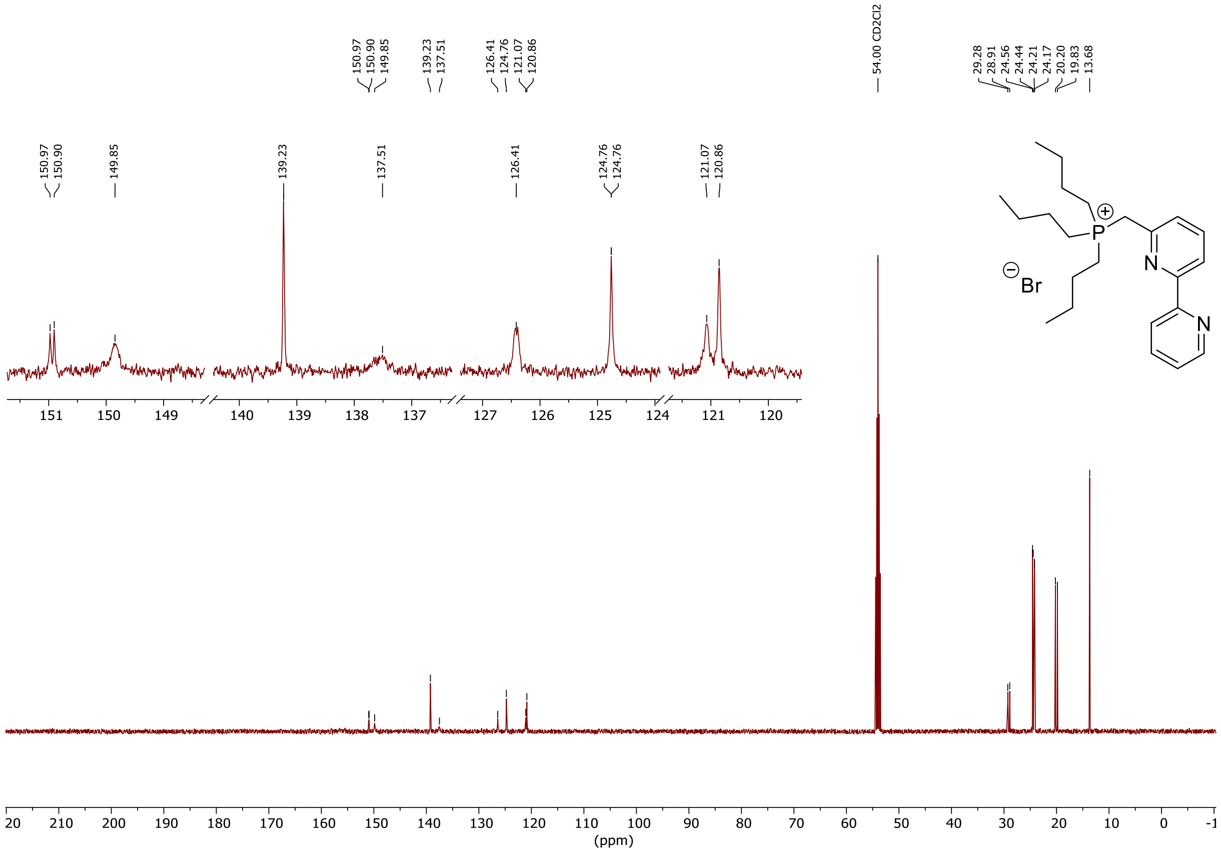

Fig. S210 $^{13}C\{^{1}H\}$ NMR (126 MHz, methylene chloride-d_2, 298 K) spectrum of compound **2p**

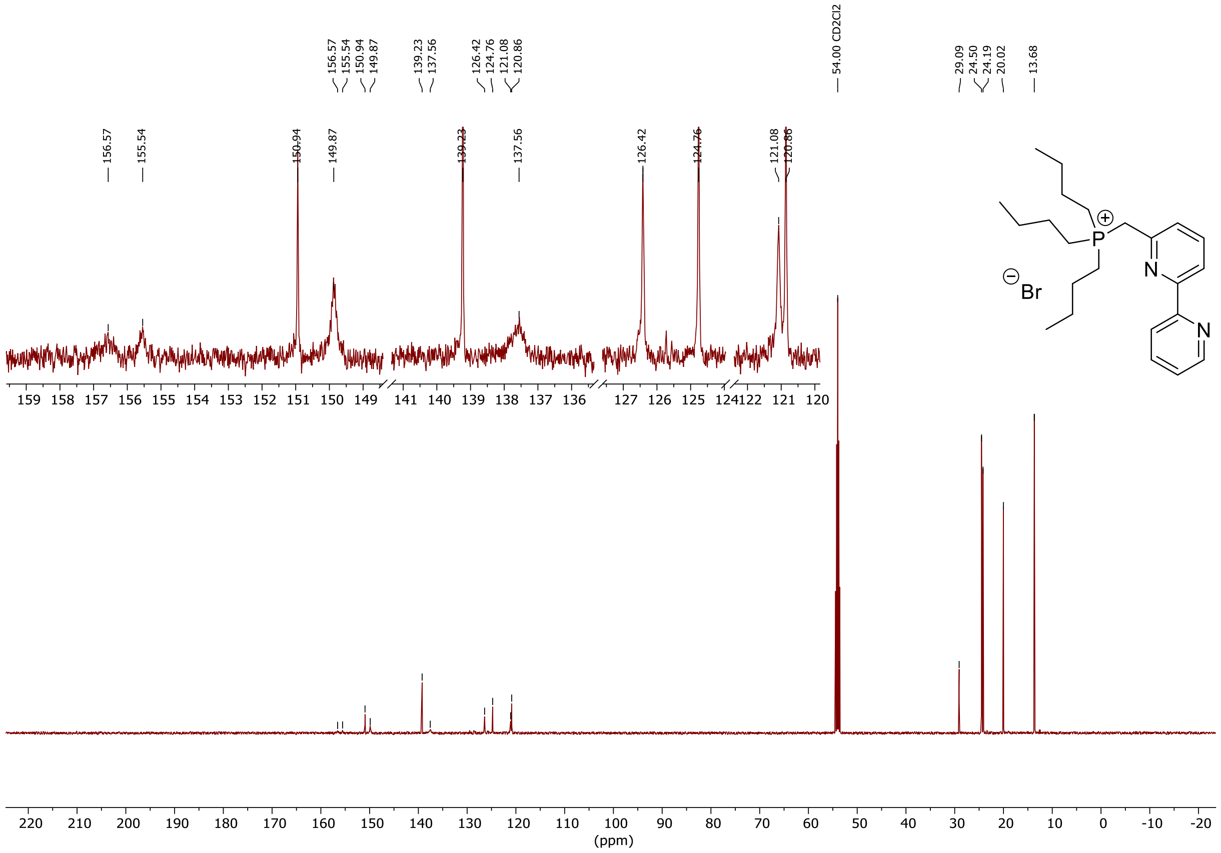

Fig. S211 $^{13}C\{^{1}H,^{31}P\}$ NMR (126 MHz, methylene chloride-d_2, 298 K) spectrum of compound **2p**

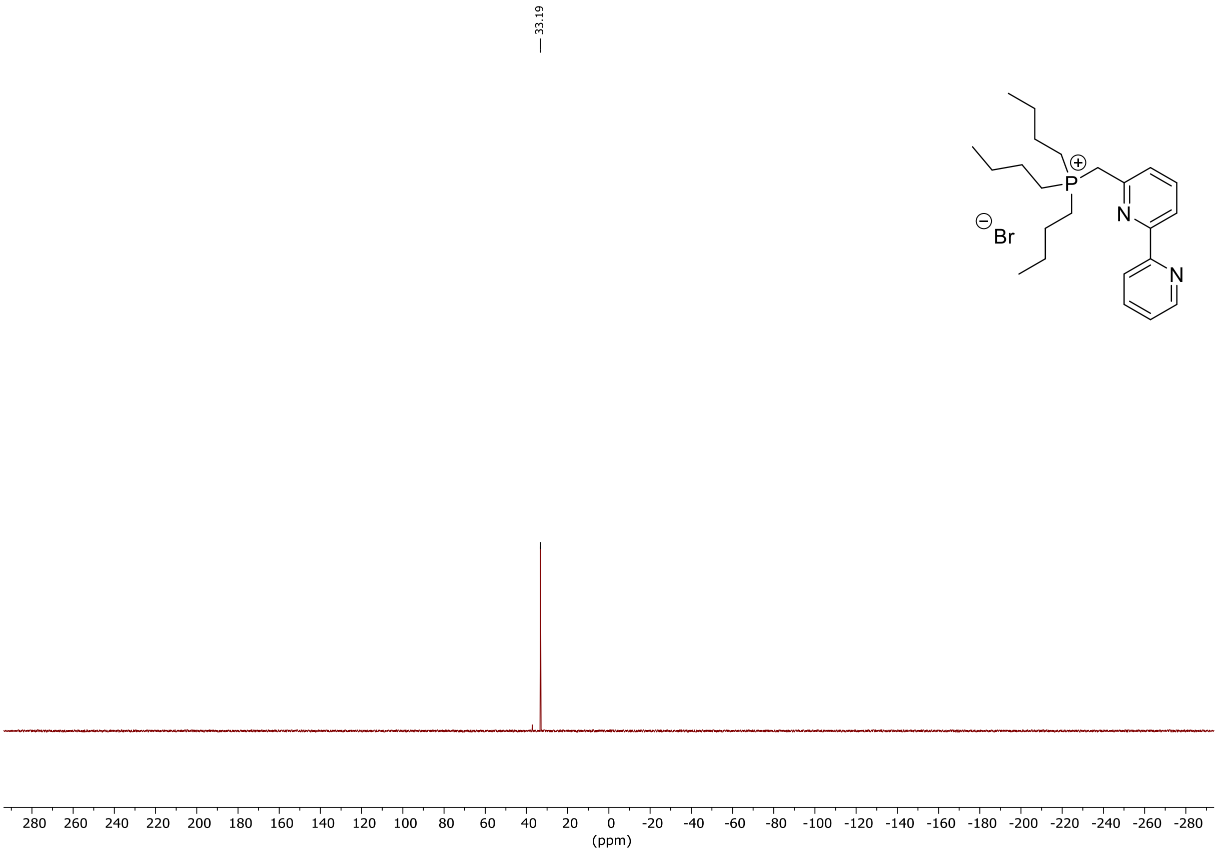

Fig. S212 ^{31}P NMR (203 MHz, methylene chloride-d_2, 298 K) spectrum of compound **2p**

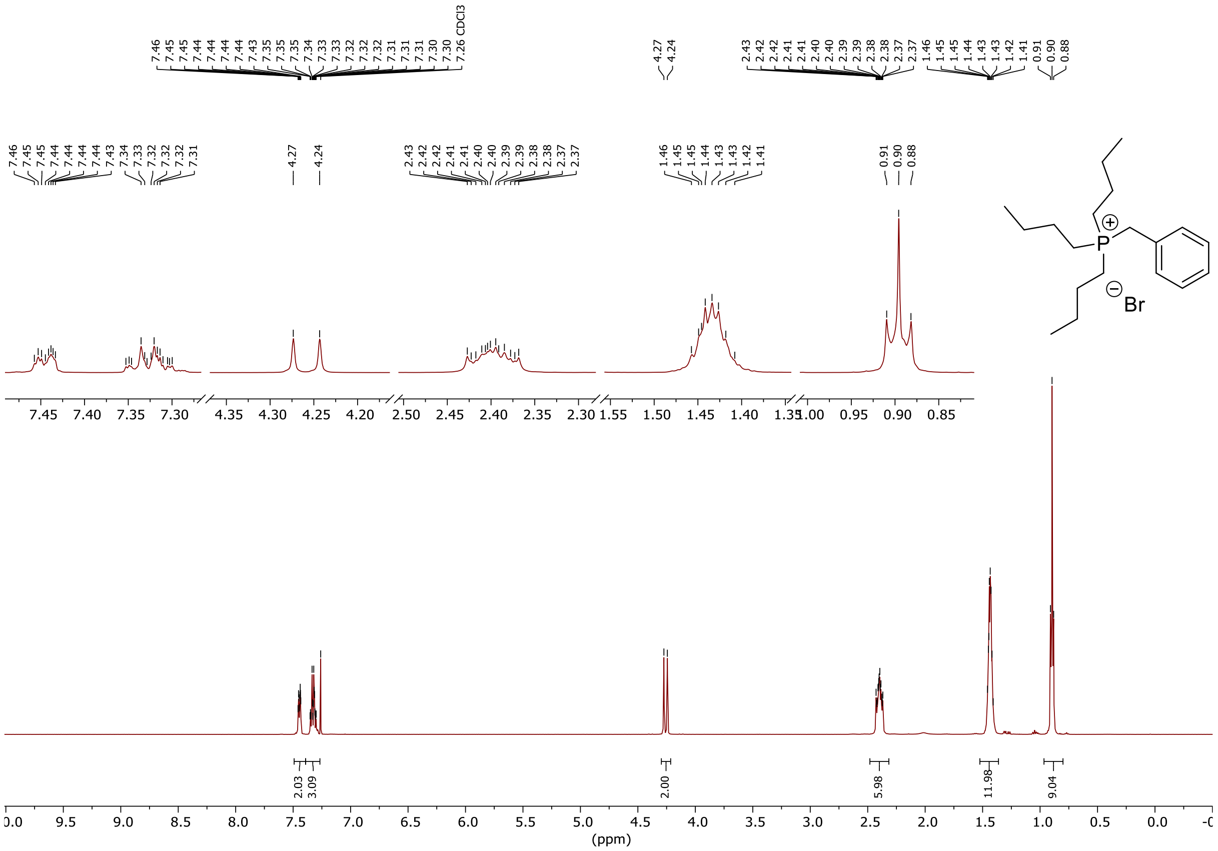

Fig. S213 ^{1}H NMR (501 MHz, chloroform-*d*, 298 K) spectrum of compound **9a**

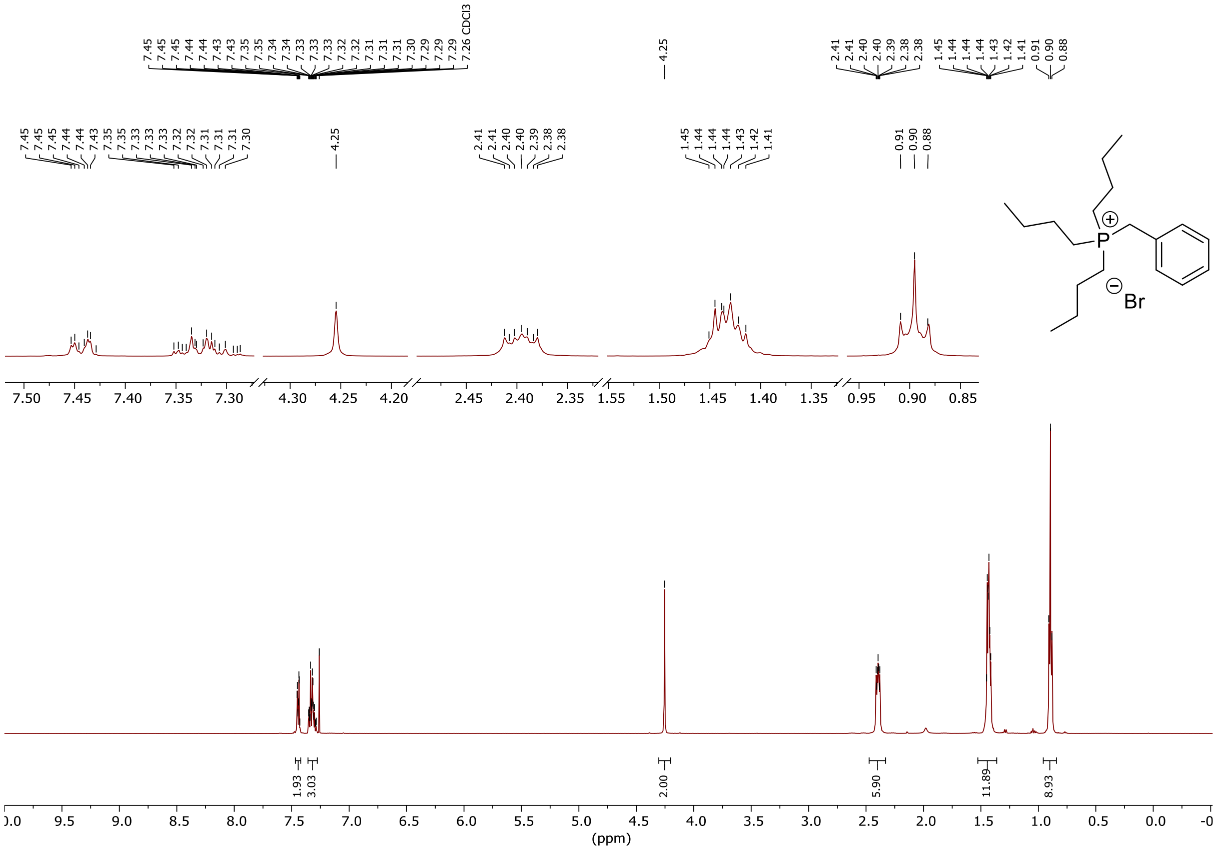

Fig. S214 ^{1}H{^{31}P} NMR (501 MHz, chloroform-*d*, 298 K) spectrum of compound **9a**

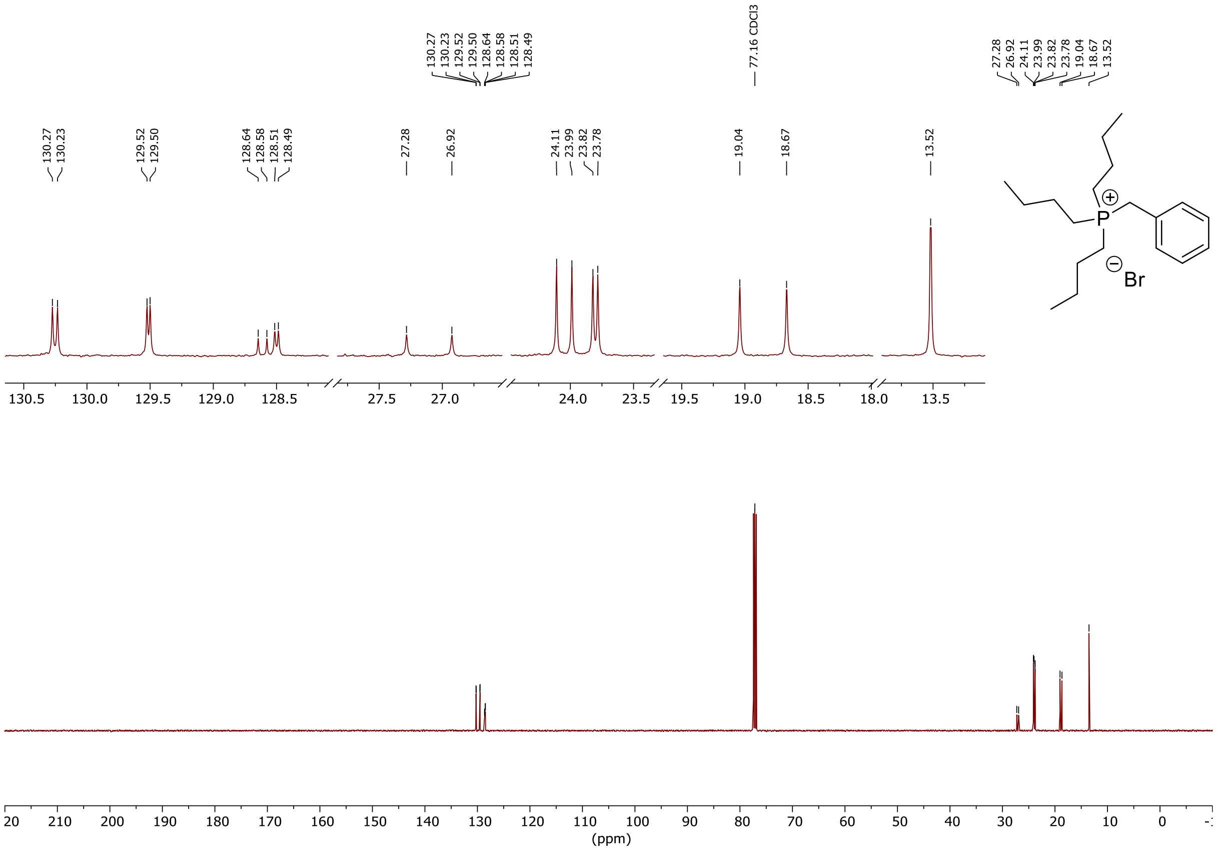

Fig. S215 $^{13}C\{^1H\}$ NMR (126 MHz, chloroform-*d*, 298 K) spectrum of compound **9a**

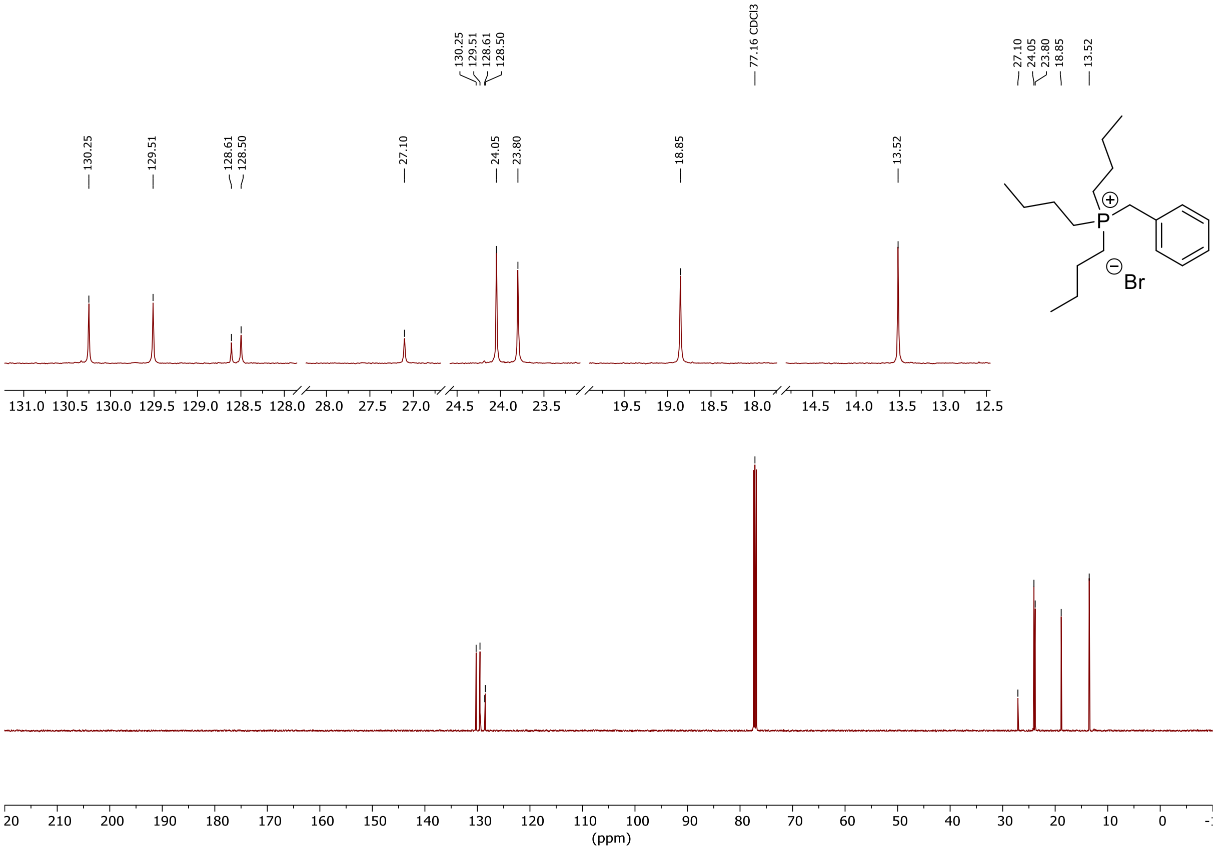

Fig. S216 $^{13}C\{^1H,^{31}P\}$ NMR (126 MHz, chloroform-*d*, 298 K) spectrum of compound **9a**

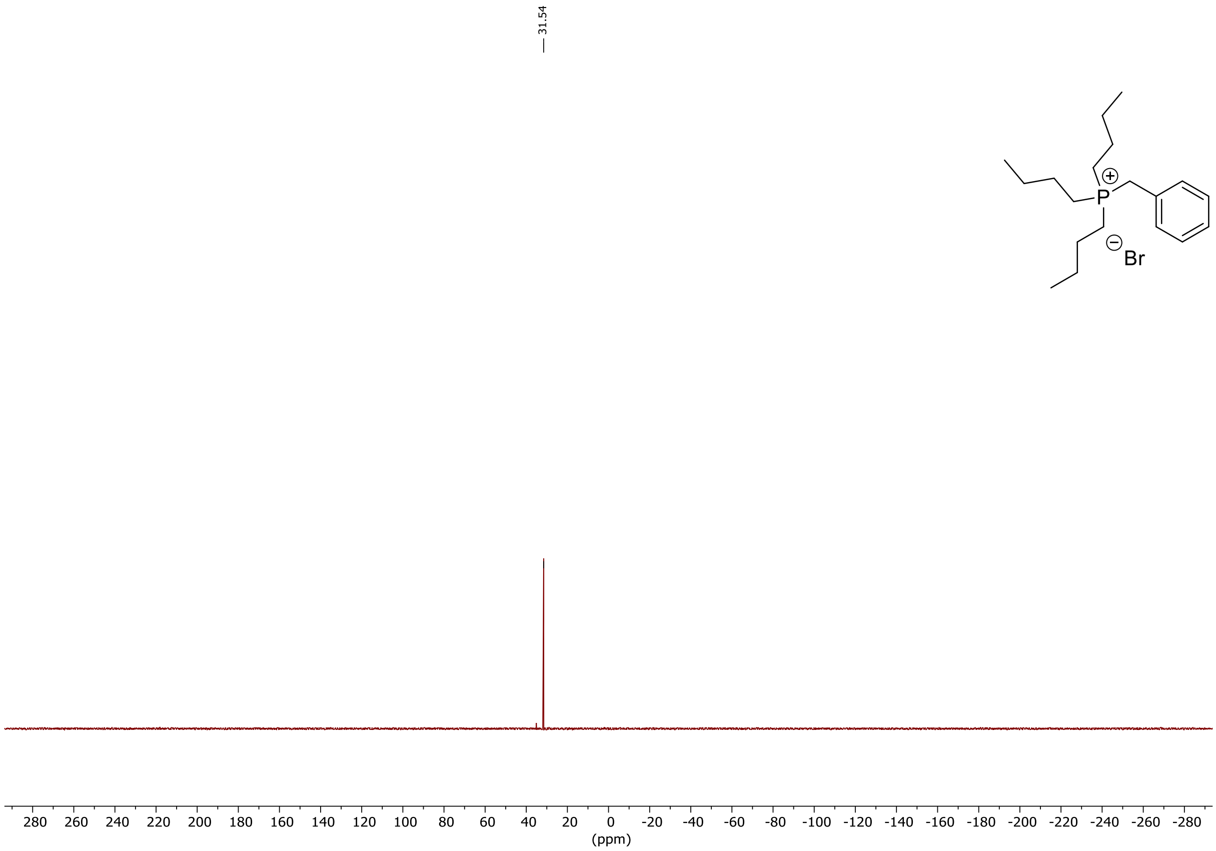

Fig. S217 ^{31}P NMR (203 MHz, chloroform-*d*, 298 K) spectrum of compound **9a**

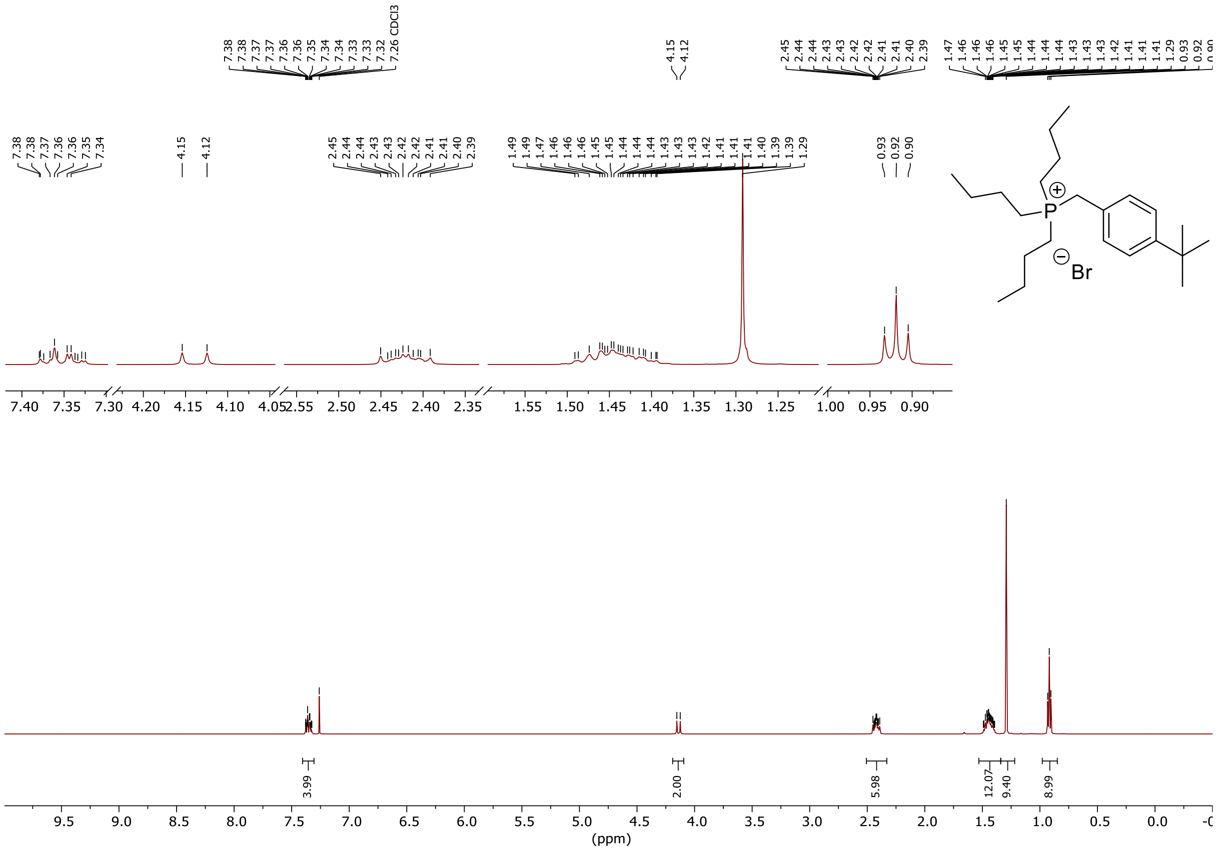

Fig. S218 ^{1}H NMR (501 MHz, chloroform-*d*, 298 K) spectrum of compound **9b**

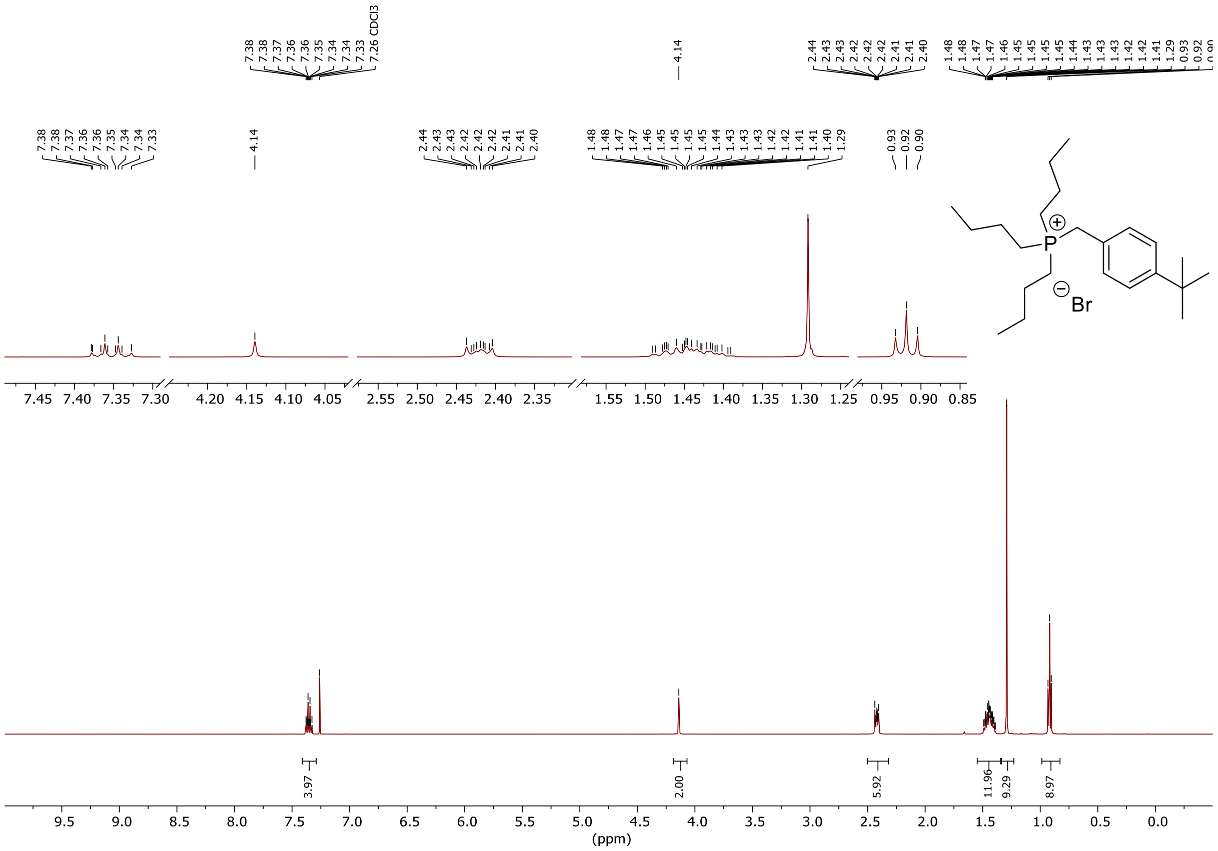

Fig. S219 ^{1}H{^{31}P} NMR (501 MHz, chloroform-*d*, 298 K) spectrum of compound **9b**

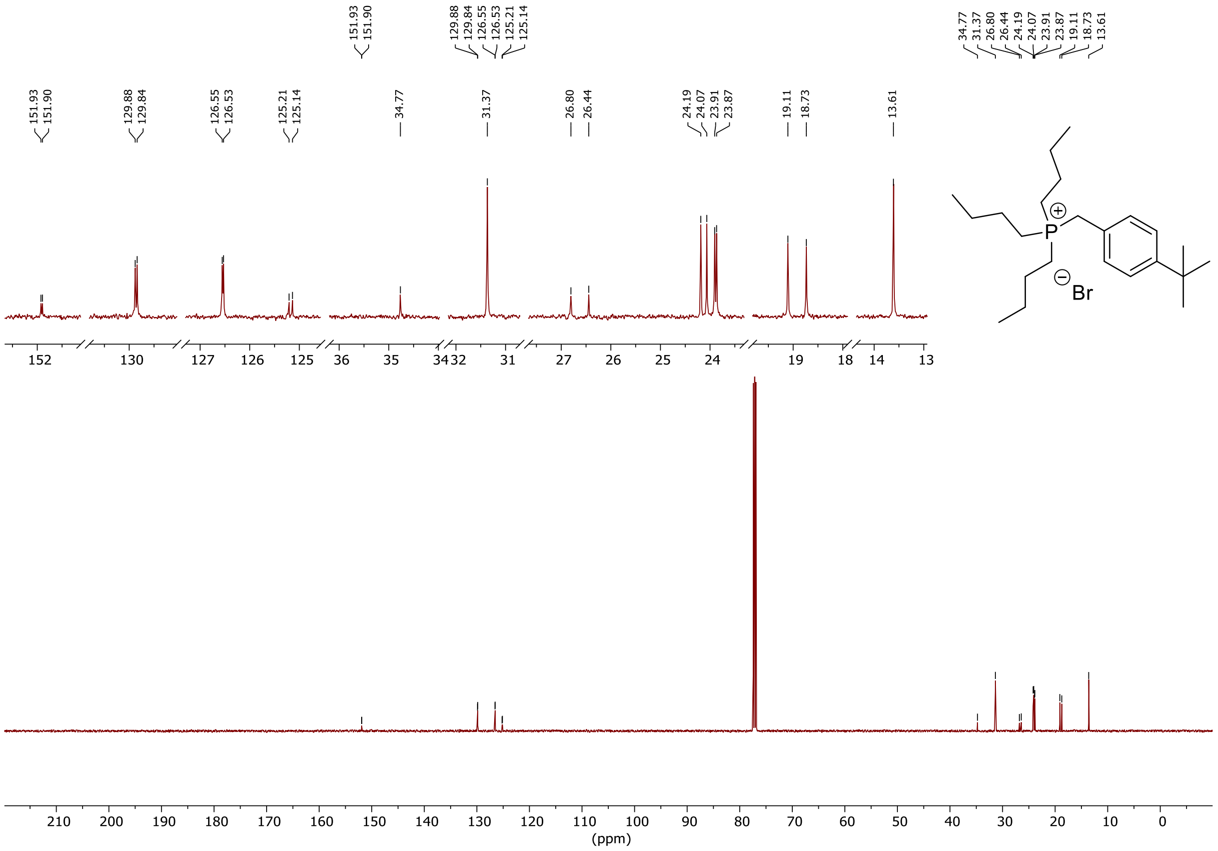

Fig. S220 $^{13}C\{^{1}H\}$ NMR (126 MHz, chloroform-*d*, 298 K) spectrum of compound **9b**

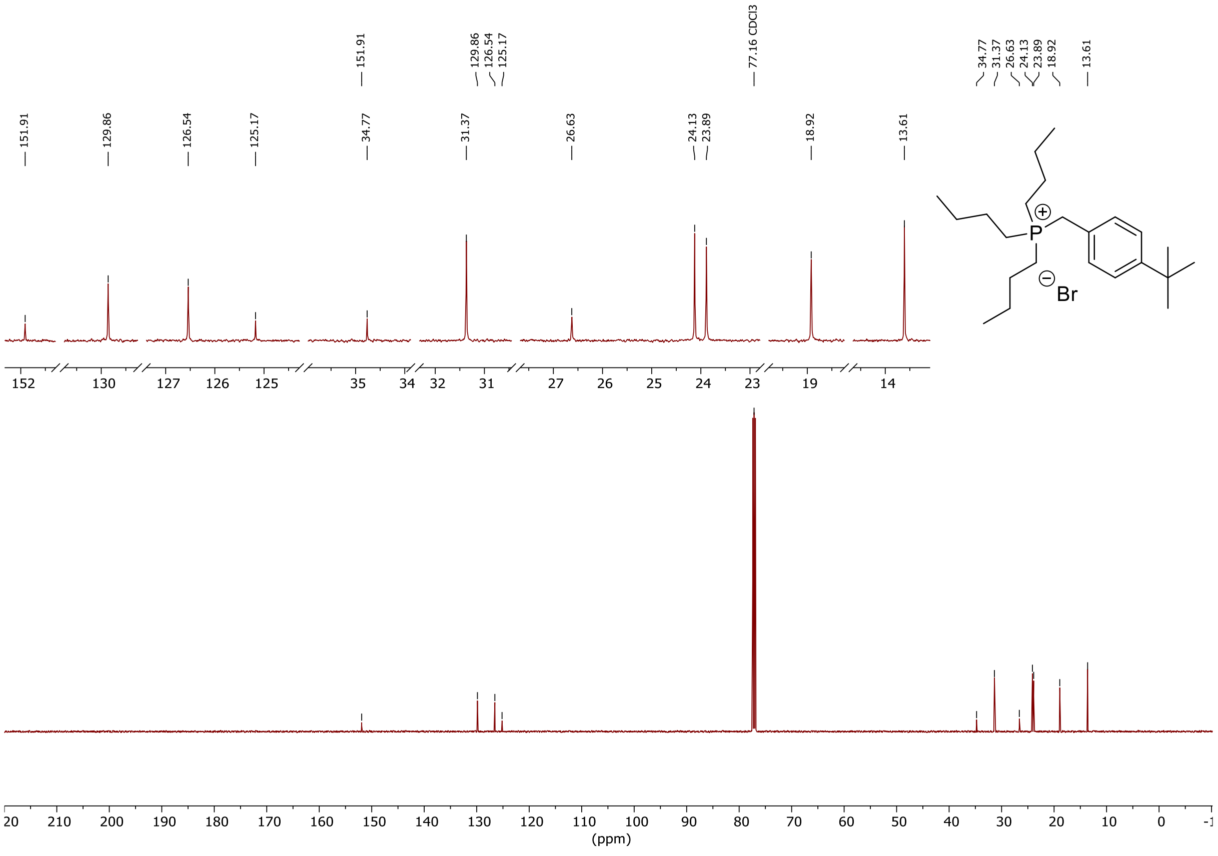

Fig. S221 $^{13}C\{^{1}H,^{31}P\}$ NMR (126 MHz, chloroform-*d*, 298 K) spectrum of compound **9b**

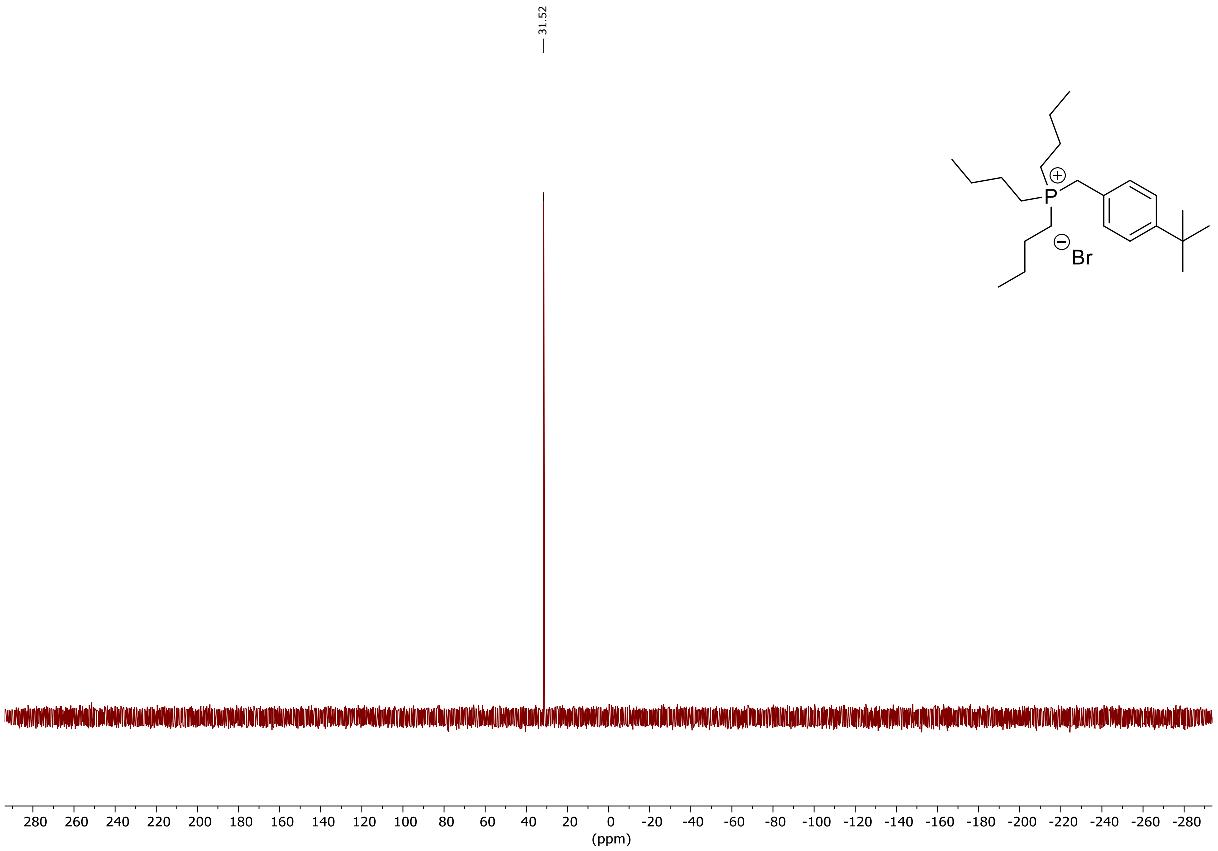

Fig. S222 ^{31}P NMR (203 MHz, chloroform-*d*, 298 K) spectrum of compound **9b**

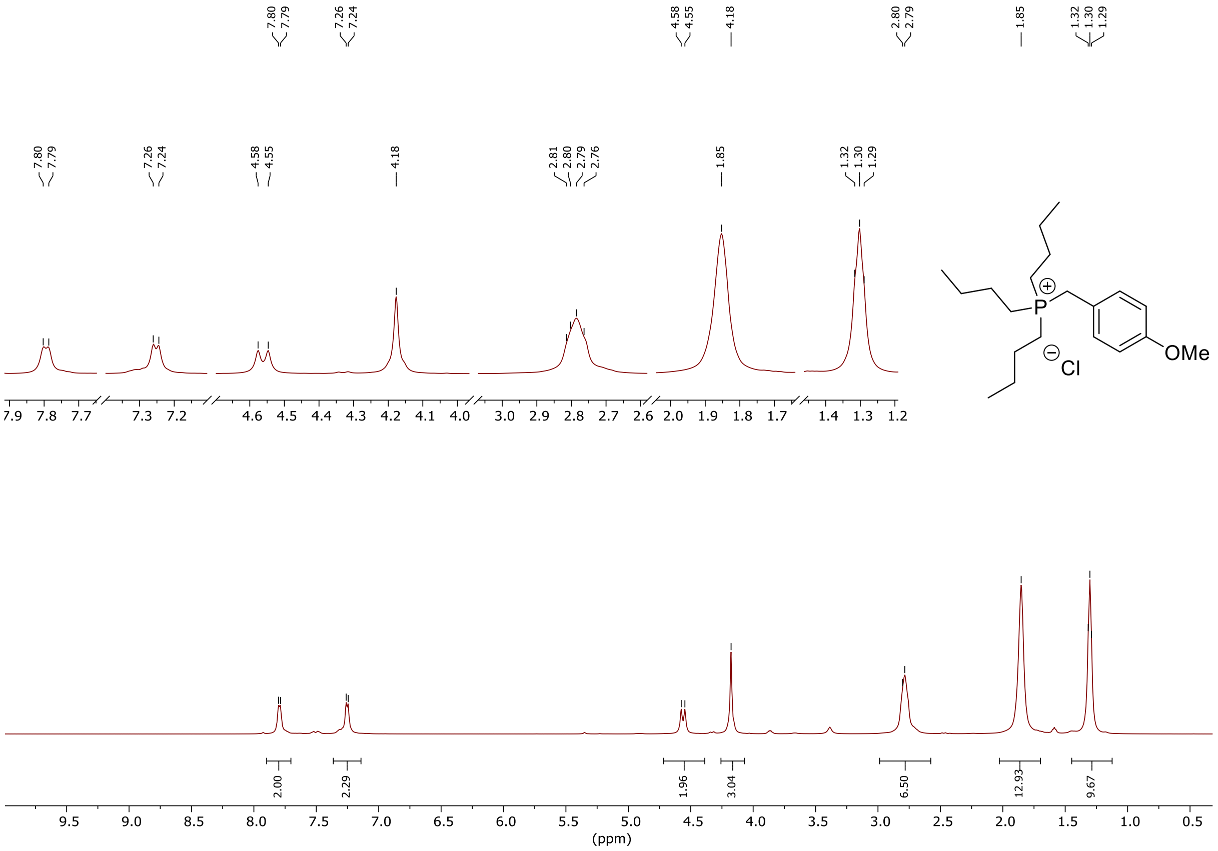

Fig. S223 ^{1}H NMR (501 MHz, chloroform-*d*, 298 K) spectrum of compound **9c**

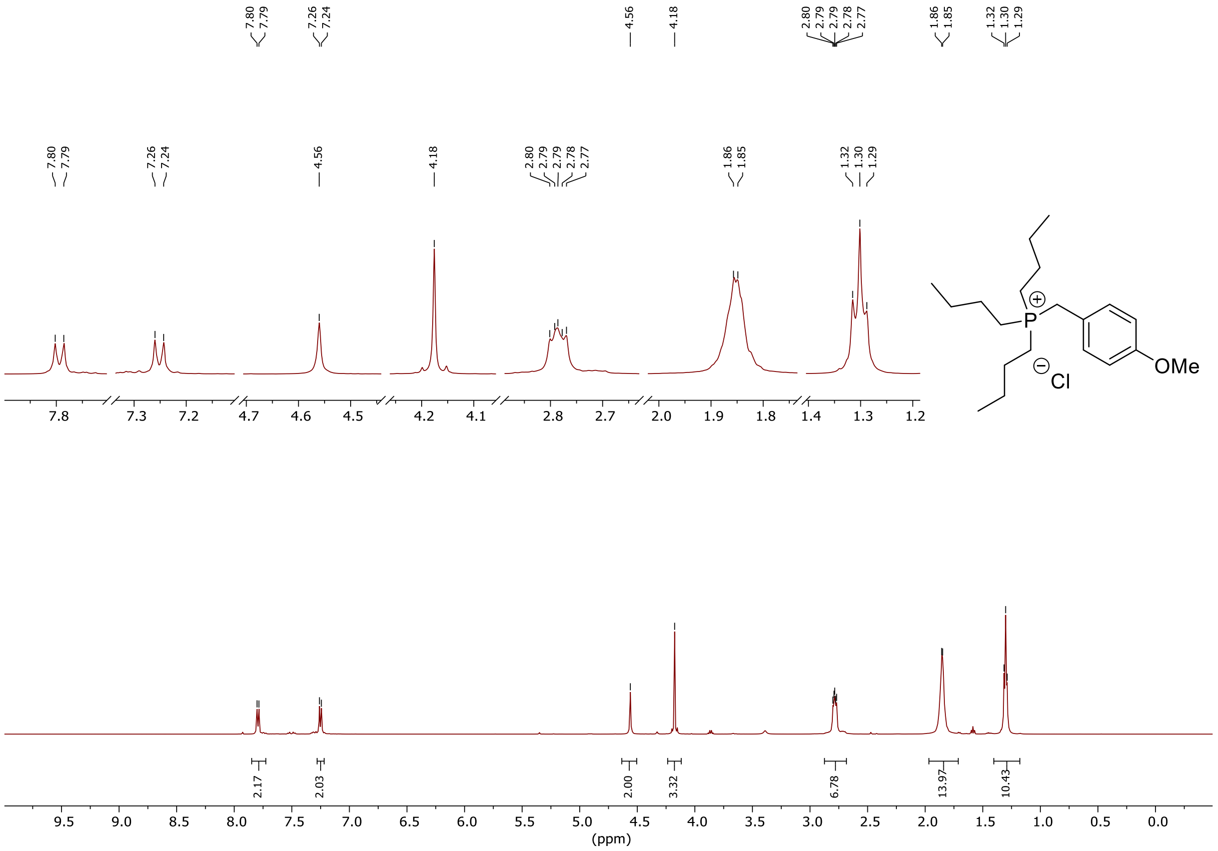

Fig. S224 ^{1}H{^{31}P} NMR (501 MHz, chloroform-*d*, 298 K) spectrum of compound **9c**

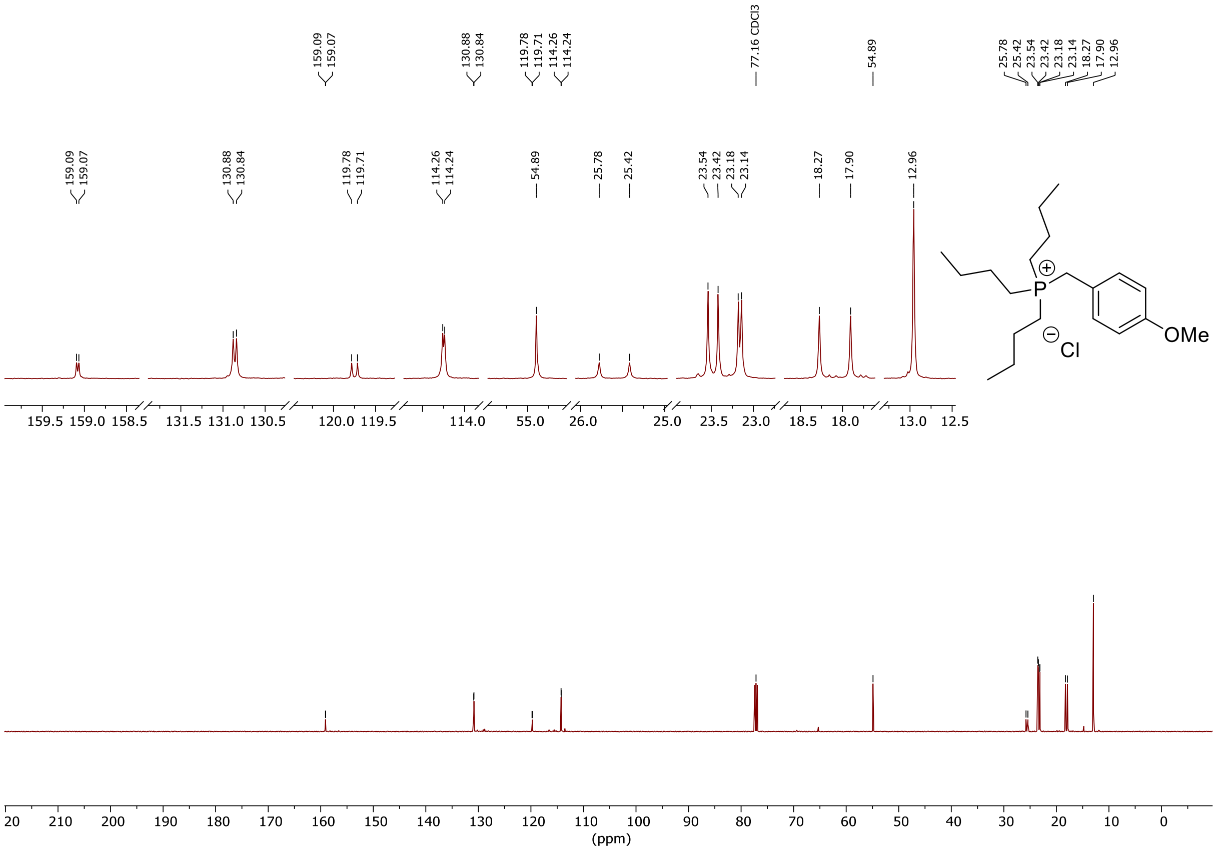

Fig. S225 $^{13}C\{^{1}H\}$ NMR (126 MHz, chloroform-*d*, 298 K) spectrum of compound **9c**

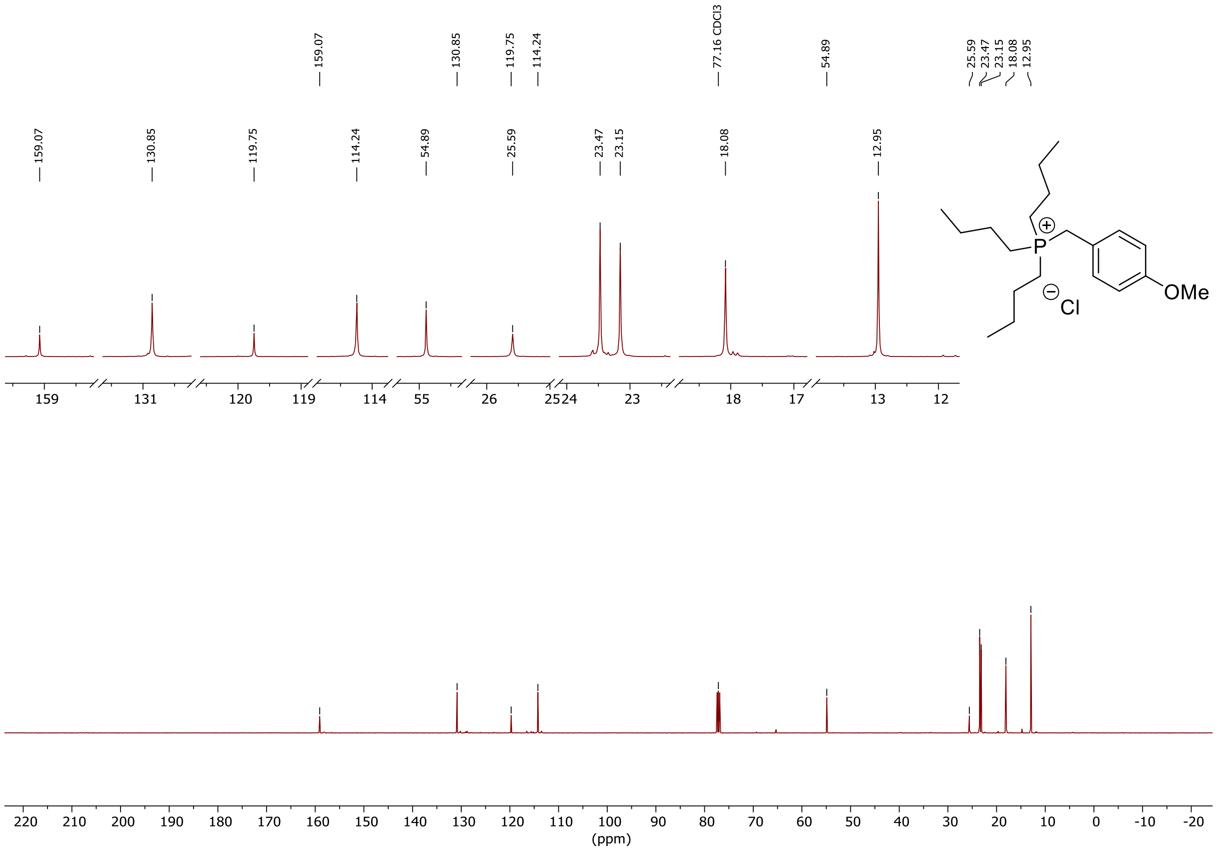

Fig. S226 $^{13}C\{^{1}H,^{31}P\}$ NMR (126 MHz, chloroform-*d*, 298 K) spectrum of compound **9c**

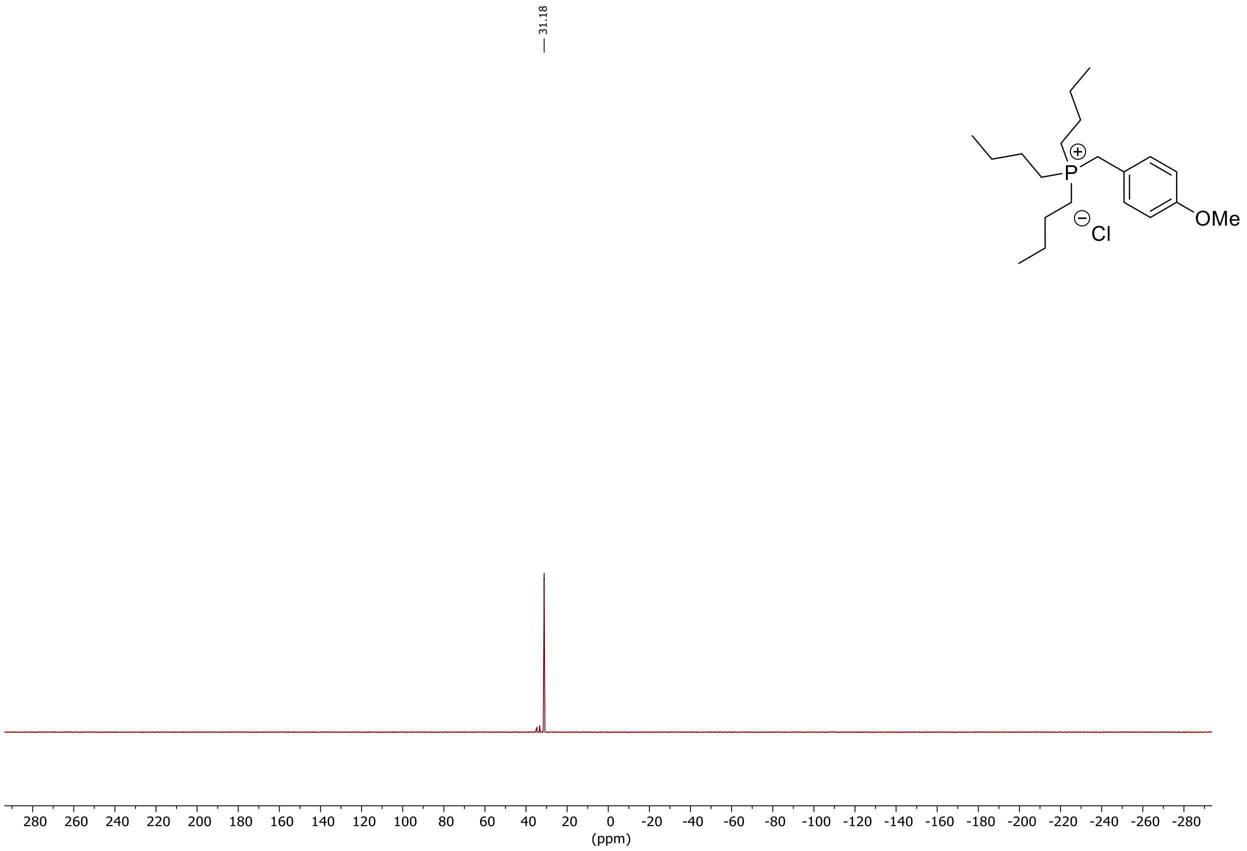

Fig. S227 ^{31}P NMR (203 MHz, chloroform-*d*, 298 K) spectrum of compound **9c**

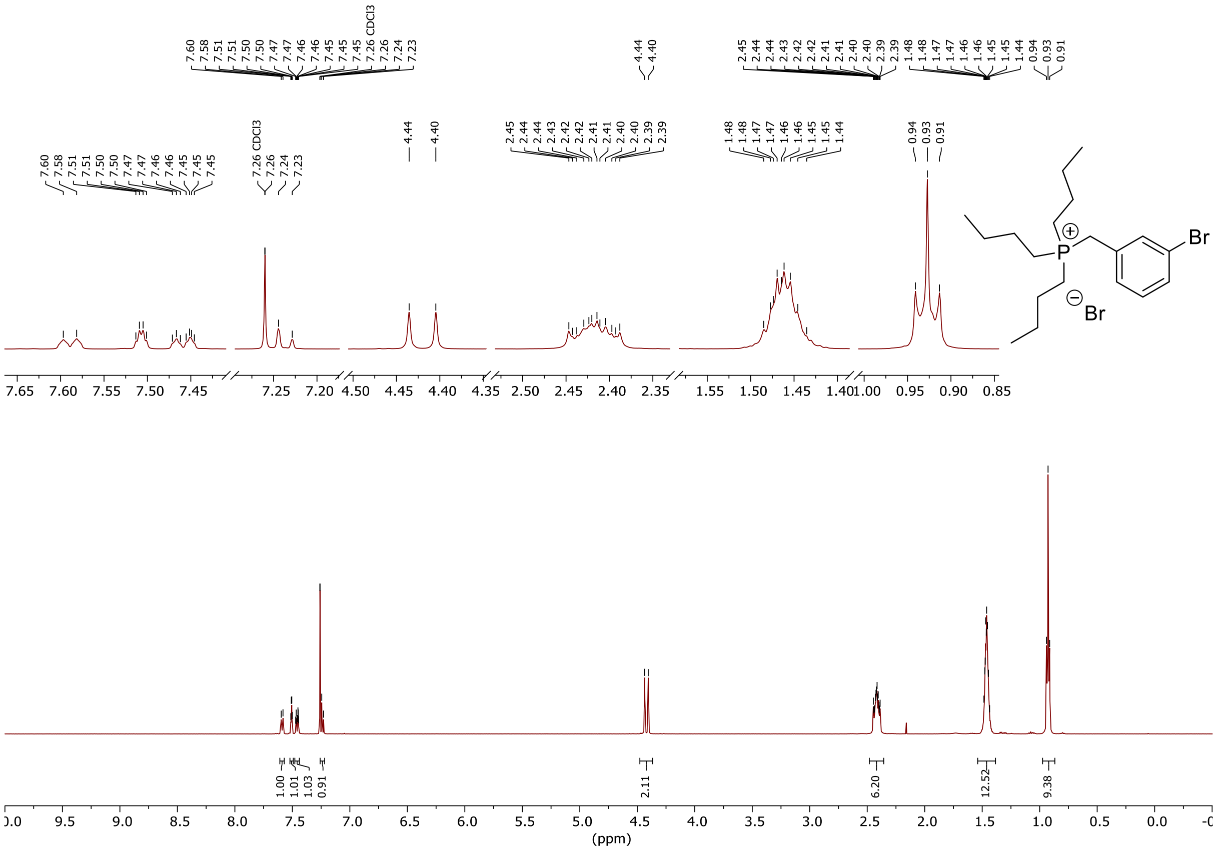

Fig. S228 ^{1}H NMR (501 MHz, chloroform-*d*, 298 K) spectrum of compound **9d**

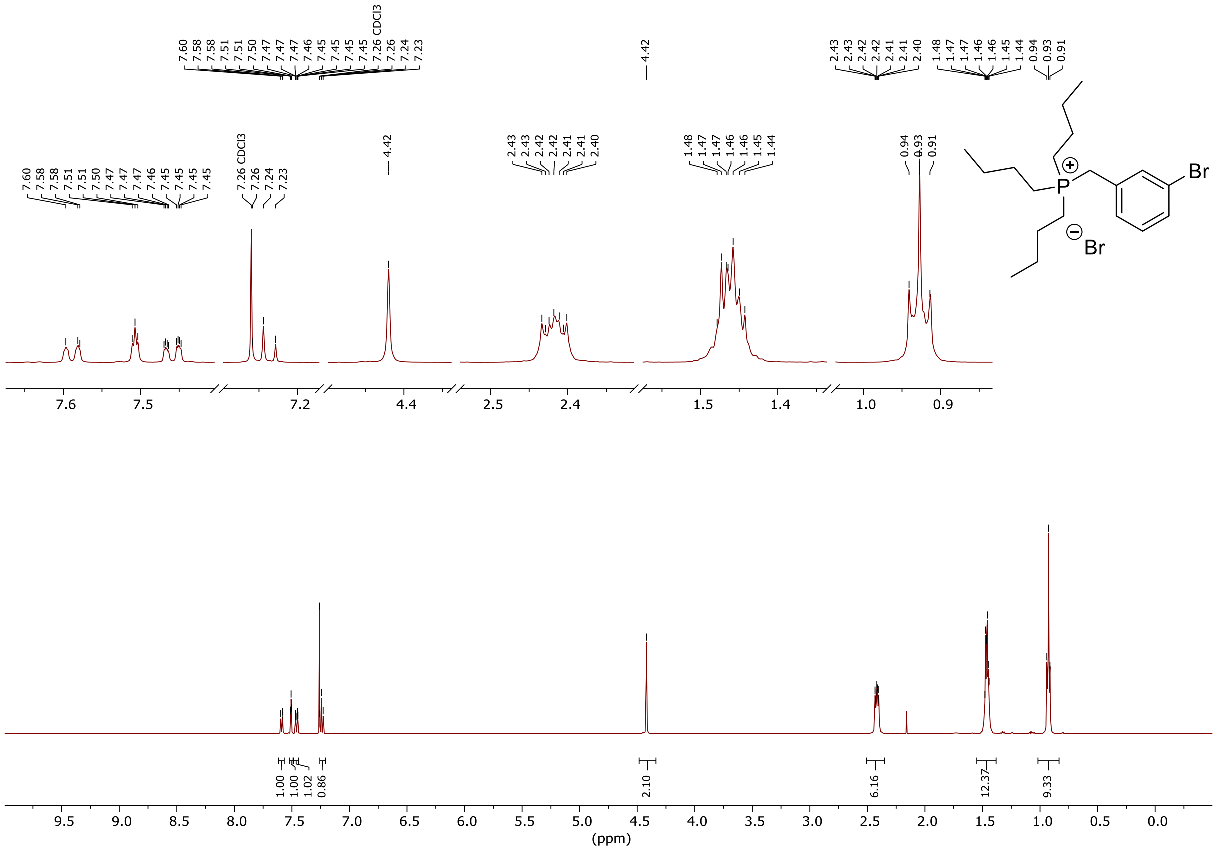

Fig. S229 ^{1}H{^{31}P} NMR (501 MHz, chloroform-*d*, 298 K) spectrum of compound **9d**

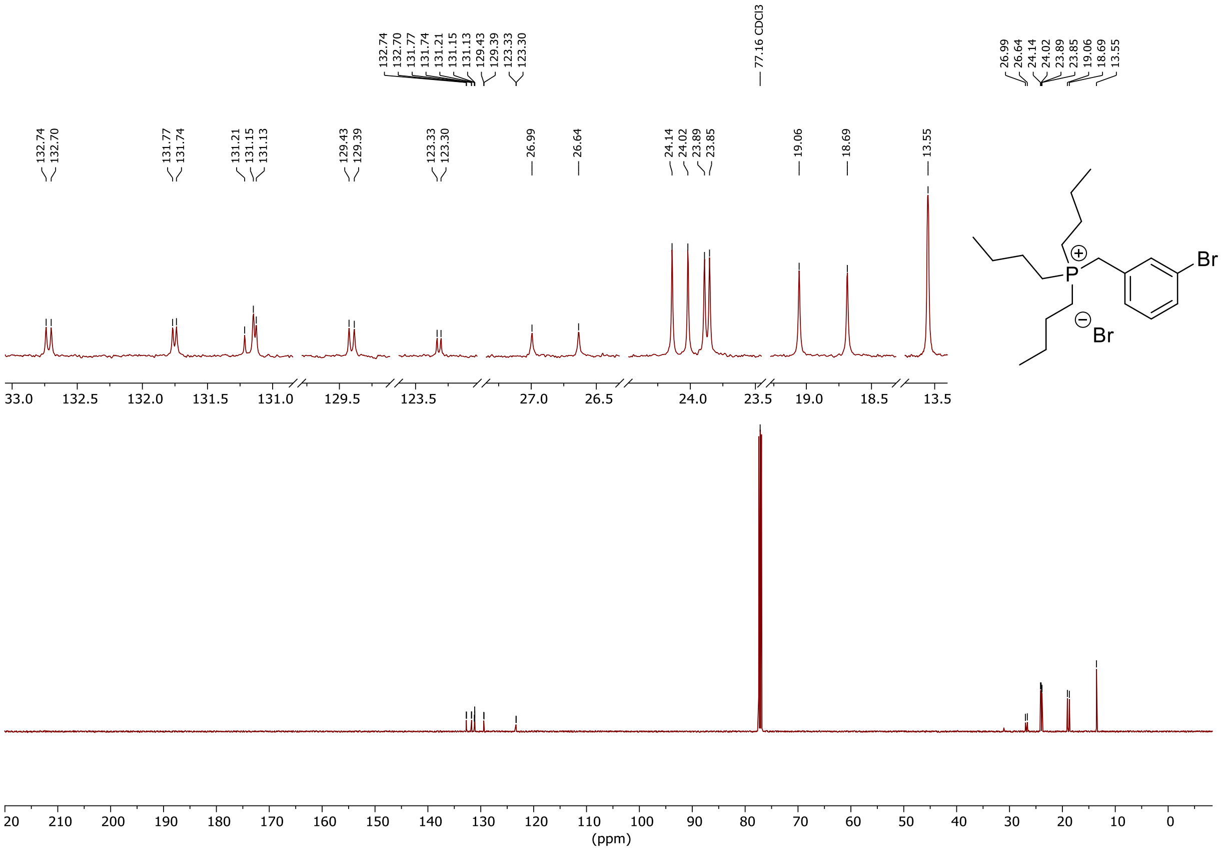

Fig. S230 $^{13}C\{^1H\}$ NMR (126 MHz, chloroform-*d*, 298 K) spectrum of compound **9d**

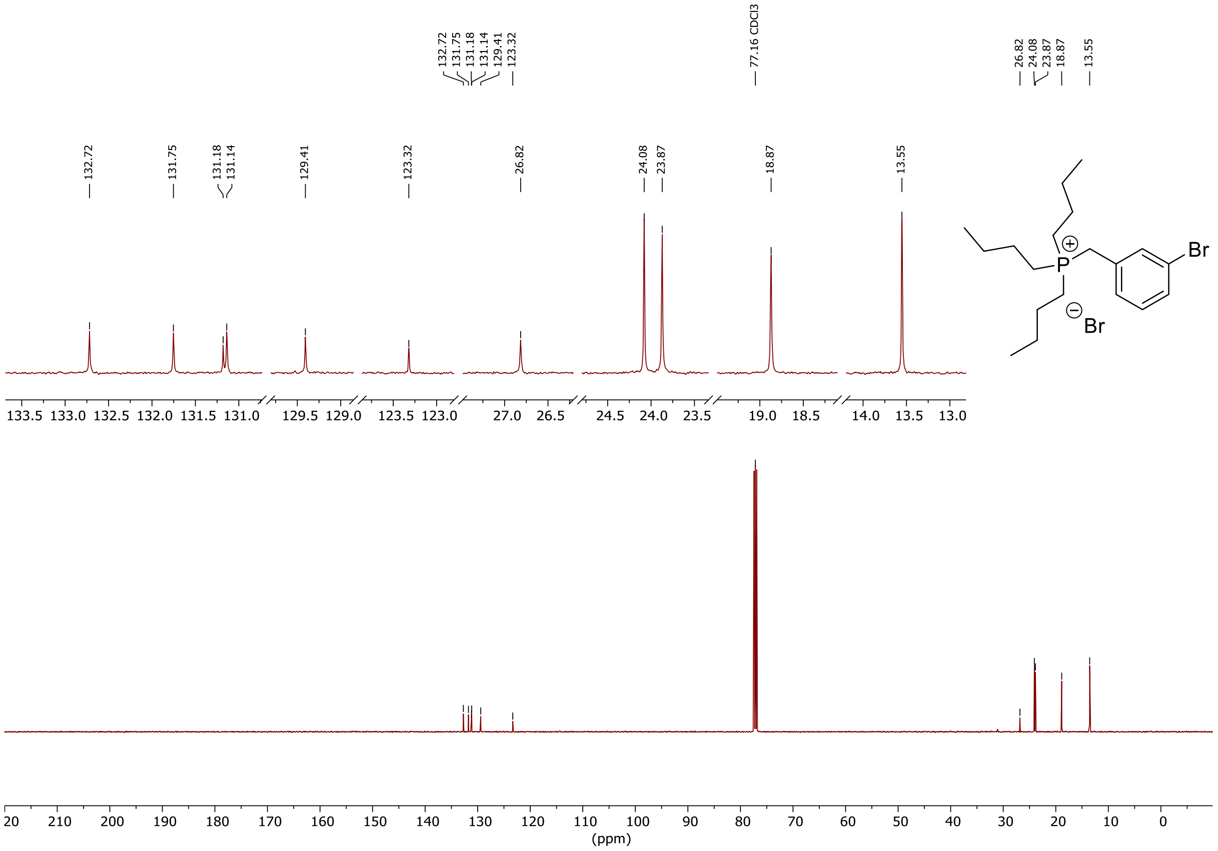

Fig. S231 $^{13}C\{^1H,^{31}P\}$ NMR (126 MHz, chloroform-*d*, 298 K) spectrum of compound **9d**

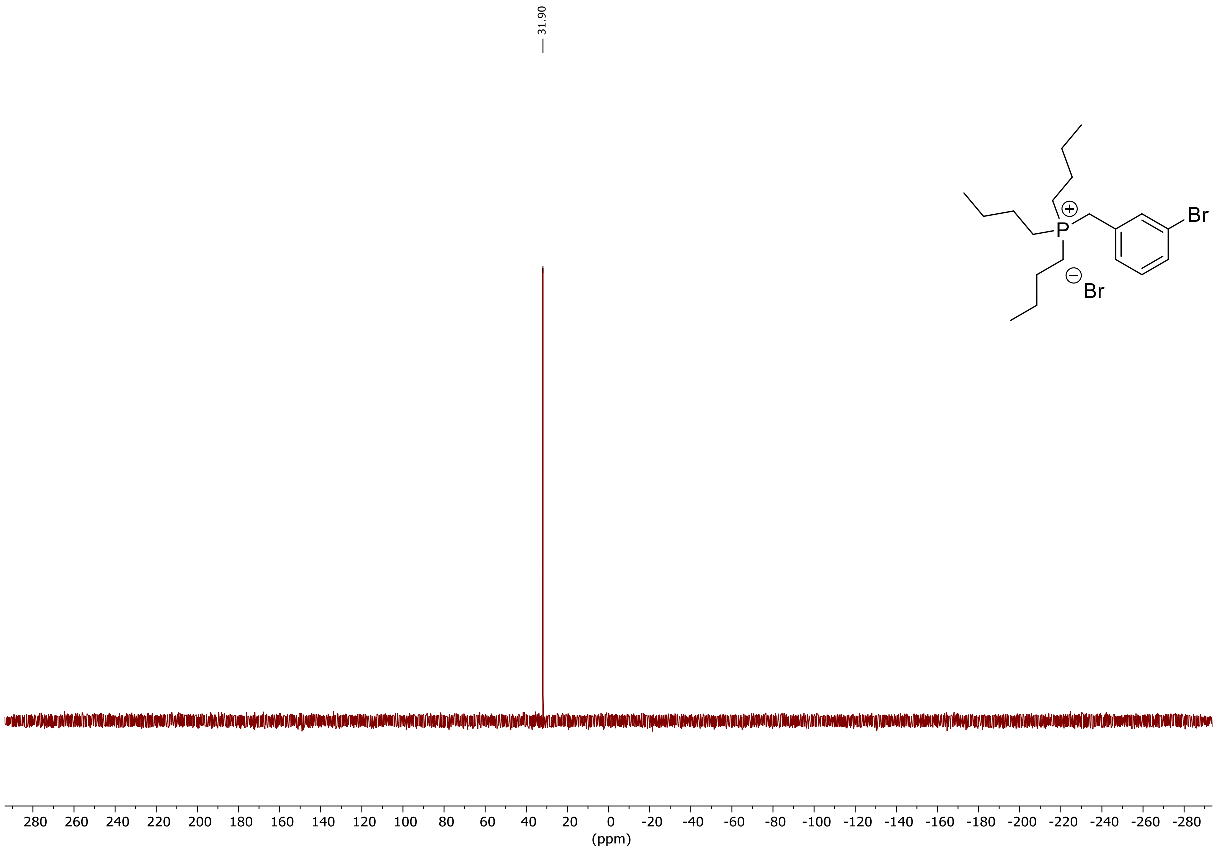

Fig. S232 ^{31}P NMR (203 MHz, chloroform-*d*, 298 K) spectrum of compound **9d**

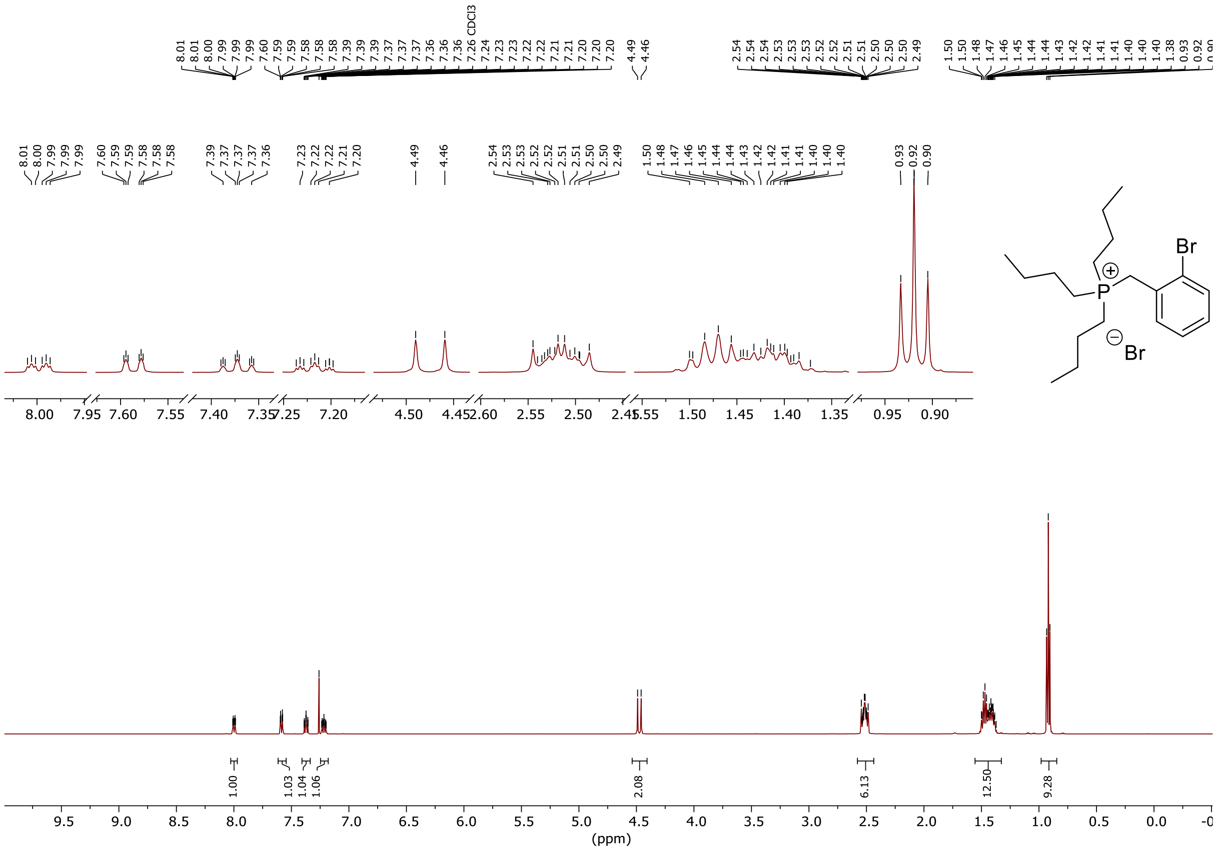

Fig. S233 ^{1}H NMR (501 MHz, chloroform-*d*, 298 K) spectrum of compound **9e**

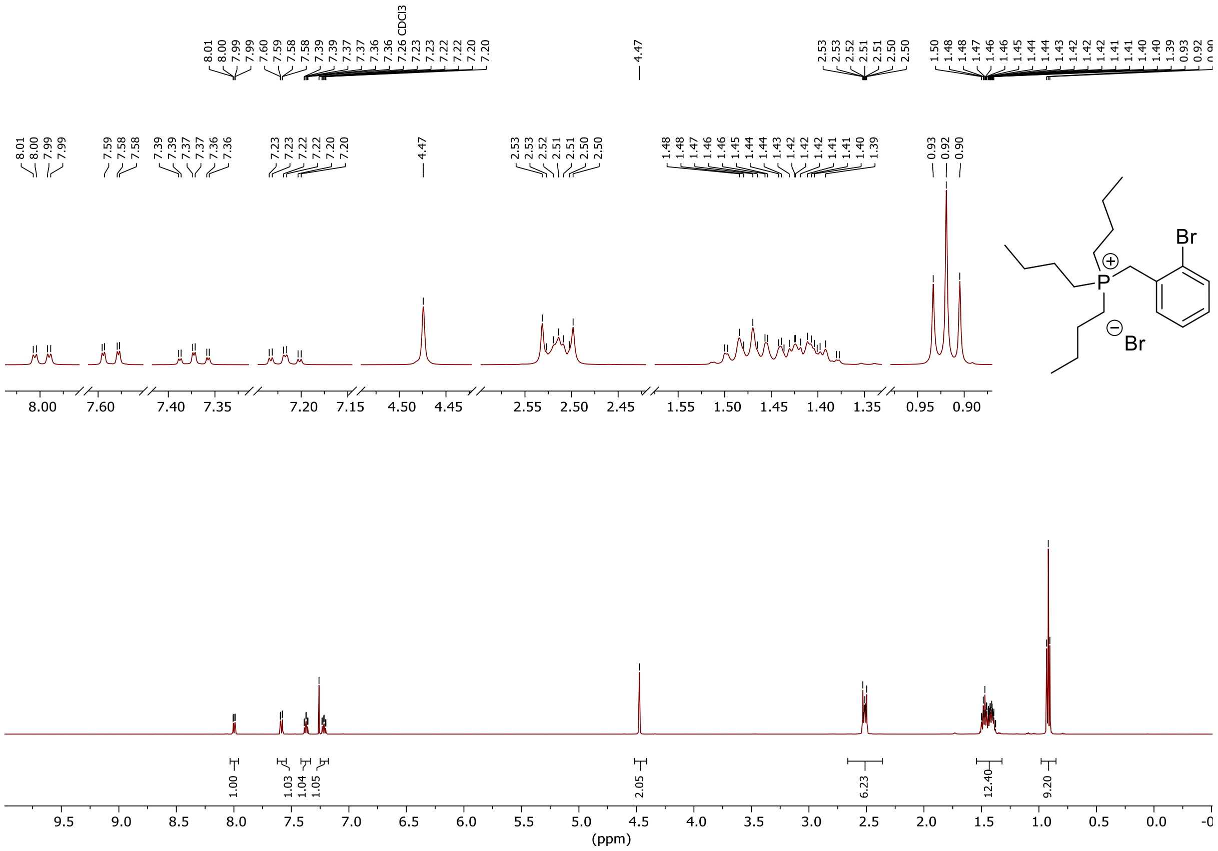

Fig. S234 $^{1}H\{^{31}P\}$ NMR (501 MHz, chloroform-*d*, 298 K) spectrum of compound **9e**

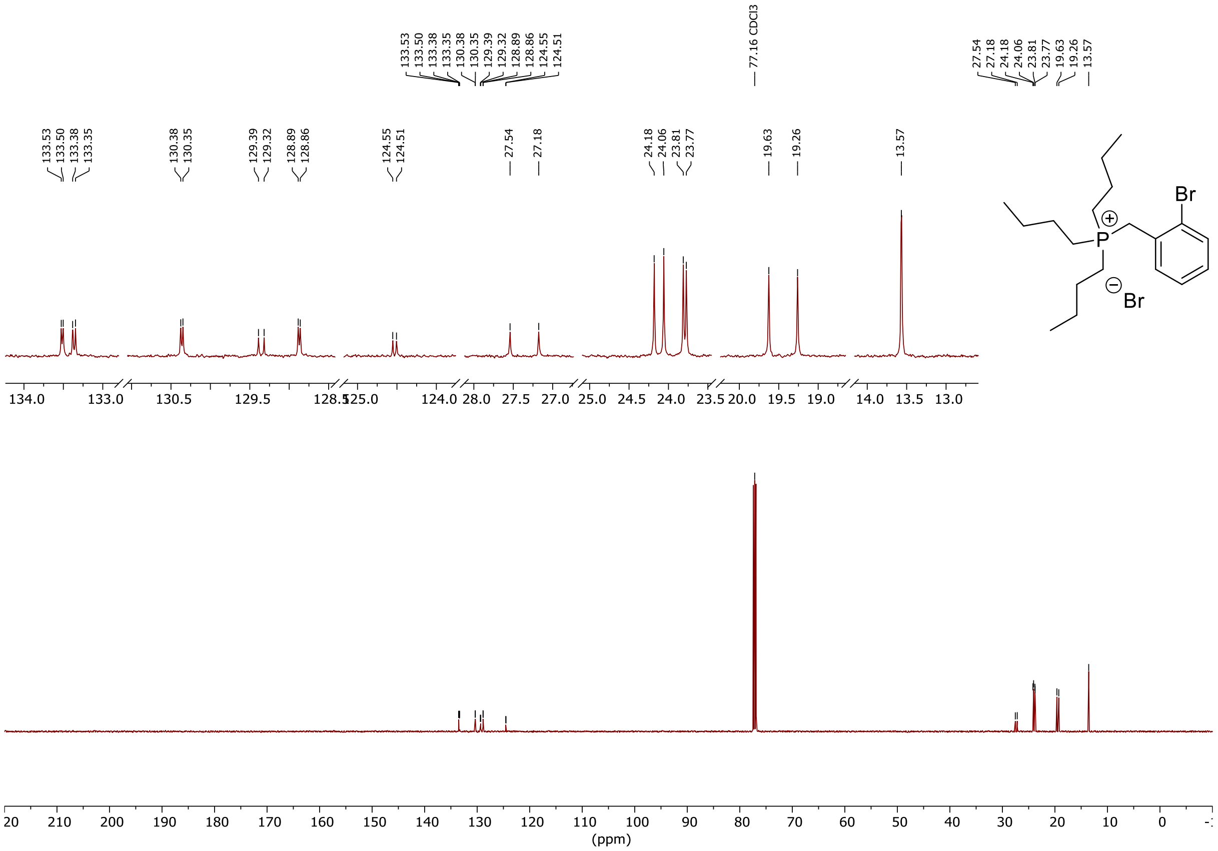

Fig. S235 $^{13}C\{^{1}H\}$ NMR (126 MHz, chloroform-*d*, 298 K) spectrum of compound **9e**

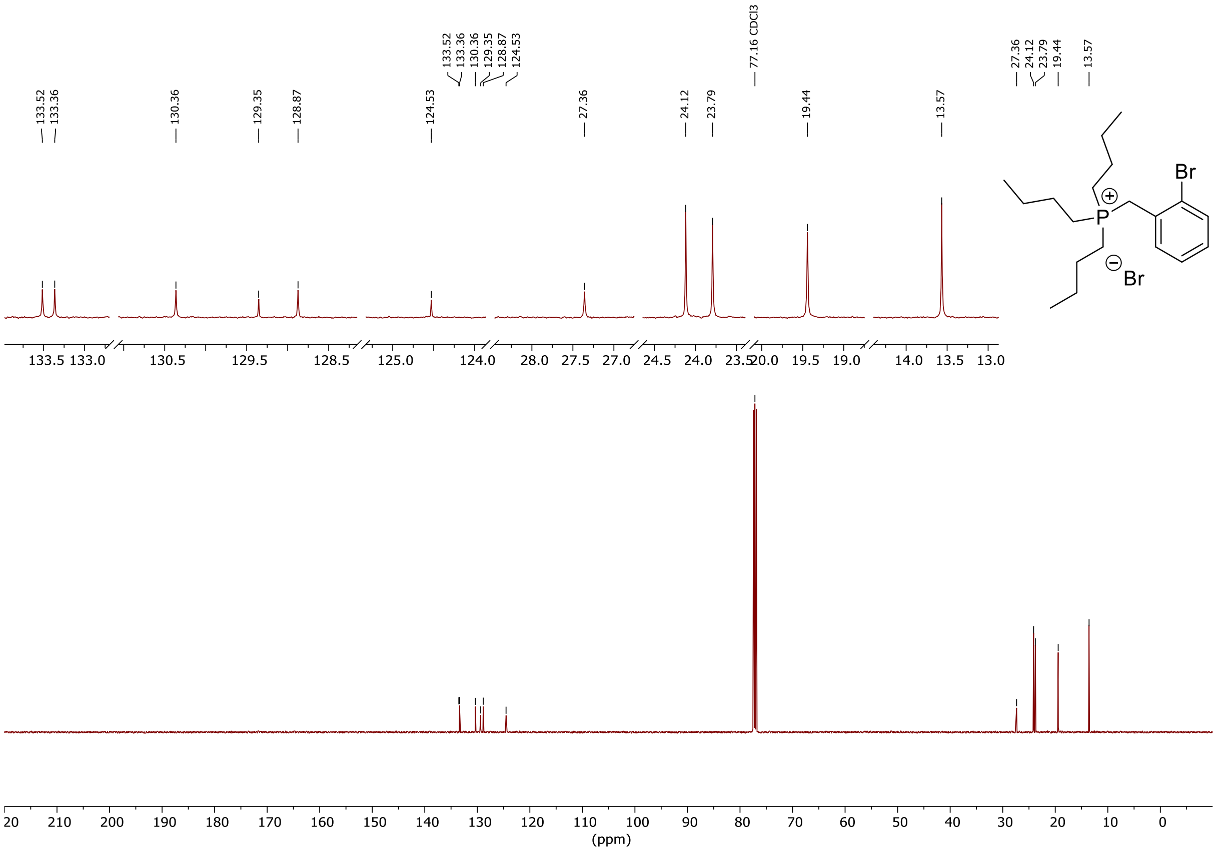

Fig. S236 $^{13}C\{^{1}H,^{31}P\}$ NMR (126 MHz, chloroform-*d*, 298 K) spectrum of compound **9e**

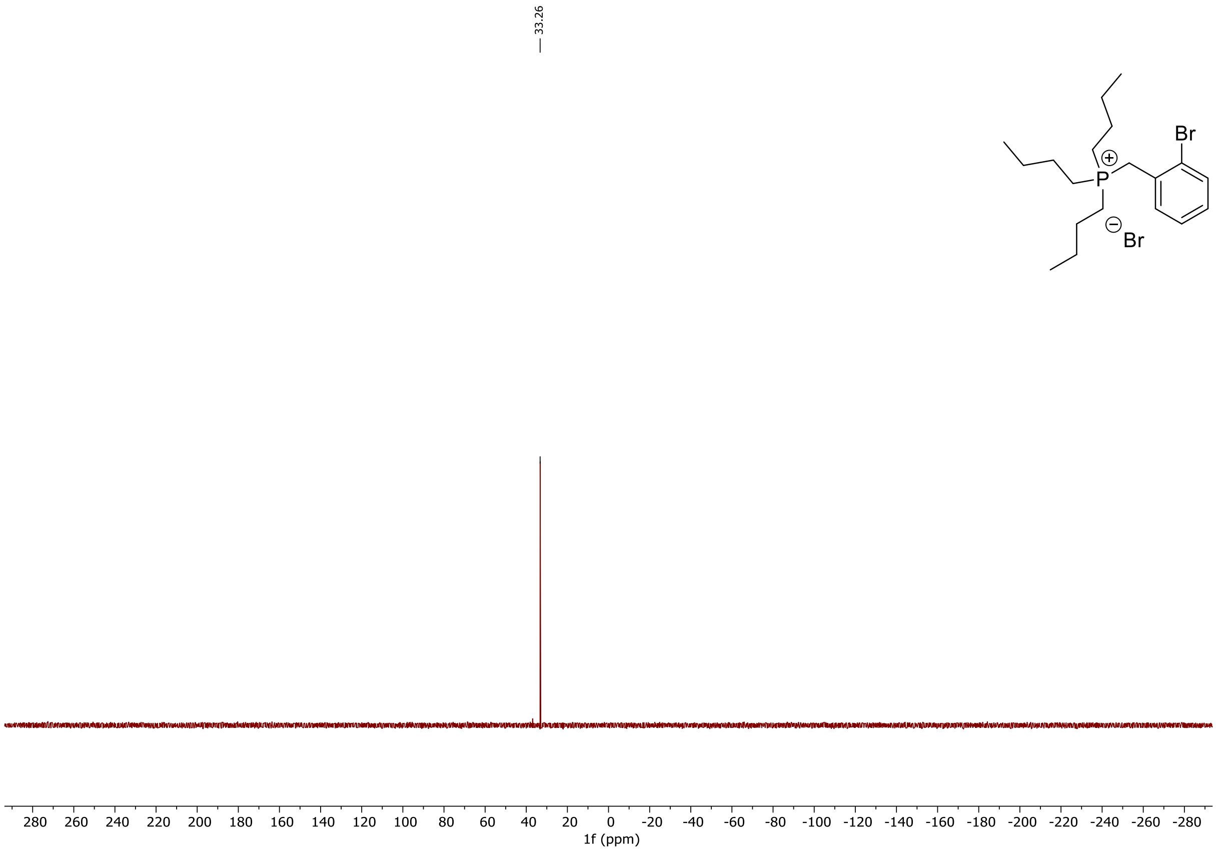

Fig. S237 ^{31}P NMR (203 MHz, chloroform-*d*, 298 K) spectrum of compound **9e**

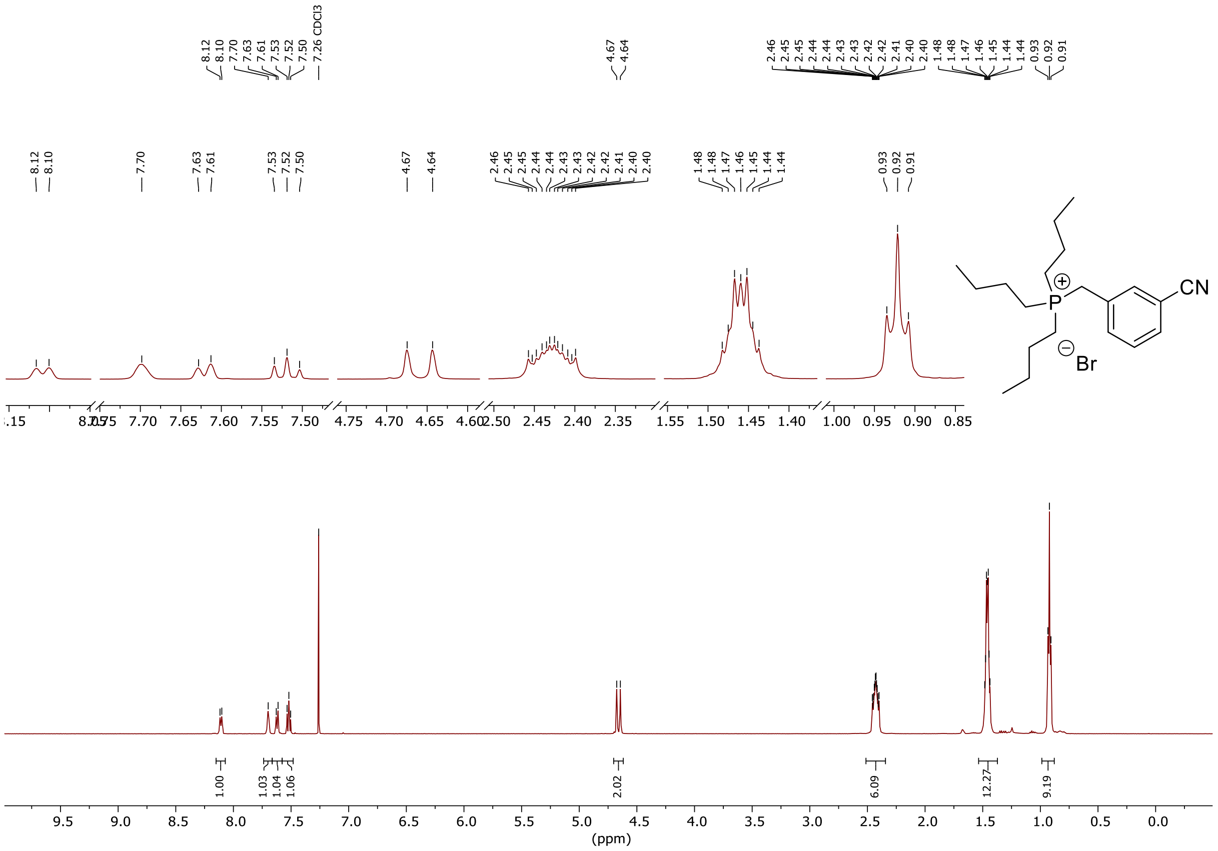

Fig. S238 ^{1}H NMR (501 MHz, chloroform-*d*, 298 K) spectrum of compound **9f**

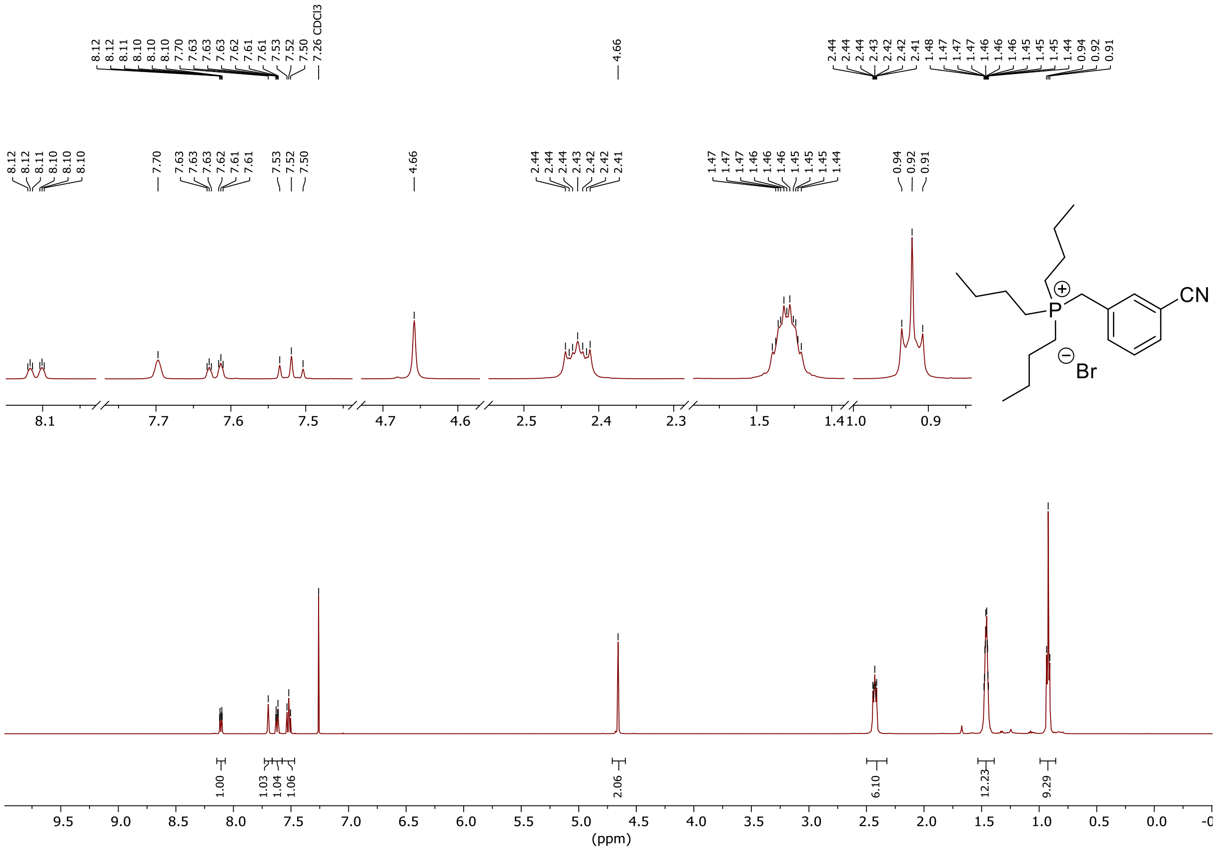

Fig. S239 ^{1}H{^{31}P} NMR (501 MHz, chloroform-*d*, 298 K) spectrum of compound **9f**

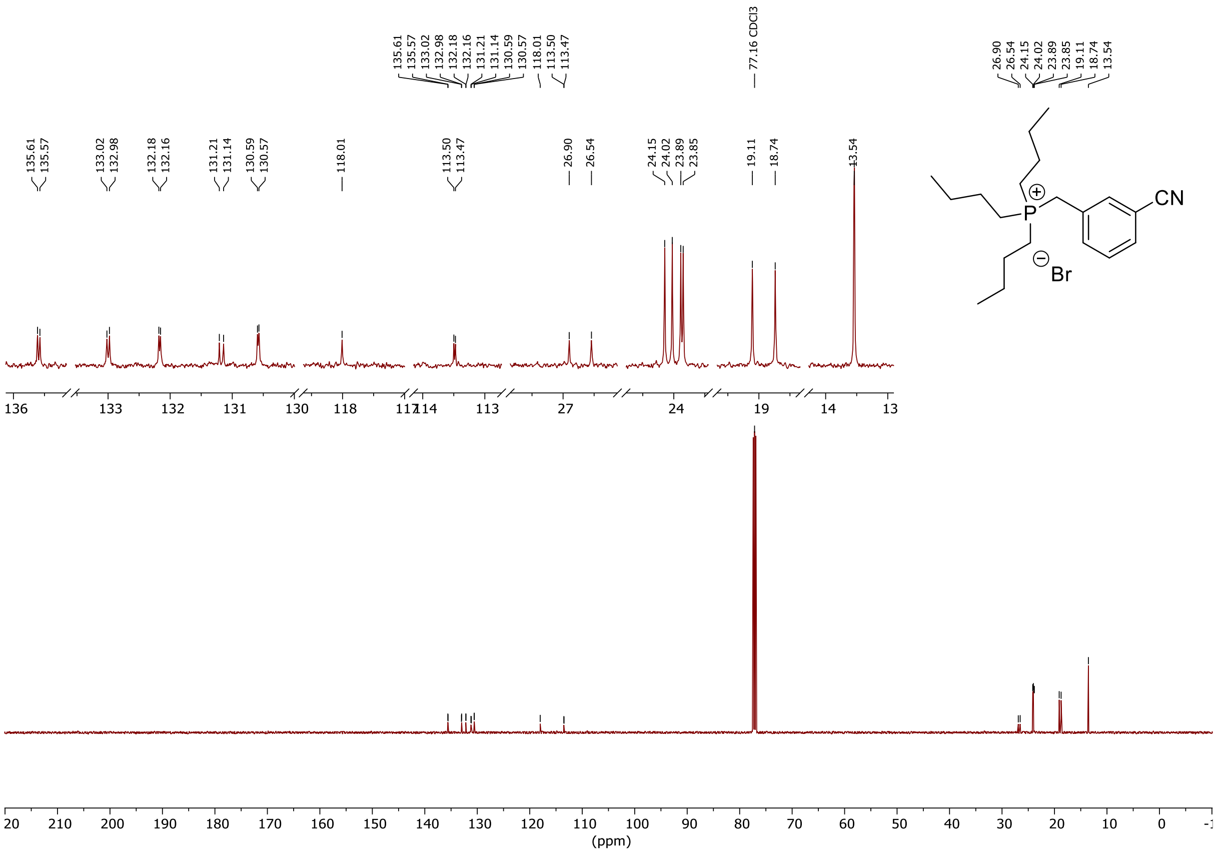

Fig. S240 $^{13}C\{^{1}H\}$ NMR (126 MHz, chloroform-*d*, 298 K) spectrum of compound **9f**

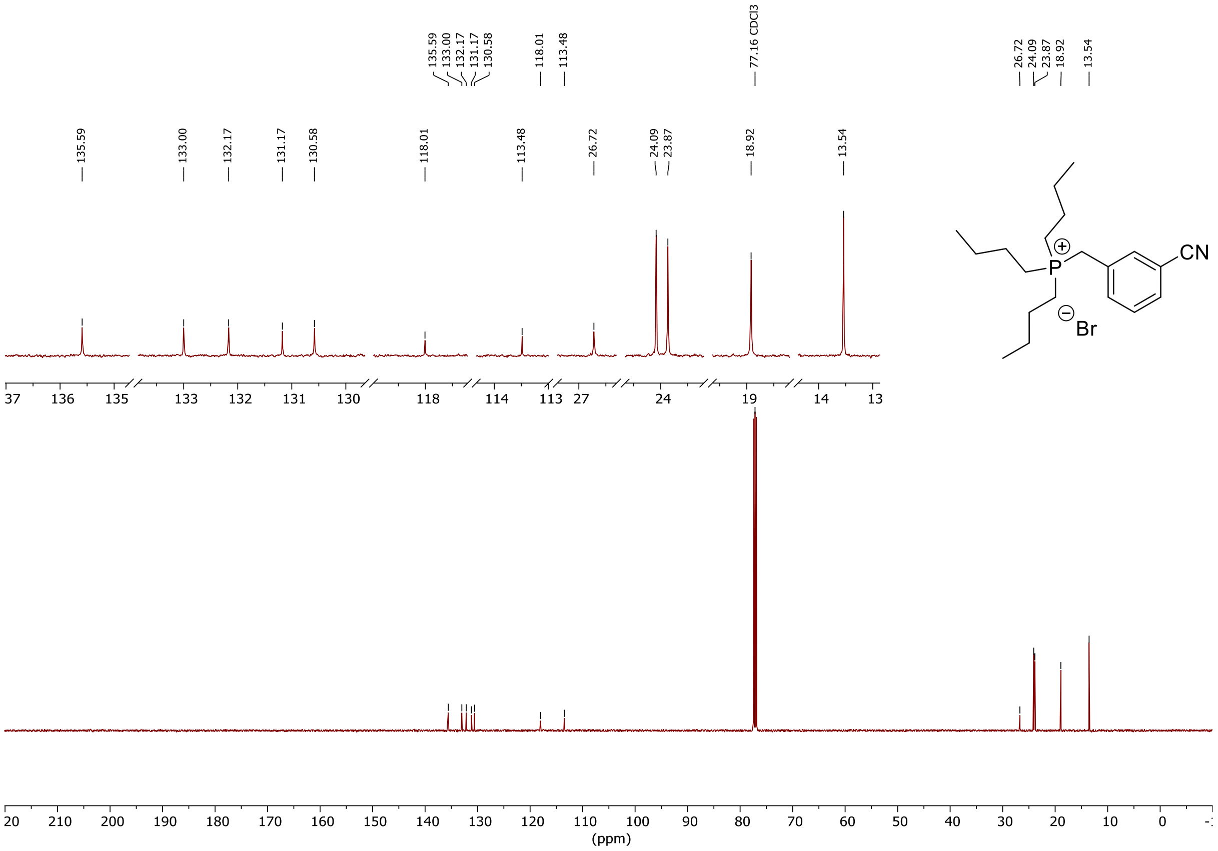

Fig. S241 $^{13}C\{^{1}H,^{31}P\}$ NMR (126 MHz, chloroform-*d*, 298 K) spectrum of compound **9f**

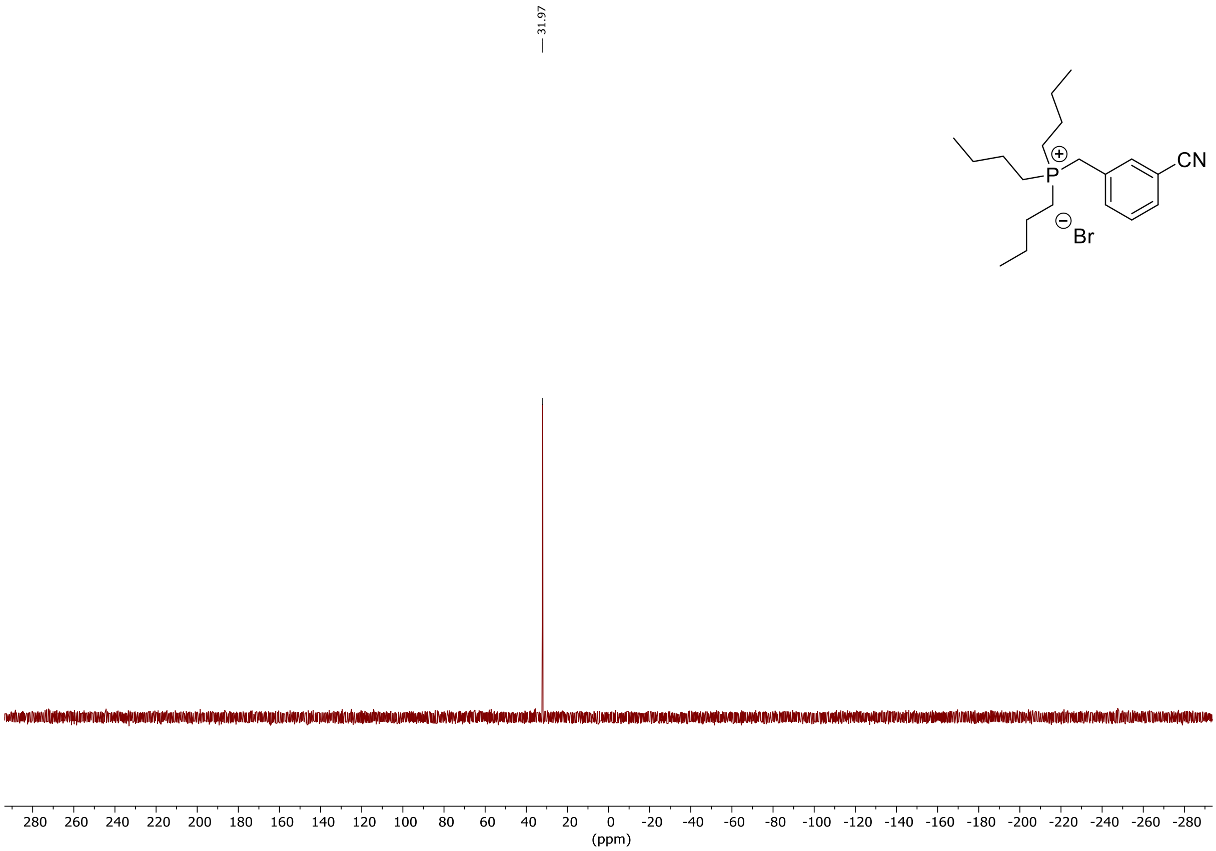

Fig. S242 ^{31}P NMR (203 MHz, chloroform-*d*, 298 K) spectrum of compound **9f**

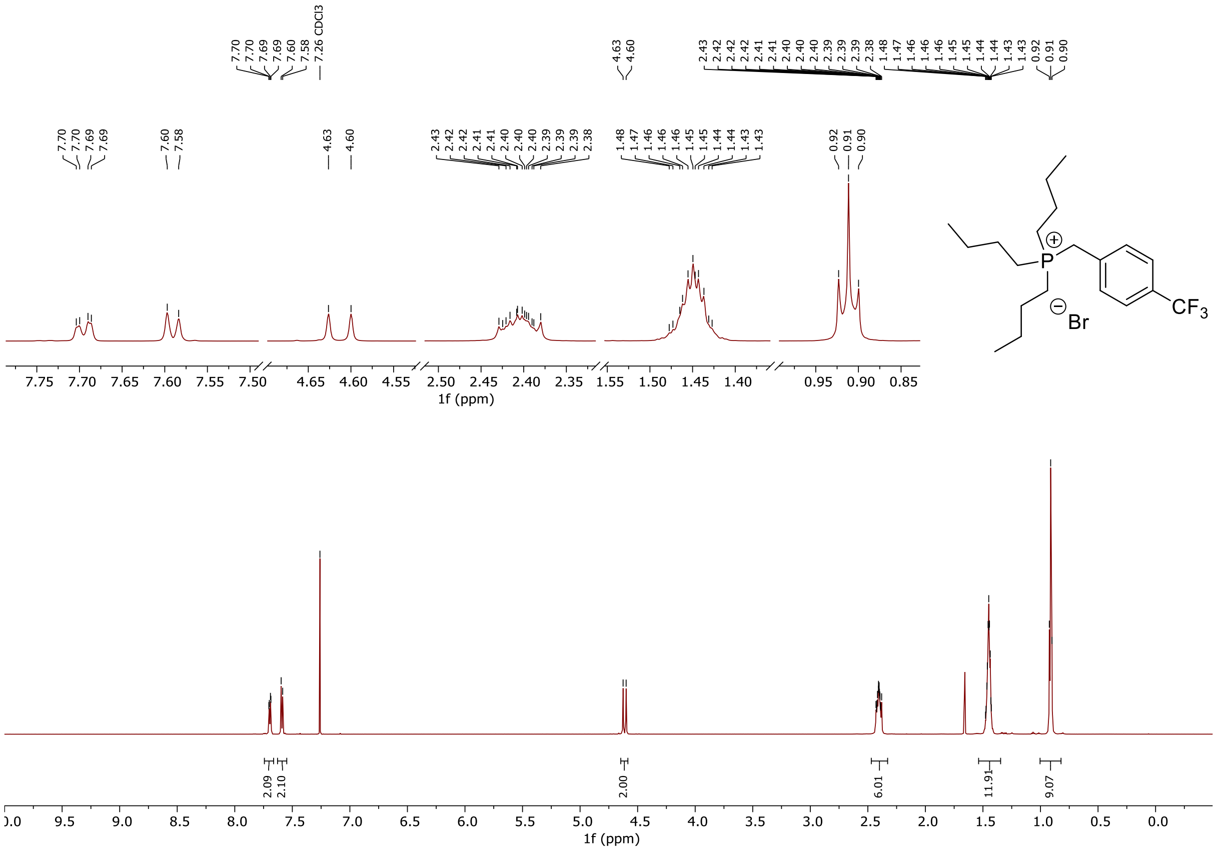

Fig. S243 ^{1}H NMR (501 MHz, chloroform-*d*, 298 K) spectrum of compound **9g**

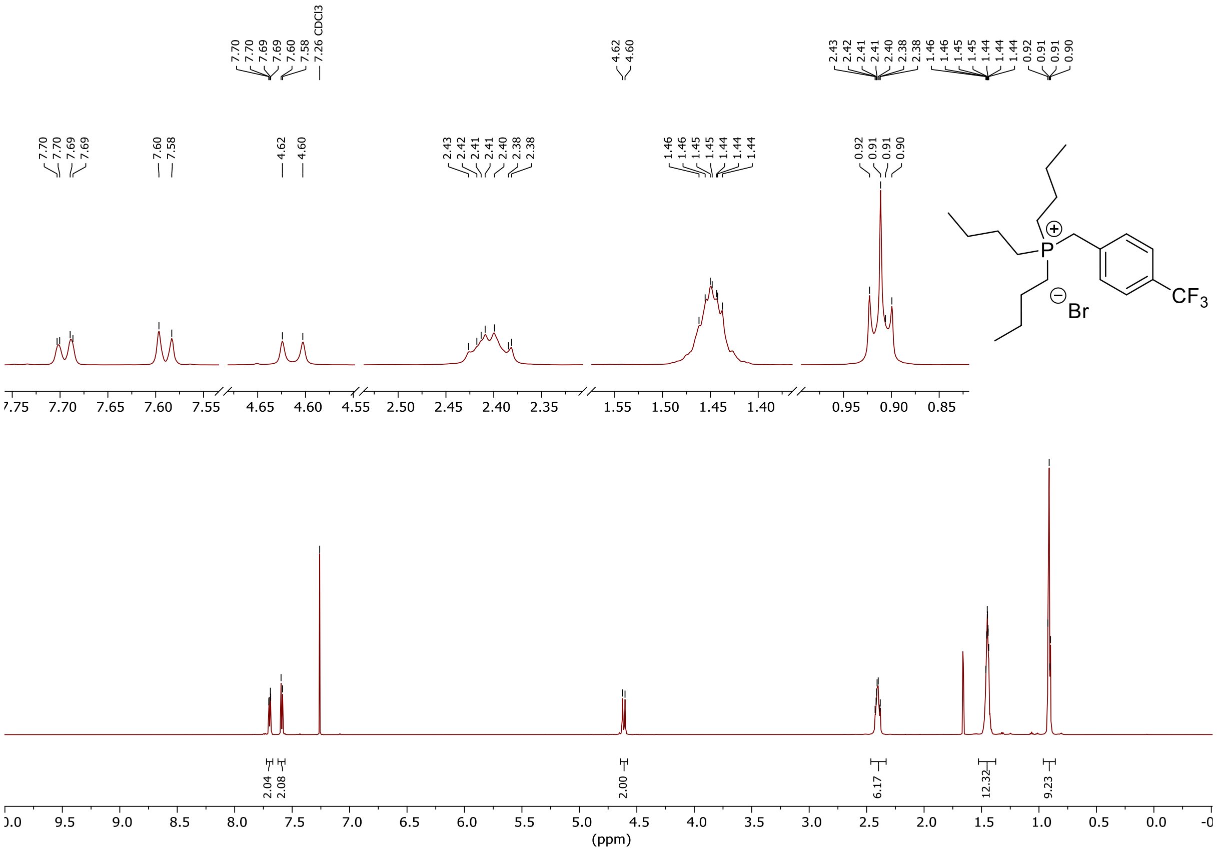

Fig. S244 $^{1}H\{^{31}P\}$ NMR (501 MHz, chloroform-*d*, 298 K) spectrum of compound **9g**

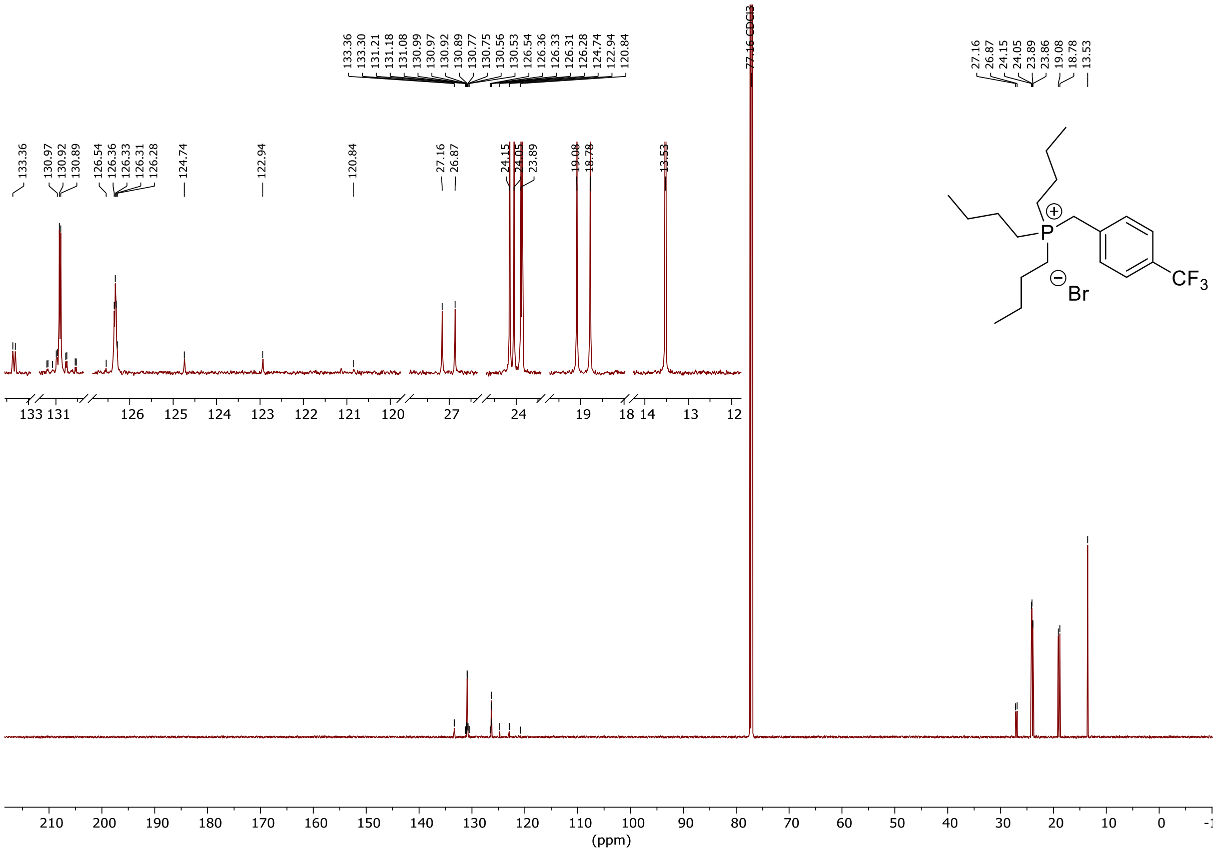

Fig. S245 $^{13}C\{^{1}H\}$ NMR (126 MHz, chloroform-*d*, 298 K) spectrum of compound **9g**

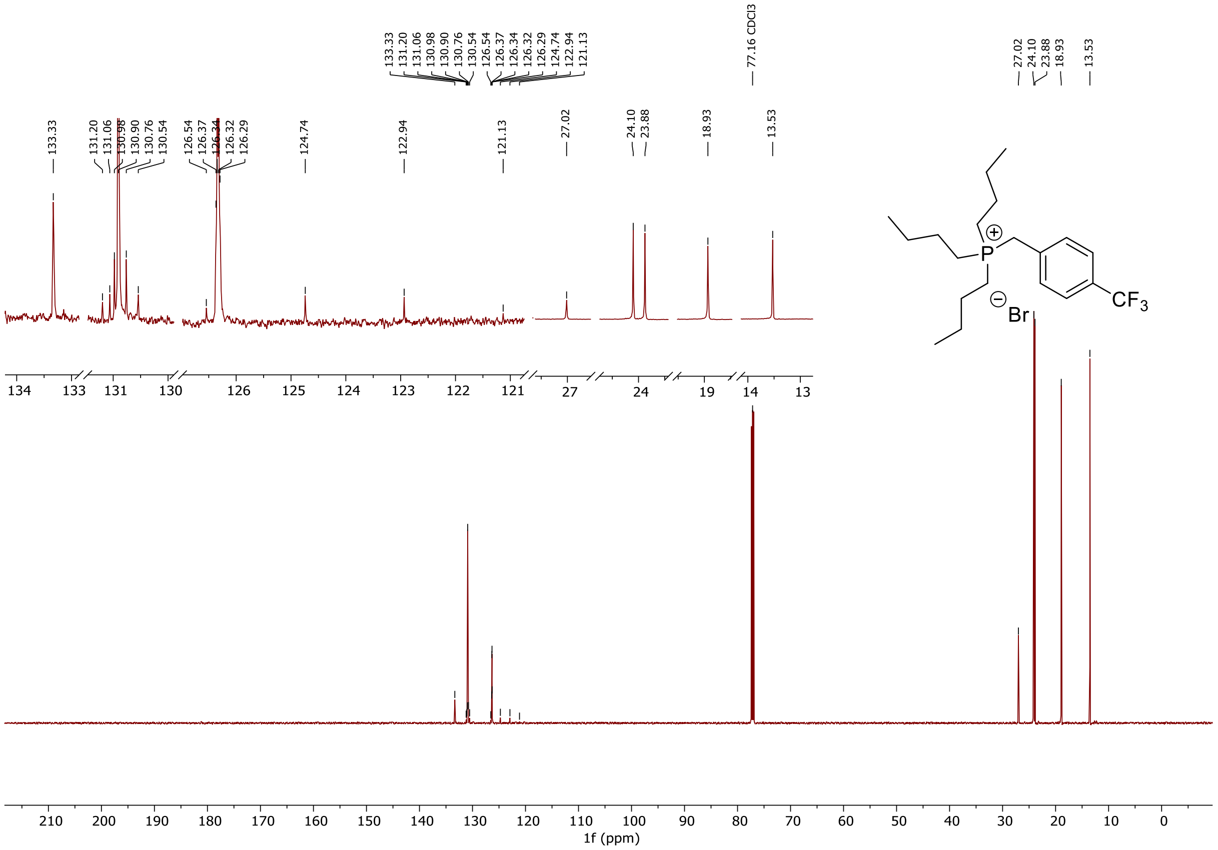

Fig. S246 $^{13}C\{^{1}H,^{31}P\}$ NMR (126 MHz, chloroform-*d*, 298 K) spectrum of compound **9g**

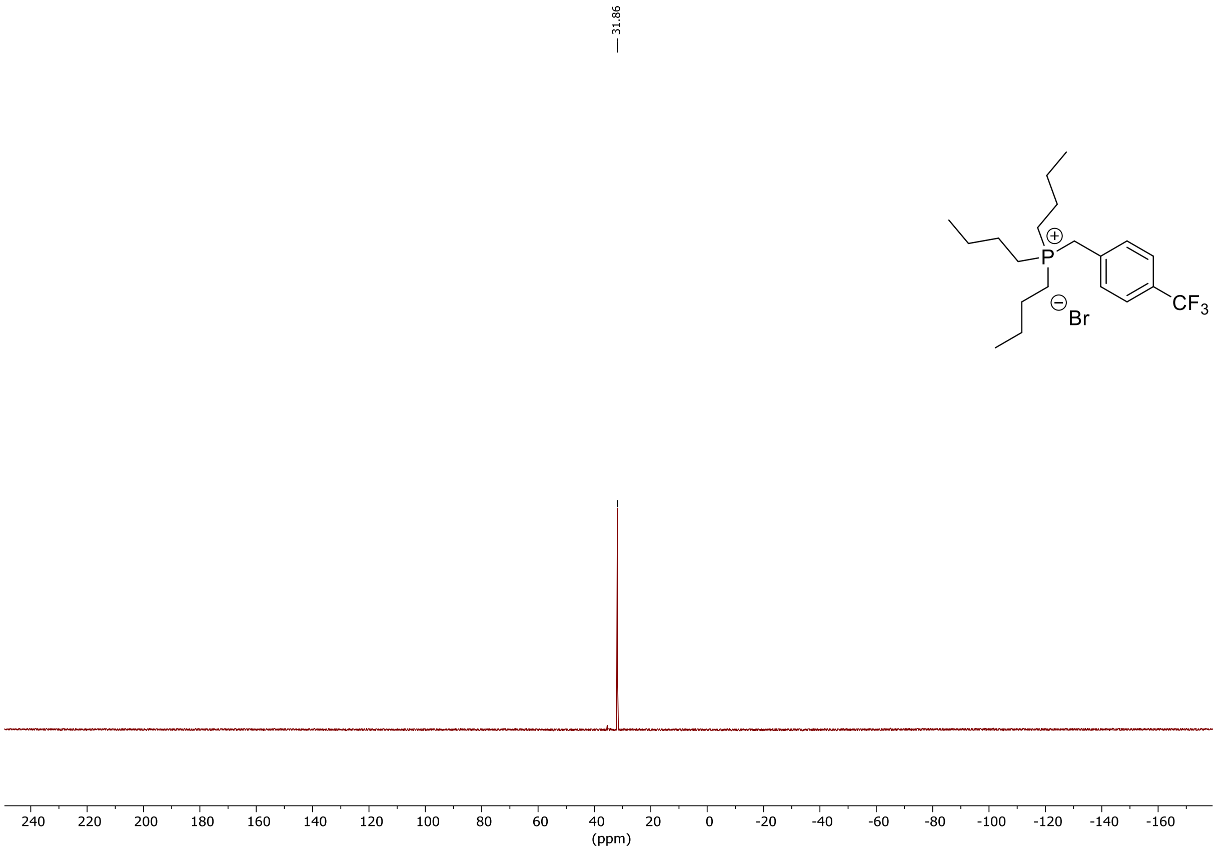

Fig. S247 ^{31}P NMR (203 MHz, chloroform-*d*, 298 K) spectrum of compound **9g**

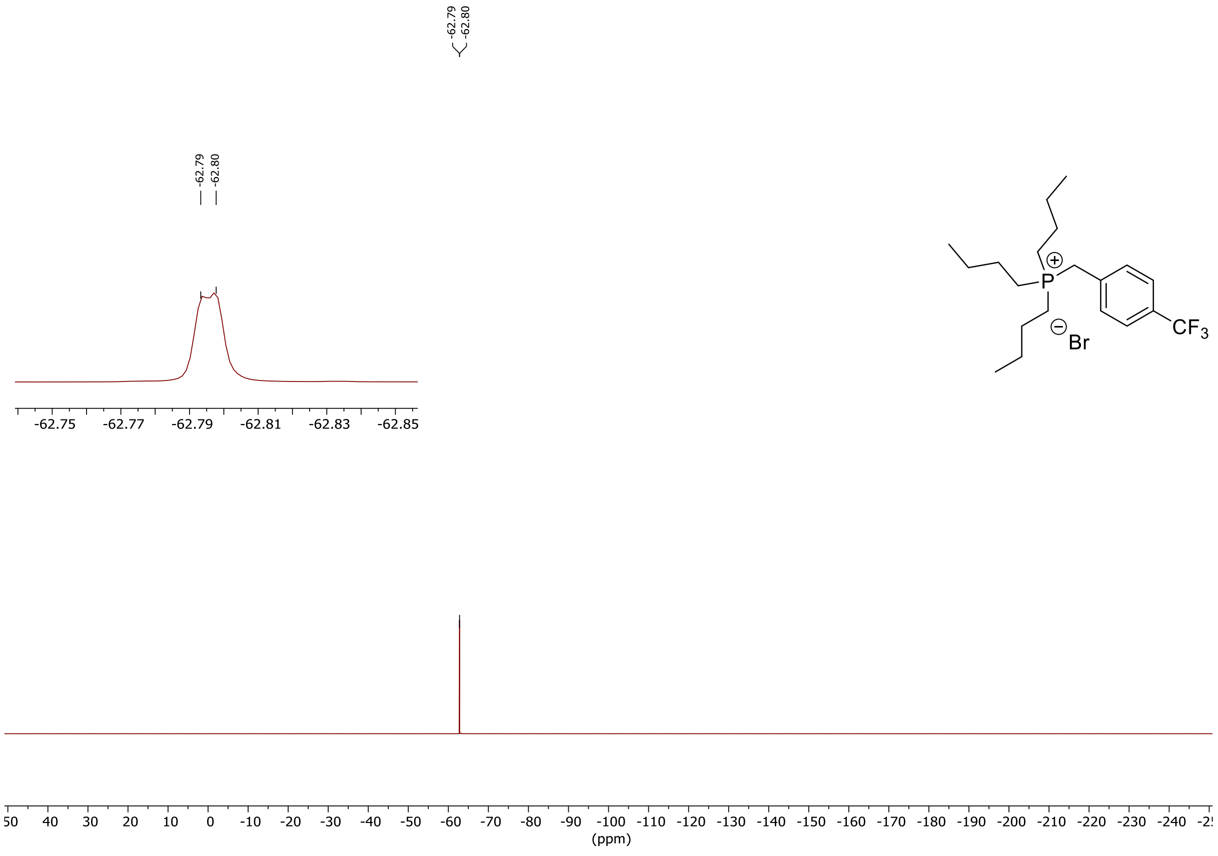

Fig. S248 ^{19}F NMR (565 MHz, chloroform-*d*, 298 K) spectrum of compound **9g**

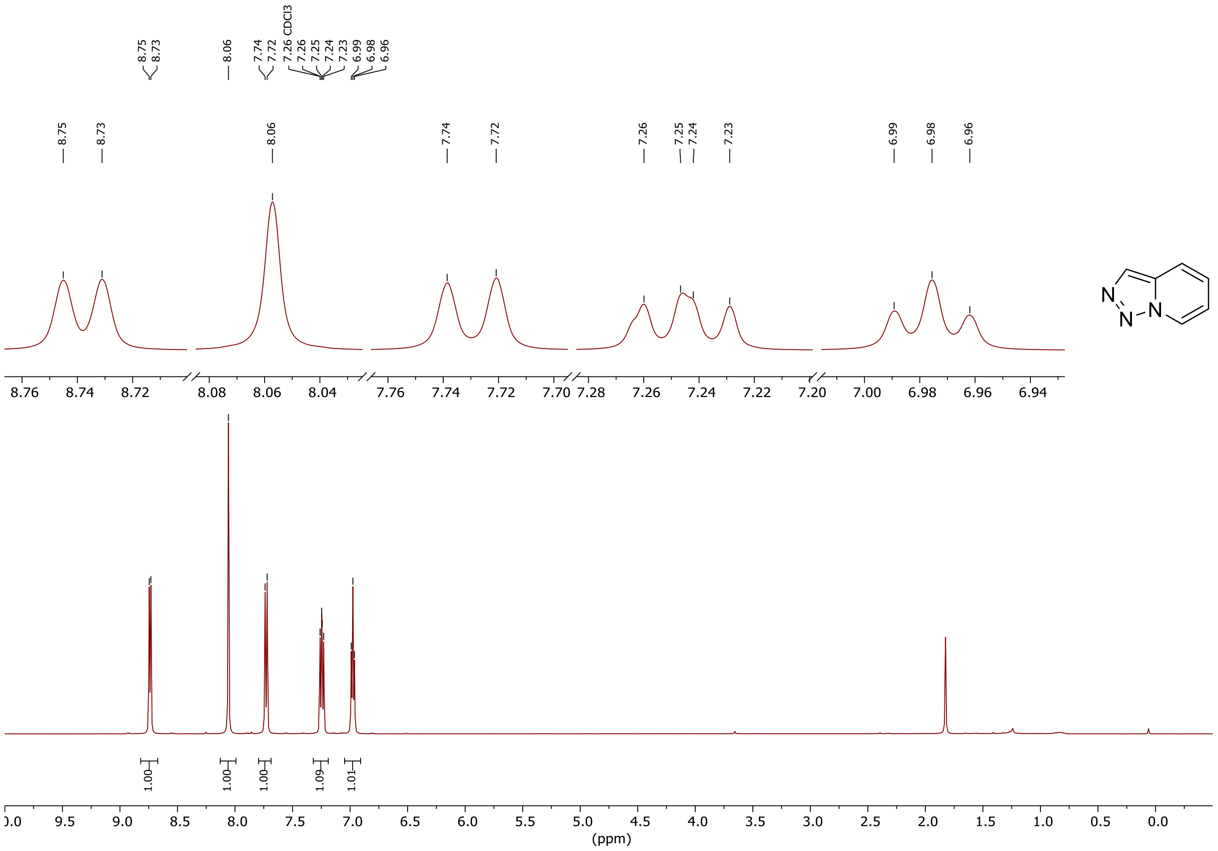

Fig. S249 ^{1}H NMR (501 MHz, chloroform-*d*, 298 K) spectrum of compound **3a**

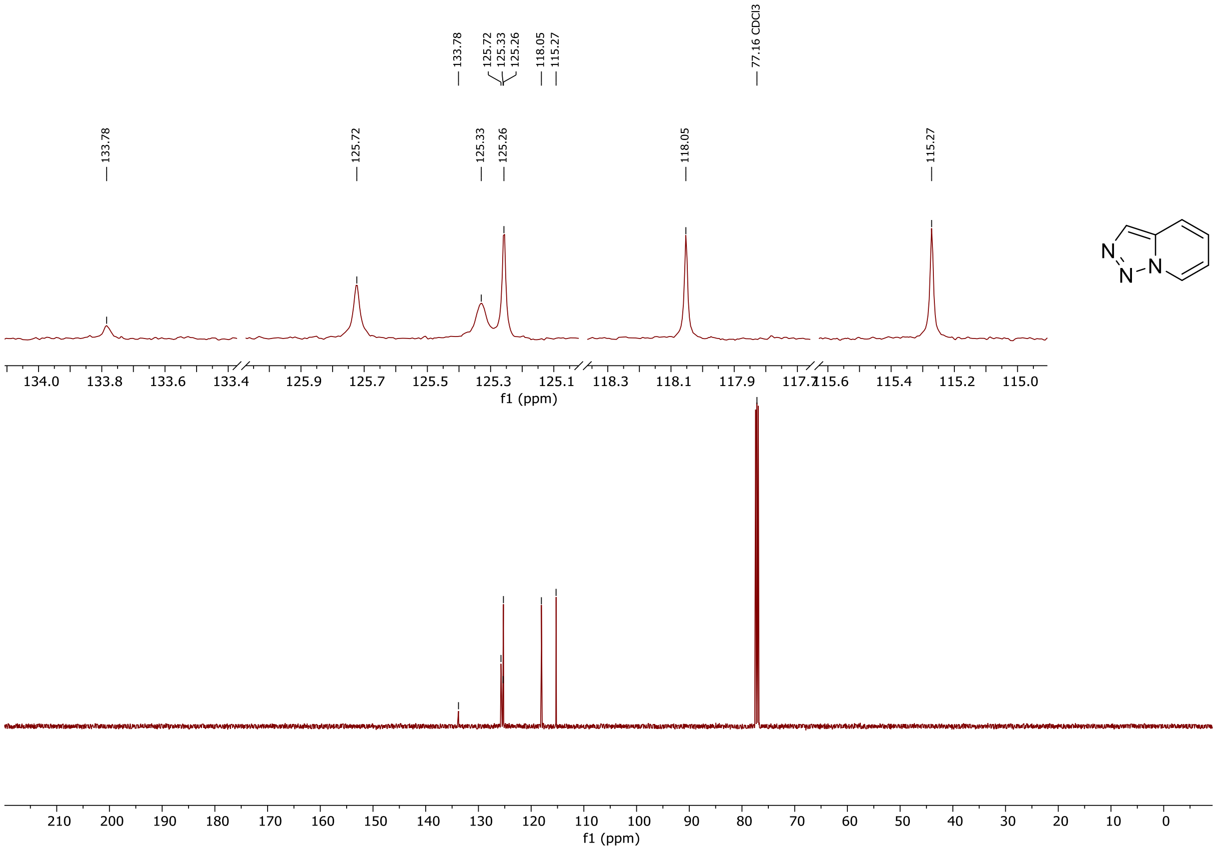

Fig. S250 ^{13}C{^{1}H} NMR (126 MHz, chloroform-*d*, 298 K) spectrum of compound **3a**

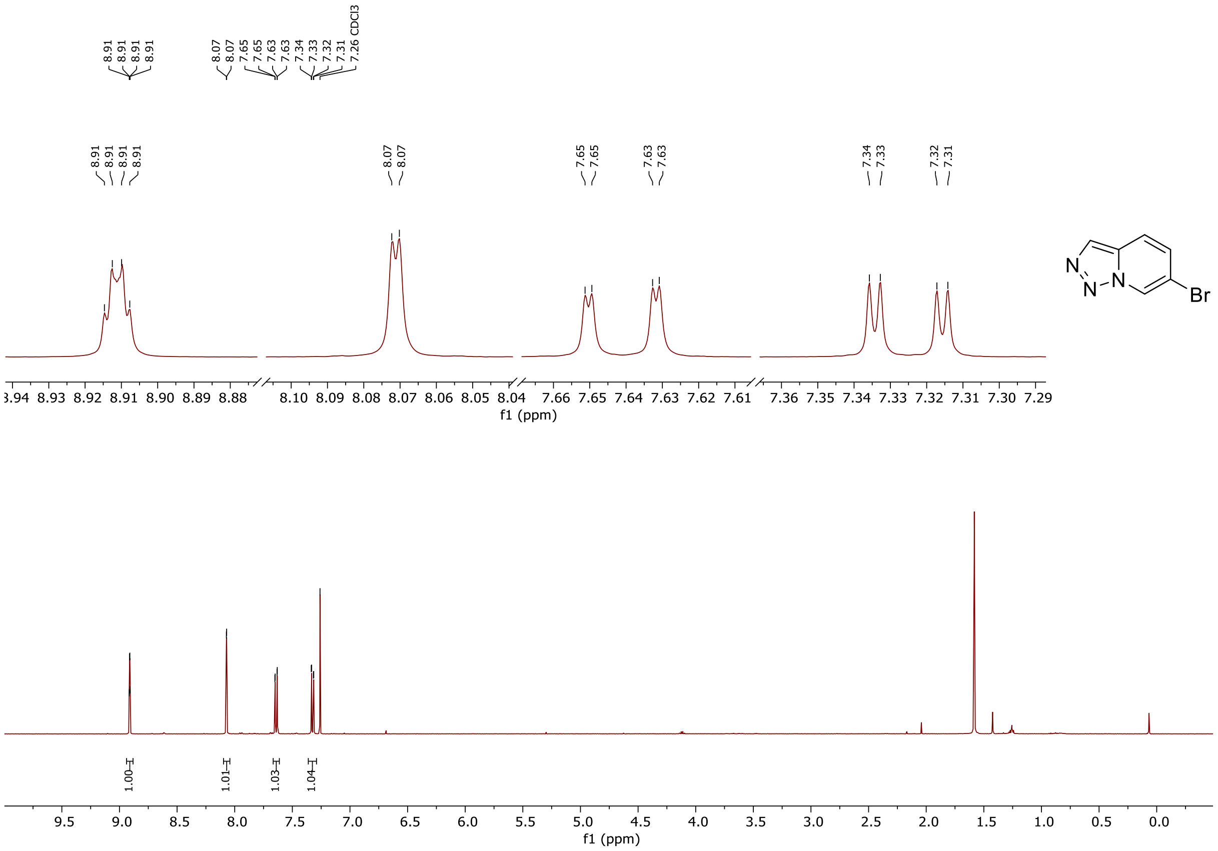

Fig. S251 ^{1}H NMR (501 MHz, chloroform-*d*, 298 K) spectrum of compound **3b**

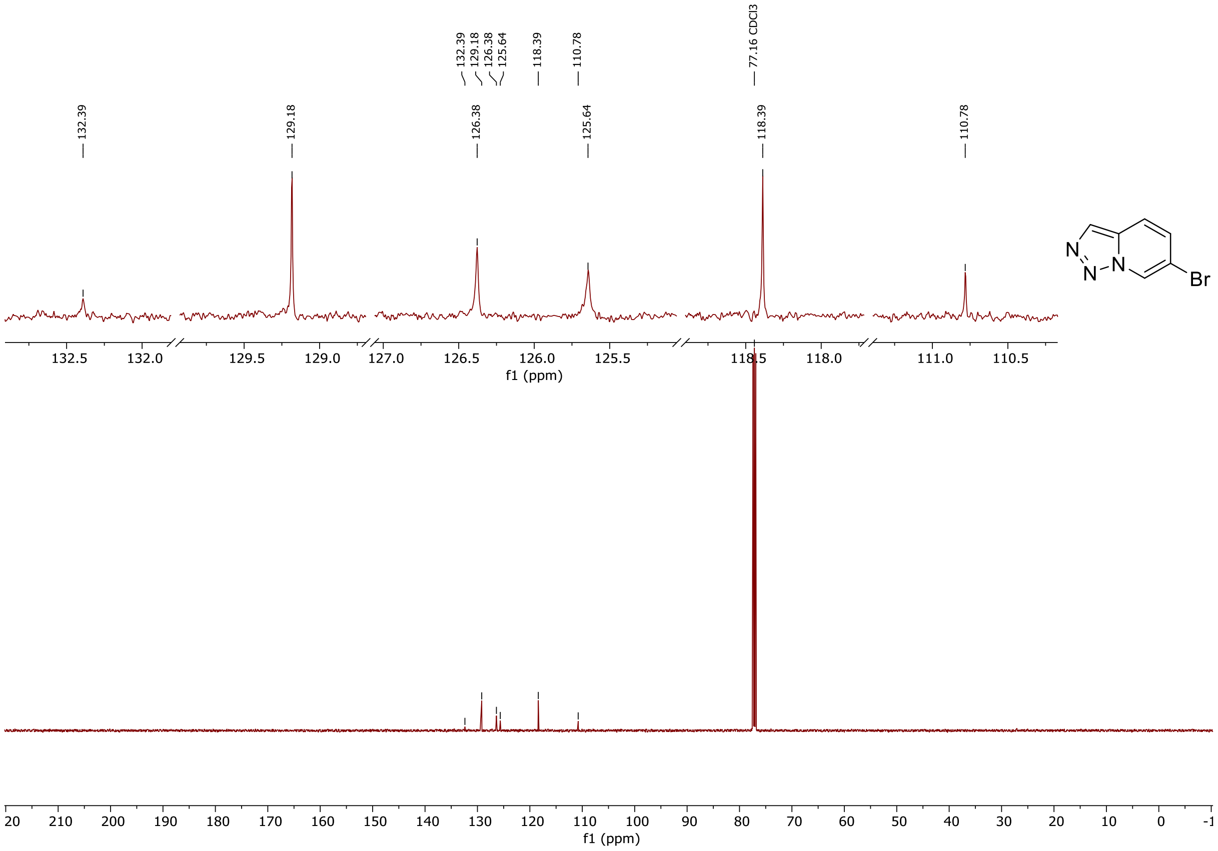

Fig. S252 ^{13}C{^{1}H} NMR (126 MHz, chloroform-*d*, 298 K) spectrum of compound **3b**

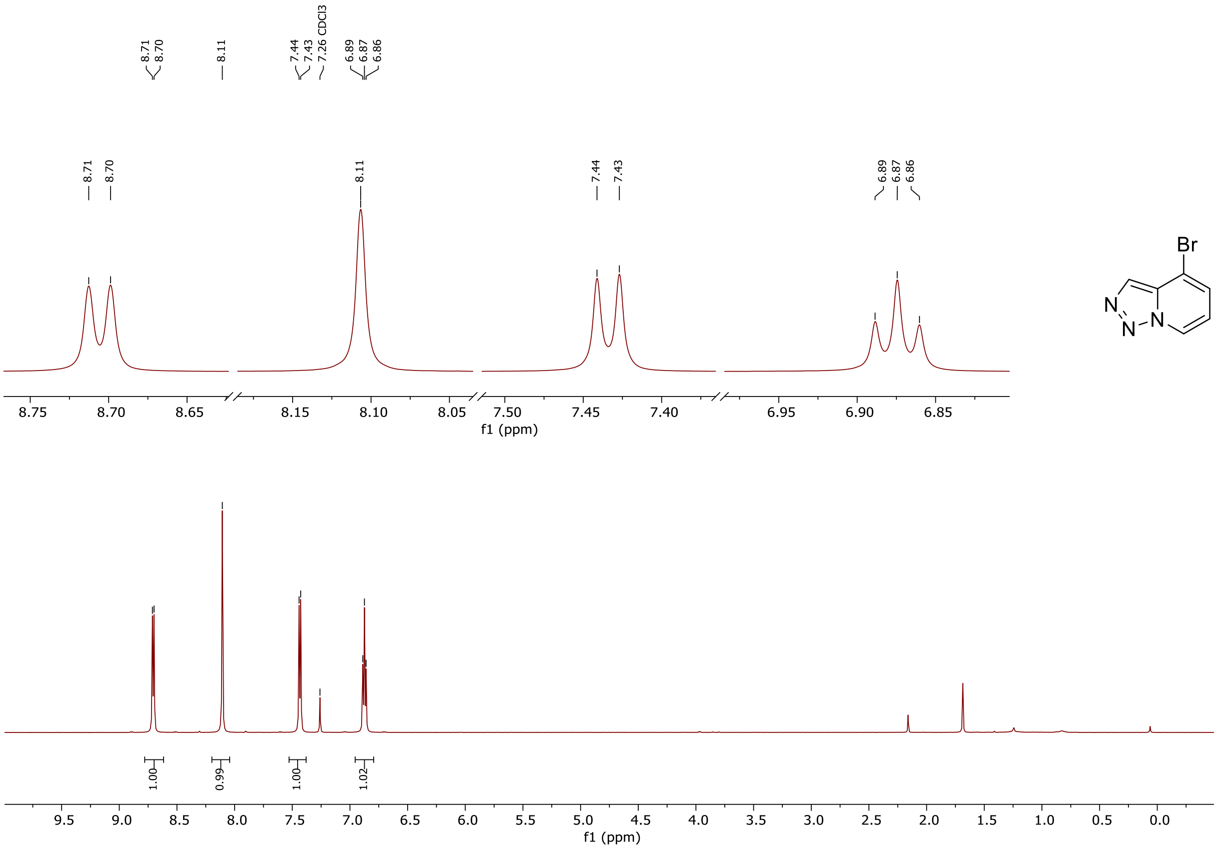

Fig. S253 ^{1}H NMR (501 MHz, chloroform-*d*, 298 K) spectrum of compound **3c**

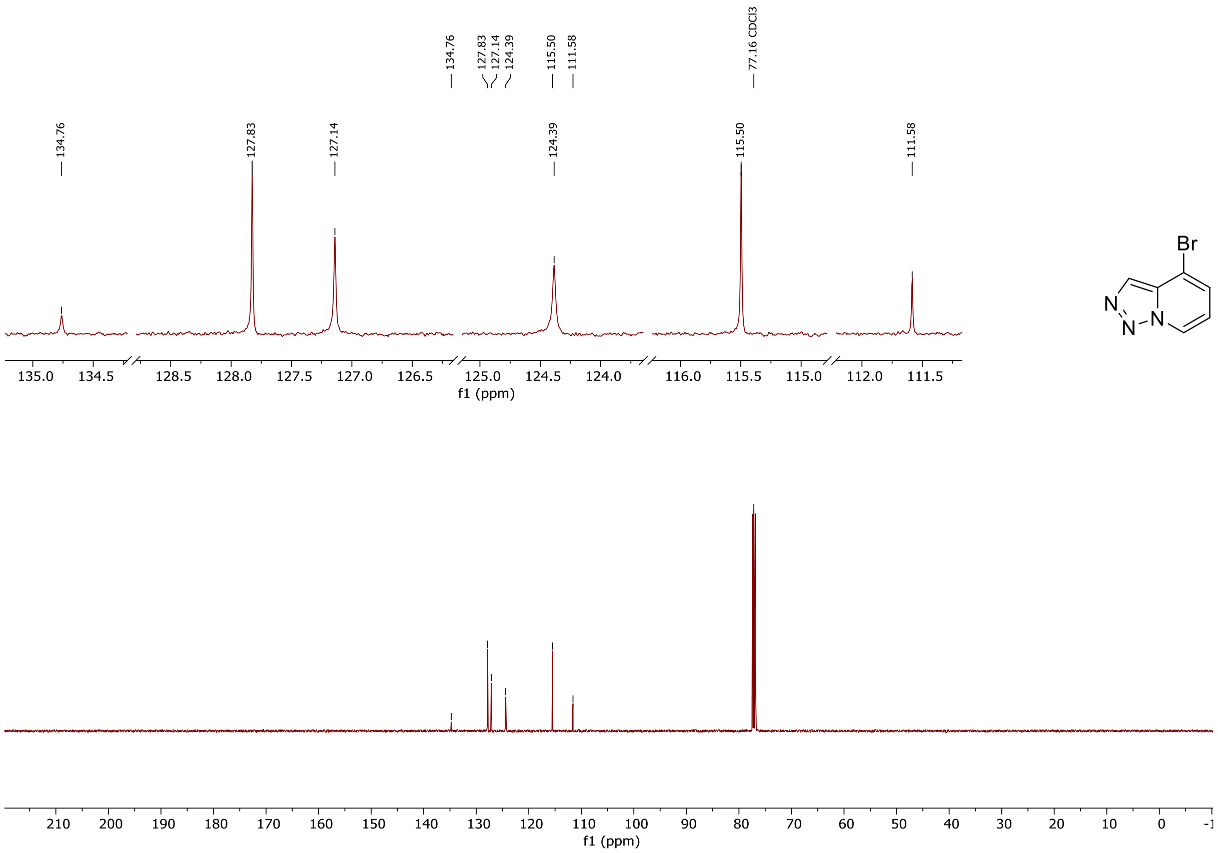

Fig. S254 ^{13}C{^{1}H} NMR (126 MHz, chloroform-*d*, 298 K) spectrum of compound **3c**

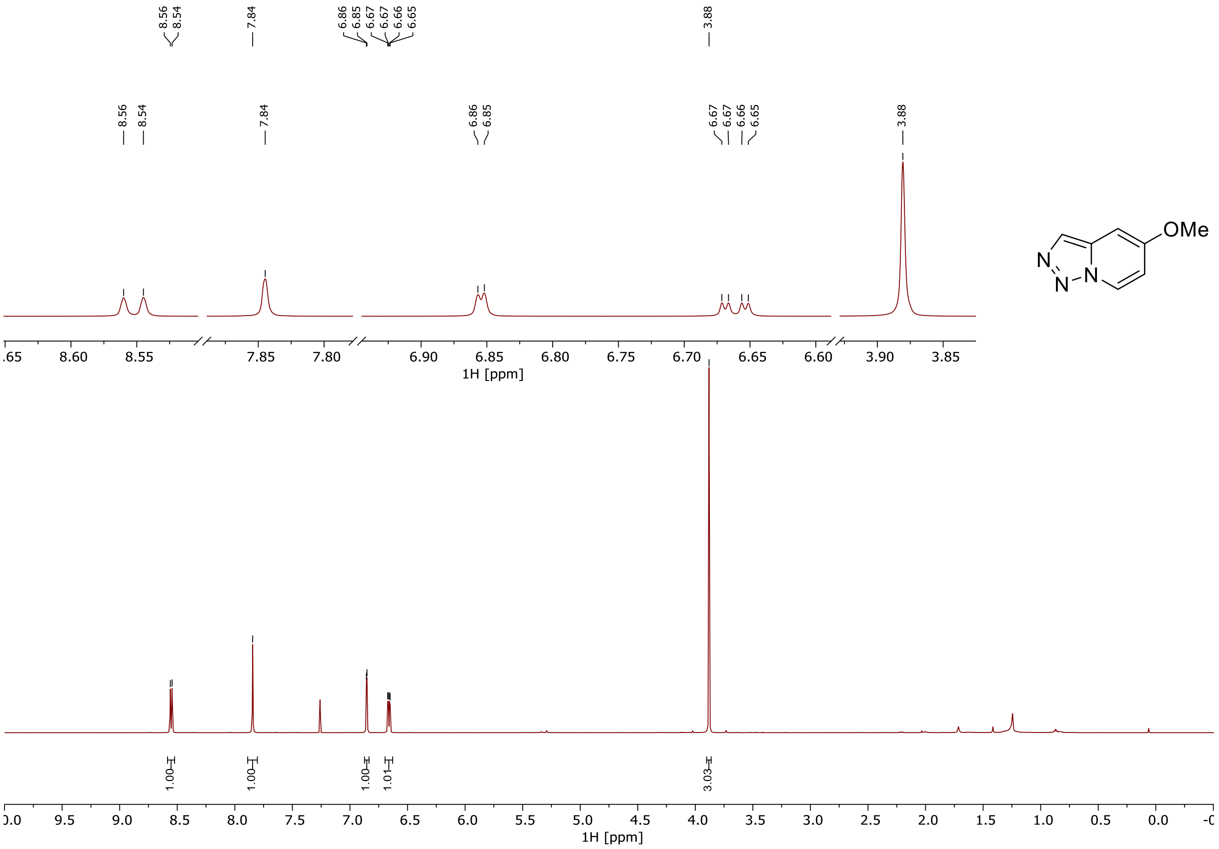

Fig. S255 ^{1}H NMR (501 MHz, chloroform-*d*, 298 K) spectrum of compound **3d**

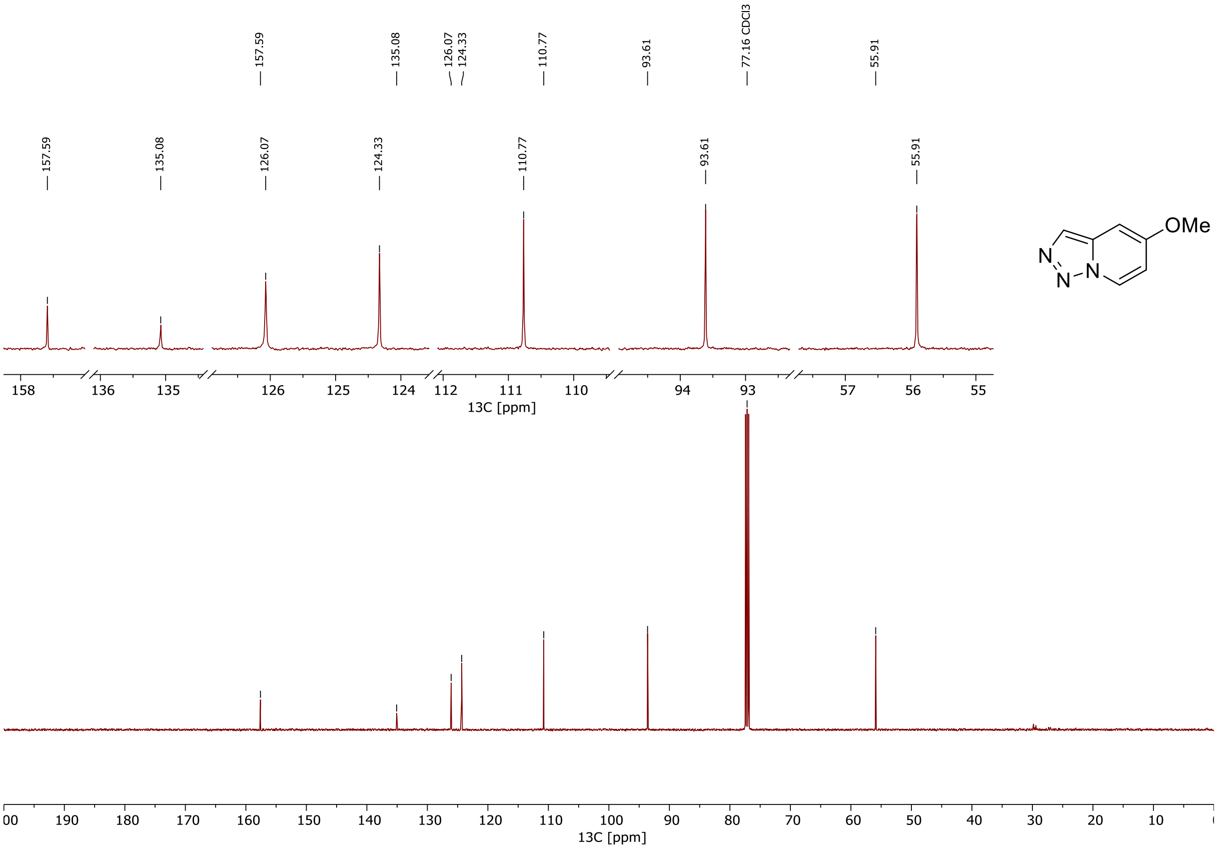

Fig. S256 ^{13}C{^{1}H} NMR (126 MHz, chloroform-*d*, 298 K) spectrum of compound **3d**

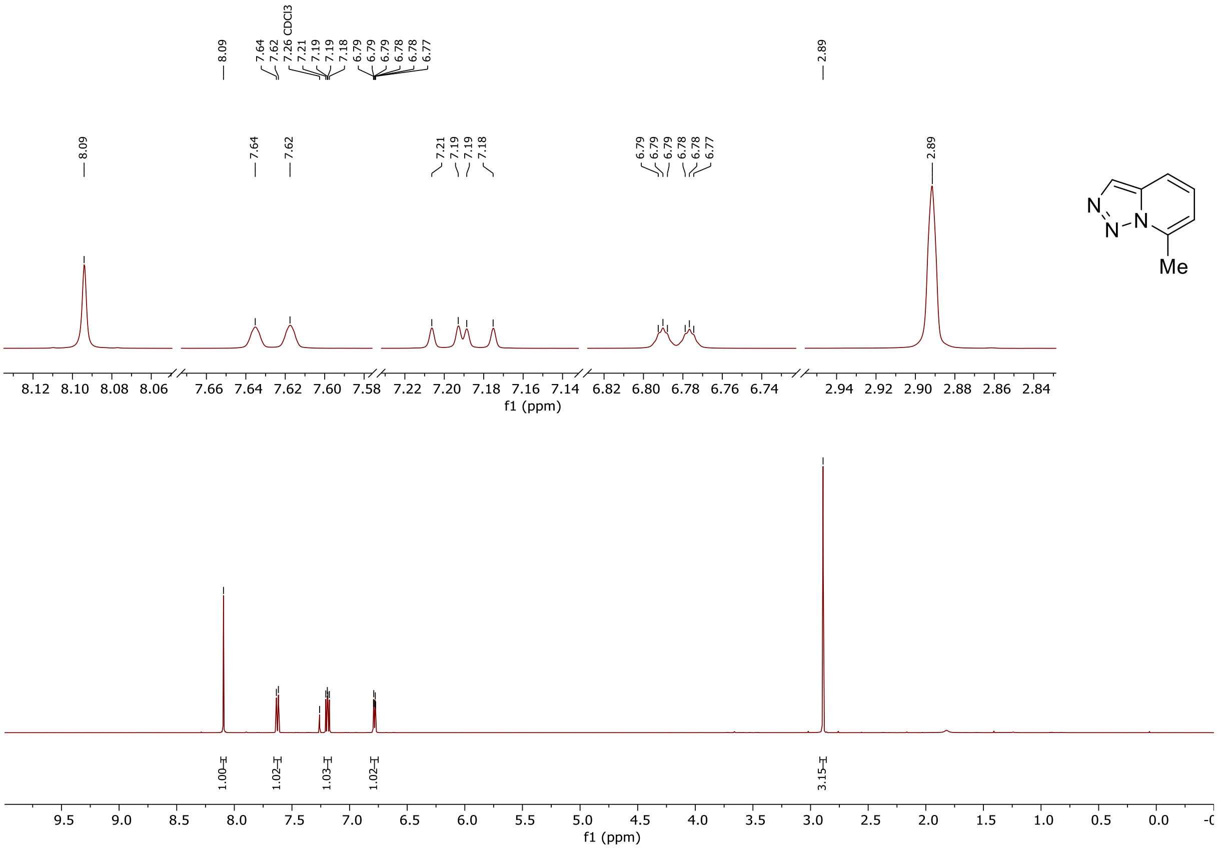

Fig. S257 ^{1}H NMR (501 MHz, chloroform-*d*, 298 K) spectrum of compound **3e**

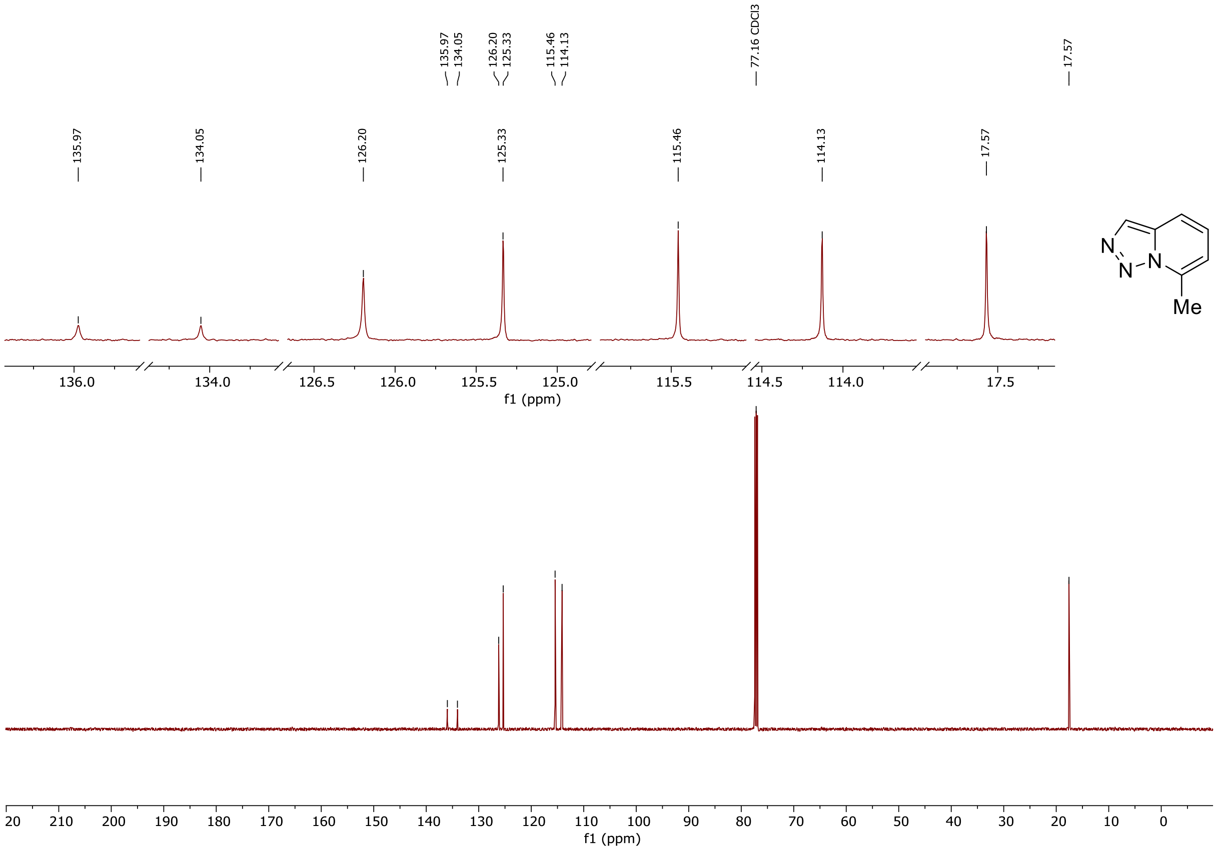

Fig. S258 ^{13}C{^{1}H} NMR (126 MHz, chloroform-*d*, 298 K) spectrum of compound **3e**

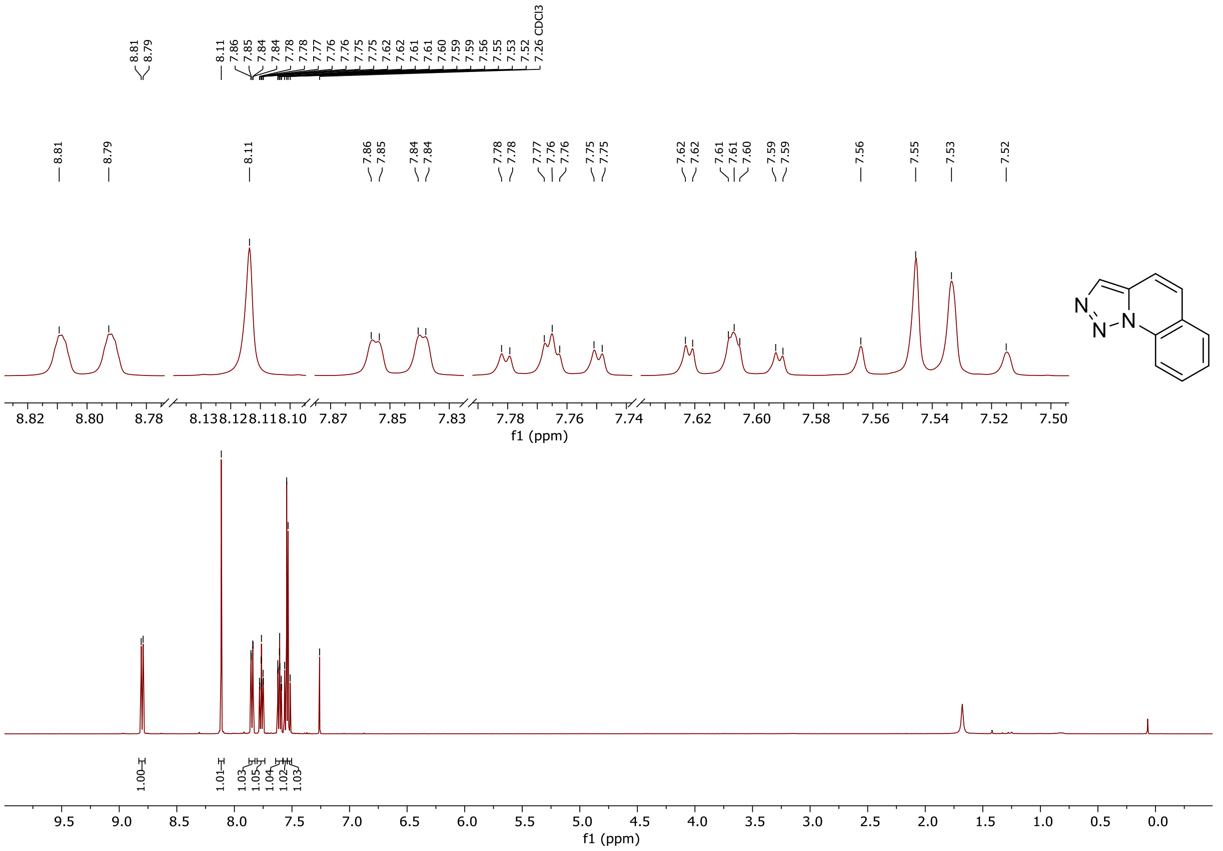

Fig. S259 ^{1}H NMR (501 MHz, chloroform-*d*, 298 K) spectrum of compound **3f**

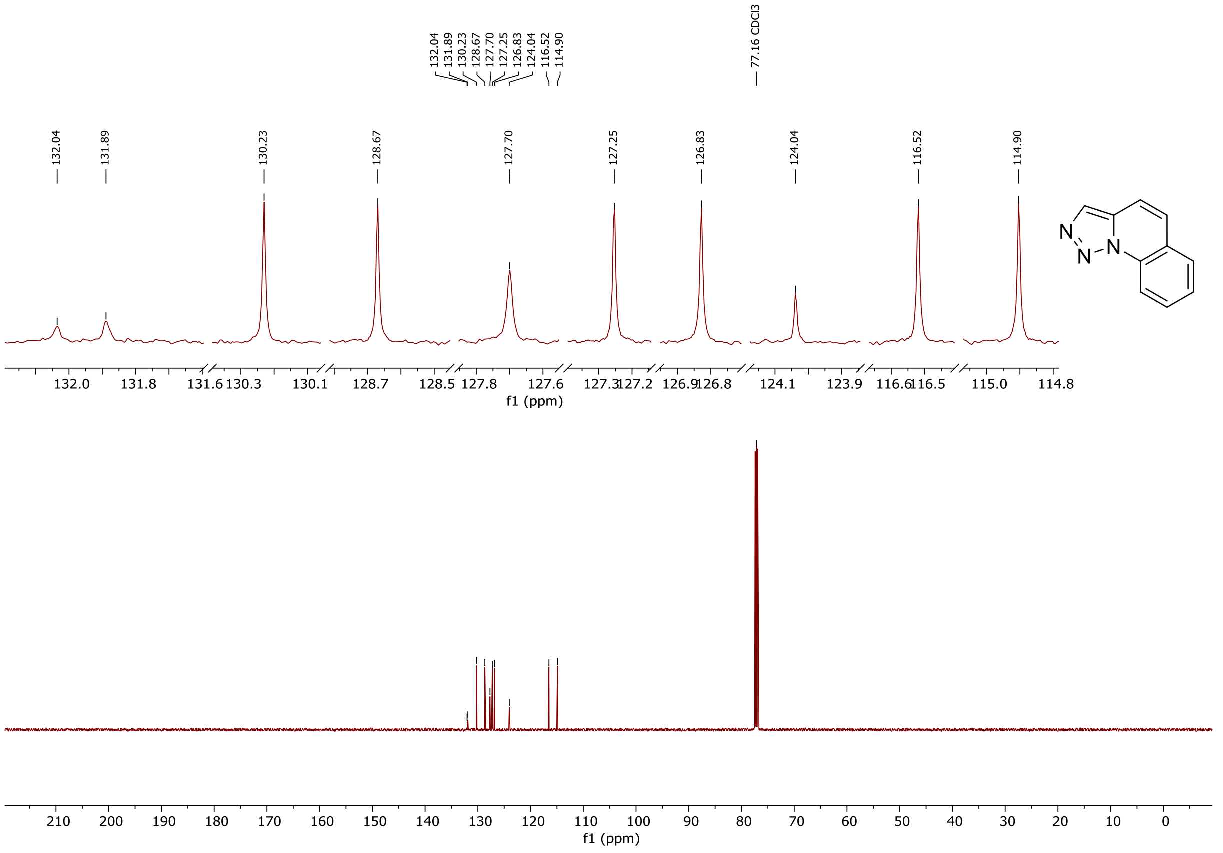

Fig. S260 $^{13}C\{^{1}H\}$ NMR (126 MHz, chloroform-*d*, 298 K) spectrum of compound **3f**

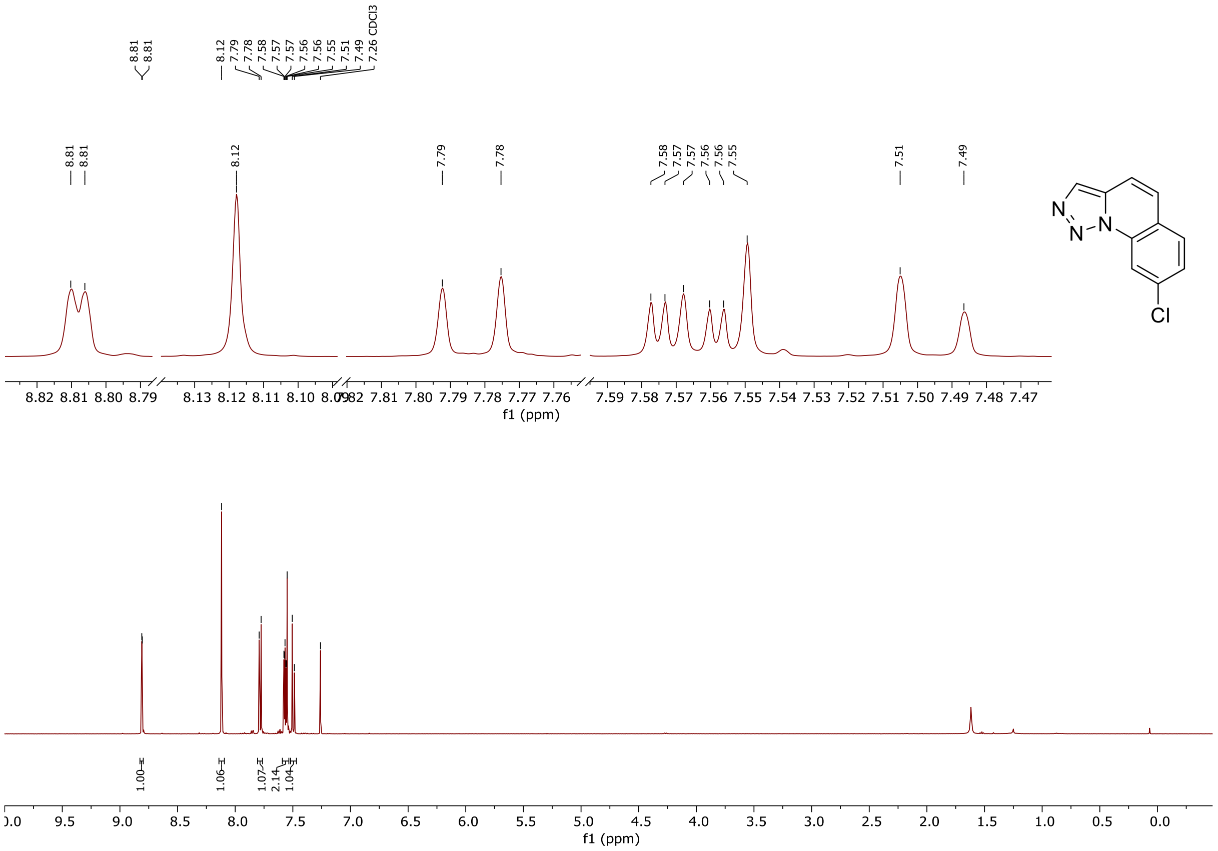

Fig. S261 ^{1}H NMR (501 MHz, chloroform-*d*, 298 K) spectrum of compound **3g**

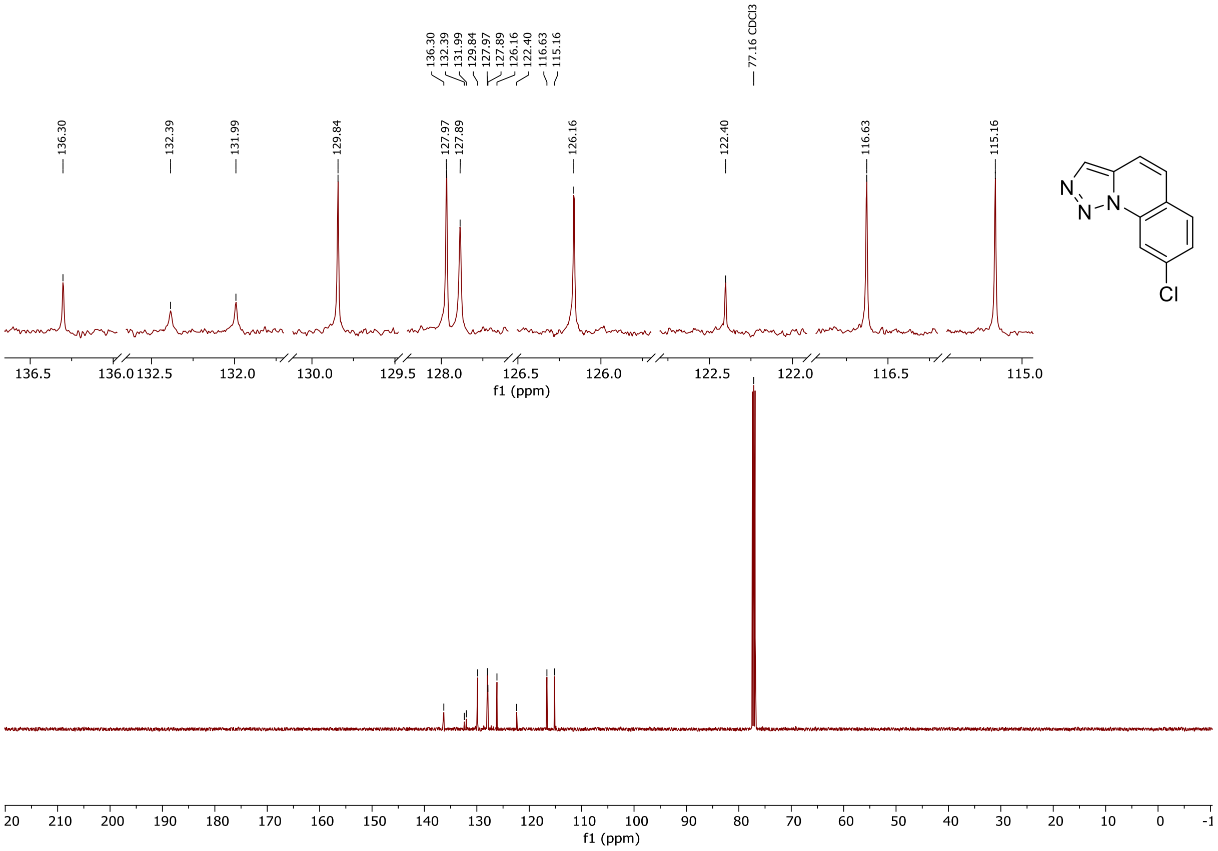

Fig. S262 $^{13}C\{^{1}H\}$ NMR (126 MHz, chloroform-*d*, 298 K) spectrum of compound **3g**

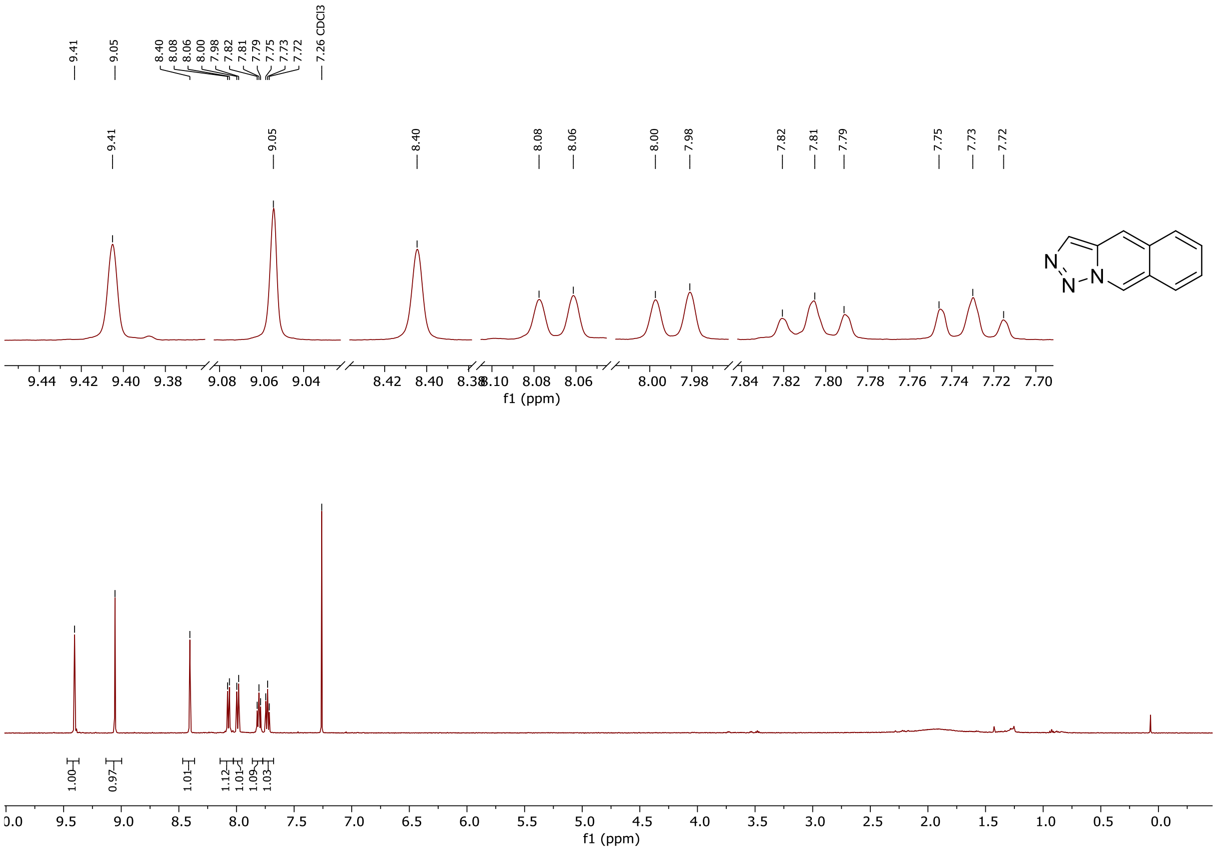

Fig. S263 ^{1}H NMR (501 MHz, chloroform-*d*, 298 K) spectrum of compound **3h**

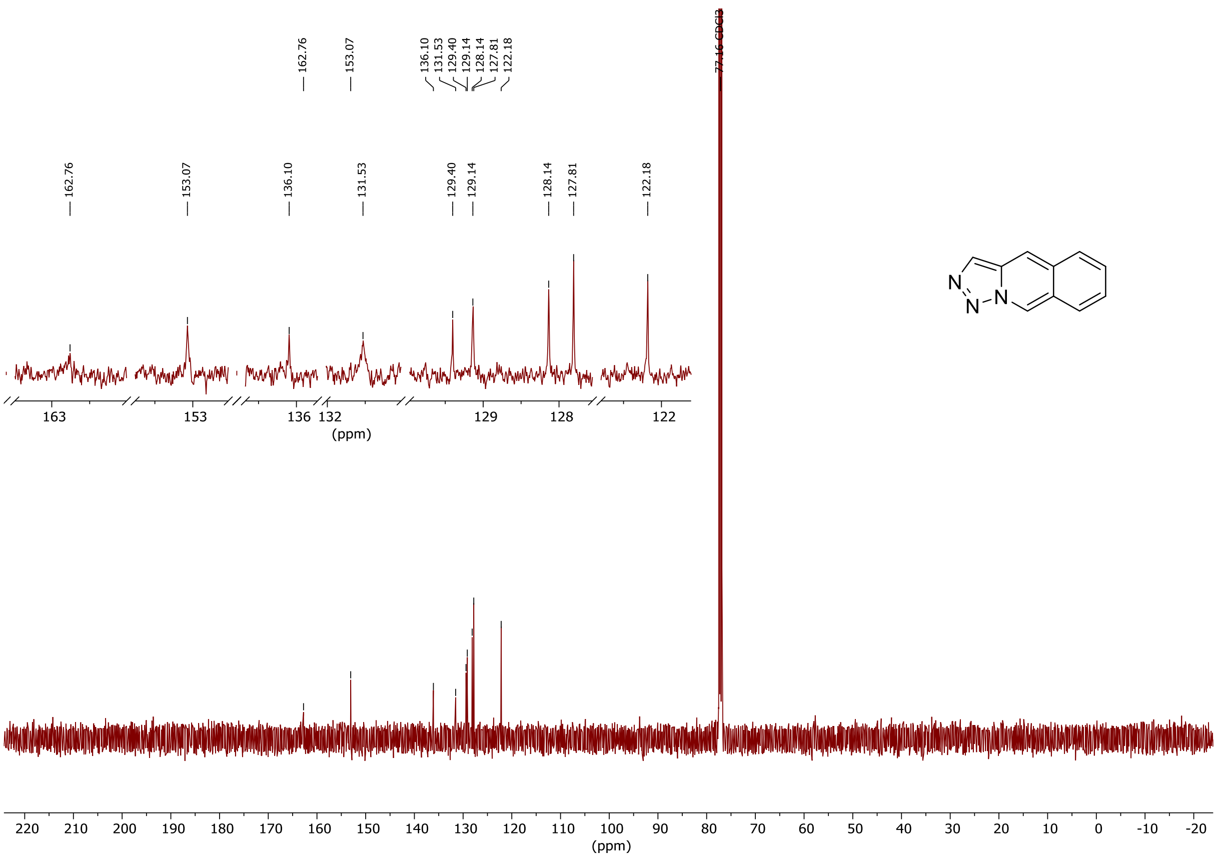

Fig. S264 ^{13}C{^{1}H} NMR (126 MHz, chloroform-*d*, 298 K) spectrum of compound **3h**

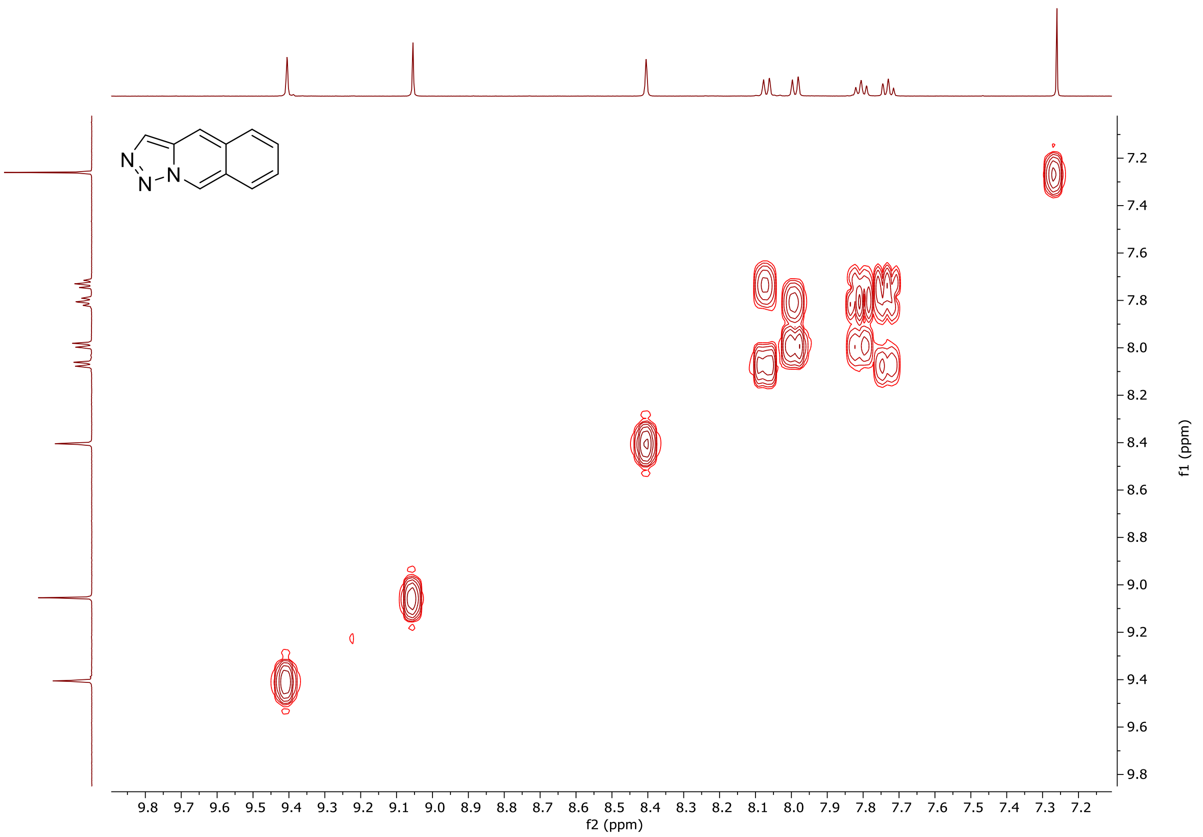

Fig. S265 COSY (501 MHz, 501 MHz, chloroform-*d*, 298 K) spectrum of compound **3h**

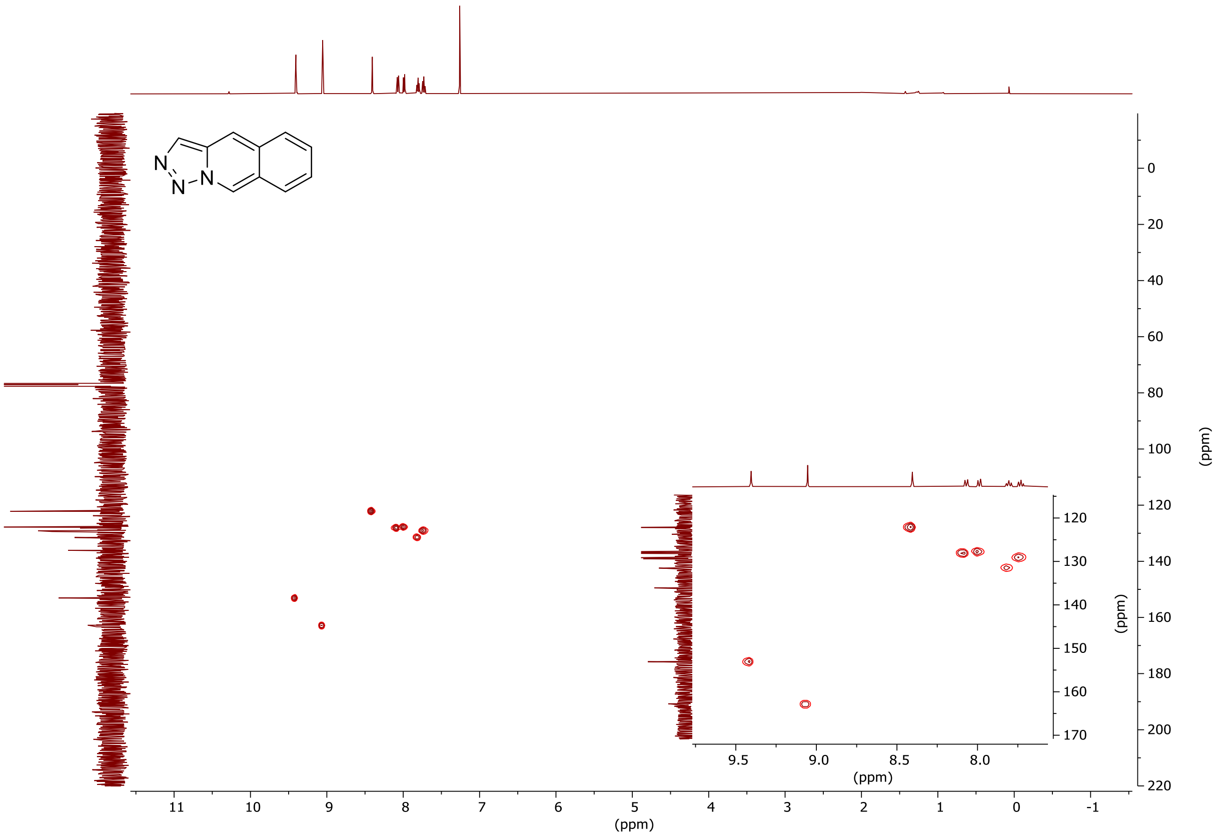

Fig. S266 HSQC (501 MHz, 126 MHz, chloroform-*d*, 298 K) spectrum of compound **3h**

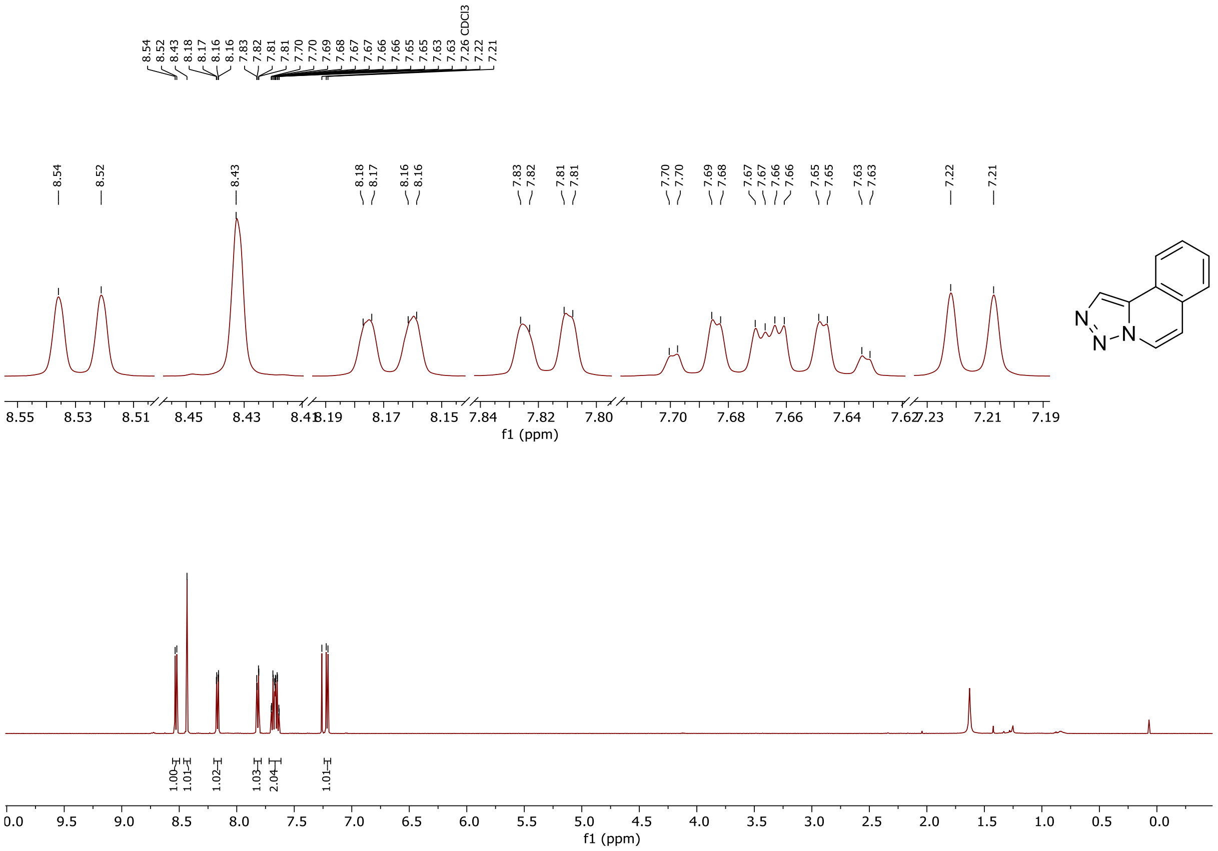

Fig. S267 ^{1}H NMR (501 MHz, chloroform-*d*, 298 K) spectrum of compound **3i**

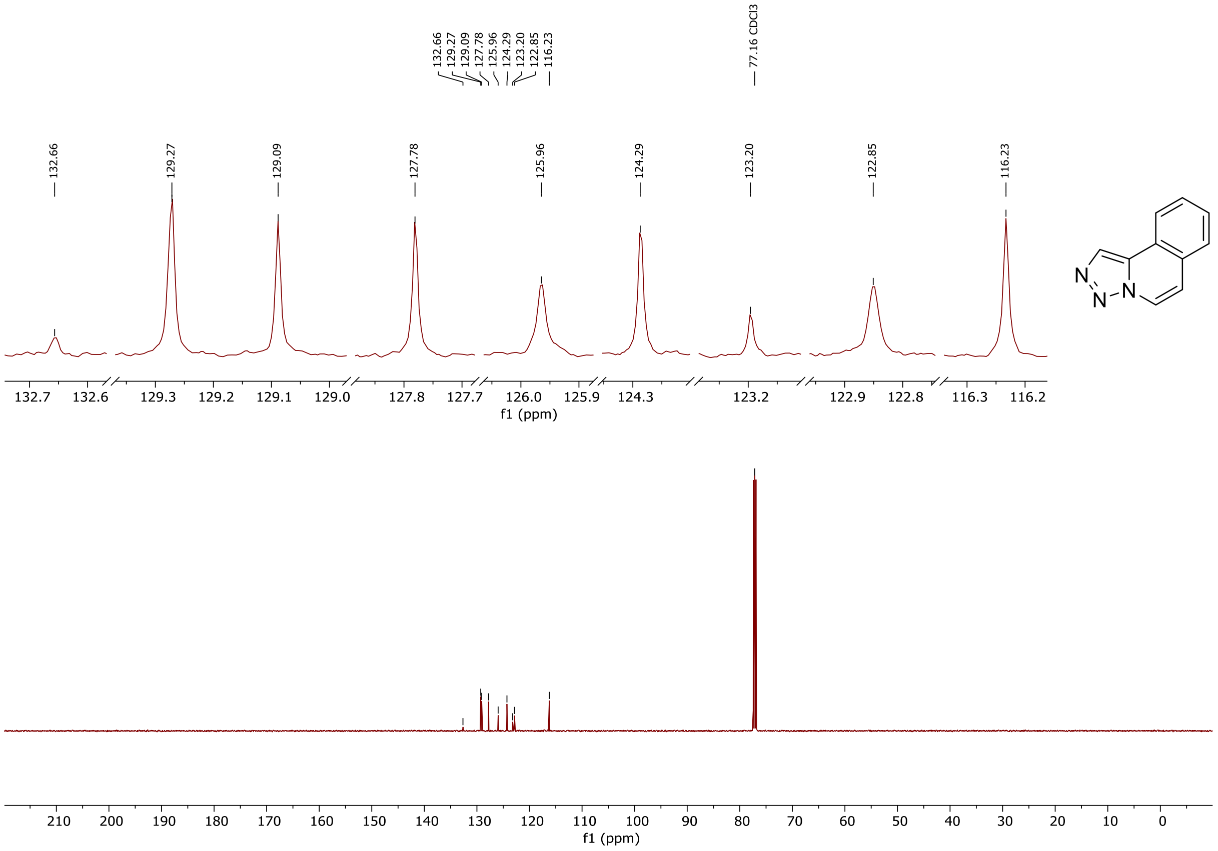

Fig. S268 ^{13}C{^{1}H} NMR (126 MHz, chloroform-*d*, 298 K) spectrum of compound **3i**

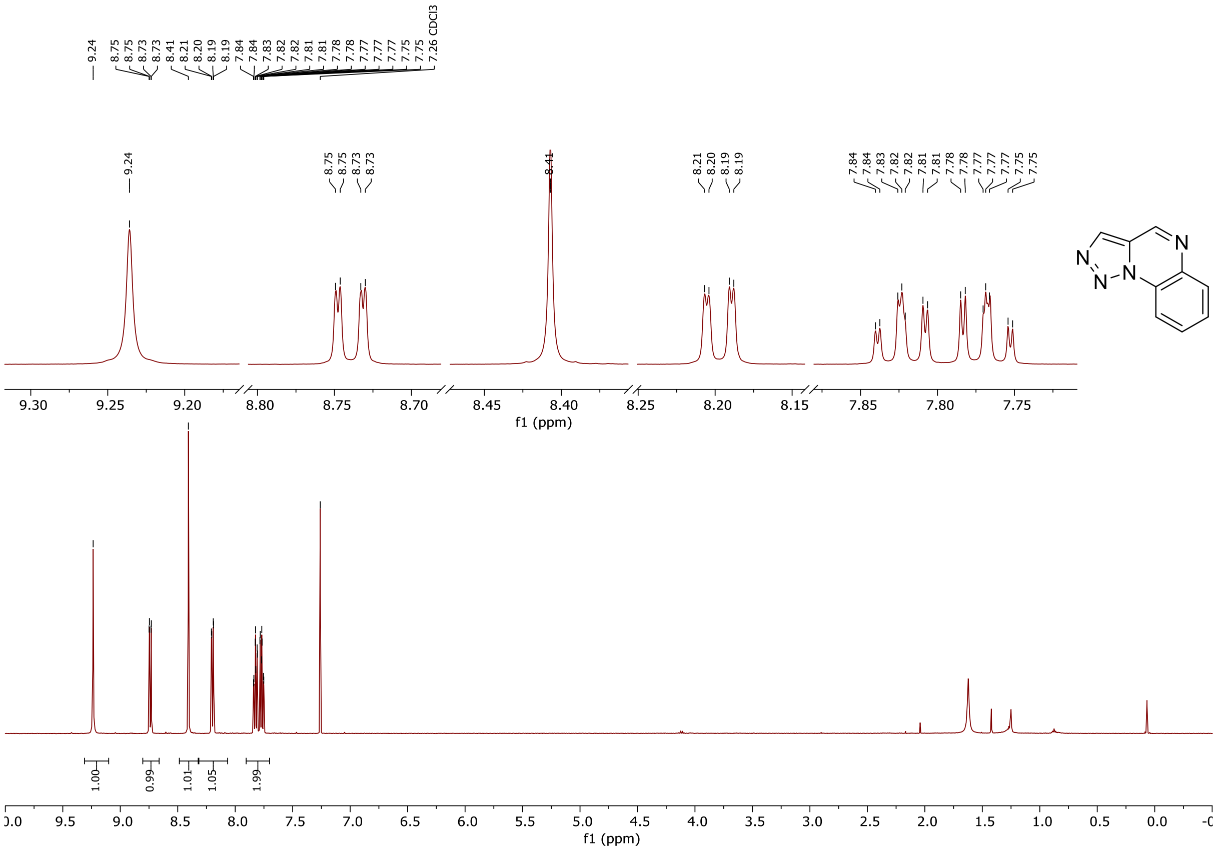

Fig. S269 ^{1}H NMR (501 MHz, chloroform-*d*, 298 K) spectrum of compound **3j**

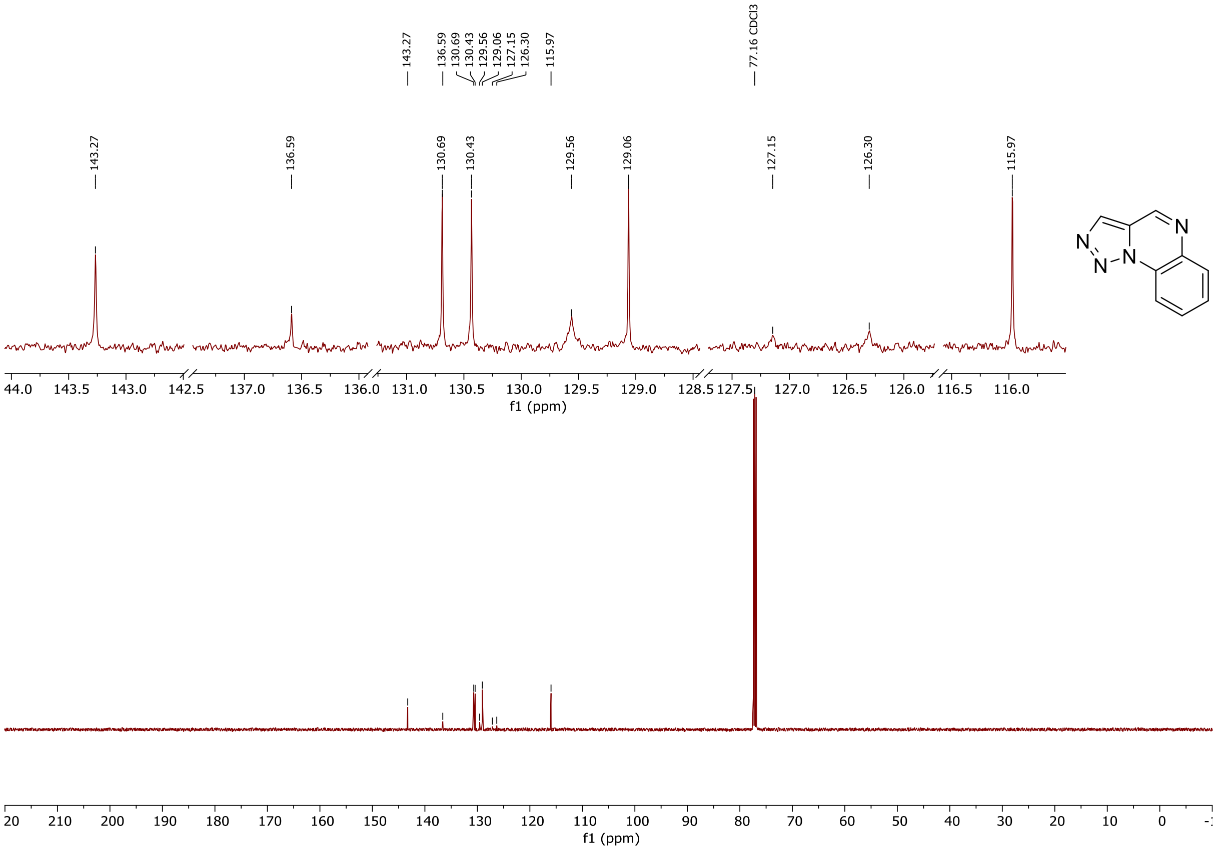

Fig. S270 ^{13}C{^{1}H} NMR (126 MHz, chloroform-*d*, 298 K) spectrum of compound **3j**

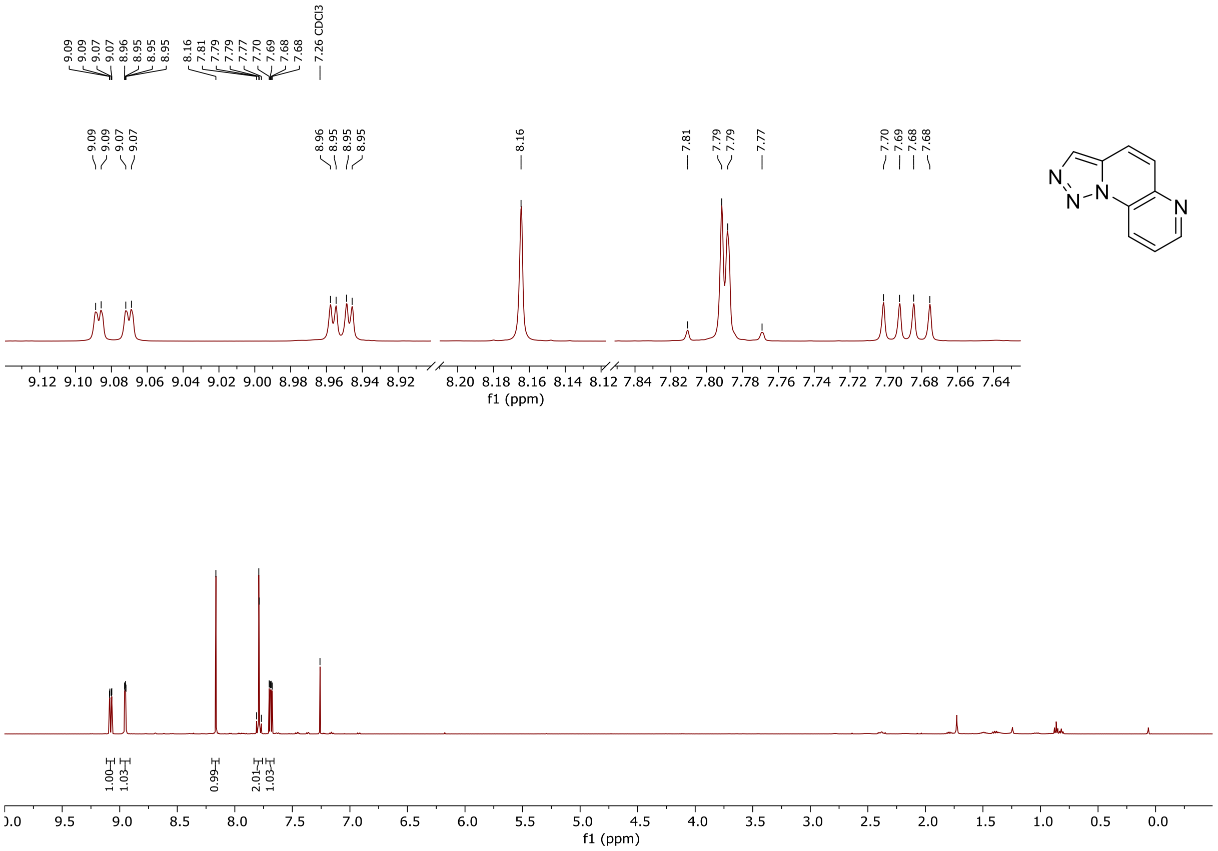

Fig. S271 ^{1}H NMR (501 MHz, chloroform-*d*, 298 K) spectrum of compound **3k**

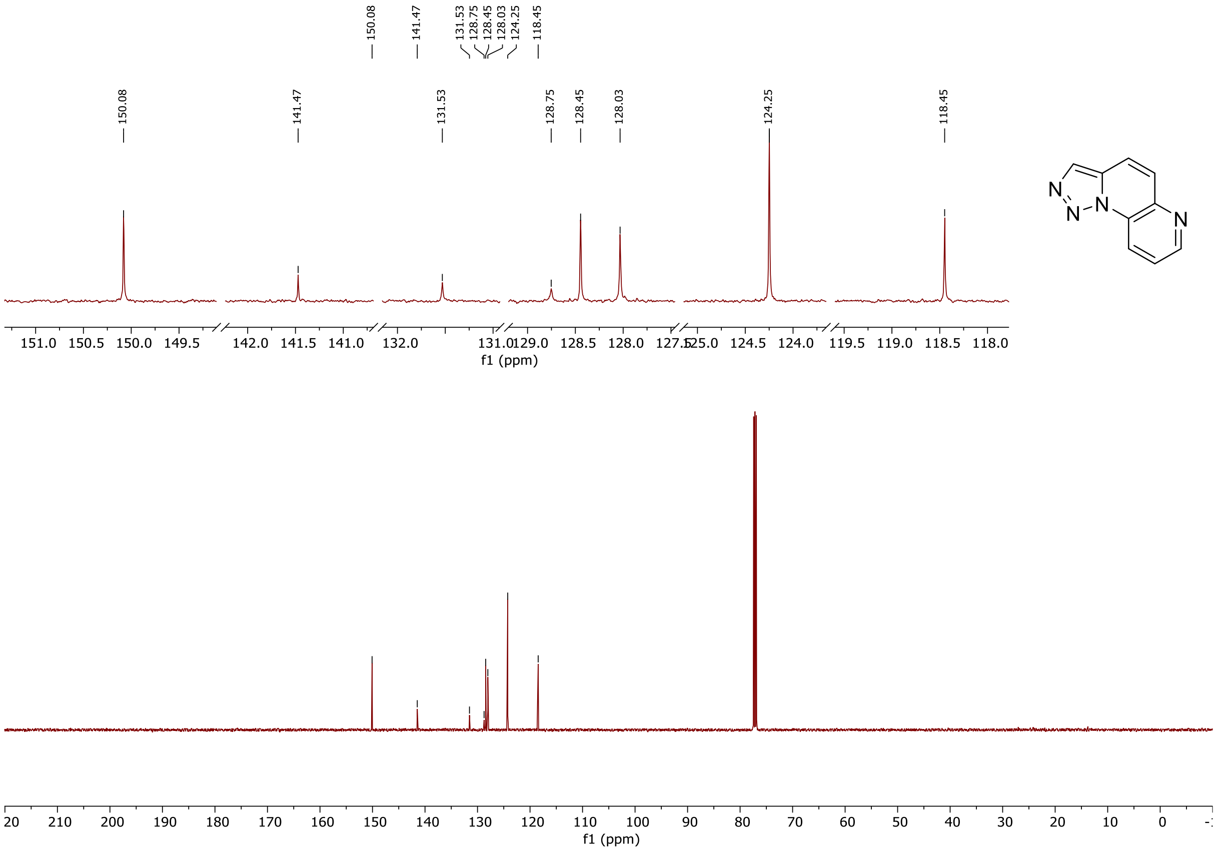

Fig. S272 ^{13}C{^{1}H} NMR (126 MHz, chloroform-*d*, 298 K) spectrum of compound **3k**

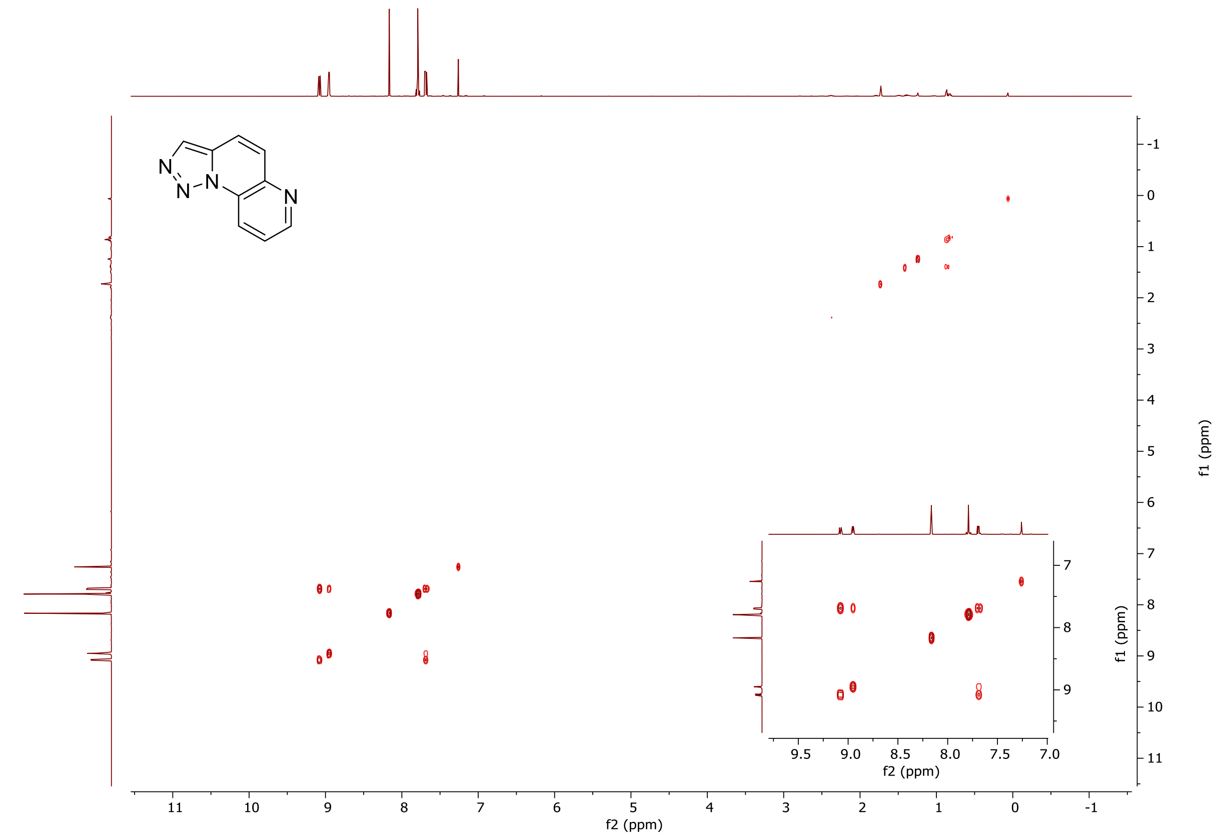

Fig. S273 COSY (501 MHz, 501 MHz, chloroform-*d*, 298 K) spectrum of compound **3k**

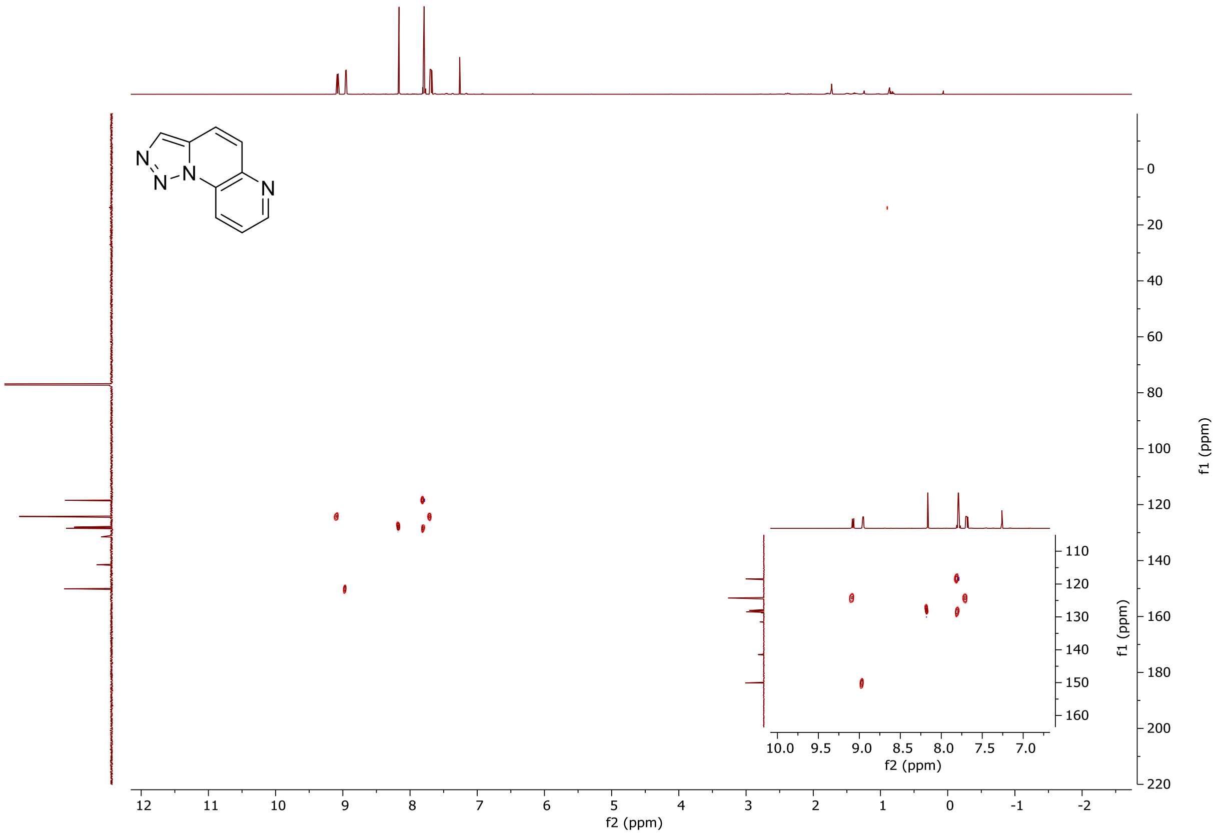

Fig. S274 HSQC (501 MHz, 126 MHz, chloroform-*d*, 298 K) spectrum of compound **3k**

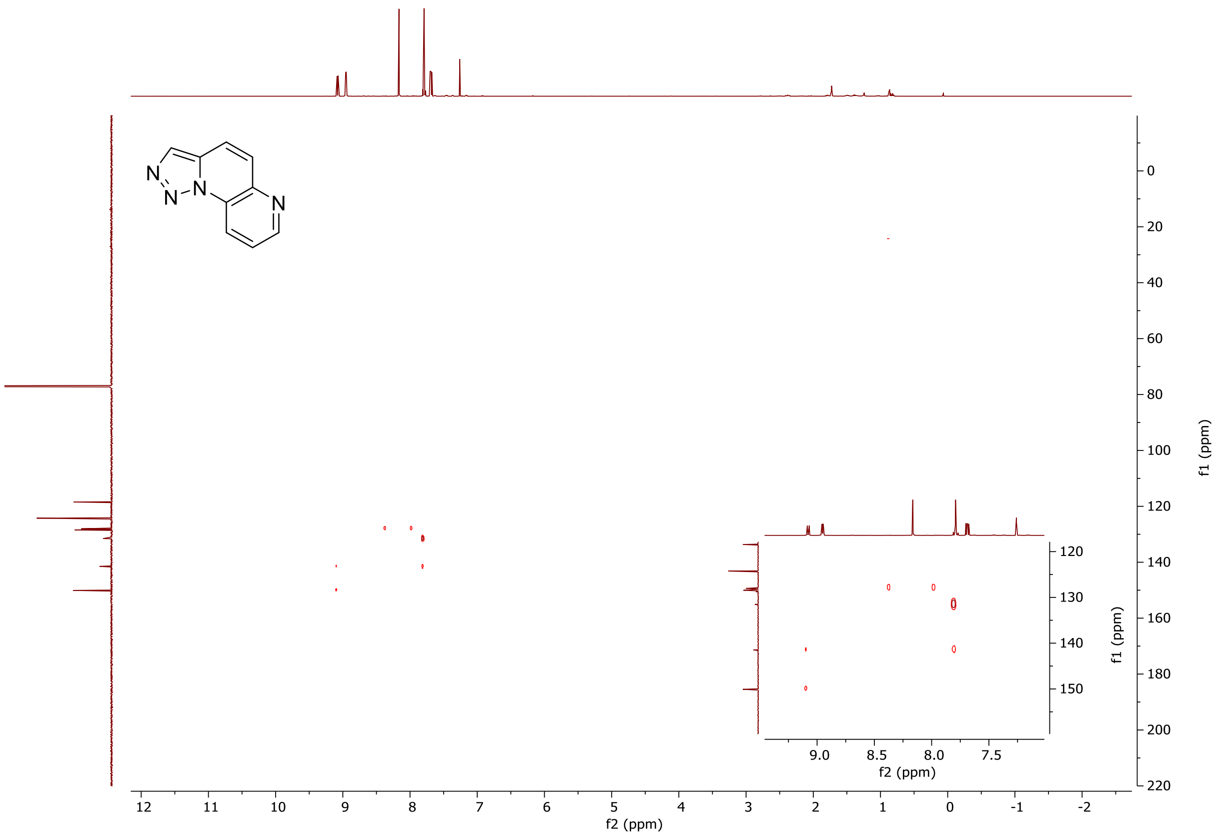

Fig. S275 HMBC (501 MHz, 126 MHz, chloroform-*d*, 298 K) spectrum of compound **3k**

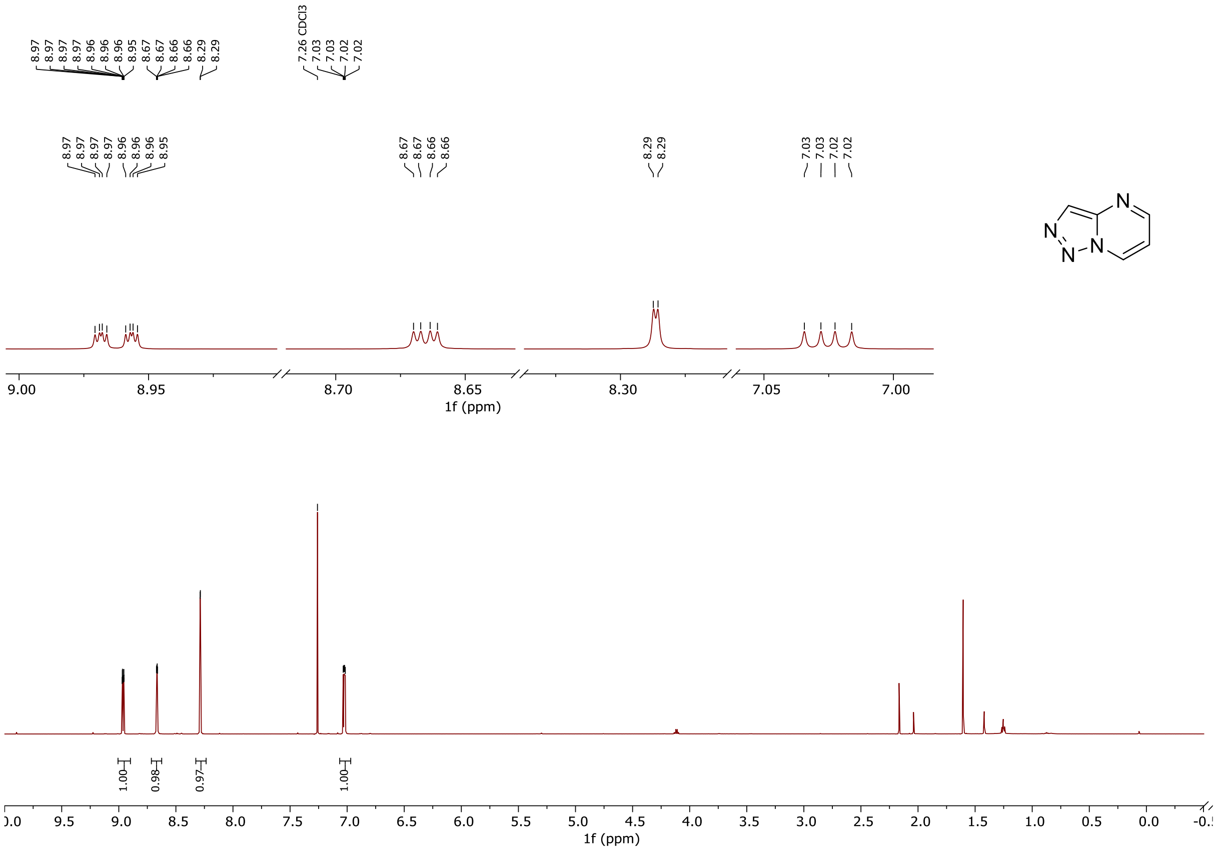

Fig. S276 ^{1}H NMR (600 MHz, chloroform-*d*, 298 K) spectrum of compound **3l**

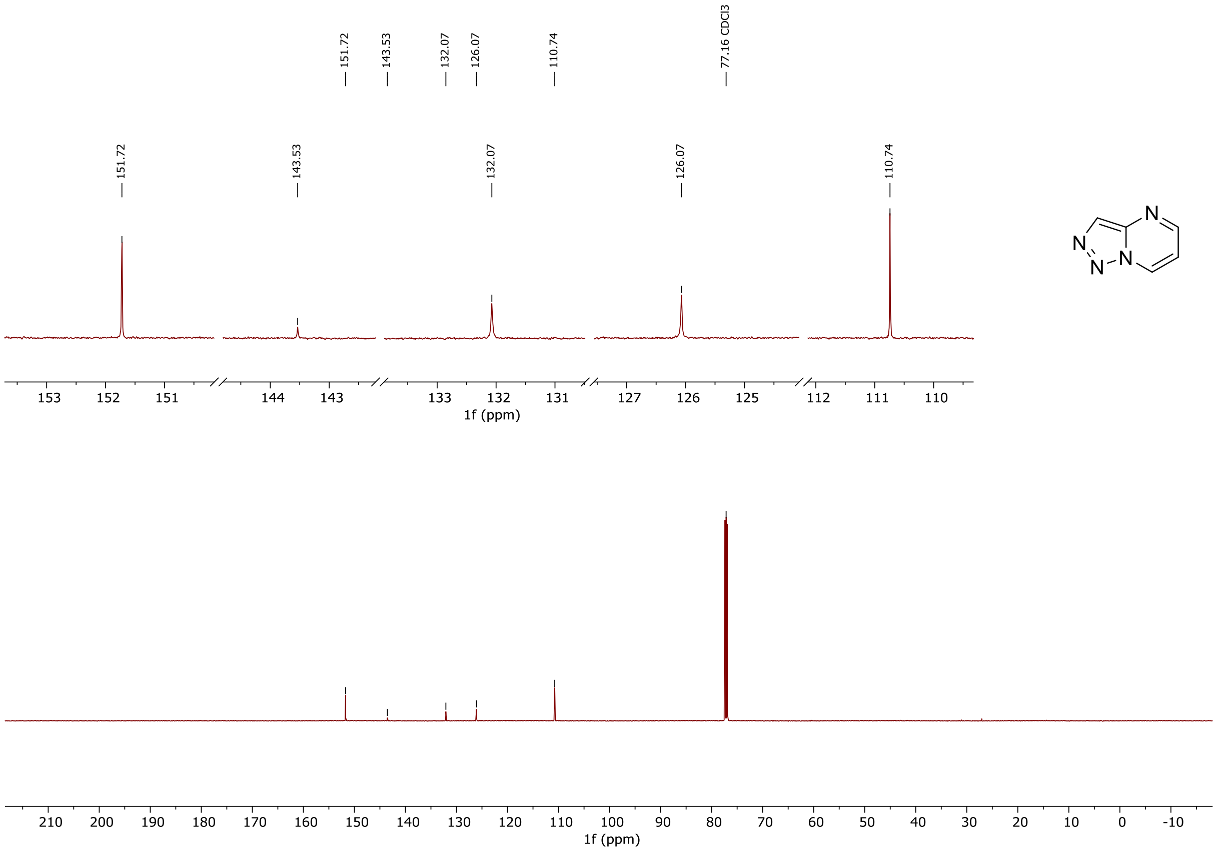

Fig. S277 ^{13}C{^{1}H} NMR (151 MHz, chloroform-*d*, 298 K) spectrum of compound **3l**

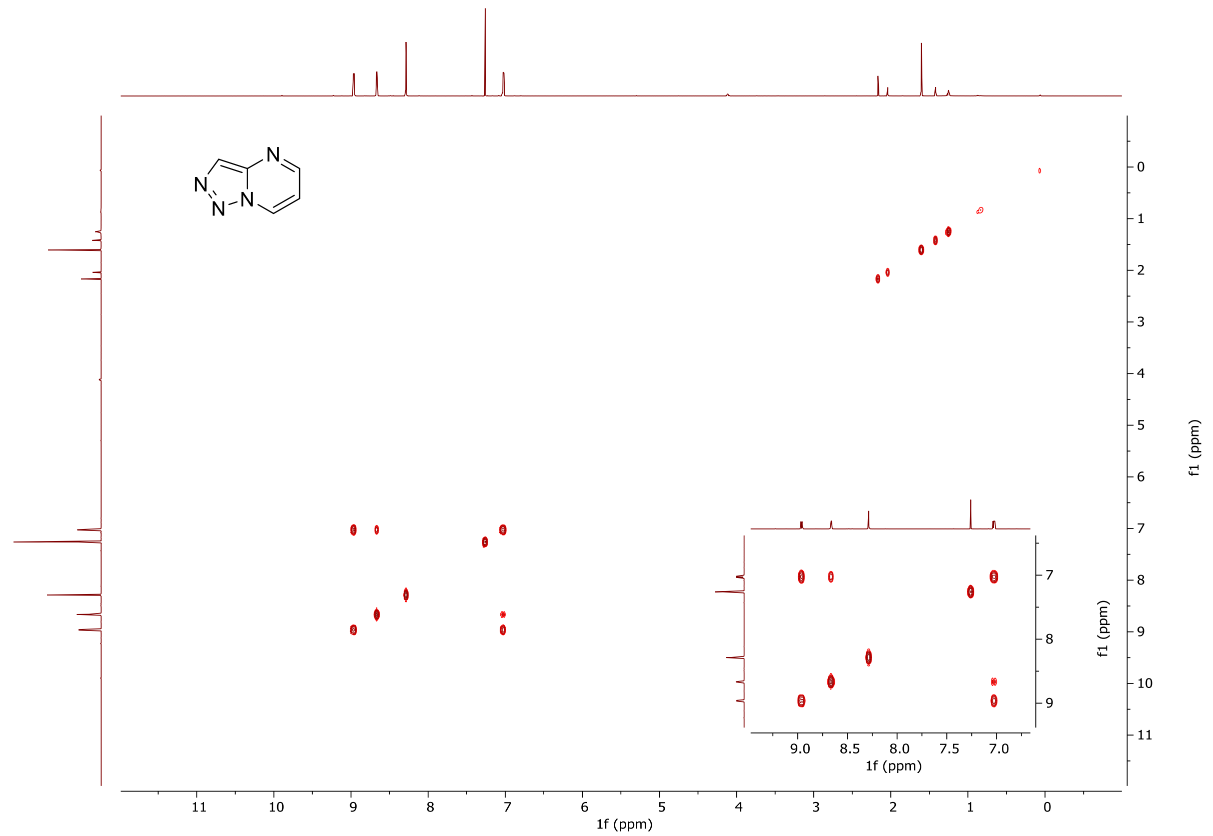

Fig. S278 COSY (501 MHz, 501 MHz, chloroform-*d*, 298 K) spectrum of compound **3l**

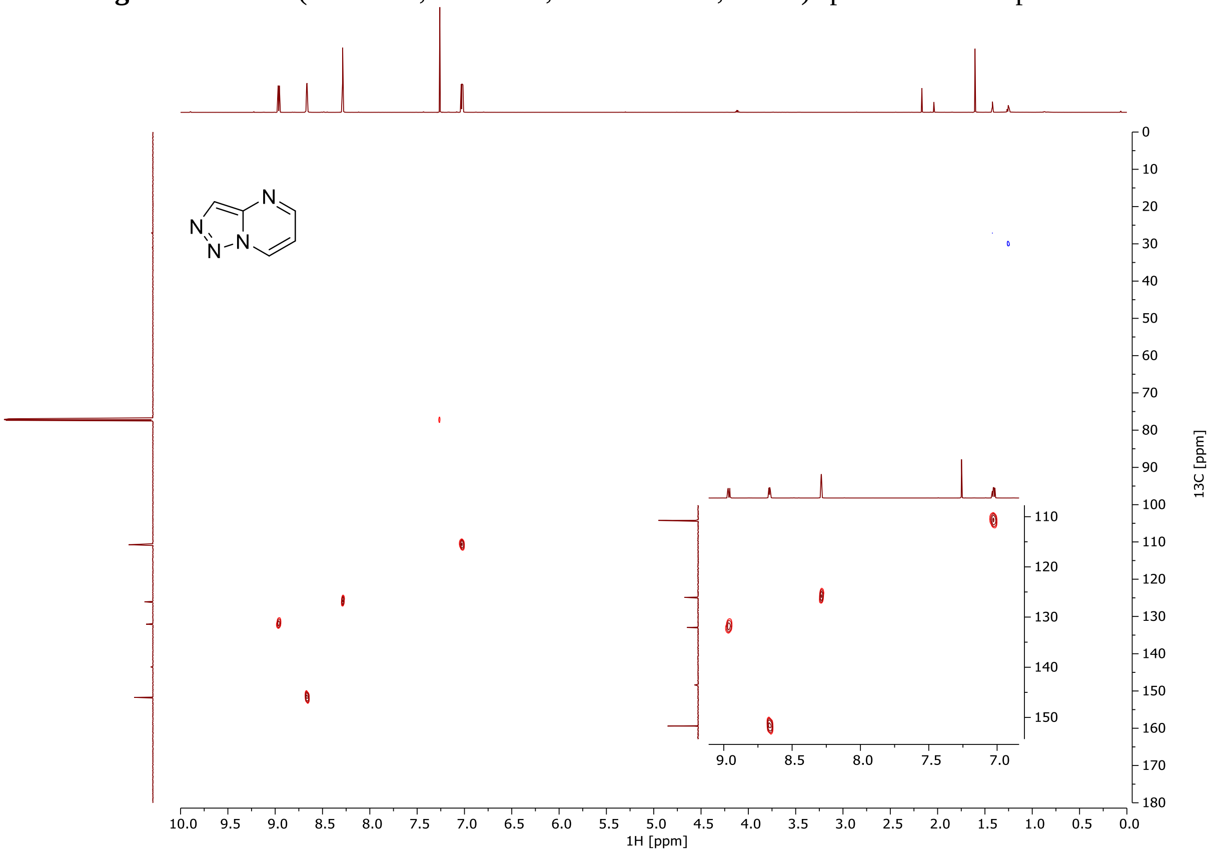

Fig. S279 HSQC (501 MHz, 126 MHz, chloroform-*d*, 298 K) spectrum of compound **3l**

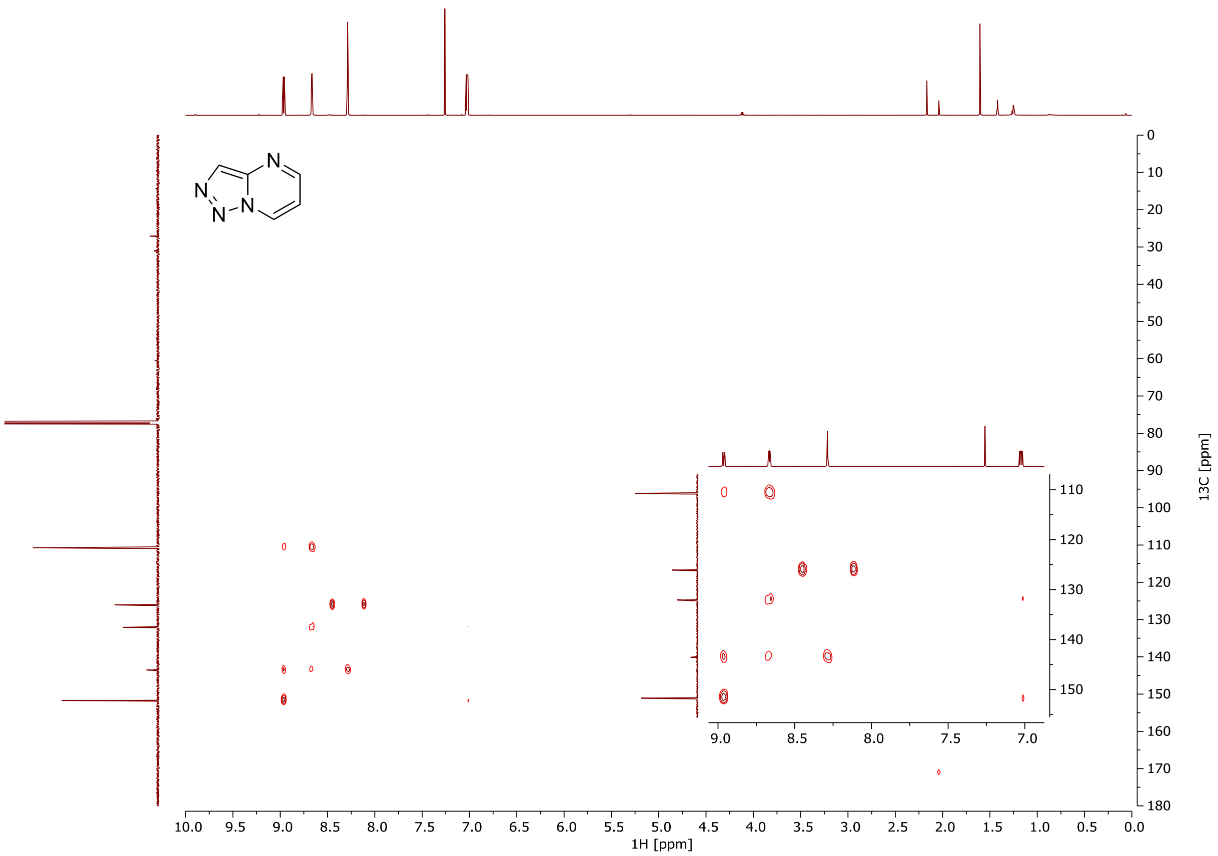

Fig. S280 HMBC (501 MHz, 126 MHz, chloroform-*d*, 298 K) spectrum of compound **3l**

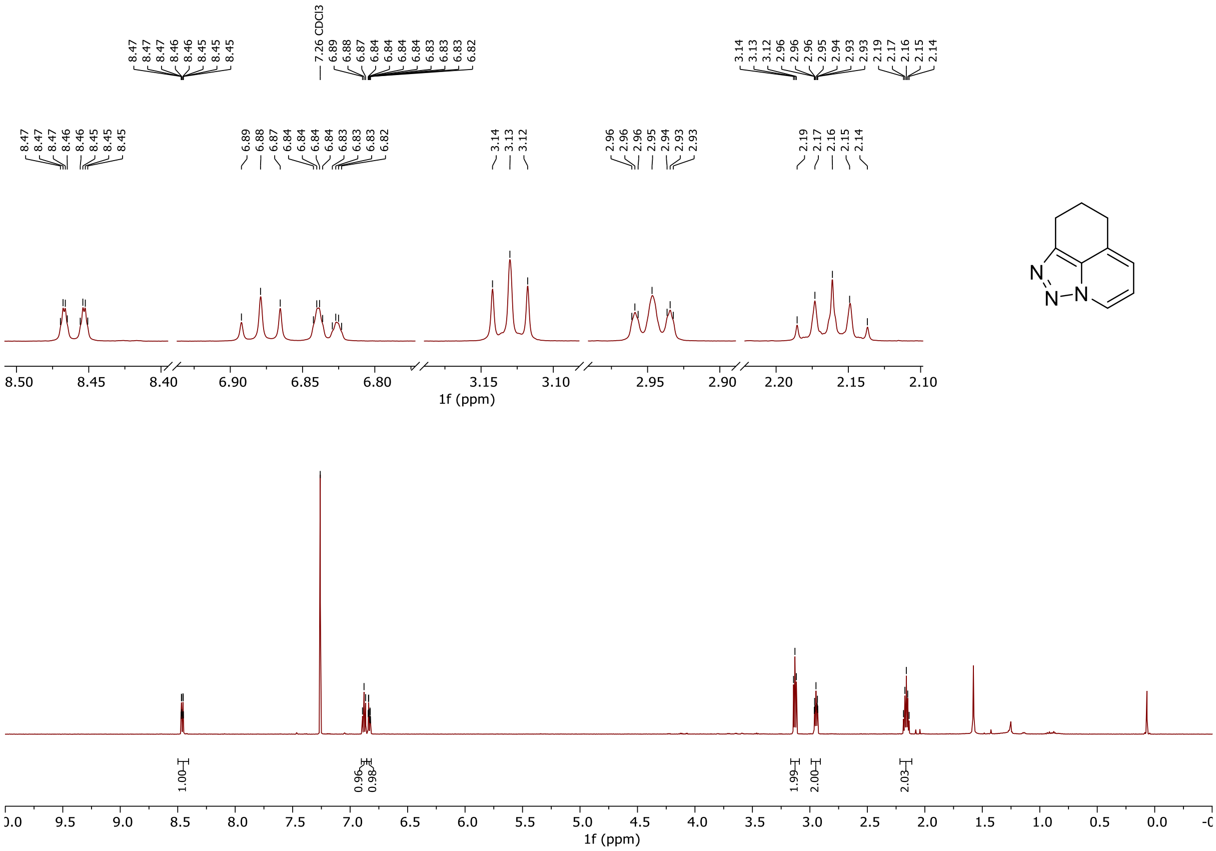

Fig. S281 ^{1}H NMR (501 MHz, chloroform-*d*, 298 K) spectrum of compound **3m**

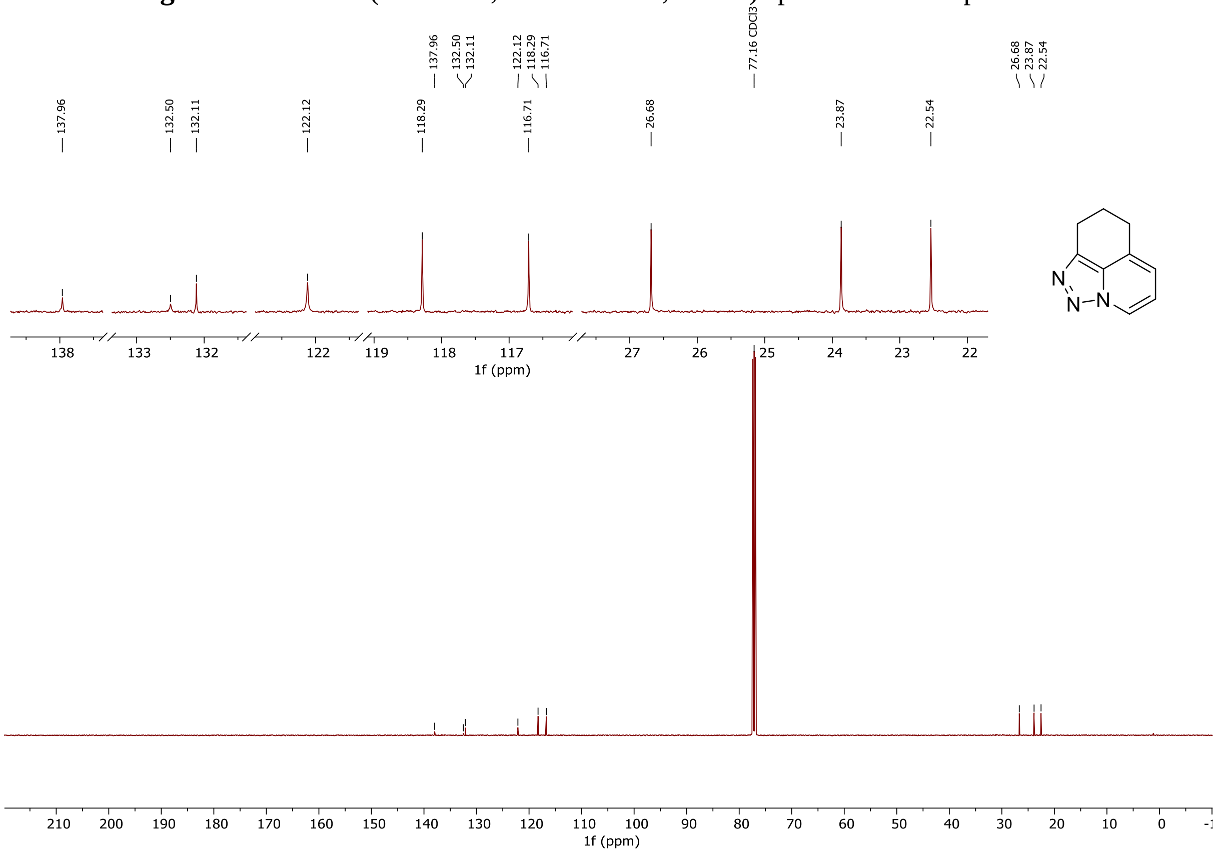

Fig. S282 $^{13}C\{^{1}H\}$ NMR (126 MHz, chloroform-*d*, 298 K) spectrum of compound **3m**

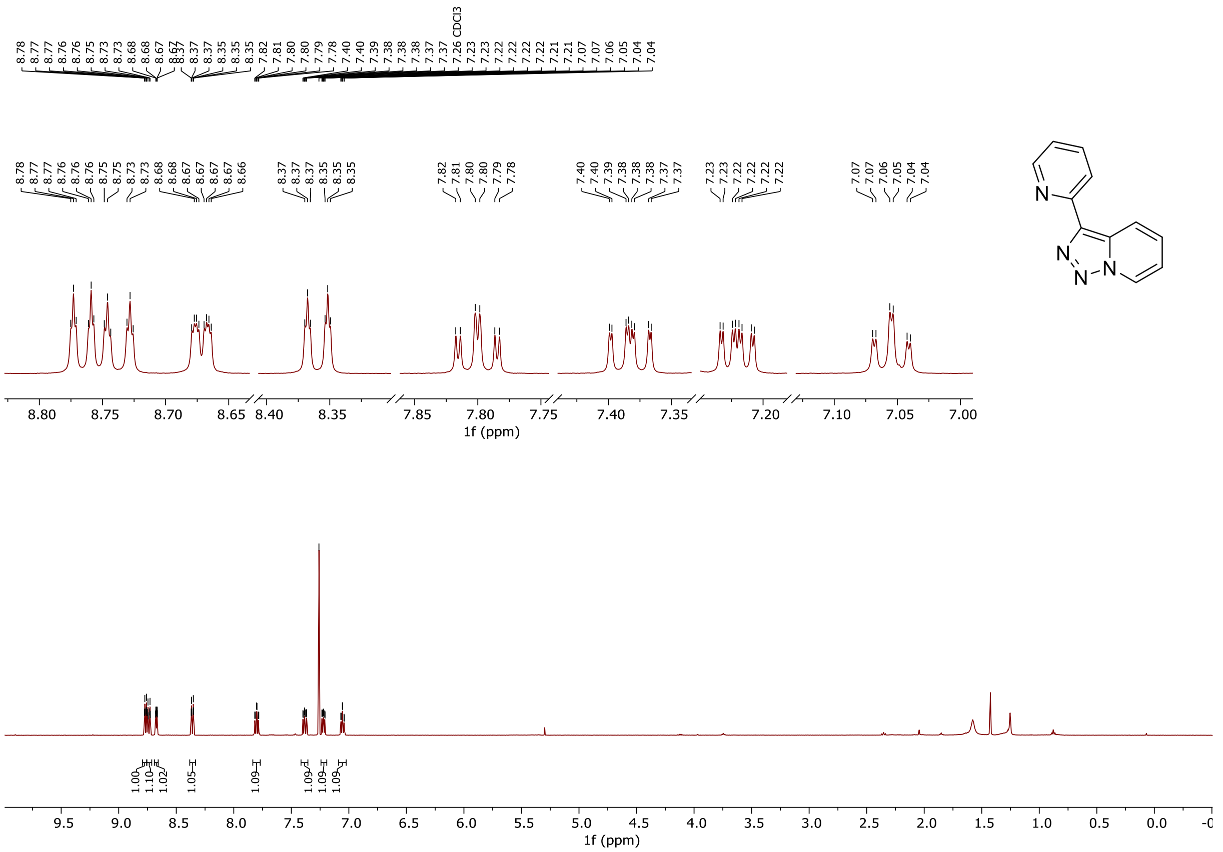

Fig. S283 ^{1}H NMR (501 MHz, chloroform-*d*, 298 K) spectrum of compound **3n**

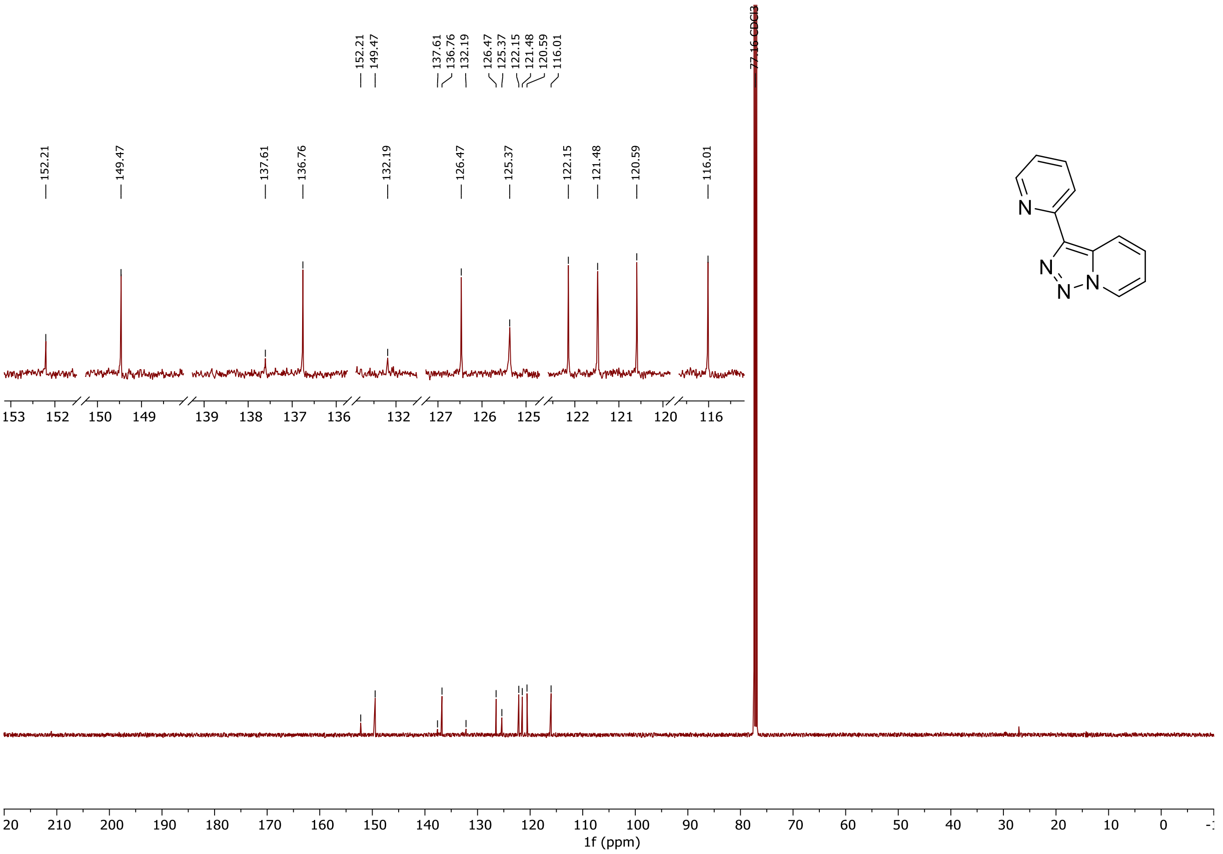

Fig. S284 ^{13}C{^{1}H} NMR of (126 MHz, chloroform-*d*, 298 K) spectrum of compound **3n**

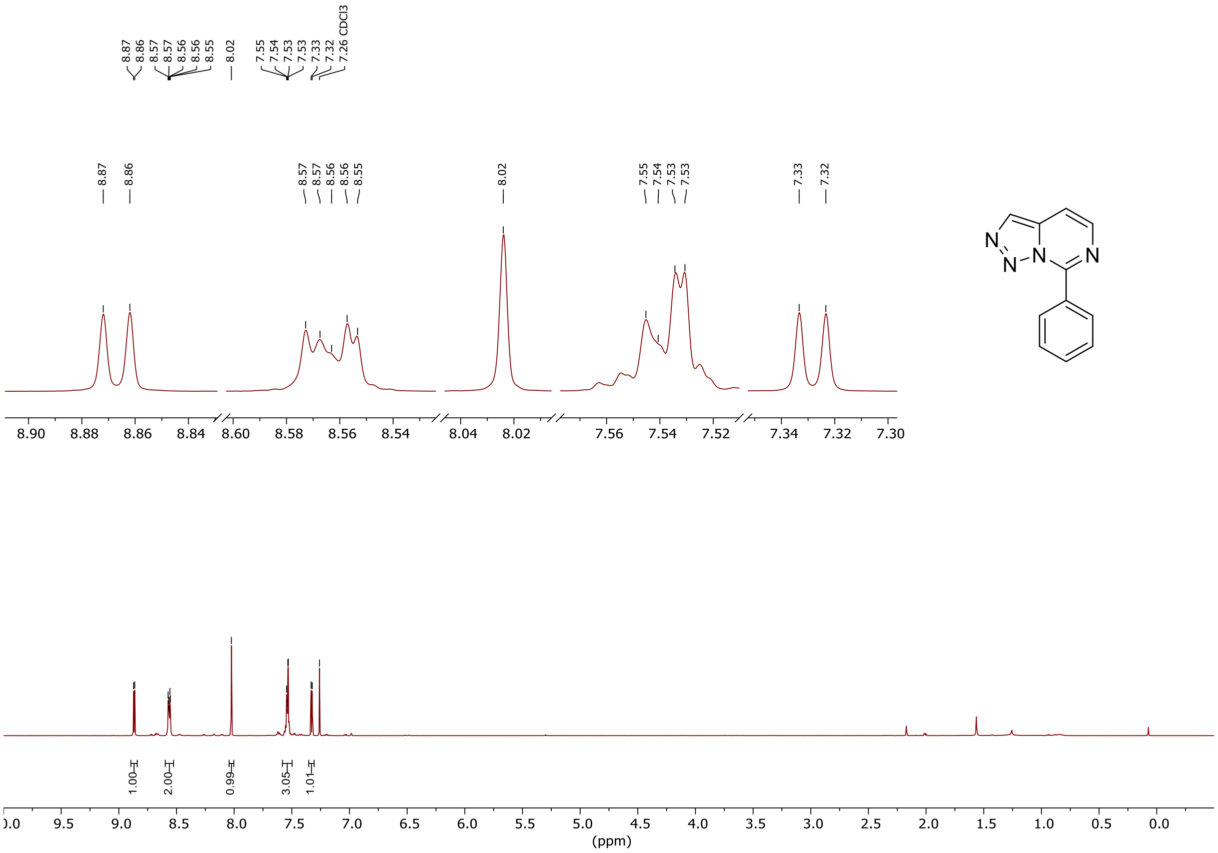

Fig. S285 ^{1}H NMR (501 MHz, chloroform-*d*, 298 K) spectrum of compound **3o**

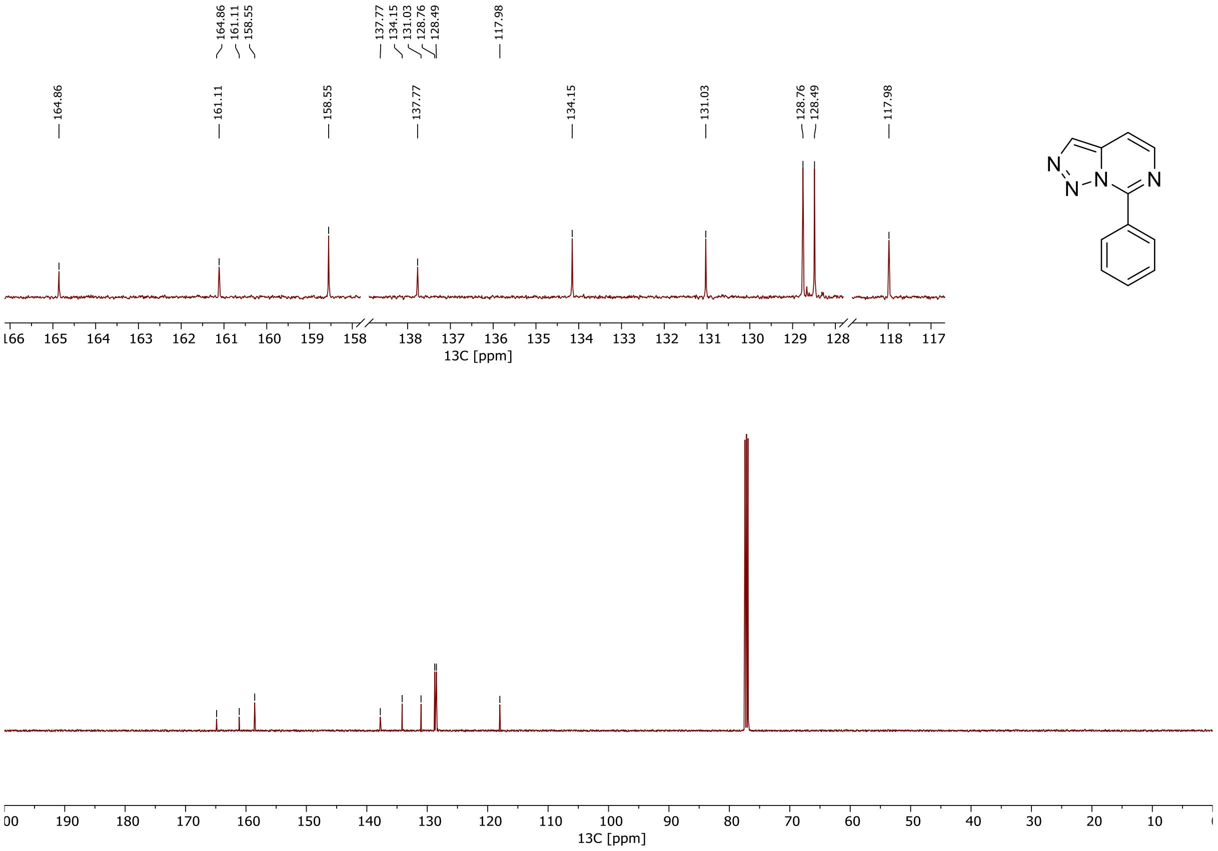

Fig. S286 $^{13}C\{^{1}H\}$ NMR (126 MHz, chloroform-*d*, 298 K) spectrum of compound of **3o**

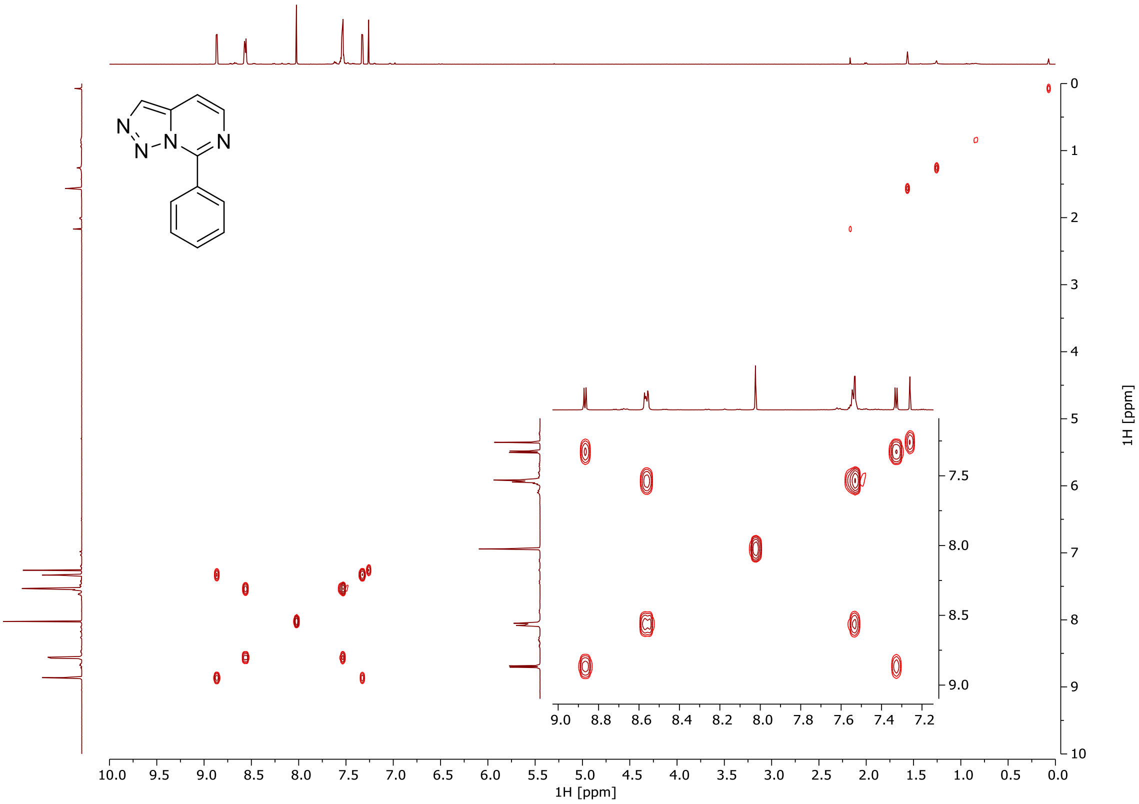

Fig. S287 COSY (501 MHz, 501 MHz, chloroform-*d*, 298 K) spectrum of compound **3o**

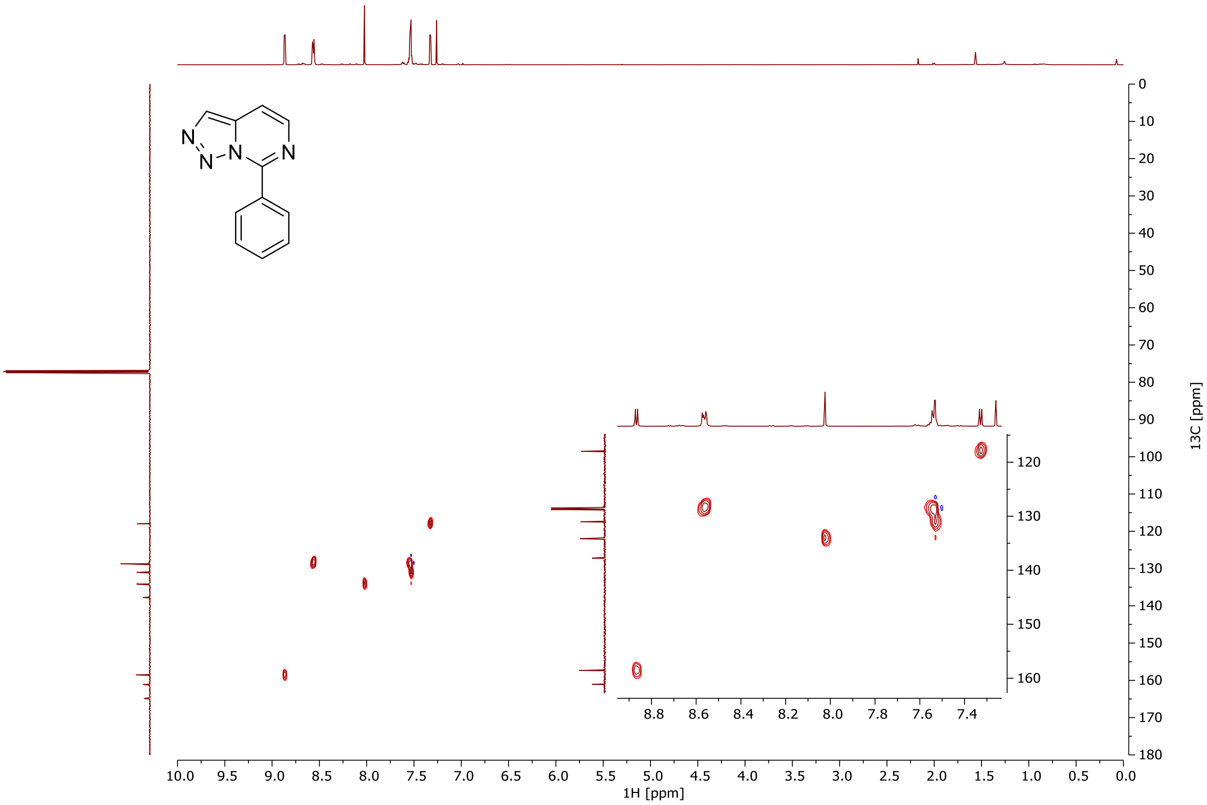

Fig. S288 HSQC (501 MHz, 126 MHz, chloroform-*d*, 298 K) spectrum of compound **3o**

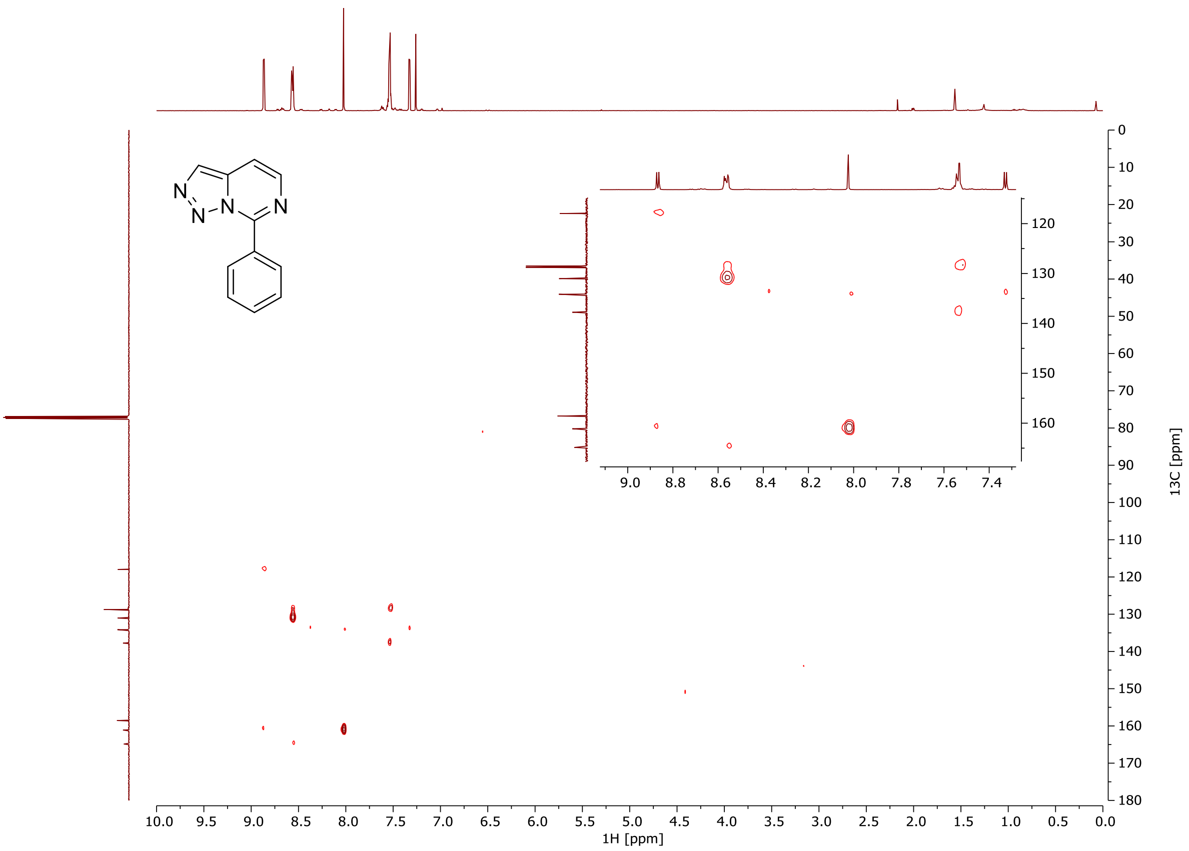

Fig. S289 HMBC (501 MHz, 126 MHz, chloroform-*d*, 298 K) spectrum of compound **3o**

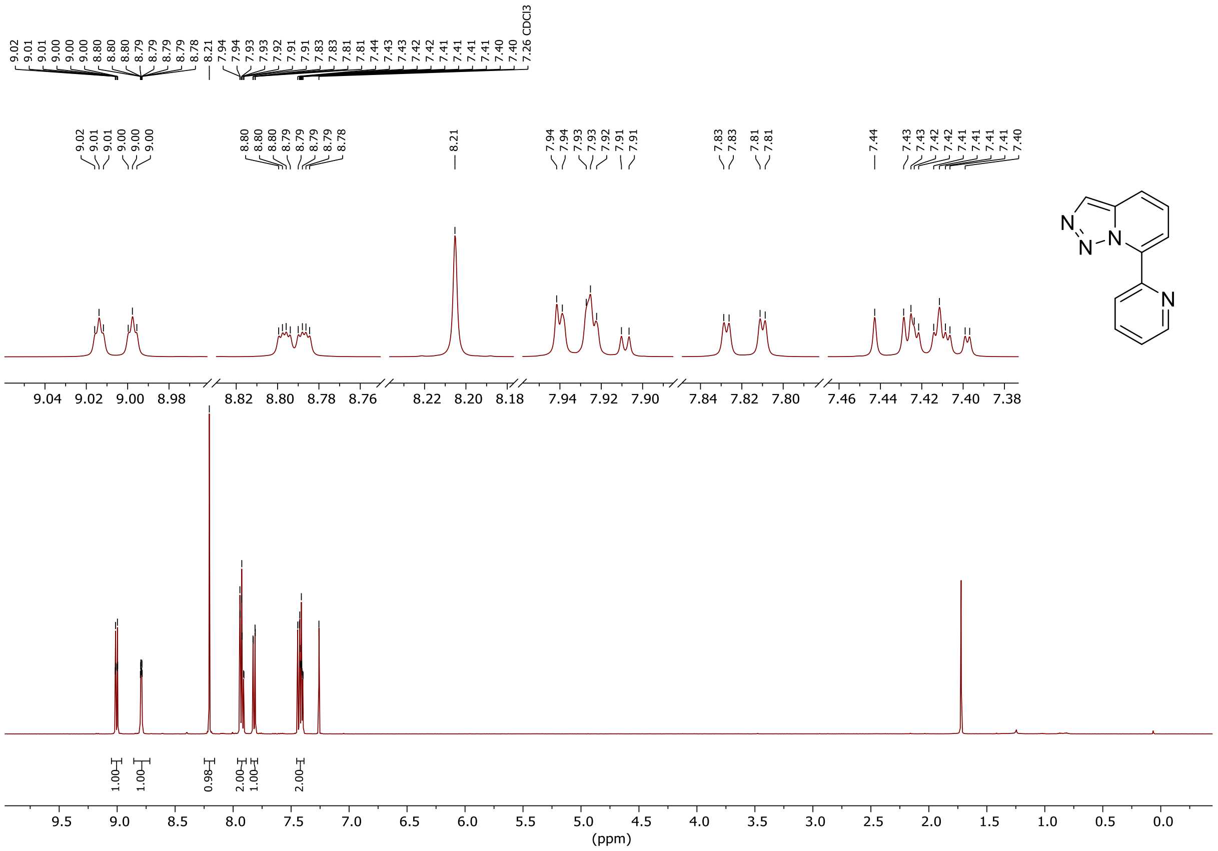

Fig. S290 ^{1}H NMR (501 MHz, chloroform-*d*, 298 K) spectrum of compound **3p**

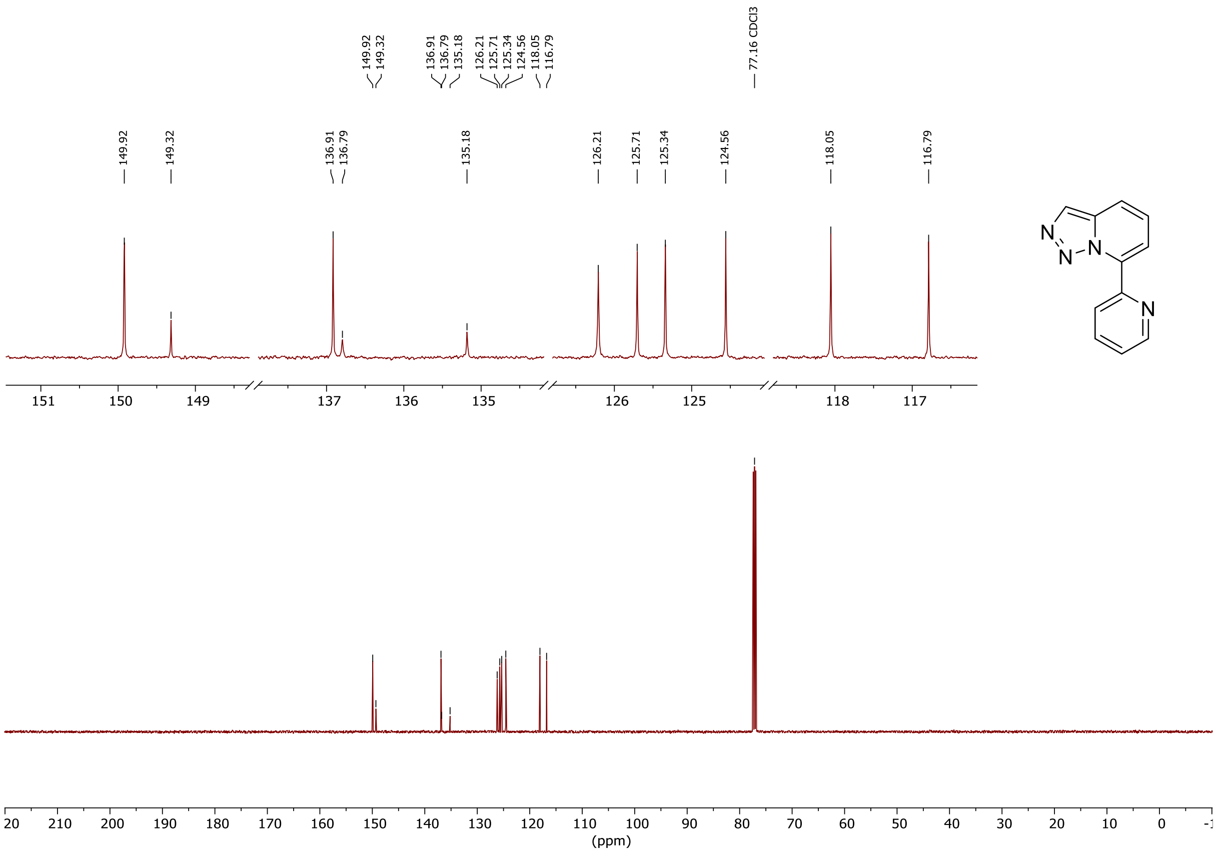

Fig. S291 $^{13}C\{^{1}H\}$ NMR (501 MHz, chloroform-*d*, 298 K) spectrum of compound **3p**

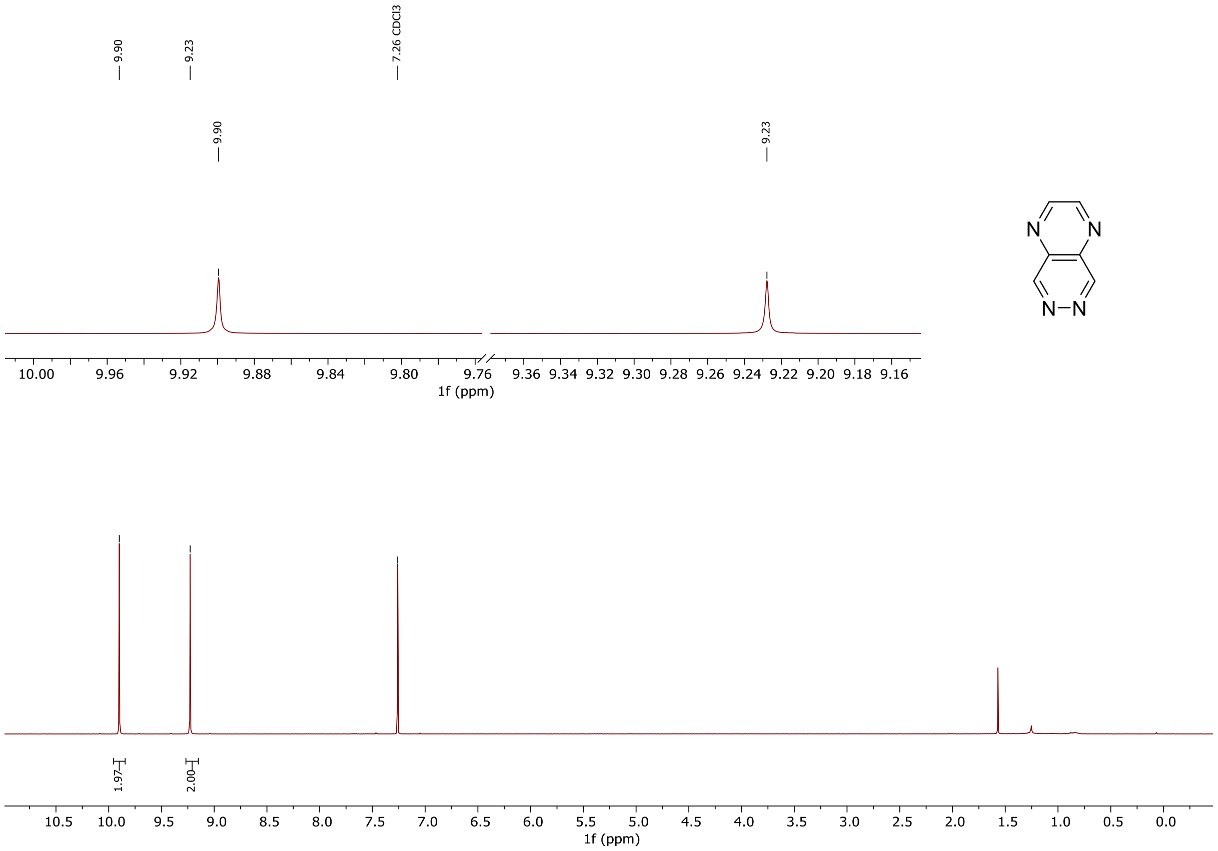

Fig. S292 ^{1}H NMR (501 MHz, chloroform-*d*, 298 K) spectrum of compound **5a**

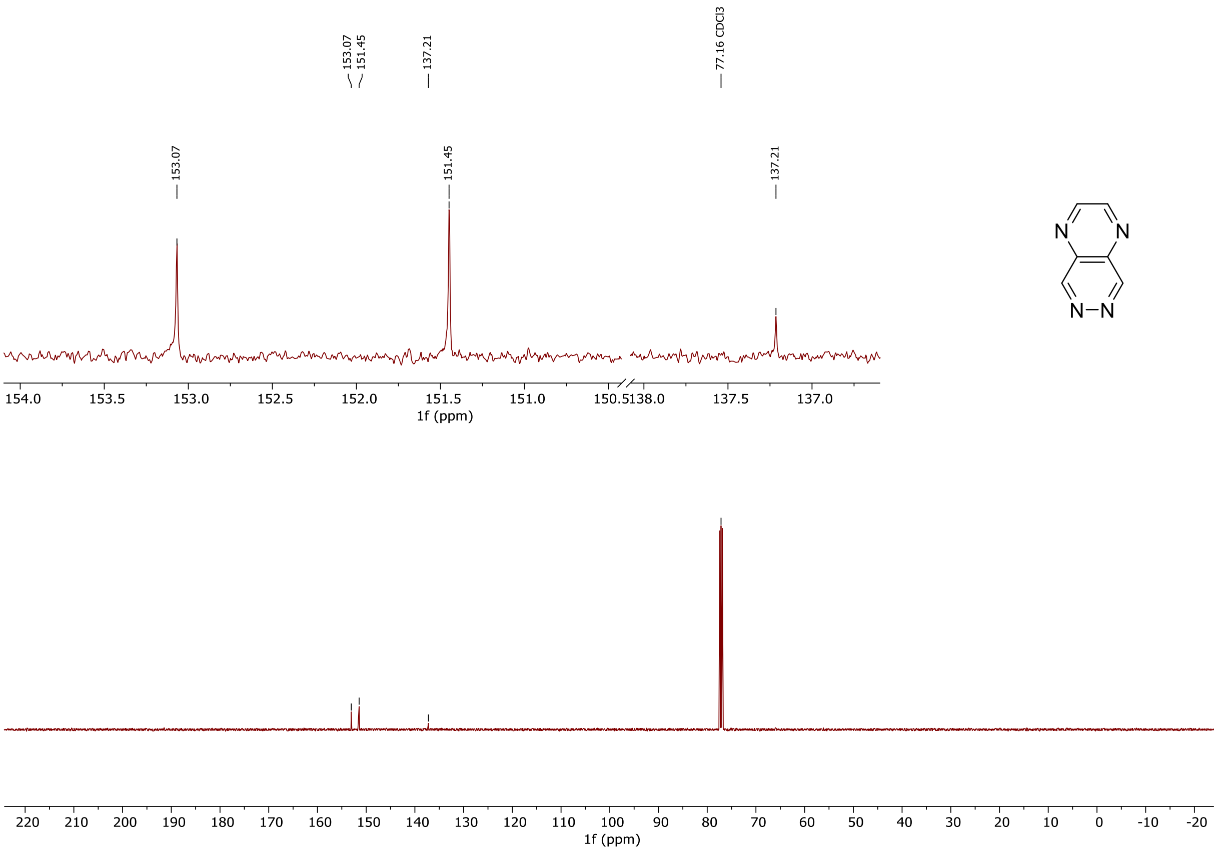

Fig. S293 $^{13}C\{^{1}H\}$ NMR (126 MHz, chloroform-*d*, 298 K) spectrum of compound **5a**

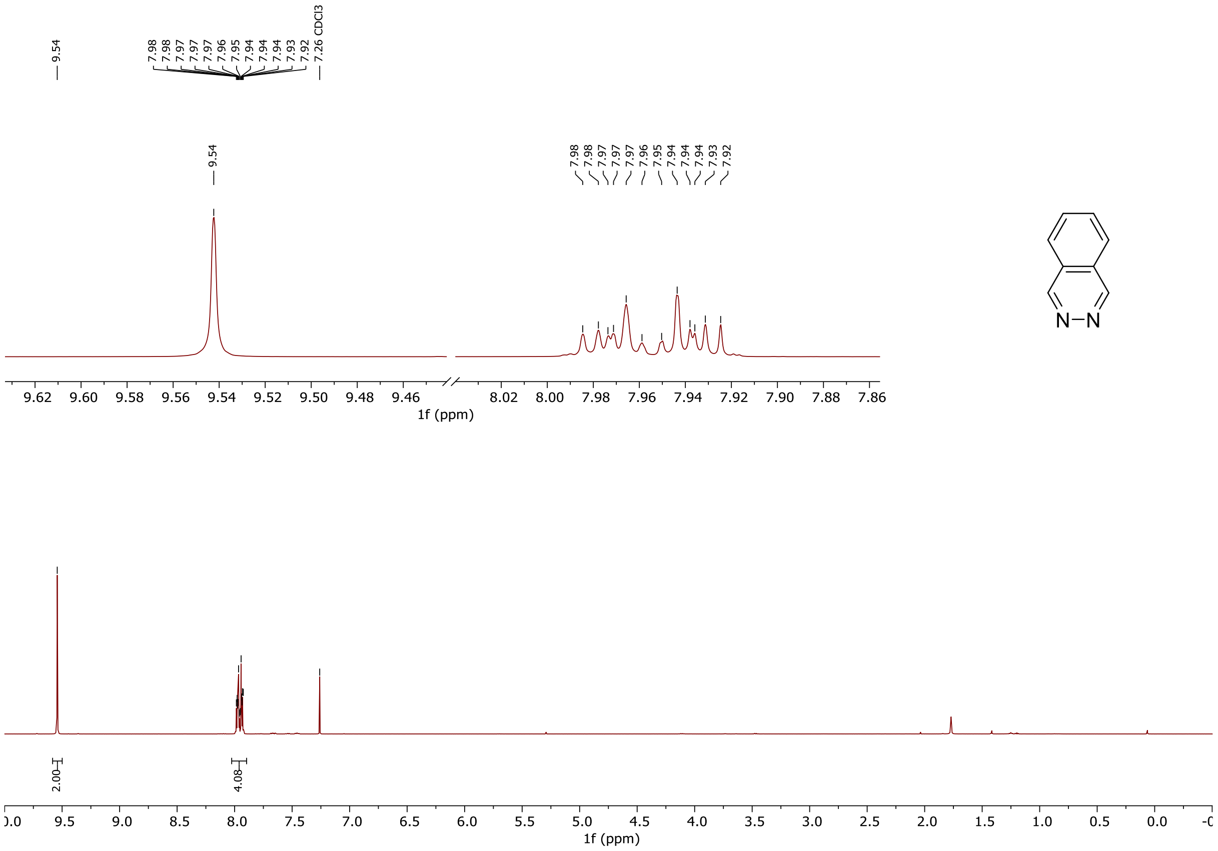

Fig. S294 ^{1}H NMR (501 MHz, chloroform-*d*, 298 K) spectrum of compound **5b**

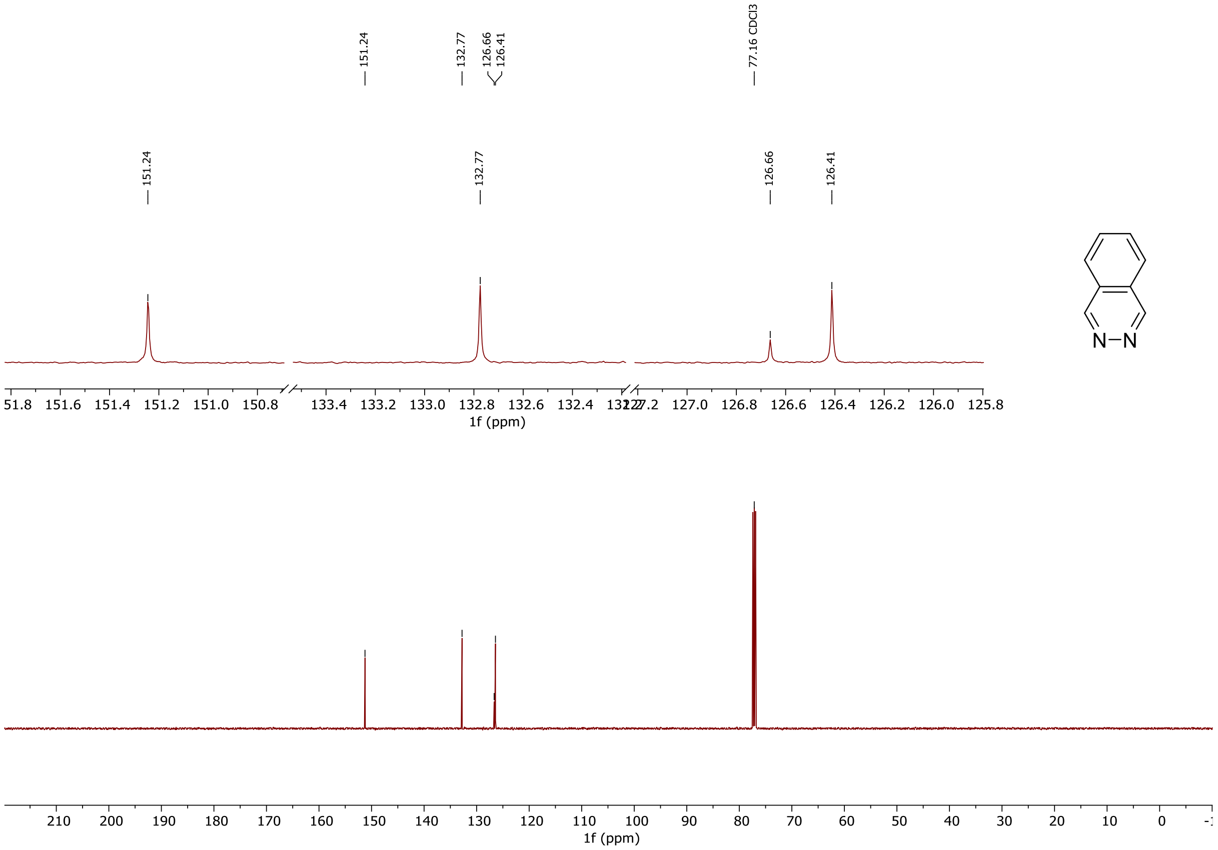

Fig. S295 ^{13}C{^{1}H} NMR (126 MHz, chloroform-*d*, 298 K) spectrum of compound **5b**

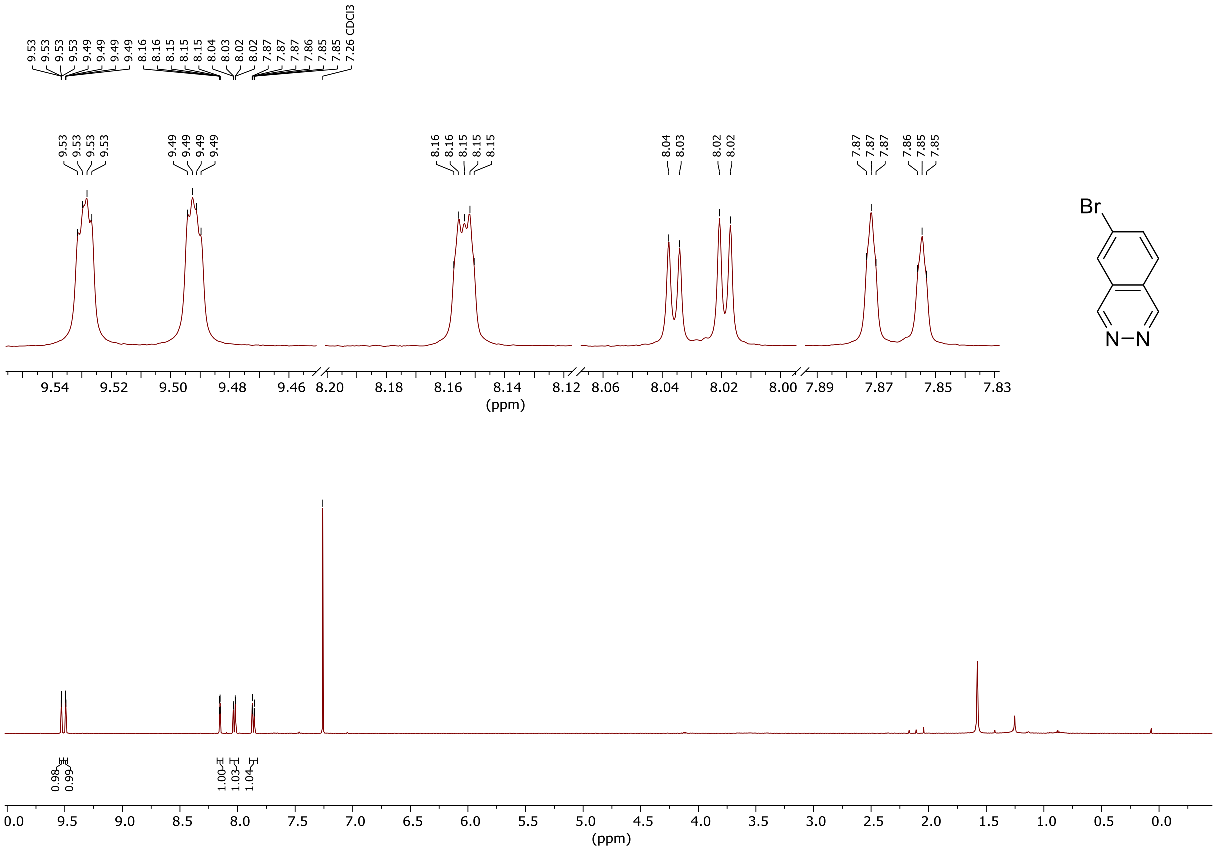

Fig. S296 ^{1}H NMR of (501 MHz, chloroform-*d*, 298 K) spectrum of compound **5c**

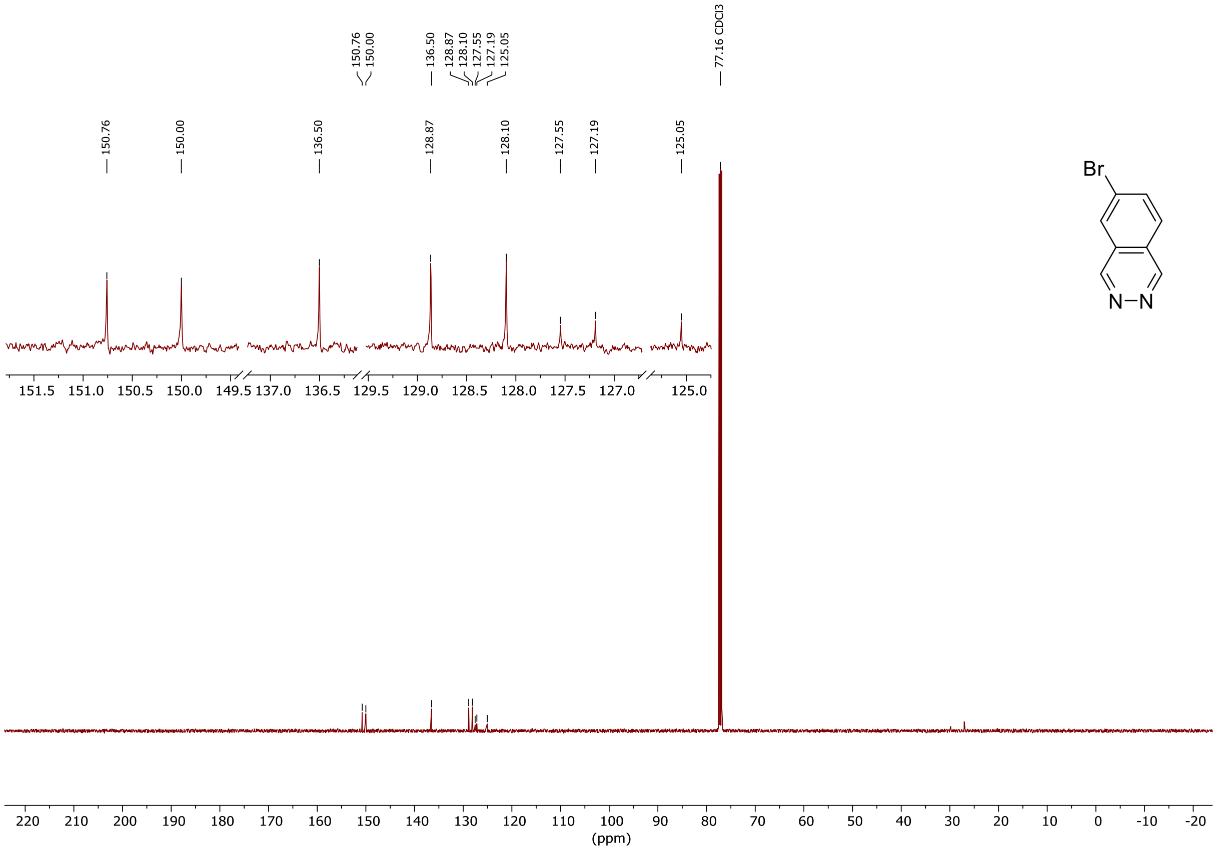

Fig. S297 ^{13}C{^{1}H} NMR of (126 MHz, chloroform-*d*, 298 K) spectrum of compound **5c**

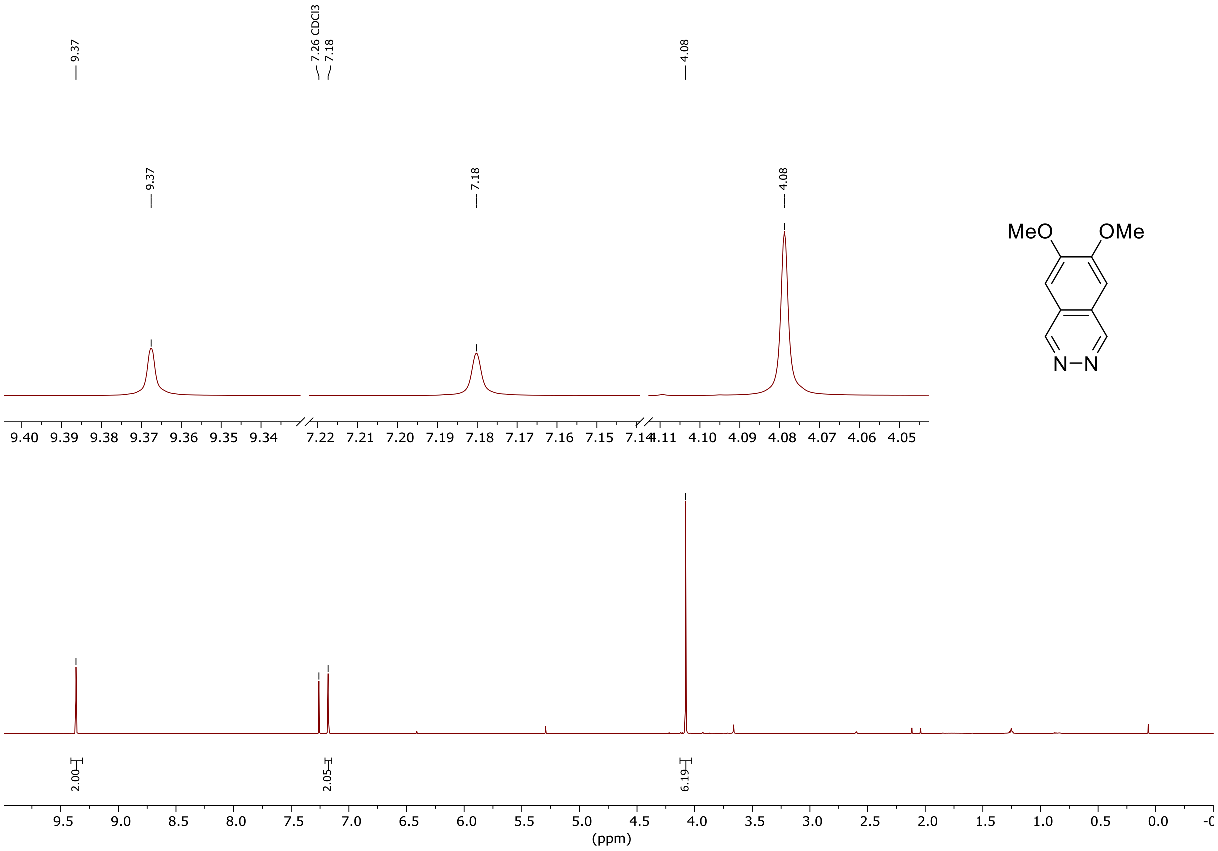

Fig. S298 ^{1}H NMR (501 MHz, chloroform-*d*, 298 K) spectrum of compound **5d**

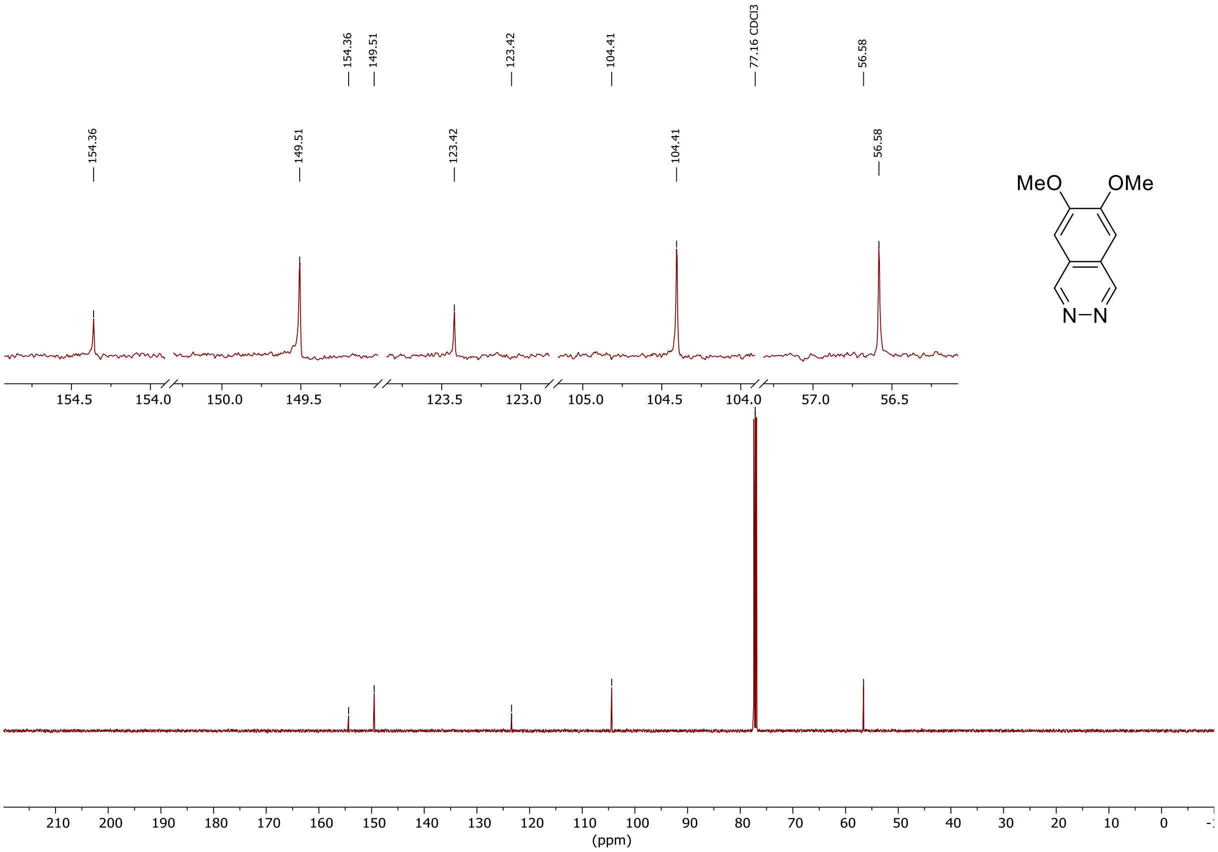

Fig. S299 ^{13}C{^{1}H} (126 MHz, chloroform-*d*, 298 K) spectrum of compound **5d**

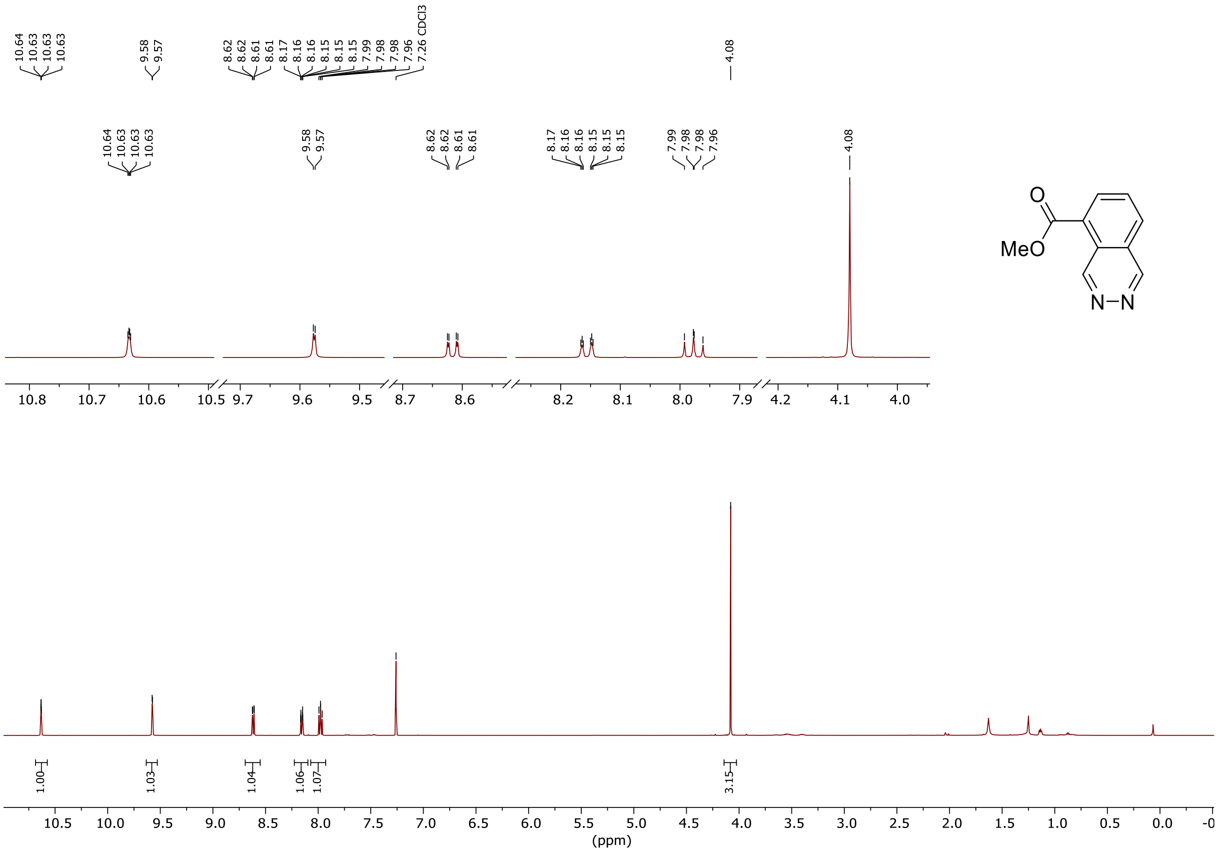

Fig. S300 ^{1}H NMR (501 MHz, chloroform-*d*, 298 K) spectrum of compound **5e**

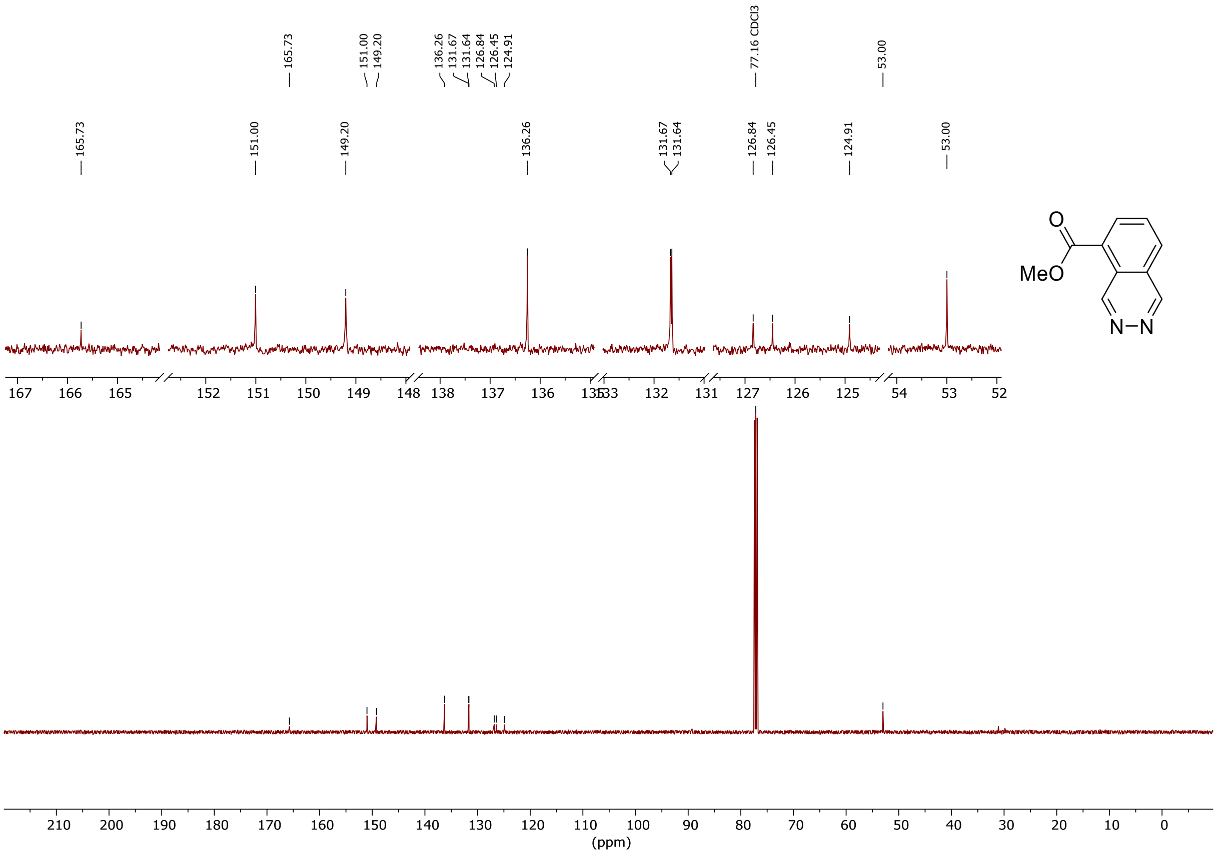

Fig. S301 ^{13}C{^{1}H} NMR (126 MHz, chloroform-*d*, 298 K) spectrum of compound **5e**

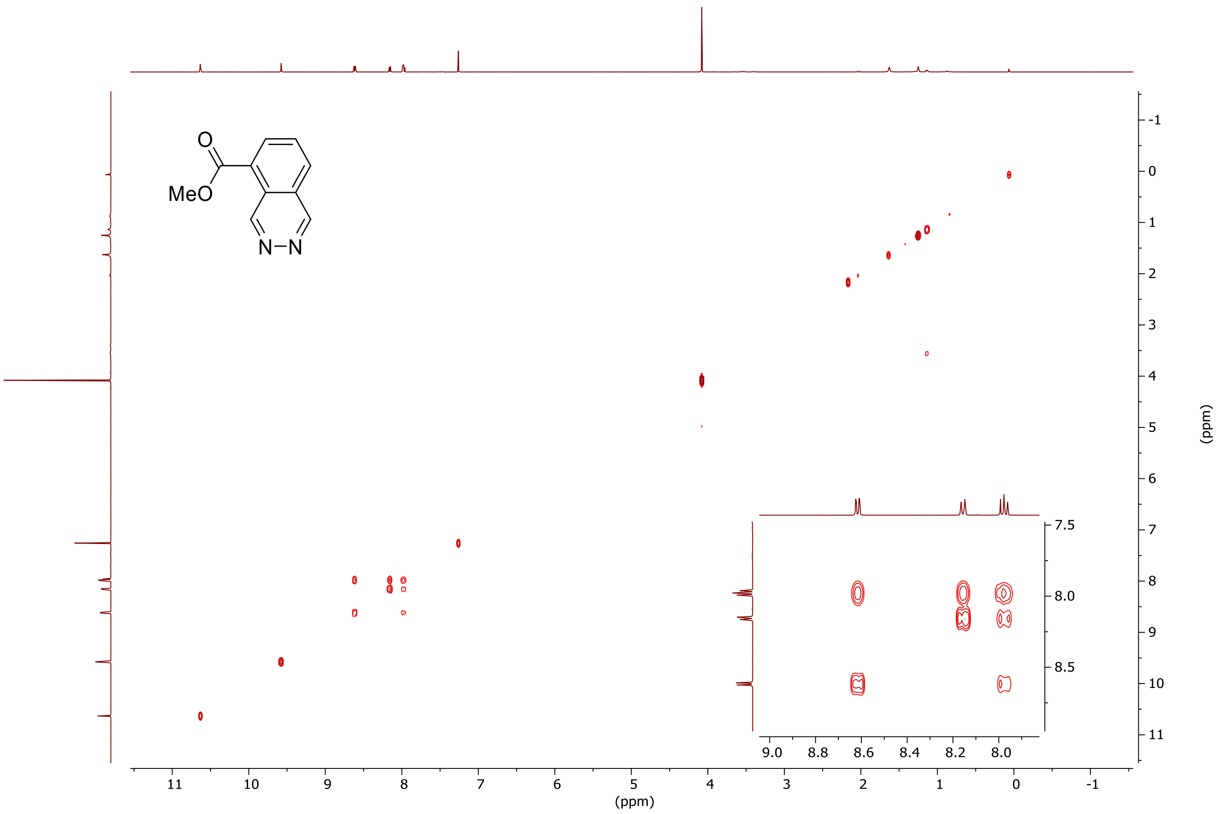

Fig. S302 COSY (501 MHz, 501 MHz, chloroform-*d*, 298 K) spectrum of compound **5e**

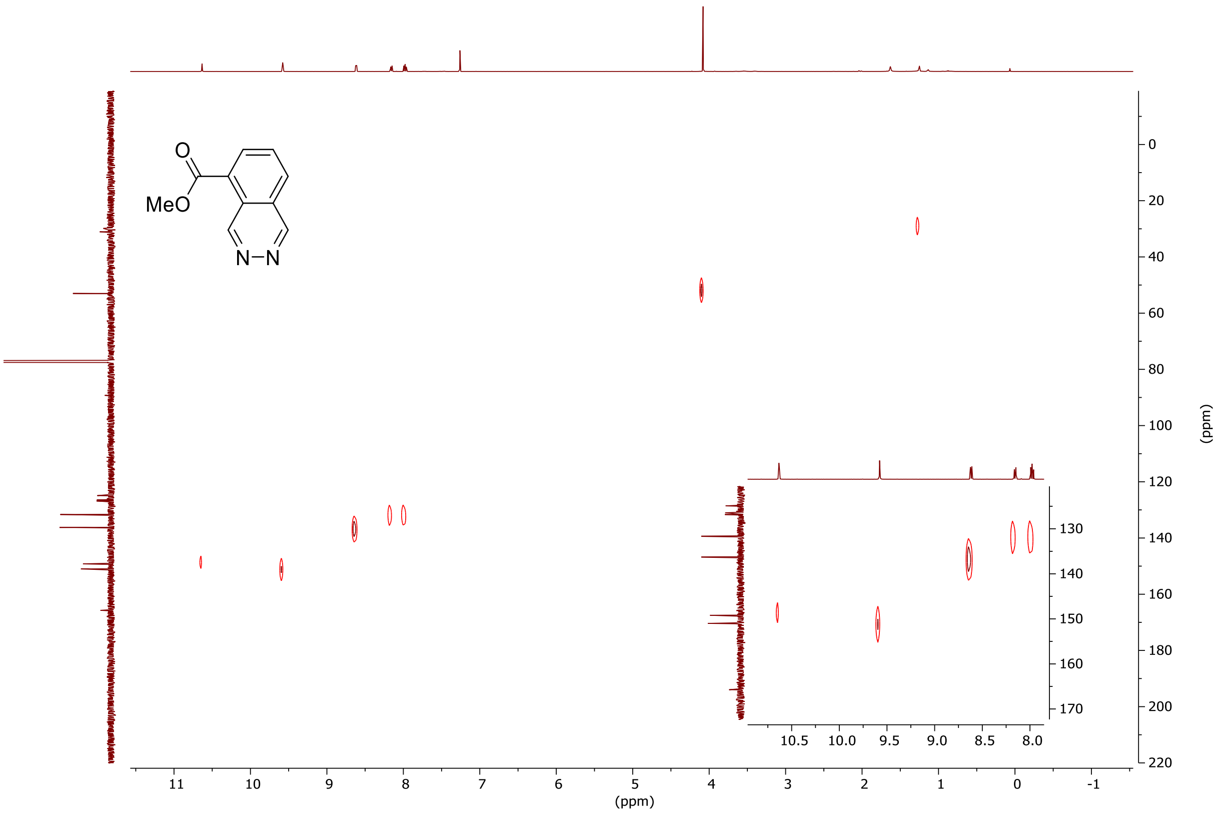

Fig. S303 HSQC (501 MHz, 126 MHz, chloroform-*d*, 298 K) spectrum of compound **5e**

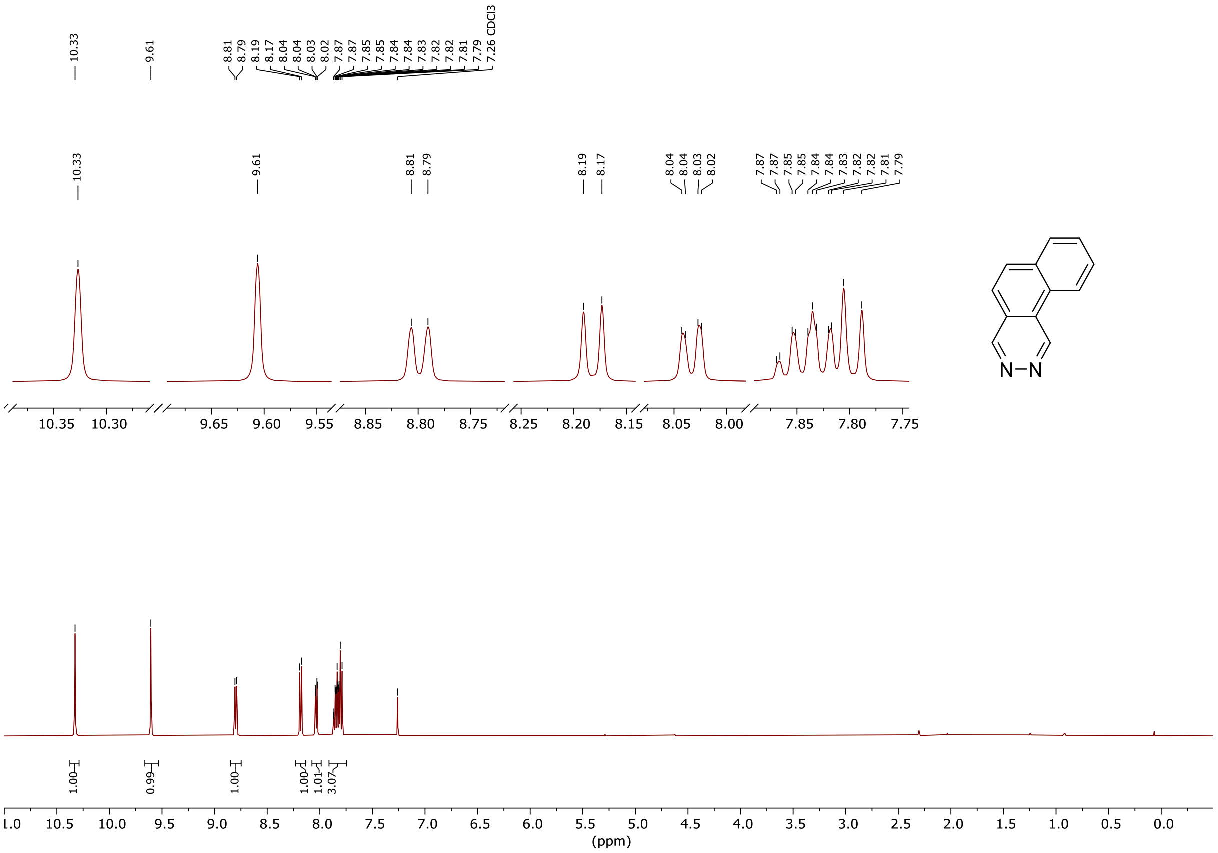

Fig. S304 ^{1}H NMR (501 MHz, chloroform-*d*, 298 K) spectrum of compound **5f**

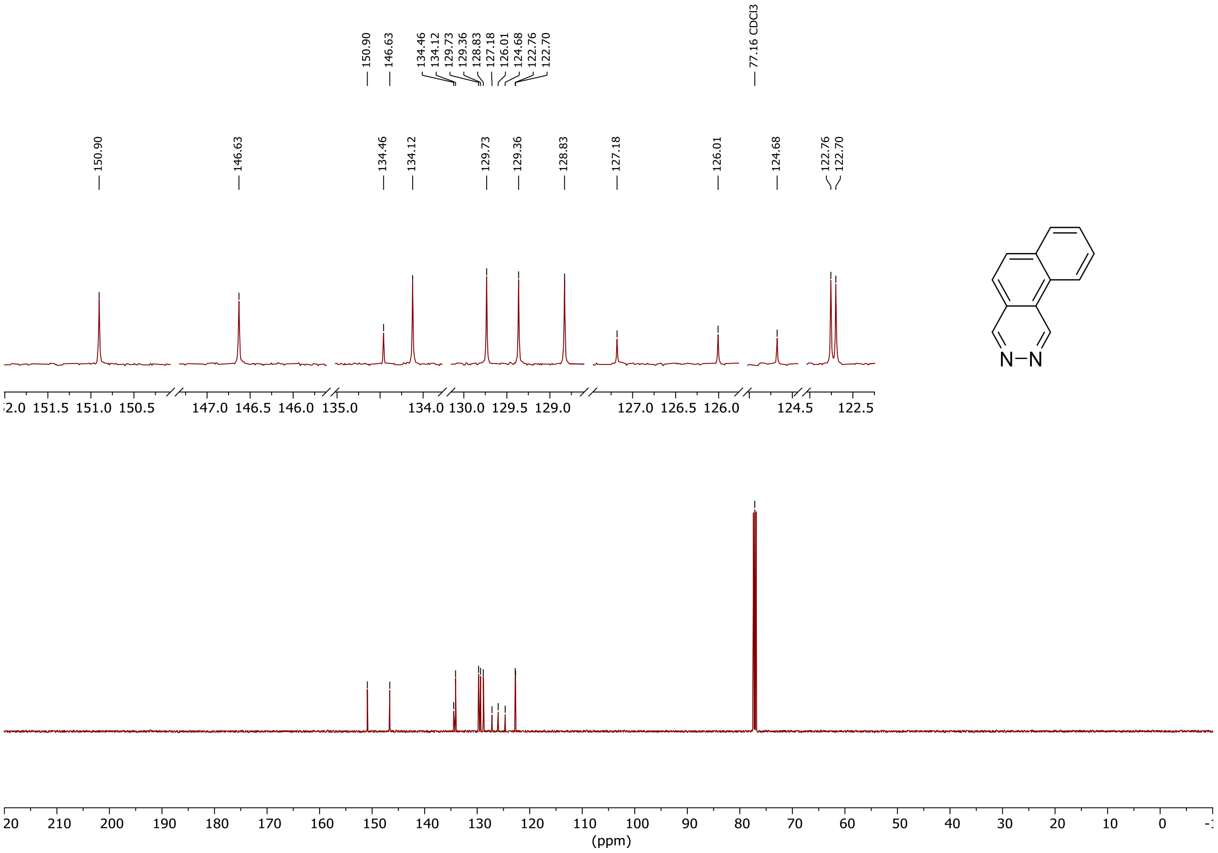

Fig. S305 ^{13}C{^{1}H} NMR (126 MHz, chloroform-*d*, 298 K) spectrum of compound **5f**

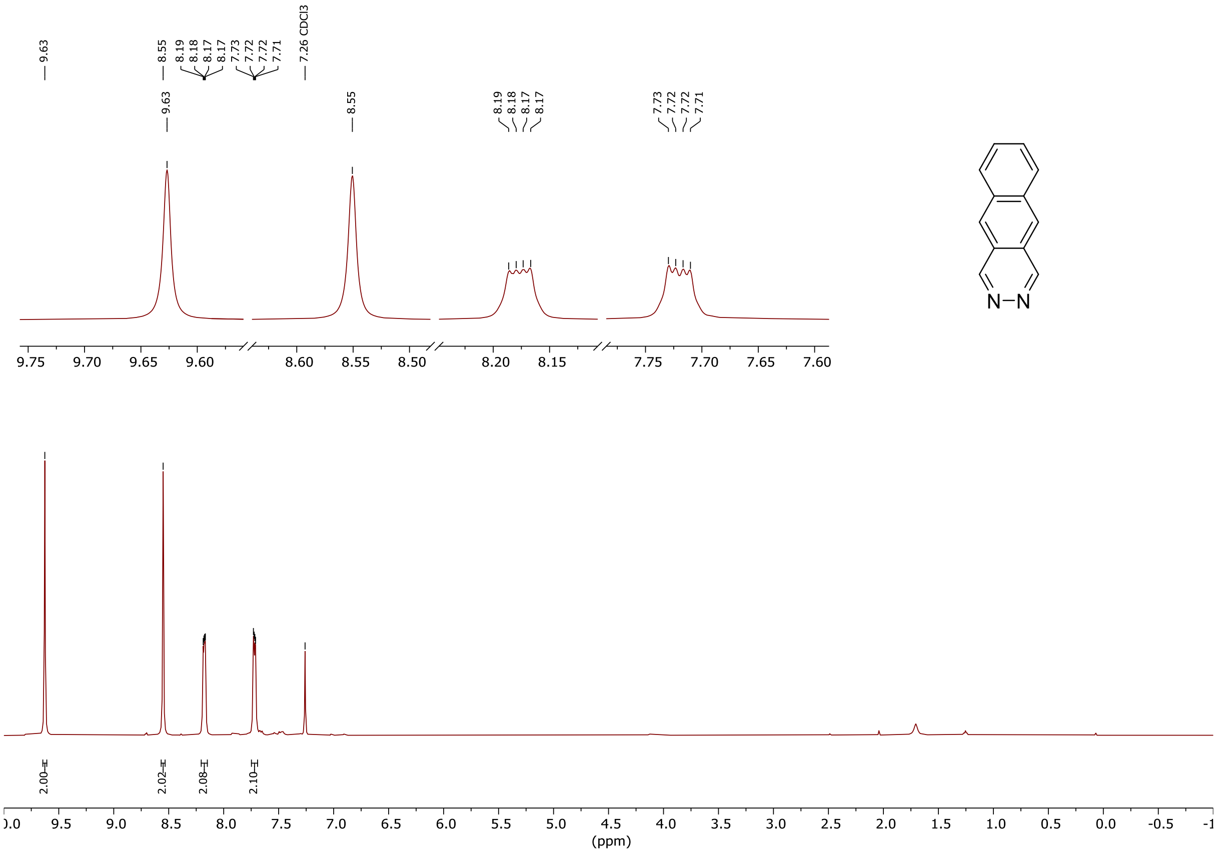

Fig. S306 ^{1}H NMR (501 MHz, chloroform-*d*, 298 K) spectrum of compound **5g**

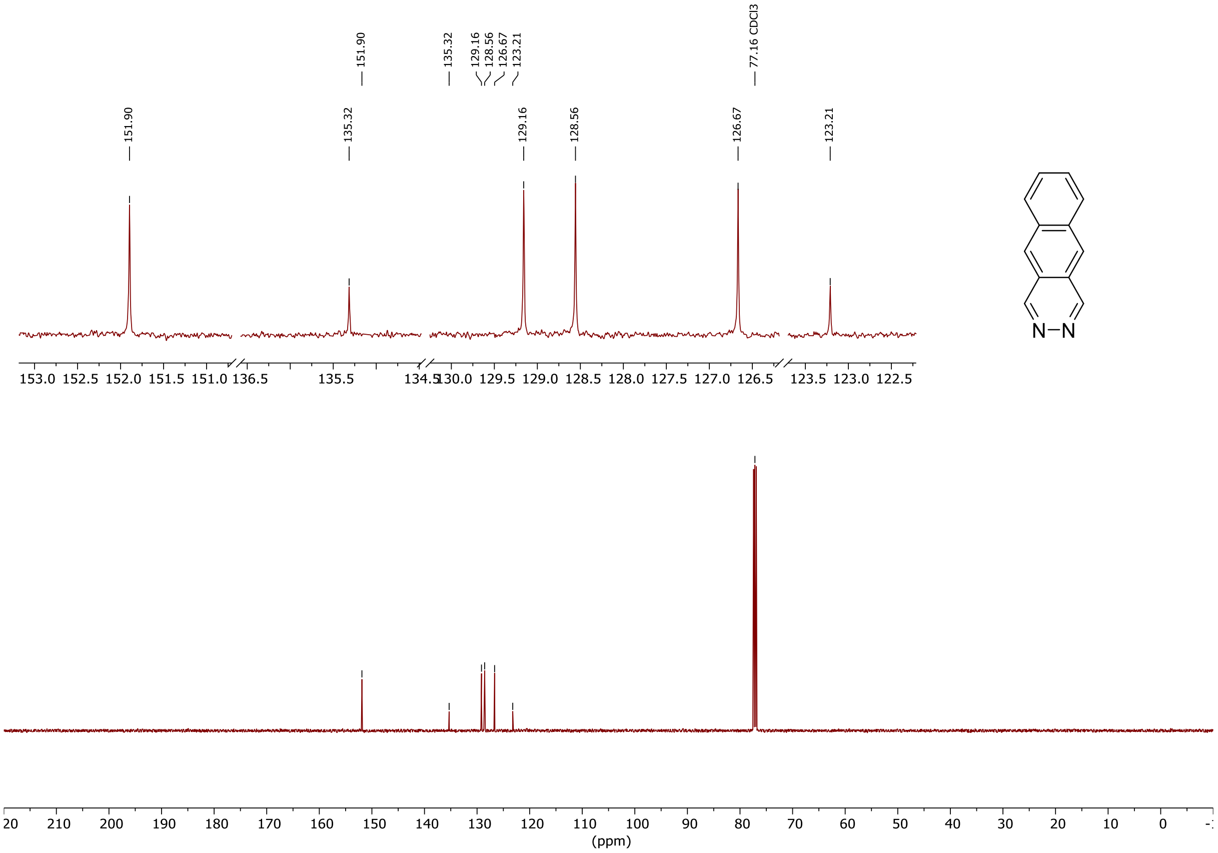

Fig. S307 ^{13}C{^{1}H} NMR (126 MHz, chloroform-*d*, 298 K) spectrum of compound **5g**

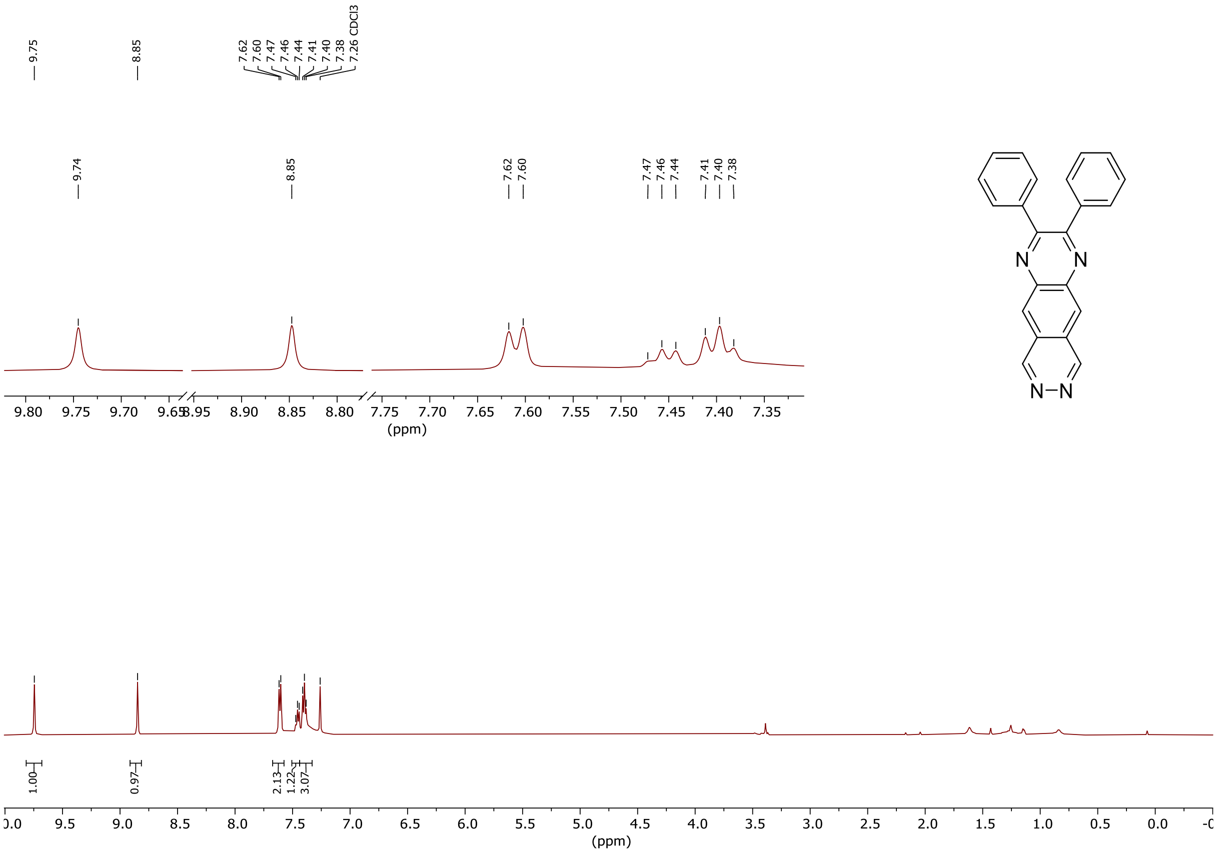

Fig. S308 ^{1}H NMR (501 MHz, chloroform-*d*, 298 K) spectrum of compound **5h**

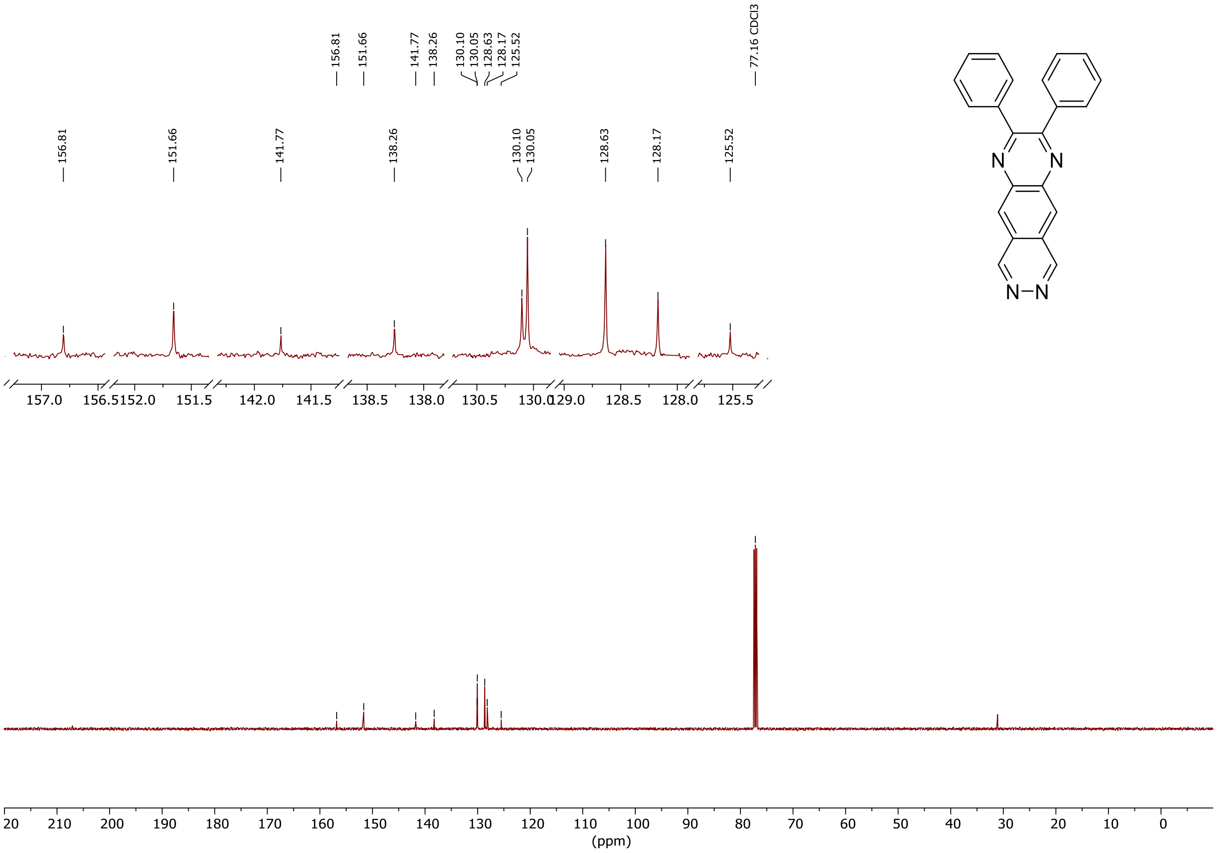

Fig. S309 ^{13}C{^{1}H} NMR (126 MHz, chloroform-*d*, 298 K) spectrum of compound **5h**

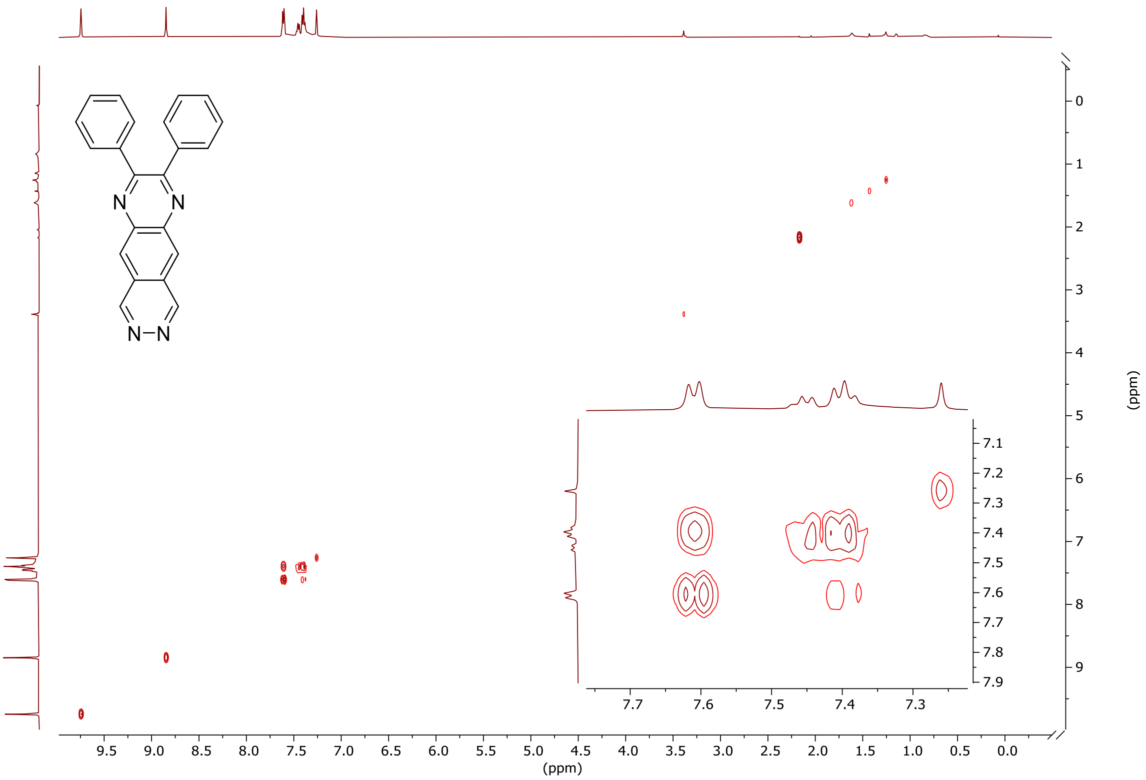

Fig. S310 COSY (501 MHz, 501 MHz, chloroform-*d*, 298 K) spectrum of compound **5h**

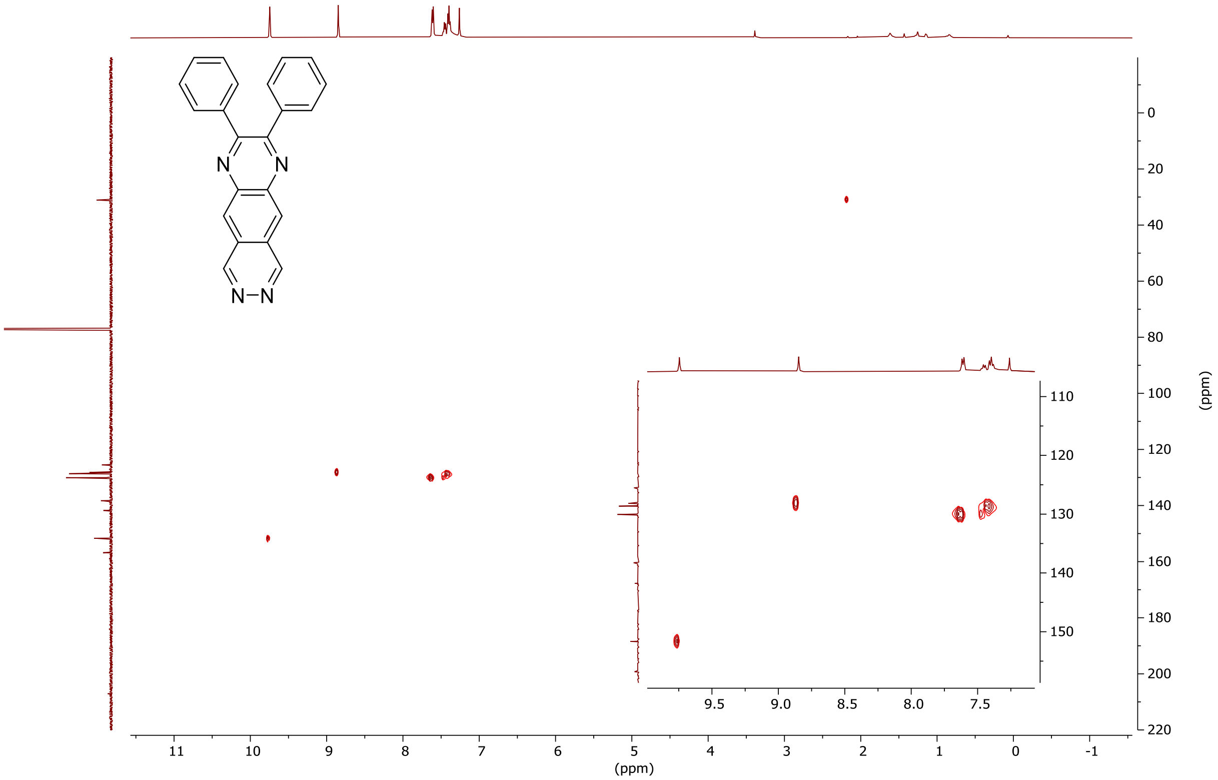

Fig. S311 HSQC (501 MHz, 126 MHz, chloroform-*d*, 298 K) spectrum of compound **5h**

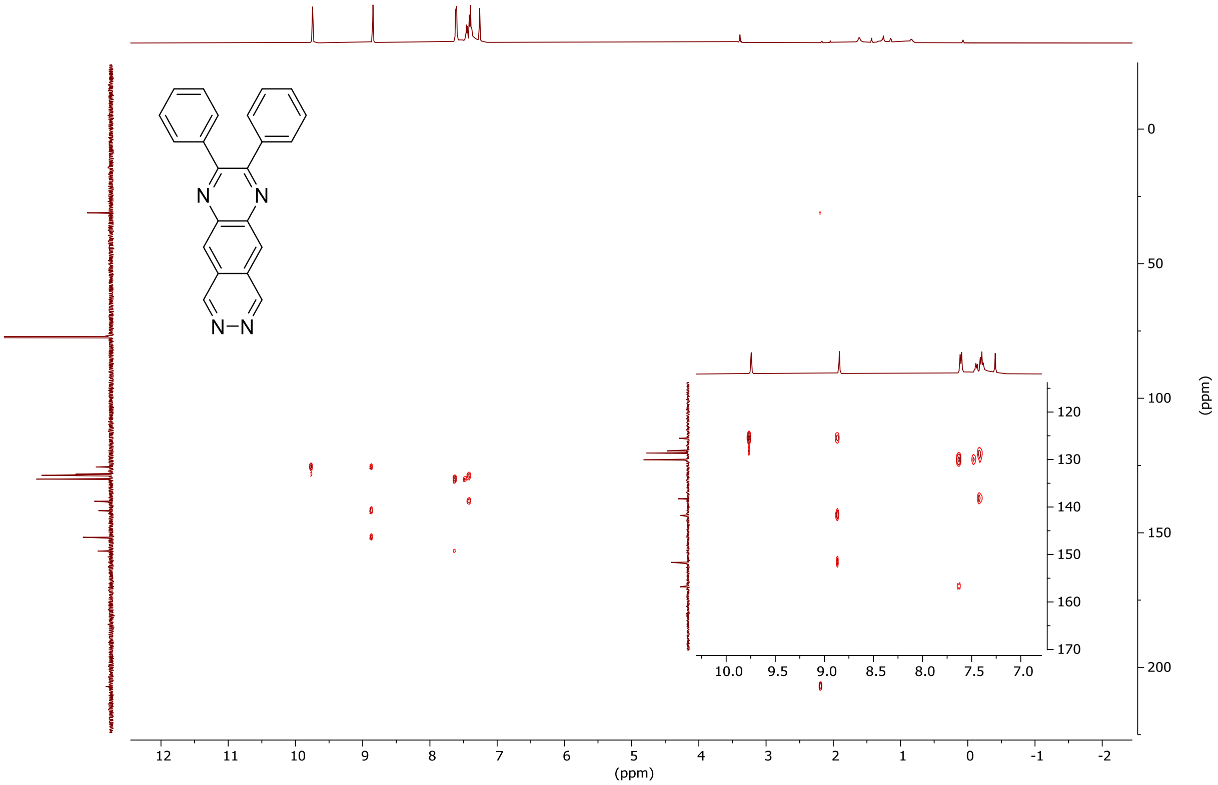

Fig. S312 HMBC (501 MHz, 126 MHz, chloroform-*d*, 298 K) spectrum of compound **5h**

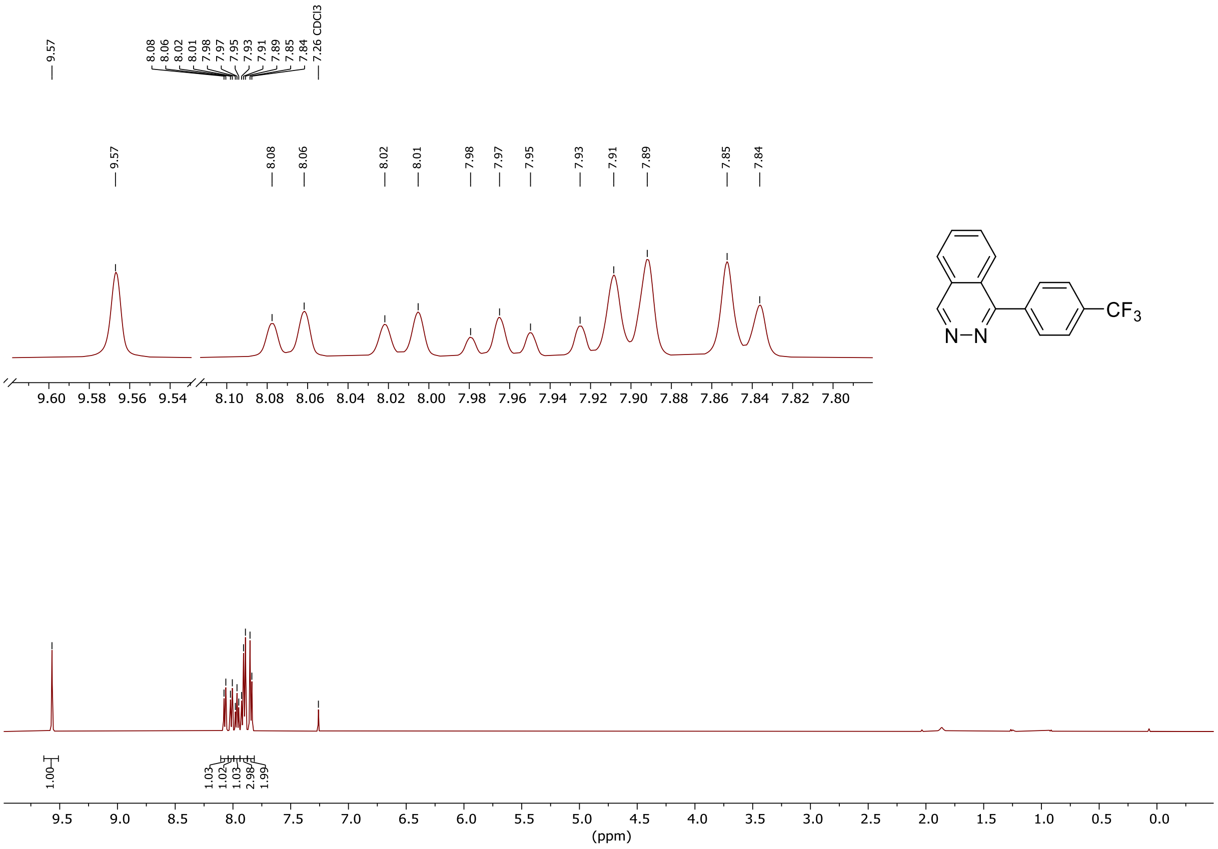

Fig. S313 ^{1}H NMR (501 MHz, chloroform-*d*, 298 K) spectrum of compound **5i**

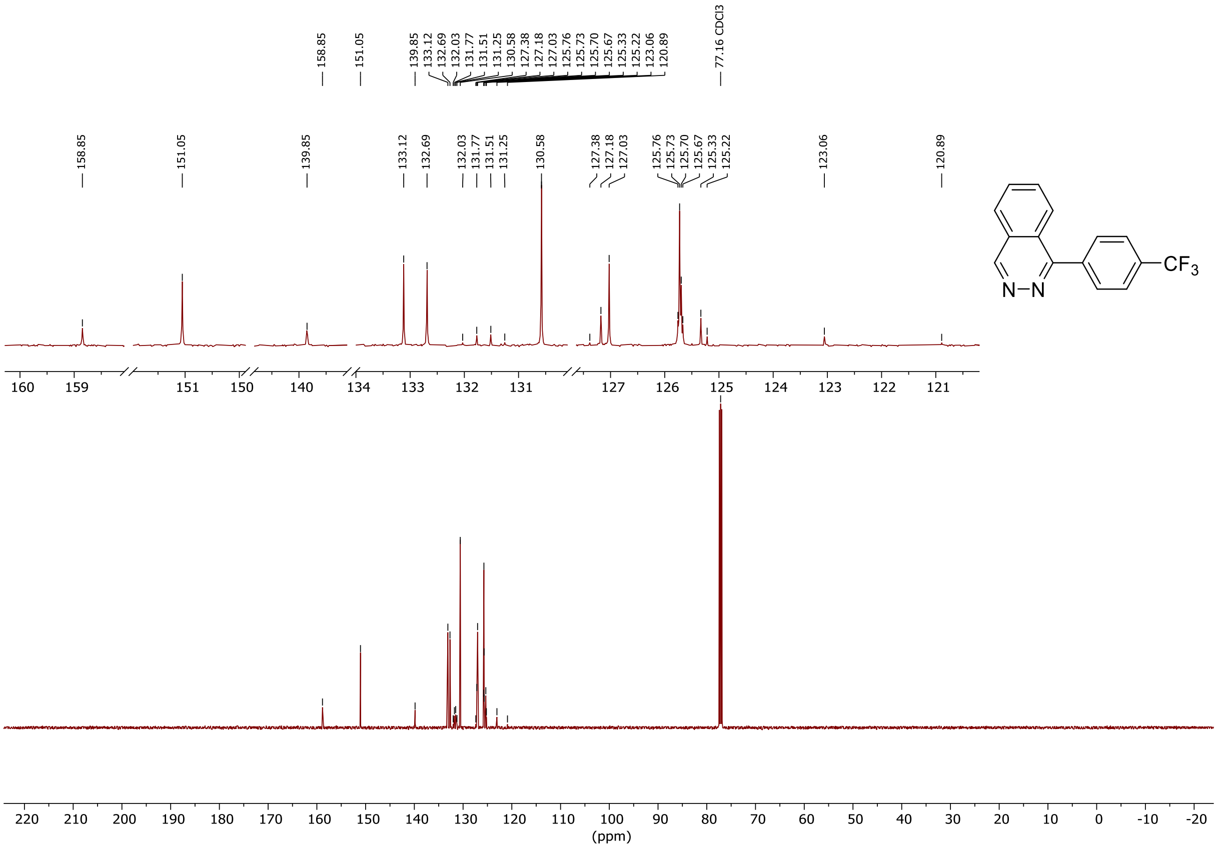

Fig. S314 ^{13}C{^{1}H} NMR (126 MHz, chloroform-*d*, 298 K) spectrum of compound **5i**

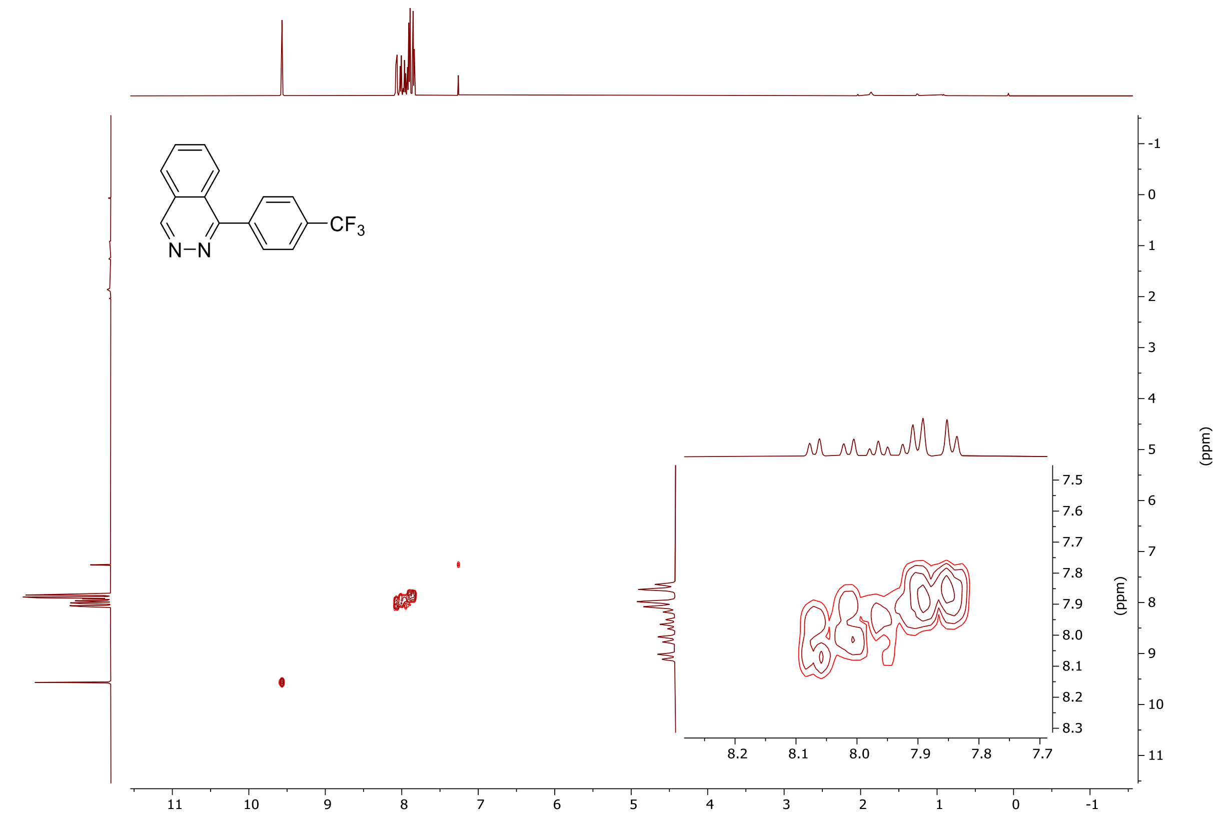

Fig. S315 COSY (501 MHz, 501 MHz, chloroform-*d*, 298 K) spectrum of compound **5i**

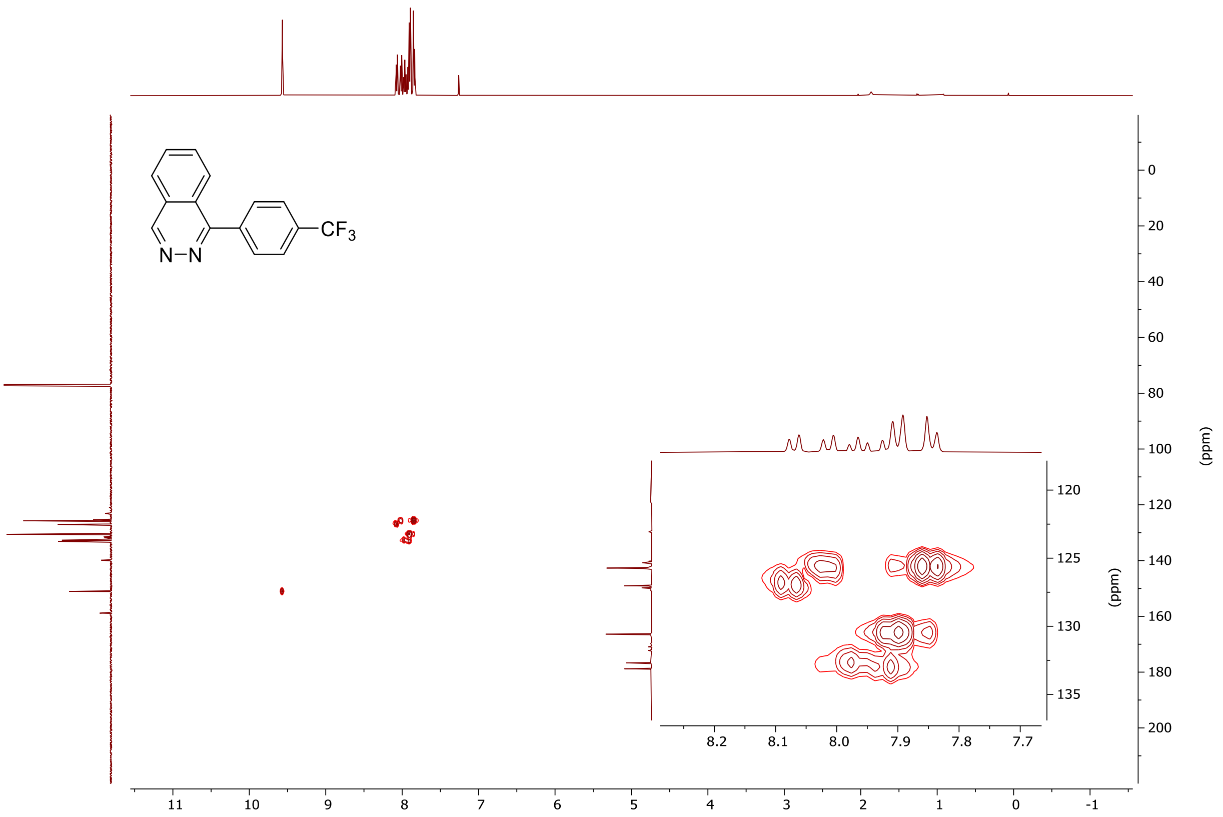

Fig. S316 HSQC (501 MHz, 126 MHz, chloroform-*d*, 298 K) spectrum of compound **5i**

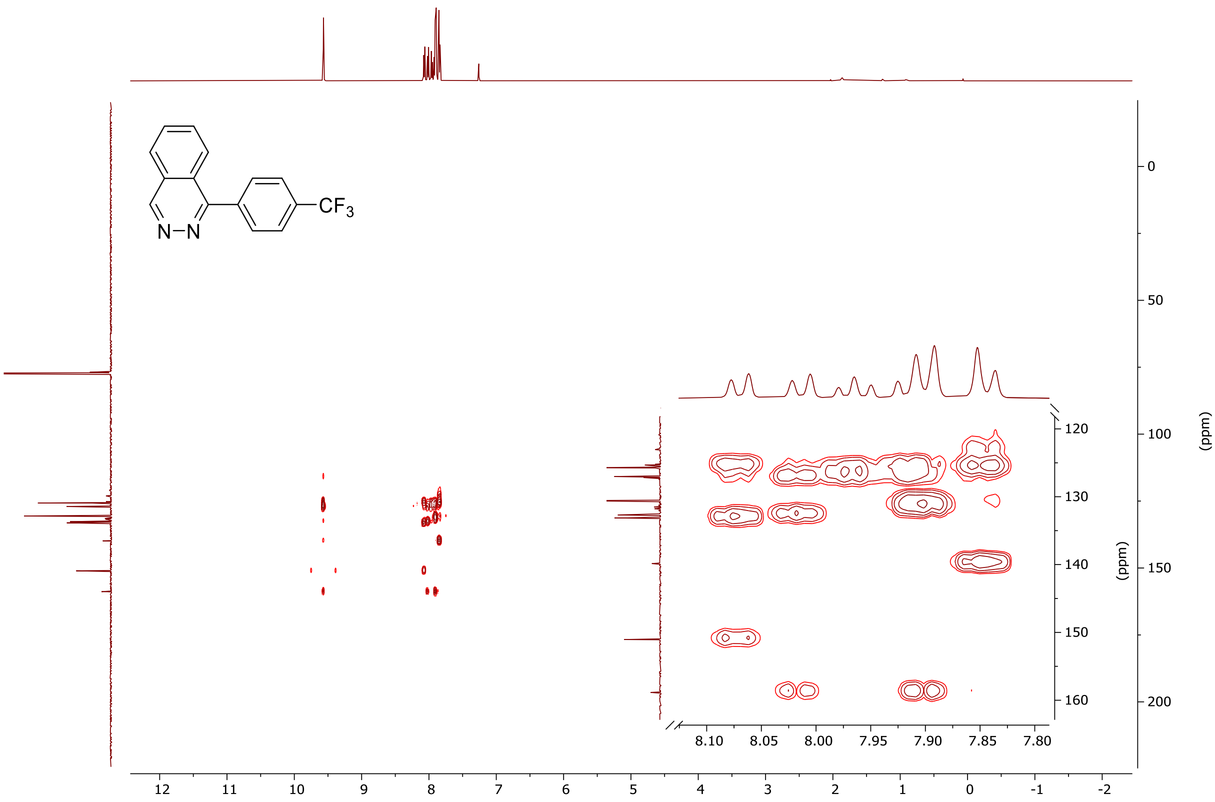

Fig. S317 HMBC (501 MHz, 126 MHz, chloroform-*d*, 298 K) spectrum of compound **5i**

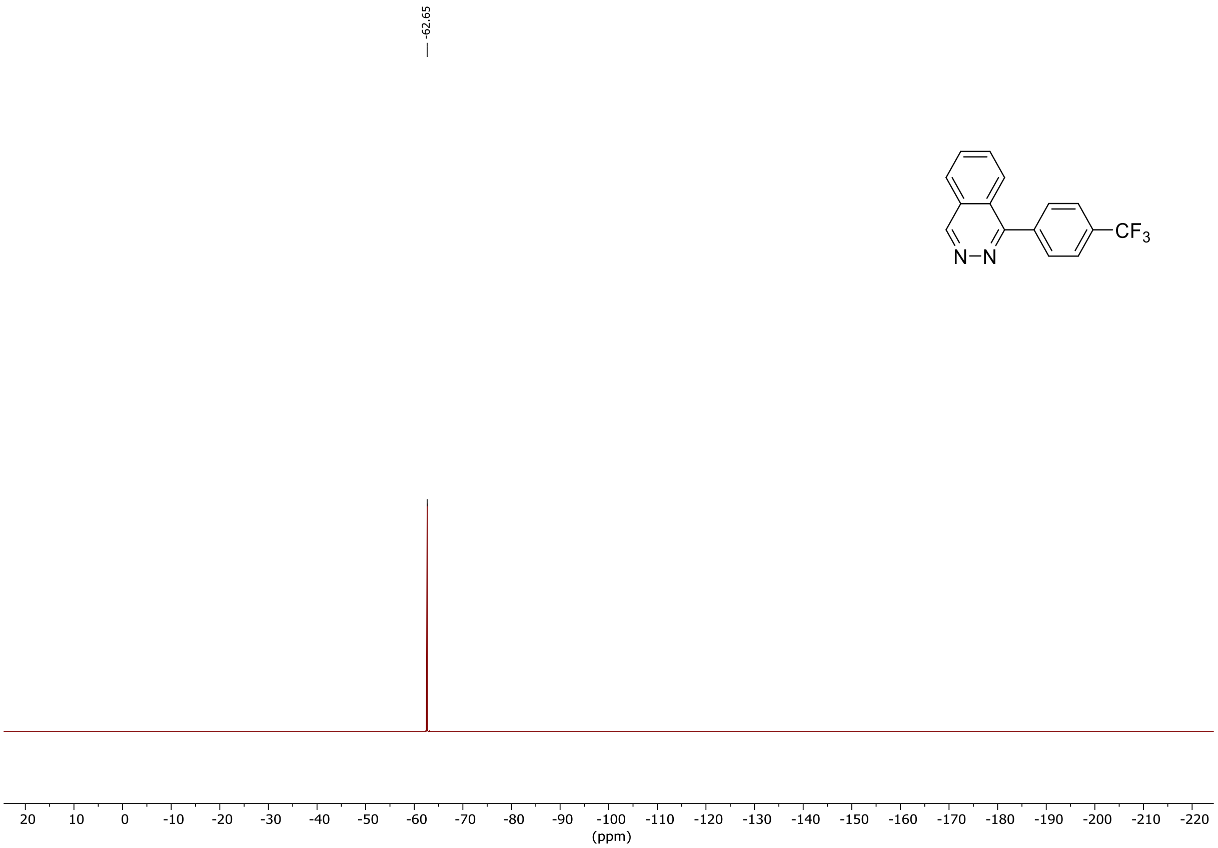

Fig. S318 ^{19}F NMR (377 MHz, chloroform-*d*, 298 K) spectrum of compound **5i**

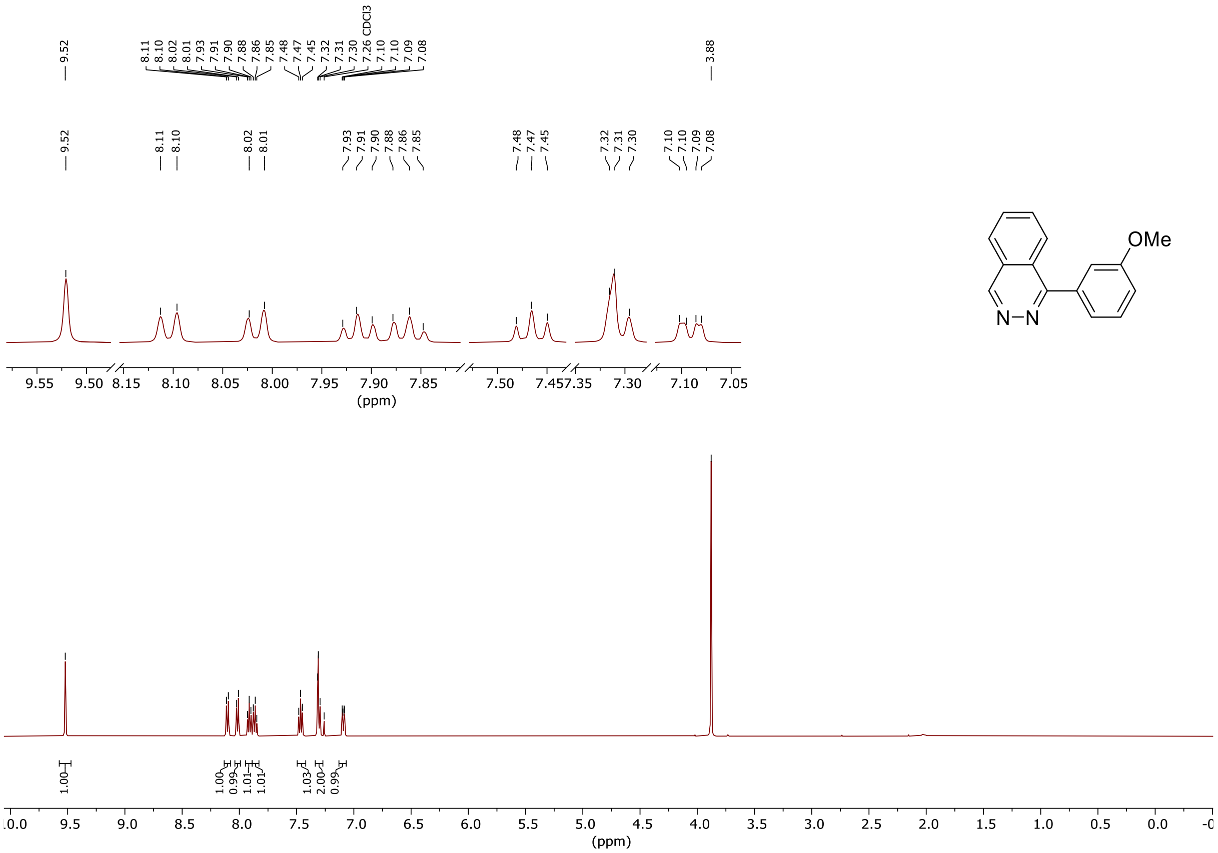

Fig. S319 ^{1}H NMR (501 MHz, chloroform-*d*, 298 K) spectrum of compound **5j**

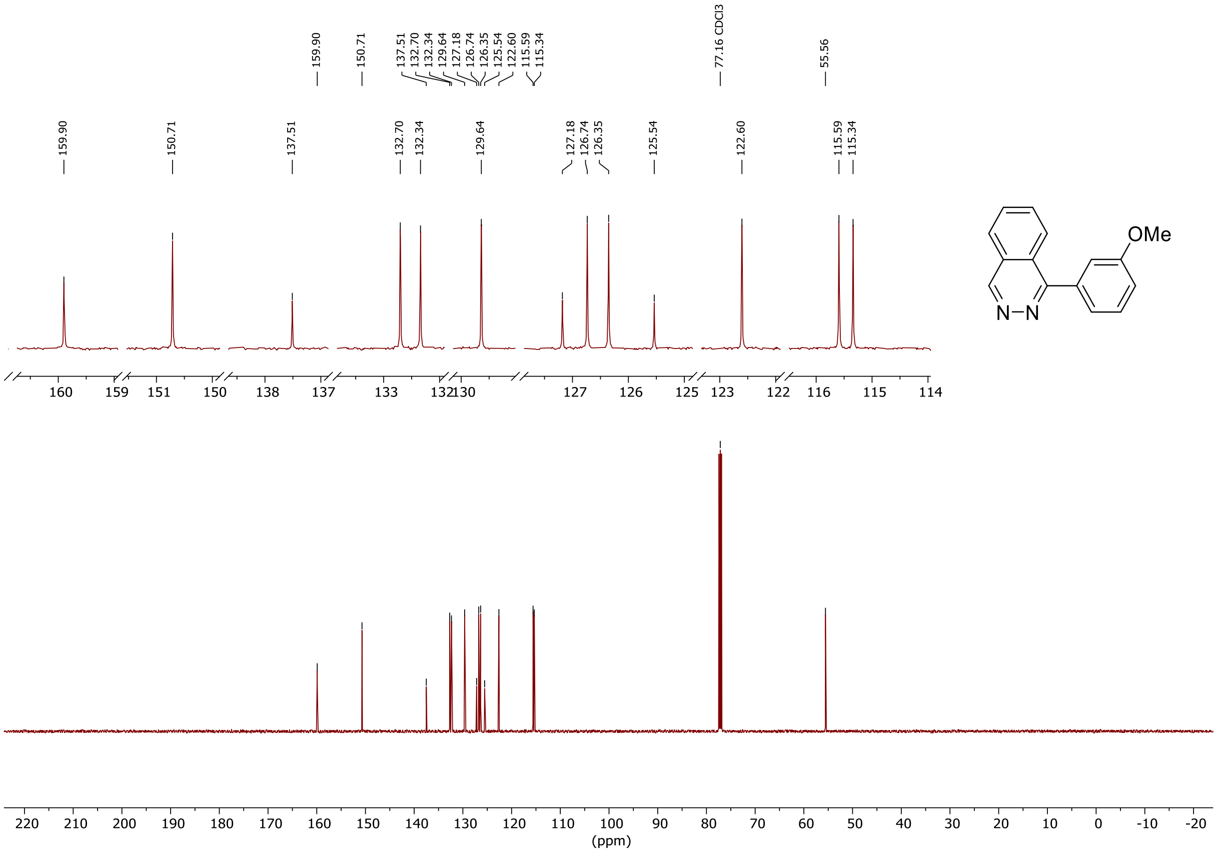

Fig. S320 ^{13}C{^{1}H} NMR (126 MHz, chloroform-*d*, 298 K) spectrum of compound **5j**

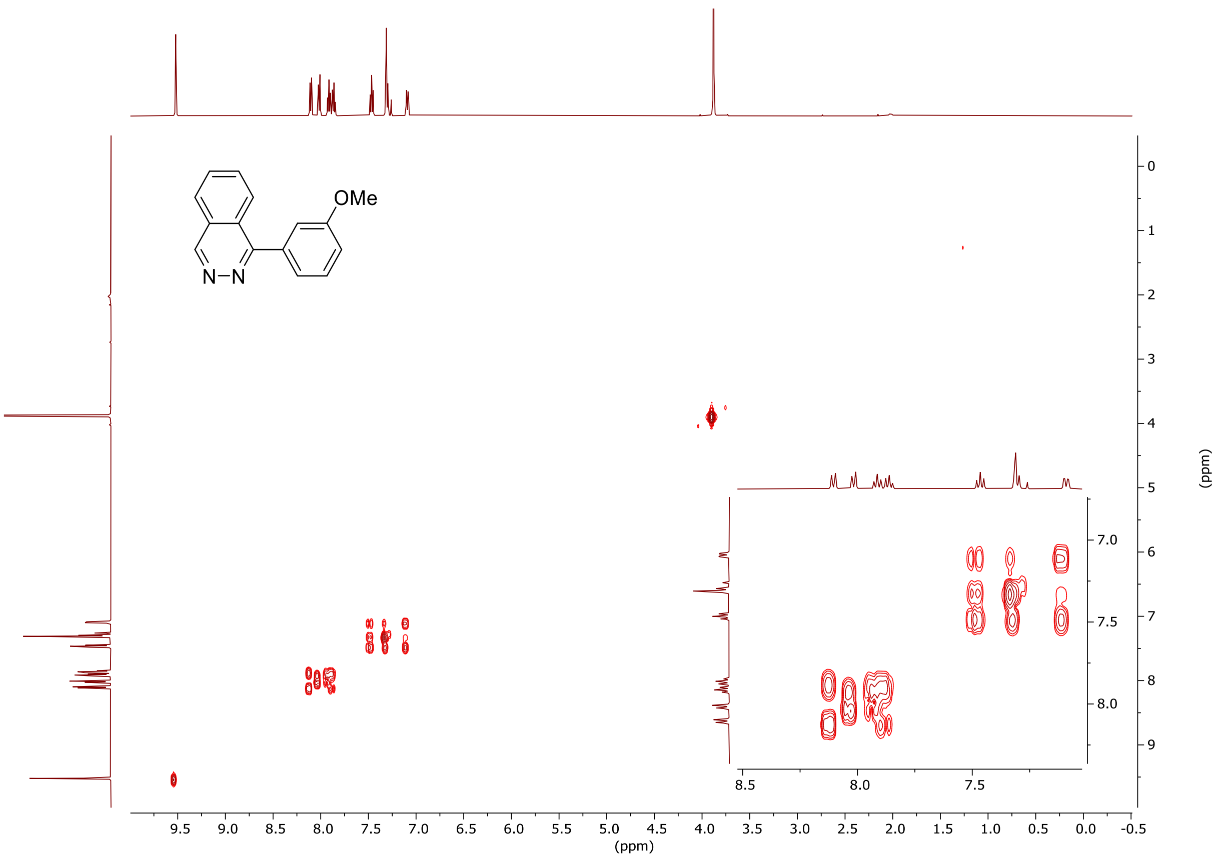

Fig. S321 COSY (501 MHz, 501 MHz, chloroform-*d*, 298 K) spectrum of compound **5j**

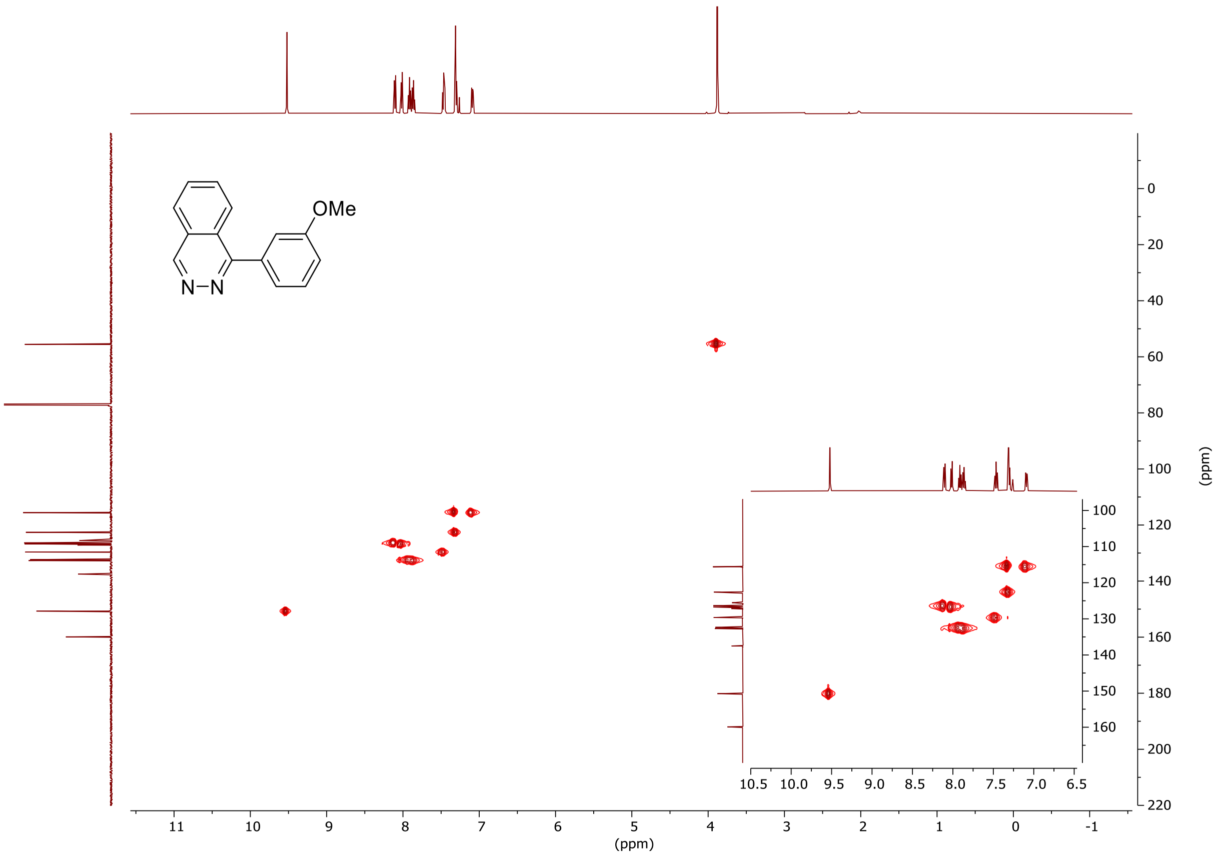

Fig. S322 HSQC (501 MHz, 126 MHz, chloroform-*d*, 298 K) spectrum of compound **5j**

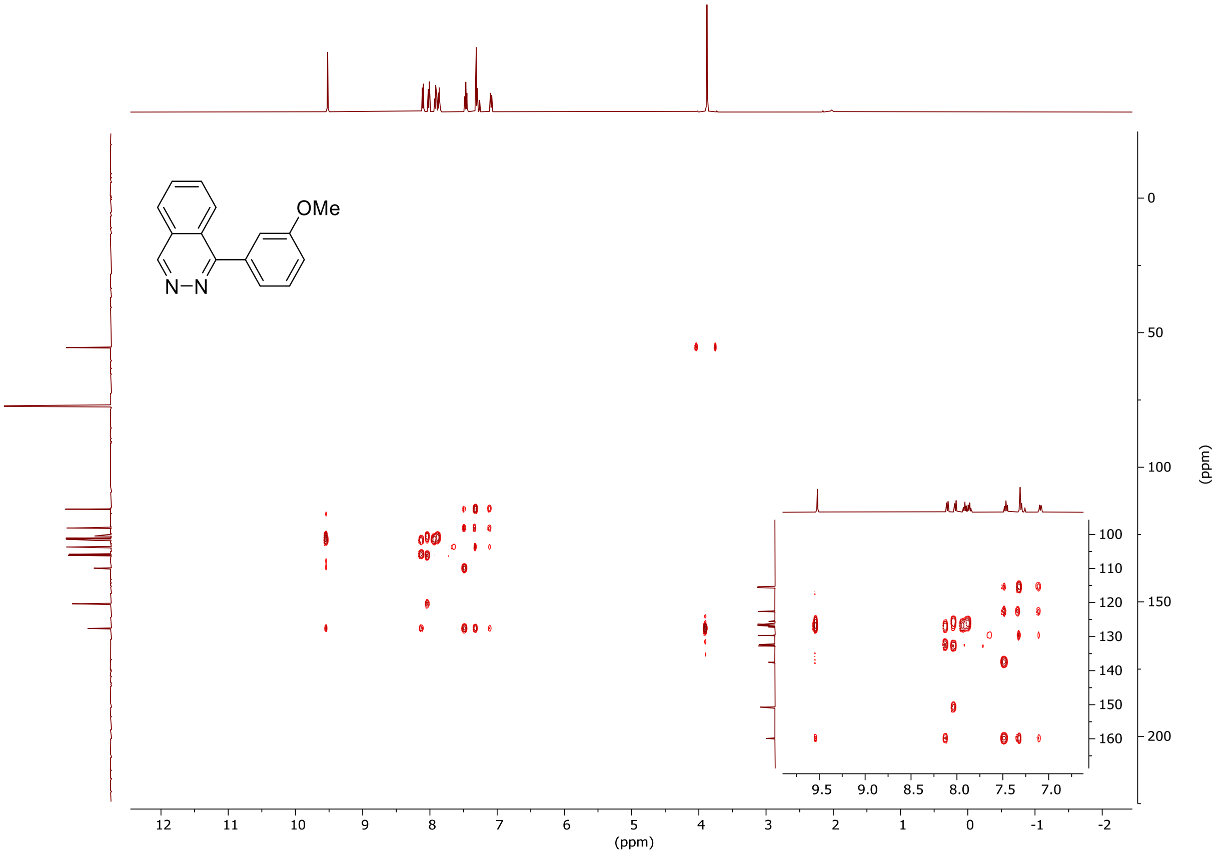

Fig. S323 HMBC (501 MHz, 126 MHz, chloroform-*d*, 298 K) spectrum of compound **5j**

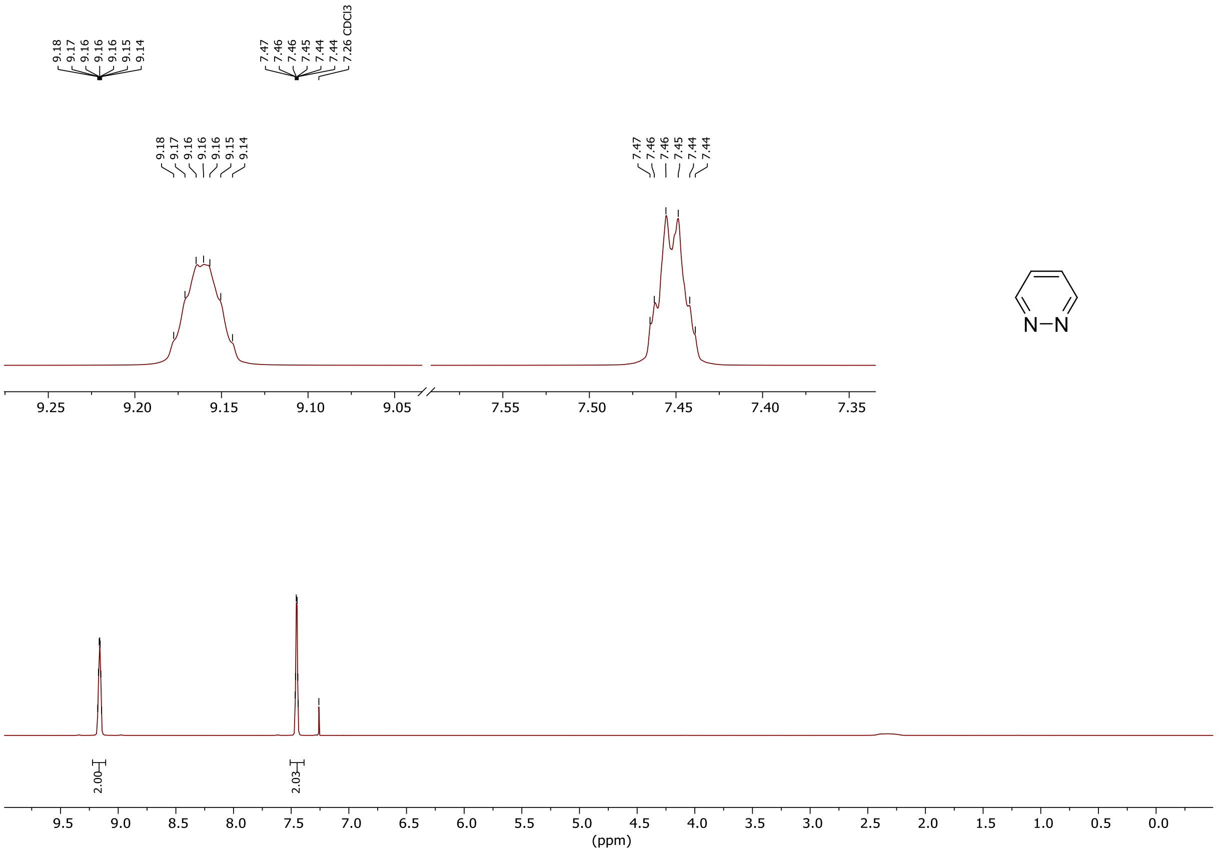

Fig. S324 ^{1}H NMR (501 MHz, chloroform-*d*, 298 K) spectrum of compound **5k**

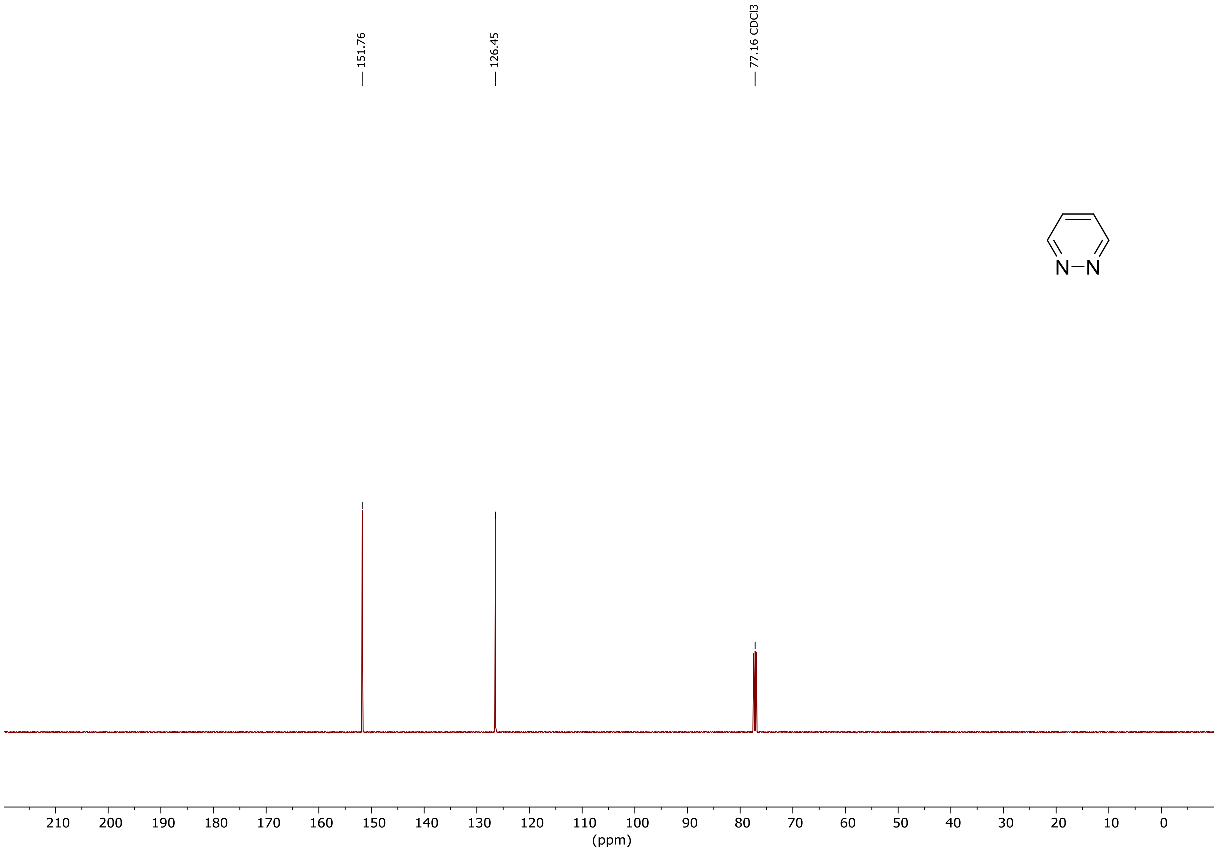

Fig. S325 $^{13}C\{^{1}H\}$ NMR (126 MHz, chloroform-*d*, 298 K) spectrum of compound **5k**

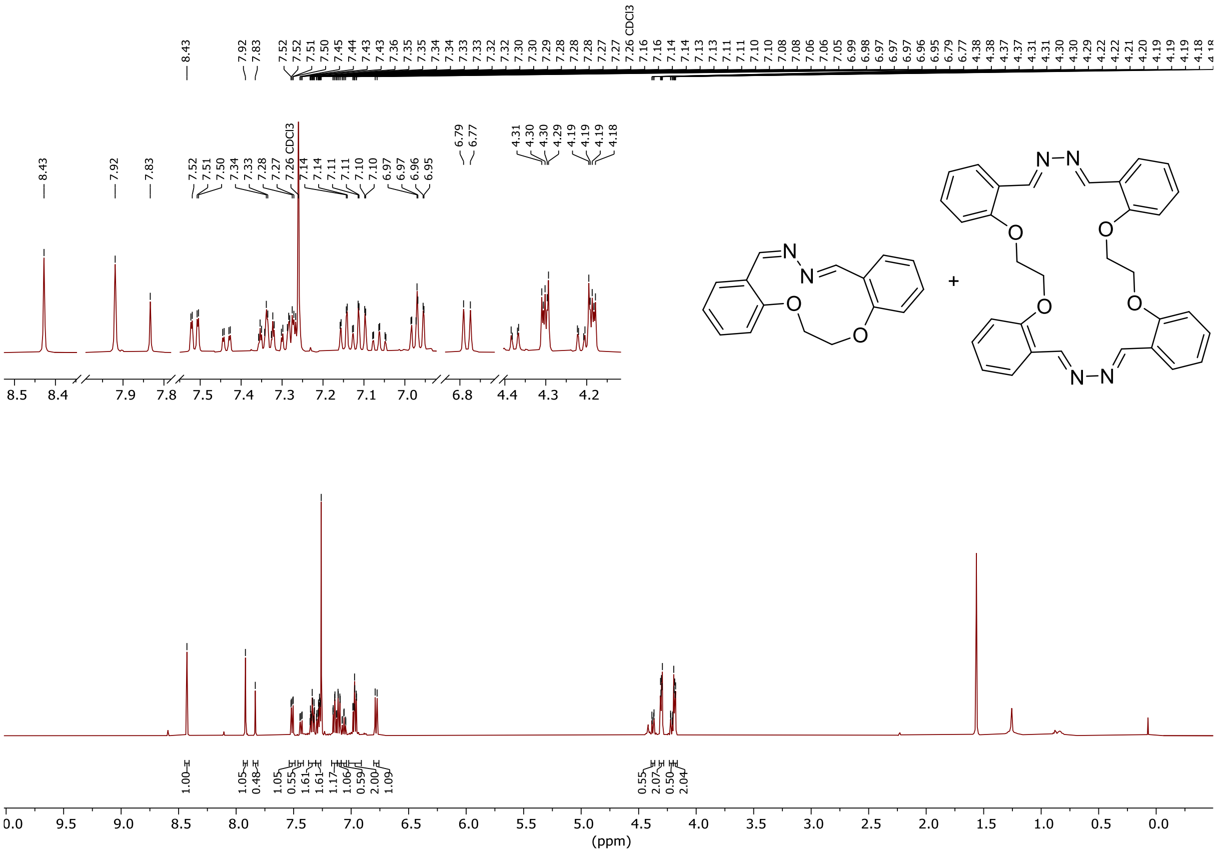

Fig. S326 ^{1}H NMR (501 MHz, chloroform-*d*, 298 K) spectrum of compound **5l** + **5l'** (trace)

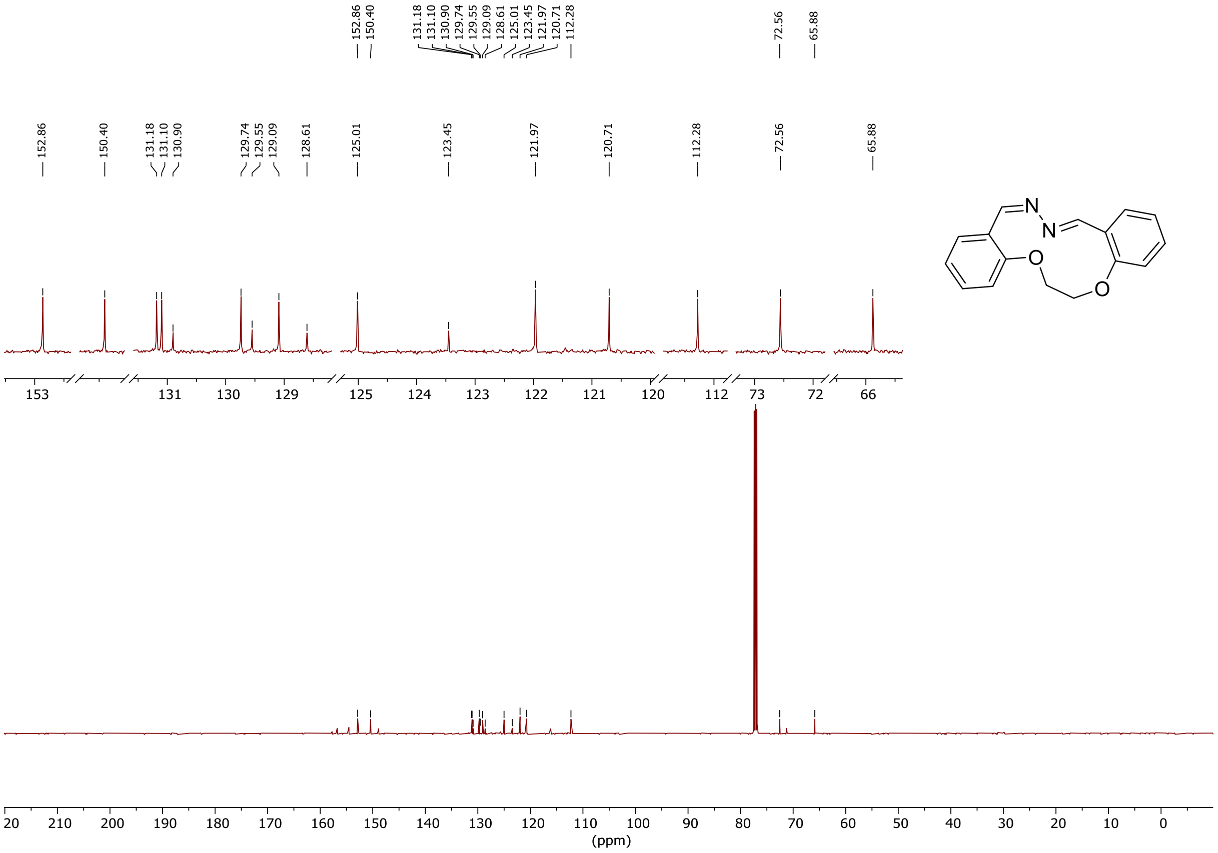

Fig. S327 ^{13}C{^{1}H} NMR (126 MHz, chloroform-*d*, 298 K) spectrum of compound **5l** + **5l'** (trace)

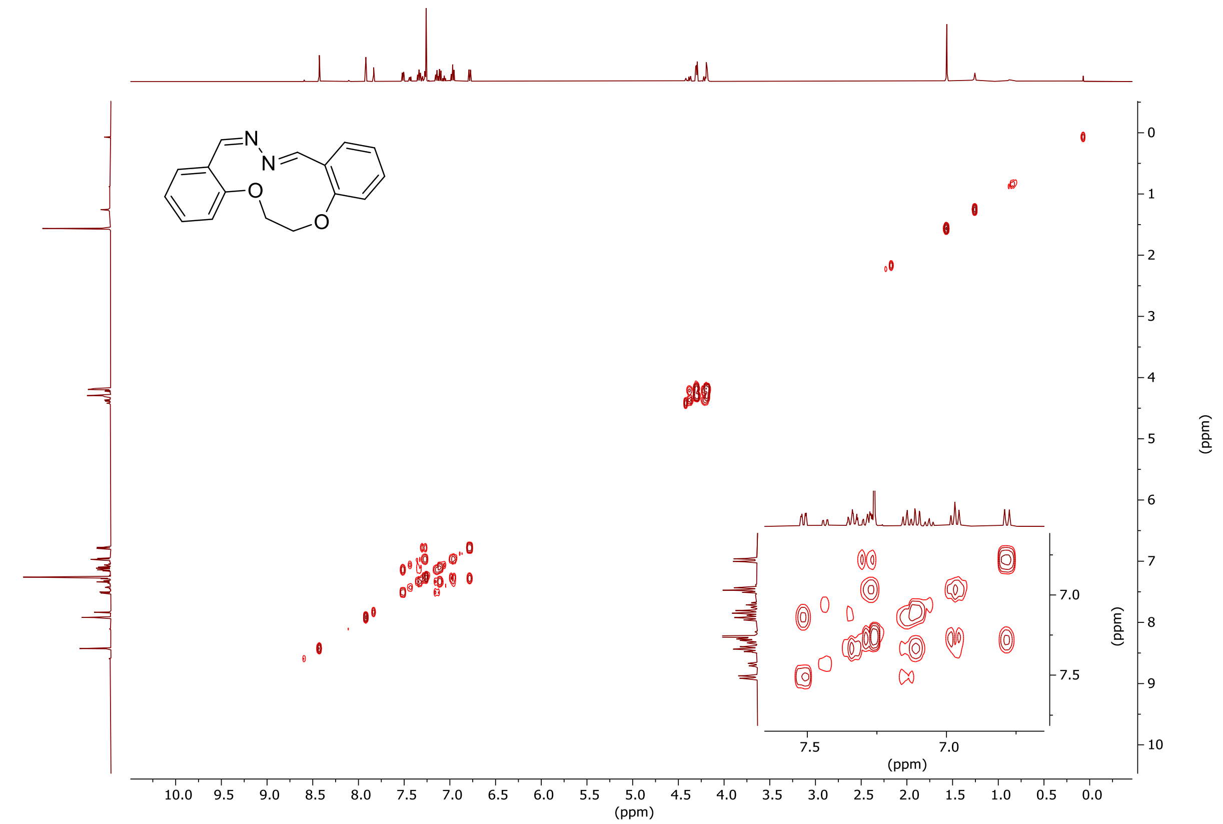

Fig. S328 COSY (501 MHz, 501 MHz, chloroform-*d*, 298 K) spectrum of compound **5l** + **5l'** (trace)

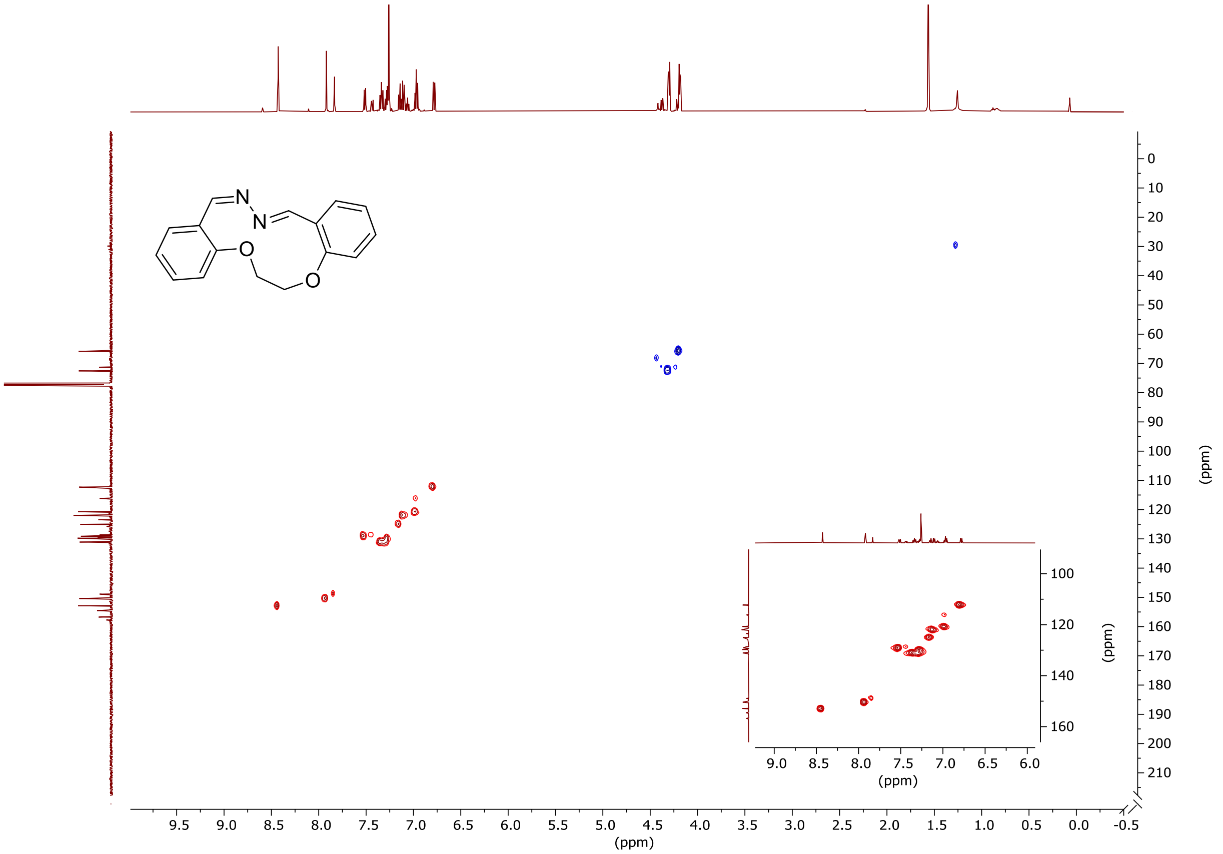

Fig. S329 HSQC (501 MHz, 126 MHz, chloroform-*d*, 298 K) spectrum of compound **5l**

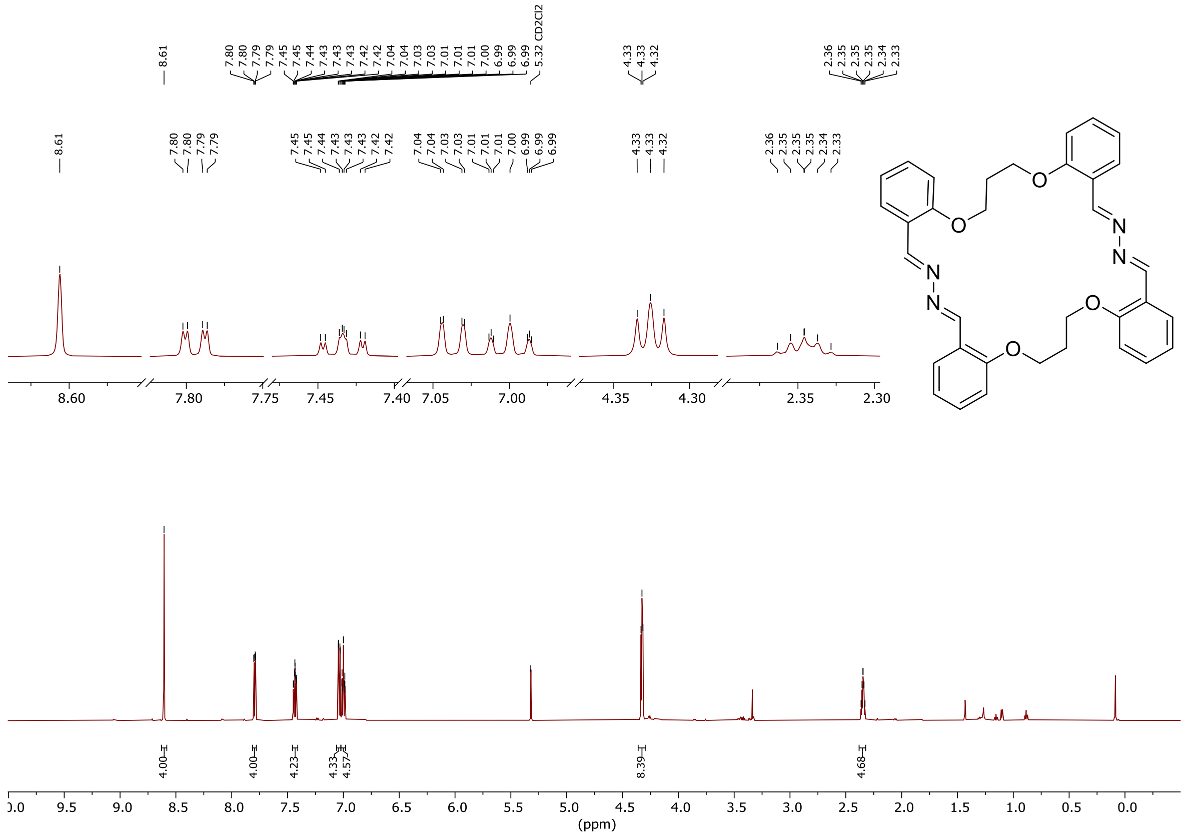

Fig. S330 ^{1}H NMR (600 MHz, methylene chloride-d_2, 298 K) spectrum of compound **5m** + **5m’**

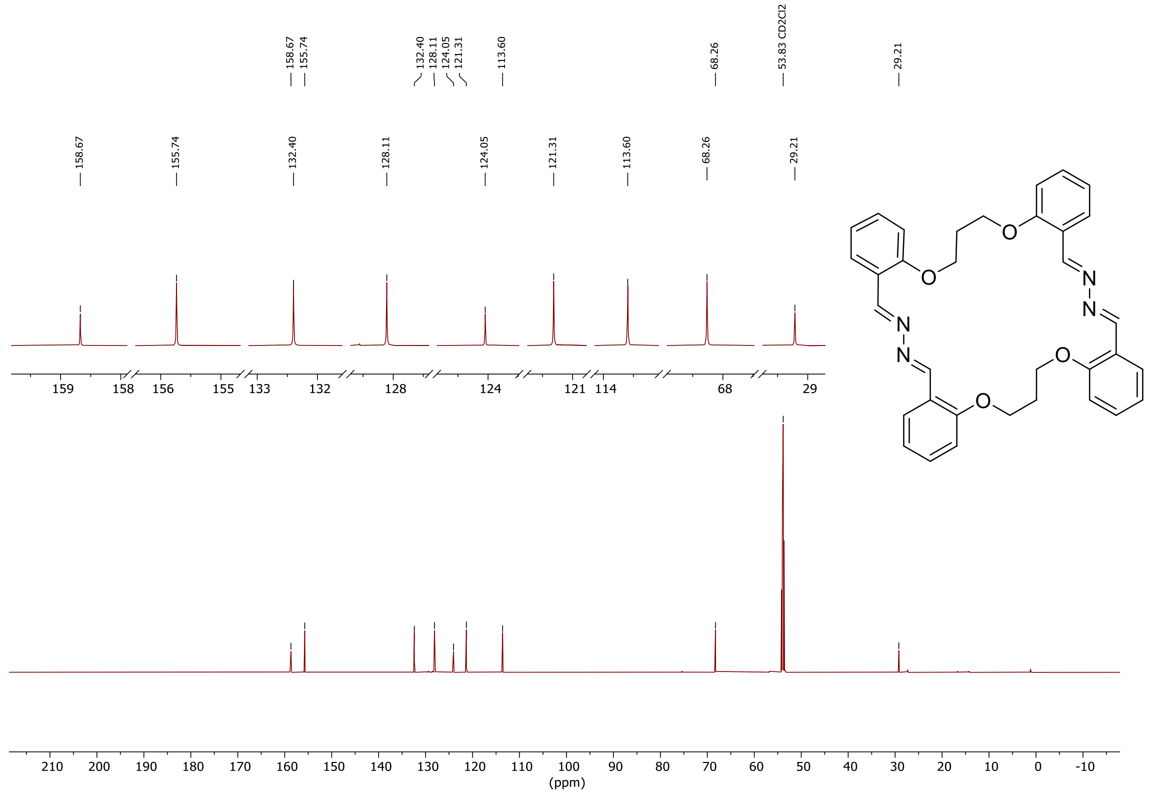

Fig. S331 ^{13}C{^{1}H} NMR (151 MHz, methylene chloride-d_2, 298 K) spectrum of compound **5m’**

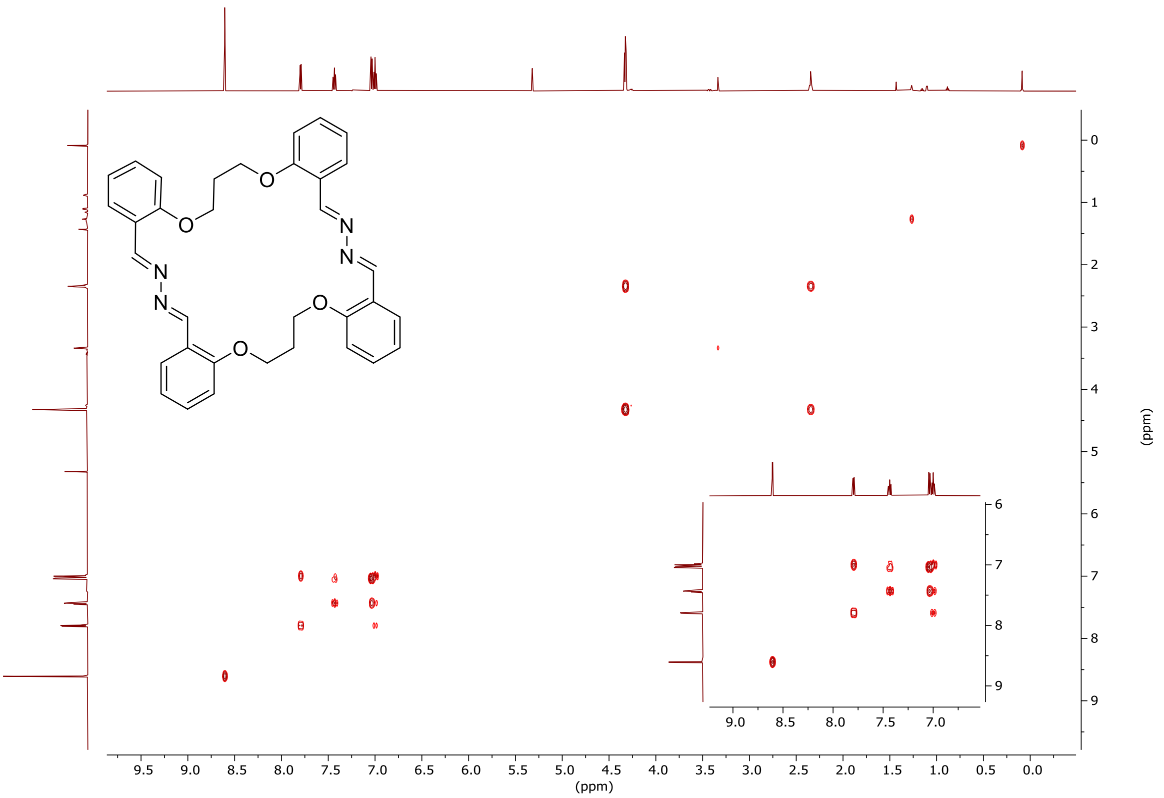

Fig. S332 COSY (600 MHz, 600 MHz, methylene chloride-d_2, 298 K) spectrum of compound **5m'**

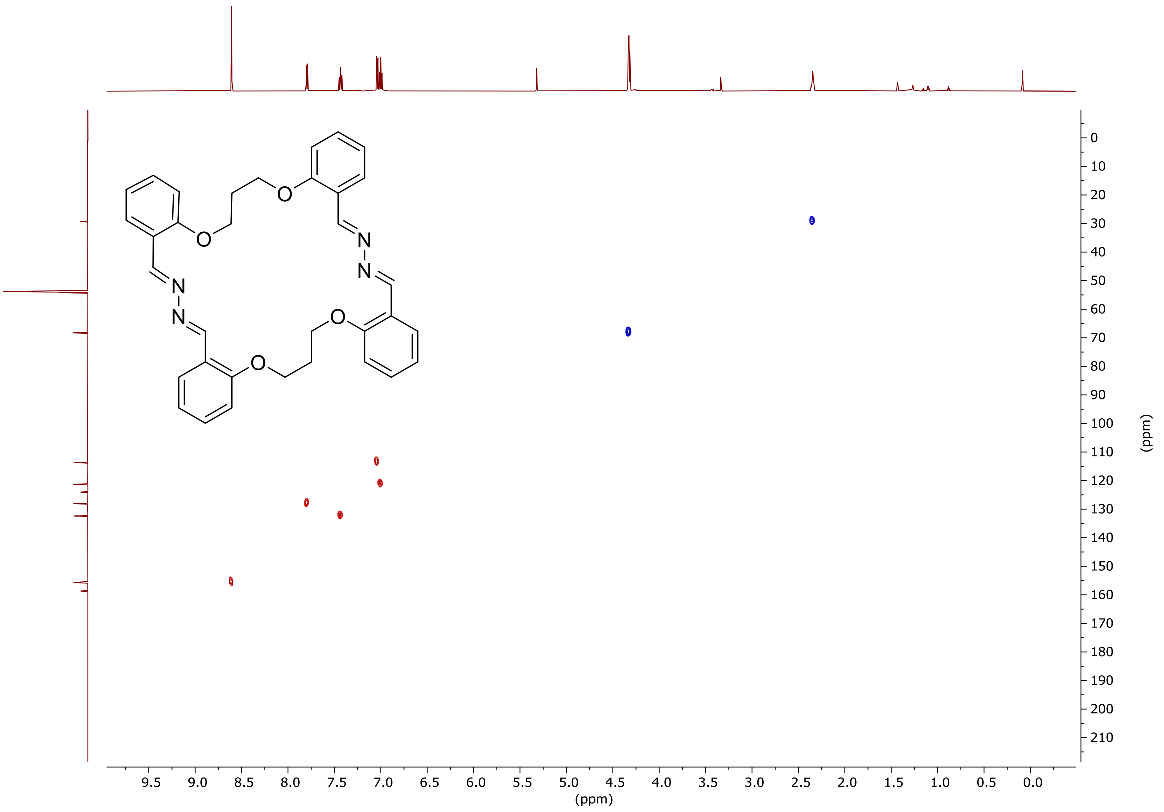

Fig. S333 HSQC (600 MHz, 151 MHz, methylene chloride-d_2, 298 K) spectrum of compound **5m'**

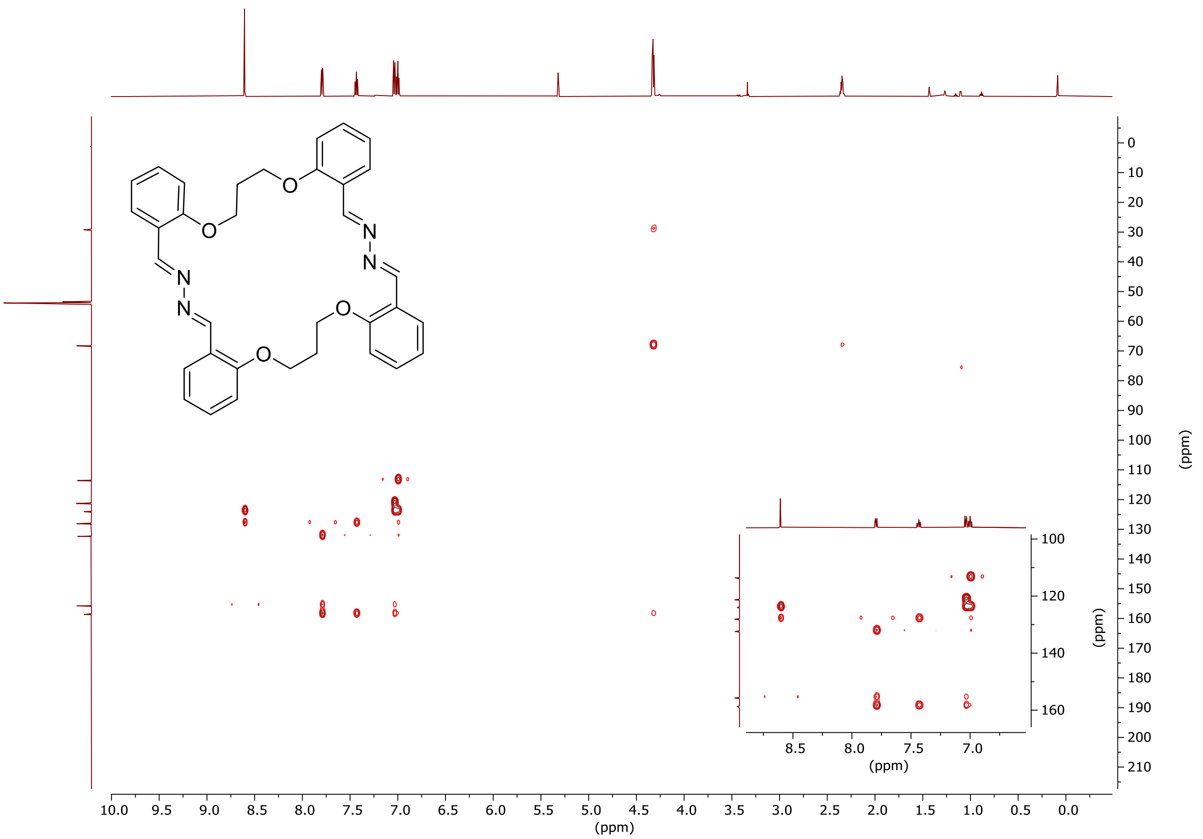

Fig. S334 HMBC of (600 MHz, 151 MHz, methylene chloride-d_2, 298 K) spectrum of compound **5m'**

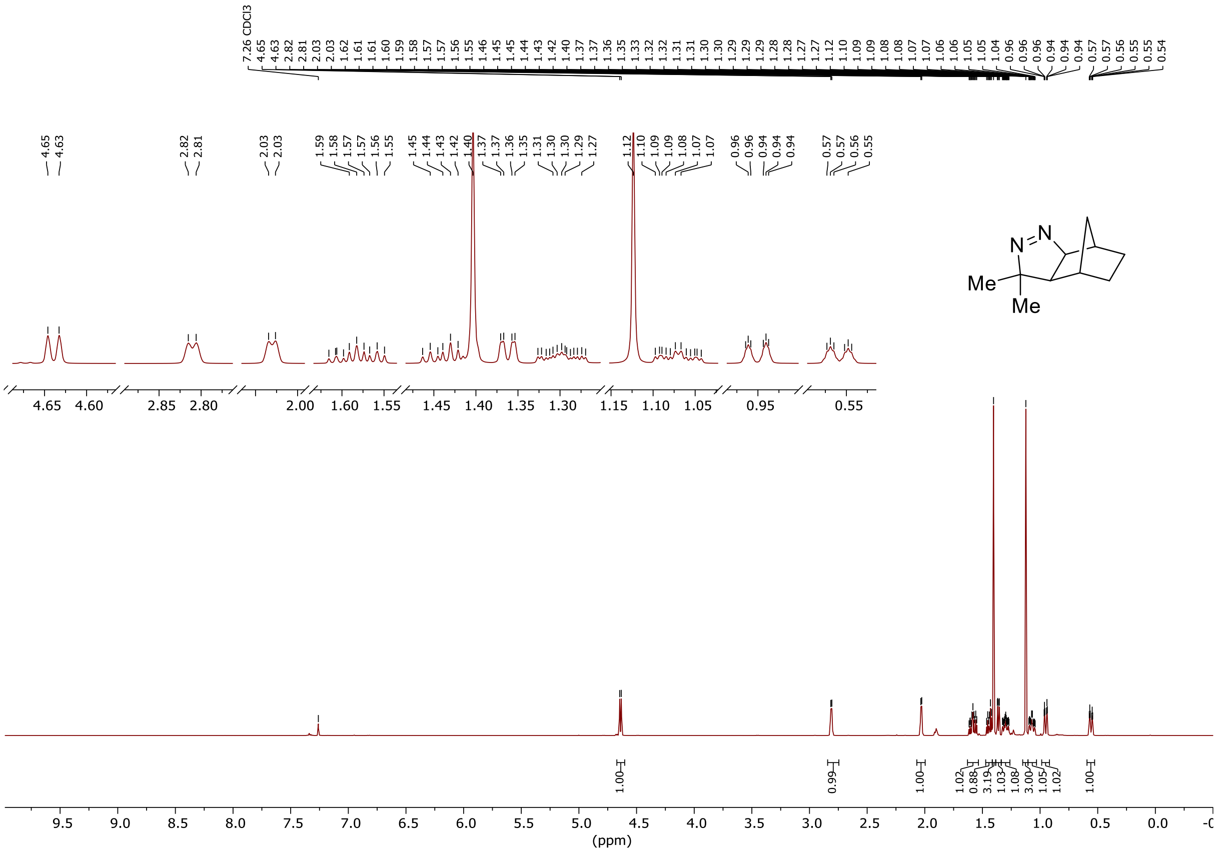

Fig. S335 ^{1}H NMR (501 MHz, chloroform-*d*, 298 K) spectrum of compound **8**

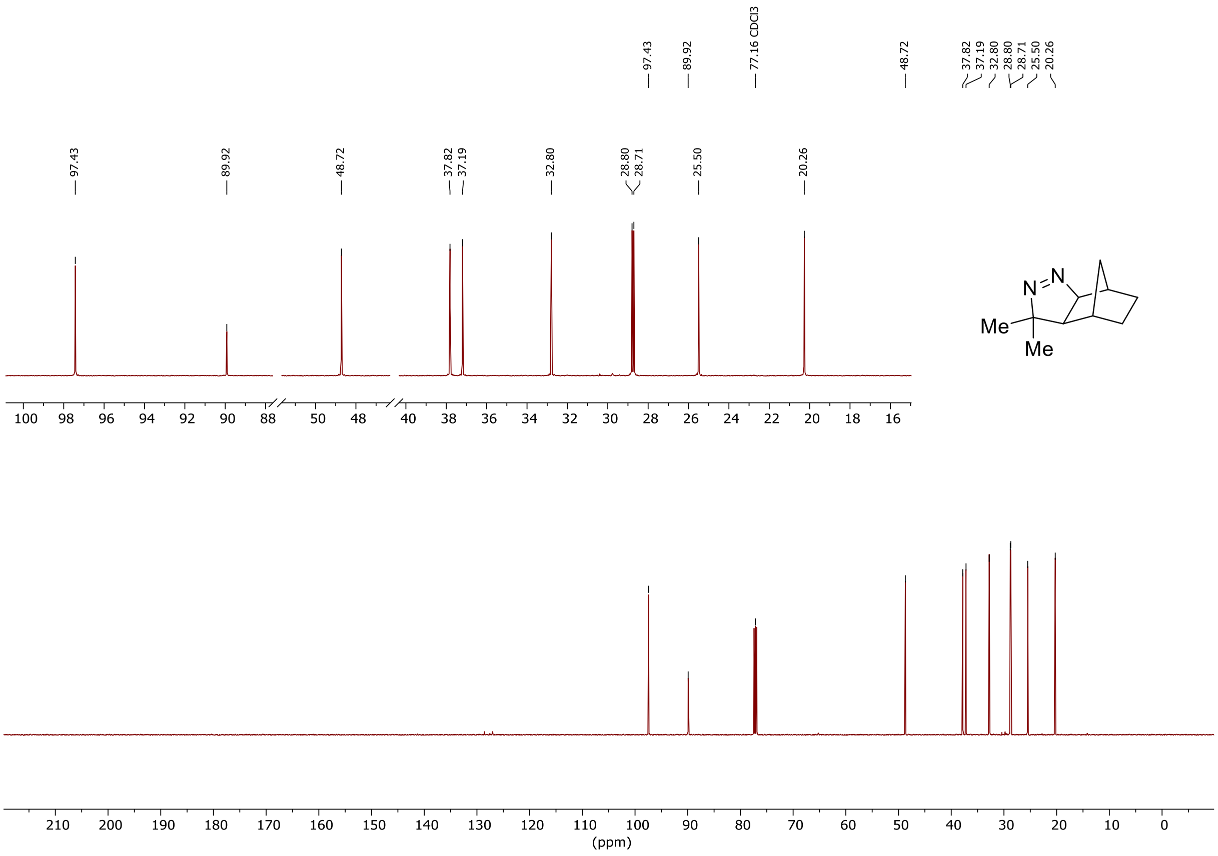

Fig. S336 ^{13}C{^{1}H} NMR (126 MHz, chloroform-*d*, 298 K) spectrum of compound **8**

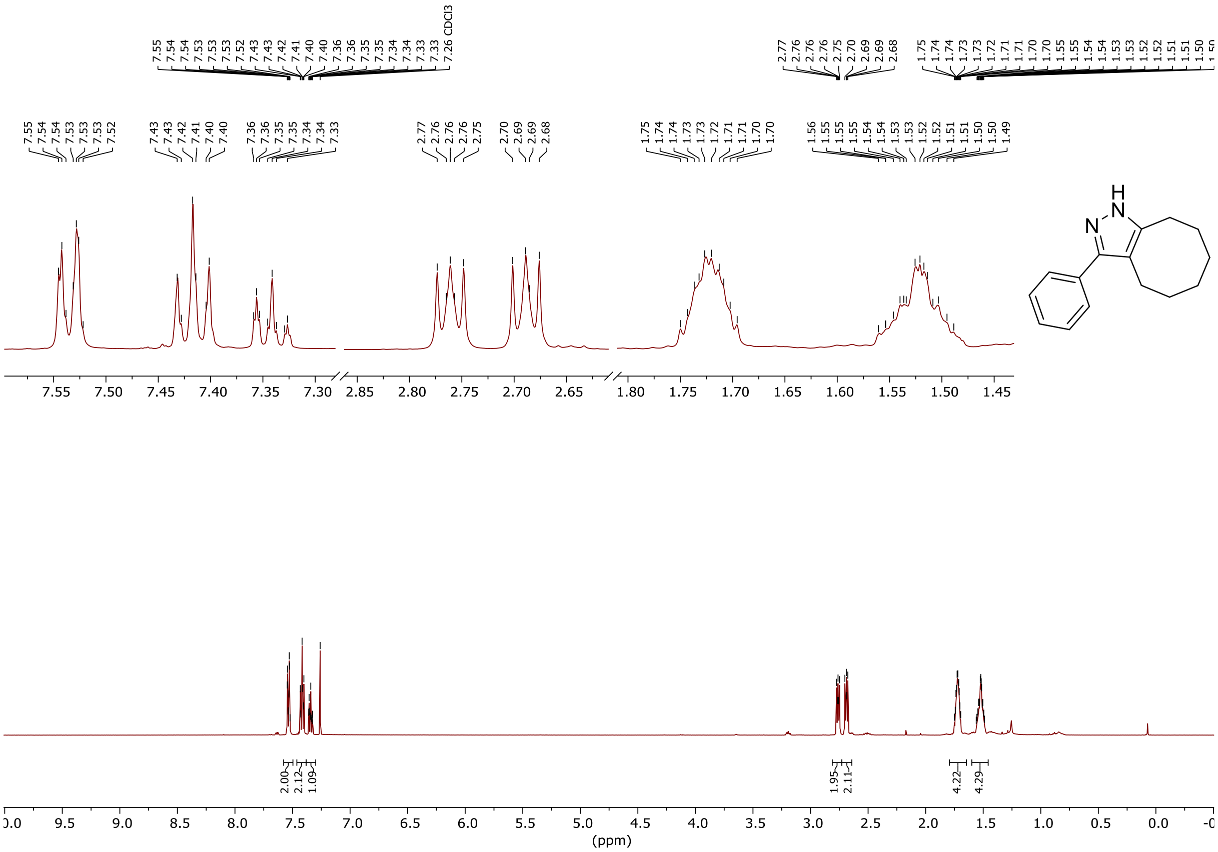

Fig. S337 ^{1}H NMR (501 MHz, chloroform-*d*, 298 K) spectrum of compound **10**

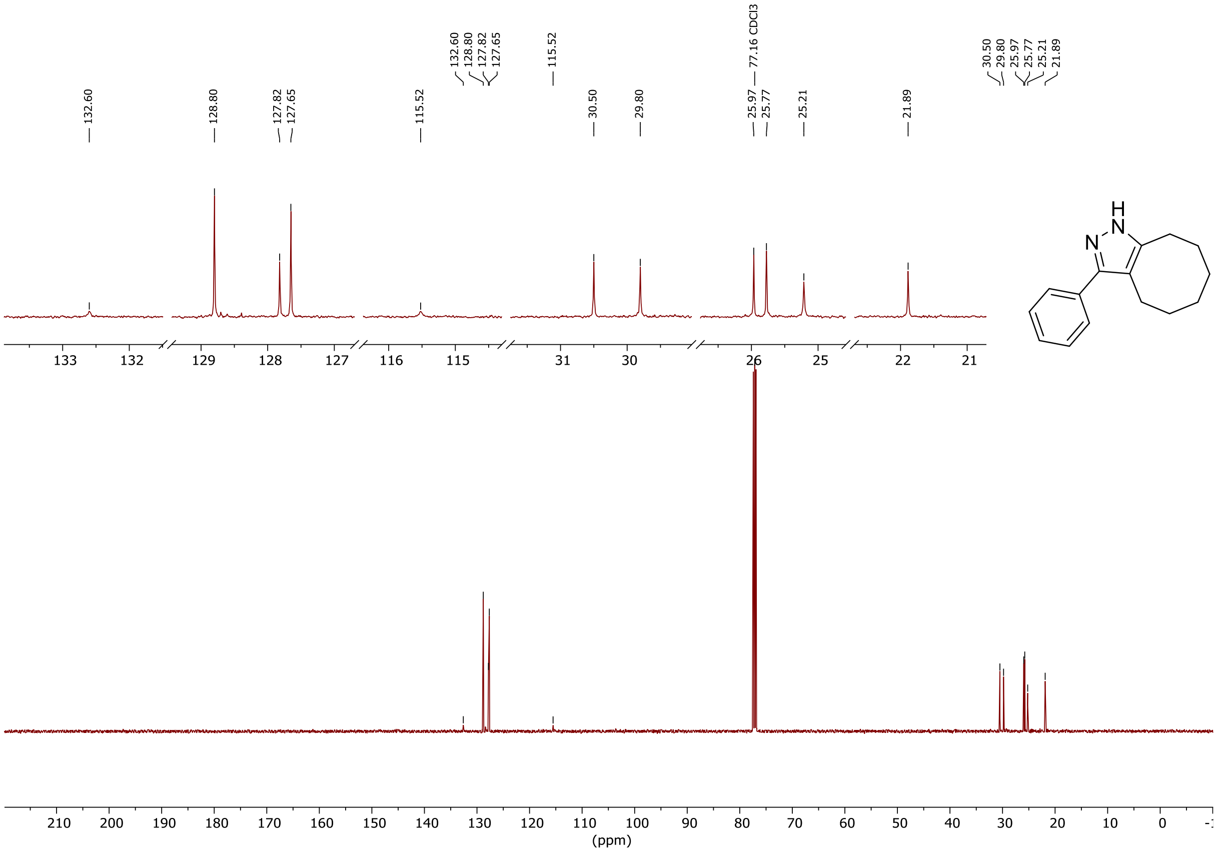

Fig. S338 $^{13}C\{^{1}H\}$ NMR (126 MHz, chloroform-*d*, 298 K) spectrum of compound **10**

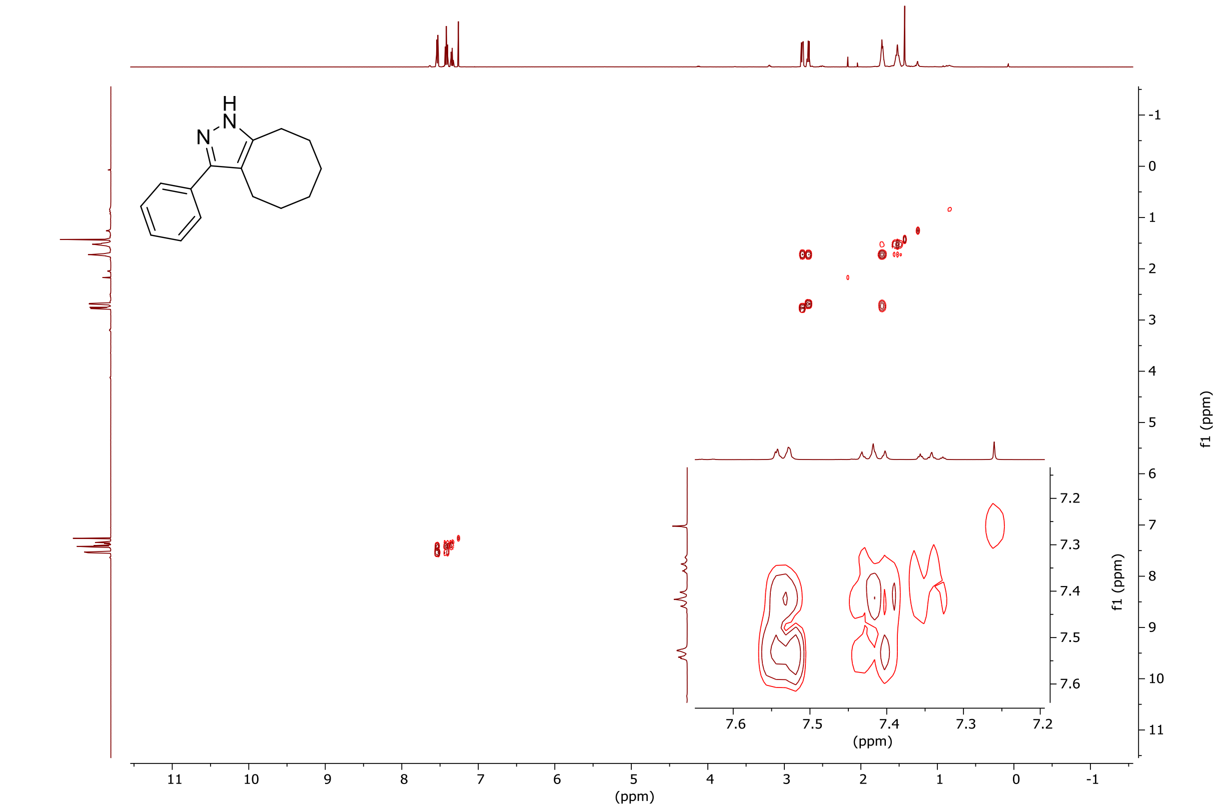

Fig. S339 COSY (501 MHz, 501 MHz, chloroform-*d*, 298 K) spectrum of compound **10**

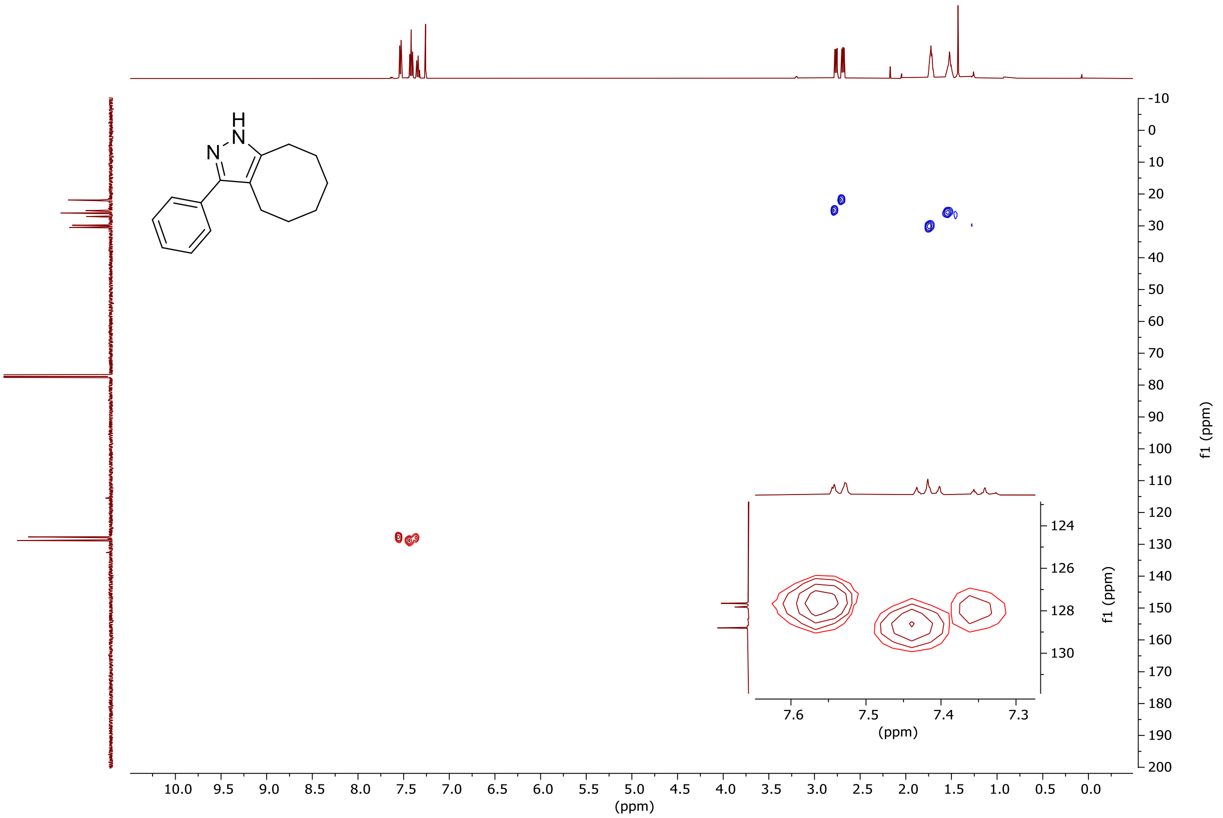

Fig. S340 HSQC (501 MHz, 126 MHz, chloroform-*d*, 298 K) spectrum of compound **10**

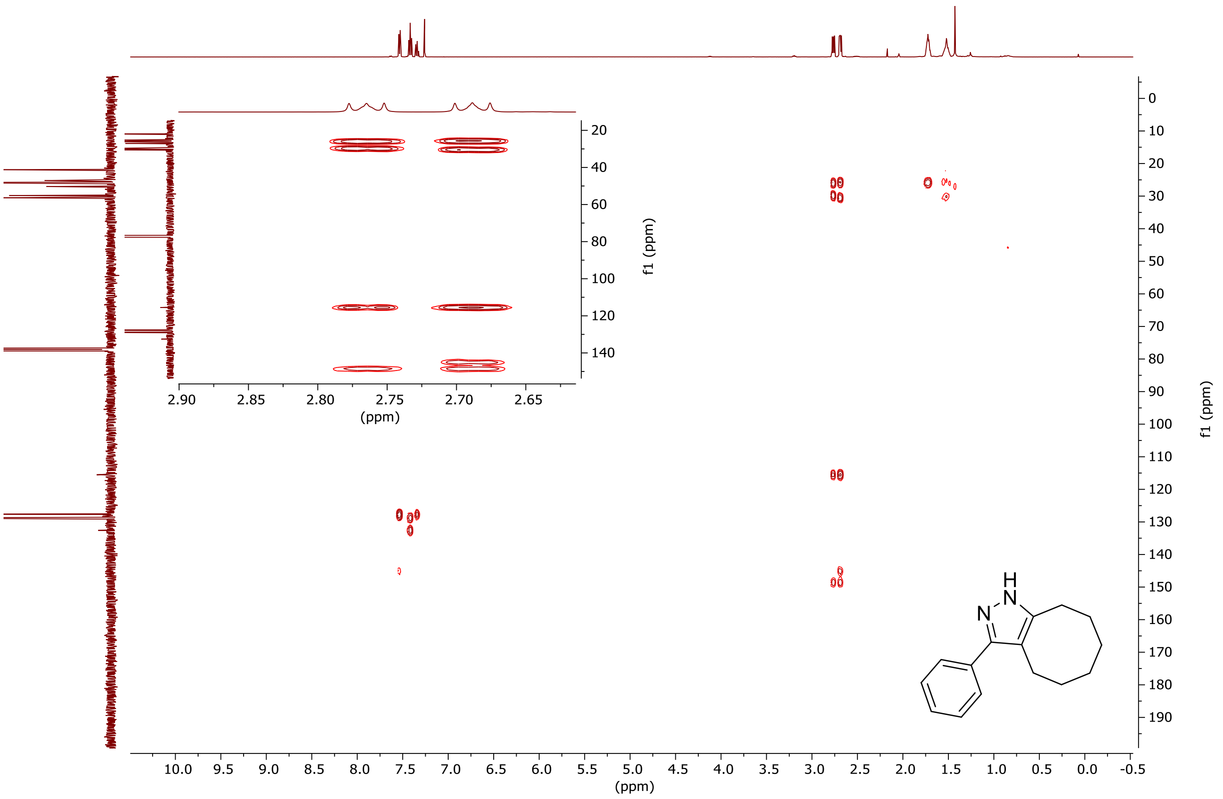

Fig. S341 HMBC (501 MHz, 126 MHz, chloroform-*d*, 298 K) spectrum of compound **10**

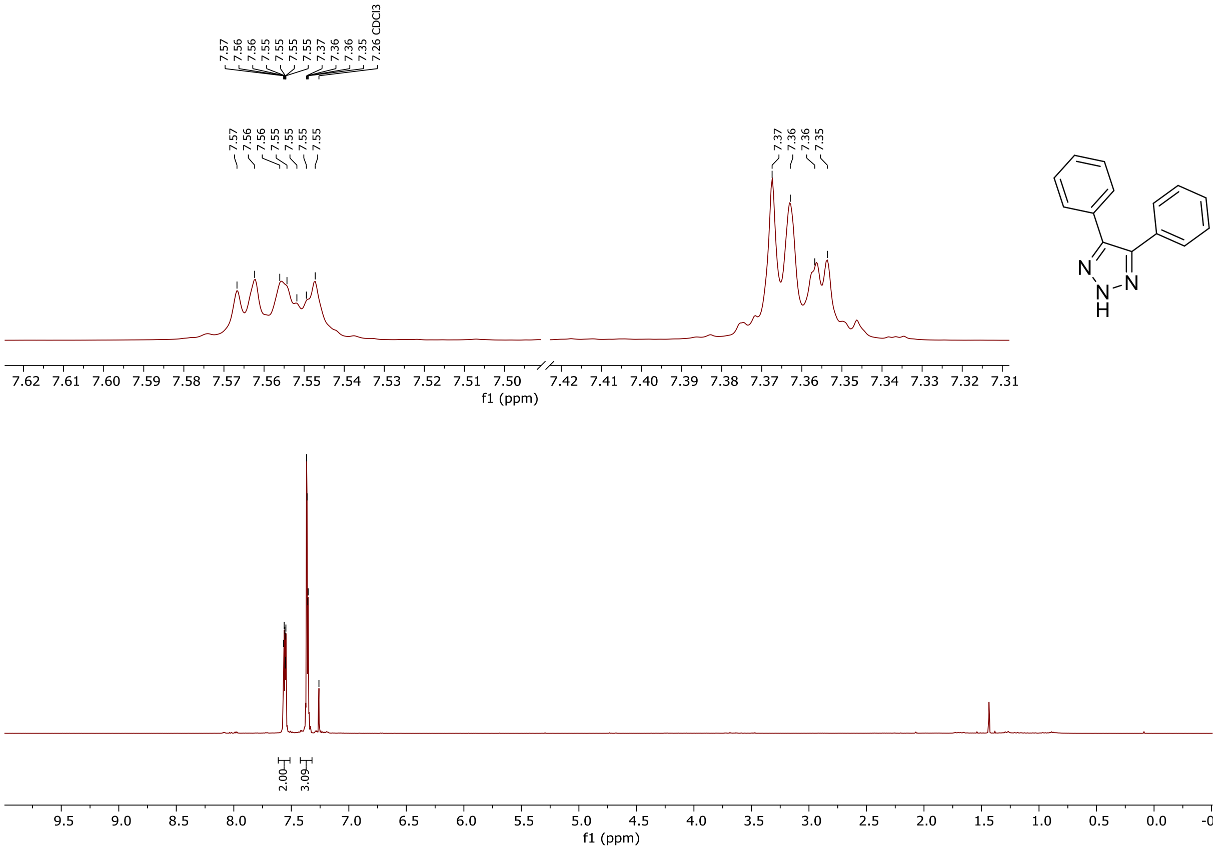

Fig. S342 ^{1}H NMR (501 MHz, chloroform-*d*, 298 K) spectrum of compound **12b**

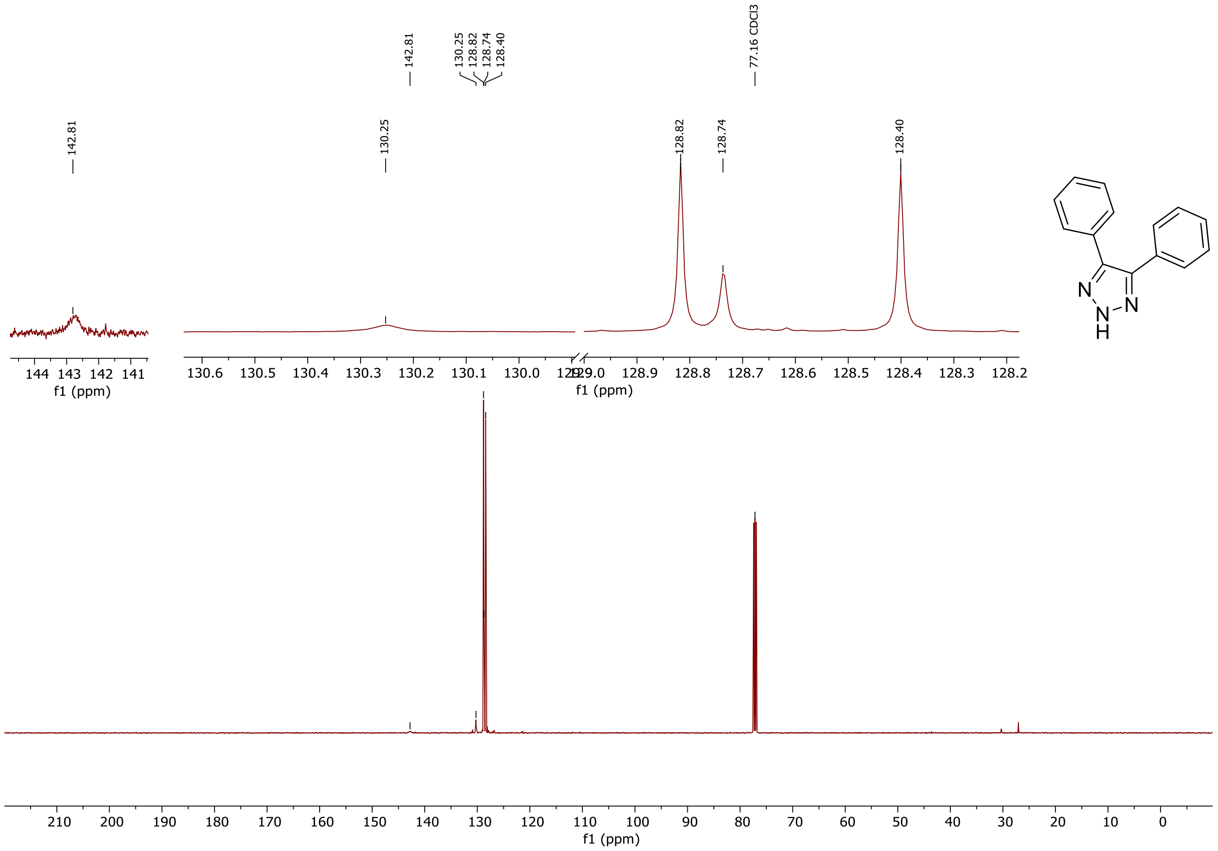

Fig. S343 ^{13}C{^{1}H} NMR(126 MHz, chloroform-*d*, 298 K) spectrum of compound **12b**

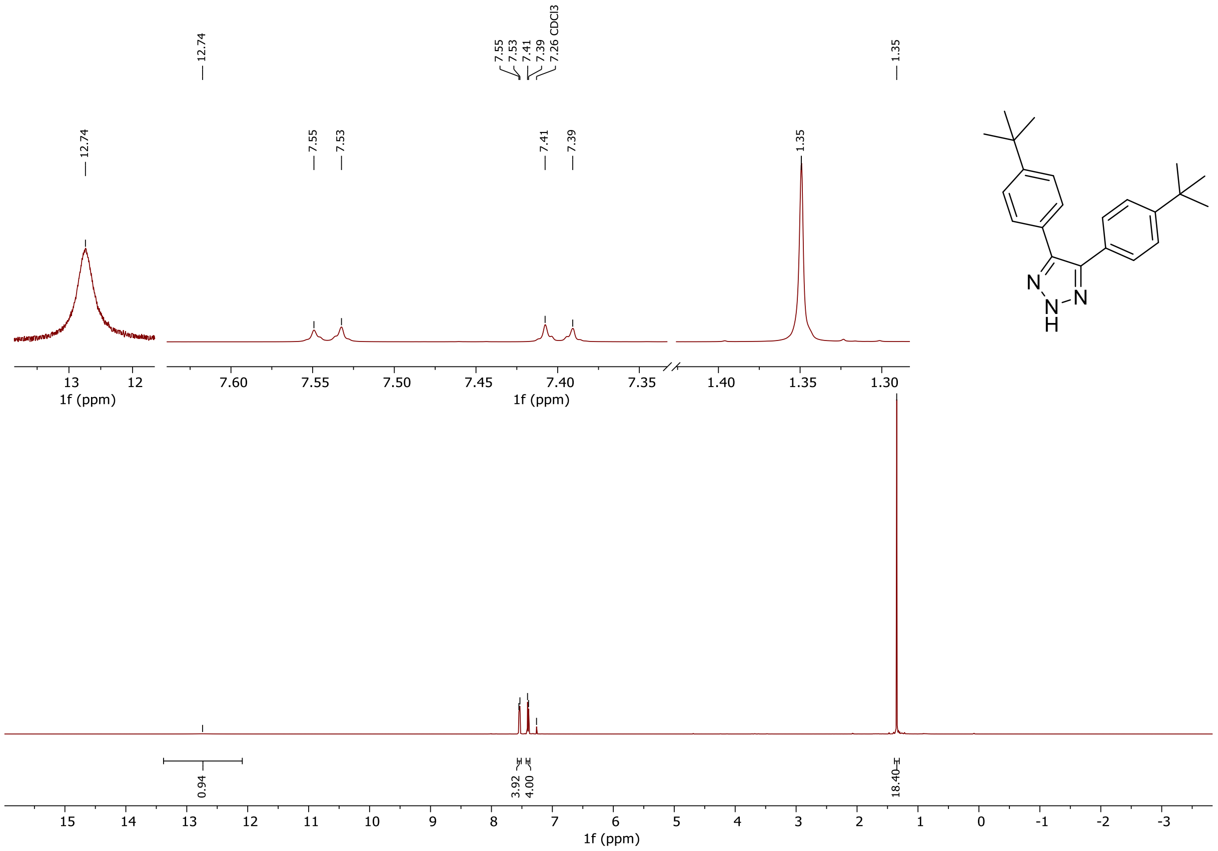

Fig. S344 ^{1}H NMR (501 MHz, chloroform-*d*, 298 K) spectrum of compound **12c**

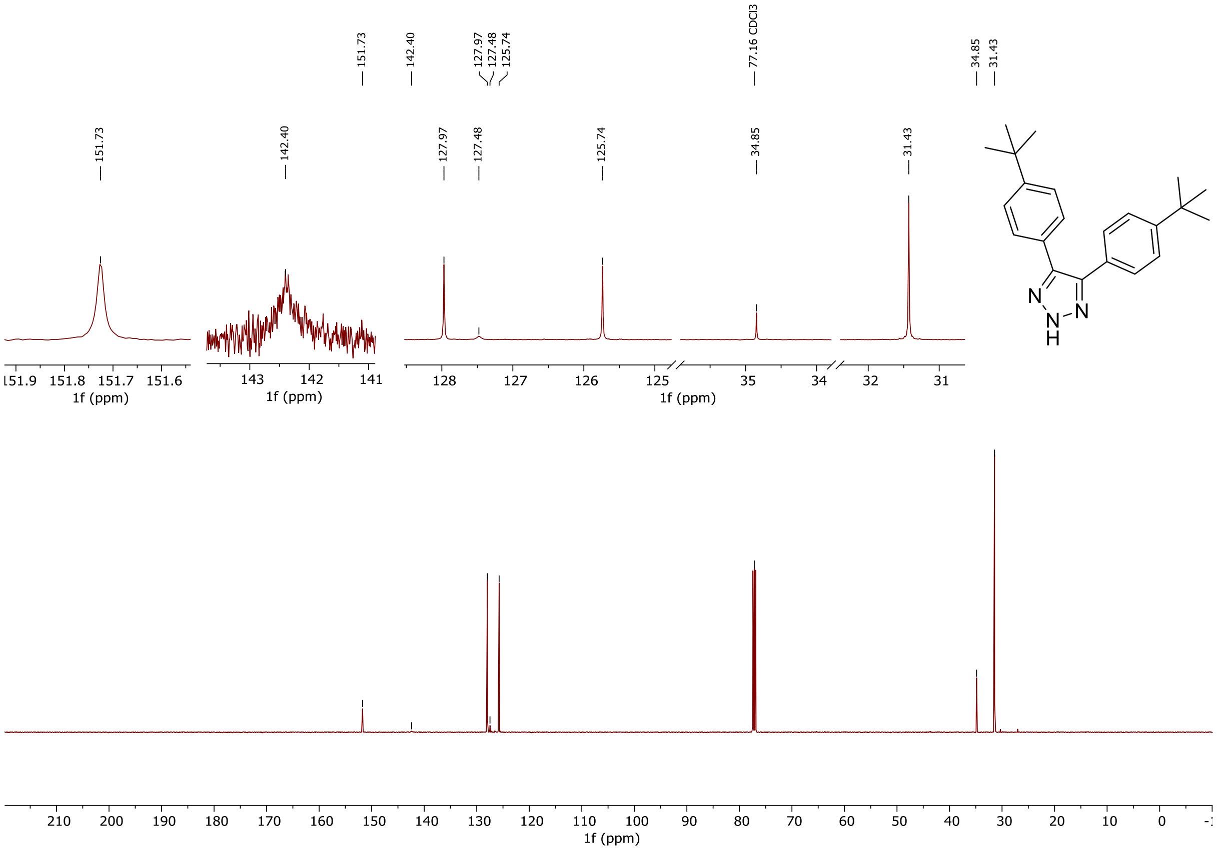

Fig. S345 $^{13}C\{^{1}H\}$ NMR (126 MHz, chloroform-*d*, 298 K) spectrum of compound **12c**

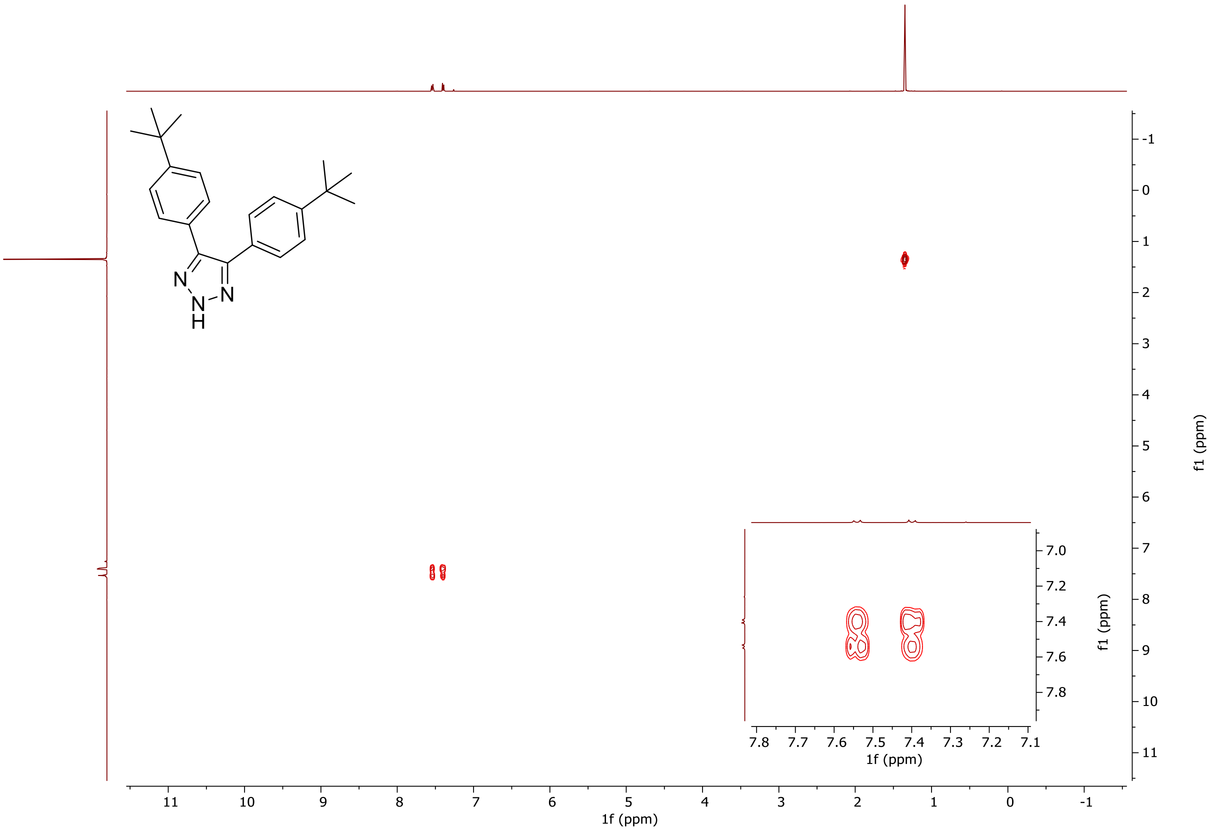

Fig. S346 COSY (501 MHz, 501 MHz, chloroform-*d*, 298 K) spectrum of compound **12c**

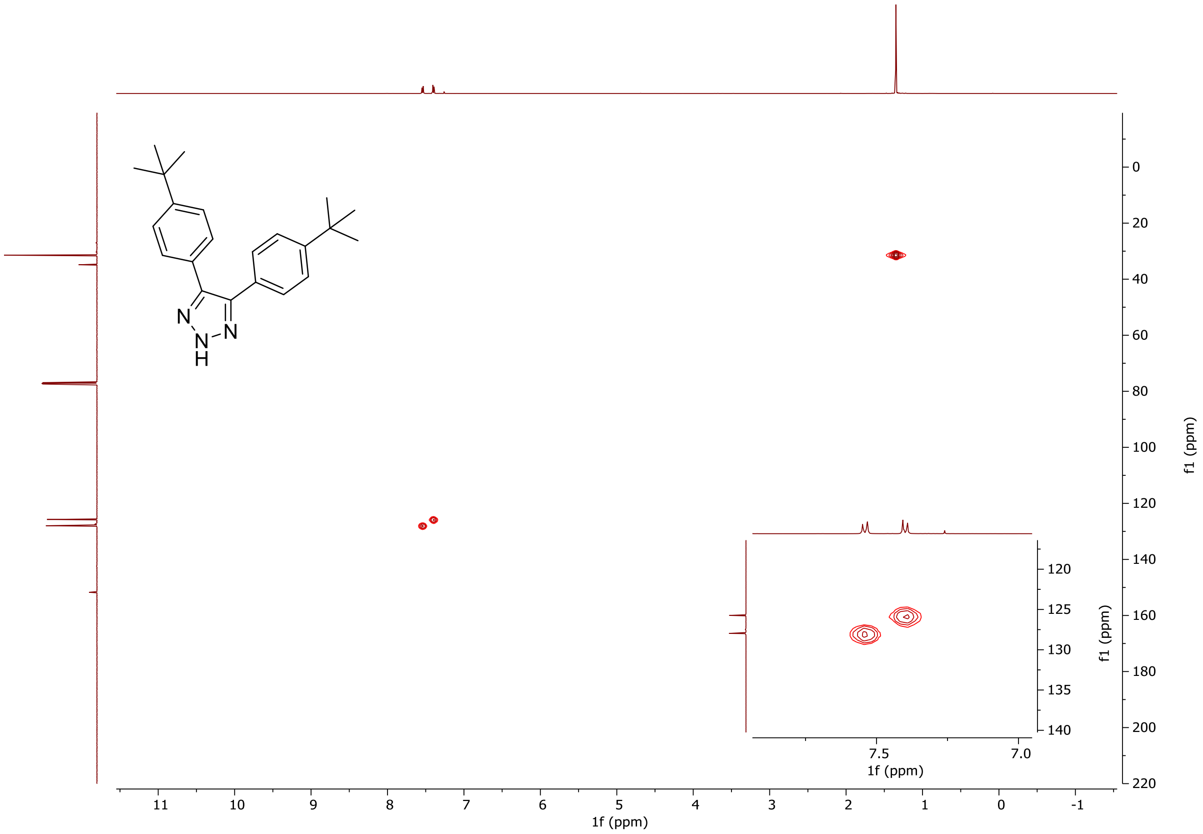

Fig. S347 HSQC (501 MHz, 126 MHz, chloroform-*d*, 298 K) spectrum of compound **12c**

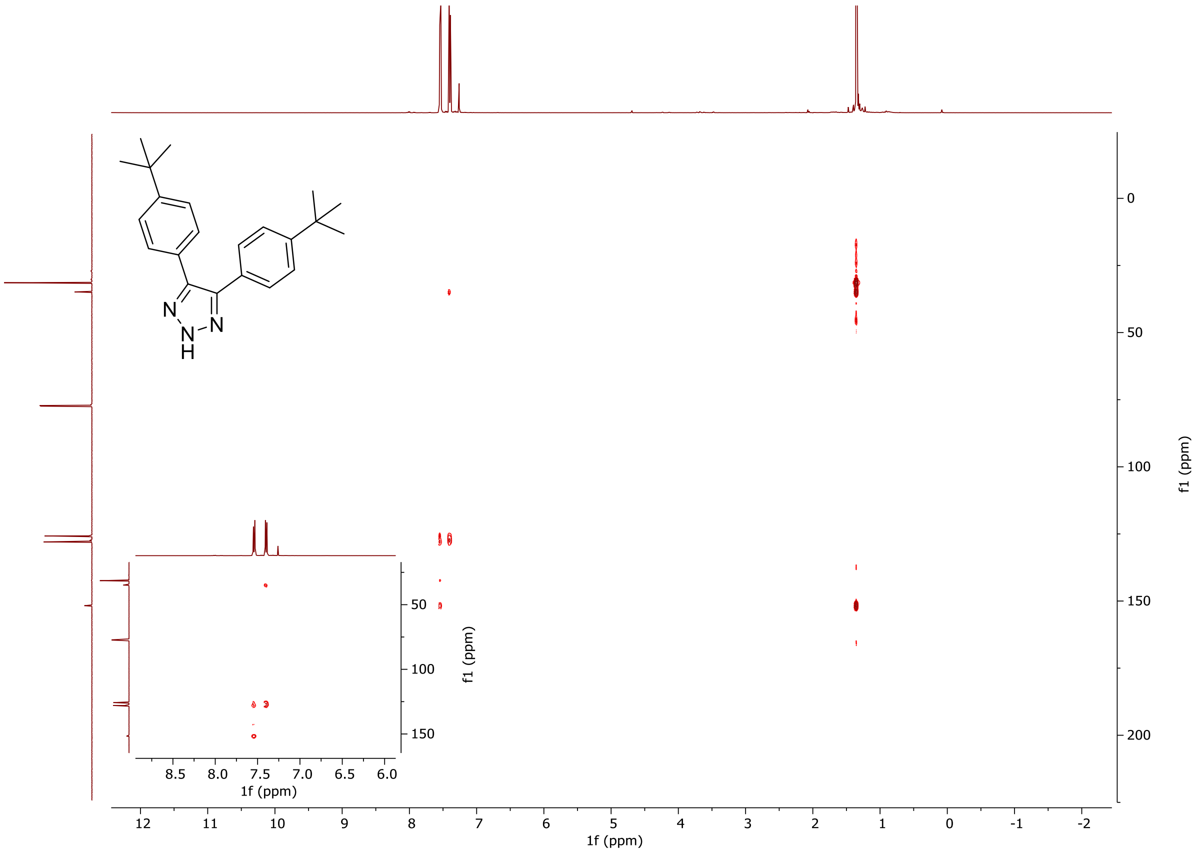

Fig. S348 HMBC (501 MHz, 126 MHz, chloroform-*d*, 298 K) spectrum of compound **12c**

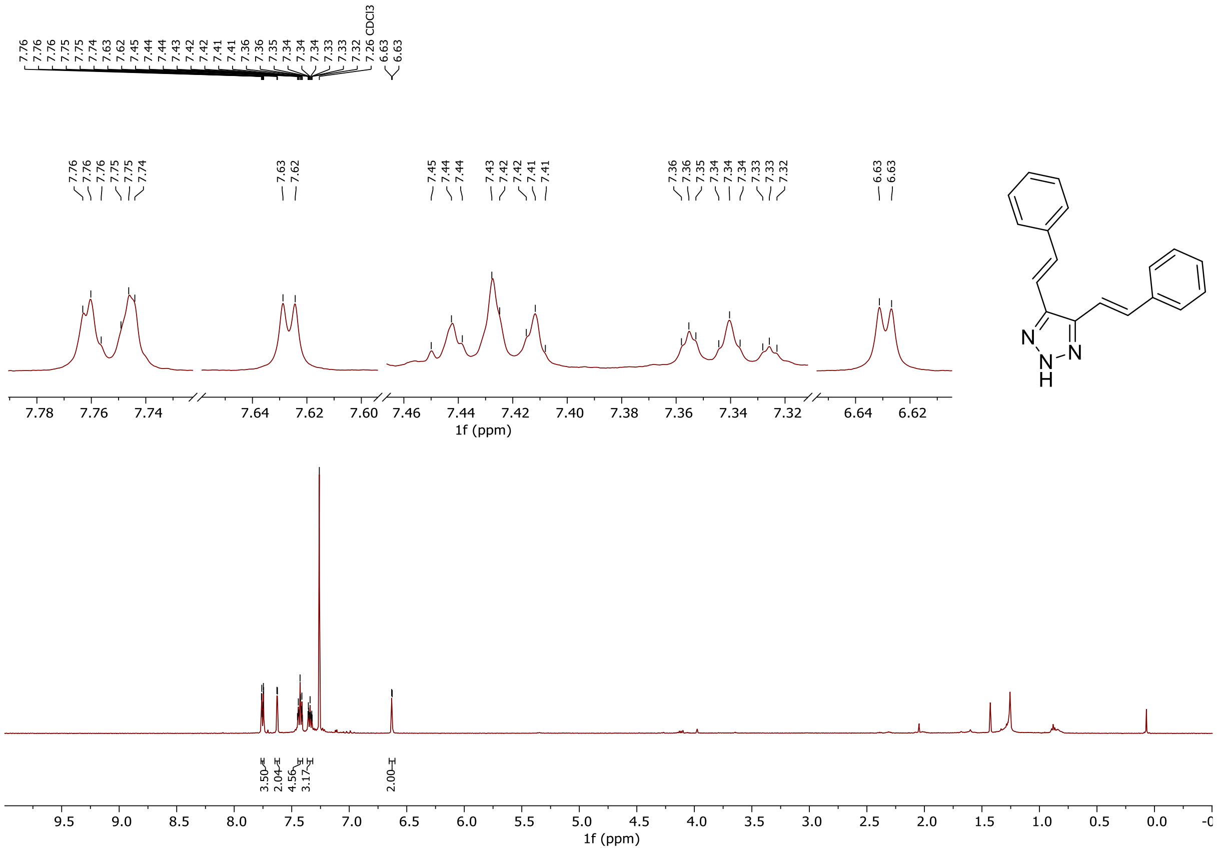

Fig. S349 ^{1}H NMR (501 MHz, chloroform-*d*, 298 K) spectrum of compound **12d**

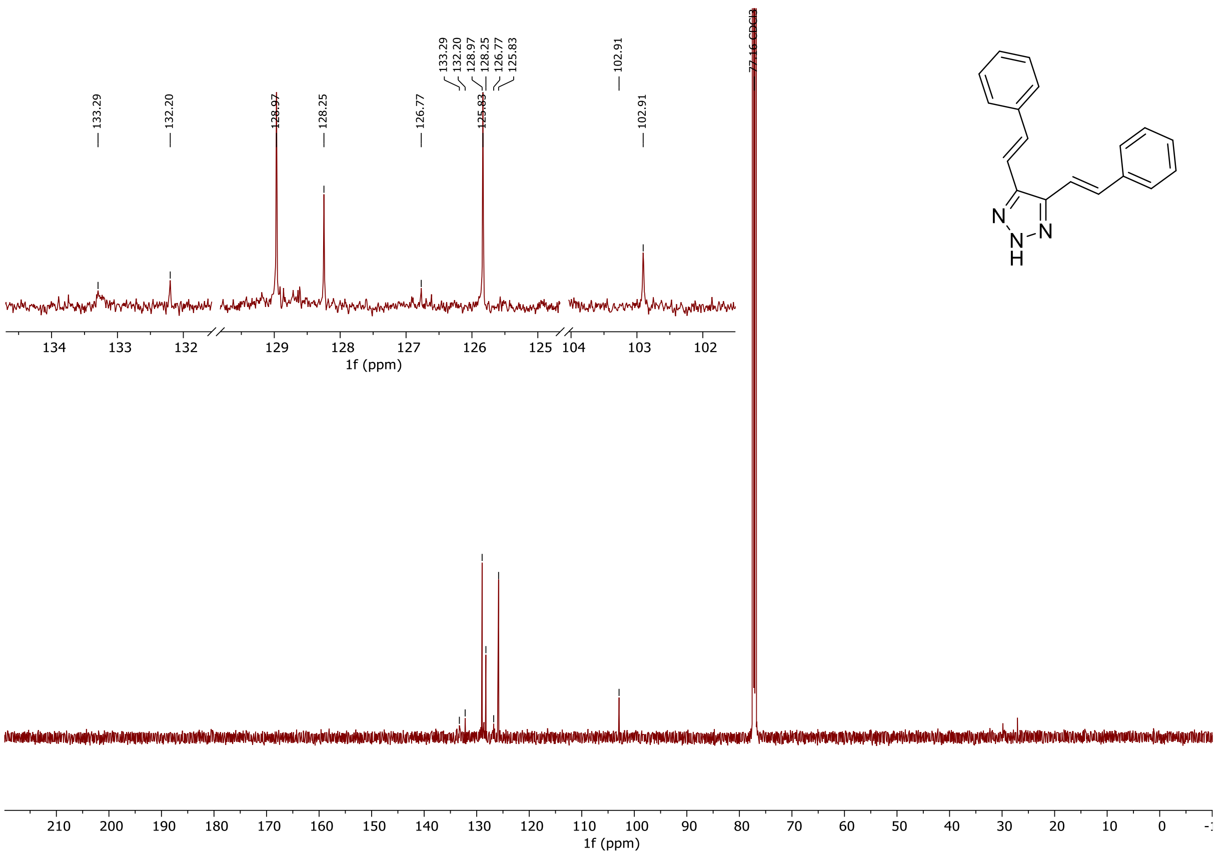

Fig. S350 ^{13}C{^{1}H} NMR (126 MHz, chloroform-*d*, 298 K) spectrum of compound **12d**

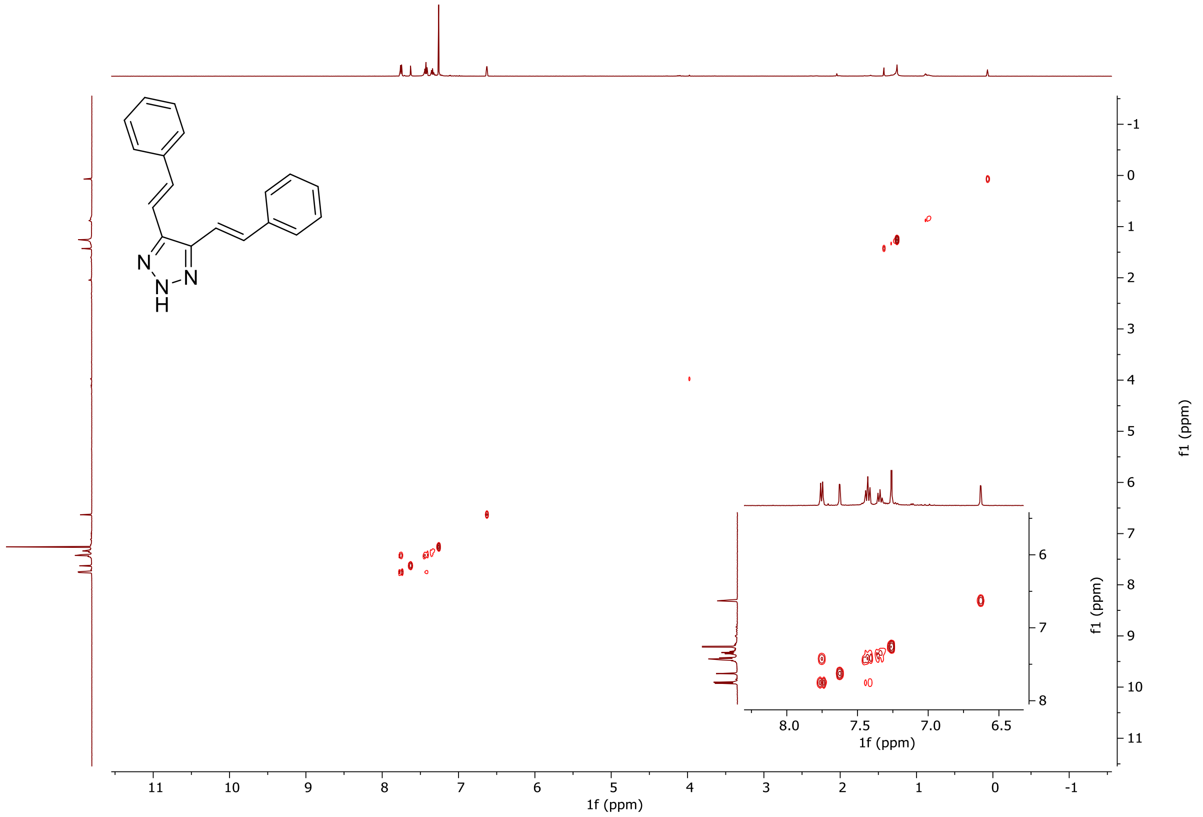

Fig. S351 COSY (501 MHz, 501 MHz, chloroform-*d*, 298 K) spectrum of compound **12d**

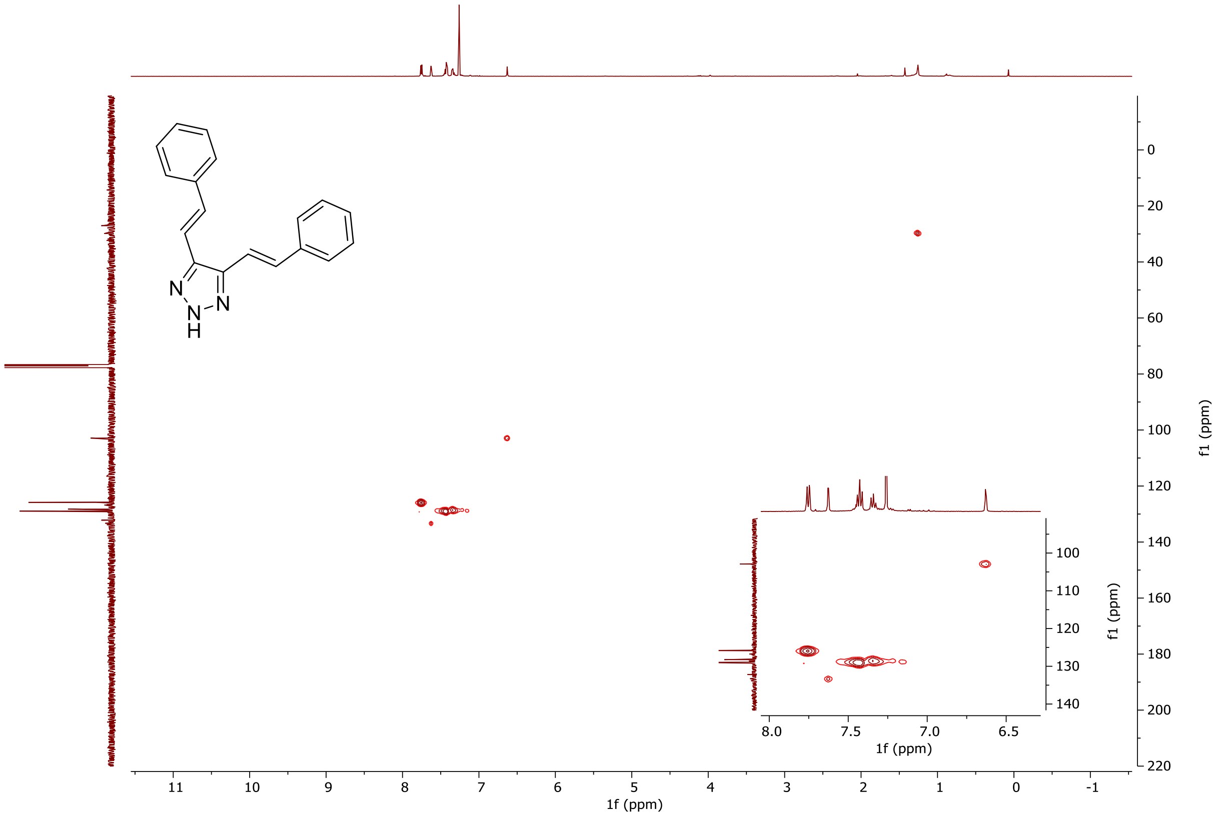

Fig. S352 HSQC (501 MHz, 126 MHz, chloroform-*d*, 298 K) spectrum of compound **12d**

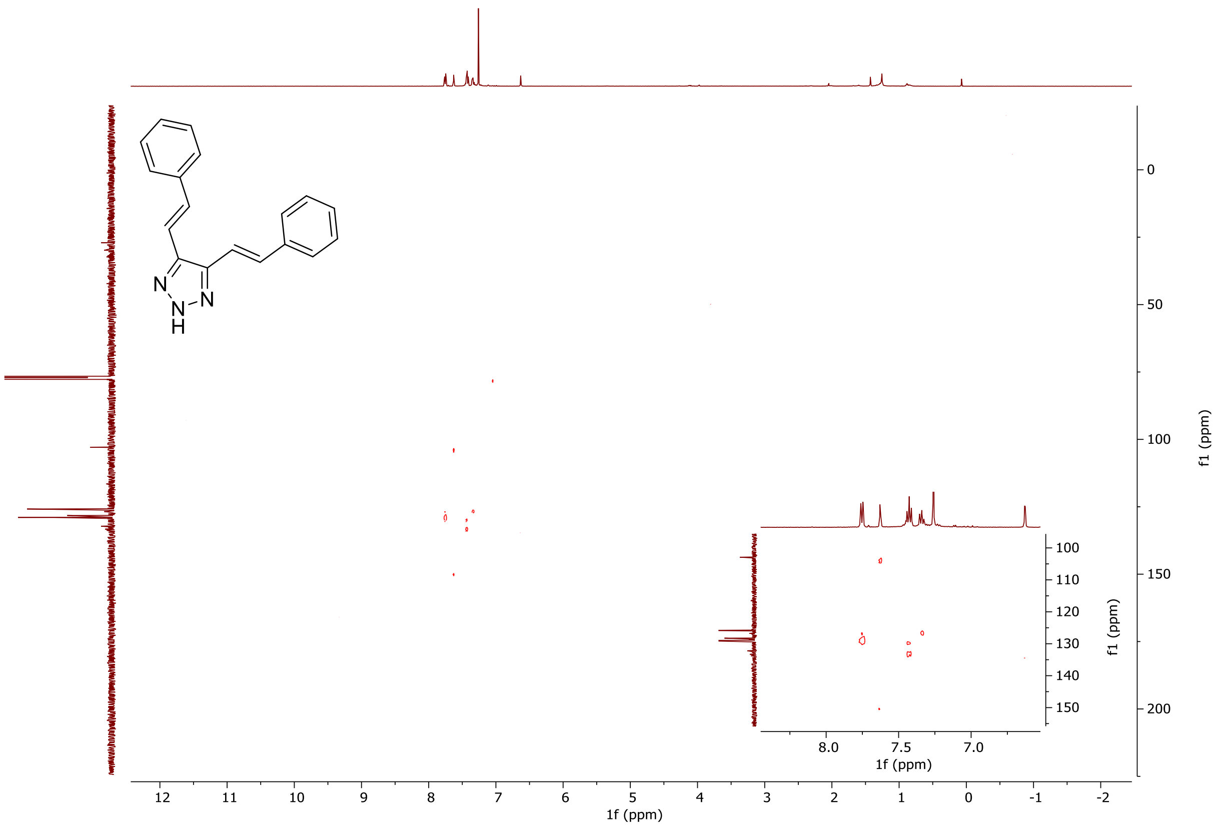

Fig. S353 HMBC (501 MHz, 126 MHz, chloroform-*d*, 298 K) spectrum of compound **12d**

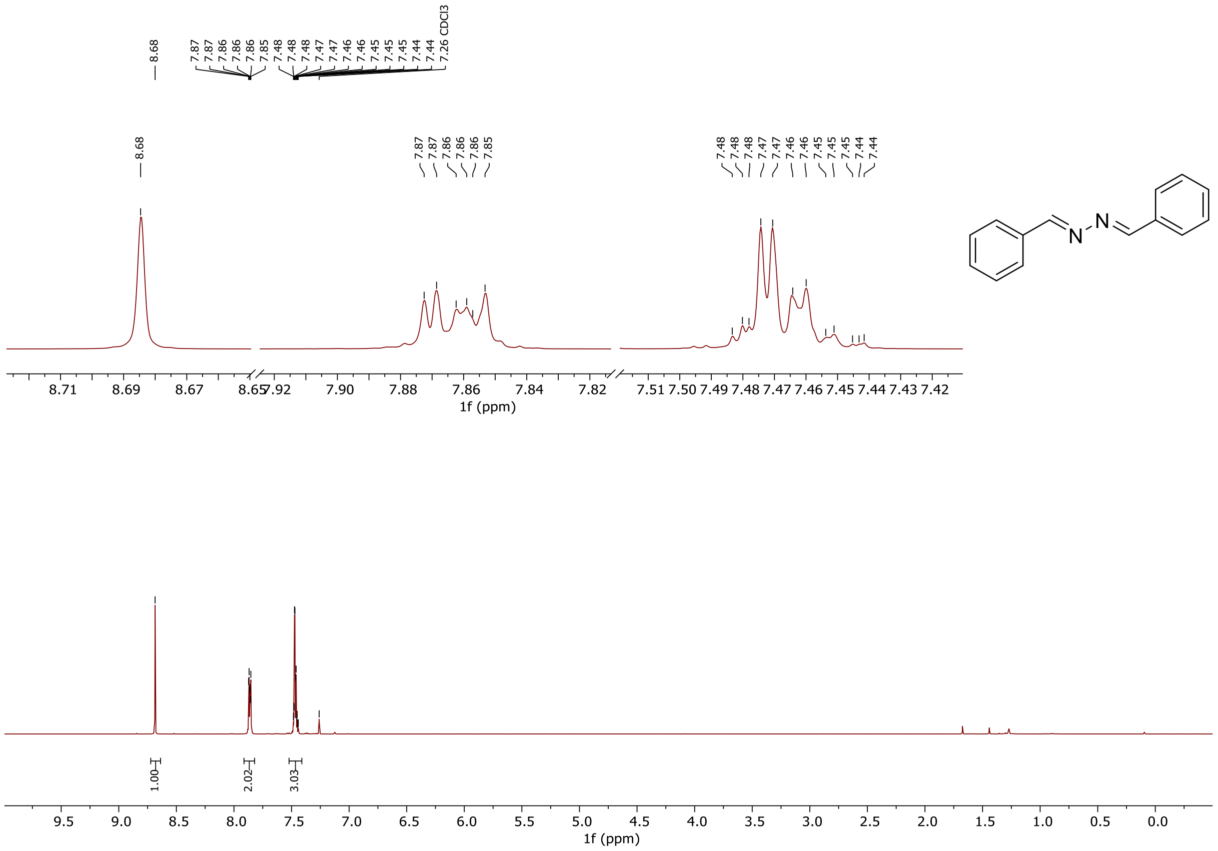

Fig. S354 ^{1}H NMR (501 MHz, chloroform-*d*, 298 K) spectrum of compound **13a**

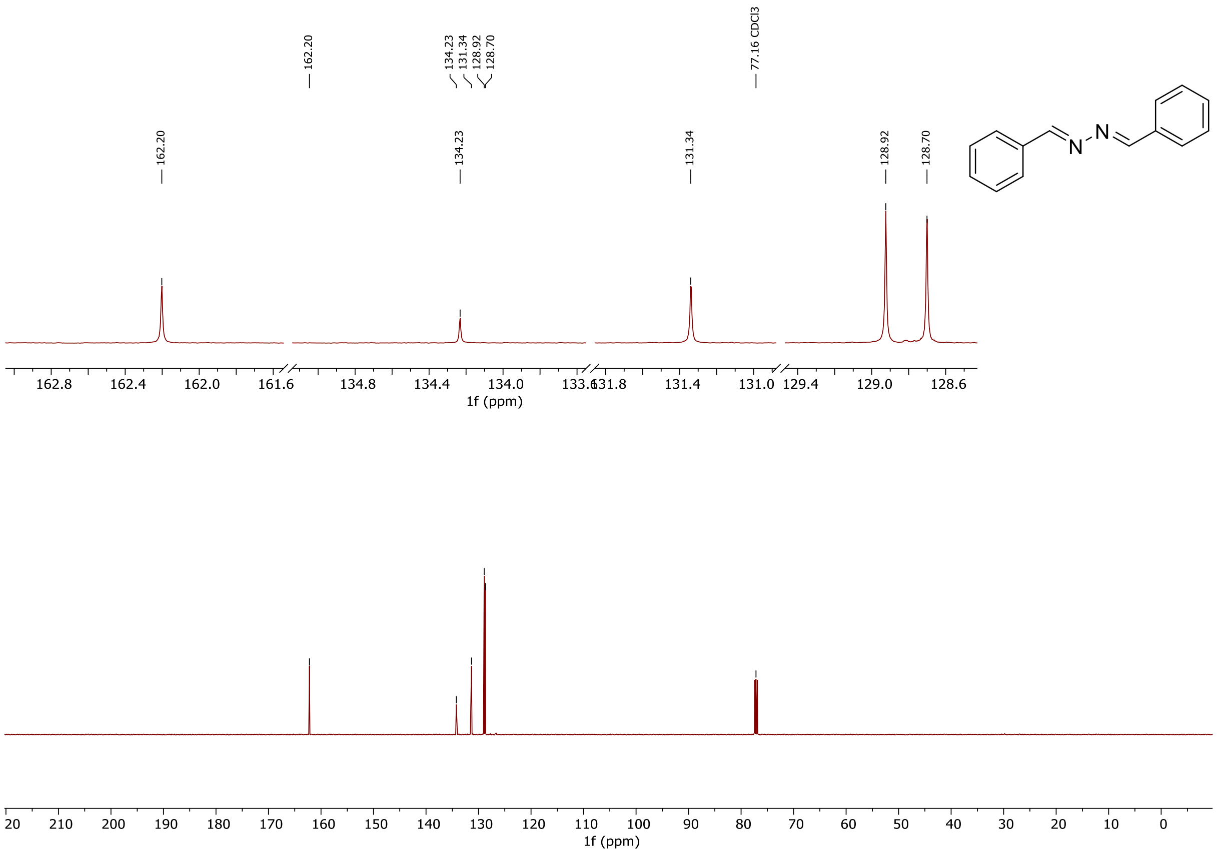

Fig. S355 ^{13}C{1H} NMR (126 MHz, chloroform-*d*, 298 K) spectrum of compound **13a**

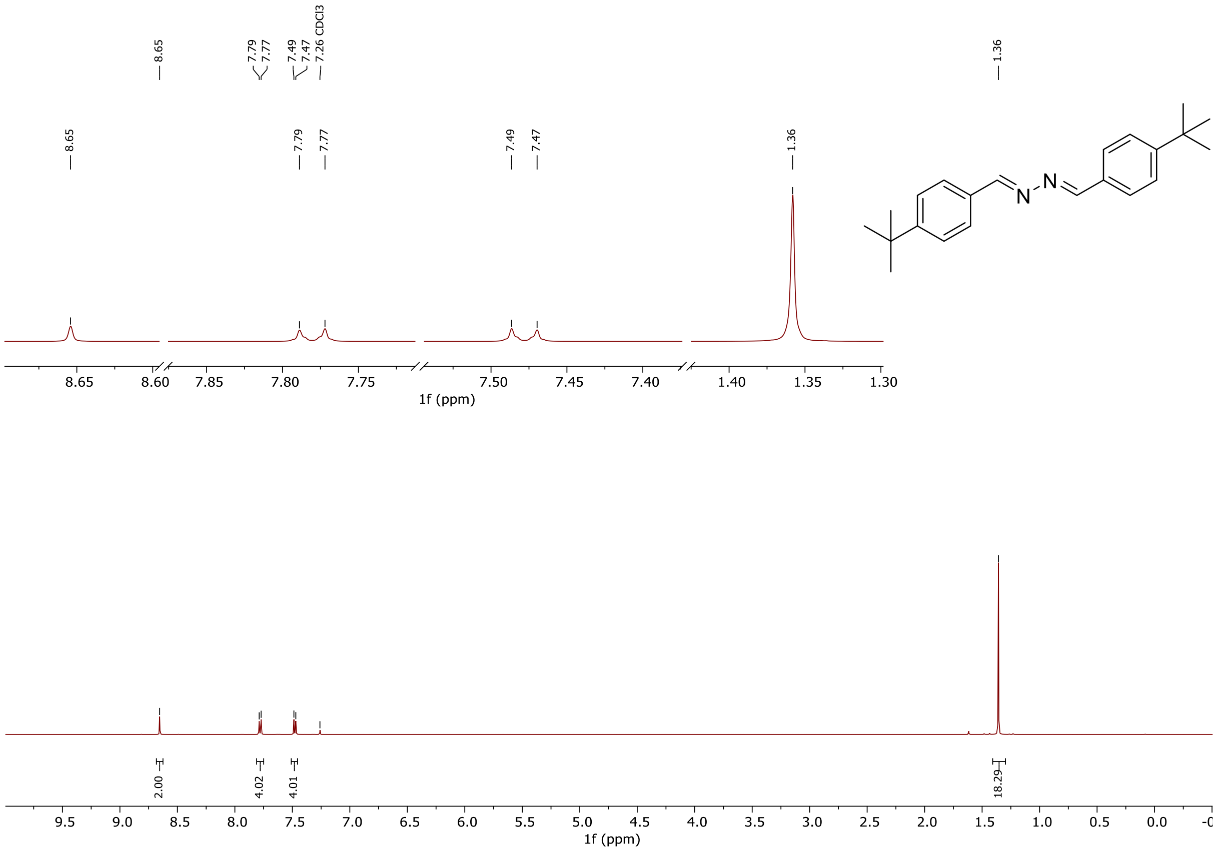

Fig. S356 ^{1}H NMR (126 MHz, chloroform-*d*, 298 K) spectrum of compound **13b**

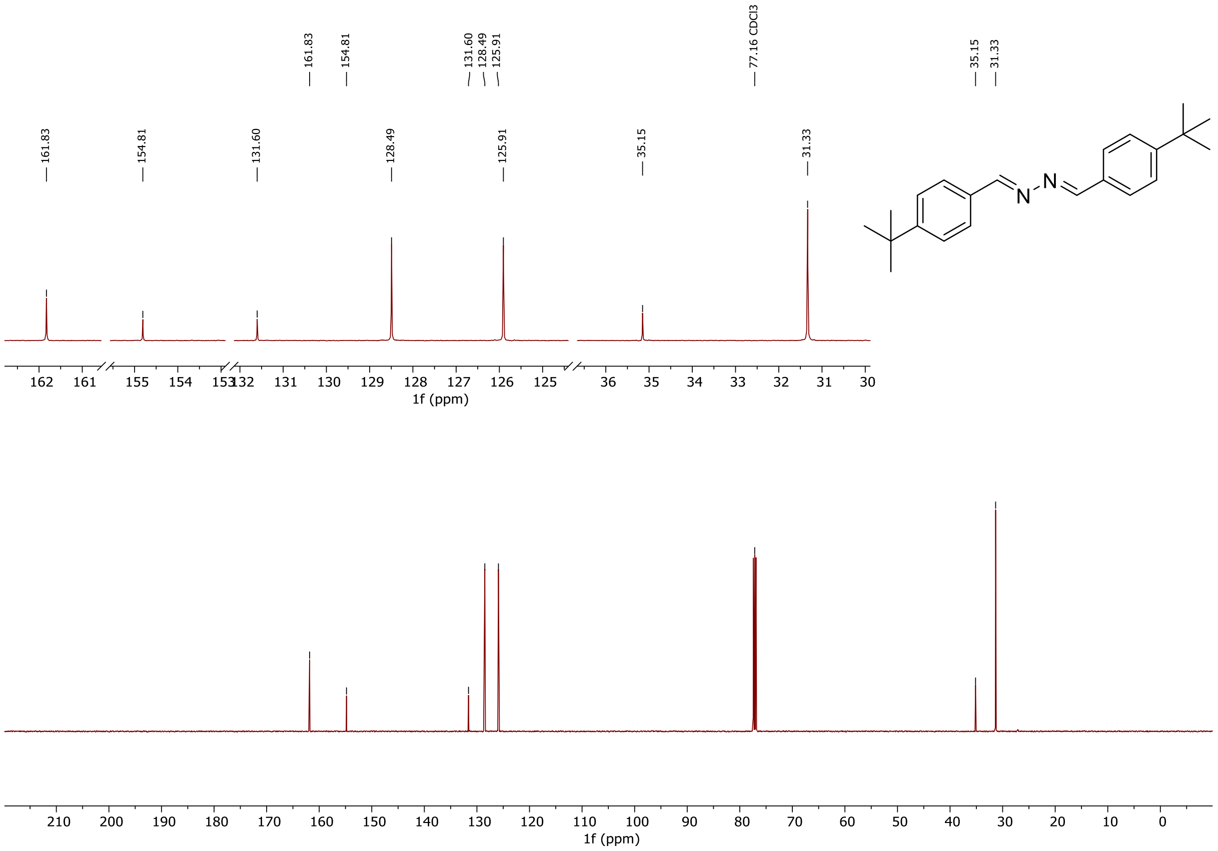

Fig. S357 ^{13}C{^{1}H} NMR (126 MHz, chloroform-*d*, 298 K) spectrum of compound **13b**

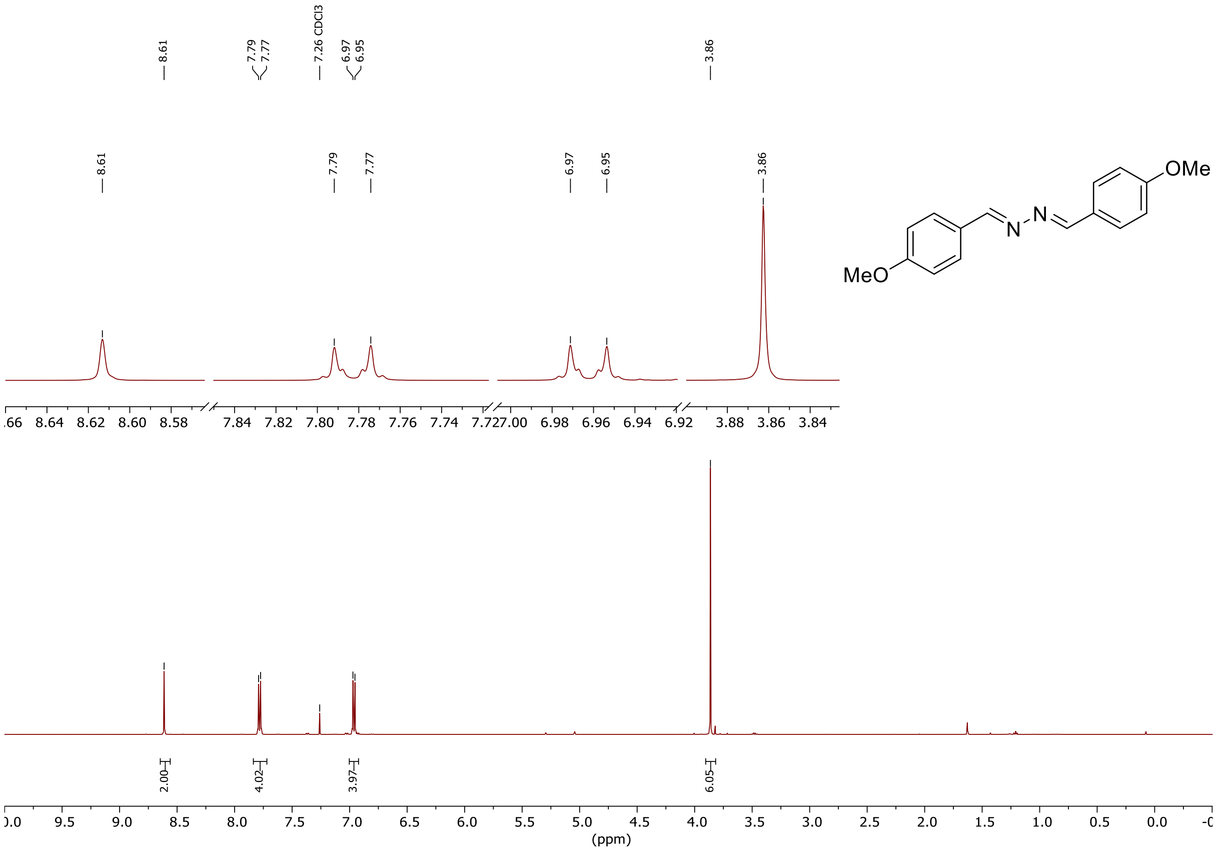

Fig. S358 ¹H NMR (501 MHz, chloroform-*d*, 298 K) spectrum of compound **13c**

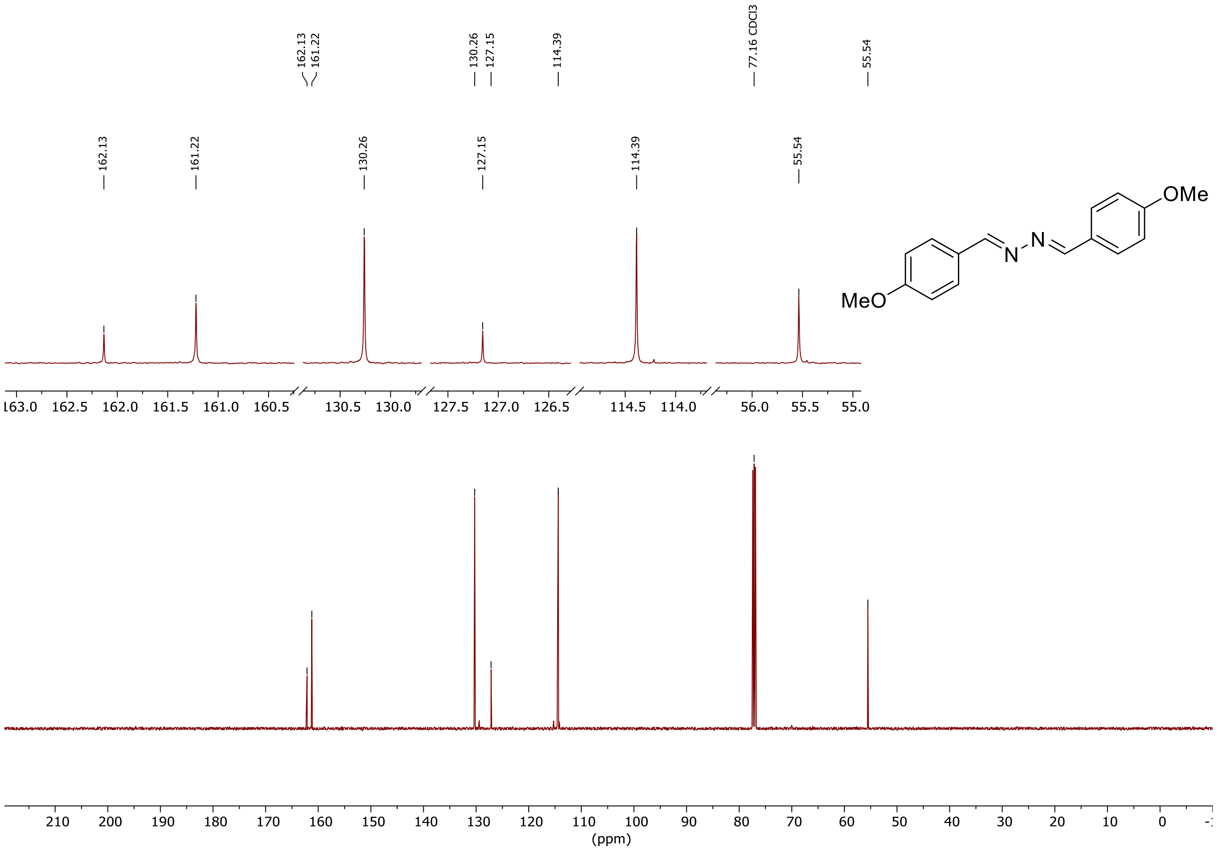

Fig. S359 ¹³C{¹H} NMR (126 MHz, chloroform-*d*, 298 K) spectrum of compound **13c**

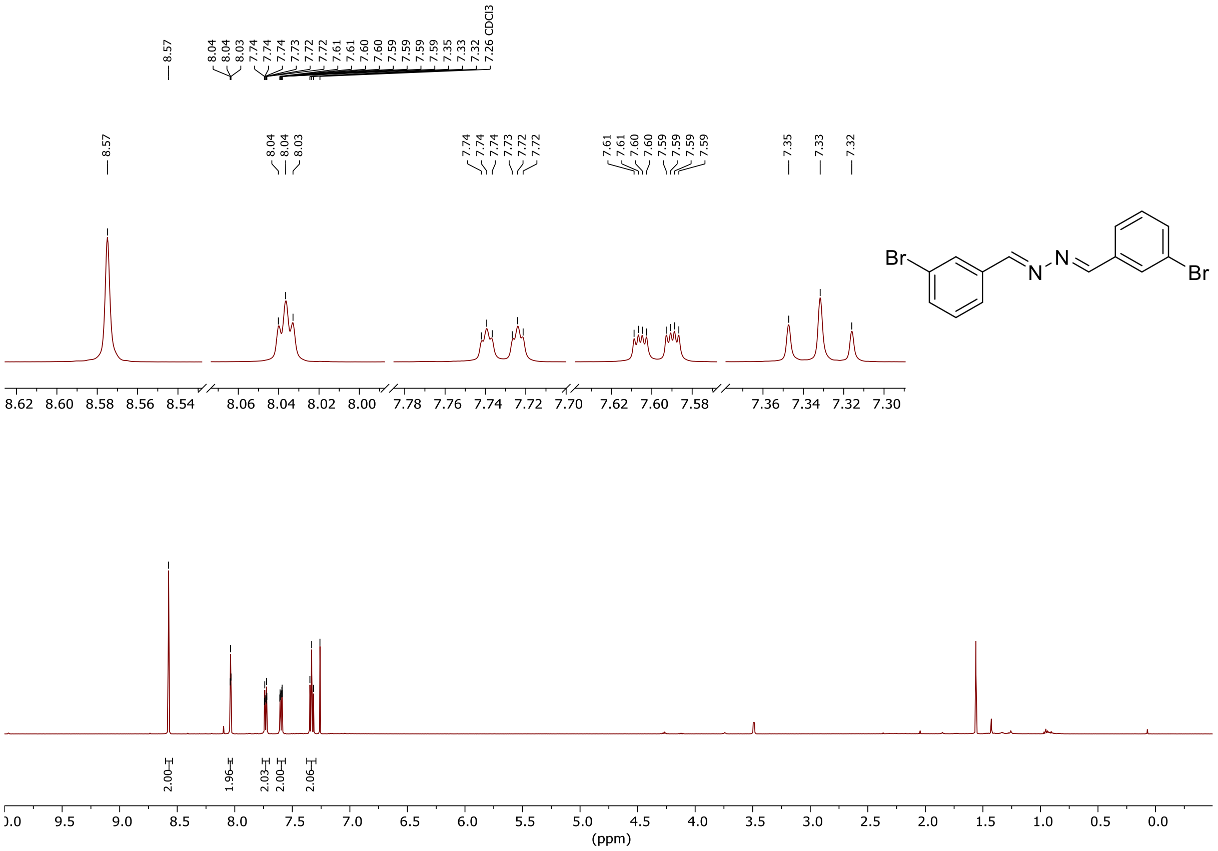

Fig. S360 ^{1}H NMR (501 MHz, chloroform-*d*, 298 K) spectrum of compound **13d**

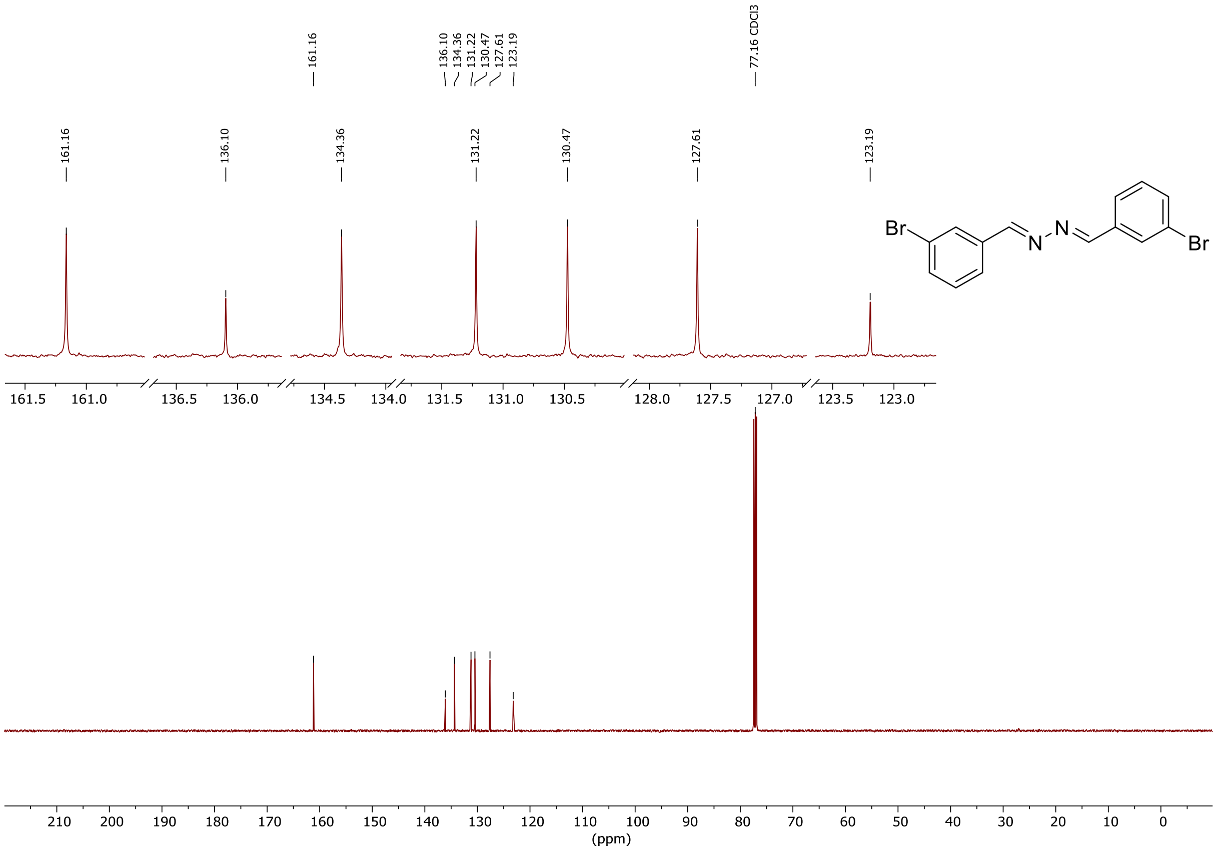

Fig. S361 ^{13}C{^{1}H} NMR (501 MHz, chloroform-*d*, 298 K) spectrum of compound **13d**

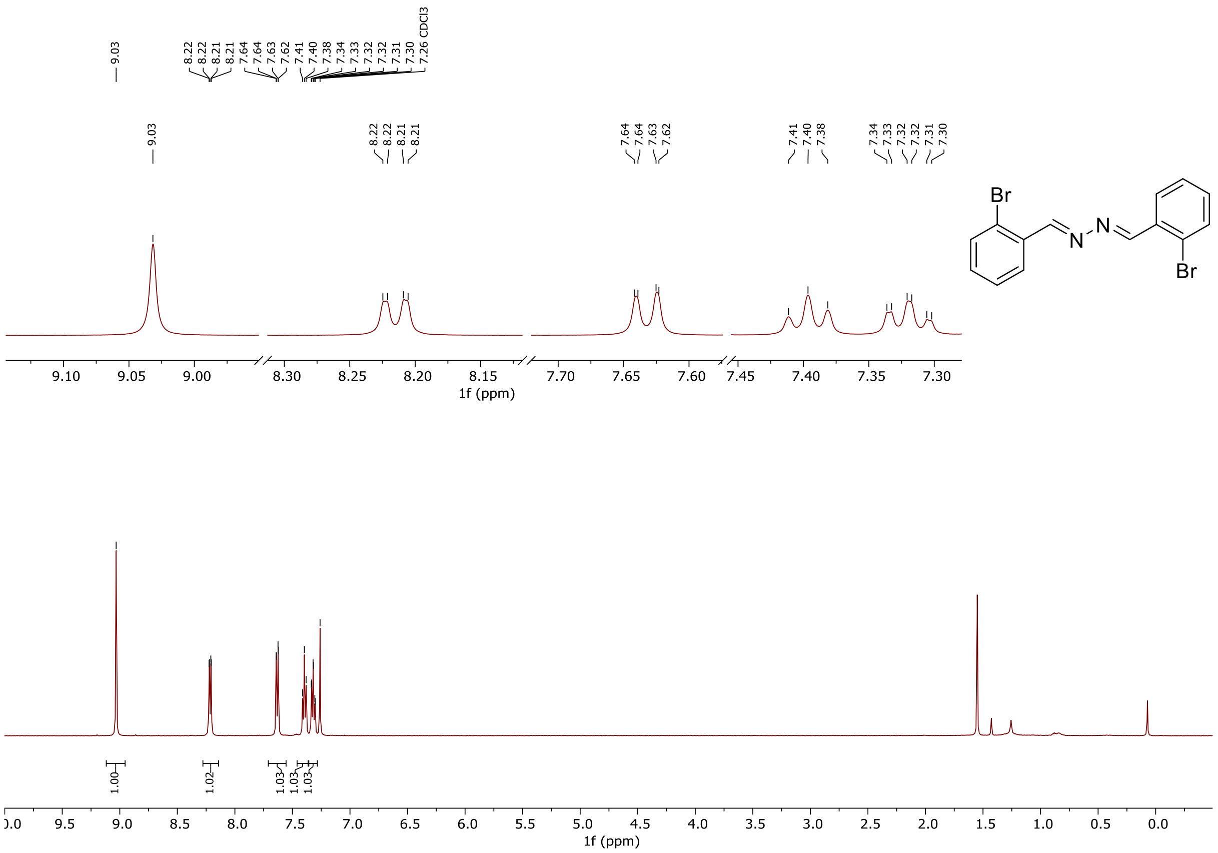

Fig. S362 ^{1}H NMR (501 MHz, chloroform-*d*, 298 K) spectrum of compound **13e**

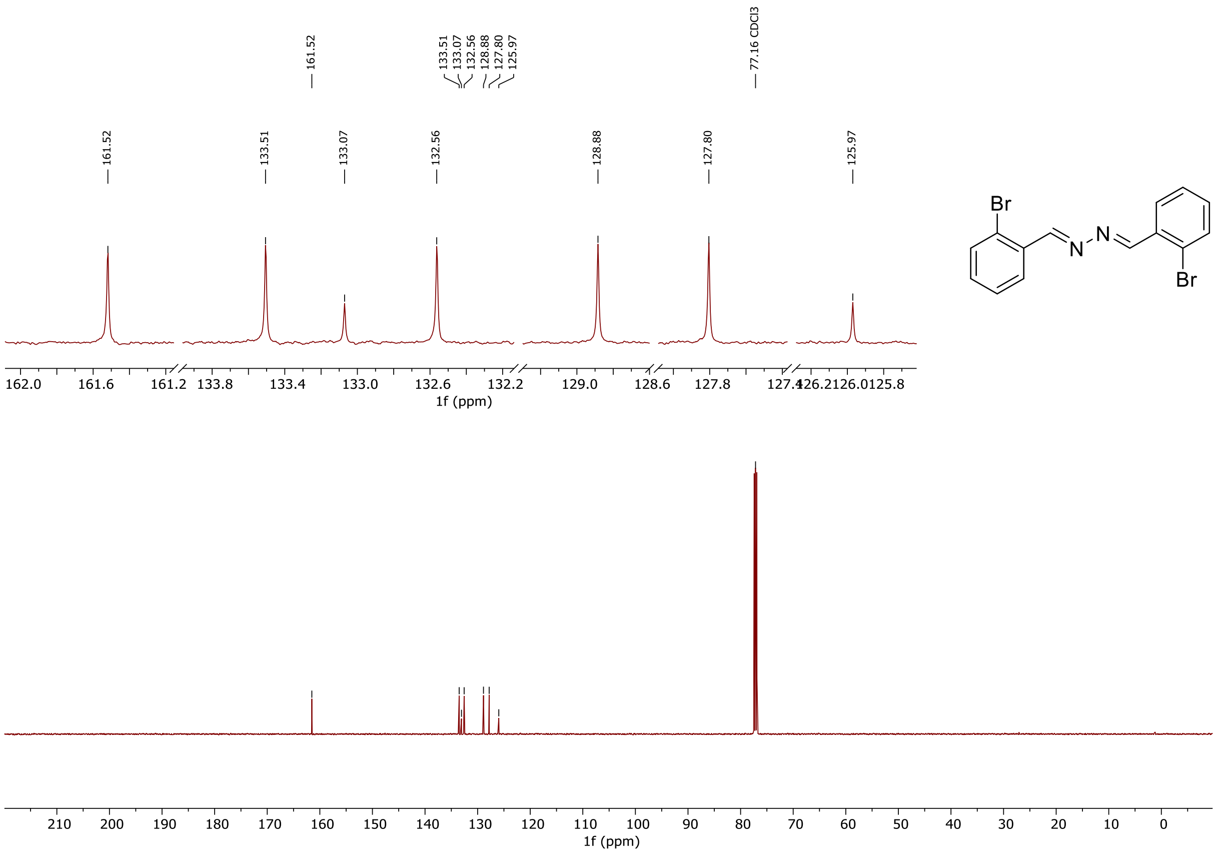

Fig. S363 ^{13}C{^{1}H} NMR (126 MHz, chloroform-*d*, 298 K) spectrum of compound **13e**

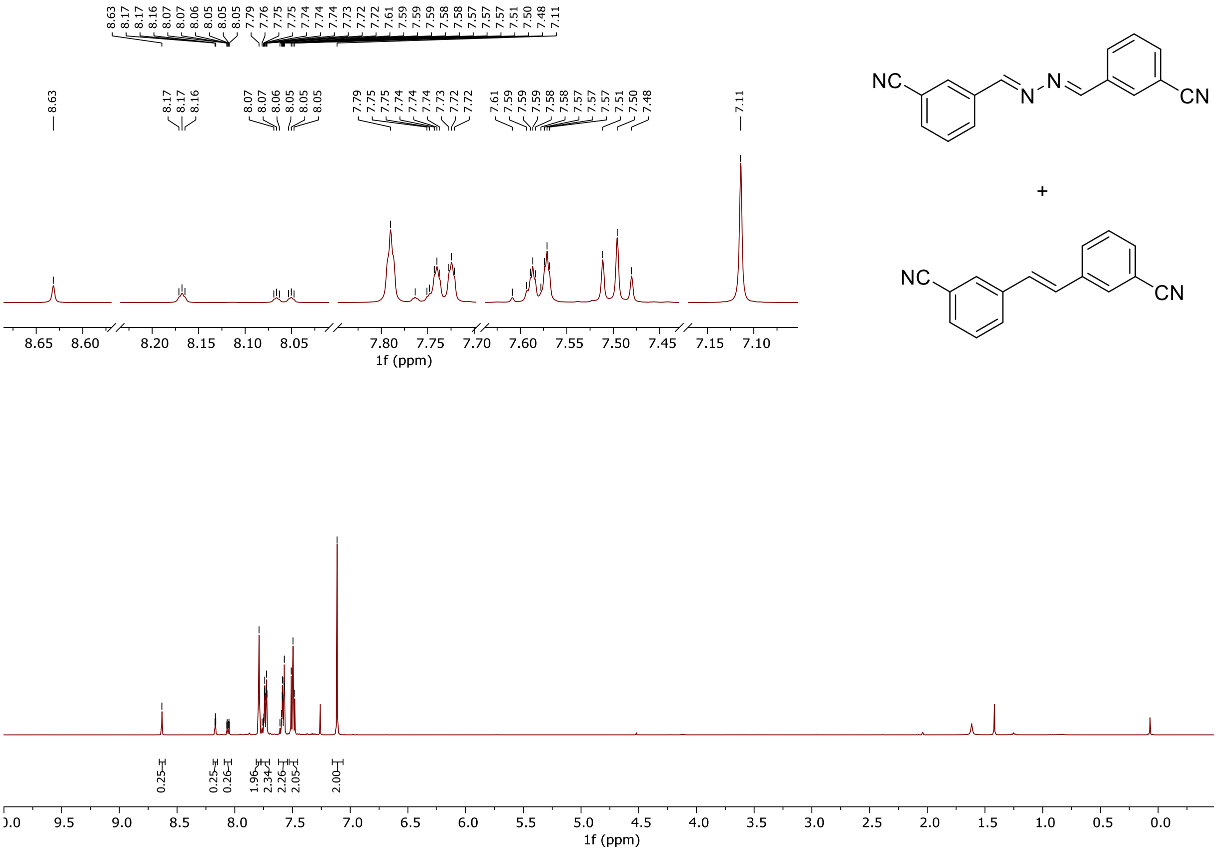

Fig. S364 ^{1}H NMR (501 MHz, chloroform-*d*, 298 K) spectrum of compound **13f** + **14f**

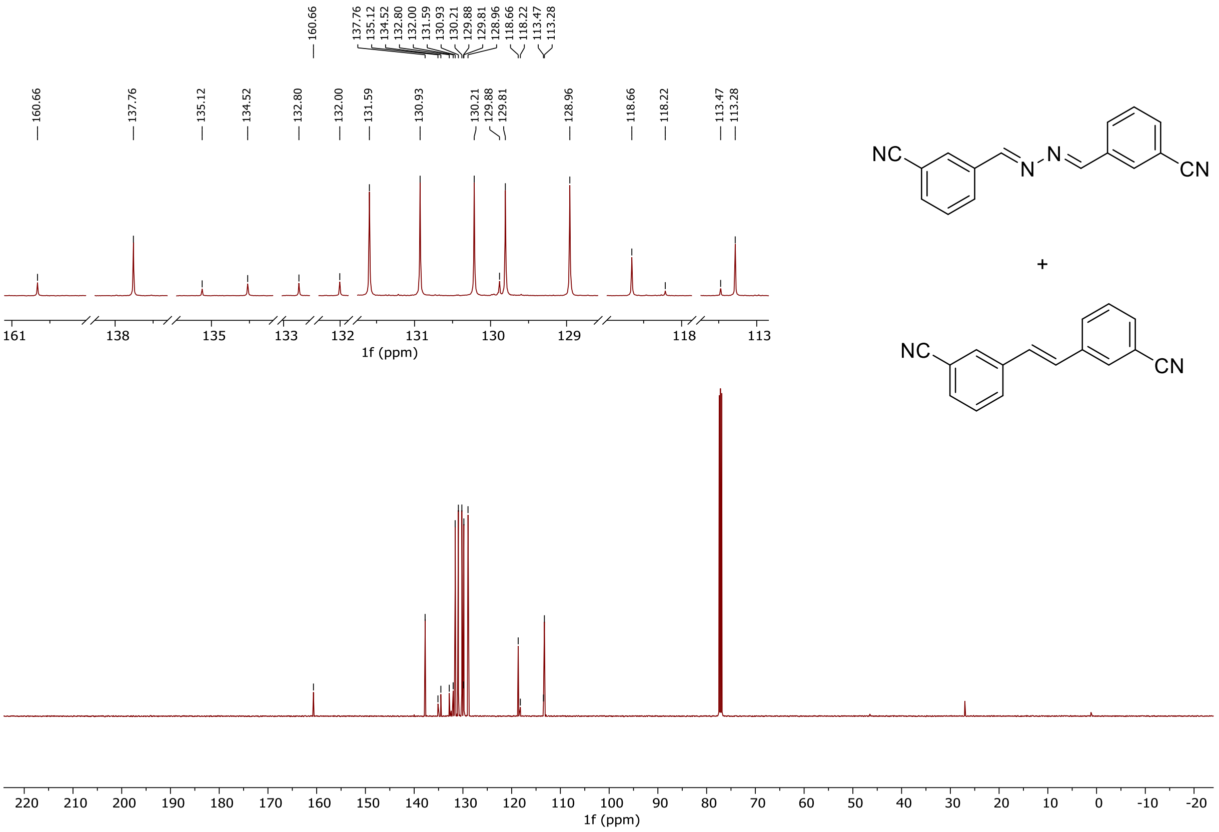

Fig. S365 ^{13}C{^{1}H} NMR (126 MHz, chloroform-*d*, 298 K) spectrum of compound **13f** + **14f**

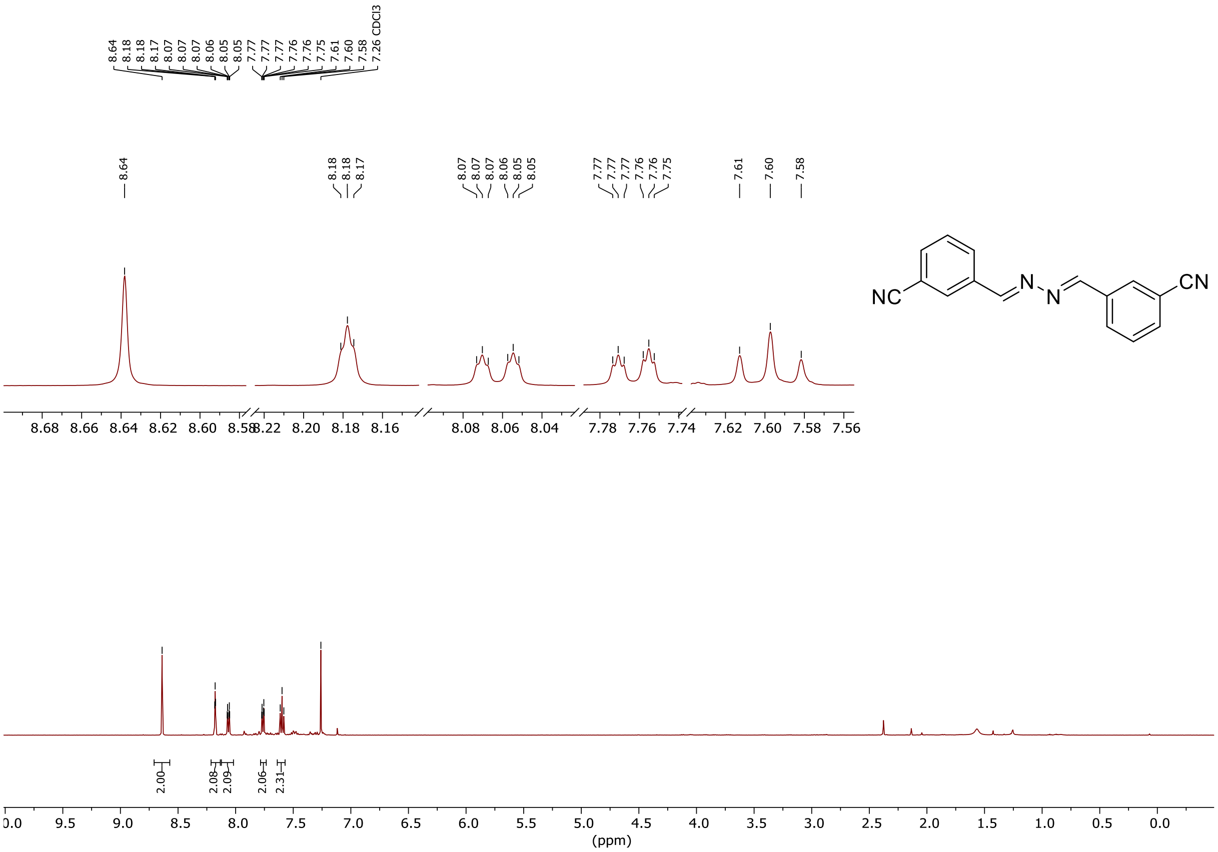

Fig. S366 ^{1}H NMR (501 MHz, chloroform-*d*, 298 K) spectrum of compound **13f**

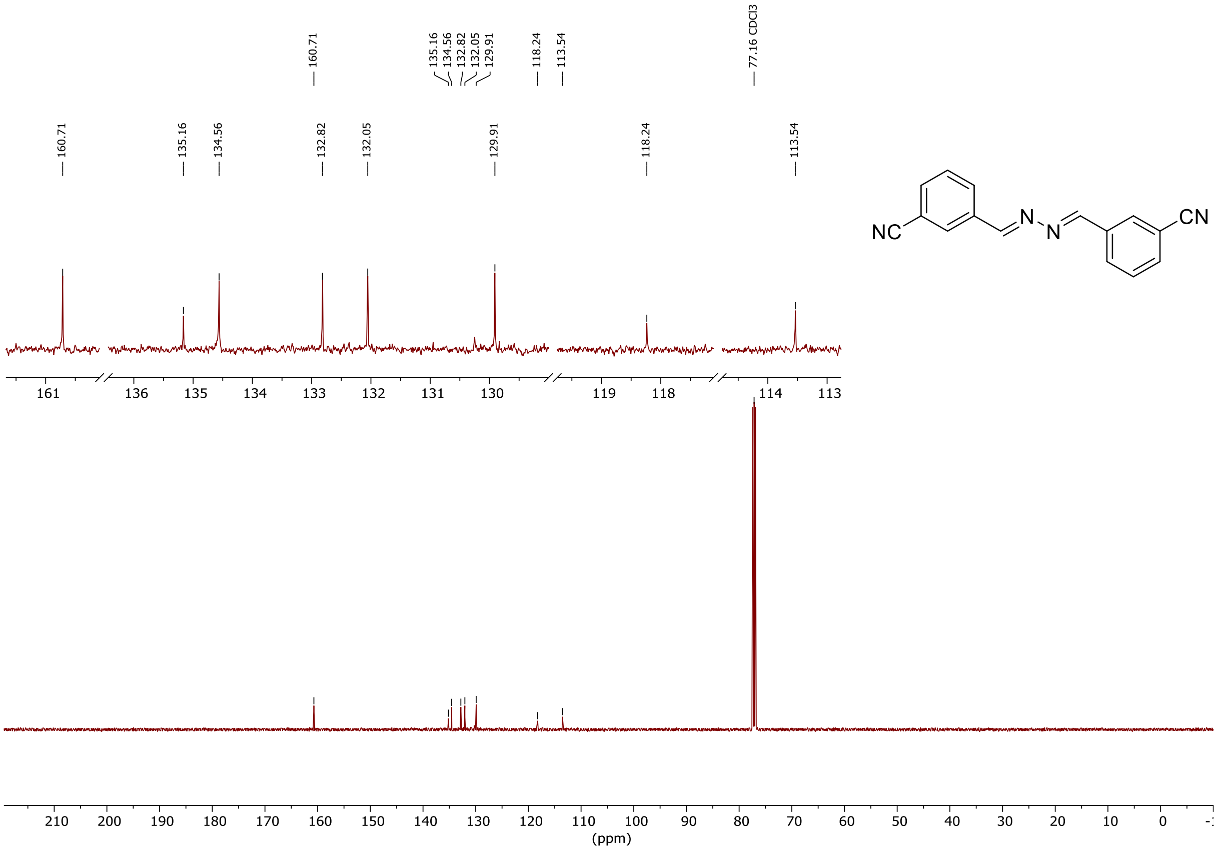

Fig. S367 ^{13}C{^{1}H} NMR (126 MHz, chloroform-*d*, 298 K) spectrum of compound **13f**

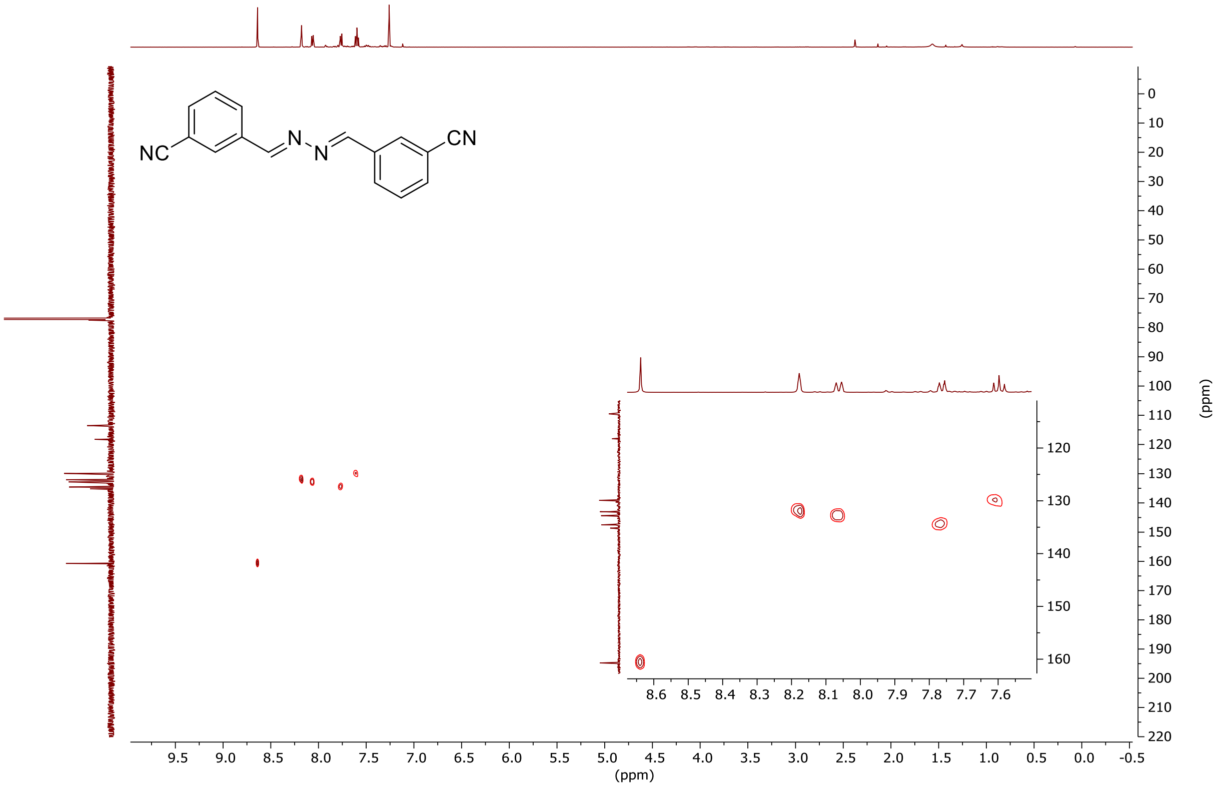

Fig. S368 HSQC (501 MHz, 126 MHz, chloroform-*d*, 298 K) spectrum of compound **13f**

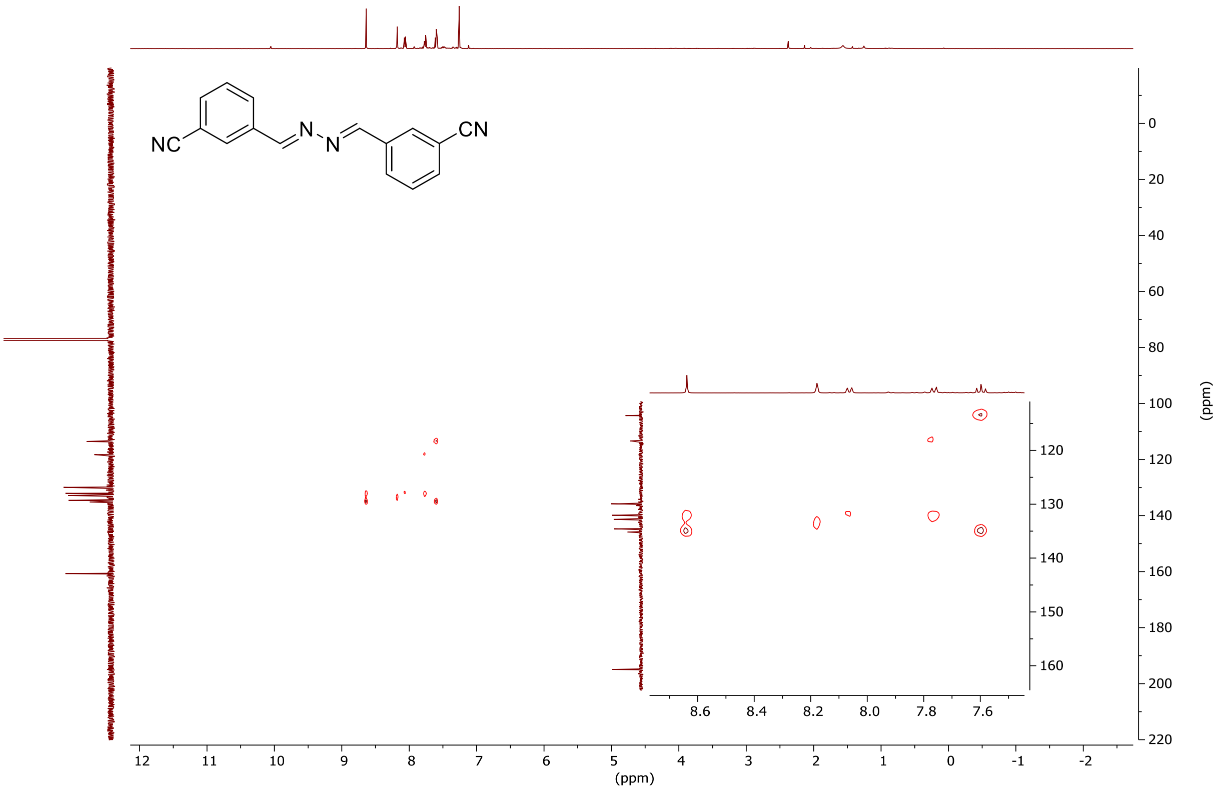

Fig. S369 HMBC (501 MHz, 126 MHz, chloroform-*d*, 298 K) spectrum of compound **13f**

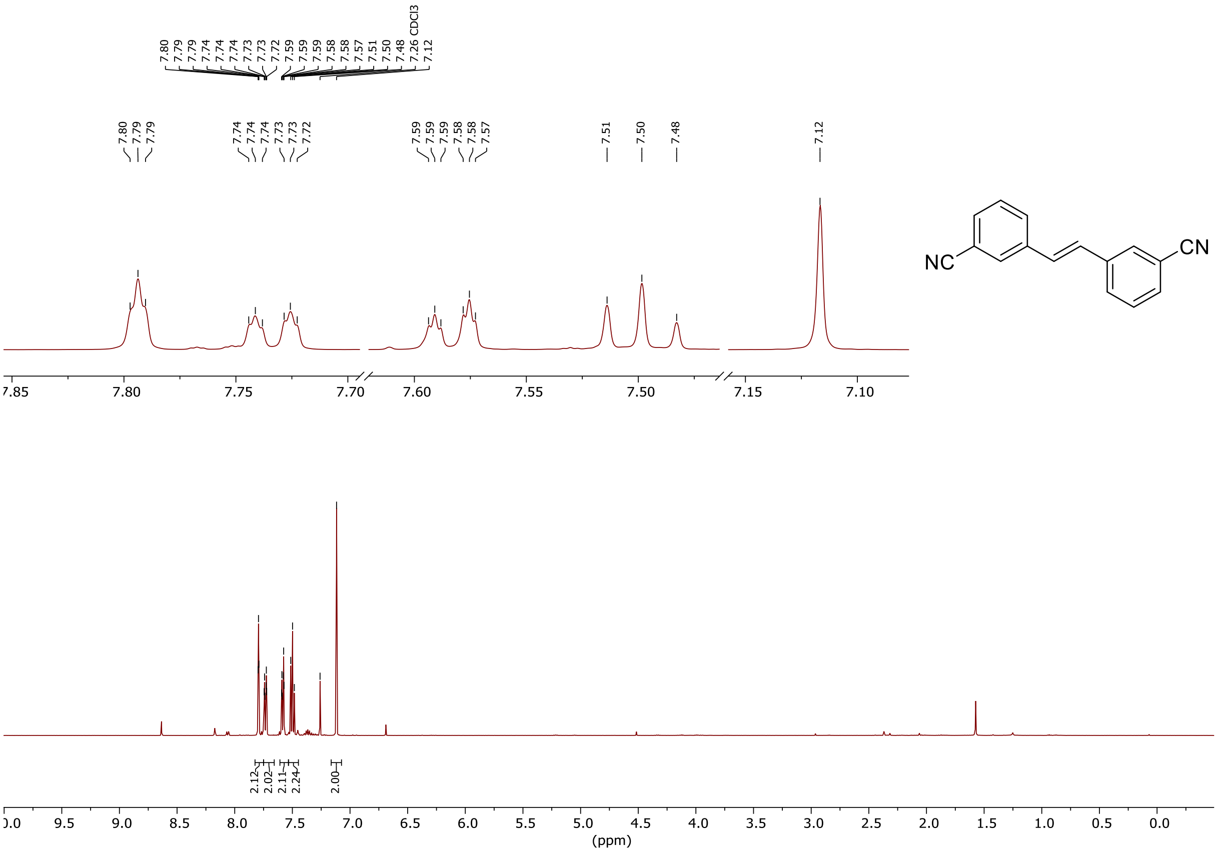

Fig. S370 ^{1}H NMR (501 MHz, chloroform-*d*, 298 K) spectrum of compound **14f**

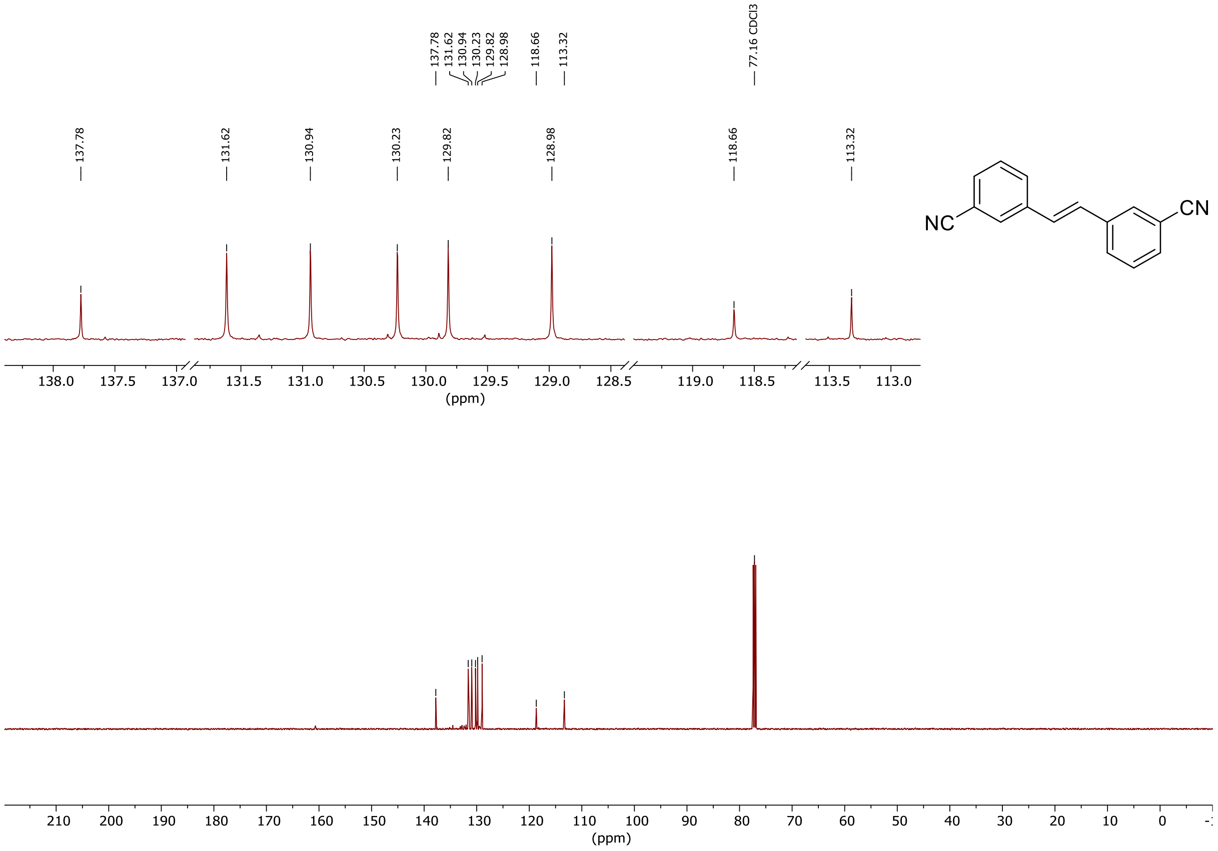

Fig. S371 ^{13}C{^{1}H} NMR (126 MHz, chloroform-*d*, 298 K) spectrum of compound **14f**

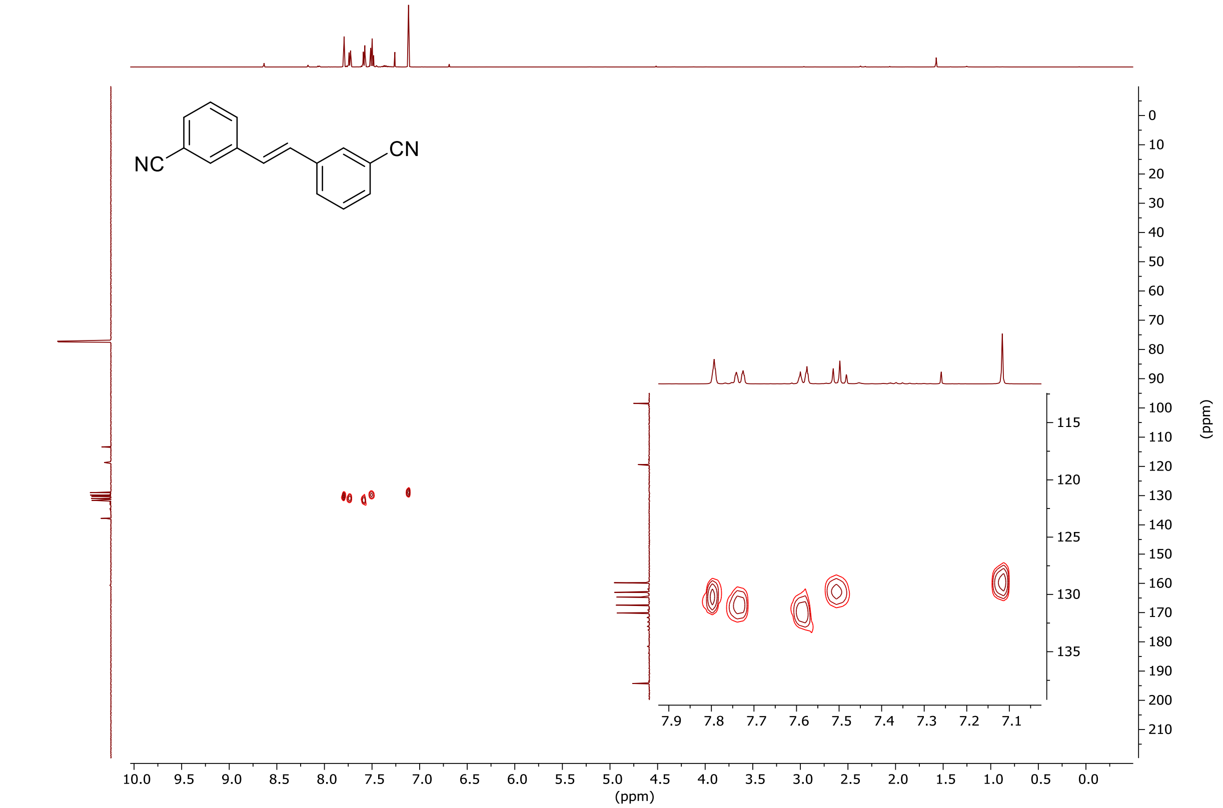

Fig. S372 HSQC (501 MHz, 126 MHz, chloroform-*d*, 298 K) spectrum of compound **14f**

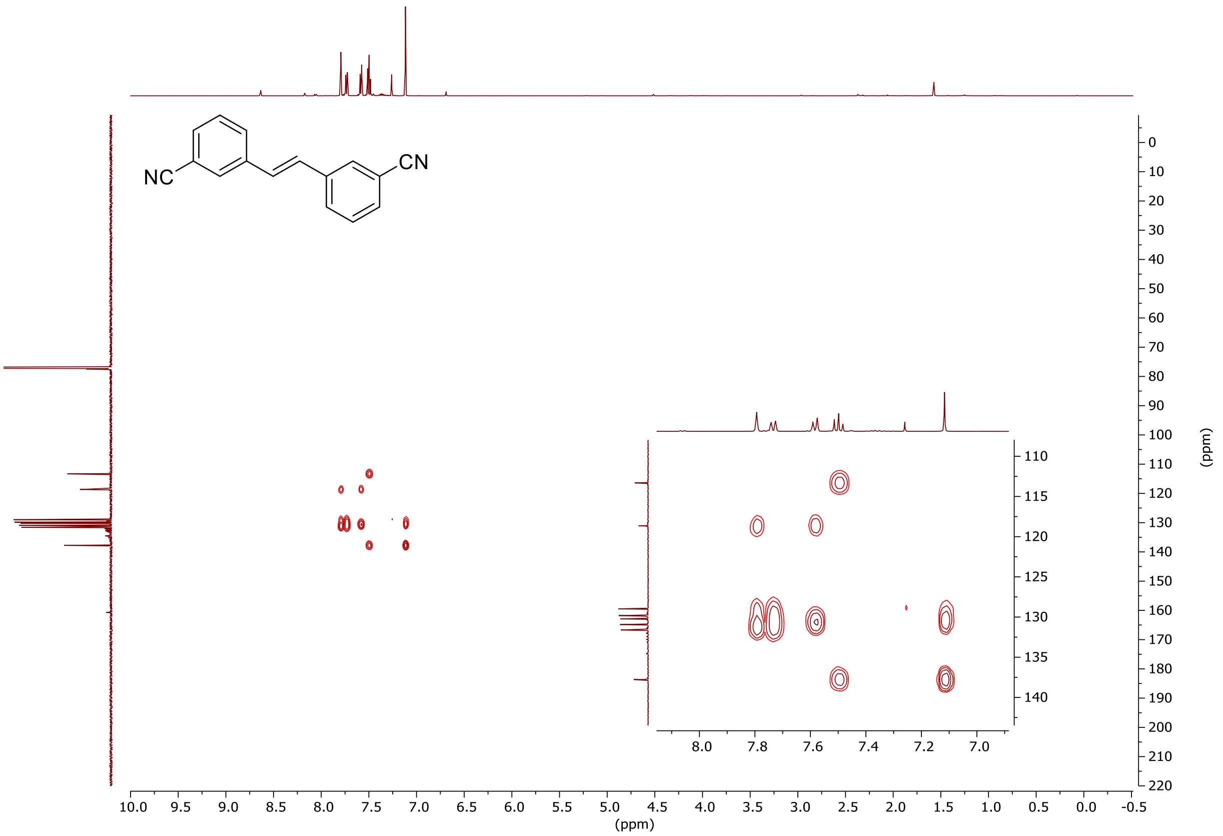

Fig. S373 HMBC (501 MHz, 126 MHz, chloroform-*d*, 298 K) spectrum of compound **14f**

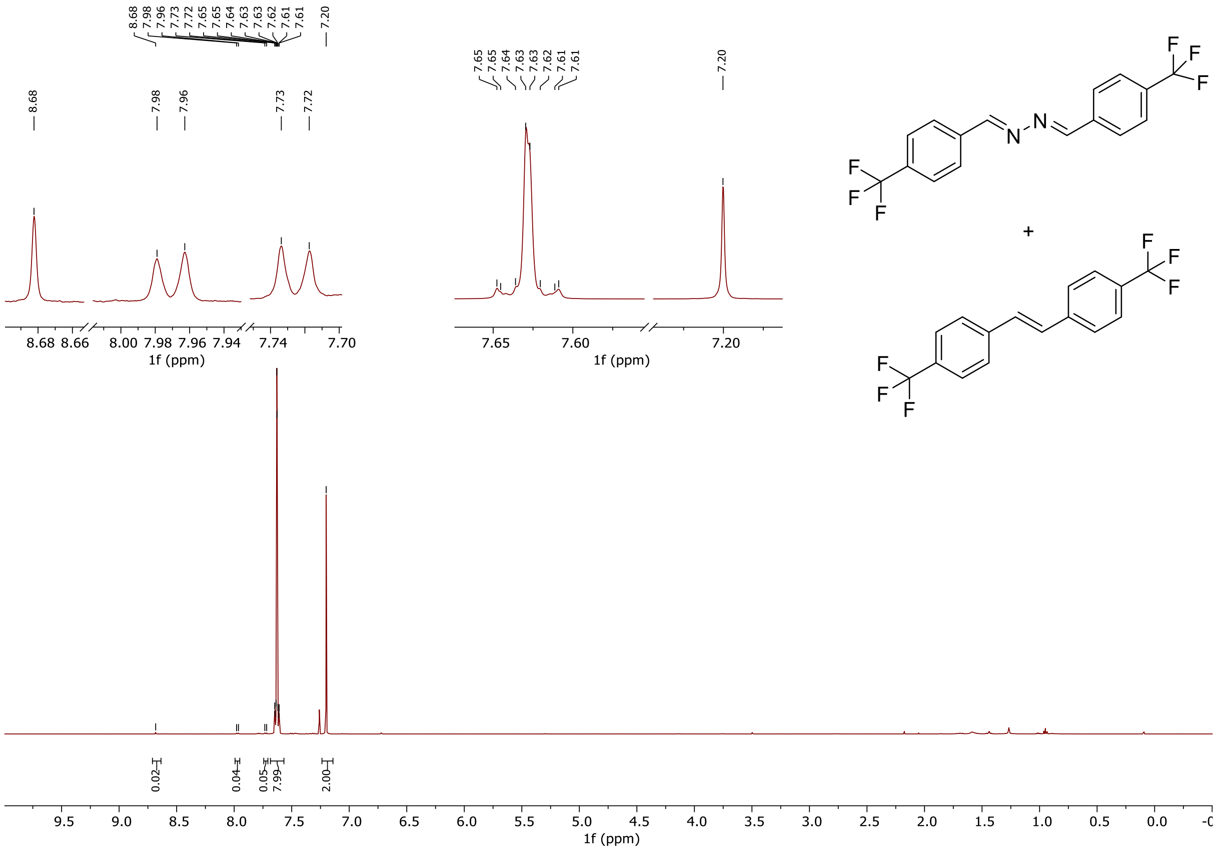

Fig. S374 ^{1}H NMR (501 MHz, chloroform-*d*, 298 K) spectrum of compound **14g** + **13g**

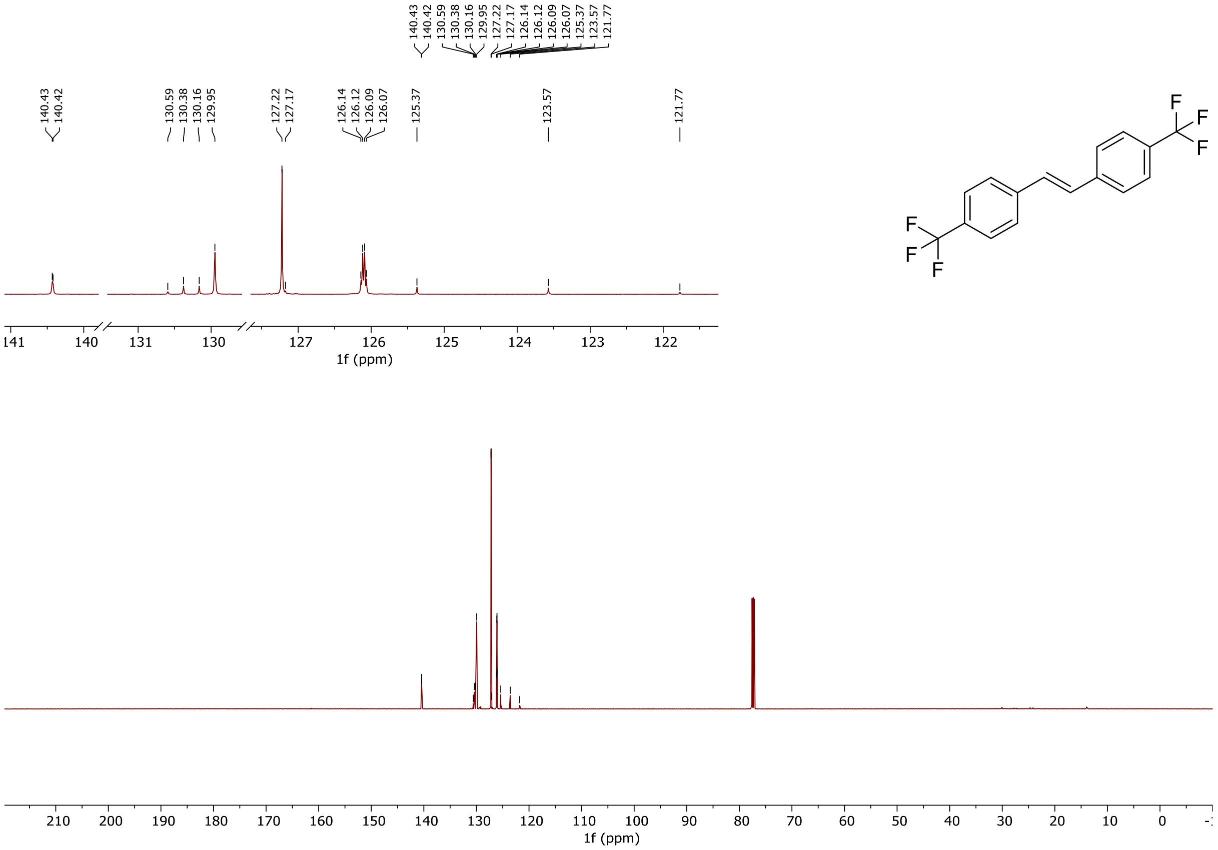

Fig. S375 ^{13}C{^{1}H} NMR (126 MHz, chloroform-*d*, 298 K) spectrum of compound **14g** + **13g**

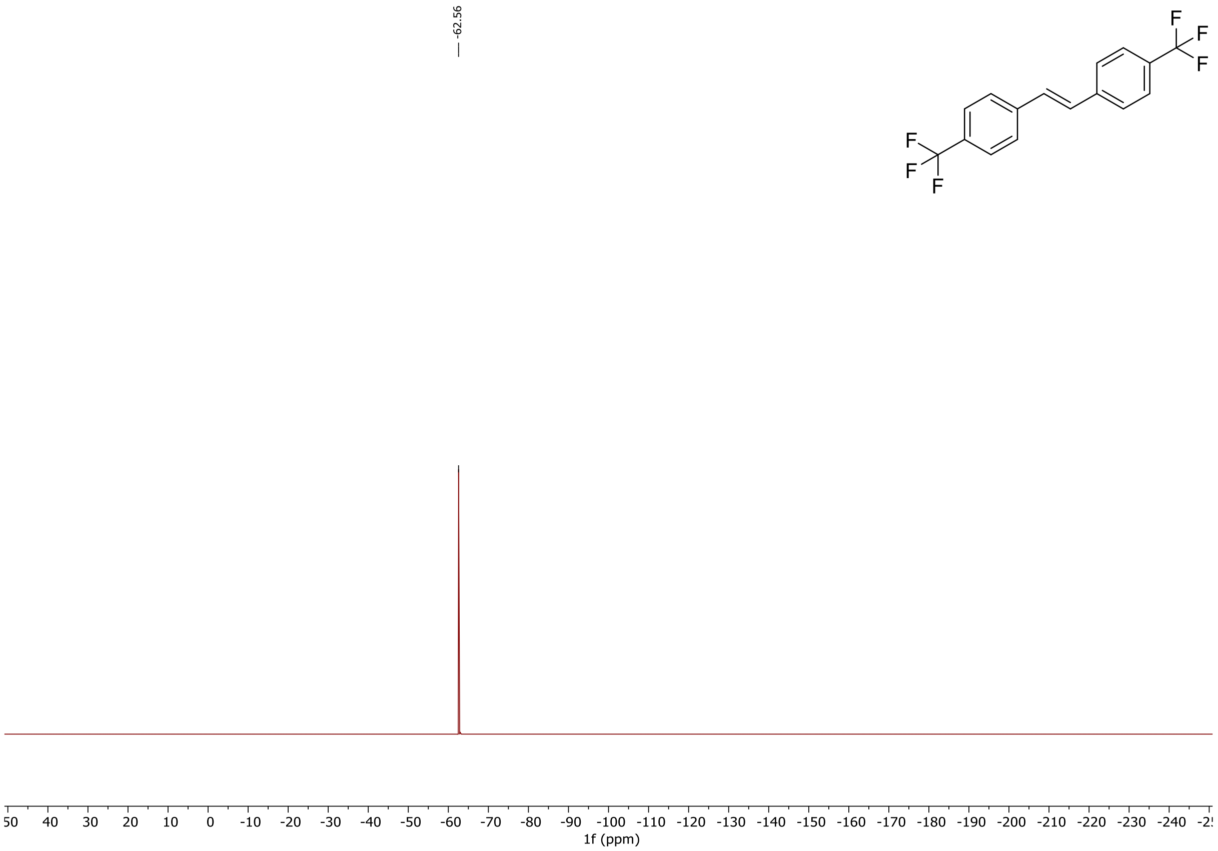

Fig. S376 ^{19}F NMR (565 MHz, chloroform-*d*, 298 K) spectrum of compound **14g**

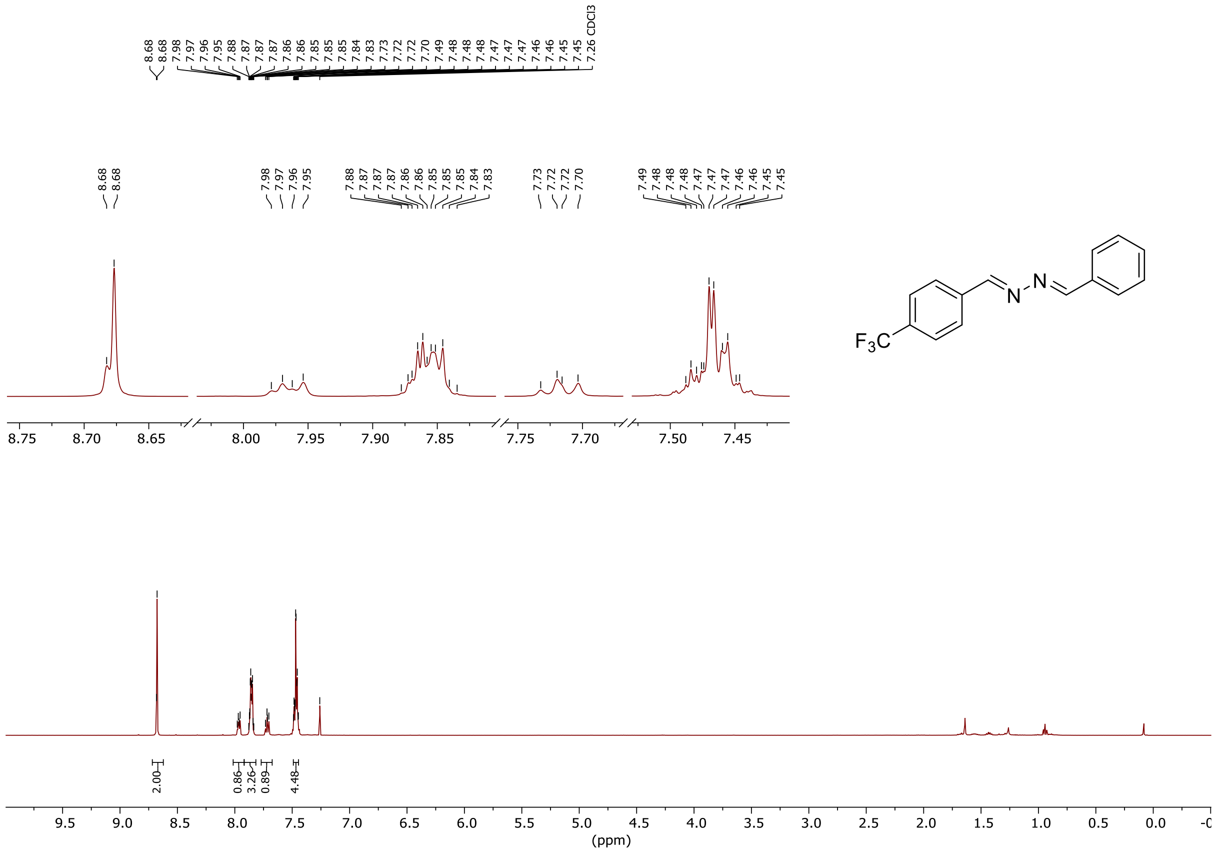

Fig. S377 ^{1}H NMR (501 MHz, chloroform-*d*, 298 K) spectrum of compound **13h**

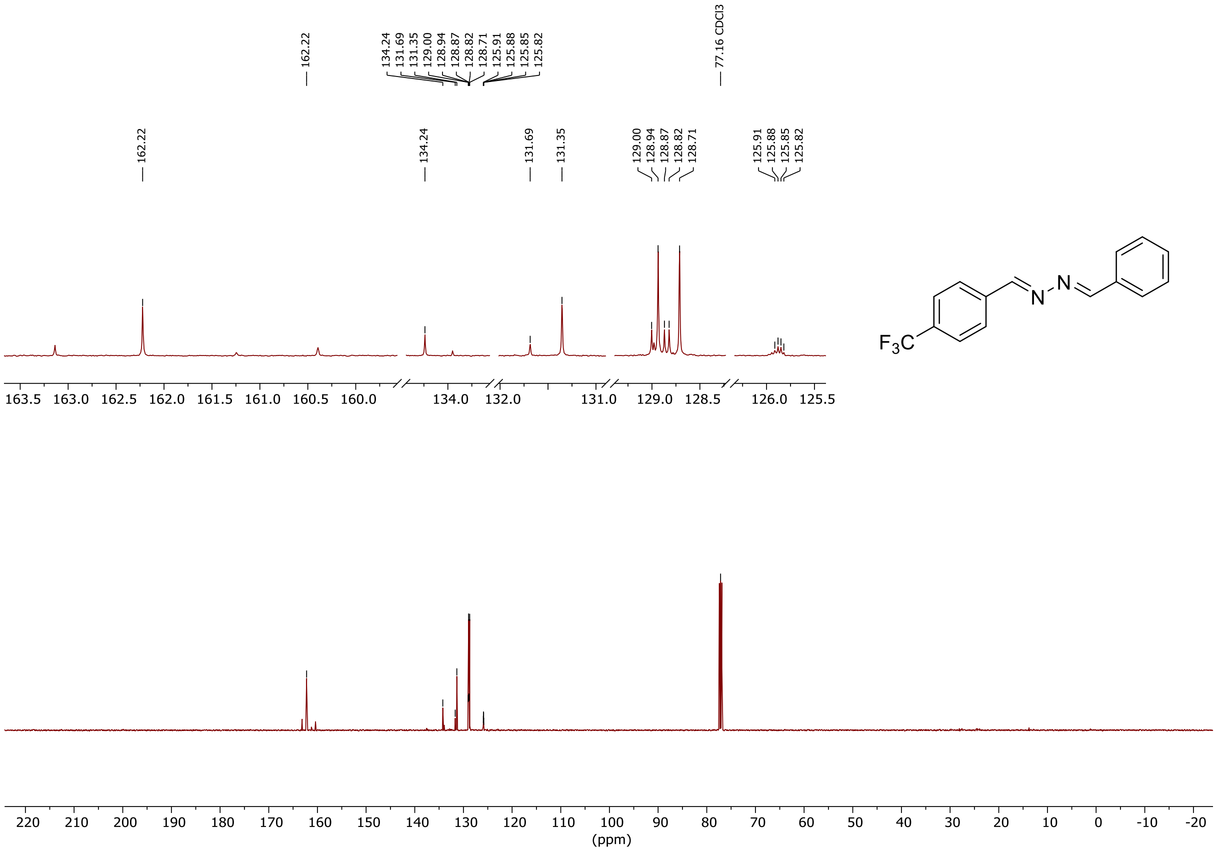

Fig. S378 ^{13}C{^{1}H} NMR (126 MHz, chloroform-*d*, 298 K) spectrum of compound **13h**

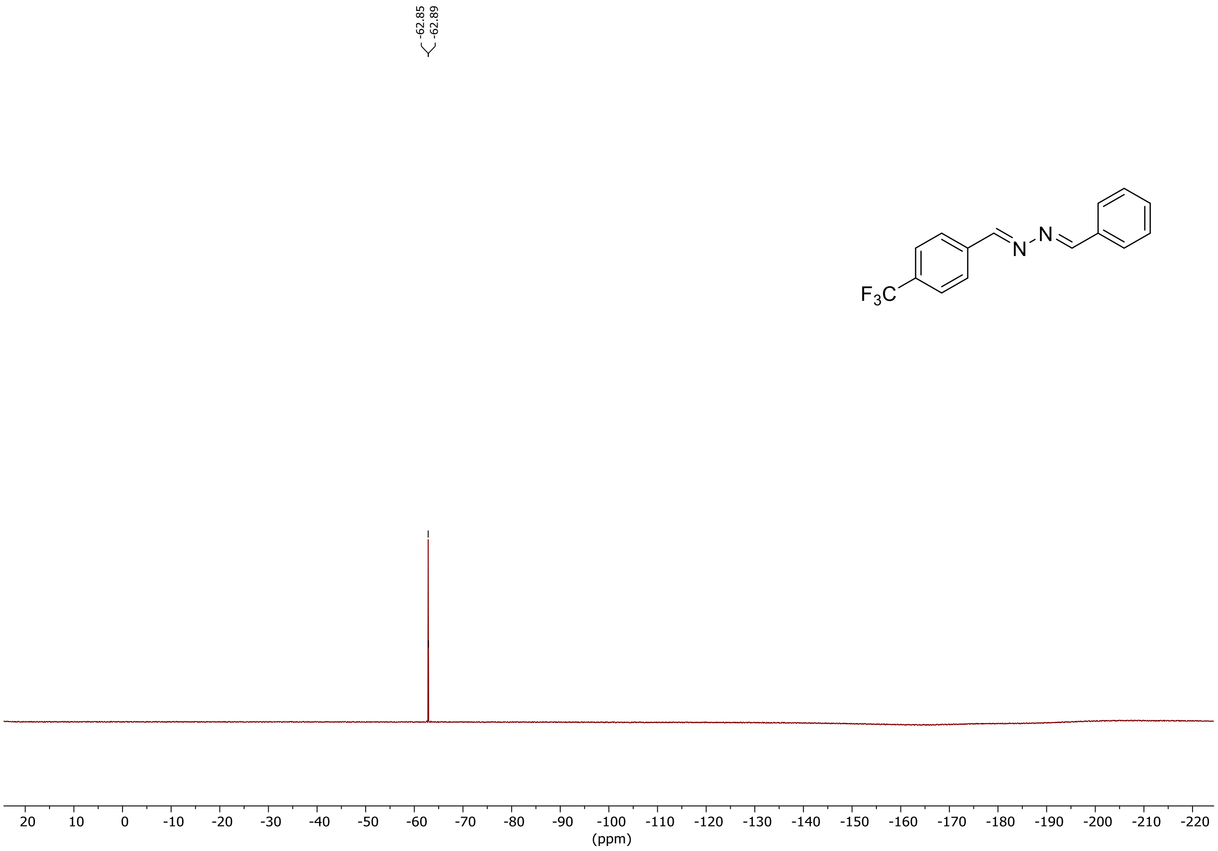

Fig. S379 ^{19}F NMR (565 MHz, chloroform-*d*, 298 K) spectrum of compound **13h**

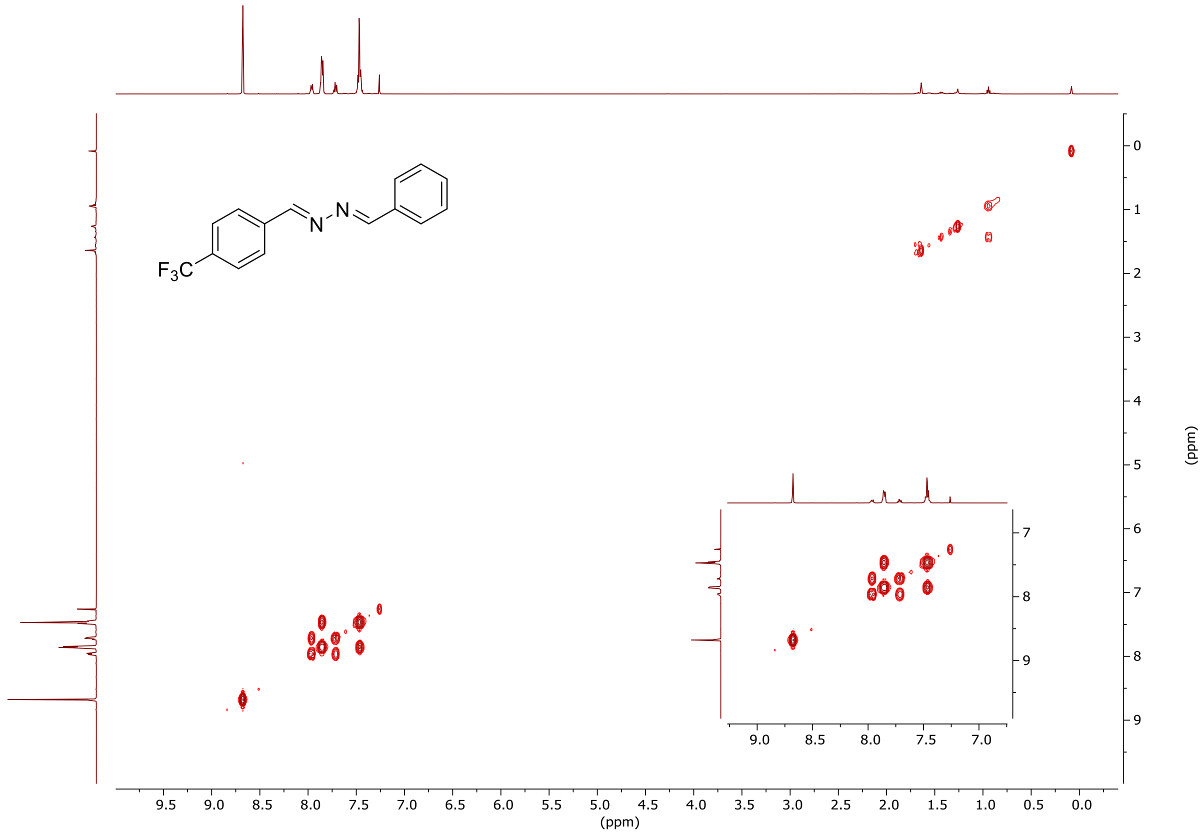

Fig. S380 COSY (501 MHz, 501 MHz, chloroform-*d*, 298 K) spectrum of compound **13h**

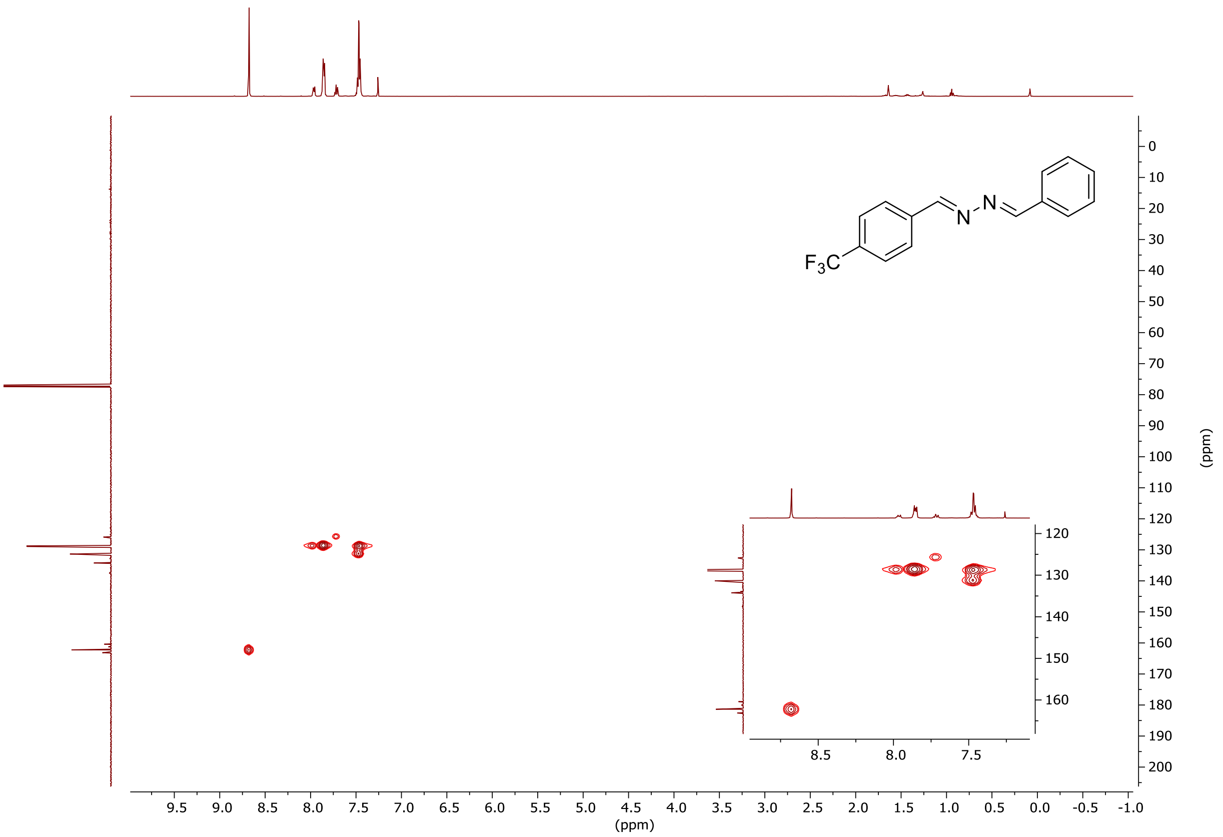

Fig. S381 HSQC (501 MHz, 126 MHz, chloroform-*d*, 298 K) spectrum of compound **13h**

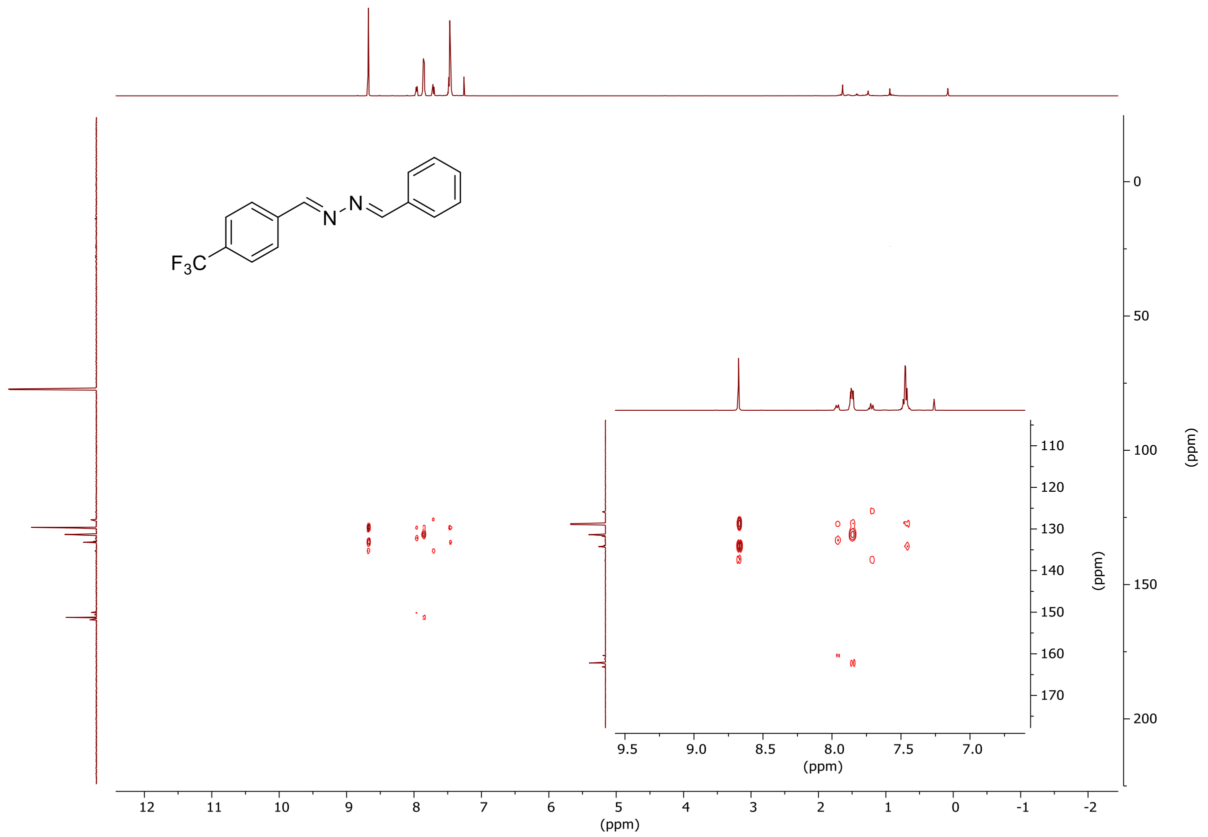

Fig. S382 HMBC (501 MHz, 126 MHz, chloroform-*d*, 298 K) spectrum of compound **13h**

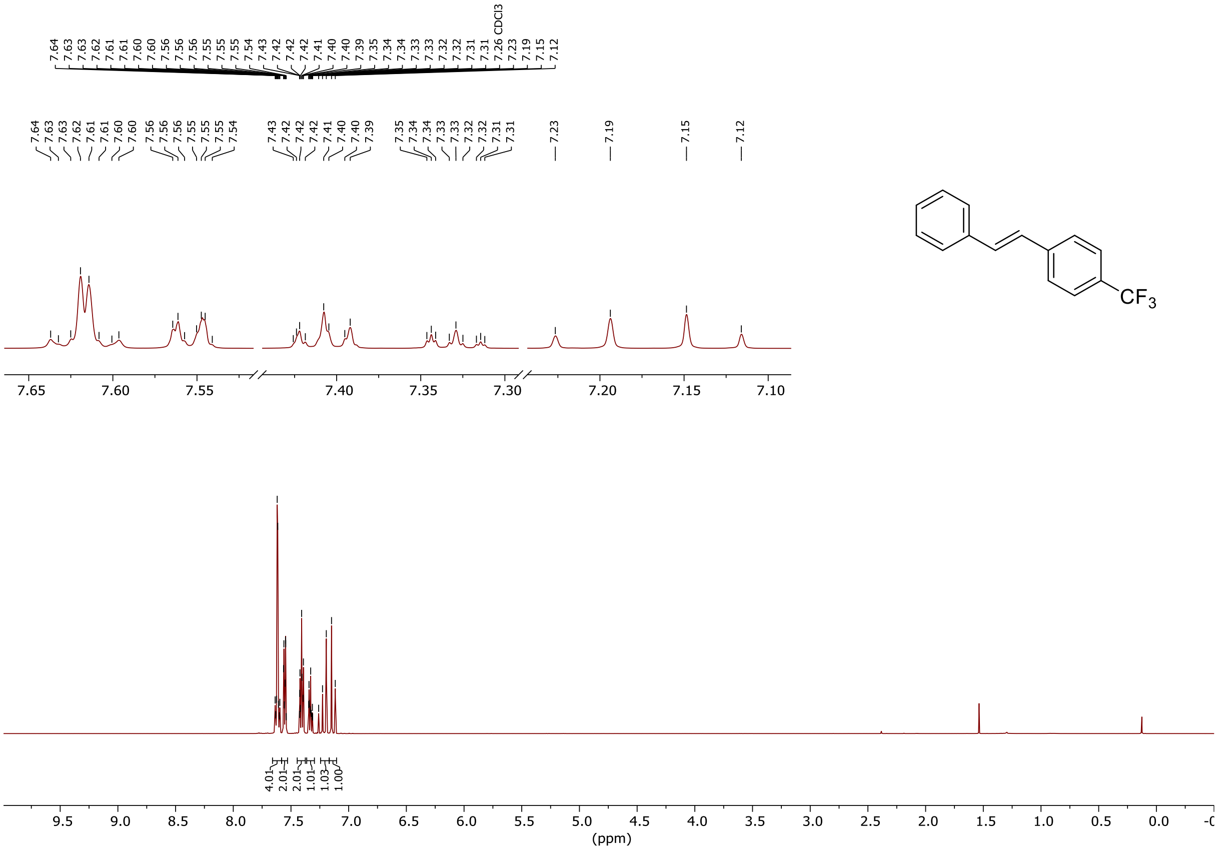

Fig. S383 ^{1}H NMR (501 MHz, chloroform-*d*, 298 K) spectrum of compound **14h**

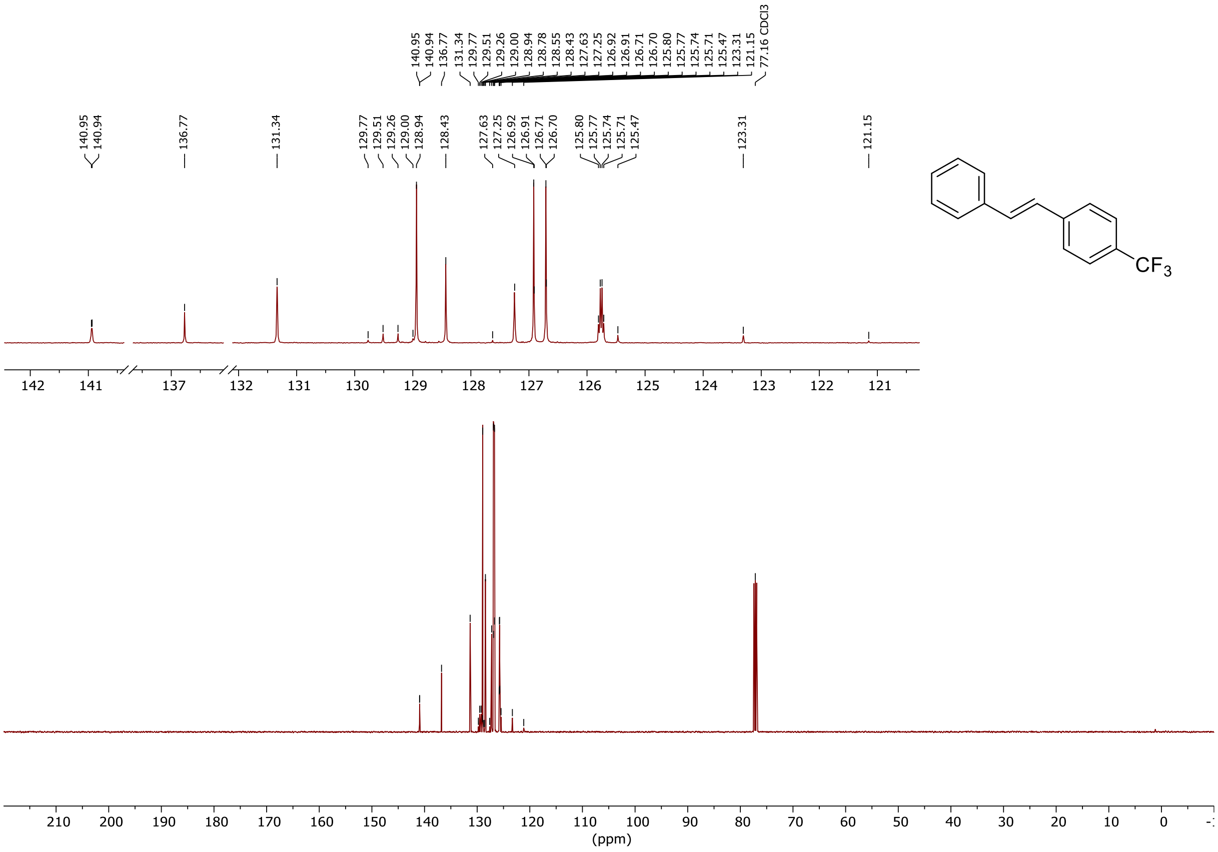

Fig. S384 ^{13}C{1H} NMR (126 MHz, chloroform-*d*, 298 K) spectrum of compound **14h**

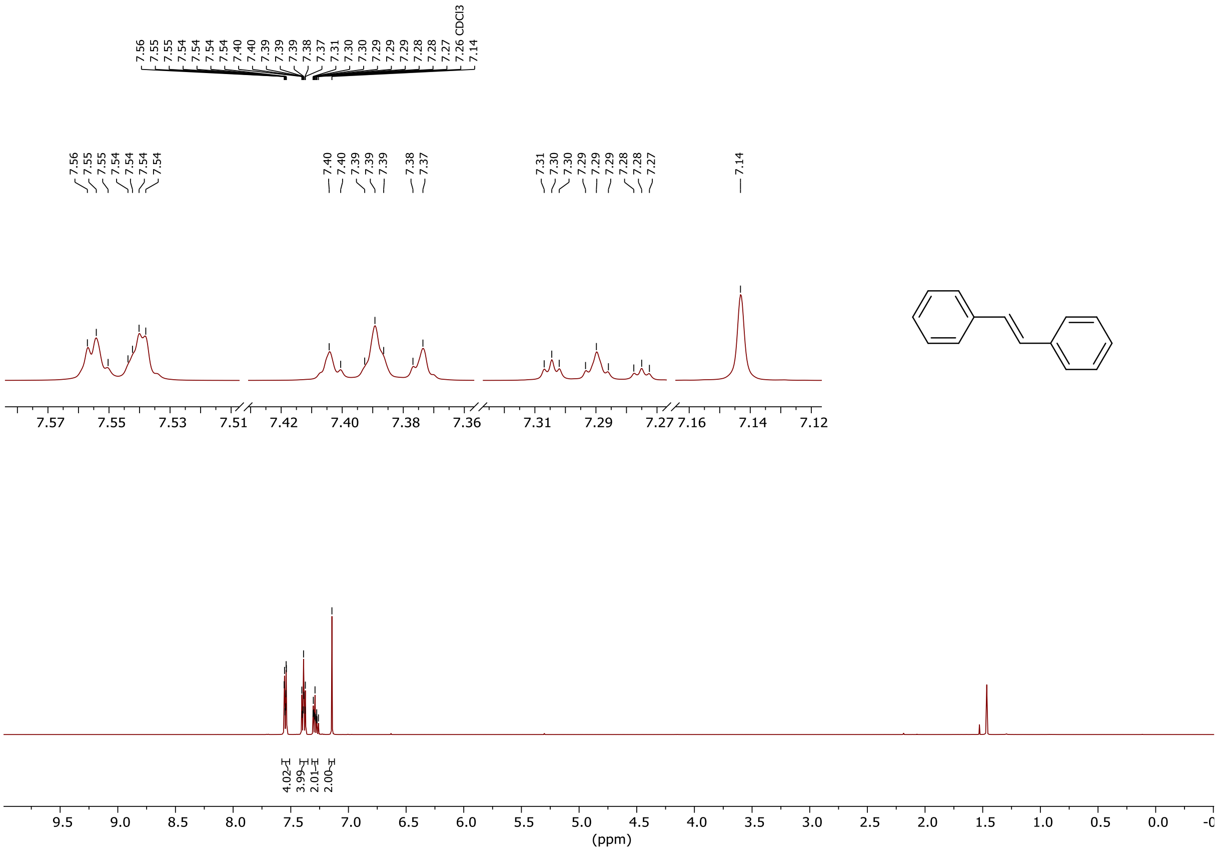

Fig. S385 ^{1}H NMR (501 MHz, chloroform-*d*, 298 K) spectrum of compound **14a**

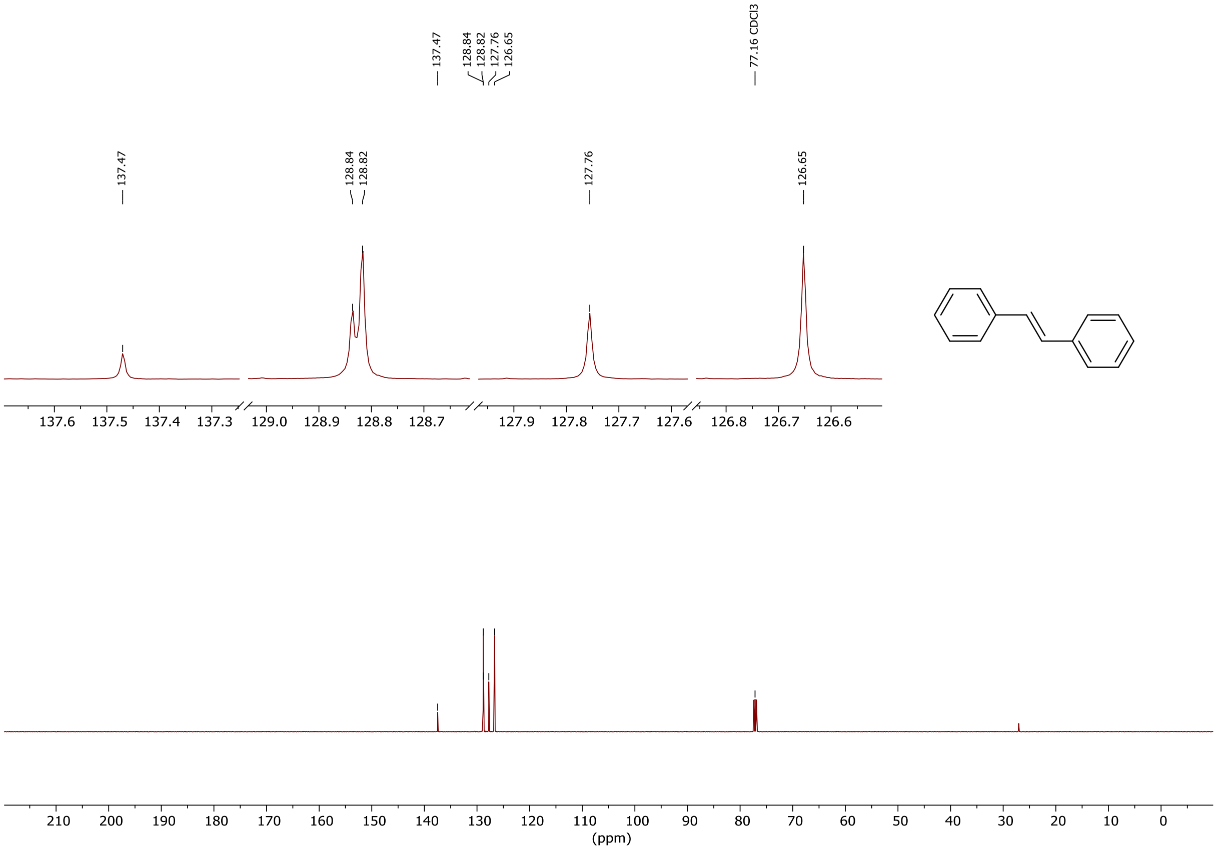

Fig. S386 $^{13}C\{^{1}H\}$ NMR (126 MHz, chloroform-*d*, 298 K) spectrum of compound **14a**

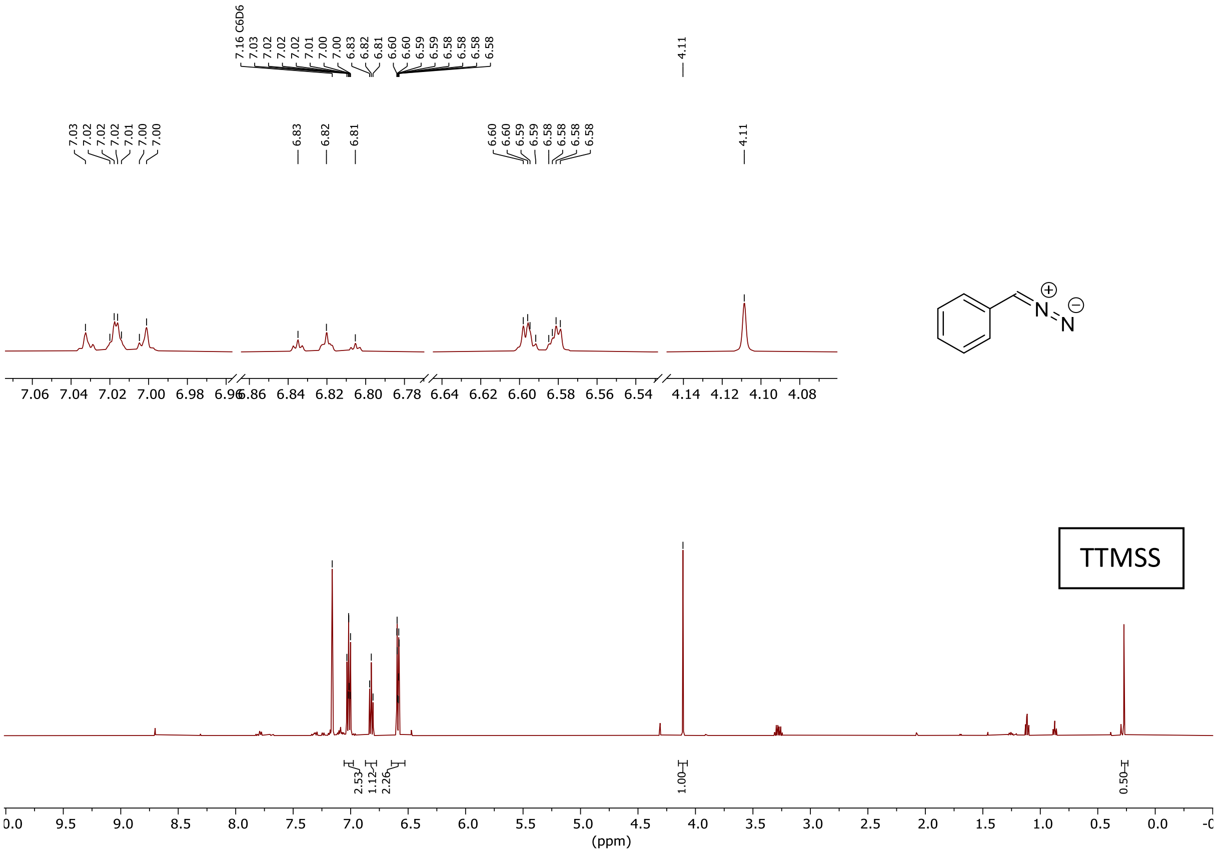

Fig. S387 ^{1}H NMR (501 MHz, benzene-d_6, 298 K) spectrum of compound **15a** with TTMSS as internal standard

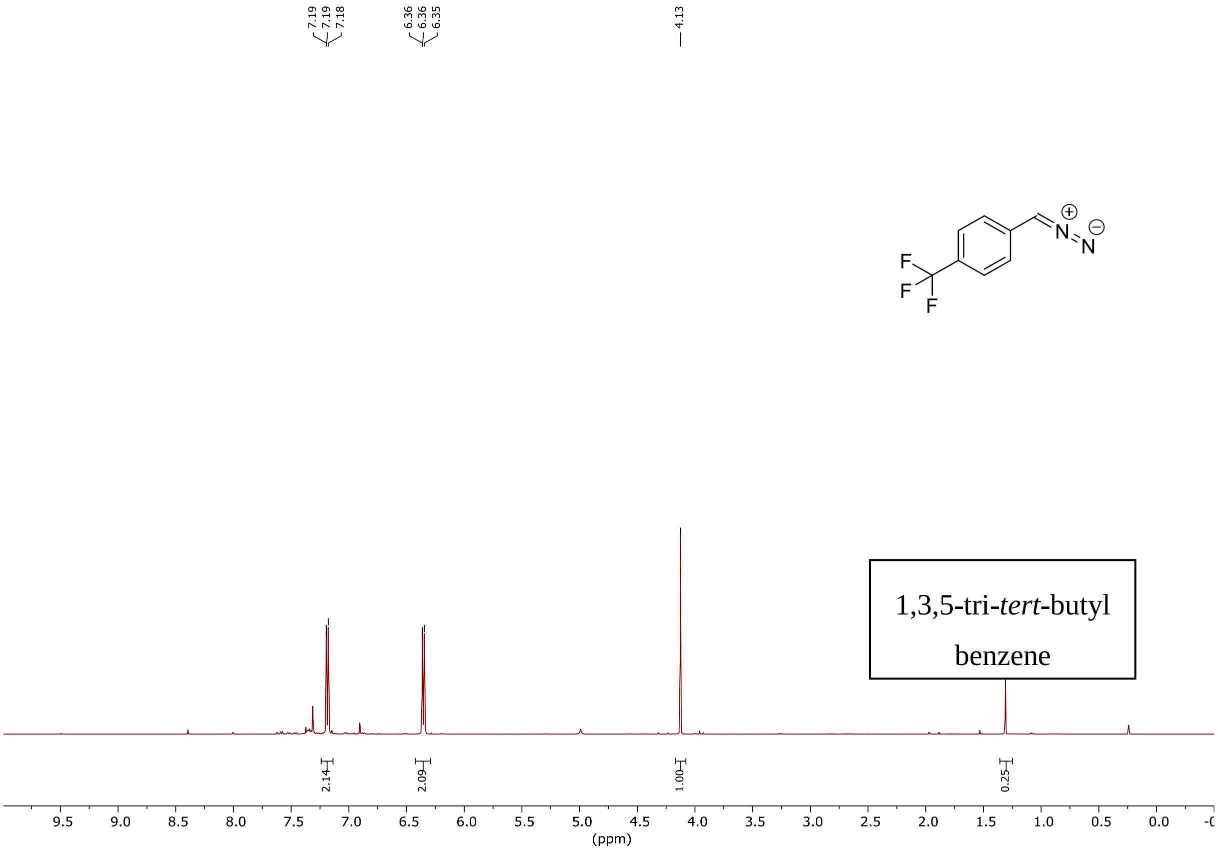

Fig. S388 ^{1}H NMR (501 MHz, chloroform-*d*, 298 K) spectrum of compound **15b** with 1,3,5-tri-*tert*-butyl benzene

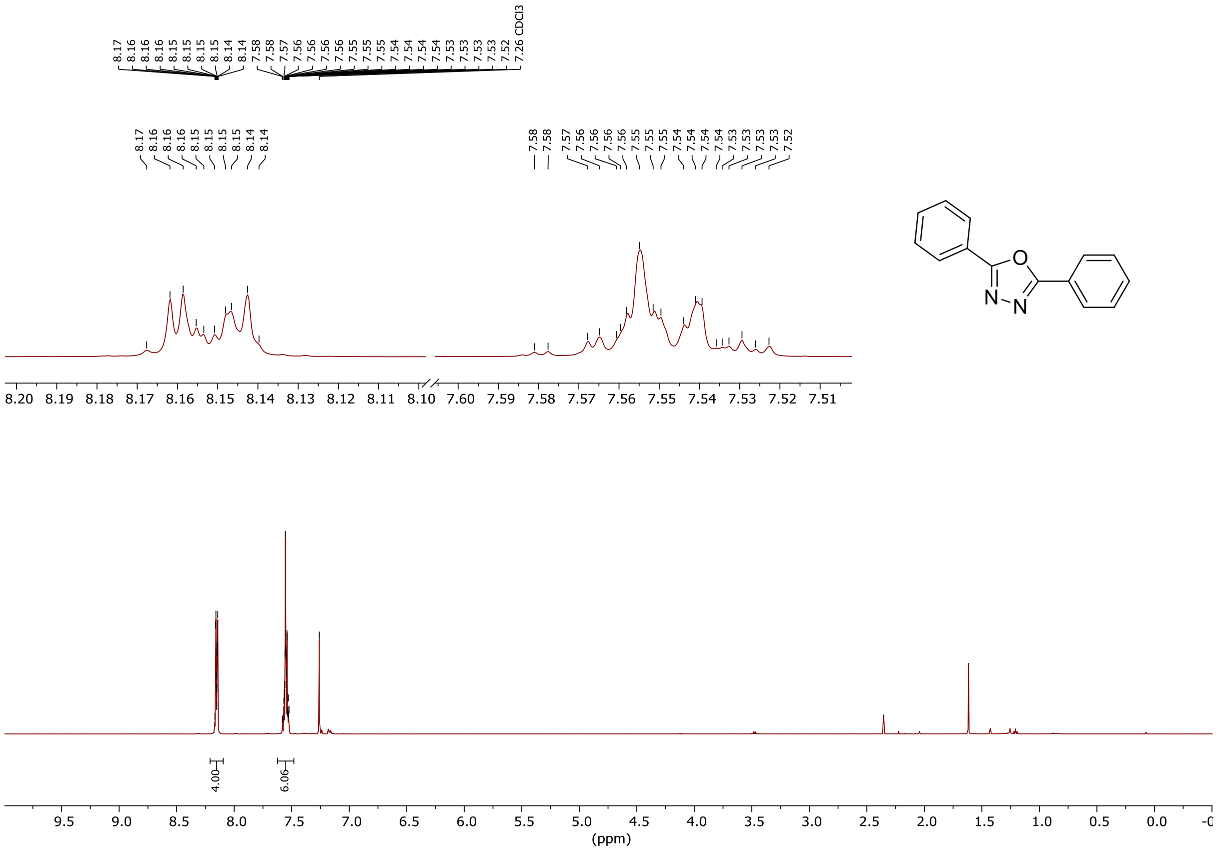

Fig. S389 ^{1}H NMR (501 MHz, chloroform-*d*, 298 K) spectrum of compound **16**

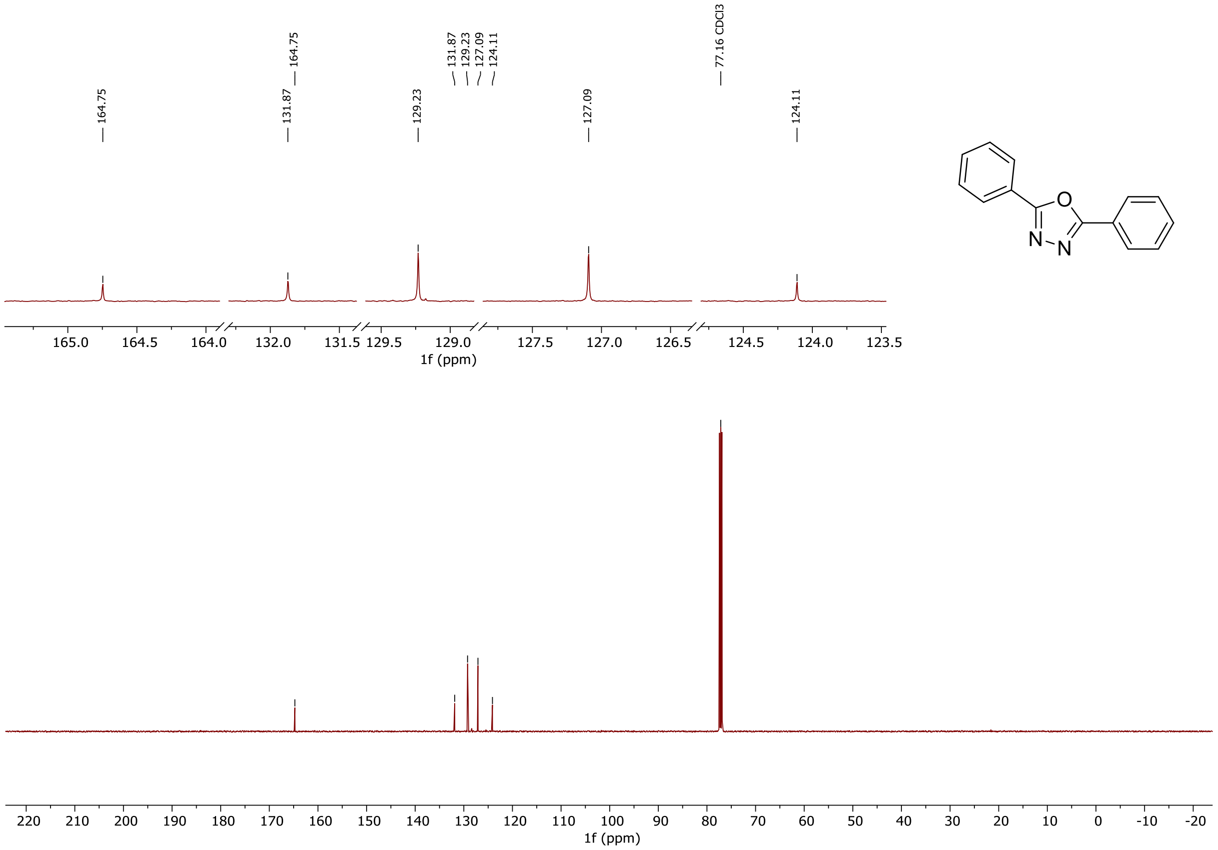

Fig. S390 $^{13}C\{^{1}H\}$ NMR (126 MHz, chloroform-*d*, 298 K) spectrum of compound **16**

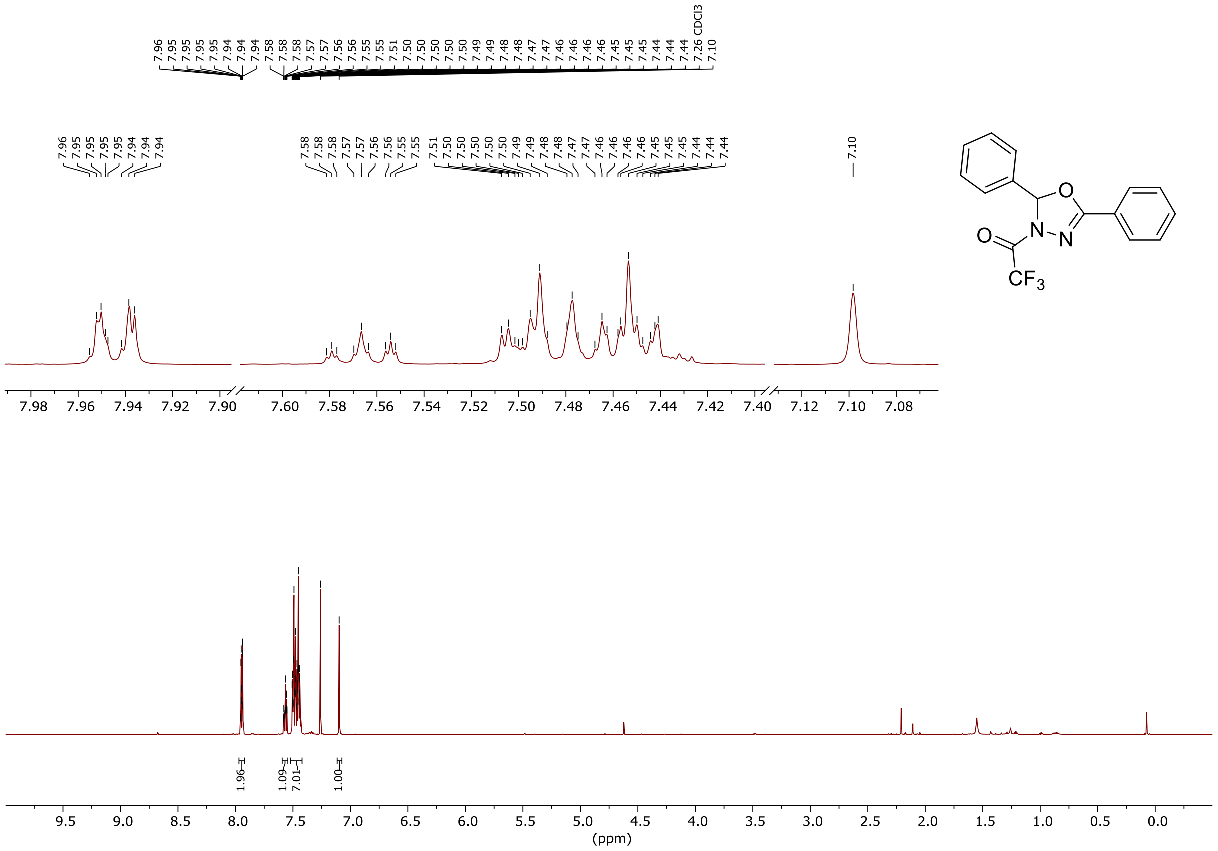

Fig. S391 ^{1}H NMR (501 MHz, chloroform-*d*, 298 K) spectrum of compound **17**

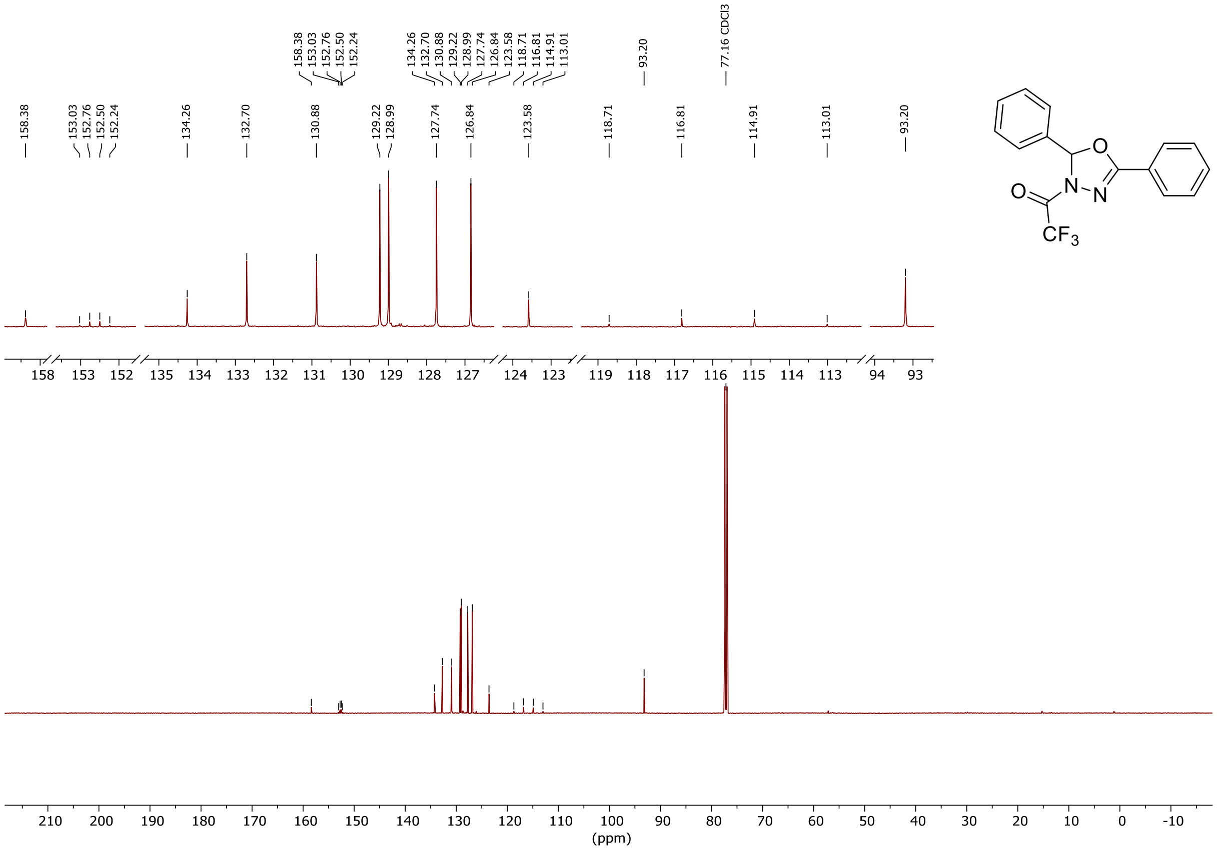

Fig. S392 $^{13}C\{^{1}H\}$ NMR (126 MHz, chloroform-*d*, 298 K) spectrum of compound **17**

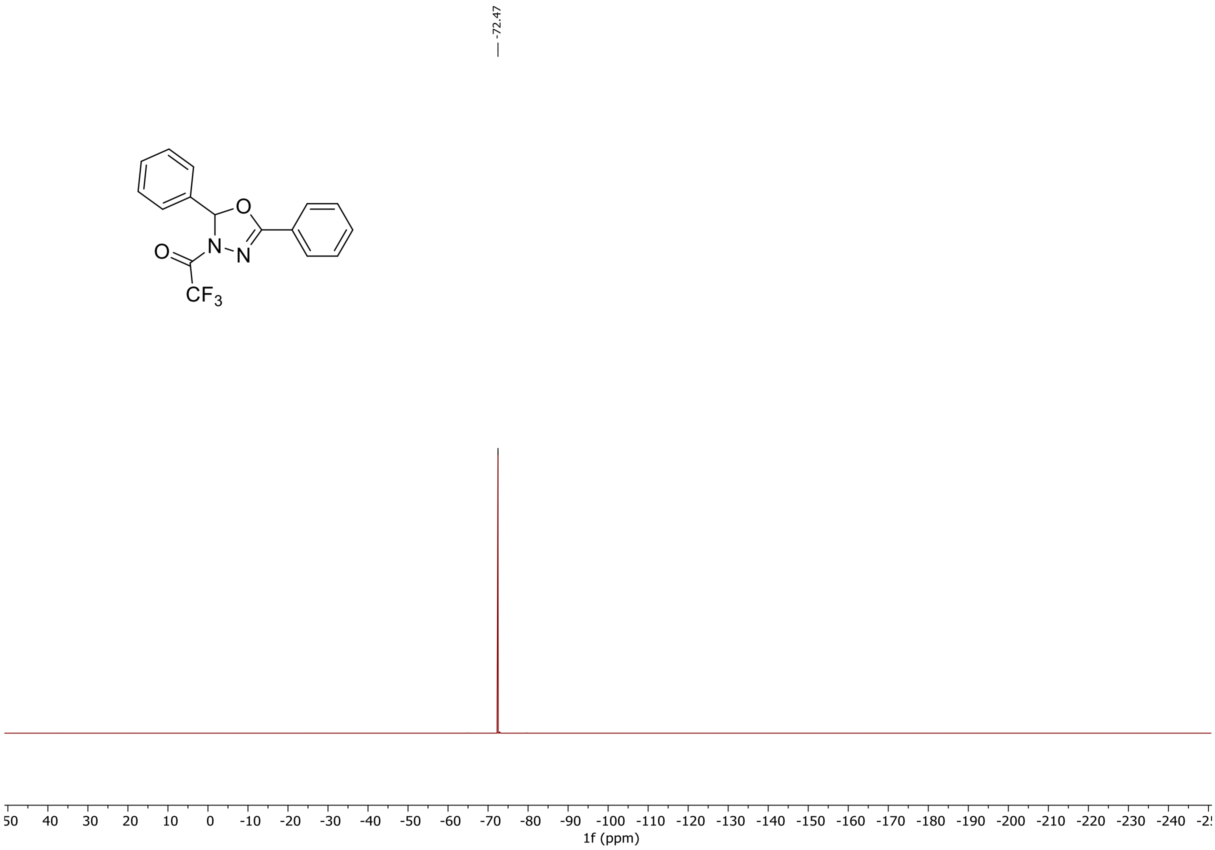

Fig. S393 ^{19}F NMR (565 MHz, chloroform-*d*, 298 K) spectrum of compound **17**

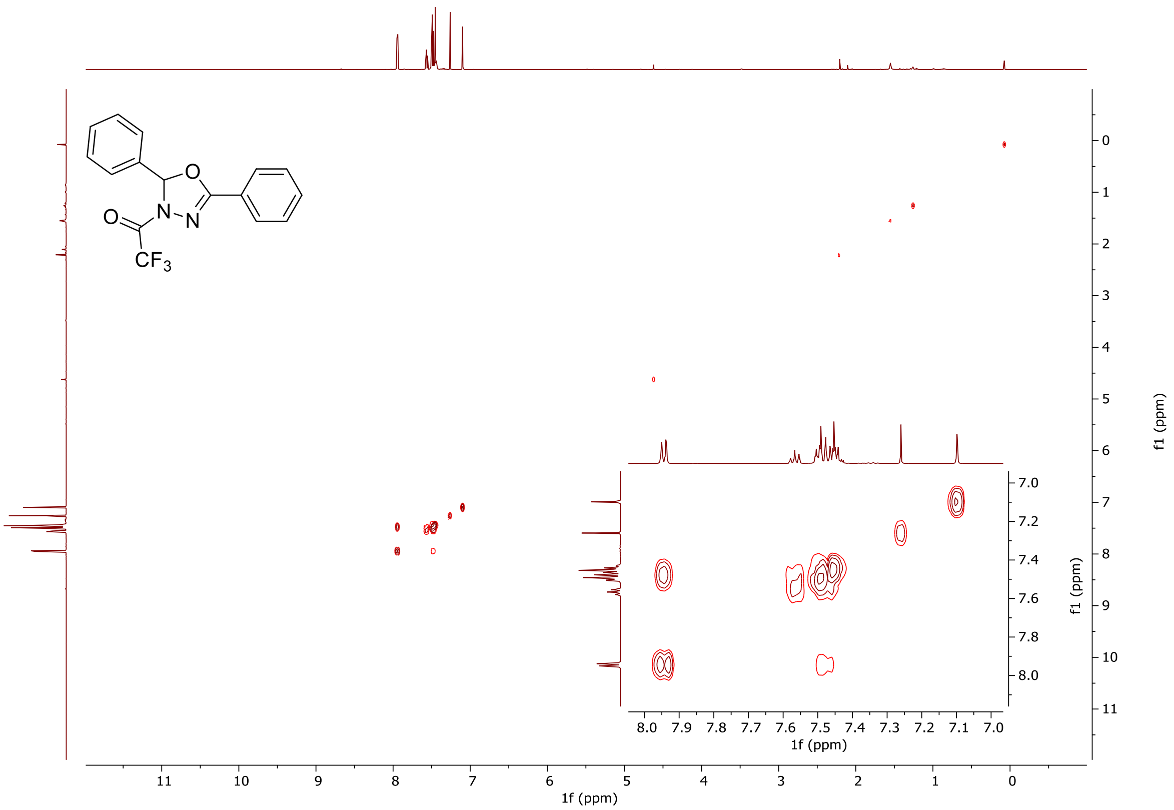

Fig. S394 COSY (501 MHz, 501 MHz, chloroform-*d*, 298 K) spectrum of compound **17**

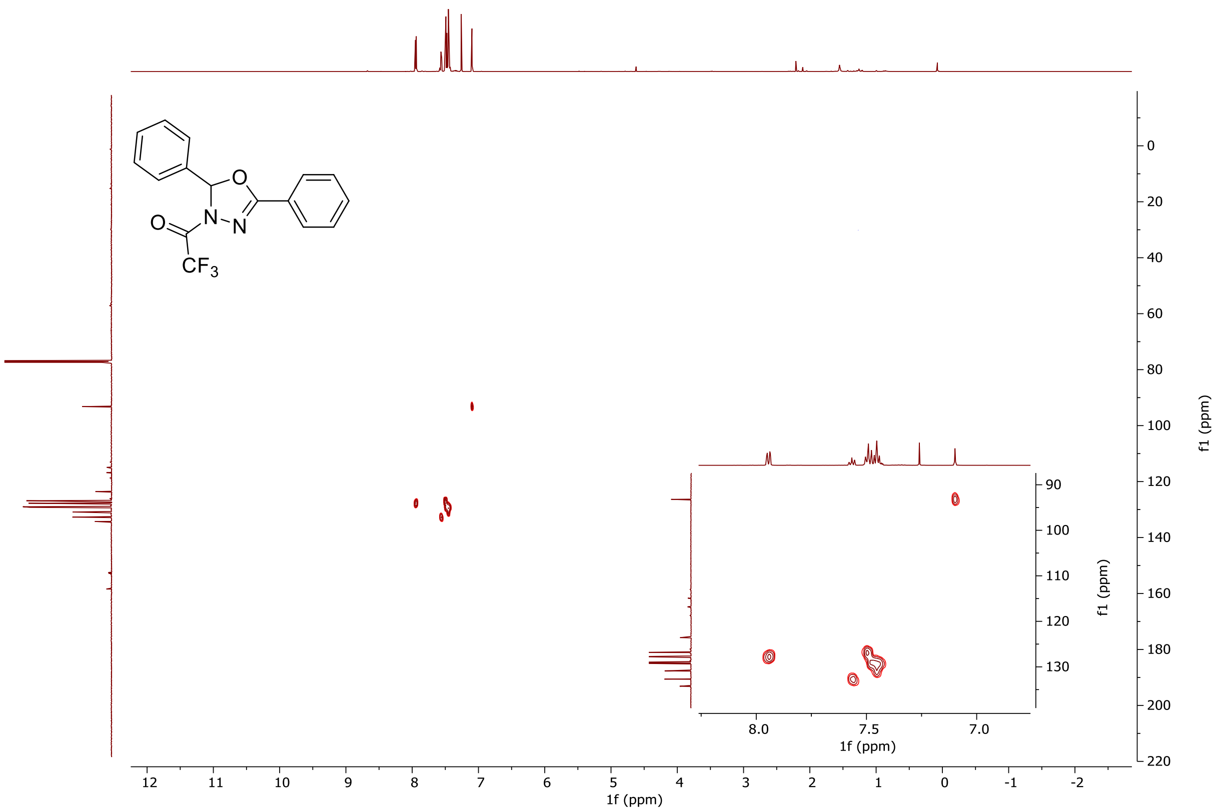

Fig. S395 HSQC (501 MHz, 126 MHz, chloroform-*d*, 298 K) spectrum of compound **17**

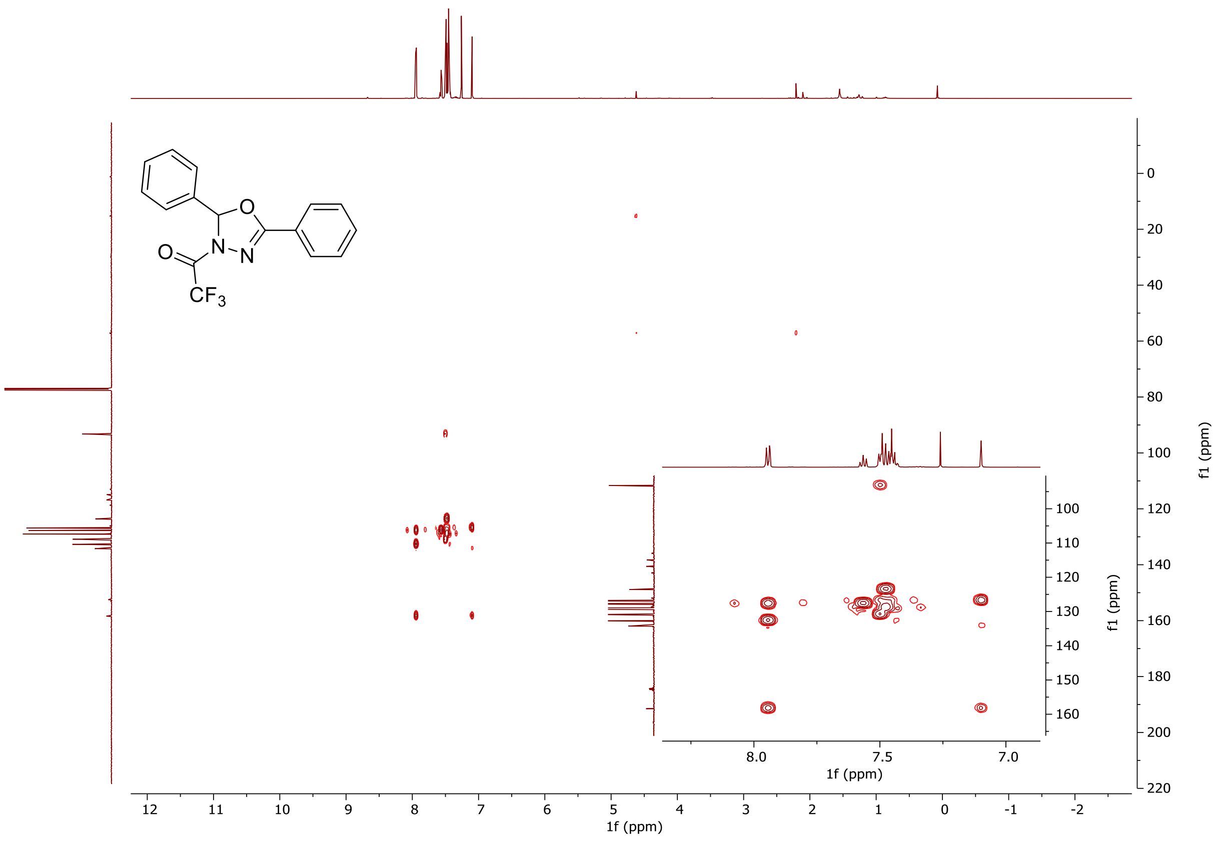

Fig. S396 HMBC (501 MHz, 126 MHz, chloroform-*d*, 298 K) spectrum of compound **17**

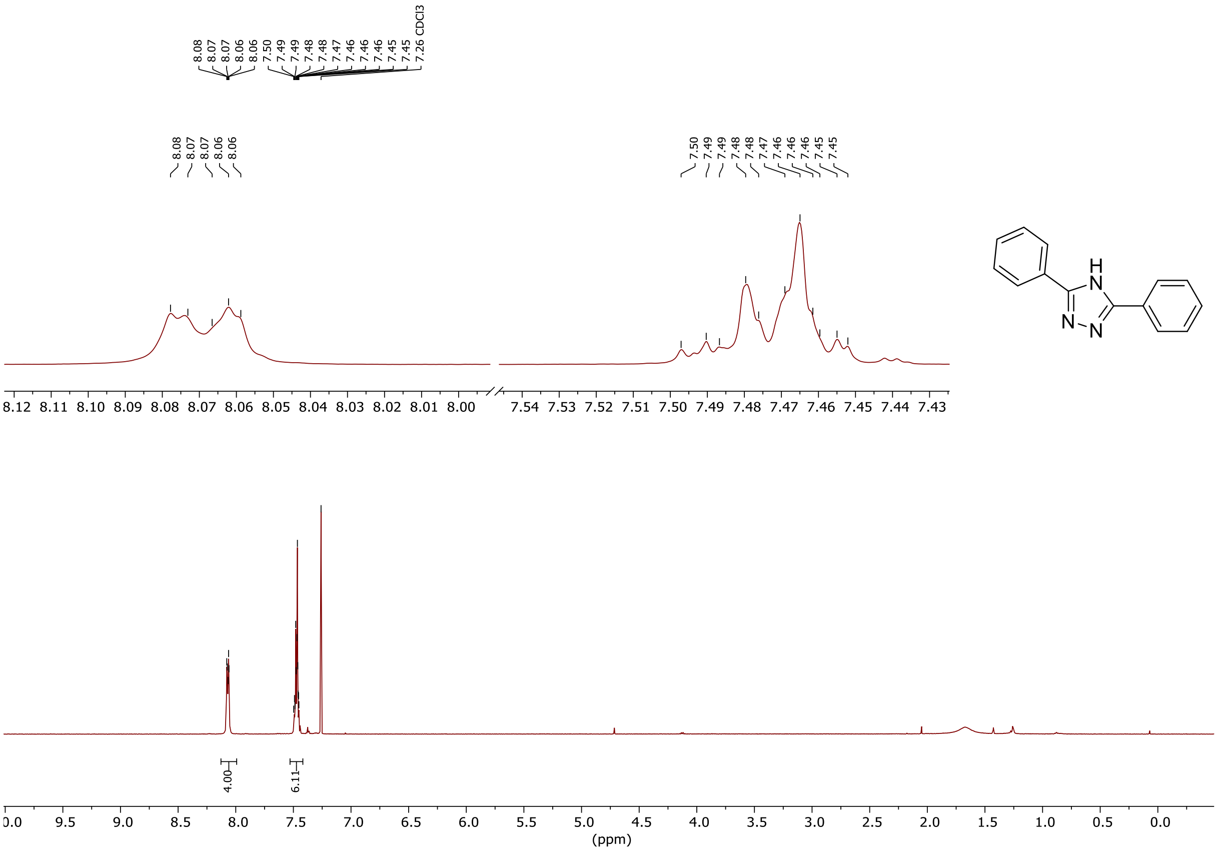

Fig. S397 ^{1}H NMR (501 MHz, chloroform-*d*, 298 K) spectrum of compound **18**

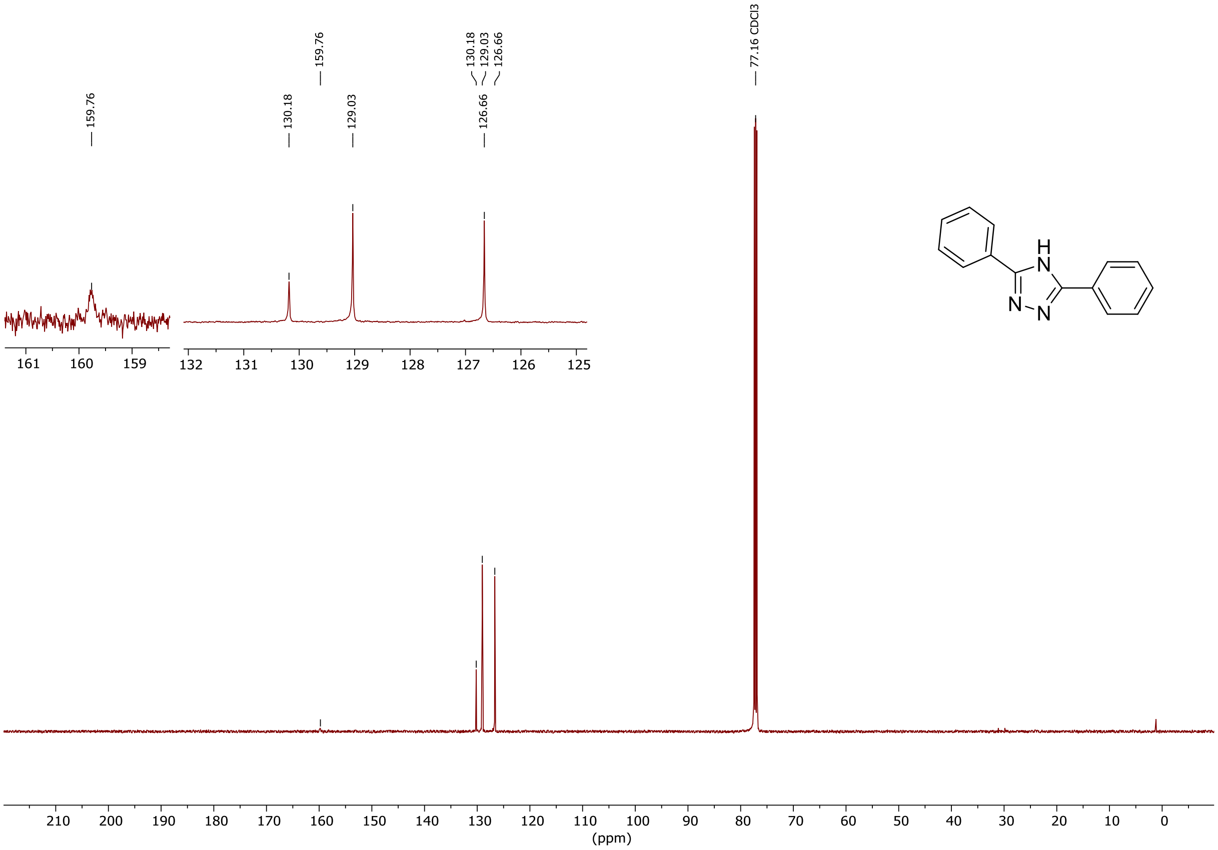

Fig. S398 ^{13}C{^{1}H} NMR (126 MHz, chloroform-*d*, 298 K) spectrum of compound **18**

8. References

[1] S. Adhikari, S. Mandal, A. Ghosh, P. Das, D. Das *J. Org. Chem.* **2015**, *80*, 8530–8538.
[2] J. C. Lukesh, III; K. K. Wallin; R. T. Raines, *Chem. Commun.* **2014**, *50*, 9591–9594.
[3] P. Alam, N. L. C. Leung, H. Su, Z. Qiu, R. T. K. Kwok, J. W. Y. Lam, B. Z. Tang *Chem. – Eur. J.* **2017**, *23*, 14911–14917.
[4] F. Diederich, U. Jonas, V. Gramlich, A. Herrmann, H. Ringsdorf, C. Thilgen *Helv. Chim. Acta.* **1993**, *76*, 2445–2453.
[5] I. Čorić, J. H. Kim, T. Vlaar, M. Patil, W. Thiel, B. List *Angew. Chem. Int. Ed.* **2013**, *52*, 3490–3493.
[6] C. Fieldhouse, A. Glen, T. Fujimoto, J. S. Robinson, *Substituted Cyclopentanes, Tetrahydrofuranes and Pyrrolidines as Orexin Receptor Antagonists*, **2015**, WO2015124934A1.
[7] T. Drennhaus, D. Leifert, J. Lammert, J. P. Drennhaus, K. Bergander, C. G. Daniliuc, A. Studer *J. Am. Chem. Soc.* **2023**, *145*, 8665–8676.
[8] S. Nomura, K. Endo-Umeda, M. Makishima, Y. Hashimoto, M. Ishikawa *ChemMedChem* **2016**, *11*, 2347–2360.
[9] J. J. Kiddle *Tetrahedron Lett.* **2000**, *41*, 1339–1341.
[10] R. V. H. Jones, W. E. Lindsell, G. C. Paddon-Jones, D. D. Palmer, P. N. Preston, G. M. Rosair, A. J. Whitton *J. Organomet. Chem.* **2006**, *691*, 2378–2385.
[11] P. Das, M. K. Kesharwani, A. K. Mandal, E. Suresh, B. Ganguly, A. Das *Org. Biomol. Chem.* **2012**, *10*, 2263.
[12] J. Cvengros, S. Toma, S. Marque, A. Loupy *Can. J. Chem.* **2004**, *82*, 1365–1371.
[13] T. Hirayama, S. Ueda, T. Okada, N. Tsurue, K. Okuda, H. Nagasawa *Chem. – Eur. J.* **2014**, *20*, 4156–4162.
[14] S. Pankajakshan, Z. Guang Chng, R. Ganguly, T. Peng Loh *Chem. Commun.* **2015**, *51*, 5929–5931.
[15] K. Iizumi, K. P. Nakayama, K. Kato, K. Muto, J. Yamaguchi *J. Org. Chem.* **2022**, *87*, 11909–11918.
[16] S. Liu, J. Sawicki, T. G. Driver *Org. Lett.* **2012**, *14*, 3744–3747.
[17] Z.-H. Shang, Z.-X. Zhang, W.-Z. Weng, Y.-F. Wang, T.-W. Cheng, Q.-Y. Zhang, L.-Q. Song, T.-Q. Shao, K.-X. Liu, Y.-P. Zhu *Adv. Synth. Catal.* **2021**, *363*, 490–496.
[18] O. J. Edirin, J. D. Carrick *J. Org. Chem.* **2024**, *89*, 7201–7209.
[19] G. Bentabed-Ababsa, F. Blanco, A. Derdour, F. Mongin, F. Trécourt, G. Quéguiner, R. Ballesteros, B. Abarca *J. Org. Chem.* **2009**, *74*, 163–169.
[20] N. R. Patel, R. N. Castle *J. Heterocycl. Chem.* **1966**, *3*, 512–517.
[21] J. H. An, K. D. Kim, J. H. Lee *J. Org. Chem.* **2021**, *86*, 2876–2894.
[22] T. Mi, R. Li, *Synthesis Method of 6-Bromophthalazine*, **2020**, CN111925331A.
[23] R. A. Bunce, T. Harrison, B. Nammalwar *Heterocycl. Commun.* **2012**, *18*, 123–126.
[24] S. N. Kessler, H. A. Wegner *Org. Lett.* **2012**, *14*, 3268–3271.
[25] Y. E. Türkmen, T. J. Montavon, S. A. Kozmin, V. H. Rawal *J. Am. Chem. Soc.* **2012**, *134*, 9062–9065.
[26] M. S. Ledovskaya, M. V. Polynski, V. P. Ananikov *Chem. – Asian J.* **2021**, *16*, 2286–2297.
[27] L. Hu, C. Mück-Lichtenfeld, T. Wang, G. He, M. Gao, J. Zhao *Chem. – Eur. J.* **2016**, *22*, 911–915.
[28] G. Ganesan, P. Balasubramaniam, M. Yadav, H. Janwadkar, A. Papalkar, A. Chaskar *Synlett* **2025**, *36*, 1927–1931.
[29] P. Seboletswe, G. Kumar, N. Gcabashe, K. Olofinsan, S. Islam, Al. Idris, P. Singh *Chem. Biodivers.* **2025**, *22*, e202401556.
[30] F. Zhao, J. Luo, Q. Tan, Y. Liao, S. Peng, G.-J. Deng *Adv. Synth. Catal.* **2012**, *354*, 1914–1918.

[31]J. Ariai, J. Becker, U. Gellrich *Eur. J. Org. Chem.* **2024**, *27*, e202301252.
[32]Z. Shang, J. Reiner, J. Chang, K. Zhao *Tetrahedron Lett.* **2005**, *46*, 2701–2704.
[33]X. Meng, C. Yu, P. Zhao *RSC Adv.* **2014**, *4*, 8612–8616.
[34]G. M. Sheldrick *Acta Crystallogr. Sect. C Struct. Chem.* **2015**, *71*, 3–8.
[35]O. V. Dolomanov, L. J. Bourhis, R. J. Gildea, J. A. K. Howard, H. Puschmann *J. Appl. Crystallogr.* **2009**, *42*, 339–341.
[36]S. W. Youn, B. S. Kim, A. R. Jagdale *J. Am. Chem. Soc.* **2012**, *134*, 11308–11311.
[37]Gaussian 16, Revision C.01, M. J. Frisch, G. W. Trucks, H. B. Schlegel, G. E. Scuseria, M. A. Robb, J. R. Cheeseman, G. Scalmani, V. Barone, G. A. Petersson, H. Nakatsuji, X. Li, M. Caricato, A. V. Marenich, J. Bloino, B. G. Janesko, R. Gomperts, B. Mennucci, H. P. Hratchian, J. V. Ortiz, A. F. Izmaylov, J. L. Sonnenberg, Williams, F. Ding, F. Lipparini, F. Egidi, J. Goings, B. Peng, A. Petrone, T. Henderson, D. Ranasinghe, V. G. Zakrzewski, J. Gao, N. Rega, G. Zheng, W. Liang, M. Hada, M. Ehara, K. Toyota, R. Fukuda, J. Hasegawa, M. Ishida, T. Nakajima, Y. Honda, O. Kitao, H. Nakai, T. Vreven, K. Throssell, J. A. Montgomery Jr., J. E. Peralta, F. Ogliaro, M. J. Bearpark, J. J. Heyd, E. N. Brothers, K. N. Kudin, V. N. Staroverov, T. A. Keith, R. Kobayashi, J. Normand, K. Raghavachari, A. P. Rendell, J. C. Burant, S. S. Iyengar, J. Tomasi, M. Cossi, J. M. Millam, M. Klene, C. Adamo, R. Cammi, J. W. Ochterski, R. L. Martin, K. Morokuma, O. Farkas, J. B. Foresman, D. J. Fox **2016**.
[38]F. Weigend, R. Ahlrichs *Phys. Chem. Chem. Phys.* **2005**, *7*, 3297.
[39]L. Goerigk, S. Grimme *J. Chem. Theory Comput.* **2011**, *7*, 291–309.
[40]S. Grimme, S. Ehrlich, L. Goerigk *J. Comput. Chem.* **2011**, *32*, 1456–1465.
[41]E. R. Johnson, A. D. Becke *J. Chem. Phys.* **2005**, *123*, 024101.
[42]E. R. Johnson, A. D. Becke *J. Chem. Phys.* **2006**, *124*, 174104.
[43]GaussView, Version 6.1, Roy Dennington, Todd A. Keith, and John M. Millam, Semichem Inc., Shawnee Mission, KS, 2016.
[44]G. Knizia **2021,** IboView; see http://www.iboview.org..
